# Supplementary material for: Rhodium hydride enabled enantioselective intermolecular C–H silylation to access acyclic stereogenic Si–H
Source: Nat Commun. 2022 Feb 14;13:847. doi: 10.1038/s41467-022-28439-w (PMC8844420; doi:10.1038/s41467-022-28439-w)
Supplement: Supplementary file 1 — Supplementary Information [file 41467_2022_28439_MOESM1_ESM.pdf]

Supplementary Information for

Rhodium Hydride Enabled Enantioselective Intermolecular C-H Silylation to Access Acyclic Stereogenic Si-H

Kun An,[a] Wenpeng Ma,[a] Li-Chuan Liu,[a] Tao He,[a] Guiyu Guan,[a] Qing-Wei Zhang,[*,b] and Wei He[*,a]

[a]MOE Key Laboratory of Bioorganic Phosphorus Chemistry & Chemical Biology and School of Pharmaceutical Sciences, Tsinghua University, Beijing, P. R. China

[b]Department of Chemistry, University of Science and Technology of China, Hefei, P. R. China

*Correspondence to: qingweiz@ustc.edu.cn; whe@tsinghua.edu.cn

Table of Contents

Supplementary Notes………...S2

Supplementary Methods……………………………………………S2

Supplementary Discussion………………………………………...S57

Supplementary References……...S303

1. Supplementary Notes

Unless otherwise noted, all experiments were manipulated under an atmosphere of argon using standard Schlenk technique or in a nitrogen-filled glovebox. ^{1}H NMR, ^{13}C NMR, ^{19}F NMR, ^{29}Si NMR, and ^{31}P NMR spectra were recorded on Bruker Aescend™ 400M or 500M Spectrometers (^{1}H 400 or 500 MHz, ^{13}C 100 or 125 MHz,^{19}F 376 MHz, ^{29}Si 80 MHz, ^{31}P 162 MHz). Chemical shifts (δ) were quoted in parts per million (ppm) referenced to residual solvent peaks. Optical rotations were measured on a Rudolph Autopl VI polarimeter. High resolution mass spectrometric (HRMS) analyses data were obtained by EI or APCI technique. High pressure liquid chromatography (HPLC) analysis was performed on a Shimadzu SPD-M20A liqiud chromatograph. All organic solvents were dried using standard, published methods and distilled prior for using. All other chemicals were used as received from Sigma-Aldrich, TCI, and Acros without further purification. The ligands of MeO-Biphep derivatives and DTBM-Segphos were purchased from Sinocompound Catalyst Co., Ltd (Jiangsu, China) and used as received. The ligands of DTBM-BINAP and TMS-Segphos were synthesized following reported procedures.[1-2]

2. Supplementary Methods

2.1 Synthesis and Characterization of [Rh]-H Catalyst

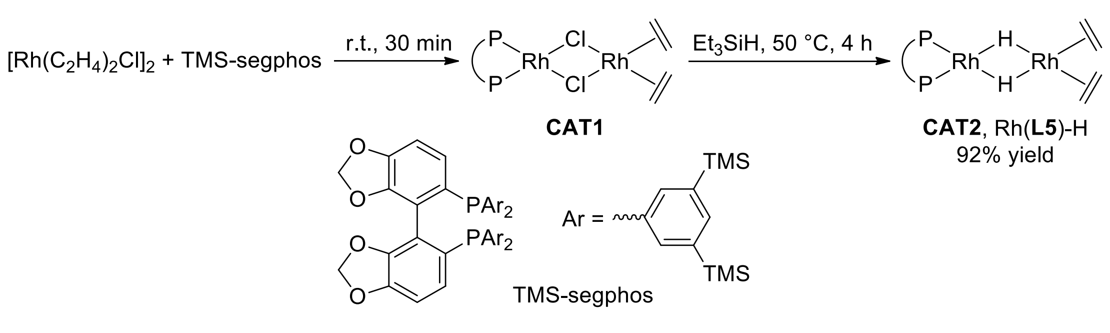

The rhodium hydride catalyst Rh(**L5**)-H was synthesized following reported procedure.[3] We have attempted to isolate Rh(**L5**)-H in pure form, but removing the excess Et_3SiH under vacuum would cause **CAT2** to decompose, and recrystallizing

CAT2 was troubled by its great solubility. Therefore, the pre-formed Rh(**L5**)-H stock solution was used directly without further purification.

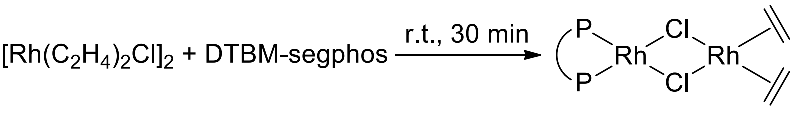

CAT3, quantitative yield

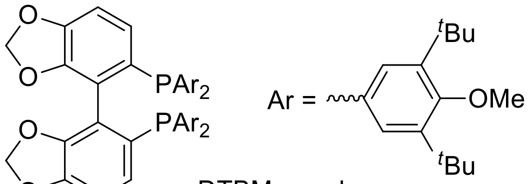

DTBM-segphos

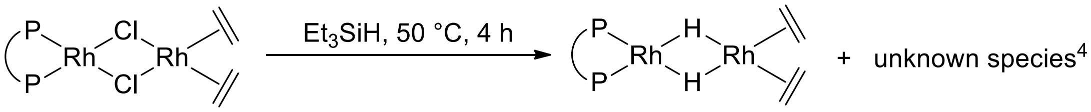

CAT3 **CAT4**, 18% yield

Consistent with our previous observation, the reaction of $[Rh(C_2H_4)_2Cl]_2$ with 2.0 equiv. of DTBM-segphos failed to generate the dimeric $[Rh(DTBM\text{-}segphos)Cl]_2$.[3-4] Therefore, equal equivalent of $[Rh(C_2H_4)_2Cl]_2$ with DTBM-segphos were employed to generate the corresponding [Rh]-Cl complex **CAT3** quantitatively. The detailed synthetic procedure was listed as follows.

$[Rh(C_2H_4)_2Cl]_2$ (7.8 mg, 0.02 mmol, 1.0 equiv.), DTBM-segphos (23.6 mg, 0.02 mmol, 1.0 equiv.) and toluene-d_8 (2.0 mL) were added into a sealed tube in N_2-flushed glovebox. The reaction mixture was stirred at r.t. for 30 minutes and subjected to NMR analysis. ^{1}H NMR (500 MHz, toluene-d_8) δ 8.04-8.02 (m, 4H), 6.97-6.92 (m, 2H), 6.10 (d, J = 5.0 Hz, 2H), 5.36 (d, J = 5.0 Hz, 2H), 5.24 (br s, 4H), 5.06 (s, 2H), 3.53 (s, 6H), 3.44 (s, 6H), 2.67 (br s, 8H), 1.57 (s, 72H). ^{31}P NMR (162 MHz, toluene-d_8) δ 50.9 (d, J_{Rh-P} = 198 Hz). Et_3SiH (32 μL, 0.2 mmol, 10.0 equiv.) was added in one portion to the above prepared [Rh]-Cl solution and the stirring continued for 4 hours at 50 °C. The reaction mixture was subjected to NMR analysis, a characteristic hydride signal [^{1}H NMR (500 MHz, toluene-d_8) δ -7.43 ~ -7.66 (m)] and [^{31}P NMR (162 MHz, toluene-d_8) δ 35.6 (d, J_{Rh-P} = 144 Hz)] were observed corresponding to the [Rh]-H species **CAT4**.

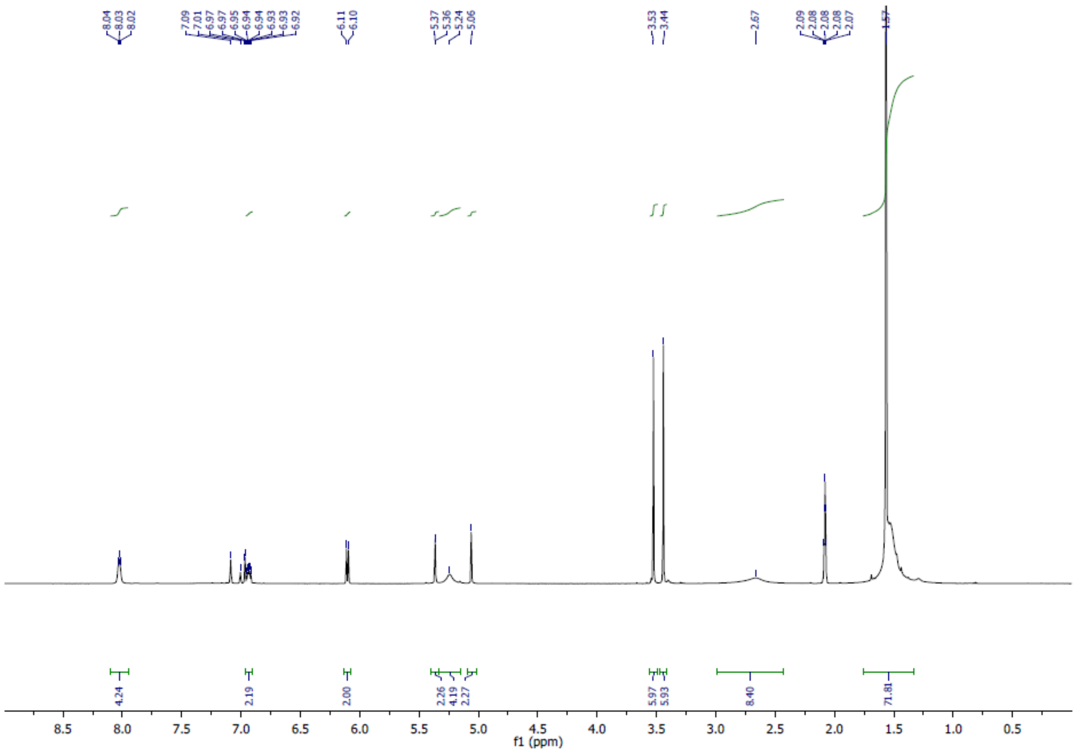

Supplementary Figure 1. ^{1}H NMR spectrum of **CAT3**

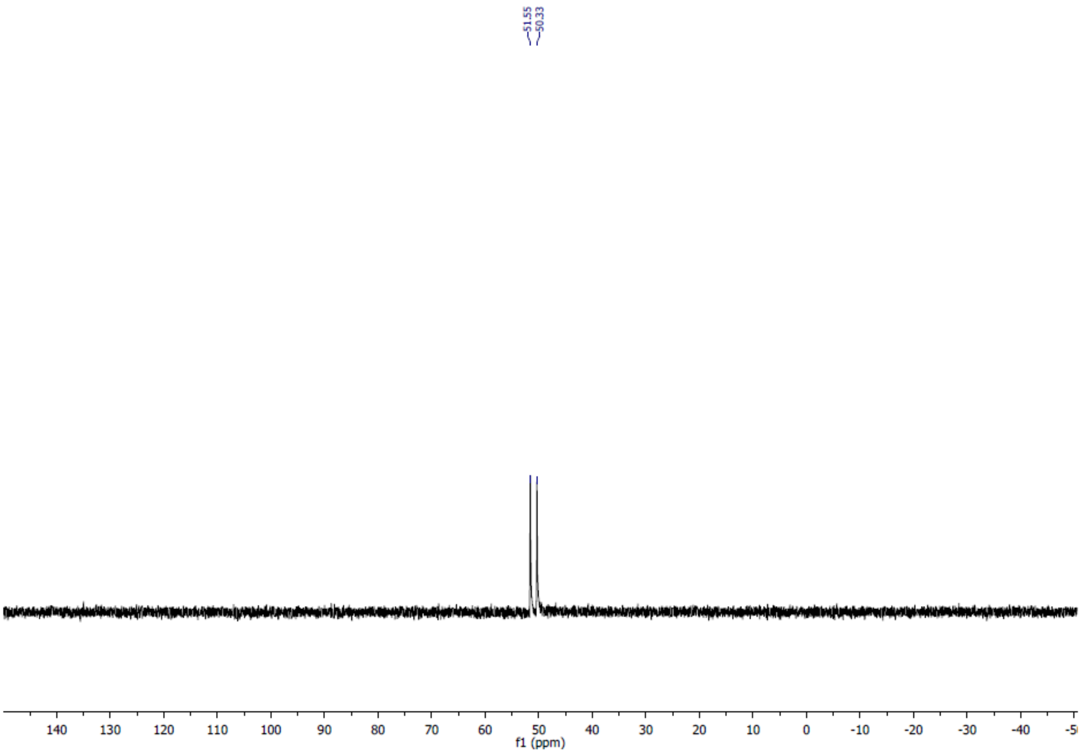

Supplementary Figure 2. ^{31}P NMR spectrum of **CAT3**

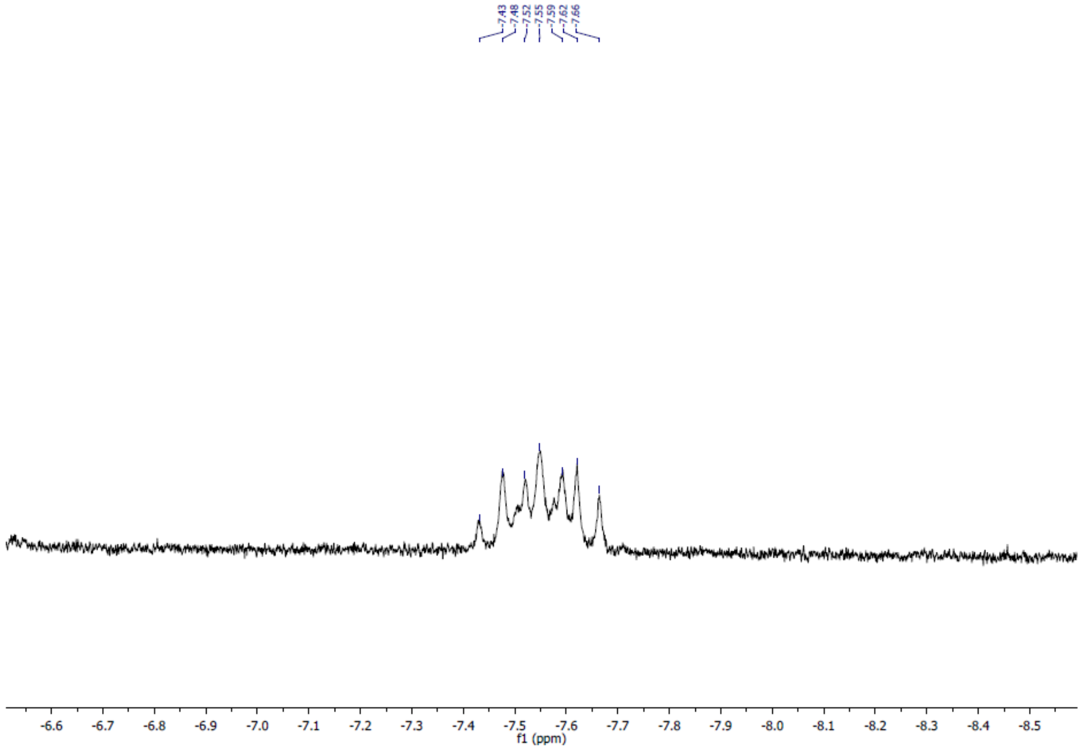

Supplementary Figure 3. Zoom-in ^{1}H NMR spectrum of **CAT4** (hydride region)

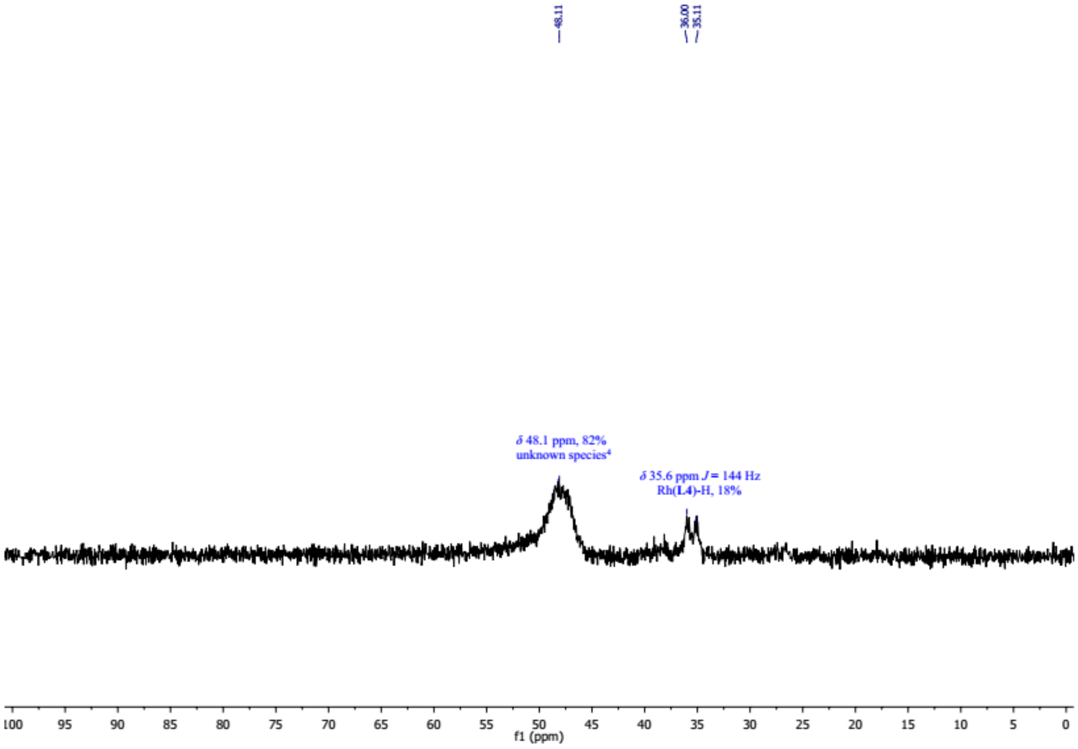

Supplementary Figure 4. ^{31}P NMR spectrum of **CAT4**

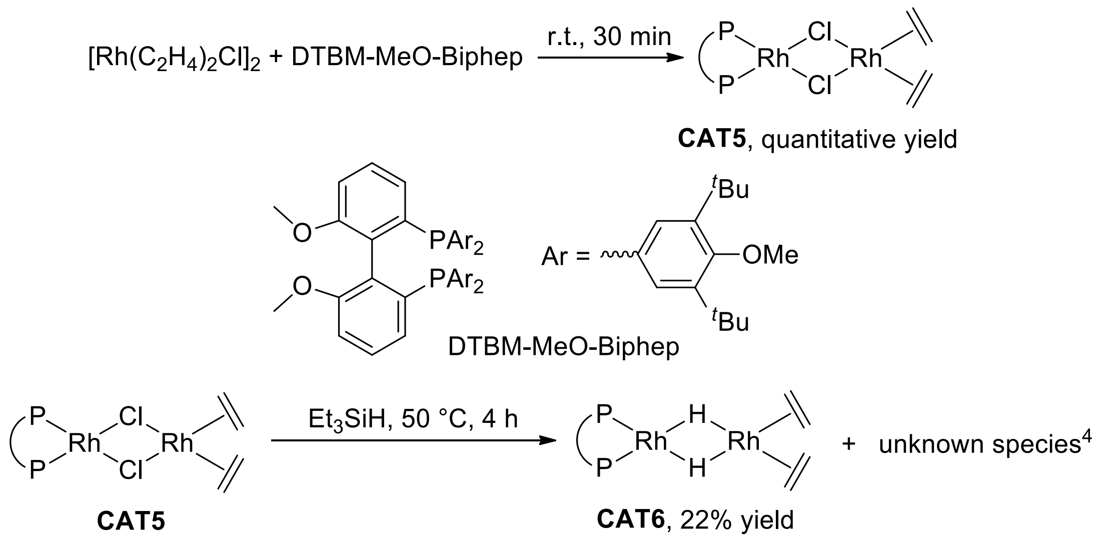

Consistent with our previous observations, the reaction of $[Rh(C_2H_4)_2Cl]_2$ with 2.0 equiv. of DTBM-MeO-biphep failed to generate the dimeric [Rh(DTBM-MeO-biphep)Cl]$_2$. Therefore, equal equivalent of $[Rh(C_2H_4)_2Cl]_2$ with DTBM-MeO-biphep were employed to generate the corresponding [Rh]-Cl complex **CAT5** quantitatively. The detailed synthetic procedure was listed as follows.

$[Rh(C_2H_4)_2Cl]_2$ (7.8 mg, 0.02 mmol, 1.0 equiv.), DTBM-MeO-biphep (23.0 mg, 0.02 mmol, 1.0 equiv.) and toluene-d$_8$ (2.0 mL) were added into a sealed tube in N_2-flushed glove box. The reaction mixture was stirred at r.t. for 30 minutes and subjected to NMR analysis. ^{1}H NMR (500 MHz, toluene-d$_8$) δ 8.13-8.11 (m, 4H), 7.06-7.02 (m, 2H), 6.61-6.58 (m, 2H), 6.06 (d, J = 10.0 Hz, 2H), 5.21 (br s, 4H), 3.49 (s, 6H), 3.43 (s, 6H), 3.19 (s, 6H), 2.63 (br s, 8H), 1.59 (s, 72H). ^{31}P NMR (162 MHz, toluene-d$_8$) δ 52.0 (d, J_{Rh-P} = 200 Hz). Et_3SiH (32 μL, 0.2 mmol, 10.0 equiv.) was added in one portion to the above prepared [Rh]-Cl solution and the stirring continued for 4 hours at 50 °C. The reaction mixture was subjected to NMR analysis, a characteristic hydride signal [^{1}H NMR (500 MHz, toluene-d$_8$) δ -7.43 ~ -7.61 (m)] and [^{31}P NMR (162 MHz, toluene-d$_8$) δ 41.0 (d, J_{Rh-P} = 131 Hz)] were observed corresponding to the [Rh]-H species **CAT6**.

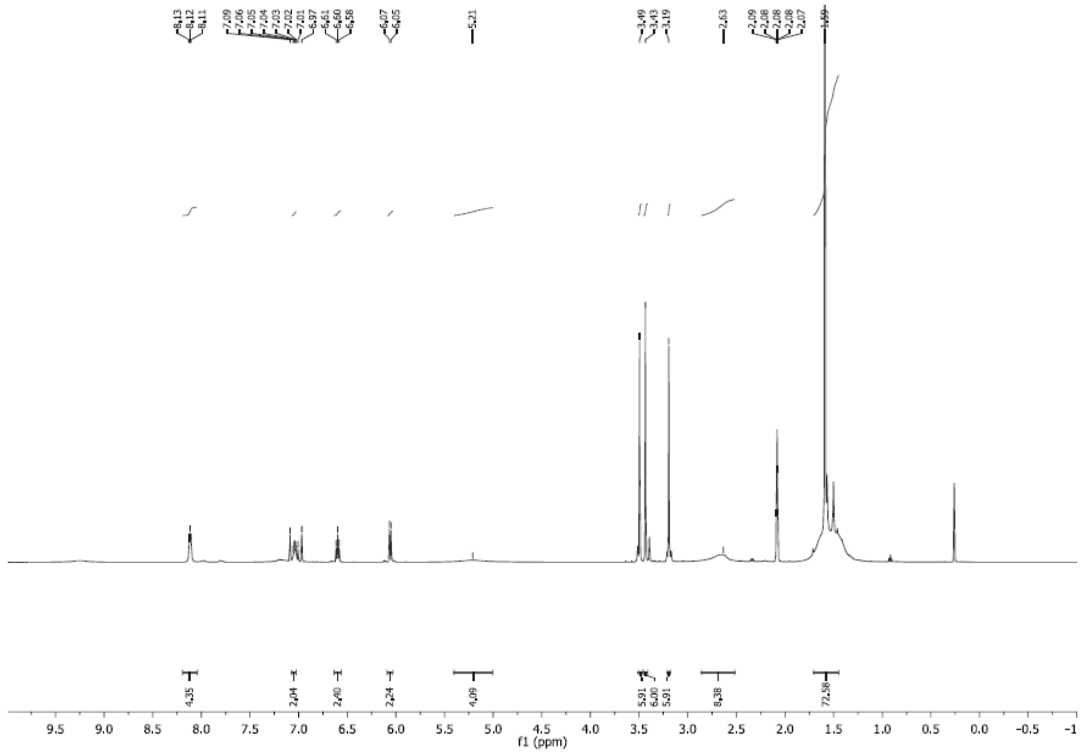

Supplementary Figure 5. ^{1}H NMR spectrum of **CAT5**

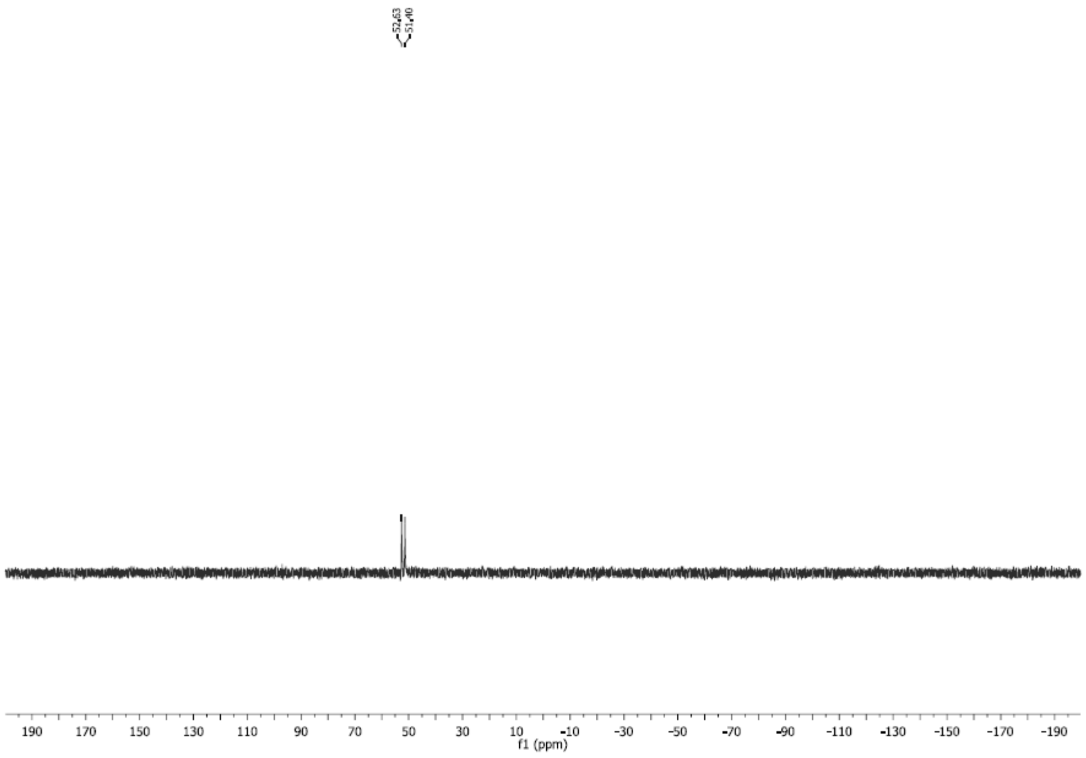

Supplementary Figure 6. ^{31}P NMR spectrum of **CAT5**

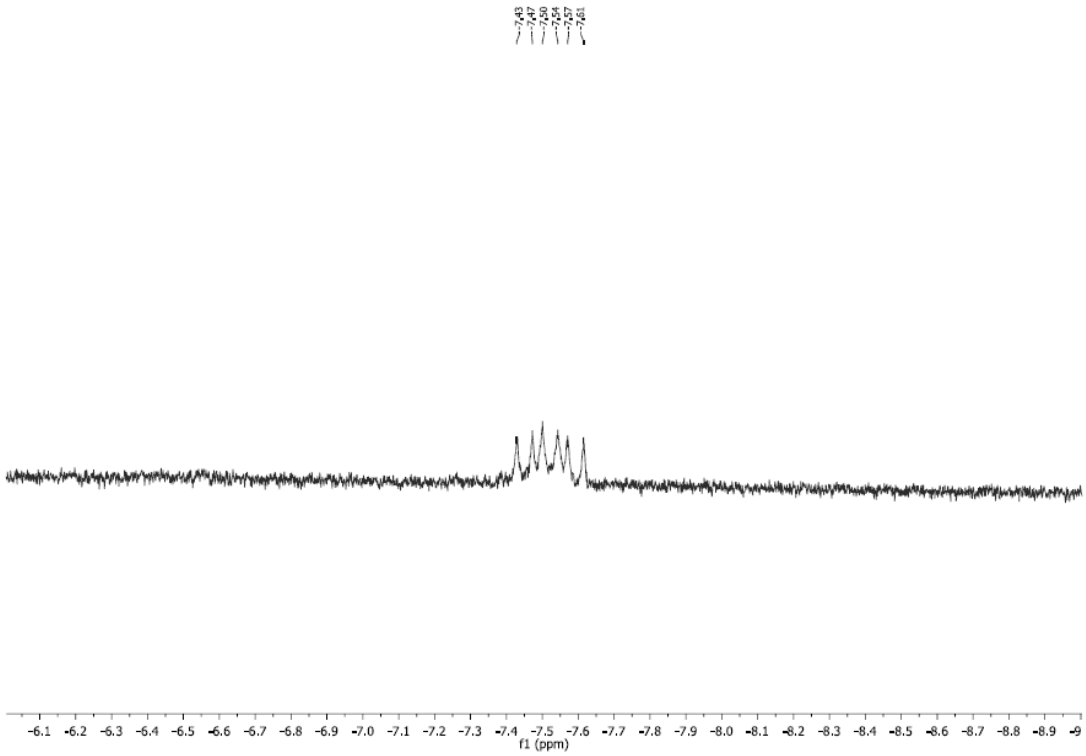

Supplementary Figure 7. Zoom-in ^{1}H NMR spectrum of **CAT6** (hydride region)

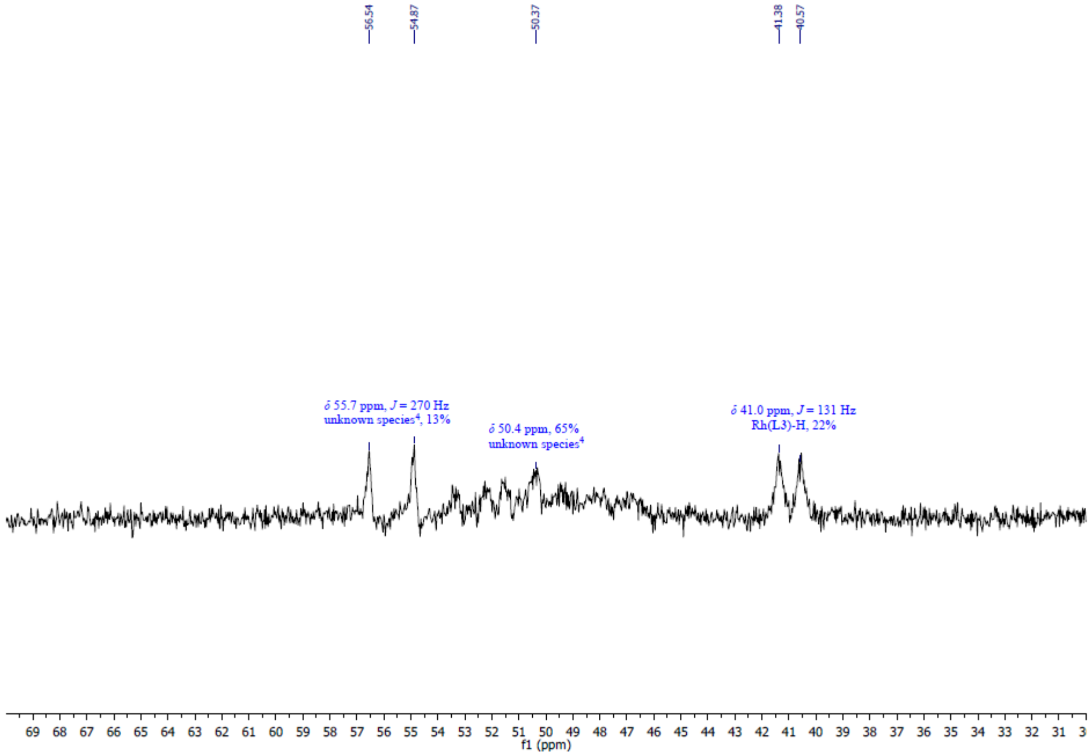

Supplementary Figure 8. ^{31}P NMR spectrum of **CAT6**

2.2 Typical Procedure for the Synthesis of Substrates and Characterization

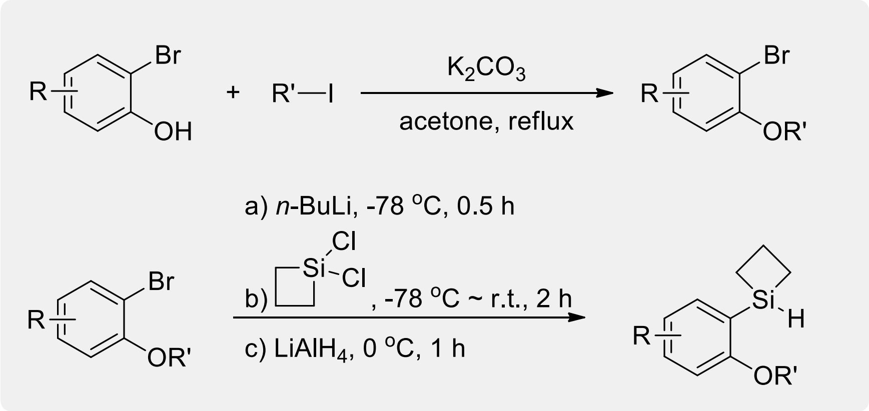

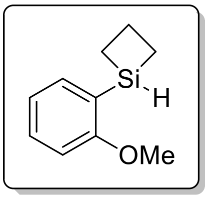

1-(2-methoxyphenyl)siletane (1b)

2-bromophenol (5.54 g, 32 mmol, 1.0 equiv.) and MeI (5.1 g, 36 mmol, 1.1 equiv.) were dissolved in acetone (40.0 mL) into a 100 mL round-bottom flask fitted with a condenser, followed by addition of K_2CO_3 (8.80 g, 64 mmol, 2.0 equiv.). The reaction mixture was stirred at reflux for 12 h, then cooled to r.t. and concentrated. Water (100.0 mL) was added, the aqueous phase was extracted with ethyl acetate and the organic layer was dried over Na_2SO_4, filtered and concentrated under vacuum. The resulting residue was purified by silica gel column chromatography to give 1-bromo-2-methoxybenzene as a colorless oil (5.7 g, 95% yield).

A solution of 1-bromo-2-methoxybenzene (1.49 g, 8 mmol, 1.0 equiv.) in anhydrous THF (40.0 mL) was cooled to -78 °C. ^{n}BuLi (3.8 mL, 2.4 M, 9 mmol, 1.1 equiv.) was added dropwise and the reaction mixture was maintained at this temperature for 0.5 h. 1,1-dichlorosiletane (1.27 g, 9 mmol, 1.1 equiv.) was added in one portion and the mixture was allowed to warm to room temperature for 2 h. The mixture was cooled to 0 °C and a suspension of $LiAlH_4$ (380 mg, 10 mmol, 1.2 equiv.) was carefully added. The reaction mixture was stirred for 1 h, then water (20.0 mL) was added dropwise. The aqueous phase was extracted with ethyl acetate and the organic layer was dried over Na_2SO_4, filtered and concentrated under vacuum. The resulting residue was purified by silica gel column chromatography to give the desired product **1b** as a

colorless oil (926 mg, 65% yield). ^{1}H NMR (400 MHz, $CDCl_3$) δ 7.57 (dd, J_1 = 7.2 Hz, J_2 =1.6 Hz, 1H), 7.45-7.43 (m, 1H), 7.05 (t, J = 7.2 Hz, 1H), 6.90 (d, J = 8.4 Hz, 1H), 5.25 (d, J = 2.4 Hz, 1H), 3.88 (s, 3H), 2.39-2.27 (m, 2H), 1.42-1.38 (m, 4H); ^{13}C NMR (100 MHz, $CDCl_3$) δ 164.4, 136.0, 132.0, 124.0, 120.8, 109.7, 55.5, 19.9, 12.5; ^{29}Si NMR (80 MHz, $CDCl_3$) δ -6.8. HRMS-EI exact mass calcd. for $C_{10}H_{14}OSi^+$ ($[M]^+$) requires m/z 178.0808, found m/z 178.0808.

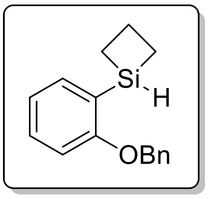

1-(2-(benzyloxy)phenyl)siletane (**1a**)

Colorless oil; isolated yield 66%; ^{1}H NMR (400 MHz, $CDCl_3$) δ 7.58 (dd, J_1 = 7.2 Hz, J_2 = 1.6 Hz, 1H), 7.48-7.40 (m, 5H), 7.37-7.35 (m, 1H), 7.05 (t, J = 7.2 Hz, 1H), 6.94 (d, J = 8.4 Hz, 1H), 5.30-5.29 (m, 1H), 5.15 (s, 2H), 2.29-2.21 (m, 2H), 1.43-1.35 (m, 4H); ^{13}C NMR (100 MHz, $CDCl_3$) δ 163.4, 137.2, 136.2, 132.0, 128.6, 127.9, 127.2, 124.4, 121.1, 110.8, 69.9, 19.8, 12.5; ^{29}Si NMR (80 MHz, $CDCl_3$) δ -6.6. HRMS-EI exact mass calcd. for $C_{16}H_{18}OSi^+$ ($[M]^+$) requires m/z 254.1121, found m/z 254.1121.

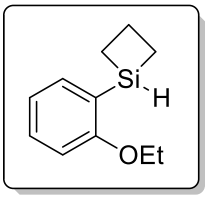

1-(2-ethoxyphenyl)siletane (**1c**)

Colorless oil; isolated yield 68%; ^{1}H NMR (400 MHz, $CDCl_3$) δ 7.52 (dd, J_1 = 7.2 Hz, J_2 =1.6 Hz, 1H), 7.42-7.38 (m, 1H), 6.99 (t, J = 7.2 Hz, 1H), 6.85 (d, J = 8.4 Hz, 1H), 5.20-5.18 (m, 1H), 4.08 (q, J = 7.2 Hz, 2H), 2.38-2.20 (m, 2H), 1.46-1.39 (m, 7H); ^{13}C NMR (100 MHz, $CDCl_3$) δ 163.9, 136.1, 131.9, 124.1, 120.7, 110.4, 63.7, 19.9, 14.9, 12.6; ^{29}Si NMR (80 MHz, $CDCl_3$) δ -6.1. HRMS-EI exact mass calcd. for $C_{11}H_{16}OSi^+$ ($[M]^+$) requires m/z 192.0965, found m/z 192.0964.

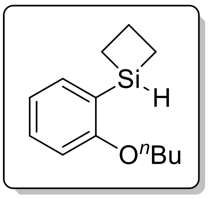

1-(2-butoxyphenyl)siletane (**1d**)

Colorless oil; isolated yield 69%; ^{1}H NMR (400 MHz, $CDCl_3$) δ 7.55 (dd, J_1 = 7.2 Hz, J_2 = 1.6 Hz, 1H), 7.44-7.40 (m, 1H), 7.02 (t, J = 7.2 Hz, 1H), 6.88 (d, J = 8.4 Hz, 1H), 5.24-5.22 (m, 1H), 4.03 (t, J = 6.4 Hz, 2H), 2.28-2.22 (m, 2H), 1.86-1.80 (m, 2H), 1.61-1.53 (m, 2H), 1.45-1.37 (m, 4H), 1.03 (t, J = 7.6 Hz, 3H); ^{13}C NMR (100 MHz, $CDCl_3$) δ 164.0, 136.0, 131.9, 124.0, 120.6, 110.2, 67.6, 31.5, 19.8, 19.4, 14.0, 12.6; ^{29}Si NMR (80 MHz, $CDCl_3$) δ -6.5.

HRMS-EI exact mass calcd. for $C_{13}H_{20}OSi^{+}$ ($[M]^{+}$) requires m/z 220.1278, found m/z 220.1277.

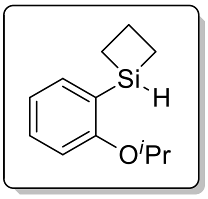

1-(2-isopropoxyphenyl)siletane (1e)

Colorless oil; isolated yield 68%; ^{1}H NMR (400 MHz, $CDCl_3$) δ 7.51 (dd, J_1 = 7.2 Hz, J_2 = 1.6 Hz, 1H), 7.40-7.36 (m, 1H), 6.97 (td, J_1 = 7.2 Hz, J_2 = 0.8 Hz, 1H), 6.86 (d, J = 8.0 Hz, 1H), 5.19-5.15 (m, 1H), 4.66-4.60 (m, 1H), 2.37-2.17 (m, 2H), 1.43-1.32 (m, 10H); ^{13}C NMR (100 MHz, $CDCl_3$) δ 162.8, 136.3, 131.8, 124.9, 120.4, 111.5, 69.8, 22.2, 19.8, 12.6; ^{29}Si NMR (80 MHz, $CDCl_3$) δ -6.2. HRMS-EI exact mass calcd. for $C_{12}H_{18}OSi^{+}$ ($[M]^{+}$) requires m/z 206.1121, found m/z 206.1120.

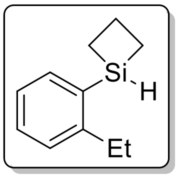

1-(2-ethylphenyl)siletane (1f)

Colorless oil; isolated yield 70%; ^{1}H NMR (400 MHz, $CDCl_3$) δ 7.63 (d, J = 7.7 Hz, 1H), 7.42 (t, J = 7.5 Hz, 1H), 7.28 (d, J = 6.8 Hz, 2H), 5.34 (s, 1H), 2.79 (q, J = 7.6 Hz, 2H), 2.37-2.19 (m, 2H), 1.43-1.40 (m, 4H), 1.28 (t, J = 7.5 Hz, 3H); ^{13}C NMR (100 MHz, $CDCl_3$) δ 150.1, 135.0, 134.3, 130.4, 127.8, 125.4, 29.5, 19.6, 16.2, 12.9; ^{29}Si NMR (80 MHz, $CDCl_3$) δ -8.4. HRMS-APCI exact mass calcd. for $C_{11}H_{15}Si^{+}$ ($[M-H]^{+}$) requires m/z 175.0938, found m/z 175.0977.

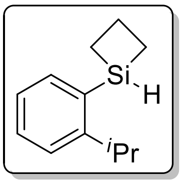

1-(2-isopropylphenyl)siletane (1g)

Colorless oil; isolated yield 74%; ^{1}H NMR (400 MHz, $CDCl_3$) δ 7.63 (d, J = 7.2 Hz, 1H), 7.48 (t, J = 7.6 Hz, 1H), 7.38 (d, J = 7.7 Hz, 1H), 7.29 (t, J = 7.4 Hz, 1H), 5.38 (s, 1H), 3.17-3.10 (m, 1H), 2.39-2.22 (m, 2H), 1.47-1.42 (m, 4H), 1.34 (d, J = 6.9 Hz, 6H); ^{13}C NMR (100 MHz, $CDCl_3$) δ 154.9, 134.9, 133.9, 130.6, 125.6, 124.7, 34.6, 24.3, 19.5, 13.1; ^{29}Si NMR (80 MHz, $CDCl_3$) δ -8.6. HRMS-APCI exact mass calcd. for $C_{12}H_{17}Si^{+}$ ($[M-H]^{+}$) requires m/z 189.1094, found m/z 189.0922.

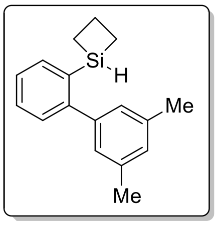

1-(3',5'-dimethyl-[1,1'-biphenyl]-2-yl)siletane (1h)

Colorless oil; isolated yield 62%; ^{1}H NMR (400 MHz, $CDCl_3$) δ 7.74 (dd, J_1 = 7.2 Hz, J_2 = 1.6 Hz, 1H), 7.50-7.46 (m, 1H), 7.42-7.38 (m, 2H), 7.01-7.00 (m, 3H), 4.86-4.85 (m, 1H), 2.37 (s, 6H), 2.22-2.15 (m, 1H), 2.10-2.02 (m, 1H), 1.25-1.20 (m, 4H); ^{13}C NMR (100

MHz, $CDCl_3$) δ 149.2, 143.2, 137.9, 135.2, 134.9, 130.0, 129.0, 128.7, 126.6, 126.5, 21.5, 18.9, 14.0; ^{29}Si NMR (80 MHz, $CDCl_3$) δ -8.1. HRMS-EI exact mass calcd. for $C_{17}H_{20}Si^+$ ($[M]^+$) requires m/z 252.1329, found m/z 252.1328.

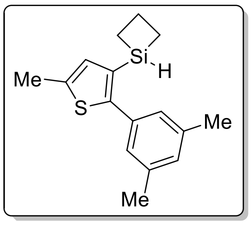

1-(2-(3,5-dimethylphenyl)-5-methylthiophen-3-yl)siletane (**1i**)

Colorless oil; isolated yield 70%; ^{1}H NMR (400 MHz, $CDCl_3$) δ 7.05 (s, 2H), 6.96 (s, 1H), 6.91 (s, 1H), 5.05 (t, J = 3.0 Hz, 1H), 2.53 (s, 3H), 2.34 (s, 6H), 2.27-2.13 (m, 3H), 1.32-1.24 (m, 4H); ^{13}C NMR (100 MHz, $CDCl_3$) δ 151.1, 139.7, 138.1, 135.7, 132.7, 132.0, 129.4, 126.4, 21.4, 19.5, 15.1, 13.8; ^{29}Si NMR (80 MHz, $CDCl_3$) δ -14.2. HRMS-APCI exact mass calcd. for $C_{16}H_{19}SSi^+$ ($[M-H]^+$) requires m/z 271.0971, found m/z 271.0983.

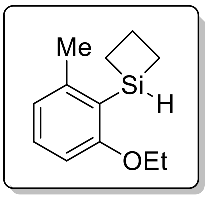

1-(2-ethoxy-6-methylphenyl)siletane (**1j**)

Colorless oil; isolated yield 65%; ^{1}H NMR (400 MHz, $CDCl_3$) δ 7.28 (t, J = 8.0 Hz, 1H), 6.81 (d, J = 7.6 Hz, 1H), 6.71 (d, J = 8.0 Hz, 1H), 5.22-5.18 (m, 1H), 4.05 (q, J = 7.2 Hz, 2H), 2.42 (s, 3H), 2.41-2.32 (m, 1H), 2.17-2.09 (m, 1H), 1.52 -1.39 (m, 7H); ^{13}C NMR (100 MHz, $CDCl_3$) δ 164.1, 145.5, 131.2, 122.9, 122.8, 107.9, 63.7, 22.3, 20.0, 15.5, 14.8; ^{29}Si NMR (80 MHz, $CDCl_3$) δ -14.5. HRMS-EI exact mass calcd. for $C_{12}H_{18}OSi^+$ ($[M]^+$) requires m/z 206.1121, found m/z 206.1121.

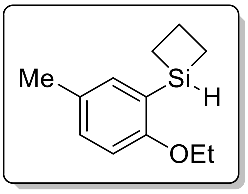

1-(2-ethoxy-5-methylphenyl)siletane (**1k**)

Colorless oil; isolated yield 61%; ^{1}H NMR (400 MHz, $CDCl_3$) δ 7.33 (d, J = 2.4 Hz, 1H), 7.20 (dd, J_1 = 8.4 Hz, J_2 = 1.6 Hz, 1H), 6.77 (d, J = 8.4 Hz, 1H), 5.21-5.17 (m, 1H), 4.06 (q, J = 6.8 Hz, 2H), 2.40-2.32 (m, 4H), 2.27-2.17 (m, 1H), 1.47-1.31 (m, 7H);^{13}C NMR (100 MHz, $CDCl_3$) δ 161.9, 136.6, 132.3, 129.7, 124.0, 110.5, 63.8, 20.6, 19.8, 14.9, 12.6; ^{29}Si NMR (80 MHz, $CDCl_3$) δ -6.2. HRMS-EI exact mass calcd. for $C_{12}H_{18}OSi^+$ ($[M]^+$) requires m/z 206.1121, found m/z 206.1120.

1-(2-ethoxy-4-methylphenyl)siletane (1l)

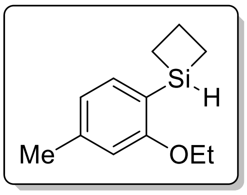

Colorless oil; isolated yield 69%; ^{1}H NMR (400 MHz, $CDCl_3$) δ 7.42-7.38 (m, 1H), 6.83-6.80 (m, 1H), 6.69-6.67 (m, 1H), 5.18-5.13 (m, 1H), 4.07 (q, J = 7.2 Hz, 2H), 2.38-2.18 (m, 5H), 1.46-1.32 (m, 7H); ^{13}C NMR (100 MHz, $CDCl_3$) δ 164.1, 142.4, 136.0, 121.5, 120.6, 111.5, 63.6, 22.0, 19.8, 14.9, 12.7; ^{29}Si NMR (80 MHz, $CDCl_3$) δ -6.3. HRMS-EI exact mass calcd. for $C_{12}H_{18}OSi^+$ ($[M]^+$) requires m/z 206.1121, found m/z 206.1121.

1-(2-ethoxy-3-methylphenyl)siletane (1m)

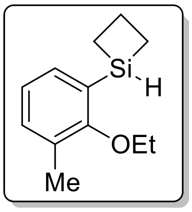

Colorless oil; isolated yield 64%; ^{1}H NMR (400 MHz, $CDCl_3$) δ 7.42 (dd, J_1 = 7.2 Hz, J_2 = 1.6 Hz, 1H), 7.31-7.29 (m, 1H), 7.12 (t, J = 7.6 Hz, 1H), 5.30-3.27 (m, 1H), 3.94 (q, J = 7.2 Hz, 2H), 2.34 (s, 3H), 2.32 -2.21 (m, 1H), 1.44 (t, J = 7.2 Hz, 3H), 1.42-1.36 (m, 4H);^{13}C NMR (100 MHz, $CDCl_3$) δ 162.5, 134.0, 133.5, 130.3, 128.9, 124.2, 69.3, 19.7, 16.4, 15.7, 12.8; ^{29}Si NMR (80 MHz, $CDCl_3$) δ -9.3. HRMS-EI exact mass calcd. for $C_{12}H_{18}OSi^+$ ($[M]^+$) requires m/z 206.1121, found m/z 206.1122.

1-(2-ethoxy-4-fluorophenyl)siletane (1n)

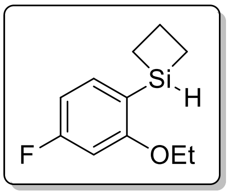

Colorless oil; isolated yield 70%; ^{1}H NMR (400 MHz, $CDCl_3$) δ 7.44 (t, J = 7.6 Hz, 1H), 6.68 (td, J_1 = 8.4 Hz, J_2 = 2.0 Hz, 1H), 6.56 (dd, J = 11.6 Hz, J_2 = 2.0 Hz, 1H), 5.15-5.12 (m, 1H), 4.04 (q, J = 7.2 Hz, 2H), 2.36-2.15 (m, 2H), 1.44 (t, J = 7.2 Hz, 3H), 1.40-1.30 (m, 4H); ^{13}C NMR (100 MHz, $CDCl_3$) δ 166.3 (d, J = 173 Hz), 165.0 (d, J = 63 Hz), 137.2 (d, J = 10 Hz), 119.4, 107.3 (d, J = 20 Hz), 98.9 (d, J = 25 Hz), 64.0, 19.8, 14.7, 12.6; ^{19}F NMR (376 MHz, $CDCl_3$) δ -108.0; ^{29}Si NMR (80 MHz, $CDCl_3$) δ -6.3. HRMS-EI exact mass calcd. for $C_{11}H_{15}FOSi^+$ ($[M]^+$) requires m/z 210.0871, found m/z 210.0869.

1-(4-chloro-2-ethoxyphenyl)siletane (1o)

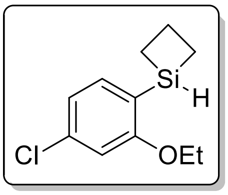

Colorless oil; isolated yield 71%; ^{1}H NMR (400 MHz, $CDCl_3$) δ 7.42 (d, J = 7.6 Hz, 1H), 6.97 (dd, J_1 = 7.6 Hz, J_2 = 1.6 Hz, 1H), 6.83 (d, J = 1.6 Hz, 1H), 5.17-5.14 (m, 1H), 4.05 (q, J = 7.2 Hz, 2H), 2.34-2.19 (m, 2H), 1.46-1.34 (m, 7H); ^{13}C NMR (100 MHz, $CDCl_3$) δ 164.5, 137.6, 136.8, 122.5, 120.8, 111.2, 64.0, 19.9, 14.7, 12.5; ^{29}Si NMR (80 MHz, $CDCl_3$) δ -6.2.

HRMS-EI exact mass calcd. for $C_{11}H_{15}ClOSi^+$ ($[M]^+$) requires m/z 226.0575, found m/z 226.0574.

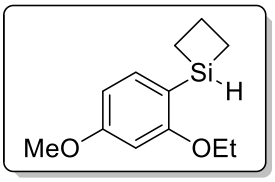

1-(2-ethoxy-4-methoxyphenyl)siletane (1p)

Colorless oil; isolated yield 70%; ^{1}H NMR (400 MHz, $CDCl_3$) δ 7.41 (d, J = 8.0 Hz, 1H), 6.51 (dd, J_1 = 8.0 Hz, J_2 = 2.0 Hz, 1H), 6.41 (d, J = 2.4 Hz, 1H), 5.11-5.10 (m, 1H), 4.04 (q, J = 7.2 Hz, 2H), 3.82 (s, 3H), 2.32-2.28 (m, 1H), 2.20-2.12 (m, 1H), 1.44-1.25 (m, 7H); ^{13}C NMR (100 MHz, $CDCl_3$) δ 165.4, 163.4, 137.1, 115.5, 104.8, 98.4, 55.4, 19.7, 14.8, 12.8; ^{29}Si NMR (80 MHz, $CDCl_3$) δ -6.3. HRMS-EI exact mass calcd. for $C_{12}H_{18}O_2Si^+$ ($[M]^+$) requires m/z 222.1071, found m/z 222.1070.

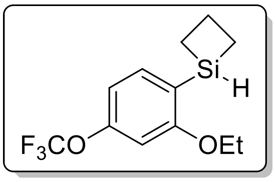

1-(2-ethoxy-4-(trifluoromethoxy)phenyl)siletane (1q).

Colorless oil; isolated yield 68%; ^{1}H NMR (400 MHz, $CDCl_3$) δ 7.49 (d, J = 8.0 Hz, 1H), 6.85-6.82 (m, 1H), 6.66 (d, J = 1.2 Hz, 1H), 5.17-5.13 (m, 1H), 4.05 (q, J = 7.2 Hz, 2H), 2.33-2.18 (m, 2H), 1.44 (t, J = 7.2 Hz, 3H), 1.40-1.32 (m, 4H); ^{13}C NMR (100 MHz, $CDCl_3$) δ 164.9, 152.4 (d, J = 2.0 Hz), 137.0, 122.7, 120.6 (q, J = 256 Hz), 112.4, 103.7, 64.1, 19.9, 14.6, 12.4; ^{19}F NMR (376 MHz, $CDCl_3$) δ -57.6; ^{29}Si NMR (80 MHz, $CDCl_3$) δ -6.3. HRMS-EI exact mass calcd. for $C_{12}H_{15}O_2F_3Si^+$ ($[M]^+$) requires m/z 276.0788, found m/z 276.0788.

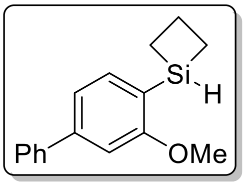

1-(3-methoxy-[1,1'-biphenyl]-4-yl)siletane (1r)

Colorless oil; isolated yield 58%; ^{1}H NMR (400 MHz, $CDCl_3$) δ 7.69-7.64 (m, 3H), 7.54-7.50 (m, 2H), 7.46- 7.42 (m, 1H), 7.29 (dd, J_1 = 7.6 Hz, J_2 =1.6 Hz, 1H), 7.13 (d, J = 1.6 Hz, 1H), 5.32-5.29 (m, 1H), 3.97 (s, 3H), 2.43-2.28 (m, 2H), 1.48-1.41 (m, 4H); ^{13}C NMR (100 MHz, $CDCl_3$) δ 164.9, 145.4, 141.4, 136.5, 128.9, 127.8, 127.4, 122.7, 119.8, 108.7, 55.6, 20.0, 12.5; ^{29}Si NMR (80 MHz, $CDCl_3$) δ -6.9. HRMS-EI exact mass calcd. for $C_{16}H_{18}OSi^+$ ($[M]^+$) requires m/z 254.1121, found m/z 254.1123.

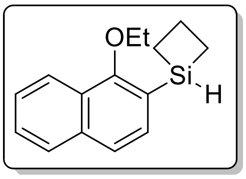

1-(1-ethoxynaphthalen-2-yl)siletane (1s)

Colorless oil; isolated yield 67%; ^{1}H NMR (400 MHz, $CDCl_3$) δ 8.00 (d, *J* = 8.4 Hz, 1H), 7.89 (d, *J* = 9.2 Hz, 1H), 7.81-7.79 (m, 1H), 7.45 (t, *J* = 7.6 Hz, 1H), 7.35 (t, *J* = 7.2 Hz, 1H), 7.21 (d, *J* = 9.2 Hz, 1H), 5.45-5.41 (m, 1H), 4.18 (q, *J* = 7.2 Hz, 2H), 2.44-2.39 (m, 1H), 2.24-2.16 (m, 1H), 1.58-1.54 (m, 4H), 1.44 (t, *J* = 7.2 Hz, 3H); ^{13}C NMR (100 MHz, $CDCl_3$) δ 162.6, 138.2, 132.6, 129.3, 128.7, 126.6, 126.5, 123.5, 117.5, 113.5, 64.8, 20.3, 15.6, 15.1; ^{29}Si NMR (80 MHz, $CDCl_3$) δ -15.0. HRMS-EI exact mass calcd. for $C_{15}H_{18}OSi^+$ ($[M]^+$) requires m/z 242.1121, found m/z 242.1122.

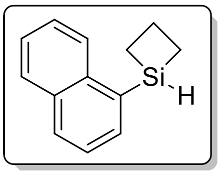

1-(naphthalen-1-yl)siletane (1t)

Colorless oil; isolated yield 68%; ^{1}H NMR (400 MHz, $CDCl_3$) δ 8.21 (s, 1H), 7.91-7.86 (m, 3H), 7.75 (dd, J_1 = 8.2 Hz, J_2 = 1.2 Hz, 1H), 7.56-7.51 (m, 2H), 5.37-5.35 (m, 1H), 2.45-2.29 (m, 2H), 1.47-1.42 (m, 4H); ^{13}C NMR (100 MHz, $CDCl_3$) δ 135.6, 134.2, 133.3, 133.1, 130.3, 128.3, 127.9, 127.5, 126.9, 126.3, 20.0, 13.0; ^{29}Si NMR (80 MHz, $CDCl_3$) δ -1.6. HRMS-EI exact mass calcd. for $C_{13}H_{14}Si^+$ ($[M]^+$) requires m/z 198.0859, found m/z 198.0859.

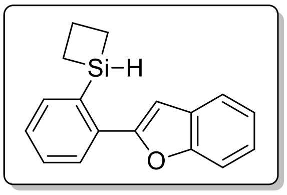

2-(2-(siletan-1-yl)phenyl)benzofuran (1v)

Colorless oil; isolated yield 73%; ^{1}H NMR (400 MHz, $CDCl_3$) δ 7.88-7.83 (m, 2H), 7.65 (d, *J* = 7.4 Hz, 1H), 7.55 (t, *J* = 7.4 Hz, 2H), 7.46 (t, *J* = 7.4 Hz, 1H), 7.37-7.28 (m, 2H), 7.04 (s, 1H), 5.42 (s, 1H), 2.30-2.23 (m, 2H), 1.57-1.42 (m, 4H); ^{13}C NMR (100 MHz, $CDCl_3$) δ 157.4, 154.7, 136.1, 135.5, 134.1, 130.0, 129.2, 128.1, 126.3, 124.5, 123.3, 121.3, 111.2, 102.3, 18.6, 14.6; ^{29}Si NMR (80 MHz, $CDCl_3$) δ -7.8. HRMS-APCI exact mass calcd. for $C_{17}H_{15}OSi^+$ ($[M-H]^+$) requires m/z 263.0887, found m/z 263.0897.

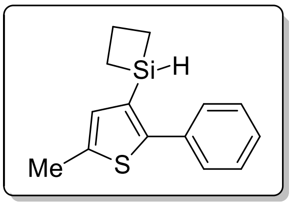

1-(5-methyl-2-phenylthiophen-3-yl)siletane (1w)

Colorless oil; isolated yield 67%; ^{1}H NMR (400 MHz, $CDCl_3$) δ 7.44 (d, *J* = 8.1 Hz, 2H), 7.39 (t, *J* = 7.1 Hz, 2H), 7.34 (d, *J* = 7.7 Hz, 1H), 6.95 (s, 1H), 5.09 (t, *J* = 3.0 Hz, 1H), 2.56 (s, 3H), 2.30– 2.11 (m, 2H), 1.36-1.19 (m, 4H); ^{13}C NMR (100 MHz, $CDCl_3$) δ 150.7, 140.1, 135.9, 133.0, 132.1, 128.6, 128.6, 127.7, 19.7, 15.1, 13.6; ^{29}Si NMR (80

MHz, $CDCl_3$) δ -14.0. HRMS-APCI exact mass calcd. for $C_{14}H_{15}SSi^+$ ($[M-H]^+$) requires m/z 243.0658, found m/z 243.0668.

2.3 Typical Procedure for the Synthesis of Monohydrosilanes and Characterization

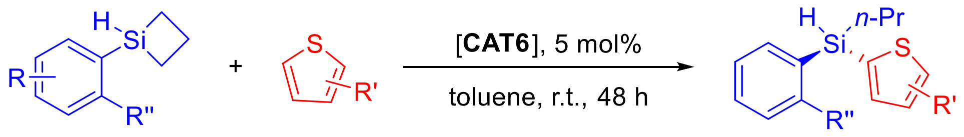

1 **2** **3**

$[Rh(C_2H_4)_2Cl]_2$ (7.8 mg, 0.02 mmol, 1.0 equiv.), DTBM-MeO-biphep (23.0 mg, 0.02 mmol, 1.0 equiv.) and toluene (2.0 mL) were added into a sealed tube in N_2-flushed glove box. The reaction mixture was stirred at r.t. for 30 minutes. Then Et_3SiH (32 μL, 0.2 mmol, 10.0 equiv.) was added in one portion and the stirring continued for 4 hours at 50 °C to afford the stock solution of **CAT6**. Silacyclobutane substrate **1** (0.1 mmol), thiophene **2** (0.2 mmol) and **CAT6** stock solution (0.5 mL) were added into a sealed tube equipped with magnetic stirring bar in N_2-flushed glove box, and the total volume of toluene solution was adjusted to be 1.0 mL. The tube was removed from glovebox and stirred at r.t. for 48 h. The reaction mixture was diluted with dichloromethane (2.0 mL), and the organic layer was concentrated under reduced pressure. The residue was purified by pre-TLC to afford the corresponding monohydrosilane **3**.

(2-(benzyloxy)phenyl)(5-methylthiophen-2-yl)(propyl)silane (3aa)

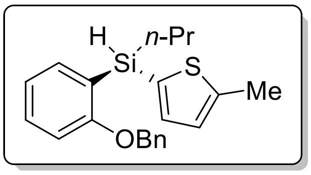

Colorless oil; isolated yield 57%, 92% ee; $[\alpha]_D^{22}$ = +170 (*c* = 1.0, DCM); ^1H NMR (400 MHz, $CDCl_3$) δ 7.45 (dd, J_1 = 7.2 Hz, J_2 = 2.0 Hz, 1H), 7.36-7.30 (m, 6H), 7.12 (d, J = 3.6 Hz, 1H), 6.95 (t, J = 7.2 Hz, 1H), 6.87 (d, J = 8.4 Hz, 1H), 6.82 (d, J = 2.8 Hz, 1H), 5.10 (s, 2H), 4.96 (t, J = 3.6 Hz, 1H), 2.51 (s, 3H), 1.50-1.41 (m, 2H), 1.16-1.11 (m, 2H), 0.93 (t, J = 7.2 Hz, 3H); ^{13}C NMR (100 MHz, $CDCl_3$) δ 163.4, 146.4, 137.2, 137.1, 137.0, 131.7, 131.6, 128.6, 127.9, 127.5, 126.9, 123.5, 121.0, 110.8, 70.0, 18.3, 17.8, 15.7, 15.3; ^{29}Si NMR (80 MHz, $CDCl_3$) δ -23.1. HRMS-EI exact mass calcd. for $C_{21}H_{24}OSSi^+$ ($[M]^+$) requires m/z 352.1212, found m/z 352.1308.

The enantiomeric excess was determined by HPLC on Chiralcel OD-H column (hexane :

isopropanol = 100 : 0, flowing rate = 0.4 mL/min, 35 °C, UV detection at λ = 245 nm) t_{R1} = 44.9 min (minor), t_{R2} = 53.2 min (major).

(2-(benzyloxy)phenyl)(5-ethylthiophen-2-yl)(propyl)silane (3ab)

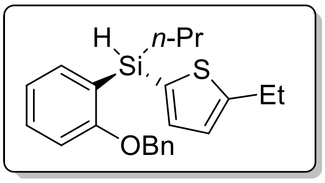

Colorless oil; isolated yield 63%, 90% ee; $[\alpha]_D^{22}$ = +0.4 (*c* = 1.0, DCM); ^{1}H NMR (400 MHz, $CDCl_3$) *δ* 7.47 (d, *J* = 7.2 Hz, 1H), 7.36 (d, *J* = 4.8 Hz, 6H), 7.15 (d, *J* = 3.2 Hz, 1H), 6.96 (t, *J* = 7.2 Hz, 1H), 6.88 (d, *J* = 8.2 Hz, 1H), 6.85 (d, *J* = 3.2 Hz, 1H), 5.11 (s, 2H), 4.98 (t, *J* = 3.8 Hz, 1H), 2.88 (q, *J* = 7.6 Hz, 2H), 1.47 (q, *J* = 7.6 Hz, 2H), 4.98 (t, *J* = 7.2 Hz, 3H), 1.18-1.11 (m, 2H), 0.95 (t, *J* = 7.2 Hz, 3H); ^{13}C NMR (100 MHz, $CDCl_3$) *δ* 163.4, 154.1, 137.2, 137.2, 136.8, 131.7, 131.0, 128.5, 127.9, 127.5, 125.0, 123.5, 121.0, 110.8, 70.0, 23.5, 18.3, 17.8, 16.1, 15.7; ^{29}Si NMR (80 MHz, $CDCl_3$) *δ* -22.9. HRMS-EI exact mass calcd. for $C_{22}H_{26}OSSi^+$ ($[M]^+$) requires m/z 366.1468, found m/z 366.1465.

The enantiomeric excess was determined by HPLC on Chiralcel OD-H column (hexane : isopropanol = 99.9 : 0.1, flowing rate = 1.0 mL/min, 35 °C, UV detection at λ = 249 nm) t_{R1} = 13.0 min (major), t_{R2} = 15.2 min (minor).

(2-(benzyloxy)phenyl)(propyl)(5-propylthiophen-2-yl)silane (3ac)

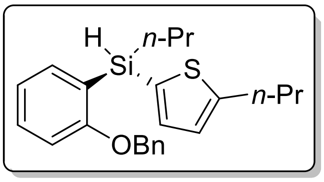

Colorless oil; isolated yield 63%, 88% ee; $[\alpha]_D^{22}$ = -10.5 (*c* = 1.0, DCM); ^{1}H NMR (400 MHz, $CDCl_3$) *δ* 7.46 (d, *J* = 7.2 Hz, 1H), 7.38-7.26 (m, 6H), 7.14 (d, *J* = 3.6 Hz, 1H), 6.95 (t, *J* = 7.2 Hz, 1H), 6.88 (d, *J* = 8.2 Hz, 1H), 6.83 (d, *J* = 3.2 Hz, 1H), 5.10 (s, 2H), 4.96 (t, *J* = 3.8 Hz, 1H), 2.81 (t, *J* = 7.6 Hz, 2H), 1.70 (q, *J* = 7.6 Hz, 2H), 1.46 (q, *J* = 7.6 Hz, 2H), 1.16-1.10 (m, 2H), 0.99-0.92 (m, 6H); ^{13}C NMR (100 MHz, $CDCl_3$) *δ* 163.4, 152.3, 137.2, 136.7, 131.7, 131.1, 128.5, 127.9, 127.5, 125.7, 123.5, 121.0, 115.0, 110.8, 70.0, 32.2, 25.1, 18.3, 17.8, 15.7, 13.9; ^{29}Si NMR (80 MHz, $CDCl_3$) *δ* -22.9. HRMS-EI exact mass calcd. for $C_{22}H_{26}OSSi^+$ ($[M]^+$) requires m/z 366.1468, found m/z 366.1471.

The enantiomeric excess was determined by HPLC on Chiralcel OD-H column (hexane : isopropanol = 99.9 : 0.1, flowing rate = 1.0 mL/min, 35 °C, UV detection at λ = 242 nm) t_{R1} = 13.7 min (major), t_{R2} = 16.0 min (minor).

(2-(benzyloxy)phenyl)(5-butylthiophen-2-yl)(propyl)silane (**3ad**)

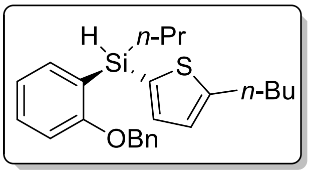

Colorless oil; isolated yield 64%, 88% ee; $[\alpha]_D^{22}$ = -6.4 (c = 1.0, DCM); ^{1}H NMR (400 MHz, CDCl$_3$) δ 7.45 (t, J = 6.0 Hz, 2H), 7.36-7.28 (m, 6H), 7.13 (d, J = 3.2 Hz, 1H), 6.95 (t, J = 7.2 Hz, 1H), 6.87 (d, J = 8.4 Hz, 1H), 6.83 (d, J = 3.2 Hz, 1H), 5.10 (s, 2H), 4.96 (t, J = 3.8 Hz, 1H), 2.83 (t, J = 7.6 Hz, 2H), 1.67-1.64 (m, 2H), 1.49-1.34 (m, 4H), 1.14-1.11 (m, 2H), 0.95-0.91 (m, 6H); ^{13}C NMR (100 MHz, CDCl$_3$) δ 163.4, 152.6, 137.2, 136.7, 131.7, 129.6, 128.5, 127.9, 127.5, 125.6, 123.5, 121.0, 115.0, 110.8, 70.0, 34.0, 29.8, 22.4, 18.3, 17.8, 15.7, 14.0; ^{29}Si NMR (80 MHz, CDCl$_3$) δ -22.9. HRMS-EI exact mass calcd. for $C_{24}H_{30}OSSi^+$ ($[M]^+$) requires m/z 394.1781, found m/z 394.1782.

The enantiomeric excess was determined by HPLC on Chiralcel OD-H column (hexane : isopropanol = 99.9 : 0.1, flowing rate = 1.0 mL/min, 35 °C, UV detection at λ = 262 nm) t_{R1} = 13.7 min (major), t_{R2} = 16.0 min (minor).

(2-(benzyloxy)phenyl)(5-chlorothiophen-2-yl)(propyl)silane (**3ae**)

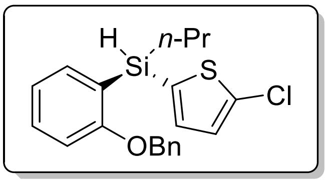

Colorless oil; isolated yield 50%, 81% ee; $[\alpha]_D^{22}$ = -0.8 (c = 1.0, DCM); ^{1}H NMR (400 MHz, CDCl$_3$) δ 7.45 (d, J = 7.2 Hz, 1H), 7.39-7.32 (m, 6H), 7.05 (d, J = 3.6 Hz, 1H), 6.97 (t, J = 7.2 Hz, 1H), 6.92 (d, J = 3.6 Hz, 1H), 6.89 (d, J = 8.2 Hz, 1H), 5.09 (s, 2H), 4.90 (t, J = 3.8 Hz, 1H), 1.44 (q, J = 7.6 Hz, 2H), 1.15-1.07 (m, 2H), 0.93 (t, J = 7.2 Hz, 3H); ^{13}C NMR (100 MHz, CDCl$_3$) δ 163.3, 137.1, 136.9, 136.0, 135.4, 134.1, 132.1, 128.6, 128.0, 127.6, 127.5, 122.4, 121.1, 110.8, 70.1, 18.2, 17.7, 15.4; ^{29}Si NMR (80 MHz, CDCl$_3$) δ -22.1. HRMS-EI exact mass calcd. for $C_{20}H_{21}OClSSi^+$ ($[M]^+$) requires m/z 372.0765, found m/z 372.0763.

The enantiomeric excess was determined by HPLC on Chiralcel OD-H column (hexane : isopropanol = 99.9 : 0.1, flowing rate = 0.8 mL/min, 35 °C, UV detection at λ = 235 nm) t_{R1} = 16.8 min (minor), t_{R2} = 19.7 min (major).

methyl 5-((2-(benzyloxy)phenyl)(propyl)silyl)thiophene-2-carboxylate (**3af**)

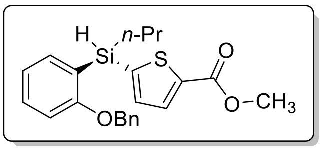

Colorless oil; isolated yield 62%, 56% ee; $[\alpha]_D^{22}$ = -1.8 (*c* = 1.0, DCM); ^{1}H NMR (400 MHz, $CDCl_3$) δ 7.79 (d, *J* = 3.6 Hz, 1H), 7.46 (d, *J* = 7.0 Hz, 1H), 7.39-7.31 (m, 6H), 7.24 (d, *J* = 3.6 Hz, 1H), 6.98 (t, *J* = 7.3 Hz, 1H), 6.90 (d, *J* = 8.3 Hz, 1H), 5.08 (s, 2H), 4.97 (t, *J* = 3.8 Hz, 1H), 3.88 (s, 3H), 1.46 (q, *J* = 7.6 Hz, 2H), 1.20-1.13 (m, 2H), 0.95 (t, *J* = 7.2 Hz, 3H); ^{13}C NMR (100 MHz, $CDCl_3$) δ 163.4, 162.7, 143.4, 138.9, 137.1, 136.8, 136.6, 134.2, 132.3, 128.6, 128.0, 127.6, 121.9, 121.2, 110.8, 70.1, 52.2, 18.2, 17.7, 15.3; ^{29}Si NMR (80 MHz, $CDCl_3$) δ -22.1. HRMS-EI exact mass calcd. for $C_{22}H_{24}O_3SSi^+$ ($[M]^+$) requires m/z 396.1210, found m/z 396.1211.

The enantiomeric excess was determined by HPLC on Chiralcel OD-H column (hexane : isopropanol = 99.7 : 0.3, flowing rate = 1.0 mL/min, 35 °C, UV detection at λ = 253 nm) t_{R1} = 22.4 min (major), t_{R2} = 29.6 min (minor).

(2-(benzyloxy)phenyl)(5-phenylthiophen-2-yl)(propyl)silane (**3ag**)

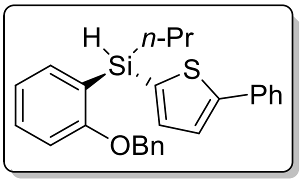

Colorless oil; isolated yield 60%, 54% ee; $[\alpha]_D^{22}$ = +12.7 (*c* = 1.0, DCM); ^{1}H NMR (400 MHz, $CDCl_3$) δ 7.64 (d, *J* = 7.6 Hz, 2H), 7.55 (d, *J* = 6.4 Hz, 1H), 7.42-7.35 (m, 9H), 7.31 (d, *J* = 3.8 Hz, 2H), 7.02 (t, *J* = 7.2 Hz, 1H), 6.94 (d, *J* = 8.4 Hz, 1H), 5.15 (s, 2H), 5.06 (t, *J* = 3.8 Hz, 1H), 1.57-1.51 (m, 2H), 1.26-1.19 (m, 2H), 1.01 (t, *J* = 7.2 Hz, 3H); ^{13}C NMR (100 MHz, $CDCl_3$) δ 163.4, 150.6, 137.7, 137.2, 137.1, 134.6, 133.8, 131.9, 129.0, 128.6, 128.0, 127.6, 127.6, 126.2, 124.5, 123.0, 121.1, 110.8, 70.1, 18.3, 17.8, 15.6; ^{29}Si NMR (80 MHz, $CDCl_3$) δ -22.4. HRMS-EI exact mass calcd. for $C_{26}H_{26}OSSi^+$ ($[M]^+$) requires m/z 414.1468, found m/z 414.1465.

The enantiomeric excess was determined by HPLC on Chiralcel OD-H column (hexane : isopropanol = 99.7 : 0.3, flowing rate = 1.0 mL/min, 35 °C, UV detection at λ = 288 nm) t_{R1} =12.5 min (minor), t_{R2} = 15.0 min (major).

(2-(benzyloxy)phenyl)(propyl)(thiophen-2-yl)silane (**3ah**)

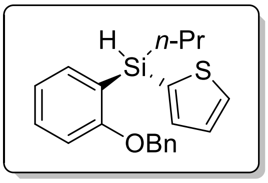

Colorless oil; isolated yield 65%, 90% ee; $[\alpha]_D^{22}$ = -0.6 (*c* = 1.0, DCM); ^{1}H NMR (400 MHz, $CDCl_3$) δ 7.62 (d, *J* = 4.6 Hz, 1H), 7.46 (d, *J* = 7.2 Hz, 1H), 7.36 (d, *J* = 6.0 Hz, 7H), 7.18 (t, *J* = 4.2 Hz, 1H), 6.97 (t, *J* = 7.2 Hz, 1H), 6.89 (d, *J* = 8.4 Hz, 1H),

5.11 (s, 2H), 5.03 (t, *J* = 4.0 Hz, 1H), 1.48 (q, *J* = 7.6 Hz, 2H), 1.21-1.14 (m, 2H), 0.95 (t, *J* = 7.2 Hz, 3H); ^{13}C NMR (100 MHz, $CDCl_3$) δ 163.4, 137.1, 137.1, 136.6, 133.7, 131.9, 131.5, 128.6, 128.2, 127.9, 127.5, 123.2, 121.0, 110.8, 70.0, 18.3, 17.8, 15.7; ^{29}Si NMR (80 MHz, $CDCl_3$) δ -22.5. HRMS-EI exact mass calcd. for $C_{20}H_{22}OSSi^+$ ($[M]^+$) requires m/z 338.1155, found m/z 338.1153.

The enantiomeric excess was determined by HPLC on Chiralcel OD-H column (hexane : isopropanol = 100 : 0, flowing rate = 1.0 mL/min, 35 °C, UV detection at λ = 236 nm) t_{R1} = 21.9 min (major), t_{R2} = 26.2 min (minor).

(2-(benzyloxy)phenyl)(4-methylthiophen-2-yl)(propyl)silane (**3ai**)

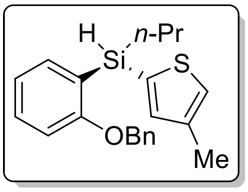

Colorless oil; isolated yield 53%, 90% ee; $[\alpha]_D^{22}$ = +52.0 (*c* = 1.0, DCM); ^{1}H NMR (400 MHz, $CDCl_3$) δ 7.45 (dd, J_1 = 7.2 Hz, J_2 = 1.6 Hz, 1H), 7.39-7.30 (m, 6H), 7.16 (s, 1H), 7.09 (s, 1H), 6.95 (t, *J* = 7.2 Hz, 1H), 6.88 (d, *J* = 8.0 Hz, 1H), 5.09 (s, 2H), 4.95 (t, *J* = 3.6 Hz, 1H), 2.25 (s, 3H), 1.48-1.43 (m, 2H), 1.17-1.11 (m, 2H), 0.93 (t, *J* = 7.6 Hz, 3H); ^{13}C NMR (100 MHz, $CDCl_3$) δ 163.4, 139.1, 137.2, 137.1, 133.8, 131.8, 128.6, 127.9, 127.6, 127.1, 123.3, 121.0, 115.0, 110.8, 70.0, 18.3, 17.8, 15.6, 15.2; ^{29}Si NMR (80 MHz, $CDCl_3$) δ -22.7. HRMS-EI exact mass calcd. for $C_{21}H_{24}OSSi^+$ ($[M]^+$) requires m/z 352.1212, found m/z 352.1315.

The enantiomeric excess was determined by HPLC on Chiralcel OD3-H column (hexane : isopropanol = 100 : 0, flowing rate = 1.0 mL/min, 22 °C, UV detection at λ = 231 nm) t_{R1} = 21.3 min (major), t_{R2} = 24.2 min (minor).

(2-(benzyloxy)phenyl)(4-methoxythiophen-2-yl)(propyl)silane (**3aj**)

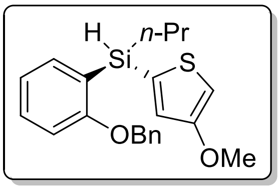

Colorless oil; isolated yield 65%, 86% ee; $[\alpha]_D^{22}$ = +8.3 (*c* = 1.0, DCM); ^{1}H NMR (400 MHz, $CDCl_3$) δ 7.47 (d, *J* = 6.4 Hz, 1H), 7.38-7.30 (m, 7H), 6.99-6.97 (m, 2H), 6.89 (d, *J* = 8.2 Hz, 1H), 6.54 (s, 1H), 5.11 (s, 2H), 4.94 (t, *J* = 3.6 Hz, 1H), 3.80 (s, 3H), 1.47 (q, *J* = 7.6 Hz, 3H), 1.18-1.11 (m, 2H), 0.95 (t, *J* = 7.2 Hz, 3H); ^{13}C NMR (100 MHz, $CDCl_3$) δ 163.4, 160.3, 137.1, 137.1, 133.6, 131.9, 128.6, 127.9, 127.5, 122.8, 121.1, 110.8, 103.4, 70.0, 57.8, 18.2, 17.8, 15.4; ^{29}Si NMR (80 MHz, $CDCl_3$) δ -22.1. HRMS-EI exact mass calcd. for $C_{21}H_{24}O_2SSi^+$ ($[M]^+$) requires m/z 368.1261, found m/z

368.1259.

The enantiomeric excess was determined by HPLC on Chiralcel OD-H column (hexane : isopropanol = 99.7 : 0.3, flowing rate = 1.0 mL/min, 35 °C, UV detection at λ = 219 nm) t_{R1} = 18.5 min (minor), t_{R2} = 19.9 min (major).

(2-(benzyloxy)phenyl)(4-chlorothiophen-2-yl)(propyl)silane (**3ak**)

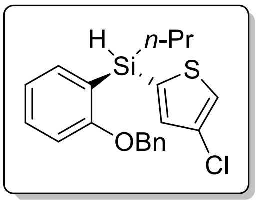

Colorless oil; isolated yield 68%, 80% ee; $[\alpha]_D^{22}$ = +7.2 (*c* = 1.0, DCM); ^{1}H NMR (400 MHz, $CDCl_3$) *δ* 7.48 (d, *J* = 7.2 Hz, 1H), 7.41-7.34 (m, 7H), 7.12 (s, 1H), 6.99 (t, *J* = 7.2 Hz, 1H), 6.91 (d, *J* = 8.4 Hz, 1H), 5.10 (s, 2H), 4.94 (t, *J* = 3.8 Hz, 1H), 1.47 (q, *J* = 7.6 Hz, 3H), 1.18-1.11 (m, 2H), 0.96 (t, *J* = 7.3 Hz, 3H); ^{13}C NMR (100 MHz, $CDCl_3$) *δ* 163.4, 137.1, 136.8, 136.2, 135.7, 132.2, 128.7, 128.1, 127.7, 126.5, 125.9, 122.2, 121.1, 110.8, 70.1, 18.2, 17.7, 15.3; ^{29}Si NMR (80 MHz, $CDCl_3$) *δ* -22.8. HRMS-EI exact mass calcd. for $C_{20}H_{21}OClSSi^+$ ($[M]^+$) requires m/z 372.0765, found m/z 372.0763.

The enantiomeric excess was determined by HPLC on Chiralcel OD-H column (hexane : isopropanol = 99.9 : 0.1, flowing rate = 0.8 mL/min, 35 °C, UV detection at λ = 213 nm) t_{R1} = 23.2 min (minor), t_{R2} = 27.6 min (major).

methyl 5-((2-(benzyloxy)phenyl)(propyl)silyl)thiophene-3-carboxylate (**3al**)

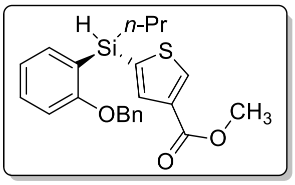

Colorless oil; isolated yield 70%, 89% ee; $[\alpha]_D^{22}$ = +0.2 (*c* = 1.0, DCM); ^{1}H NMR (400 MHz, $CDCl_3$) *δ* 8.31 (s, 1H), 7.74 (s, 1H), 7.47 (d, *J* = 7.0 Hz, 1H), 7.40-7.30 (m, 6H), 6.98 (t, *J* = 7.2 Hz, 1H), 6.90 (d, *J* = 8.2 Hz, 1H), 5.10 (s, 2H), 4.98 (t, *J* = 3.8 Hz, 1H), 3.86 (s, 3H), 1.47 (q, *J* = 7.6 Hz, 2H), 1.20-1.13 (m, 2H), 0.95 (t, *J* = 7.2 Hz, 3H); ^{13}C NMR (100 MHz, $CDCl_3$) *δ* 163.5, 163.4, 138.6, 137.1, 136.9, 135.9, 134.9, 132.2, 128.6, 128.0, 127.5, 122.3, 121.1, 110.8, 70.1, 51.8, 18.2, 17.7, 15.3; ^{29}Si NMR (80 MHz, $CDCl_3$) *δ* -22.3. HRMS-EI exact mass calcd. for $C_{22}H_{24}O_3SSi^+$ ($[M]^+$) requires m/z 396.1210, found m/z 396.1209.

The enantiomeric excess was determined by HPLC on Chiralcel OD-H column (hexane : isopropanol = 99.7 : 0.3, flowing rate = 1.0 mL/min, 35 °C, UV detection at λ = 213 nm) t_{R1} = 41.3 min (minor), t_{R2} = 44.3 min (major).

(2-(benzyloxy)phenyl)(4,5-dimethylthiophen-2-yl)(propyl)silane (**3am**)

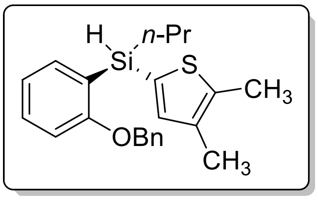

Colorless oil; isolated yield 65%, 90% ee; $[\alpha]_D^{22}$ = -6.8 (*c* = 1.0, DCM); ^{1}H NMR (400 MHz, $CDCl_3$) δ 7.46 (d, *J* = 6.6 Hz, 1H), 7.38-7.31 (m, 6H), 6.99 (s, 1H), 6.95 (t, *J* = 7.4 Hz, 1H), 6.88 (d, *J* = 8.3 Hz, 1H), 5.11 (s, 2H), 4.94 (t, *J* = 3.8 Hz, 1H), 2.35 (s, 3H), 2.12 (s, 3H), 1.46 (q, *J* = 7.6 Hz, 2H), 1.14-1.11 (m, 2H), 0.94 (t, *J* = 7.2 Hz, 3H); ^{13}C NMR (100 MHz, $CDCl_3$) δ 163.3, 140.0, 139.6, 137.2, 137.1, 134.8, 131.7, 128.6, 128.5, 127.9, 127.6, 123.6, 121.0, 110.7, 70.0, 18.3, 17.8, 15.7, 13.5, 13.4; ^{29}Si NMR (80 MHz, $CDCl_3$) δ -23.1. HRMS-EI exact mass calcd. for $C_{22}H_{26}OSSi^+$ ($[M]^+$) requires m/z 366.1468, found m/z 366.1465.

The enantiomeric excess was determined by HPLC on Chiralcel OD-H column (hexane : isopropanol = 99.9 : 0.1, flowing rate = 1.0 mL/min, 35 °C, UV detection at λ = 236 nm) t_{R1} = 14.6 min (major), t_{R2} = 16.0 min (minor).

benzo[b]thiophen-2-yl(2-(benzyloxy)phenyl)(propyl)silane (**3an**)

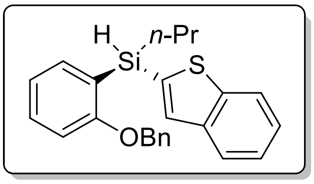

Colorless oil; isolated yield 57%, 86% ee; $[\alpha]_D^{22}$ = +292 (*c* = 1.0, DCM); ^{1}H NMR (400 MHz, $CDCl_3$) δ 7.8-7.85 (m, 1H), 7.78-7.75 (m, 1H), 7.51-7.49 (m, 2H), 7.40-7.30 (m, 7H), 7.26 (s, 1H), 6.97 (t, *J* = 7.2 Hz, 1H), 6.90 (d, *J* = 8.4 Hz, 1H), 5.10 (s, 2H), 5.05 (t, *J* = 3.6 Hz, 1H), 1.51-1.47 (m, 2H), 1.23-1.18 (m, 2H), 0.96 (t, *J* = 7.2 Hz, 3H); ^{13}C NMR (100 MHz, $CDCl_3$) δ 163.5, 144.2, 141.1, 137.2, 137.0, 136.3, 133.6, 132.1, 128.6, 128.0, 127.6, 124.3, 124.0, 123.6, 122.5, 122.2, 121.1, 110.8, 70.1, 18.3, 17.8, 15.3; ^{29}Si NMR (80 MHz, $CDCl_3$) δ -21.6. HRMS-EI exact mass calcd. for $C_{124}H_{24}OSSi^+$ ($[M]^+$) requires m/z 388.1312, found m/z 388.1313.

The enantiomeric excess was determined by HPLC on Chiralcel OD-H column (hexane : isopropanol = 100 : 0, flowing rate = 1.0 mL/min, 35 °C, UV detection at λ = 239 nm) t_{R1} = 34.7 min (minor), t_{R2} = 37.8 min (major).

(2-methoxyphenyl)(5-methylthiophen-2-yl)(propyl)silane (**3ba**)

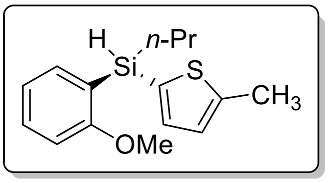

Colorless oil; isolated yield 54%, 88% ee; $[\alpha]_D^{22}$ = +18.0 (*c* = 1.0, DCM); ^{1}H NMR (400 MHz, $CDCl_3$) δ 7.43-7.35 (m, 2H), 7.17 (d, *J* = 3.2 Hz, 1H), 6.93 (t, *J* = 7.2 Hz, 1H), 6.85-6.82 (m, 2H), 4.89 (t, *J* = 3.6 Hz, 1H), 3.84 (s, 3H), 2.52 (s, 3H), 1.50-1.43 (m, 2H), 1.16-1.10 (m, 2H), 0.97 (t, *J* = 7.2 Hz, 3H); ^{13}C NMR (100 MHz, $CDCl_3$) δ 164.2, 146.4, 136.9, 136.9, 131.8, 126.9, 123.2, 120.8, 109.6, 55.3, 18.3, 17.9, 15.9, 15.3; ^{29}Si NMR (80 MHz, $CDCl_3$) δ -22.5. HRMS-EI exact mass calcd. for $C_{15}H_{20}OSSi^+$ ($[M]^+$) requires m/z 276.0999, found m/z 276.0998.

The enantiomeric excess was determined by HPLC on Chiralcel OD-H column (hexane : isopropanol = 100 : 0, flowing rate = 1.0 mL/min, 35 °C, UV detection at λ = 241 nm) t_{R1} = 8.7 min (minor), t_{R2} = 9.5 min (major).

(2-methoxyphenyl)(4-methylthiophen-2-yl)(propyl)silane (3bi)

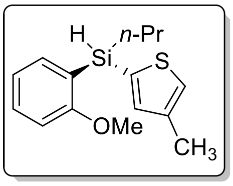

Colorless oil; isolated yield 50%, 90% ee; $[\alpha]_D^{22}$ = -10.0 (*c* = 1.0, DCM); ^{1}H NMR (400 MHz, $CDCl_3$) δ 7.43-7.36 (m, 2H), 7.17 (d, *J* = 7.2 Hz, 1H), 6.94 (t, *J* = 7.2 Hz, 1H), 6.85 (d, *J* = 8.4 Hz, 1H), 4.91 (t, *J* = 3.7 Hz, 1H), 3.84 (s, 3H), 2.28 (s, 3H), 1.51-1.43 (m, 2H), 1.17-1.11 (m, 2H), 0.98 (t, *J* = 7.2 Hz, 3H); ^{13}C NMR (100 MHz, $CDCl_3$) δ 164.3, 139.0, 139.0, 136.9, 134.0, 131.8, 127.2, 123.0, 120.8, 109.7, 55.3, 18.3, 17.9, 15.8, 15.2; ^{29}Si NMR (80 MHz, $CDCl_3$) δ -22.3. HRMS-EI exact mass calcd. for $C_{15}H_{20}OSSi^+$ ($[M]^+$) requires m/z 276.0999, found m/z 276.0998.

The enantiomeric excess was determined by HPLC on Chiralcel OD-H and AD-H column (hexane : isopropanol = 100 : 0, flowing rate = 0.5 mL/min, 35 °C, UV detection at λ = 233 nm) t_{R1} = 27.3 min (minor), t_{R2} = 28.2 min (major).

benzo[b]thiophen-2-yl(2-methoxyphenyl)(propyl)silane (3bn)

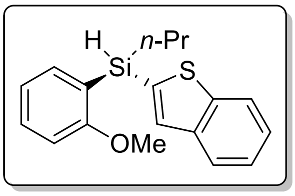

Colorless oil; isolated yield 51%, 88% ee; $[\alpha]_D^{22}$ = -52.0 (*c* = 1.0, DCM); ^{1}H NMR (400 MHz, $CDCl_3$) δ 7.87-7.87 (m, 1H), 7.83-7.80 (m, 1H), 7.60 (s, 1H), 7.48 (dd, J_1 = 7.2 Hz, J_2 = 2.0 Hz, 1H), 7.41 (td, J_1 = 8.0 Hz, J_2 = 1.6 Hz, 1H), 7.38-7.31 (m, 2H), 6.96-6.94 (m, 1H), 6.87 (d, *J* = 8.4 Hz, 1H), 5.02 (t, *J* = 3.6 Hz, 1H), 3.86 (s, 3H), 1.55-1.50 (m, 2H), 1.26 -1.21 (m, 2H), 1.01 (t, *J* = 7.2 Hz, 3H); ^{13}C NMR (100 MHz,

$CDCl_3$) δ 164.3, 144.3, 141.0, 137.0, 136.5, 133.6, 132.1, 124.4, 124.0, 123.6, 122.3, 122.2, 120.9, 109.7, 55.3, 18.3, 17.9, 15.5; ^{29}Si NMR (80 MHz, $CDCl_3$) δ -20.9. HRMS-EI exact mass calcd. for $C_{18}H_{20}OSSi^+$ ($[M]^+$) requires m/z 312.0999, found m/z 312.0997.

The enantiomeric excess was determined by HPLC on Chiralcel OD-H column (hexane : isopropanol = 100 : 0, flowing rate = 1.0 mL/min, 35 °C, UV detection at λ = 239 nm) t_{R1} = 13.8 min (minor), t_{R2} = 14.8 min (major).

(2-ethoxyphenyl)(5-methylthiophen-2-yl)(propyl)silane (**3ca**)

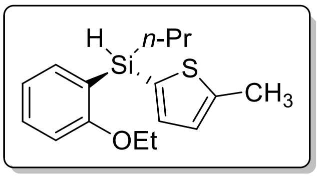

Colorless oil; isolated yield 68%, 92% ee; $[\alpha]_D^{22}$ = +50.0 (*c* = 1.0, DCM); ^{1}H NMR (400 MHz, $CDCl_3$) δ 7.42 (dd, J_1 = 7.2 Hz, J_2 = 1.6 Hz, 1H), 7.37-7.32 (m, 1H), 7.18 (d, *J* = 3.2 Hz, 1H), 6.91 (t, *J* = 7.2 Hz, 1H), 6.83-6.80 (m, 2H), 4.90 (t, *J* = 3.6 Hz, 1H), 4.06 (q, *J* = 7.2 Hz, 2H), 2.52 (s, 3H), 1.52-1.41 (m, 5H), 1.19-1.13 (m, 2H), 0.98 (t, *J* = 7.2 Hz, 3H); ^{13}C NMR (100 MHz, $CDCl_3$) δ 163.6, 146.3, 137.0, 136.9, 131.9, 131.7, 126.9, 123.2, 120.6, 110.3, 63.5, 18.4, 17.9, 16.0, 15.3, 14.9; ^{29}Si NMR (80 MHz, $CDCl_3$) δ -22.3. HRMS-EI exact mass calcd. for $C_{16}H_{22}OSSi^+$ ($[M]^+$) requires m/z 290.1155, found m/z 290.1155.

The enantiomeric excess was determined by HPLC on Chiralcel OD-H column (hexane : isopropanol = 100 : 0, flowing rate = 0.5 mL/min, 35 °C, UV detection at λ = 239 nm) t_{R1} = 17.9 min (minor), t_{R2} = 19.0 min (major).

(2-ethoxyphenyl)(4-methylthiophen-2-yl)(propyl)silane (**3ci**)

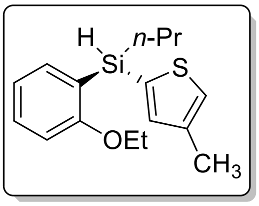

Colorless oil; isolated yield 63%, 90% ee; $[\alpha]_D^{22}$ = -52.0 (*c* = 1.0, DCM); ^{1}H NMR (400 MHz, $CDCl_3$) δ 7.43 (dd, J_1 = 7.2 Hz, J_2 = 1.6 Hz, 1H), 7.37-7.33 (m, 1H), 7.17 (s, 1H), 6.92 (t, *J* = 7.2 Hz, 1H), 6.82 (d, *J* = 8.0 Hz, 1H), 4.91 (t, *J* = 3.6 Hz, 1H), 4.06 (q, *J* = 7.2 Hz, 2H), 2.29 (s, 3H), 1.49 (q, *J* = 7.6 Hz, 2H), 1.42 (t, *J* = 7.2 Hz, 3H), 1.21-1.16 (m, 2H), 0.98 (t, *J* = 7.2 Hz, 3H); ^{13}C NMR (100 MHz, $CDCl_3$) δ 163.6, 139.0, 139.0, 137.0, 134.1, 131.8, 127.1, 123.0, 120.6, 110.3, 63.5, 18.4, 17.9, 15.9, 15.2, 14.9; ^{29}Si NMR (80 MHz, $CDCl_3$) δ -22.0. HRMS-EI exact mass calcd. for $C_{16}H_{22}OSSi^+$ ($[M]^+$) requires m/z 290.1155, found m/z 290.1155.

The enantiomeric excess was determined by HPLC on Chiralcel OD-H column (hexane : isopropanol = 100 : 0, flowing rate = 0.5 mL/min, 35 °C, UV detection at λ = 239 nm) t_{R1} = 18.5 min (minor), t_{R2} = 19.4 min (major).

benzo[b]thiophen-2-yl(2-ethoxyphenyl)(propyl)silane (**3cn**)

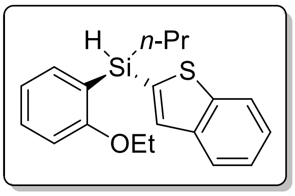

Colorless oil; isolated yield 67%, 89% ee; $[\alpha]_D^{22}$ = +238 (*c* = 1.0, DCM); ^{1}H NMR (400 MHz, $CDCl_3$) δ 7.89-7.87 (m, 1H), 7.81 (dd, J_1 = 6.4 Hz, J_2 = 2.4 Hz, 1H), 7.61 (s, 1H), 7.48 (dd, J_1 = 7.2 Hz, J_2 = 1.6 Hz, 1H), 7.41-7.35 (m, 1H), 7.34-7.30 (m, 2H), 6.94 (t, *J* = 7.2 Hz, 1H), 6.84 (d, *J* = 8.0 Hz, 1H), 5.02 (t, *J* = 3.6 Hz, 1H), 4.07 (q, *J* = 7.2 Hz, 2H), 1.58- 1.52 (m, 2H), 1.42 (t, *J* = 7.2 Hz, 3H), 1.29-1.24 (m, 2H), 1.01 (t, *J* = 7.2 Hz, 3H); ^{13}C NMR (100 MHz, $CDCl_3$) δ 163.6, 139.0, 139.0, 137.0, 134.1, 131.8, 127.1, 123.0, 120.6, 110.3, 63.5, 18.4, 17.9, 15.9, 15.2, 14.9; ^{29}Si NMR (80 MHz, $CDCl_3$) δ -20.9. HRMS-EI exact mass calcd. for $C_{19}H_{22}OSSi^+$ ($[M]^+$) requires m/z 326.1155, found m/z 326.1155.

The enantiomeric excess was determined by HPLC on Chiralcel OJ-H column (hexane : isopropanol = 100 : 0, flowing rate = 1.0 mL/min, 35 °C, UV detection at λ = 215 nm) t_{R1} = 12.3 min (major), t_{R2} = 16.3 min (minor).

(2-butoxyphenyl)(5-methylthiophen-2-yl)(propyl)silane (**3da**)

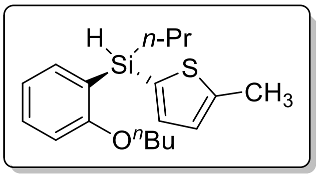

Colorless oil; isolated yield 63%, 90% ee; $[\alpha]_D^{22}$ = +132 (*c* = 1.0, DCM); ^{1}H NMR (400 MHz, $CDCl_3$) δ 7.41 (dd, J_1 = 7.2 Hz, J_2 = 1.6 Hz, 1H), 7.35 (td, J_1 = 8.0 Hz, J_2 = 1.6 Hz, 1H), 7.16 (d, *J* = 3.2 Hz, 1H), 6.91 (t, *J* = 7.2 Hz, 1H), 6.84-6.81 (m, 2H), 4.91 (t, *J* = 3.6 Hz, 1H), 3.98 (t, *J* = 6.4 Hz, 2H), 2.52 (s, 2H), 1.81-1.74 (m, 2H), 1.52-1.44 (m, 4H), 1.19-1.12 (m, 2H), 1.00-0.95 (m, 6H); ^{13}C NMR (100 MHz, $CDCl_3$) δ 163.8, 146.3, 137.0, 136.8, 131.9, 131.7, 126.9, 123.1, 120.5, 110.2, 67.6, 31.5, 19.5, 18.4, 17.9, 15.9, 15.3, 14.0; ^{29}Si NMR (80 MHz, $CDCl_3$) δ -22.9. HRMS-EI exact mass calcd. for $C_{18}H_{26}OSSi^+$ ($[M]^+$) requires m/z 318.1468, found m/z 318.1471.

The enantiomeric excess was determined by HPLC on Chiralcel OD-H column (hexane : isopropanol = 99.9 : 0.1, flowing rate = 1.0 mL/min, 35 °C, UV detection at λ = 285 nm) t_{R1} = 6.8 min (minor), t_{R2} = 7.5 min (major).

(2-butoxyphenyl)(4-methylthiophen-2-yl)(propyl)silane (**3di**)

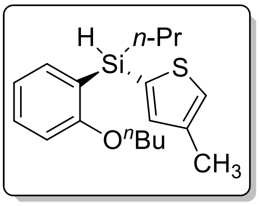

Colorless oil; isolated yield 65%, 89% ee; $[\alpha]_D^{22}$ = +166 (c = 1.0, DCM); ^{1}H NMR (400 MHz, $CDCl_3$) δ 7.42 (dd, J_1 = 7.2 Hz, J_2 = 1.6 Hz, 1H), 7.38-7.33 (m, 1H), 7.17 (d, J = 6.8 Hz, 1H), 6.92 (t, J = 7.2 Hz, 1H), 6.82 (d, J = 8.4 Hz, 1H), 4.93 (t, J = 3.6 Hz, 1H), 3.98 (t, J = 6.4 Hz, 2H), 1.81-1.74 (m, 2H), 1.51-1.44 (m, 4H), 1.20-1.16 (m, 2H), 1.01-0.95 (m, 6H); ^{13}C NMR (100 MHz, $CDCl_3$) δ 163.8, 139.0, 138.9, 137.1, 134.1, 131.8, 127.1, 122.9, 120.5, 110.2, 67.6, 31.5, 19.4, 18.4, 17.9, 15.8, 15.2, 14.0; ^{29}Si NMR (80 MHz, $CDCl_3$) δ -22.6. HRMS-EI exact mass calcd. for $C_{18}H_{26}OSSi^+$ ($[M]^+$) requires m/z 318.1468, found m/z 318.1470.

The enantiomeric excess was determined by HPLC on Chiralcel OD-H column (hexane : isopropanol = 99.9 : 0.1, flowing rate = 1.0 mL/min, 35 °C, UV detection at λ = 270 nm) t_{R1} = 6.9 min (minor), t_{R2} = 7.6 min (major).

benzo[b]thiophen-2-yl(2-butoxyphenyl)(propyl)silane (**3dn**)

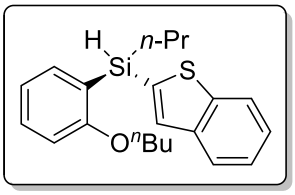

Colorless oil; isolated yield 61%, 86% ee; $[\alpha]_D^{22}$ = +226 (c = 1.0, DCM); ^{1}H NMR (400 MHz, $CDCl_3$) δ 7.89-7.87 (m, 1H), 7.82-7.80 (m, 1H), 7.60 (s, 1H), 7.47 (dd, J_1 = 7.2 Hz, J_2 = 1.6 Hz, 1H), 7.41-7.31 (m, 3H), 6.94 (m, t, J = 7.2 Hz, 1H), 6.85 (d, J = 8.0 Hz, 1H), 5.03 (t, J = 3.6 Hz, 1H), 3.99 (t, J = 6.0 Hz, 2H), 1.80-1.73 (m, 2H), 1.58-1.52 (m, 2H), 1.48-1.42 (m, 2H), 1.29-1.23 (m, 2H), 1.02 (t, J = 7.2 Hz, 3H), 0.93 (t, J = 7.2 Hz, 3H); ^{13}C NMR (100 MHz, $CDCl_3$) δ 163.9, 144.2, 141.1, 137.1, 136.6, 133.4, 132.0, 124.3, 124.0, 123.6, 122.2, 122.1, 120.6, 110.2, 67.6, 31.4, 19.4, 18.4, 17.9, 15.5, 14.0; ^{29}Si NMR (80 MHz, $CDCl_3$) δ -21.5. HRMS-EI exact mass calcd. for $C_{21}H_{26}OSSi^+$ ($[M]^+$) requires m/z 354.1468, found m/z 354.1470.

The enantiomeric excess was determined by HPLC on Chiralcel AD-H column (hexane : isopropanol = 99.9 : 0.1, flowing rate = 1.0 mL/min, 35 °C, UV detection at λ = 300 nm) t_{R1} = 5.6 min (minor), t_{R2} = 6.2 min (major).

(2-isopropoxyphenyl)(5-methylthiophen-2-yl)(propyl)silane (**3ea**)

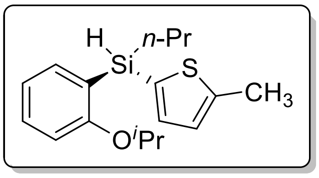

Colorless oil; isolated yield 69%, 90% ee; $[\alpha]_D^{22}$ = +142 (*c* = 1.0, DCM); ^{1}H NMR (400 MHz, $CDCl_3$) *δ* 7.42 (dd, J_1 = 7.2 Hz, J_2 = 1.6 Hz, 1H), 7.36-7.31 (m, 1H), 7.18 (d, *J* = 3.6 Hz, 1H), 6.89 (td, J_1 = 7.2 Hz, J_2 = 0.8 Hz, 1H), 6.84-6.80 (m, 2H), 4.89 (t, *J* = 4.0 Hz, 1H), 4.65-4.59 (m, 1H), 2.52 (s, 3H), 1.54-1.44 (m, 2H), 1.34 (dd, J_1 = 6.0 Hz, J_2 = 4.0 Hz, 6H), 1.20-1.15 (m, 2H), 0.98 (t, *J* = 7.2 Hz, 3H); ^{13}C NMR (100 MHz, $CDCl_3$) *δ* 162.4, 146.2, 137.3, 136.8, 132.1, 131.6, 126.8, 123.8, 120.2, 111.0, 69.3, 22.1, 22.1, 18.4, 17.9, 16.0, 15.3; ^{29}Si NMR (80 MHz, $CDCl_3$) *δ* -22.5. HRMS-EI exact mass calcd. for $C_{17}H_{24}OSSi^+$ ($[M]^+$) requires m/z 304.1312, found m/z 304.1314.

The enantiomeric excess was determined by HPLC on Chiralcel OD-H column (hexane : isopropanol = 100 : 0, flowing rate = 0.6 mL/min, 35 °C, UV detection at λ = 208 nm) t_{R1} = 10.8 min (minor), t_{R2} = 11.6 min (major).

(2-isopropoxyphenyl)(4-methylthiophen-2-yl)(propyl)silane (**3ei**)

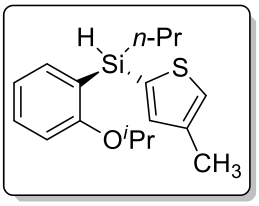

Colorless oil; isolated yield 58%, 94% ee; $[\alpha]_D^{22}$ = +80.0 (*c* = 1.0, DCM); ^{1}H NMR (400 MHz, $CDCl_3$) *δ* 7.43 (dd, J_1 = 7.2 Hz, J_2 =2.0 Hz, 1H), 7.36-7.32 (m, 1H), 7.17 (s, 2H), 6.89 (t, *J* = 7.2 Hz, 1H), 6.82 (d, *J* = 8.4 Hz, 1H), 4.90 (t, *J* = 4.0 Hz, 1H), 4.65-4.59 (m, 1H), 2.29 (s, 3H), 1.52-1.47 (m, 2H), 1.33 (t, *J* = 6.0 Hz, 6H), 1.22-1.16 (m, 2H), 0.99 (t, *J* = 7.2 Hz, 3H); ^{13}C NMR (100 MHz, $CDCl_3$) *δ* 162.4, 139.0, 138.9, 137.3, 134.3, 131.6, 127.0, 123.6, 120.2, 111.0, 69.3, 31.7, 22.8, 22.1, 22.0, 18.4, 17.9, 15.9, 15.2, 14.3; ^{29}Si NMR (80 MHz, $CDCl_3$) *δ* -22.0. HRMS-EI exact mass calcd. for $C_{17}H_{24}OSSi^+$ ($[M]^+$) requires m/z 304.1312, found m/z 304.1310.

The enantiomeric excess was determined by HPLC on Chiralcel OD-H column (hexane : isopropanol = 100 : 0, flowing rate = 0.6 mL/min, 35 °C, UV detection at λ = 226 nm) t_{R1} = 11.1 min (minor), t_{R2} = 11.7 min (major).

benzo[b]thiophen-2-yl(2-isopropoxyphenyl)(propyl)silane (**3en**)

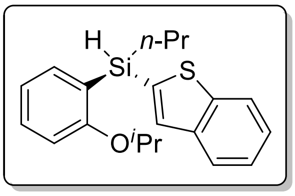

Colorless oil; isolated yield 65%, 90% ee; $[\alpha]_D^{22}$ = +314 (*c* = 1.0, DCM); ^{1}H NMR (400 MHz, $CDCl_3$) δ 7.91-7.89 (m, 1H), 7.84-7.81 (m, 1H), 7.62 (s, 1H), 7.49 (dd, J_1 = 7.2 Hz, J_2 =1.6 Hz, 1H), 7.40-7.32 (m, 3H), 6.92 (t, *J* = 7.6 Hz, 1H), 6.85 (d, *J* = 8.4 Hz, 1H), 5.03 (t, *J* = 3.6 Hz, 1H), 4.67-4.61 (m, 1H), 1.59-1.54 (m, 2H), 1.36-1.34 (m, 6H), 1.30-1.25 (m, 2H), 1.04 (t, *J* = 7.2 Hz, 3H); ^{13}C NMR (100 MHz, $CDCl_3$) δ 162.5, 144.2, 141.1, 137.4, 136.8, 133.5, 131.9, 124.3, 124.0, 123.6, 122.8, 122.2, 120.3, 111.1, 69.5, 22.1, 22.0, 18.4, 17.9, 15.7; ^{29}Si NMR (80 MHz, $CDCl_3$) δ -22.9. HRMS-EI exact mass calcd. for $C_{20}H_{24}OSSi^+$ ($[M]^+$) requires m/z 340.1312, found m/z 340.1312.

The enantiomeric excess was determined by HPLC on Chiralcel OJ-H column (hexane : isopropanol = 100 : 0, flowing rate = 1.0 mL/min, 35 °C, UV detection at λ = 244 nm) t_{R1} = 8.8 min (major), t_{R2} = 13.0 min (minor).

(2-ethylphenyl)(5-methylthiophen-2-yl)(propyl)silane (**3fa**)

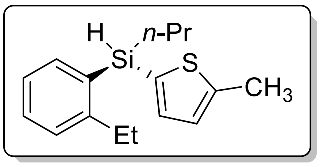

Colorless oil; isolated yield 76%, 95% ee; $[\alpha]_D^{22}$ = -4 (*c* = 1.0, DCM); ^{1}H NMR (400 MHz, $CDCl_3$) δ 7.55 (d, *J* = 7.3 Hz, 1H), 7.39 (t, *J* = 7.5 Hz, 1H), 7.30-7.24 (m, 1H), 7.21 (t, *J* = 7.3 Hz, 1H), 7.11 (d, *J* = 3.1 Hz, 1H), 6.86 (s, 1H), 5.09 (t, *J* = 3.8 Hz, 1H), 2.80 (q, *J* = 7.5 Hz, 2H), 2.55 (s, 3H), 1.56 (q, *J* = 7.5 Hz, 2H), 1.23-1.16 (m, 5H), 1.04 (t, *J* = 7.0 Hz, 3H); ^{13}C NMR (100 MHz, $CDCl_3$) δ 150.6, 146.7, 137.0, 136.0, 132.8, 131.9, 130.3, 128.1, 127.1, 125.3, 29.4, 18.4, 17.9, 16.3, 16.3, 15.3, 1.2; ^{29}Si NMR (80 MHz, $CDCl_3$) δ -25.7. HRMS-APCI exact mass calcd. for $C_{16}H_{22}SSi^+$ ($[M]^+$) requires m/z 274.1206, found m/z 274.1212.

The enantiomeric excess was determined by HPLC on Chiralcel OJ-H column (hexane : isopropanol = 100 : 0, flowing rate = 0.2 mL/min, 22 °C, UV detection at λ = 227 nm) t_{R1} =26.9 min (major), t_{R2} = 29.0 min (minor).

(2-ethylphenyl)(4-methylthiophen-2-yl)(propyl)silane (**3fi**)

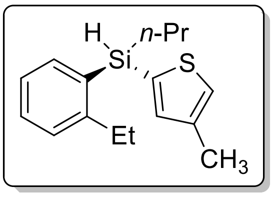

Colorless oil; isolated yield 78%, 91% ee; $[\alpha]_D^{22}$ = -4 (*c* = 1.0, DCM); ^{1}H NMR (400 MHz, $CDCl_3$) δ 7.55 (d, *J* = 7.4 Hz, 1H), 7.39 (t, *J* = 7.6 Hz, 1H), 7.30-7.25 (m, 1H), 7.22 (d, *J* = 7.8 Hz, 2H), 7.09 (s, 1H), 5.10 (t, *J* = 3.8 Hz, 1H), 2.79 (q, *J* = 7.5 Hz, 2H), 2.30 (s, 3H), 1.58-1.52 (m, 2H), 1.21-1.19 (m, 5H), 1.04 (t, *J* = 7.3 Hz, 3H); ^{13}C NMR (100 MHz, $CDCl_3$) δ 150.7, 139.3, 139.0, 136.0, 134.1, 132.7, 130.3, 128.1, 127.4, 125.3, 29.5, 18.4, 17.9, 16.3, 16.2, 15.2; ^{29}Si NMR (80 MHz, $CDCl_3$) δ -25.4. HRMS-APCI exact mass calcd. for $C_{16}H_{21}SSi^+$ ($[M-H]^+$) requires m/z 273.1128, found m/z 273.1128.

The enantiomeric excess was determined by HPLC on Chiralcel OJ-H column (hexane : isopropanol = 100 : 0, flowing rate = 0.4 mL/min, 35 °C, UV detection at λ = 254 nm) t_{R1} = 11.0 min (major), t_{R2} = 12.2 min (minor).

benzo[b]thiophen-2-yl(2-ethylphenyl)(propyl)silane (**3fn**)

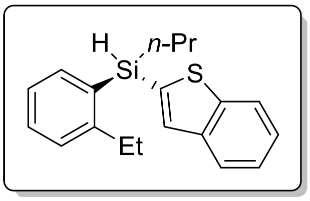

Colorless oil; isolated yield 80%, 92% ee; $[\alpha]_D^{22}$ = +6.4 (*c* = 1.0, DCM); ^{1}H NMR (400 MHz, $CDCl_3$) δ 7.91 (d, *J* = 7.2 Hz, 1H), 7.83 (d, *J* = 8.3 Hz, 1H), 7.62 (d, *J* = 7.4 Hz, 1H), 7.55 (s, 1H), 7.42 (d, *J* = 7.5 Hz, 1H), 7.37-7.35 (m, 2H), 7.31- 7.24 (m, 2H), 5.22 (t, *J* = 3.8 Hz, 1H), 2.83 (q, *J* = 7.6 Hz, 2H), 1.62 (q, *J* = 7.7 Hz, 2H), 1.31-1.28 (m, 2H), 1.23 (t, *J* = 7.5 Hz, 3H), 1.08 (t, *J* = 7.2 Hz, 3H); ^{13}C NMR (100 MHz, $CDCl_3$) δ 150.8, 144.2, 141.1, 136.5, 136.1, 133.6, 131.9, 130.6, 128.2, 125.4, 124.5, 124.2, 123.7, 122.3, 29.5, 18.4, 17.9, 16.3, 15.9; ^{29}Si NMR (80 MHz, $CDCl_3$) δ -24.4. HRMS-APCI exact mass calcd. for $C_{19}H_{21}SSi^+$ ($[M-H]^+$) requires m/z 309.1128, found m/z 309.1142.

The enantiomeric excess was determined by HPLC on Chiralcel OD-H column (hexane : isopropanol = 100 : 0, flowing rate = 0.5 mL/min, 35 °C, UV detection at λ = 268 nm) t_{R1} = 16.0 min (major), t_{R2} = 17.5 min (minor).

(2-isopropylphenyl)(5-methylthiophen-2-yl)(propyl)silane (**3ga**)

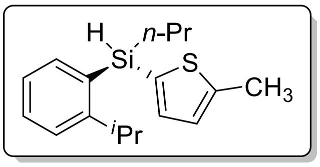

Colorless oil; isolated yield 82%, 90% ee; $[\alpha]_D^{22}$ = -4 (*c* = 1.0, DCM); ^{1}H NMR (400 MHz, $CDCl_3$) δ 7.52 (d, *J* = 7.5 Hz, 1H), 7.41 (t, *J* = 7.5 Hz, 1H), 7.33 (d, *J* = 7.7 Hz, 1H), 7.19 (t, *J* = 7.3 Hz, 1H), 7.10 (s, 1H), 6.84 (s, 1H), 5.09 (d, *J* = 4.1 Hz, 1H), 3.21 (p, *J* = 6.7 Hz, 1H), 2.53 (s, 3H), 1.55 (q, *J* = 7.7 Hz, 2H), 1.22-1.16 (m, 8H), 1.03 (t, *J* = 7.2 Hz, 3H); ^{13}C NMR (100 MHz, $CDCl_3$) δ 155.5, 146.7, 136.9, 136.1, 132.4, 132.0, 130.5, 127.1, 125.5, 125.1, 34.1, 24.5, 24.5, 18.4, 18.0, 16.5, 15.3; ^{29}Si NMR (80 MHz, $CDCl_3$) δ -24.8. HRMS-APCI exact mass calcd. for $C_{17}H_{25}SSi^+$ ($[M+H]^+$) requires m/z 289.1441, found m/z 289.1444.

The enantiomeric excess was determined by HPLC on Chiralcel OD-H+OJ-H column (hexane : isopropanol = 100 : 0, flowing rate = 0.3 mL/min, 35 °C, UV detection at λ = 258 nm) t_{R1} = 28.0 min (major), t_{R2} = 29.5 min (minor).

(2-isopropylphenyl)(4-methylthiophen-2-yl)(propyl)silane (**3gi**)

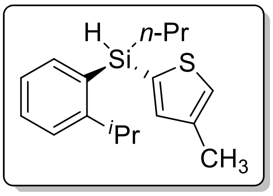

Colorless oil; isolated yield 81%, 90% ee; $[\alpha]_D^{22}$ = +11.2 (*c* = 1.0, DCM); ^{1}H NMR (400 MHz, $CDCl_3$) δ 7.52 (d, *J* = 7.4 Hz, 1H), 7.41 (t, *J* = 7.4 Hz, 1H), 7.33 (d, *J* = 8.0 Hz, 1H), 7.21-7.17 (m, 2H), 7.08 (s, 1H), 5.08 (s, 1H), 3.18 (q, *J* = 6.8 Hz, 1H), 2.28 (s, 3H), 1.57-1.52 (m, 2H), 1.23-1.15 (m, 8H), 1.03 (t, *J* = 7.2 Hz, 3H); ^{13}C NMR (100 MHz, $CDCl_3$) δ 155.5, 139.2, 139.0, 136.1, 134.2, 132.2, 130.5, 127.3, 125.5, 125.1, 34.2, 24.5, 24.4, 18.4, 18.0, 16.5, 15.2; ^{29}Si NMR (80 MHz, $CDCl_3$) δ -24.4. HRMS-APCI exact mass calcd. for $C_{17}H_{25}SSi^+$ ($[M+H]^+$) requires m/z 289.1441, found m/z 289.1441.

The enantiomeric excess was determined by HPLC on Chiralcel OD-H+OJ-H column (hexane : isopropanol = 100 : 0, flowing rate = 0.3 mL/min, 35 °C, UV detection at λ = 205 nm) t_{R1} = 28.3 min (major), t_{R2} = 31.2 min (minor).

benzo[b]thiophen-2-yl(2-isopropylphenyl)(propyl)silane (**3gn**)

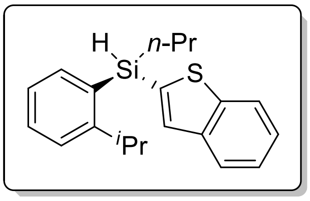

Colorless oil; isolated yield 84%, 93% ee; $[\alpha]_D^{22}$ = +14.6 (*c* = 1.0, DCM); ^{1}H NMR (400 MHz, $CDCl_3$) δ 7.90 (d, *J* = 7.3 Hz, 1H), 7.82 (d, *J* = 8.2 Hz, 1H), 7.59 (d, *J* = 7.5 Hz, 1H), 7.54 (s, 1H), 7.45 (t, *J* = 7.5 Hz, 1H), 7.38-7.34 (m, 3H), 7.23 (t, *J* = 7.4 Hz, 1H), 5.22 (d, *J* = 4.1 Hz, 1H), 3.24 (p, *J* = 6.8 Hz, 1H), 1.64-1.58 (m, 2H), 1.31-1.21 (m, 8H), 1.07 (t, *J* = 7.7 Hz, 3H); ^{13}C NMR (100 MHz, $CDCl_3$) δ 155.6, 144.2, 141.1, 136.7, 136.1, 133.6, 131.5, 130.8, 125.6, 125.3, 124.5, 124.2, 123.7, 122.3, 34.4, 24.5, 18.4, 17.9, 16.2; ^{29}Si NMR (80 MHz, $CDCl_3$) δ -23.5. HRMS-APCI exact mass calcd. for $C_{20}H_{23}SSi^+$ ($[M-H]^+$) requires m/z 323.1284, found m/z 323.1297.

The enantiomeric excess was determined by HPLC on Chiralcel OD-H column (hexane : isopropanol = 100 : 0, flowing rate = 0.4 mL/min, 35 °C, UV detection at λ = 263 nm) t_{R1} = 35.3 min (major), t_{R2} = 40.5 min (minor).

(3',5'-dimethyl-[1,1'-biphenyl]-2-yl)(5-methylthiophen-2-yl)(propyl)silane (**3ha**)

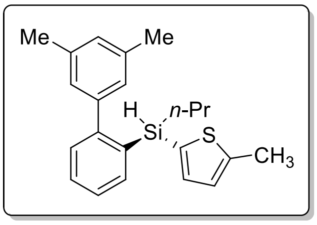

Colorless oil; isolated yield 65%, 74% ee; $[\alpha]_D^{22}$ = -102 (*c* = 1.0, DCM); ^{1}H NMR (400 MHz, $CDCl_3$) δ 7.65 (dd, J_1 = 7.2 Hz, J_2 = 1.6 Hz, 1H), 7.43 (td, J_1 = 7.6 Hz, J_2 = 1.6 Hz, 1H), 7.45-7.29 (m, 2H), 7.01-6.99 (m, 2H), 6.89 (s, 2H), 6.83-6.82 (m, 1H), 4.75 (t, *J* = 4.0 Hz, 1H), 2.53 (s, 3H), 2.31 (s, 6H), 1.33-1.26 (m, 1H), 0.84 (t, *J* = 7.2 Hz, 3H), 0.79-0.71 (m, 1H), 0.68-0.60 (m, 1H); ^{13}C NMR (100 MHz, $CDCl_3$) δ 149.8, 146.4, 143.4, 137.4, 136.7, 136.5, 133.4, 132.8, 129.6, 129.3, 128.7, 127.3, 127.0, 126.9, 126.4, 21.4, 18.2, 17.7, 15.8, 15.3; ^{29}Si NMR (80 MHz, $CDCl_3$) δ -23.1. HRMS-EI exact mass calcd. for $C_{22}H_{26}SSi^+$ ($[M]^+$) requires m/z 350.1519, found m/z 350.1524.

The enantiomeric excess was determined by its silanol derivative via HPLC on Chiralcel OD-H column (hexane : isopropanol = 99 : 1, flowing rate = 0.5 mL/min, 21 °C, UV detection at λ = 194 nm) t_{R1} = 18.2 min (major), t_{R2} = 25.1 min (minor).

(3',5'-dimethyl-[1,1'-biphenyl]-2-yl)(4-methylthiophen-2-yl)(propyl)silane (**3hi**)

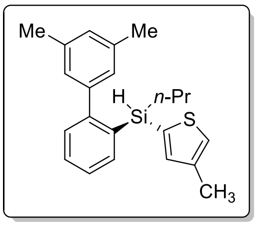

Colorless oil; isolated yield 62%, 81% ee; $[\alpha]_D^{22}$ = -28.0 (c = 1.0, DCM); ^{1}H NMR (400 MHz, $CDCl_3$) δ 7.66 (dd, J_1 = 7.2 Hz, J_2 = 1.6 Hz, 1H), 7.43 (td, J_1 = 7.6 Hz, J_2 = 1.6 Hz, 1H), 7.36-7.29 (m, 2H), 7.17 (s, 1H), 6.97 (d, J = 13.6 Hz, 2H), 6.87 (s, 2H), 4.76 (t, J = 3.6 Hz, 1H), 2.30 (s, 6H), 2.27 (s, 3H), 1.32-1.27 (m, 2H), 0.85 (t, J = 7.2 Hz, 3H), 0.80-0.76 (m, 1H), 0.70-0.62 (m, 1H); ^{13}C NMR (100 MHz, $CDCl_3$) δ 149.9, 143.4, 139.0, 138.8, 137.4, 136.5, 134.9, 133.3, 129.7, 129.3, 128.7, 127.3, 127.2, 126.4, 21.4, 18.2, 17.7, 15.8, 15.2; ^{29}Si NMR (80 MHz, $CDCl_3$) δ -22.7. HRMS-EI exact mass calcd. for $C_{22}H_{26}SSi^+$ ($[M]^+$) requires m/z 350.1519, found m/z 350.1523.

The enantiomeric excess was determined by HPLC on Chiralcel IC column (hexane : isopropanol = 100 : 0, flowing rate = 0.2 mL/min, 22 °C, UV detection at λ = 239 nm) t_{R1} = 26.6 min (major), t_{R2} = 28.2 min (minor).

benzo[b]thiophen-2-yl(3',5'-dimethyl-[1,1'-biphenyl]-2-yl)(propyl)silane (**3hn**)

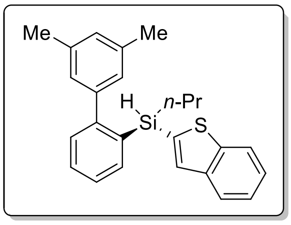

Colorless oil; isolated yield 69%, 84% ee; $[\alpha]_D^{22}$ = -134 (c = 1.0, DCM); ^{1}H NMR (400 MHz, $CDCl_3$) δ 7.88-7.86 (m, 1H), 7.9-7.77 (m, 1H), 7.70 (dd, J_1 = 7.6 Hz, J_2 = 1.6 Hz, 1H), 7.45 (td, J = 7.6 Hz, J_2 =1.6 Hz, 1H), 7.38-7.30 (m, 5H), 6.96 (s, 1H), 6.87 (s, 2H), 4.86 (t, J = 3.6 Hz, 1H), 2.23 (s, 6H), 1.39-1.31 (m, 1H), 0.90-0.86 (m, 4H), 0.79-0.71 (m, 1H); ^{13}C NMR (100 MHz, $CDCl_3$) δ 150.1, 144.2, 143.2, 141.1, 137.5, 137.3, 136.5, 133.4, 132.6, 129.9, 129.4, 128.9, 127.2, 126.5, 124.4, 124.1, 123.6, 122.3, 21.3, 18.2, 17.7, 15.5; ^{29}Si NMR (80 MHz, $CDCl_3$) δ -21.7. HRMS-EI exact mass calcd. for $C_{25}H_{26}SSi^+$ ($[M]^+$) requires m/z 386.1519, found m/z 386.1525.

The enantiomeric excess was determined by HPLC on Chiralcel OD-H column (hexane : isopropanol = 100 : 0, flowing rate = 0.6 mL/min, 35 °C, UV detection at λ = 213 nm) t_{R1} = 23.8 min (minor), t_{R2} = 25.2 min (major).

(2-(3,5-dimethylphenyl)-5-methylthiophen-3-yl)(5-methylthiophen-2-yl)(propyl)silane (**3ia**)

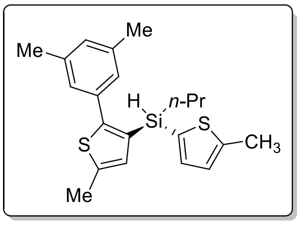

Colorless oil; isolated yield 74%, 90% ee; $[\alpha]_D^{22}$ = +55.6 (*c* = 1.0, DCM); ^{1}H NMR (400 MHz, $CDCl_3$) δ 7.10 (s, 1H), 7.06 (s, 2H), 6.96 (s, 1H), 6.84 (s, 1H), 6.81 (s, 1H), 4.86 (t, *J* = 3.0 Hz, 1H), 2.54 (s, 3H), 2.50 (s, 3H), 2.32 (s, 6H), 1.44-1.36 (m, 2H), 0.98-0.89 (m, 5H); ^{13}C NMR (100 MHz, $CDCl_3$) δ 151.4, 146.6, 139.4, 137.8, 136.8, 135.8, 132.5, 132.4, 131.2, 129.4, 127.3, 127.0, 21.4, 18.1, 17.8, 16.4, 15.3, 15.1; ^{29}Si NMR (80 MHz, $CDCl_3$) δ -30.9. HRMS-APCI exact mass calcd. for $C_{21}H_{25}S_2Si^+$ ($[M-H]^+$) requires m/z 369.1161, found m/z 369.1172.

The enantiomeric excess was determined by HPLC on Chiralcel OD-H column (hexane : isopropanol = 100 : 0, flowing rate = 1.0 mL/min, 35 °C, UV detection at λ = 212 nm) t_{R1} = 13.8 min (major), t_{R2} = 18.5 min (minor).

(2-(3,5-dimethylphenyl)-5-methylthiophen-3-yl)(4-methylthiophen-2-yl)(propyl)silane (**3ii**)

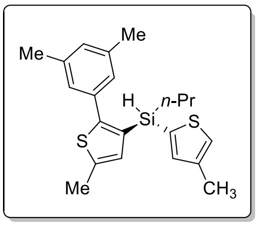

Colorless oil; isolated yield 72%, 91% ee; $[\alpha]_D^{22}$ = +15.2 (*c* = 1.0, DCM); ^{1}H NMR (400 MHz, $CDCl_3$) δ 7.18 (s, 1H), 7.06 (s, 1H), 7.04 (s, 2H), 6.95 (s, 1H), 6.80 (s, 1H), 4.86 (t, *J* = 4.0 Hz, 1H), 2.50 (s, 3H), 2.31 (s, 6H), 2.28 (s, 3H), 1.43-1.34 (m, 2H), 0.99-0.89 (m, 5H); ^{13}C NMR (100 MHz, $CDCl_3$) δ 151.6, 139.4, 139.2, 138.8, 137.8, 135.7, 134.5, 132.4, 131.0, 129.4, 127.3, 127.2, 21.4, 18.1, 17.8, 16.4, 15.2, 15.1; ^{29}Si NMR (80 MHz, $CDCl_3$) δ -30.7. HRMS-APCI exact mass calcd. for $C_{21}H_{27}S_2Si^+$ ($[M+H]^+$) requires m/z 371.1318, found m/z 371.1314.

The enantiomeric excess was determined by HPLC on Chiralcel OD-H column (hexane : isopropanol = 100 : 0, flowing rate = 1.0 mL/min, 35 °C, UV detection at λ = 258 nm) t_{R1} = 13.4 min (major), t_{R2} = 16.1 min (minor).

benzo[b]thiophen-2-yl(2-(3,5-dimethylphenyl)-5-methylthiophen-3-yl)(propyl)silane (**3in**)

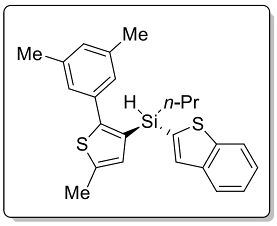

Colorless oil; isolated yield 74%, 93% ee; $[\alpha]_D^{22}$ = +65.4 (*c* = 1.0, DCM); ^{1}H NMR (400 MHz, $CDCl_3$) δ 7.90 (d, *J* = 7.0 Hz, 1H), 7.82 (d, *J* = 7.9 Hz, 1H), 7.52 (s, 1H), 7.38-7.32 (m, 2H), 7.08 (s, 2H), 6.95 (s, 1H), 6.86 (s, 1H), 4.99 (t, *J* = 4.0 Hz, 1H), 2.52 (s, 3H), 2.30 (s, 6H), 1.47 (q, *J* = 7.6 Hz, 2H), 1.09-1.01 (m, 2H), 0.94 (t, *J* = 7.2 Hz, 3H); ^{13}C NMR (100 MHz, $CDCl_3$) δ 152.0, 144.2, 141.1, 139.6, 137.9, 136.9, 135.6, 133.4, 132.4, 130.2, 129.5, 127.3, 124.5, 124.2, 123.7, 122.3, 21.4, 18.2, 17.7, 16.1, 15.1; ^{29}Si NMR (80 MHz, $CDCl_3$) δ -29.5. HRMS-APCI exact mass calcd. for $C_{24}H_{25}S_2Si^+$ ($[M-H]^+$) requires m/z 405.1161, found m/z 405.1170.

The enantiomeric excess was determined by HPLC on Chiralcel OD-H column (hexane : isopropanol = 100 : 0, flowing rate = 1.0 mL/min, 35 °C, UV detection at λ = 228 nm) t_{R1} = 19.1 min (major), t_{R2} = 26.0 min (minor).

(2-ethoxy-6-methylphenyl)(5-methylthiophen-2-yl)(propyl)silane (**3ja**)

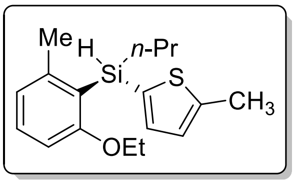

Colorless oil; isolated yield 62%, 91% ee; $[\alpha]_D^{22}$ = -362 (*c* = 1.0, DCM); ^{1}H NMR (400 MHz, $CDCl_3$) δ 7.26-7.17 (m, 2H), 6.80-6.71 (m, 2H), 6.67 (d, *J* = 8.4 Hz, 1H), 5.13 (t, *J* = 4.0 Hz, 1H), 4.09-4.03 (m, 2H), 2.51-2.50 (m, 6H), 1.52-1.46 (m, 2H), 1.42 (t, *J* = 7.2 Hz, 3H), 1.23-1.18 (m, 2H), 0.99 (t, *J* = 7.2 Hz, 6H); ^{13}C NMR (100 MHz, $CDCl_3$) δ 164.0, 146.0, 145.9, 136.7, 132.8, 131.1, 126.6, 123.1, 121.7, 107.7, 63.5, 23.6, 18.7, 17.8, 16.9, 15.2, 14.9; ^{29}Si NMR (80 MHz, $CDCl_3$) δ -30.6. HRMS-EI exact mass calcd. for $C_{17}H_{24}OSSi^+$ ($[M]^+$) requires m/z 304.1312, found m/z 304.1312.

The enantiomeric excess was determined by HPLC on Chiralcel ID-H column (hexane : isopropanol = 100 : 0, flowing rate = 0.2 mL/min, 35 °C, UV detection at λ = 248 nm) t_{R1} = 15.9 min (minor), t_{R2} = 17.5 min (major).

(2-ethoxy-6-methylphenyl)(4-methylthiophen-2-yl)(propyl)silane (**3ji**)

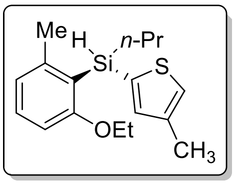

Colorless oil; isolated yield 53%, 90% ee; $[\alpha]_D^{22}$ = -52.0 (*c* = 1.0, DCM); ^{1}H NMR (400 MHz, $CDCl_3$) δ 7.22 (t, *J* = 8.0 Hz, 1H), 7.15-7.11 (m, 2H), 6.77 (d, *J* = 7.6 Hz, 1H), 6.66 (d, *J* = 8.4 Hz, 1H), 5.13 (t, *J* = 4.0 Hz, 1H), 4.07-4.02 (m, 2H), 2.49 (s, 3H), 2.26

(d, J = 1.2 Hz, 3H), 1.51-1.44 (m, 2H), 1.40 (t, J = 7.2 Hz, 3H), 1.23-1.17 (m, 2H), 0.98 (t, J = 7.2 Hz, 3H); ^{13}C NMR (100 MHz, $CDCl_3$) δ 164.1, 146.0, 138.8, 138.7, 135.0, 131.1, 126.8, 123.1, 121.6, 107.7, 63.5, 23.6, 18.7, 17.9, 16.8, 15.2, 14.8; ^{29}Si NMR (80 MHz, $CDCl_3$) δ -30.5. HRMS-EI exact mass calcd. for $C_{17}H_{24}OSSi^+$ ($[M]^+$) requires m/z 304.1312, found m/z 304.1313.

The enantiomeric excess was determined by HPLC on Chiralcel OD3-H column (hexane : isopropanol = 100 : 0, flowing rate = 0.5 mL/min, 24 °C, UV detection at λ = 242 nm) t_{R1} = 19.4 min (major), t_{R2} = 22.0 min (minor).

benzo[b]thiophen-2-yl(2-ethoxy-6-methylphenyl)(propyl) silane (**3jn**)

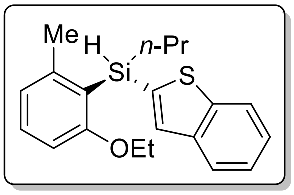

Colorless oil; isolated yield 62%, 96% ee; $[\alpha]_D^{22}$ = -394 (c = 1.0, DCM); ^{1}H NMR (400 MHz, $CDCl_3$) δ 7.90-7.88 (m, 1H), 7.83-7.80 (m, 1H), 7.62 (s, 1H), 7.35-7.27 (m, 2H), 6.85- 6.83 (m, 1H), 6.72 (d, J = 8.4 Hz, 1H), 5.28 (t, J = 4.0 Hz, 1H), 4.13-4.07 (m, 2H), 2.57 (s, 3H), 1.59-1.54 (m, 2H), 1.44 (t, J = 7.2 Hz, 3H), 1.36-1.31 (m, 2H), 1.05 (t, J = 7.2 Hz, 3H); ^{13}C NMR (100 MHz, $CDCl_3$) δ 164.1, 146.1, 144.2, 141.0, 137.6, 133.1, 131.4, 124.2, 123.9, 123.4, 123.2, 122.2, 120.8, 107.8, 63.6, 23.6, 18.7, 17.9, 16.5, 14.8; ^{29}Si NMR (80 MHz, $CDCl_3$) δ -29.3. HRMS-EI exact mass calcd. for $C_{20}H_{24}OSSi^+$ ($[M]^+$) requires m/z 340.1312, found m/z 340.1313.

The enantiomeric excess was determined by HPLC on Chiralcel OD-H column (hexane : isopropanol = 100 : 0, flowing rate = 0.5 mL/min, 35 °C, UV detection at λ = 246 nm) t_{R1} = 22.3 min (major), t_{R2} = 27.7 min (minor).

(2-ethoxy-5-methylphenyl)(5-methylthiophen-2-yl)(propyl)silane (**3ka**)

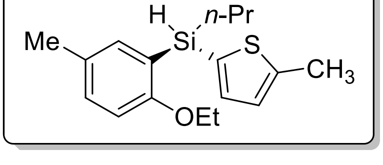

Colorless oil; isolated yield 68%, 89% ee; $[\alpha]_D^{22}$ = +98 (c = 1.0, DCM); ^{1}H NMR (400 MHz, $CDCl_3$) δ 7.29-7.28 (m, 1H), 7.22 (d, J = 3.2 Hz, 1H), 7.18 (d, J = 7.6 Hz, 1H), 6.87 (d, J = 3.2 Hz, 1H), 6.76 (d, J = 8.4 Hz, 1H), 4.92 (t, J = 4.0 Hz, 1H), 4.06 (q, J = 7.2 Hz, 2H), 2.56 (s, 3H), 2.30 (s, 3H), 1.58-1.50 (m, 2H), 1.45 (t, J = 7.2 Hz, 3H), 1.23-1.17 (m, 2H), 1.02 (t, J = 7.2 Hz, 3H); ^{13}C NMR (100 MHz, $CDCl_3$) δ 161.6, 146.2, 137.5, 136.8, 132.1, 132.1, 129.6, 126.8, 123.0, 110.3, 63.6, 20.6, 18.4, 17.9, 16.1, 15.3, 15.0; ^{29}Si NMR (80 MHz, $CDCl_3$) δ -22.0.

HRMS-EI exact mass calcd. for $C_{17}H_{24}OSSi^+$ ($[M]^+$) requires m/z 304.1312, found m/z 304.1313.

The enantiomeric excess was determined by HPLC on Chiralcel OJ-H column (hexane : isopropanol = 100 : 0, flowing rate = 0.3 mL/min, 35 °C, UV detection at λ = 257 nm) t_{R1} = 15.3 min (minor), t_{R2} = 16.8 min (major).

(2-ethoxy-5-methylphenyl)(5-methylthiophen-2-yl)(propyl)silane (3ki)

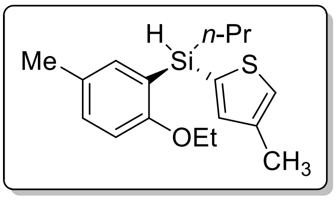

Colorless oil; isolated yield 54%, 91% ee; $[\alpha]_D^{22}$ = +83.0 (c = 1.0, DCM); ^{1}H NMR (400 MHz, $CDCl_3$) δ 7.29- 7.28 (m, 1H), 7.22 (d, J = 3.2 Hz, 1H), 7.18 (d, J = 7.6 Hz, 1H), 6.87 (d, J = 3.2 Hz, 1H), 6.76 (d, J = 8.4 Hz, 1H), 4.92 (t, J = 4.0 Hz, 1H), 4.06 (q, J = 7.2 Hz, 2H), 2.34 (s, 3H), 2.28 (s, 3H), 1.49 (q, J = 7.6 Hz, 2H), 1.41 (t, J = 7.2 Hz, 3H), 1.9-1.12 (m, 1H), 0.98 (t, J = 7.2 Hz, 3H); ^{13}C NMR (100 MHz, $CDCl_3$) δ 163.8, 142.2, 138.9, 138.8, 136.9, 134.5, 127.0, 121.4, 119.5, 111.4, 63.4, 22.0, 18.4, 17.9, 16.0, 15.2, 14.9; ^{29}Si NMR (80 MHz, $CDCl_3$) δ -22.3. HRMS-EI exact mass calcd. for $C_{17}H_{24}OSSi^+$ ($[M]^+$) requires m/z 304.1312, found m/z 304.1313.

The enantiomeric excess was determined by HPLC on Chiralcel OJ-H column (hexane : isopropanol = 100 : 0, flowing rate = 0.3 mL/min, 35 °C, UV detection at λ = 247 nm) t_{R1} = 14.9 min (minor), t_{R2} = 16.8 min (major).

benzo[b]thiophen-2-yl(2-ethoxy-5-methylphenyl)(propyl)silane (3kn)

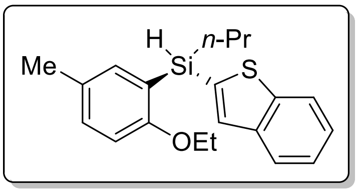

Colorless oil; isolated yield 60%, 94% ee; $[\alpha]_D^{22}$ = +158 (c = 1.0, DCM); ^{1}H NMR (400 MHz, $CDCl_3$) δ 7.91-7.89 (m, 1H), 7.84-7.81 (m, 1H), 7.62 (s, 1H), 7.35-7.30 (m, 3H), 7.18 (dd, J_I = 8.4 Hz, J_I = 2.4 Hz, 1H), 6.76 (d, J = 8.4 Hz, 1H), 5.02 (t, J = 3.6 Hz, 1H), 4.05 (q, J = 7.2 Hz, 2H), 2.28 (s, 3H), 1.61-1.52 (m, 2H), 1.42 (t, J = 7.2 Hz, 3H), 1.30-1.24 (m, 2H), 1.03 (t, J = 7.2 Hz, 3H); ^{13}C NMR (100 MHz, $CDCl_3$) δ 161.7, 144.3, 141.1, 137.6, 136.8, 133.4, 132.4, 129.7, 124.3, 124.0, 123.6, 122.2, 122.0, 110.3, 63.7, 20.6, 18.4, 17.9, 15.7, 14.9; ^{29}Si NMR (80 MHz, $CDCl_3$) δ -22.6. HRMS-EI exact mass calcd. for $C_{20}H_{24}OSSi^+$ ($[M]^+$) requires m/z 340.1312, found m/z 340.1314.

The enantiomeric excess was determined by HPLC on Chiralcel AD-H column (hexane :

isopropanol = 100 : 0, flowing rate = 0.5 mL/min, 35 °C, UV detection at λ = 227 nm) t_{R1} = 11.6 min (minor), t_{R2} = 13.9 min (major).

(2-ethoxy-4-methylphenyl)(5-methylthiophen-2-yl)(propyl)silane (**3la**)

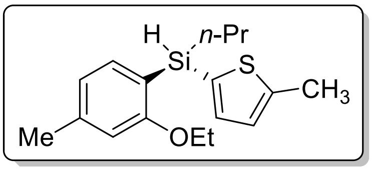

Colorless oil; isolated yield 61%, 91% ee; $[\alpha]_D^{22}$ = +114 (c = 1.0, DCM); ^{1}H NMR (400 MHz, $CDCl_3$) δ 7.30 (d, J = 7.2 Hz, 1H), 7.16 (d, J = 3.2 Hz, 1H), 6.82 (dd, J_1 = 3.2 Hz, J_2 = 1.2 Hz, 1H), 6.74 (d, J = 6.8 Hz, 1H), 6.64 (s, 1H), 4.87 (t, J = 4.0 Hz, 1H), 4.04 (q, J = 7.2 Hz, 2H), 2.51 (s, 3H), 2.33 (s, 3H), 1.51-1.45 (m, 2H), 1.41 (t, J = 7.2 Hz, 3H), 1.17-1.11 (m, 2H), 0.97 (t, J = 7.2 Hz, 3H); ^{13}C NMR (100 MHz, $CDCl_3$) δ 163.8, 146.2, 142.1, 136.9, 136.7, 132.2, 126.8, 121.4, 119.6, 111.3, 63.4, 22.0, 18.4, 17.9, 16.0, 15.3, 15.0; ^{29}Si NMR (80 MHz, $CDCl_3$) δ -22.7. HRMS-EI exact mass calcd. for $C_{17}H_{24}OSSi^+$ ($[M]^+$) requires m/z 304.1312, found m/z 304.1314.

The enantiomeric excess was determined by HPLC on Chiralcel OD-H amd AD-H column (hexane : isopropanol = 99.9 : 0.1, flowing rate = 0.5 mL/min, 35 °C, UV detection at λ = 212 nm) t_{R1} = 24.8 min (minor), t_{R2} = 26.0 min (major).

(2-ethoxy-4-methylphenyl)(4-methylthiophen-2-yl)(propyl)silane (**3li**)

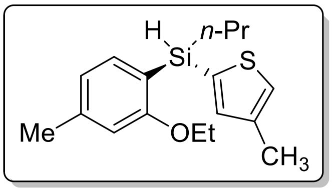

Colorless oil; isolated yield 65%, 89% ee; $[\alpha]_D^{22}$ = +156 (c = 1.0, DCM); ^{1}H NMR (400 MHz, $CDCl_3$) δ 7.30 (d, J = 7.2 Hz, 1H), 7.16 (s, 2H), 6.75 (d, J = 7.2 Hz, 1H), 6.64 (s, 1H), 4.89 (t, J = 3.6 Hz, 1H), 4.04 (q, J = 7.2 Hz, 2H), 2.34 (s, 3H), 2.28 (s, 3H), 1.49 (q, J = 7.6 Hz, 2H), 1.41 (t, J = 7.2 Hz, 3H), 1.9-1.12 (m, 1H), 0.98 (t, J = 7.2 Hz, 3H); ^{13}C NMR (100 MHz, $CDCl_3$) δ 163.8, 142.2, 138.9, 138.8, 136.9, 134.5, 127.0, 121.4, 119.5, 111.4, 63.4, 22.0, 18.4, 17.9, 16.0, 15.2, 14.9; ^{29}Si NMR (80 MHz, $CDCl_3$) δ -22.4. HRMS-EI exact mass calcd. for $C_{17}H_{24}OSSi^+$ ($[M]^+$) requires m/z 304.1312, found m/z 304.1313.

The enantiomeric excess was determined by HPLC on Chiralcel OD3-H column (hexane : isopropanol = 100 : 0, flowing rate = 0.5 mL/min, 24 °C, UV detection at λ = 224 nm) t_{R1} = 18.3 min (minor), t_{R2} = 20.1 min (major).

benzo[b]thiophen-2-yl(2-ethoxy-4-methylphenyl)(propyl)silane (**3ln**)

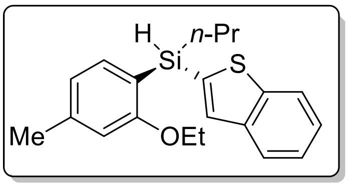

Colorless oil; isolated yield 62%, 90% ee; $[\alpha]_D^{22}$ = +178 (*c* = 1.0, DCM); ^{1}H NMR (400 MHz, $CDCl_3$) δ 7.89-7.86 (m, 1H), 7.82-7.79 (m, 1H), 7.60 (s, 1H), 7.36 (d, *J* = 7.2 Hz, 1H), 7.35-7.30 (m, 2H), 6.77 (d, *J* = 7.6 Hz, 1H), 6.67 (s, 1H), 5.00 (t, *J* = 3.6 Hz, 1H), 4.06 (q, *J* = 7.2 Hz, 2H), 2.35 (s, 3H), 1.7-1.51 (m, 2H), 1.41 (t, *J* = 7.2 Hz, 3H), 1.27-1.21 (m, 2H), 1.01 (t, *J* = 7.2 Hz, 3H); ^{13}C NMR (100 MHz, $CDCl_3$) δ 163.9, 144.2, 142.5, 141.1, 137.0, 137.0, 133.3, 124.3, 124.0, 123.6, 122.2, 121.5, 118.6, 111.4, 63.5, 22.0, 18.4, 17.9, 15.7, 14.9; ^{29}Si NMR (80 MHz, $CDCl_3$) δ -21.2. HRMS-EI exact mass calcd. for $C_{20}H_{24}OSSi^+$ ($[M]^+$) requires m/z 340.1312, found m/z 340.1312.

The enantiomeric excess was determined by HPLC on Chiralcel AD-H column (hexane : isopropanol = 99.9 : 0.1, flowing rate = 1.0 mL/min, 35 °C, UV detection at λ = 239 nm) t_{R1} = 6.4 min (minor), t_{R2} = 8.0 min (major).

(2-ethoxy-3-methylphenyl)(5-methylthiophen-2-yl)(propyl)silane (**3ma**)

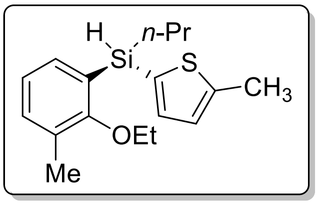

Colorless oil; isolated yield 72%, 94% ee; $[\alpha]_D^{22}$ = -66.0 (*c* = 1.0, DCM); ^{1}H NMR (400 MHz, $CDCl_3$) δ 7.23 (d, *J* = 9.2 Hz, 2H), 7.16 (d, *J* = 3.2 Hz, 1H), 7.01 (t, *J* = 7.2 Hz, 1H), 6.85 (d, *J* = 3.2 Hz, 1H), 5.00 (t, *J* = 3.6 Hz, 1H), 3.88-3.81 (m, 2H), 2.53 (s, 3H), 2.29 (s, 3H), 1.55-1.49 (m, 2H), 1.38 (t, *J* = 7.2 Hz, 3H), 1.18-1.14 (m, 2H), 1.00 (t, *J* = 7.2 Hz, 3H); ^{13}C NMR (100 MHz, $CDCl_3$) δ 162.8, 146.6, 136.9, 134.4, 133.8, 131.8, 130.4, 127.9, 127.1, 124.1, 69.1, 18.4, 17.9, 16.6, 15.9, 15.6, 15.3; ^{29}Si NMR (80 MHz, $CDCl_3$) δ -26.3. HRMS-EI exact mass calcd. for $C_{17}H_{24}OSSi^+$ ($[M]^+$) requires m/z 304.1312, found m/z 304.1317.

The enantiomeric excess was determined by HPLC on Chiralcel OJ-H column (hexane : isopropanol = 100 : 0, flowing rate = 0.2 mL/min, 23 °C, UV detection at λ = 235 nm) t_{R1} = 25.4 min (minor), t_{R2} = 29.2 min (major).

(2-ethoxy-3-methylphenyl)(4-methylthiophen-2-yl)(propyl) silane (**3mi**)

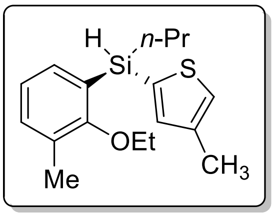

Colorless oil; isolated yield 60%, 92% ee; $[\alpha]_D^{22}$ = -110 (*c* = 1.0, DCM); ^{1}H NMR (400 MHz, $CDCl_3$) δ 7.24 (t, *J* = 7.2 Hz, 2H), 7.19 (s, 1H), 7.15 (s, 1H), 7.01 (t, *J* = 7.2 Hz, 1H), 5.01 (t, *J* = 3.6 Hz, 1H), 3.87-3.79 (m, 2H), 2.29 (s, 6H), 1.53-1.49 (m, 2H), 1.38 (t, *J* = 7.2 Hz, 3H), 1.19-1.14 (m, 2H), 1.00 (t, *J* = 7.2 Hz, 3H); ^{13}C NMR (100 MHz, $CDCl_3$) δ 162.8, 139.2, 139.0, 134.4, 134.0, 133.9, 130.4, 127.8, 127.3, 124.1, 69.1, 18.4, 17.9, 16.6, 15.9, 15.6, 15.2; ^{29}Si NMR (80 MHz, $CDCl_3$) δ -26.0. HRMS-EI exact mass calcd. for $C_{17}H_{24}OSSi^+$ ($[M]^+$) requires m/z 304.1312, found m/z 304.1311.

The enantiomeric excess was determined by HPLC on Chiralcel OJ-H column (hexane : isopropanol = 99.9 : 0.1, flowing rate = 0.2 mL/min, 35 °C, UV detection at λ = 243 nm) t_{R1} = 21.2 min (minor), t_{R2} = 22.8 min (major).

benzo[b]thiophen-2-yl(2-ethoxy-3-methylphenyl)(propyl) silane (**3mn**)

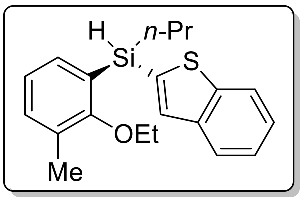

Colorless oil; isolated yield 65%, 87% ee; $[\alpha]_D^{22}$ = -134 (*c* = 1.0, DCM); ^{1}H NMR (400 MHz, $CDCl_3$) δ 7.94-7.91 (m, 1H), 7.87-7.85 (m, 1H), 7.64 (s, 1H), 7.38-7.33 (m, 3H), 7.30 (d, *J* = 6.4 Hz, 1H), 7.07 (t, *J* = 7.2 Hz, 1H), 5.16 (t, *J* = 3.6 Hz, 1H), 3.4-3.88 (m, 2H), 2.35 (s, 3H), 1.64-1.57 (m, 2H), 1.43 (t, *J* = 7.2 Hz, 3H), 1.32-1.27 (m, 2H), 1.07 (t, *J* = 7.2 Hz, 3H); ^{13}C NMR (100 MHz, $CDCl_3$) δ 162.9, 144.2, 141.1, 136.5, 134.4, 134.2, 133.6, 130.5, 127.0, 124.5, 124.2, 124.1, 123.7, 122.3, 69.1, 18.4, 17.9, 16.6, 15.7, 15.6; ^{29}Si NMR (80 MHz, $CDCl_3$) δ -24.8. HRMS-EI exact mass calcd. for $C_{20}H_{24}OSSi^+$ ($[M]^+$) requires m/z 340.1312, found m/z 340.1317.

The enantiomeric excess was determined by HPLC on Chiralcel OD3-H column (hexane : isopropanol = 100 : 0, flowing rate = 0.5 mL/min, 24 °C, UV detection at λ = 238 nm) t_{R1} = 20.0 min (major), t_{R2} = 21.8 min (minor).

(2-ethoxy-4-fluorophenyl)(5-methylthiophen-2-yl)(propyl)silane (**3na**)

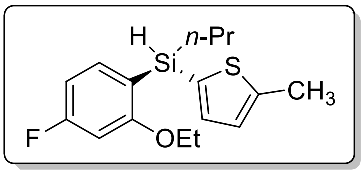

Colorless oil; isolated yield 63%, 89% ee; $[\alpha]_D^{22}$ = +38.0 (*c* = 1.0, DCM); ^{1}H NMR (400 MHz, $CDCl_3$) δ 7.36 (t, *J* = 8.0 Hz, 1H), 7.16 (d, *J* = 3.2 Hz, 1H), 6.83 (dd, J_1 = 3.2 Hz, J_2 =1.2 Hz, 1H), 6.62 (td, *J* = 8.4 Hz, J_2 = 2.0 Hz,

1H), 6.53 (dd, *J* = 11.2 Hz, J_2 = 2.0 Hz, 1H), 4.87 (t, *J* = 3.6 Hz, 1H), 4.02 (q, *J* = 7.2 Hz, 2H), 1.51-1.41 (m, 5H), 1.17 -1.11 (m, 2H), 0.98 (t, *J* = 7.2 Hz, 3H); ^{13}C NMR (100 MHz, $CDCl_3$) δ 166.1 (d, *J* = 194 Hz), 164.8 (d, *J* = 42 Hz), 146.5, 138.0 (d, *J* = 10 Hz), 136.9, 131.6, 126.9, 118.5 (d, *J* = 3.0 Hz), 107.3 (d, *J* = 20 Hz), 98.8 (d, *J* = 24 Hz), 63.9, 18.3, 17.8, 15.8, 15.3, 14.7; ^{19}F NMR (376 MHz, $CDCl_3$) δ -108.6; ^{29}Si NMR (80 MHz, $CDCl_3$) δ -21.6. HRMS-EI exact mass calcd. for $C_{16}H_{21}OFSSi^+$ ($[M]^+$) requires m/z 308.1061, found m/z 308.1064.

The enantiomeric excess was determined by HPLC on Chiralcel OD3-H column (hexane : isopropanol = 100 : 0, flowing rate = 0.5 mL/min, 23 °C, UV detection at λ = 227 nm) t_{R1} = 14.8 min (major), t_{R2} = 15.5 min (minor).

(2-ethoxy-4-fluorophenyl)(4-methylthiophen-2-yl)(propyl)silane (**3ni**)

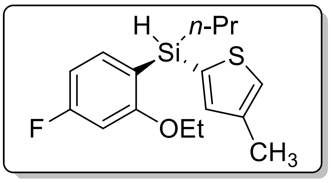

Colorless oil; isolated yield 67%, 90% ee; $[\alpha]_D^{22}$ = +94.0 (*c* = 1.0, DCM); ^{1}H NMR (400 MHz, $CDCl_3$) δ 7.36 (t, *J* = 7.6 Hz, 1H), 7.17 (d, *J* = 8.0 Hz, 1H), 6.62 (td, J_1 = 8.4 Hz, J_2 = 2.4 Hz, 1H), 6.54 (dd, J_1 = 7.6 Hz, J_2 = 2.0 Hz, 1H), 4.89 (t, *J* = 3.6 Hz, 1H), 4.02 (q, *J* = 7.2 Hz, 2H), 2.29 (s, 3H), 1.51-1.41 (m, 5H), 1.18-1.12 (m, 2H), 0.98 (t, *J* = 7.2 Hz, 3H); ^{13}C NMR (100 MHz, $CDCl_3$) δ 166.1 (d, *J* = 196 Hz), 164.8 (d, *J* = 41 Hz), 139.0 (d, *J* = 4.0 Hz), 138.0 (d, *J* = 10 Hz), 133.8, 127.2, 118.3, 107.3 (d, *J* = 20 Hz), 98.8 (d, *J* = 24 Hz), 63.9, 18.3, 17.9, 15.8, 15.2, 14.7; ^{19}F NMR (376 MHz, $CDCl_3$) δ -108.6; ^{29}Si NMR (80 MHz, $CDCl_3$) δ -22.4. HRMS-EI exact mass calcd. for $C_{16}H_{21}OFSSi^+$ ($[M]^+$) requires m/z 308.1061, found m/z 308.1063.

The enantiomeric excess was determined by HPLC on Chiralcel OJ-H and ID-H column (hexane : isopropanol = 100 : 0, flowing rate = 0.5 mL/min, 35 °C, UV detection at λ = 236 nm) t_{R1} = 19.5 min (minor), t_{R2} = 20.8 min (major).

benzo[b]thiophen-2-yl(2-ethoxy-4-fluorophenyl)(propyl)silane (**3nn**)

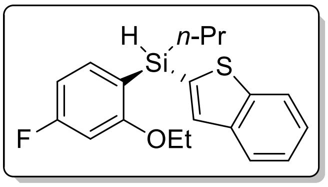

Colorless oil; isolated yield 62%, 88% ee; $[\alpha]_D^{22}$ = +174 (*c* = 1.0, DCM); ^{1}H NMR (400 MHz, $CDCl_3$) δ 7.89- 7.87 (m, 1H), 7.83-7.80 (m, 1H), 7.60 (s, 1H), 7.41 (t, *J* = 7.6 Hz, 1H), 7.35-7.31 (m, 2H), 6.64 (td, J_1 = 8.4 Hz, J_2 = 2.4 Hz, 1H), 6.56 (dd, J_1 = 11.2 z, J_2 = 2.0 Hz, 1H), 4.99 (t, *J* = 3.6 Hz, 1H), 4.04 (q, *J* = 7.2

Hz, 2H), 1.56-1.50 (m, 2H), 1.43 (t, J = 7.2 Hz, 2H), 1.26-1.21 (m, 2H), 1.01 (t, J = 7.2 Hz, 3H); ^{13}C NMR (100 MHz, $CDCl_3$) δ 166.2 (d, J = 202 Hz), 164.9 (d, J = 35 Hz), 144.2, 141.0, 138.1 (d, J = 10 Hz), 136.2, 133.5, 124.4, 124.1, 123.6, 122.3, 117.6 (d, J = 3.0 Hz), 107.4 (d, J = 20 Hz), 98.9 (d, J = 24 Hz), 64.0, 18.3, 17.9, 15.5, 14.7; ^{19}F NMR (376 MHz, $CDCl_3$) δ -108.0; ^{29}Si NMR (80 MHz, $CDCl_3$) δ -21.3. HRMS-EI exact mass calcd. for $C_{19}H_{21}OFSSi^+$ ($[M]^+$) requires m/z 344.1061, found m/z 344.1064. The enantiomeric excess was determined by HPLC on Chiralcel AD-H column (hexane : isopropanol = 99.9 : 0.1, flowing rate = 0.5 mL/min, 35 °C, UV detection at λ = 232 nm) t_{R1} = 13.7 min (minor), t_{R2} = 15.2 min (major).

(4-chloro-2-ethoxyphenyl)(5-methylthiophen-2-yl)(pro pyl)silane (**3oa**)

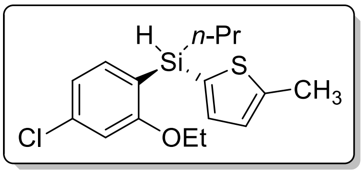

Colorless oil; isolated yield 66%, 91% ee; $[\alpha]_D^{22}$ = +60.0 (c = 1.0, DCM); ^{1}H NMR (400 MHz, $CDCl_3$) δ 7.32 (d, J = 8.0 Hz, 1H), 7.16 (d, J = 3.2 Hz, 1H), 6.90 (dd, J_1 = 7.6 Hz, J_2 = 1.6 Hz, 1H), 6.83 (d, J = 3.2 Hz, 1H), 6.79 (d, J = 2.0 Hz, 1H), 4.87 (t, J = 3.6 Hz, 1H), 4.03 (q, J = 7.2 Hz, 2H), 2.52 (s, 3H), 1.50-1.41 (m, 5H), 1.17-1.11 (m, 2H), 0.97 (t, J = 7.2 Hz, 3H); ^{13}C NMR (100 MHz, $CDCl_3$) δ 164.2, 146.6, 137.7, 137.3, 137.0, 131.2, 126.9, 121.7, 120.7, 111.1, 63.9, 18.3, 17.9, 15.7, 15.3, 14.8; ^{29}Si NMR (80 MHz, $CDCl_3$) δ -22.6. HRMS-EI exact mass calcd. for $C_{16}H_{21}OClSSi^+$ ($[M]^+$) requires m/z 324.0765, found m/z 324.0768.

The enantiomeric excess was determined by HPLC on Chiralcel OD3-H column (hexane : isopropanol = 100 : 0, flowing rate = 0.3 mL/min, 24 °C, UV detection at λ = 238 nm) t_{R1} = 24.2 min (major), t_{R2} = 25.5 min (minor).

(4-chloro-2-ethoxyphenyl)(4-methylthiophen-2-yl)(propyl)silane (**3oi**)

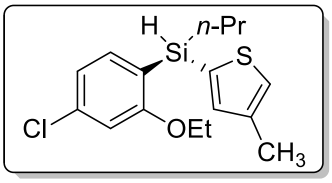

Colorless oil; isolated yield 64%, 90% ee; $[\alpha]_D^{22}$ = +52.0 (c = 1.0, DCM); ^{1}H NMR (400 MHz, $CDCl_3$) δ 7.32 (d, J = 7.6 Hz, 1H), 7.18 (s, 1H), 7.15 (s, 1H), 6.90 (dd, J_1 = 7.6 Hz, J_2 = 1.7 Hz, 1H), 6.79 (d, J = 1.6 Hz, 1H), 4.88 (t, J = 3.6 Hz, 1H), 4.03 (q, J = 7.2 Hz, 2H), 2.28 (d, J = 0.8 Hz, 3H), 1.51–1.39 (m, 5H), 1.19–1.11 (m, 2H), 0.98 (t, J = 7.2 Hz, 3H); ^{13}C NMR (100 MHz, $CDCl_3$) δ 164.2, 139.1, 139.1, 137.7, 137.4, 133.4, 127.3, 121.5, 120.7, 111.1, 63.9, 18.3, 17.9, 15.7,

15.2, 14.7; ^{29}Si NMR (80 MHz, $CDCl_3$) δ -22.2. HRMS-EI exact mass calcd. for $C_{16}H_{21}OClSSi^+$ ($[M]^+$) requires m/z 324.0765, found m/z 324.0768.

The enantiomeric excess was determined by HPLC on Chiralcel OD3-H column (hexane : isopropanol = 100 : 0, flowing rate = 0.5 mL/min, 23 °C, UV detection at λ = 235 nm) t_{R1} = 14.8 min (major), t_{R2} = 15.9 min (minor).

benzo[b]thiophen-2-yl(4-chloro-2-ethoxyphenyl)(propyl)silane (**3on**)

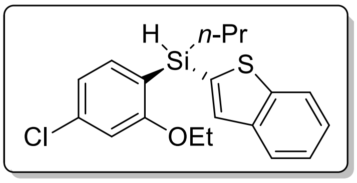

Colorless oil; isolated yield 67%, 87% ee; $[\alpha]_D^{22}$ = +80.0 (*c* = 1.0, DCM); ^{1}H NMR (400 MHz, $CDCl_3$) δ 7.93–7.85 (m, 1H), 7.84–7.77 (m, 1H), 7.59 (s, 1H), 7.41–7.28 (m, 3H), 6.92 (dd, J_1 = 7.6 Hz, J_2 = 1.6 Hz, 1H), 6.82 (d, *J* = 1.6 Hz, 1H), 4.99 (t, *J* = 3.6 Hz, 1H), 4.05 (q, *J* = 7.2 Hz, 2H), 1.57-1.50 (m, 2H), 1.42 (t, *J* = 7.2 Hz, 3H), 1.26–1.21 (m, 2H), 1.01 (t, *J* = 7.2 Hz, 3H); ^{13}C NMR (100 MHz, $CDCl_3$) δ 164.3, 144.2, 141.0, 137.8, 137.7, 135.9, 133.6, 124.5, 124.1, 123.7, 122.3, 120.9,120.7, 111.2, 64.0, 18.3, 17.9, 15.4, 14.8; ^{29}Si NMR (80 MHz, $CDCl_3$) δ -21.2. HRMS-EI exact mass calcd. for $C_{19}H_{21}OClSSi^+$ ($[M]^+$) requires m/z 360.0765, found m/z 360.0762.

The enantiomeric excess was determined by HPLC on Chiralcel OD-H column (hexane : isopropanol = 100 : 0, flowing rate = 0.5 mL/min, 35 °C, UV detection at λ = 276 nm) t_{R1} = 24.3 min (major), t_{R2} = 26.2 min (minor).

(2-ethoxy-4-methoxyphenyl)(5-methylthiophen-2-yl)(propyl)silane (**3pa**)

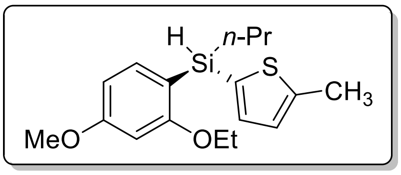

Colorless oil; isolated yield 59%, 92% ee; $[\alpha]_D^{22}$ = -46.0 (*c* = 1.0, DCM); ^{1}H NMR (400 MHz, $CDCl_3$) δ 7.32 (d, *J* = 8.0 Hz, 1H), 7.16 (d, *J* = 3.2 Hz, 1H), 6.82 (d, *J* = 2.4 Hz, 1H), 6.46 (dd, J_1 = 8.0 Hz, J_2 = 2.0 Hz, 1H), 6.39 (d, *J* = 1.6 Hz, 1H), 4.86 (t, *J* = 3.6 Hz, 1H), 4.02 (q, *J* = 7.2 Hz, 2H), 3.80 (s, 3H), 2.52 (s, 3H), 1.48 (dd, J_1 = 15.2 Hz, J_2 = 7.6 Hz, 2H), 1.41 (t, *J* = 7.2 Hz, 3H), 1.16-1.10 (m, 2H), 0.97 (t, *J* = 7.2 Hz, 3H); ^{13}C NMR (100 MHz, $CDCl_3$) δ 165.0, 163.2, 146.1, 137.8, 136.7, 132.4, 126.8, 114.5, 104.8, 98.4, 63.5, 55.4, 18.4, 17.9, 16.1, 15.3, 14.8; ^{29}Si NMR (80 MHz, $CDCl_3$) δ -23.0. HRMS-EI exact mass calcd. for $C_{17}H_{24}O_2SSi^+$ ($[M]^+$) requires m/z 320.1261, found m/z 320.1262.

The enantiomeric excess was determined by HPLC on Chiralcel OD-H column (hexane : isopropanol = 99.0 : 1.0, flowing rate = 1.0 mL/min, 35 ºC, UV detection at λ = 254 nm) t_{R1} = 5.4 min (minor), t_{R2} = 6.0 min (major).

(2-ethoxy-4-methoxyphenyl)(4-methylthiophen-2-yl)(propyl)silane (**3pi**)

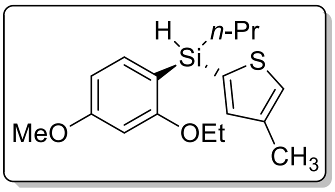

Colorless oil; isolated yield 53%, 88% ee; $[\alpha]_D^{22}$ = +216 (*c* = 1.0, DCM); ^{1}H NMR (400 MHz, $CDCl_3$) δ 7.33 (d, *J* = 8.0 Hz, 1H), 7.19-7.12 (m, 2H), 6.47 (dd, J_1 = 8.0 Hz, J_2 = 2.0 Hz, 1H), 6.39 (d, *J* = 2.0 Hz, 1H), 4.87 (t, *J* = 3.6 Hz, 1H), 4.02 (q, *J* = 7.2 Hz, 2H), 3.80 (s, 3H), 2.28 (s, 3H), 1.51-1.47 (m, 2H), 1.41 (t, *J* = 7.2 Hz, 3H), 1.17-1.11 (m, 2H), 0.98 (t, *J* = 7.2 Hz, 3H); ^{13}C NMR (100 MHz, $CDCl_3$) δ 165.1, 163.2, 138.9, 138.8, 137.9, 134.6, 126.9, 114.3, 104.8, 98.4, 63.5, 55.4, 29.9, 18.4, 17.9, 16.1, 15.2, 14.8; ^{29}Si NMR (80 MHz, $CDCl_3$) δ -22.7. HRMS-EI exact mass calcd. for $C_{17}H_{24}O_2SSi^+$ ($[M]^+$) requires m/z 320.1261, found m/z 320.1264.

The enantiomeric excess was determined by HPLC on Chiralcel OD-H column (hexane : isopropanol = 99.0 : 1.0, flowing rate = 1.0 mL/min, 35 ºC, UV detection at λ = 258 nm) t_{R1} = 5.5 min (minor), t_{R2} = 6.0 min (major).

benzo[b]thiophen-2-yl(2-ethoxy-4-methoxyphenyl) (propyl)silane (**3pn**)

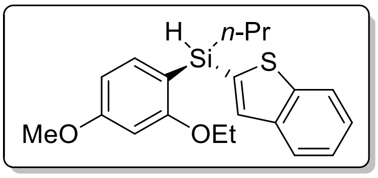

White solid; isolated yield 49%, 89% ee; $[\alpha]_D^{22}$ = +88.0 (*c* = 1.0, DCM); ^{1}H NMR (400 MHz, $CDCl_3$) δ 7.90-7.84 (m, 1H), 7.80 (dd, J_1 = 6.4 Hz, J_2 =2.4 Hz, 1H), 7.58 (s, 1H), 7.38 (d, *J* = 8.0 Hz, 1H), 7.35-7.27 (m, 2H), 6.48 (dd, J_1 = 8.0 Hz, J_2 = 2.0 Hz, 1H), 6.41 (d, *J* = 2.0 Hz, 1H), 4.98 (t, *J* = 3.6 Hz, 1H), 4.04 (q, *J* = 7.2 Hz, 2H), 3.81 (s, 3H), 1.56-1.50 (m, 2H), 1.41 (t, *J* = 7.2 Hz, 3H), 1.25-1.20 (m, 2H), 1.01 (t, *J* = 7.2 Hz, 3H); ^{13}C NMR (100 MHz, $CDCl_3$) δ 165.1, 163.4, 144.2, 141.1, 138.0, 137.2, 133.2, 124.3, 124.0, 123.6, 122.2, 113.5, 104.9, 98.4, 63.6, 55.4, 18.4, 17.9, 15.8, 14.8; ^{29}Si NMR (80 MHz, $CDCl_3$) δ -21.6. HRMS-EI exact mass calcd. for $C_{20}H_{24}O_2SSi^+$ ($[M]^+$) requires m/z 356.1261, found m/z 356.1264.

The enantiomeric excess was determined by HPLC on Chiralcel OD-H column (hexane : isopropanol = 99.0 : 1.0, flowing rate = 1.0 mL/min, 35 ºC, UV detection at λ = 213 nm) t_{R1} = 6.4 min (minor), t_{R2} = 8.3 min (major).

(2-ethoxy-4-(trifluoromethoxy)phenyl)(5-methylthiophen-2-yl)(propyl)silane (**3qa**)

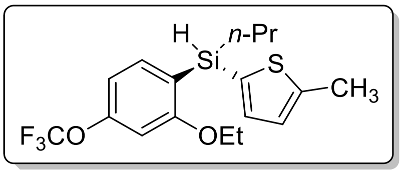

Colorless oil; isolated yield 68%, 86% ee; $[\alpha]_D^{22}$ = +88.0 (*c* = 1.0, DCM); ^{1}H NMR (400 MHz, $CDCl_3$) δ 7.41 (d, *J* = 8.0 Hz, 1H), 7.18 (d, *J* = 3.2 Hz, 1H), 6.86-6.81 (m, 1H), 6.77 (d, *J* = 8.0 Hz, 1H), 6.63 (s, 1H), 4.89 (t, *J* = 3.6 Hz, 1H), 4.05 (q, *J* = 7.2 Hz, 2H), 2.53 (s, 3H), 1.49-1.40 (m, 5H), 1.19-1.13 (m, 2H), 0.99 (t, *J* = 7.2 Hz, 3H); ^{13}C NMR (100 MHz, $CDCl_3$) δ 164.6, 152.3 (d, *J* = 2.0 Hz), 146.7, 137.8, 137.1, 131.1, 127.0, 120.6 (q, *J* = 255 Hz), 112.4, 103.6, 64.0, 18.3, 17.8, 15.7, 15.3, 14.7; ^{19}F NMR (376 MHz, $CDCl_3$) δ -57.5; ^{29}Si NMR (80 MHz, $CDCl_3$) δ -22.6. HRMS-EI exact mass calcd. for $C_{17}H_{21}O_2F_3SSi^+$ ($[M]^+$) requires m/z 374.0978, found m/z 374.0982.

The enantiomeric excess was determined by HPLC on Chiralcel OD-H column (hexane : isopropanol = 100 : 0, flowing rate = 0.4 mL/min, 35 °C, UV detection at λ = 239 nm) t_{R1} = 12.0 min (major), t_{R2} = 13.2 min (minor).

(2-ethoxy-4-(trifluoromethoxy)phenyl)(4-methylthiophen-2-yl)(propyl)silane (**3qi**)

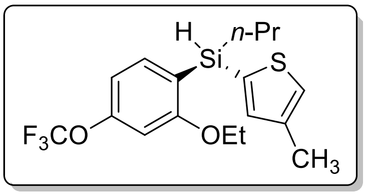

Colorless oil; isolated yield 66%, 88% ee; $[\alpha]_D^{22}$ = -8.0 (*c* = 1.0, DCM); ^{1}H NMR (400 MHz, $CDCl_3$) δ 7.41 (d, *J* = 8.0 Hz, 1H), 7.18 (d, *J* = 8.0 Hz, 2H), 6.78 (d, *J* = 8.0 Hz, 1H), 6.64 (s, 1H), 4.90 (t, *J* = 3.6 Hz, 1H), 4.05 (q, *J* = 7.2 Hz, 2H), 2.29 (s, 3H), 1.52-1.42 (m, 5H), 1.20-1.14 (m, 2H), 0.99 (t, *J* = 7.2 Hz, 3H); ^{13}C NMR (100 MHz, $CDCl_3$) δ 164.6, 152.3, 139.1 (d, *J* = 5.0 Hz), 137.8, 133.3, 127.4, 126.7, 124.4, 121.9, 120.6 (q, *J* = 243 Hz), 116.8, 112.4, 103.6, 64.0, 18.3, 17.8, 15.7, 15.2, 14.7; ^{19}F NMR (376 MHz, $CDCl_3$) δ -57.5; ^{29}Si NMR (80 MHz, $CDCl_3$) δ -22.3. HRMS-EI exact mass calcd. for $C_{17}H_{21}O_2F_3SSi^+$ ($[M]^+$) requires m/z 374.0978, found m/z 374.0983.

The enantiomeric excess was determined by HPLC on Chiralcel OD-H column (hexane : isopropanol = 100 : 0, flowing rate = 0.4 mL/min, 35 °C, UV detection at λ = 256 nm) t_{R1} = 12.2 min (major), t_{R2} = 13.2 min (minor).

benzo[b]thiophen-2-yl(2-ethoxy-4-(trifluoromethoxy)phenyl)(propyl)silane (**3qn**)

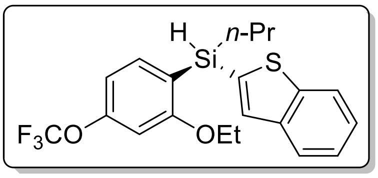

Colorless oil; isolated yield 66%, 91% ee; $[\alpha]_D^{22}$ = +180 (c = 1.0, DCM); ^{1}H NMR (400 MHz, $CDCl_3$) δ 7.90-7.87 (m, 1H), 7.83-7.81 (m, 1H), 7.61 (s, 1H), 7.46 (d, J = 8.0 Hz, 1H), 7.35-7.32 (m, 2H), 6.80-6.78 (m, 1H), 6.66 (s, 1H), 5.01 (t, J = 3.6 Hz, 1H), 4.06 (q, J = 7.2 Hz, 2H), 1.56-1.51 (m, 2H), 1.44 (t, J = 7.2 Hz, 3H), 1.28-1.22 (m, 2H), 1.01 (t, J = 7.2 Hz, 3H); ^{13}C NMR (100 MHz, $CDCl_3$) δ 164.7, 152.5 (d, J = 2.0 Hz), 144.2, 141.0, 137.9, 135.7, 133.7, 124.5, 124.1, 123.7, 122.3, 121.6 (q, J = 256 Hz), 120.9, 112.4, 103.6, 64.1, 18.3, 17.8, 15.4, 14.7; ^{19}F NMR (376 MHz, $CDCl_3$) δ -57.5; ^{29}Si NMR (80 MHz, $CDCl_3$) δ -21.2. HRMS-EI exact mass calcd. for $C_{20}H_{21}O_2F_3SSi^+$ ($[M]^+$) requires m/z 410.0978, found m/z 410.0983.

The enantiomeric excess was determined by HPLC on Chiralcel OD-H column (hexane : isopropanol = 100 : 0, flowing rate = 0.4 mL/min, 35 °C, UV detection at λ = 272 nm) t_{R1} = 21.2 min (major), t_{R2} = 23.0 min (minor).

(3-methoxy-[1,1'-biphenyl]-4-yl)(5-methylthiophen-2-yl)(propyl)silane (**3ra**)

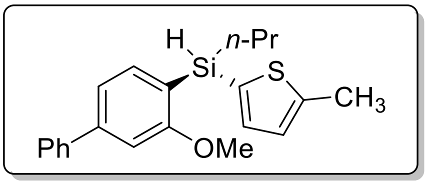

Colorless oil; isolated yield 58%, 88% ee; $[\alpha]_D^{22}$ = -36.0 (c = 1.0, DCM); ^{1}H NMR (400 MHz, $CDCl_3$) δ 7.60-7.58 (m, 2H), 7.49-7.42 (m, 3H), 7.38-7.34 (m, 1H), 7.20 (d, J = 3.2 Hz, 1H), 7.16 (dd, J_1 = 7.2 Hz, J_2 = 1.6 Hz, 1H), 7.04 (d, J = 1.6 Hz, 1H), 6.85 (dd, J_1 = 3.2 Hz, J_2 =1.2 Hz, 1H), 4.94 (t, J = 3.6 Hz, 1H), 3.91 (s, 3H), 2.53 (s, 3H), 1.54-1.49 (m, 2H), 1.9-1.14 (m, 2H), 1.00 (t, J = 7.2 Hz, 3H); ^{13}C NMR (100 MHz, $CDCl_3$) δ 164.7, 146.5, 145.1, 141.4, 137.2, 137.0, 131.7, 128.9, 127.7, 127.4, 126.9, 122.0, 119.8, 108.7, 55.4, 18.4, 17.9, 15.9, 15.3; ^{29}Si NMR (80 MHz, $CDCl_3$) δ -22.7. HRMS-EI exact mass calcd. for $C_{21}H_{24}OSSi^+$ ($[M]^+$) requires m/z 352.1312, found m/z 352.1313.

The enantiomeric excess was determined by HPLC on Chiralcel OD-H column (hexane : isopropanol = 100 : 0, flowing rate = 1.0 mL/min, 35 °C, UV detection at λ = 246 nm) t_{R1} = 15.1 min (minor), t_{R2} = 20.5 min (major).

(3-methoxy-[1,1'-biphenyl]-4-yl)(4-methylthiophen-2-yl)(propyl)silane (**3ri**)

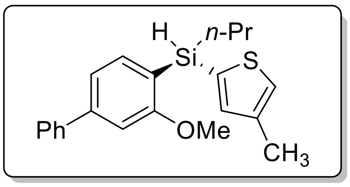

Colorless oil; isolated yield 54%, 91% ee; $[\alpha]_D^{22}$ = -32.0 (*c* = 1.0, DCM); ^{1}H NMR (400 MHz, $CDCl_3$) δ 7.60-7.58 (m, 2H), 7.49-7.42 (m, 3H), 7.38-7.34 (m, 1H), 7.19-7.16 (m, 3H), 7.04 (d, *J* = 1.6 Hz, 1H), 4.95 (t, *J* = 3.6 Hz, 1H), 3.91 (s, 3H), 2.29 (s, 3H), 1.55-1.49 (m, 2H), 1.21-1.16 (m, 2H), 1.00 (t, *J* = 7.2 Hz, 3H); ^{13}C NMR (100 MHz, $CDCl_3$) δ 164.7, 145.1, 141.4, 139.1, 139.0, 137.3, 133.9, 128.9, 127.7, 127.4, 127.3, 121.8, 119.8, 108.7, 55.4, 18.4, 17.9, 15.9, 15.2; ^{29}Si NMR (80 MHz, $CDCl_3$) δ -22.5. HRMS-EI exact mass calcd. for $C_{21}H_{24}OSSi^+$ ($[M]^+$) requires m/z 352.1312, found m/z 352.1313.

The enantiomeric excess was determined by HPLC on Chiralcel OD3-H column (hexane : isopropanol = 100 : 0, flowing rate = 1.0 mL/min, 23 °C, UV detection at λ = 235 nm) t_{R1} = 18.8 min (minor), t_{R2} = 25.9 min (major).

benzo[b]thiophen-2-yl(3-methoxy-[1,1'-biphenyl]-4-yl)(propyl)silane (**3rn**)

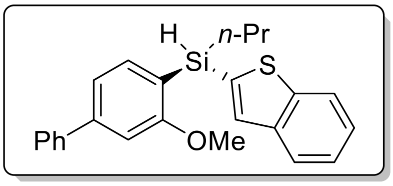

Colorless oil; isolated yield 60%, 91% ee; $[\alpha]_D^{22}$ = -108 (*c* = 1.0, DCM); ^{1}H NMR (400 MHz, $CDCl_3$) δ 7.91-7.88 (m, 1H), 7.84-7.82 (m, 1H), 7.64 (s, 1H), 7.60 (dd, J_1 = 8.4 Hz, J_2 = 1.2 Hz, 2H), 7.54 (d, *J* = 7.6 Hz, 1H), 7.39-7.32 (m, 2H), 7.39 -7.32 (m, 3H), 7.18 (dd, J_1 = 7.6 Hz, J_2 = 1.6 Hz, 1H), 7.07 (d, *J* = 1.2 Hz, 1H), 5.06 (t, *J* = 4.0 Hz, 1H), 3.93 (s, 3H), 1.58-1.54 (m, 2H), 1.29-1.25 (m, 2H), 1.03 (t, *J* = 7.2 Hz, 3H); ^{13}C NMR (100 MHz, $CDCl_3$) δ 164.8, 145.4, 144.3, 141.3, 141.1, 137.4, 136.4, 133.6, 128.9, 127.8, 127.4, 124.4, 124.0, 123.7, 122.2, 121.1, 119.9, 108.7, 55.4, 18.4, 17.9, 15.6; ^{29}Si NMR (80 MHz, $CDCl_3$) δ -21.2. HRMS-EI exact mass calcd. for $C_{24}H_{24}OSSi^+$ ($[M]^+$) requires m/z 388.1312, found m/z 388.1310.

The enantiomeric excess was determined by HPLC on Chiralcel OD-H column (hexane : isopropanol = 100 : 0, flowing rate = 1.0 mL/min, 35 °C, UV detection at λ = 268 nm) t_{R1} = 29.8 min (minor), t_{R2} = 34.9 min (major).

(1-ethoxynaphthalen-2-yl)(5-methylthiophen-2-yl)(propyl)silane (**3sa**)

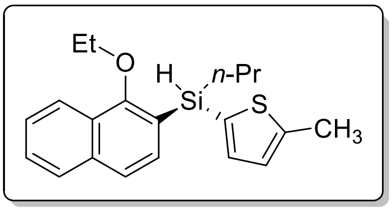

Colorless oil; isolated yield 63%, 94% ee; $[\alpha]_D^{22}$ = -132 (*c* = 1.0, DCM); ^{1}H NMR (400 MHz, $CDCl_3$) δ 8.11-8.07 (m, 1H), 7.85-7.81 (m, 1H), 7.59 (d, *J* = 8.0 Hz, 1H), 7.54-7.41 (m, 3H), 7.19 (d, *J* = 3.2 Hz, 1H), 6.86-6.85 (m, 1H), 5.14 (t, *J* = 3.6 Hz, 1H), 4.13-4.01 (m, 2H), 2.53 (s, 3H), 1.60-1.49 (m, 6H), 1.25-1.20 (m, 2H), 1.01 (t, *J* = 7.2 Hz, 3H); ^{13}C NMR (100 MHz, $CDCl_3$) δ 161.8, 146.8, 137.1, 136.5, 131.7, 131.5, 128.2, 127.7, 127.1, 126.9, 125.9, 123.8, 122.8, 122.6, 71.5, 18.4, 18.0, 15.8, 15.8, 15.3; ^{29}Si NMR (80 MHz, $CDCl_3$) δ -26.7. HRMS-EI exact mass calcd. for $C_{20}H_{24}OSSi^+$ ($[M]^+$) requires m/z 340.1312, found m/z 340.1316.

The enantiomeric excess was determined by HPLC on Chiralcel OD-H column (hexane : isopropanol = 100 : 0, flowing rate = 0.5 mL/min, 23 °C, UV detection at λ = 257 nm) t_{R1} = 31.1 min (major), t_{R2} = 33.6 min (minor).

(1-ethoxynaphthalen-2-yl)(4-methylthiophen-2-yl)(propyl)silane (3si)

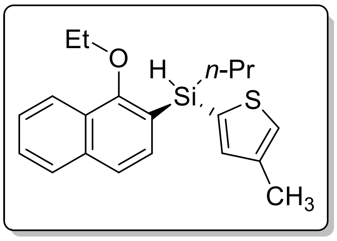

Colorless oil; isolated yield 66%, 94% ee; $[\alpha]_D^{22}$ = -164 (*c* = 1.0, DCM); ^{1}H NMR (400 MHz, $CDCl_3$) δ 8.13-8.05 (m, 1H), 7.89-7.79 (m, 1H), 7.59 (d, *J* = 8.0 Hz, 1H), 7.55-7.44 (m, 3H), 7.20 (d, *J* = 12.8 Hz, 2H), 5.15 (t, *J* = 3.6 Hz, 1H), 4.13-4.00 (m, 2H), 2.29 (s, 3H), 1.62 -1.49 (m, 4H), 1.26-1.15 (m, 3H), 1.01 (t, *J* = 7.2 Hz, 3H); ^{13}C NMR (100 MHz, $CDCl_3$) δ 161.8, 139.3, 139.1, 136.5, 133.7, 131.6, 128.2, 127.7, 127.4, 127.0, 126.0, 123.9, 122.7, 122.6, 71.5, 18.4, 17.9, 15.8, 15.8, 15.2; ^{29}Si NMR (80 MHz, $CDCl_3$) δ -26.4. HRMS-EI exact mass calcd. for $C_{20}H_{24}OSSi^+$ ($[M]^+$) requires m/z 340.1312, found m/z 340.1317.

The enantiomeric excess was determined by HPLC on Chiralcel OJ-H column (hexane : isopropanol = 100 : 0, flowing rate = 0.2 mL/min, 23 °C, UV detection at λ = 242 nm) t_{R1} = 43.0 min (minor), t_{R2} = 50.6 min (major).

benzo[b]thiophen-2-yl(1-ethoxynaphthalen-2-yl)(propyl)silane (3sn)

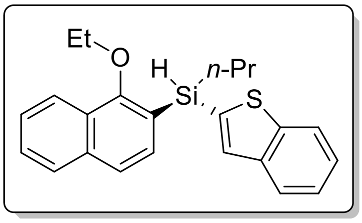

Colorless oil; isolated yield 66%, 89% ee; $[\alpha]_D^{22}$ = -90.0 (*c* = 1.0, DCM); ^{1}H NMR (400 MHz, $CDCl_3$) δ 8.12-8.09 (m, 1H), 7.90-7.81 (m, 3H), 7.63-7.60 (m, 2H), 7.53-7.50 (m, 3H), 7.37-7.31 (m, 2H), 5.26 (t, *J* = 3.6 Hz, 1H), 4.11 (d, *J* = 7.2 Hz, 2H), 1.63-1.58 (m, 2H), 1.54 (t, *J* = 7.2 Hz, 3H), 1.36-1.31 (m, 2H), 1.05 (t, *J* = 7.2 Hz, 3H); ^{13}C NMR (100 MHz, $CDCl_3$) δ 162.0, 144.3, 141.1, 136.7, 136.2, 133.7, 131.6, 128.3, 127.7, 127.1, 126.1, 124.5, 124.2, 124.0, 123.7, 122.6, 122.3, 121.9, 71.6, 18.4, 17.9, 15.8, 15.5; ^{29}Si NMR (80 MHz, $CDCl_3$) δ -25.2. HRMS-EI exact mass calcd. for $C_{23}H_{24}OSSi^+$ ($[M]^+$) requires m/z 376.1312, found m/z 376.1316.

The enantiomeric excess was determined by HPLC on Chiralcel OD-H column (hexane : isopropanol = 99.9 : 0.1, flowing rate = 1.0 mL/min, 35 °C, UV detection at λ = 224 nm) t_{R1} = 16.0 min (major), t_{R2} = 19.3 min (minor).

(5-methylthiophen-2-yl)(naphthalen-1-yl)(propyl)silane (**3ta**)

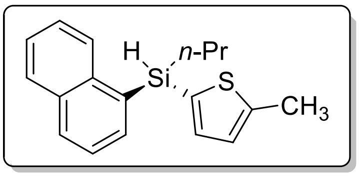

Colorless oil; isolated yield 70%, 92% ee; $[\alpha]_D^{22}$ = +42.0 (*c* = 1.0, DCM); ^{1}H NMR (400 MHz, $CDCl_3$) δ 8.15-8.12 (m, 1H), 7.92-7.86 (m, 2H), 7.80 (dd, J_1 = 6.8 Hz, J_2 = 1.2 Hz, 1H), 7.50-7.45 (m, 3H), 7.14 (d, *J* = 3.2 Hz, 1H), 6.83 (d, *J* = 3.2 Hz, 1H), 5.36 (t, *J* = 3.6 Hz, 1H), 2.52 (s, 3H), 1.60-1.52 (m, 2H), 1.33-1.27 (m, 2H), 1.02 (t, *J* = 7.2 Hz, 3H); ^{13}C NMR (100 MHz, $CDCl_3$) δ 147.0, 137.3, 137.2, 135.5, 133.4, 132.9, 131.2, 130.7, 129.0, 128.1, 127.2, 126.2, 125.8, 125.3, 18.4, 17.9, 16.2, 15.3; ^{29}Si NMR (80 MHz, $CDCl_3$) δ -23.8. HRMS-EI exact mass calcd. for $C_{18}H_{20}SSi^+$ ($[M]^+$) requires m/z 296.1050, found m/z 296.1053.

The enantiomeric excess was determined by HPLC on Chiralcel OD-H column (hexane : isopropanol = 100 : 0, flowing rate = 0.5 mL/min, 35 °C, UV detection at λ = 225 nm) t_{R1} = 21.8 min (minor), t_{R2} = 27.5 min (major).

(4-methylthiophen-2-yl)(naphthalen-1-yl)(propyl)silane (**3ti**)

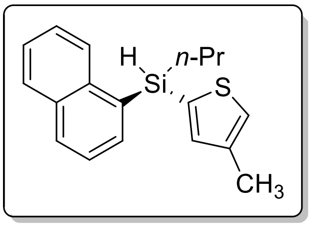

Colorless oil; isolated yield 56%, 96% ee; $[\alpha]_D^{22}$ = +138 (*c* = 1.0, DCM); ^{1}H NMR (400 MHz, $CDCl_3$) δ 8.14-8.11 (m, 1H), 7.93-7.86 (m, 2H), 7.80 (dd, J_1 = 6.8 Hz, J_2 =1.2 Hz, 1H), 7.50- 7.45 (m, 3H), 7.20 (s, 1H), 7.12 (s, 1H), 5.36 (t, *J* = 3.6 Hz, 1H), 2.26 (s, 3H), 1.59-1.53 (m, 2H), 1.33-1.28 (m, 2H), 1.01 (t, *J* = 7.2 Hz, 3H); ^{13}C NMR (100 MHz, $CDCl_3$) δ 139.4, 139.3, 137.2, 135.6, 133.4, 133.4, 132.7, 130.7, 129.0, 128.1, 127.6, 126.2, 125.8, 125.3, 18.4, 17.9, 16.2, 15.2; ^{29}Si NMR (80 MHz, $CDCl_3$) δ -23.6. HRMS-EI exact mass calcd. for $C_{18}H_{20}SSi^+$ ($[M]^+$) requires m/z 296.1050, found m/z 296.1052.

The enantiomeric excess was determined by HPLC on Chiralcel OD-H column (hexane : isopropanol = 100 : 0, flowing rate = 0.5 mL/min, 23 ºC, UV detection at λ = 224 nm) t_{R1} = 23.3 min (minor), t_{R2} = 26.9 min (major).

benzo[b]thiophen-2-yl(naphthalen-1-yl)(propyl)silane (**3tn**)

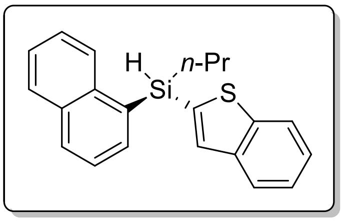

Colorless oil; isolated yield 68%, 96% ee; $[\alpha]_D^{22}$ = -156 (*c* = 1.0, DCM); ^{1}H NMR (400 MHz, $CDCl_3$) δ 8.17-8.14 (m, 1H), 7.96 (d, *J* = 8.0 Hz, 1H), 7.91-7.86 (m, 3H), 7.80-7.78 (m, 1H), 7.57 (s, 1H), 7.52-7.45 (m, 3H), 7.38-7.32 (m, 2H), 5.48 (t, *J* = 3.6 Hz, 1H), 1.69-1.60 (m, 2H), 1.44-1.39 (m, 2H), 1.06 (t, *J* = 7.2 Hz, 3H); ^{13}C NMR (100 MHz, $CDCl_3$) δ 144.3, 141.1, 137.2, 135.9, 135.7, 134.0, 133.4, 131.9, 131.0, 129.1, 128.0, 126.4, 125.9, 125.4, 124.6, 124.2, 123.8, 122.3, 18.5, 17.9, 15.9; ^{29}Si NMR (80 MHz, $CDCl_3$) δ -22.5. HRMS-EI exact mass calcd. for $C_{21}H_{20}SSi^+$ ($[M]^+$) requires m/z 332.1050, found m/z 332.1052.

The enantiomeric excess was determined by HPLC on Chiralcel OD3-H column (hexane : isopropanol = 100 : 0, flowing rate = 1.0 mL/min, 35 ºC, UV detection at λ = 239 nm) t_{R1} = 15.9 min (minor), t_{R2} = 19.1 min (major).

10-(5-methylthiophen-2-yl)-10-propyl-10H-benzo[4,5]silolo[3,2-b]benzofuran (**3va**)

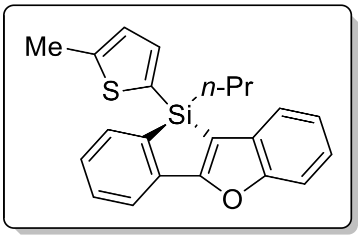

Colorless oil; isolated yield 86%, 31% ee; $[\alpha]_D^{22}$ = +29 (c = 1.0, DCM); ^{1}H NMR (400 MHz, $CDCl_3$) δ 7.72-7.59 (m, 4H), 7.46 (t, J = 7.4 Hz, 1H), 7.34-7.25 (m, 4H), 6.86 (s, 1H), 2.53 (s, 3H), 1.68-1.61 (m, 2H), 1.39-1.25 (m, 2H), 1.02 (t, J = 7.2 Hz, 3H); ^{13}C NMR (100 MHz, $CDCl_3$) δ 170.4, 158.7, 147.4, 140.0, 139.1, 137.2, 133.5, 130.7, 130.4, 129.2, 128.2, 127.3, 124.1, 123.6, 122.2, 119.8, 111.9, 111.2, 18.1, 17.9, 15.9, 15.3; ^{29}Si NMR (80 MHz, $CDCl_3$) δ -15.5. HRMS-APCI exact mass calcd. for $C_{22}H_{21}OSSi^+$ ($[M+H]^+$) requires m/z 361.1077, found m/z 350.1080.

The enantiomeric excess was determined by HPLC on Chiralcel OD-H column (hexane : isopropanol = 99.8 : 0.2, flowing rate = 1.0 mL/min, 35 °C, UV detection at λ = 327 nm) t_{R1} = 13.3 min (minor), t_{R2} = 15.8 min (major).

10-(4-methylthiophen-2-yl)-10-propyl-10H-benzo[4,5]silolo[3,2-b]benzofuran (**3vi**)

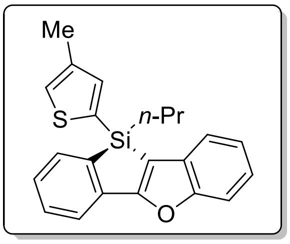

Colorless oil; isolated yield 87%, 56% ee; $[\alpha]_D^{22}$ = +36.4 (c = 1.0, DCM); ^{1}H NMR (400 MHz, $CDCl_3$) δ 7.72-7.60 (m, 4H), 7.47 (t, J = 7.5 Hz, 1H), 7.35-7.29 (m, 3H), 7.22 (d, J = 4.3 Hz, 2H), 2.28 (s, 3H), 1.68-1.61 (m, 2H), 1.42-1.28 (m, 2H), 1.02 (t, J = 7.2 Hz, 3H); ^{13}C NMR (100 MHz, $CDCl_3$) δ 170.5, 158.7, 139.9, 139.5, 139.1, 139.1, 133.5, 131.4, 130.7, 130.4, 128.2, 128.0, 124.1, 123.6, 122.2, 119.8, 111.9, 111.1, 18.1, 17.9, 15.9, 15.2; ^{29}Si NMR (80 MHz, $CDCl_3$) δ -15.2. HRMS-APCI exact mass calcd. for $C_{22}H_{21}OSSi^+$ ($[M+H]^+$) requires m/z 361.1077, found m/z 361.1082.

The enantiomeric excess was determined by HPLC on Chiralcel OD-H column (hexane : isopropanol = 99.7 : 0.3, flowing rate = 0.6 mL/min, 35 °C, UV detection at λ = 331 nm) t_{R1} = 12.2 min (minor), t_{R2} = 13.0 min (major).

10-(benzo[b]thiophen-2-yl)-10-propyl-10H-benzo[4,5]silolo[3,2-b]benzofuran (3vn)

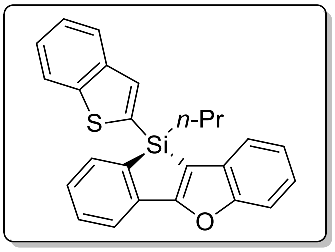

Colorless oil; isolated yield 87%, 46% ee; $[\alpha]_D^{22}$ = +68.2 (*c* = 1.0, DCM); ^{1}H NMR (400 MHz, $CDCl_3$) δ 7.87-7.85 (m, 1H), 7.880-7.71 (m, 1H), 7.72 (t, *J* = 7.7 Hz, 2H), 7.65 (s, 2H), 7.61 (d, *J* = 8.0 Hz, 1H), 7.48 (t, *J* = 7.6 Hz, 1H), 7.35-7.30 (m, 5H), 1.71-1.65 (m, 2H), 1.49-1.43 (m, 1H), 1.39-1.33 (m, 1H), 1.04 (t, *J* = 7.5 Hz, 3H); ^{13}C NMR (100 MHz, $CDCl_3$) δ 170.7, 158.8, 144.2, 140.9, 139.3, 139.2, 133.9, 133.7, 133.7, 130.6, 130.6, 128.3, 124.9, 124.3, 124.3, 123.9, 123.8, 122.3, 122.2, 120.0, 112.0, 110.7, 18.1, 17.9, 15.6; ^{29}Si NMR (80 MHz, $CDCl_3$) δ -14.9. HRMS-APCI exact mass calcd. for $C_{25}H_{21}OSSi^+$ ($[M]^+$) requires m/z 397.1077, found m/z 397.1079.

The enantiomeric excess was determined by HPLC on Chiralcel OD-H column (hexane : isopropanol = 99.5 : 0.5, flowing rate = 1.0 mL/min, 35 °C, UV detection at λ = 336 nm) t_{R1} = 10.9 min (minor), t_{R2} = 14.0 min (major).

2-methyl-4-(5-methylthiophen-2-yl)-4-propyl-4H-benzo[4,5]silolo[3,2-b]thiophene (**3wa**)

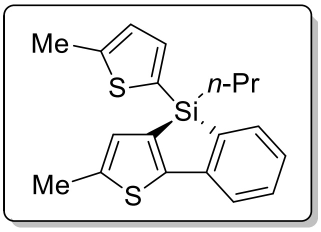

Colorless oil; isolated yield 82%, 75% ee; $[\alpha]_D^{22}$ = +5.8 (*c* = 1.0, DCM); ^{1}H NMR (400 MHz, $CDCl_3$) δ 7.55 (d, *J* = 7.0 Hz, 1H), 7.35 (d, *J* = 7.4 Hz, 2H), 7.17 (d, *J* = 6.5 Hz, 1H), 6.83 (s, 1H), 2.54 (s, 3H), 2.50 (s, 3H), 1.57-1.50 (m, 2H), 1.23-1.17 (m, 2H), 0.97 (t, *J* = 7.2 Hz, 3H); ^{13}C NMR (100 MHz, $CDCl_3$) δ 155.7, 147.1, 144.5, 142.3, 138.8, 137.6, 136.9, 133.2, 130.5, 130.1, 127.7, 127.2, 126.3, 120.5, 18.1, 17.8, 15.9, 15.7, 15.3; ^{29}Si NMR (80 MHz, $CDCl_3$) δ -15.5. HRMS-EI exact mass calcd. for $C_{19}H_{20}S_2Si^+$ ($[M]^+$) requires m/z 340.0770, found m/z 340.0767.

The enantiomeric excess was determined by HPLC on Chiralcel OD-H column (hexane : isopropanol = 99 : 1, flowing rate = 1.0 mL/min, 35 °C, UV detection at λ = 233 nm) t_{R1} = 7.9 min (major), t_{R2} = 15.1 min (minor).

2-methyl-4-(4-methylthiophen-2-yl)-4-propyl-4H-benzo[4,5]silolo[3,2-b]thiophene (**3wi**)

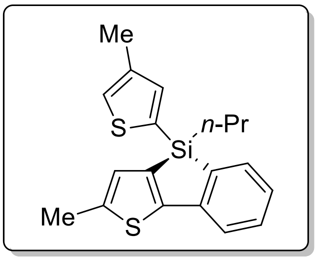

Colorless oil; isolated yield 84%, 61% ee; $[\alpha]_D^{22}$ = +19.6 (*c* = 1.0, DCM); ^{1}H NMR (400 MHz, $CDCl_3$) δ 7.56 (d, *J* = 7.1 Hz, 1H), 7.35 (d, *J* = 7.1 Hz, 2H), 7.17 (d, *J* = 6.7 Hz, 1H), 7.12 (s, 1H), 6.83 (s, 1H), 2.55 (s, 3H), 2.25 (s, 3H), 1.57-1.49 (m, 2H), 1.24-1.18 (m, 2H), 0.97 (t, *J* = 7.2 Hz, 3H); ^{13}C NMR (100 MHz, $CDCl_3$) δ 155.8, 144.6, 142.4, 139.4, 138.9, 138.6, 137.5, 133.2, 132.3, 130.6, 127.7, 127.7, 126.4, 120.5, 18.1, 17.8, 15.9, 15.7, 15.2; ^{29}Si NMR (80 MHz, $CDCl_3$) δ -15.5. HRMS-EI exact mass calcd. for $C_{19}H_{20}S_2Si^+$ ($[M]^+$) requires m/z 340.0770, found m/z 340.0766.

The enantiomeric excess was determined by HPLC on Chiralcel OD-H column (hexane : isopropanol = 99 : 1, flowing rate = 1.0 mL/min, 35 °C, UV detection at λ = 317 nm) t_{R1} = 8.3 min (major), t_{R2} = 10.5 min (minor).

4-(benzo[b]thiophen-2-yl)-2-methyl-4-propyl-4H-benzo[4,5]silolo[3,2-b]thiophene (**3wn**)

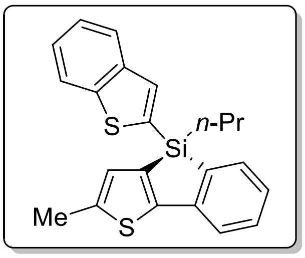

Colorless oil; isolated yield 85%, 86% ee; $[\alpha]_D^{22}$ = +22.8 (*c* = 1.0, DCM); ^{1}H NMR (400 MHz, $CDCl_3$) δ 7.85 (d, *J* = 6.0 Hz, 1H), 7.78 (d, *J* = 7.8 Hz, 1H), 7.62 (d, *J* = 7.1 Hz, 1H), 7.58 (s, 1H), 7.38 (d, *J* = 8.7 Hz, 2H), 7.35-7.31 (m, 2H), 7.20 (t, *J* = 7.0 Hz, 1H), 6.89 (s, 1H), 2.57 (s, 3H), 1.66-1.56 (m, 2H), 1.36-1.26 (m, 2H), 1.01 (t, *J* = 7.5 Hz, 3H); ^{13}C NMR (100 MHz, $CDCl_3$) δ 156.1, 144.6, 144.2, 142.6, 141.0, 138.0, 136.9, 134.8, 133.5, 133.3, 130.8, 127.6, 126.5, 124.7, 124.2, 123.8, 122.3, 120.7, 18.1, 17.8, 15.8, 15.6; ^{29}Si NMR (80 MHz, $CDCl_3$) δ -14.3. HRMS-EI exact mass calcd. for $C_{22}H_{20}S_2Si^+$ ($[M]^+$) requires m/z 376.0770, found m/z 376.0766.

The enantiomeric excess was determined by HPLC on Chiralcel OD-H column (hexane : isopropanol = 98 : 2, flowing rate = 1.0 mL/min, 35 °C, UV detection at λ = 336 nm) t_{R1} = 7.0 min (major), t_{R2} = 21.4 min (minor).

2.4 Synthetic Transformation and Control Experiments

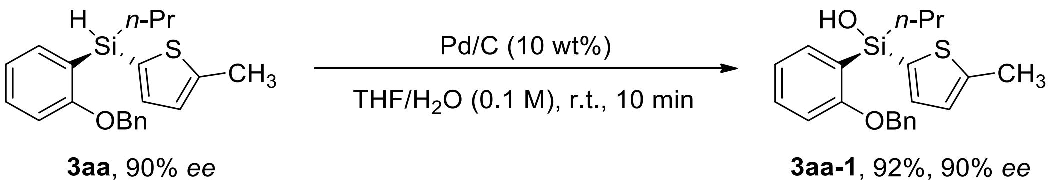

To a 10 mL reaction vial equipped with magnetic stirring bar were added monohydrosilane **3aa** (106 mg, 0.3 mmol, 90% ee), Pd/C (10.6 mg, 10 wt%) and THF (containing 1% v/v H_2O) under argon atmosphere. The reaction mixture was stirred at room temperature for 10 minutes and the reaction progress was monitored by TLC. After the starting material was fully consumed, the resulting mixture was diluted with DCM (5.0 mL), and filtered through a thin pad of celite. The solution was dried over anhydrous Na_2SO_4, filtered, concentrated in vacuo, and purified by column chromatography (95:5 hexanes/EtOAc) to afford silanol **3aa-1** in 92% yield (102 mg, 90% ee).

(2-(benzyloxy)phenyl)(5-methylthiophen-2-yl)(propyl)silanol (3aa-1)

Colorless oil; isolated yield 92%, 90% ee; $[\alpha]_D^{18}$ = -19.4 (c = 1.0, DCM); ^{1}H NMR (400 MHz, $CDCl_3$) δ 7.47 (dd, J = 7.1, 1.8 Hz, 1H), 7.41-7.31 (m, 6H), 7.14 (d, J = 3.3 Hz, 1H), 7.01 (t, J = 7.3 Hz, 1H), 6.94 (d, J = 8.3 Hz, 1H), 6.84 (d, J = 3.3 Hz, 1H), 5.08 (s, 2H), 2.80 (s, 1H), 2.53 (s, 3H), 1.57-1.47 (m, 2H), 1.15-1.11 (m, 2H), 0.98 (t, J = 7.3 Hz, 3H); ^{13}C NMR (100 MHz, $CDCl_3$) δ 163.3, 146.3, 136.6, 136.2, 135.7, 134.6, 131.8, 128.8, 128.2, 127.7, 126.8, 124.7, 121.2, 110.9, 70.3, 18.9, 18.2, 16.8, 15.2; ^{29}Si NMR (80 MHz, $CDCl_3$) δ -6.8. HRMS-EI exact mass calcd. for $C_{21}H_{24}O_2SSi^+$ ($[M]^+$) requires m/z 368.1261, found m/z 368.1262.

The enantiomeric excess was determined by HPLC on Chiralcel OD-H column (hexane : isopropanol = 100 : 0, flowing rate = 1.0 mL/min, 18 °C, UV detection at λ = 262 nm) t_{R1} = 20.7 min (minor), t_{R2} = 25.0 min (major).

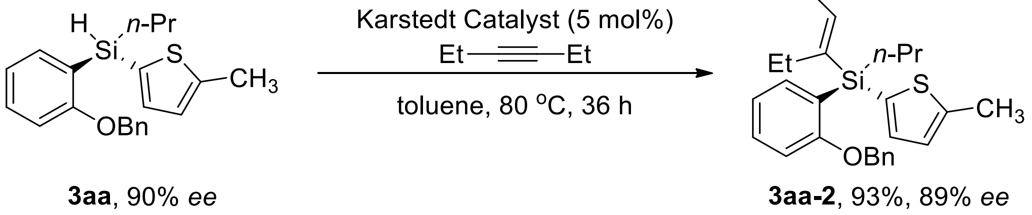

To a 10 mL reaction vial equipped with magnetic stirring bar were added

monohydrosilane **3aa** (106 mg, 0.3 mmol, 90% ee), 3-hexyne (24.6 mg, 0.3 mmol), Pt(dvds) (0.3 mL, 0.05 M, 0.015 mmol) and toluene (1.5 mL) in a N_2-flushed glovebox. After being stirred for 36 h at 80 °C, the reaction mixture was concentrated under reduced pressure and purified by silica gel column chromatography (hexane) to afford 122 mg of vinylsilane **3aa-2** (93%, 89% ee) as a colorless oil.

(E)-(2-(benzyloxy)phenyl)(hex-3-en-3-yl)(5-methylthiophen-2-yl)(propyl)silane (3aa-2)

Colorless oil; isolated yield 93%, 89% ee; $[\alpha]_D^{18}$ = -8.0 (*c* = 1.0, DCM); ^{1}H NMR (400 MHz, $CDCl_3$) δ 7.38-7.31 (m, 7H), 7.11 (d, *J* = 3.3 Hz, 1H), 6.94 (t, *J* = 7.3 Hz, 1H), 6.89 (d, *J* = 8.5 Hz, 1H), 6.86 (d, *J* = 3.4 Hz, 1H), 5.83 (t, *J* = 6.9 Hz, 1H), 5.05 (s, 2H), 2.55 (s, 3H), 2.18 (h, *J* = 7.4, 6.9 Hz, 4H), 1.49-1.35 (m, 2H), 1.24-1.16 (m, 1H), 1.13-1.05 (m, 1H), 0.97 (t, *J* = 7.5 Hz, 3H), 0.92 (t, *J* = 7.2 Hz, 3H), 0.82 (t, *J* = 7.5 Hz, 3H); ^{13}C NMR (100 MHz, $CDCl_3$) δ 163.6, 145.7, 145.4, 137.8, 137.6, 137.3, 136.9, 134.6, 131.2, 128.4, 127.7, 127.5, 126.7, 124.6, 120.7, 110.5, 69.8, 23.2, 22.0, 18.7, 18.1, 17.1, 15.3, 14.9, 14.2; ^{29}Si NMR (80 MHz, $CDCl_3$) δ -15.3. HRMS-EI exact mass calcd. for $C_{27}H_{34}OSSi^+$ ($[M]^+$) requires m/z 434.2094, found m/z 434.2098.

The enantiomeric excess was determined by HPLC on Chiralcel OD-H column (hexane : isopropanol = 100 : 0, flowing rate = 1.0 mL/min, 18 °C, UV detection at λ = 243 nm) t_{R1} = 13.5 min (major), t_{R2} = 14.7 min (minor).

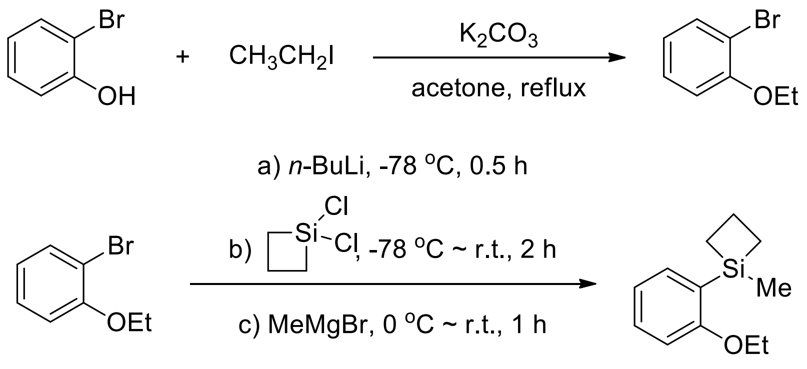

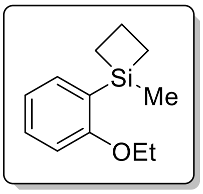

1-(2-ethoxyphenyl)-1-methylsiletane (**1u**)

Methyl substituted silacyclobutane **1u** was synthesized following similar procedure[3] in 74% yield as a colorless oil; ^{1}H NMR (500 MHz, $CDCl_3$) δ 7.47 (dd, J_1 = 7.5 Hz, J_2 = 2.5 Hz, 1H), 7.36 (td, J_1 = 7.5 Hz, J_2 = 2.5 Hz, 1H), 6.98 (t, *J* = 7.5 Hz, 1H), 6.82 (d, *J* = 5.0 Hz, 1H), 4.04 (q, *J* = 7.5

Hz, 2H), 2.17-2.11 (m, 2H), 1.40 (t, *J* = 7.5 Hz, 3H), 1.36-1.30 (m, 2H), 1.17-1.11 (m, 2H), 0.52 (s, 3H); ^{13}C NMR (125 MHz, $CDCl_3$) δ 163.6, 135.0, 131.3, 126.9, 120.5, 110.3, 63.4, 18.3, 14.9, 14.5, -0.6.

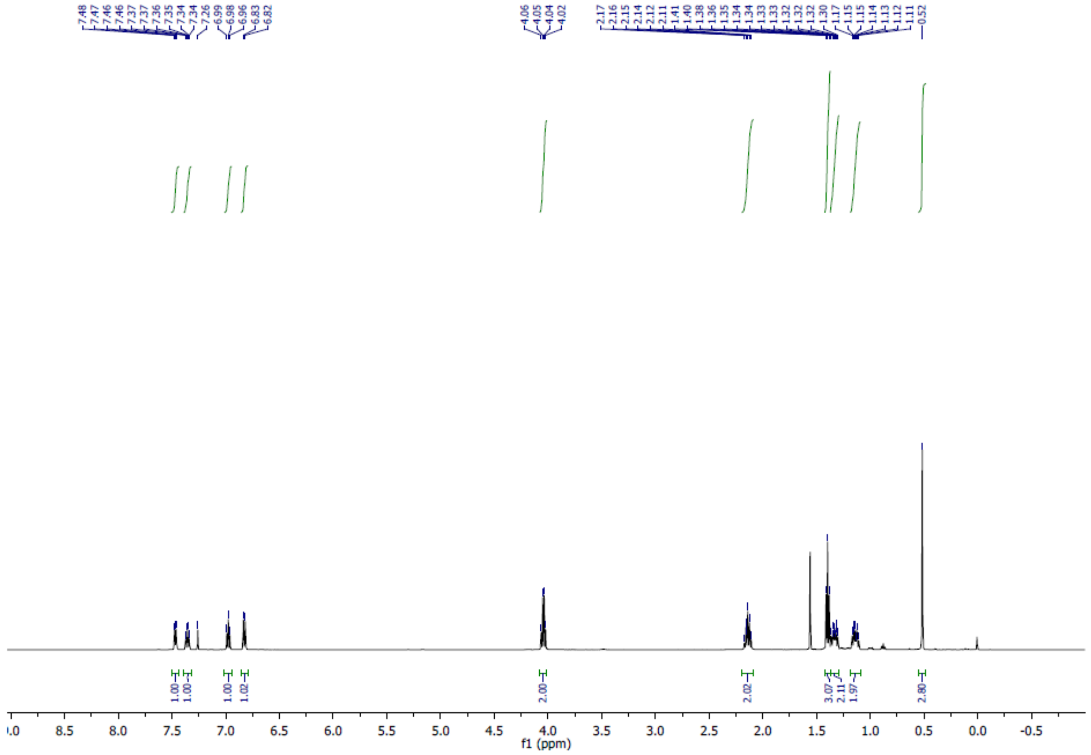

Supplementary Figure 9. ^{1}H NMR spectrum of **1u**

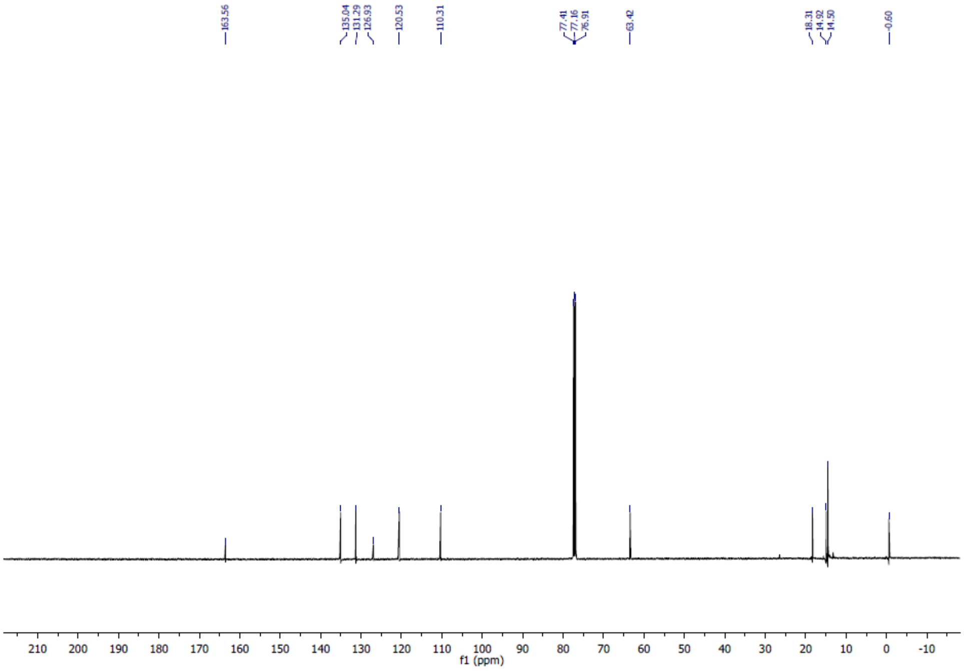

Supplementary Figure 10. ^{13}C NMR spectrum of **1u**

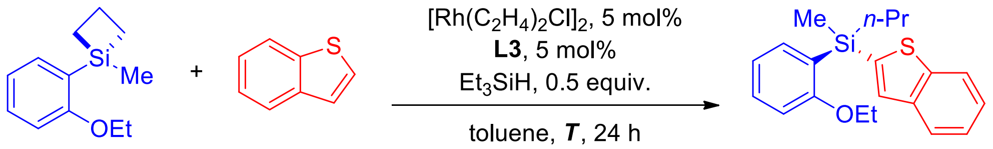

1u **2n** **3un**, ***T*** = r.t., no reaction

T = 80 °C, trace by GC-MS

In a N_2-flushed glovebox, $[Rh(C_2H_4)_2Cl]_2$ (7.8 mg, 0.02 mmol, 1.0 equiv.), DTBM-MeO-Biphep (23.0 mg, 0.02 mmol, 1.0 equiv.) and toluene (2.0 mL) were added into a sealed tube. The reaction mixture was stirred at room temperature for 30 minutes. Then, Et_3SiH (32 µL, 0.2 mmol, 10.0 equiv.) was added in one portion and the stirring continued for 4 hours at 50 °C to generate the corresponding [Rh]-H catalyst **CAT6**. To a sealed tube equipped with magnetic stirring bar were added 0.5 mL of the [Rh]-H solution, substrate **1u** (20.6 mg, 0.1 mmol, 1.0 equiv.), benzothiophene **2n** (13.4 mg, 0.1 mmol, 1.0 equiv.) and the total volume of toluene solution was adjusted to be 1.0 mL. The reaction mixture was stirred at indicated temperature for 24 hours and characterized by GC-MS analysis. No reaction took place under standard conditions with both starting materials recovered, which underscored the different reactivities of current intermolecular reaction with previous intramolecular reaction.[3]

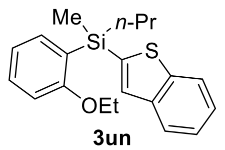

3un

M.W. = 340

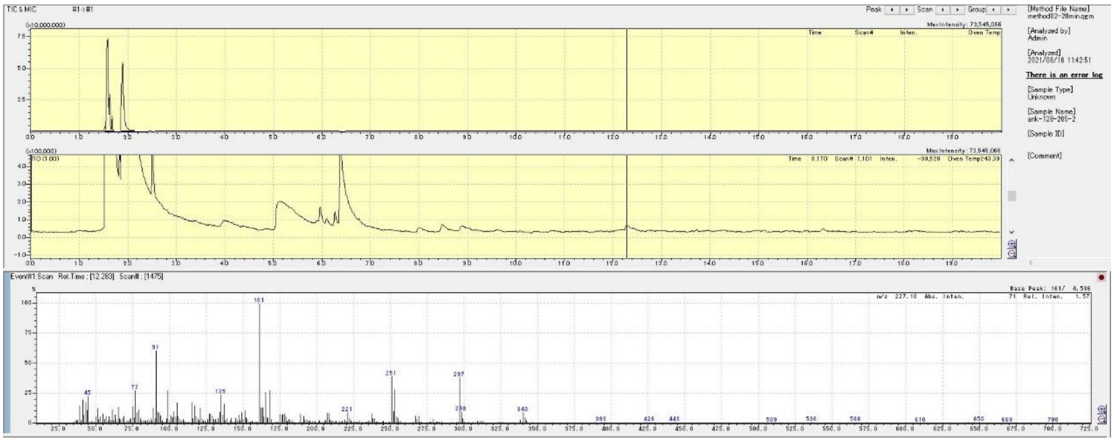

Supplementary Figure 11. Detection of **3un** by GC-MS analysis

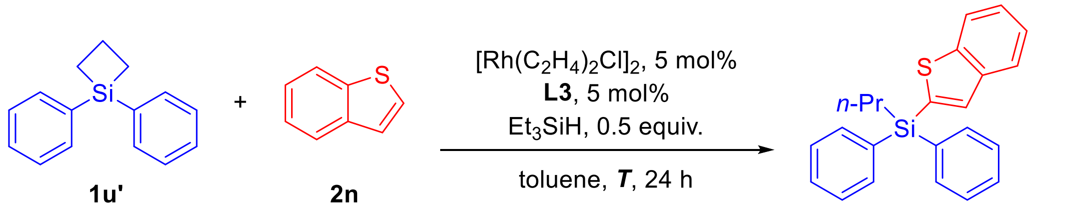

3un', ***T*** = r.t., trace by GC-MS
T = 80 °C, 9% by GC-MS

The reaction of 1,1-diphenylsilacyclobutane **1u'** with benzothiophene **2n** was conducted under the same conditions as described above.

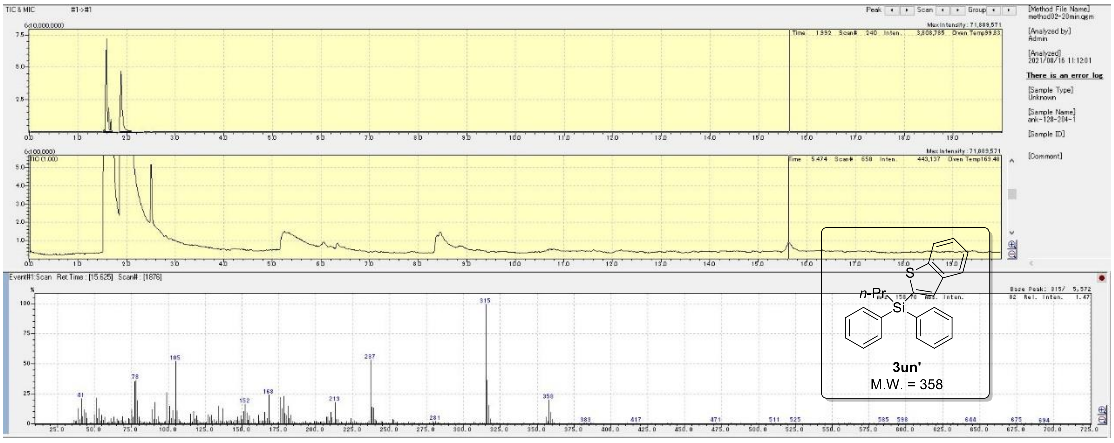

Supplementary Figure 12. Detection of **3un'** by GC-MS analysis

3. Supplementary Discussion

3.1 Determination of Absolute Configuration

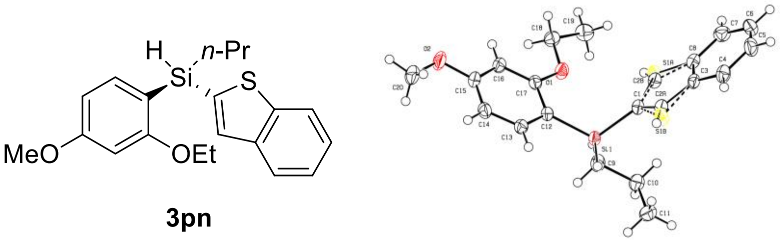

3pn

Supplementary Figure 13: Single crystal structure of **3pn**

Identification code	3pn
Empirical formula	$C_{20}H_{24}O_2SiS$
Formula weight	356.54
Temperature/K	180.00(10)
Crystal system	monoclinic
Space group	$P2_1$

a/Å	12.0691(3)
b/Å	5.94920(10)
c/Å	13.8135(4)
α/°	90
β/°	107.058(3)
γ/°	90
Volume/$Å^3$	948.20(4)
Z	2
$\rho_{calc}g/cm^3$	1.249
μ/mm^{-1}	0.243
F(000)	380.0
Crystal size/mm^3	0.42 × 0.09 × 0.05
Radiation	Mo Kα (λ = 0.71073)
2Θ range for data collection/°	5.326 to 54.962
Index ranges	-15 ≤ h ≤ 15, -7 ≤ k ≤ 7, -17 ≤ l ≤ 17
Reflections collected	20607
Independent reflections	4351 [R_{int} = 0.0357, R_{sigma} = 0.0273]
Data/restraints/parameters	4351/5/231
Goodness-of-fit on F^2	1.005
Final R indexes [I>=2σ (I)]	R_1 = 0.0304, wR_2 = 0.0811
Final R indexes [all data]	R_1 = 0.0331, wR_2 = 0.0826
Largest diff. peak/hole / e $Å^{-3}$	0.42/-0.15
Flack parameter	-0.01(4)

3.2 Identification of Catalyst Resting State

In a N_2-flushed glovebox, $[Rh(C_2H_4)_2Cl]_2$ (7.8 mg, 0.02 mmol, 1.0 equiv.), DTBM-segphos (23.6 mg, 0.02 mmol, 1.0 equiv.) and toluene-d_8 (2.0 mL) were added into a sealed tube. The reaction mixture was stirred at room temperature for 30 minutes. Then, Et_3SiH (32 µL, 0.2 mmol, 10.0 equiv.) was added in one portion and the stirring continued for 4 hours at 50 °C to generate the corresponding [Rh]-H catalyst **CAT4**. To a NMR tube equipped with J. Young valve were added 0.5 mL of the [Rh]-H solution and benzothiophene **2n** (13.4 mg, 0.1 mmol, 1.0 equiv.). The reaction mixture was subjected to NMR analysis, the ^{31}P NMR signal shifted to higher magnetic fields (35.5 ppm to 31.9 ppm) which was attributed to the coordination of benzothiophene to rhodium catalyst.[5] Then substrate **1c** (19.2 mg, 0.1 mmol, 1.0 equiv.) was added, the

reaction mixture was subjected to ^{1}H NMR analysis and monohydrosilane **3cn** could be detected after the first data acquisition.

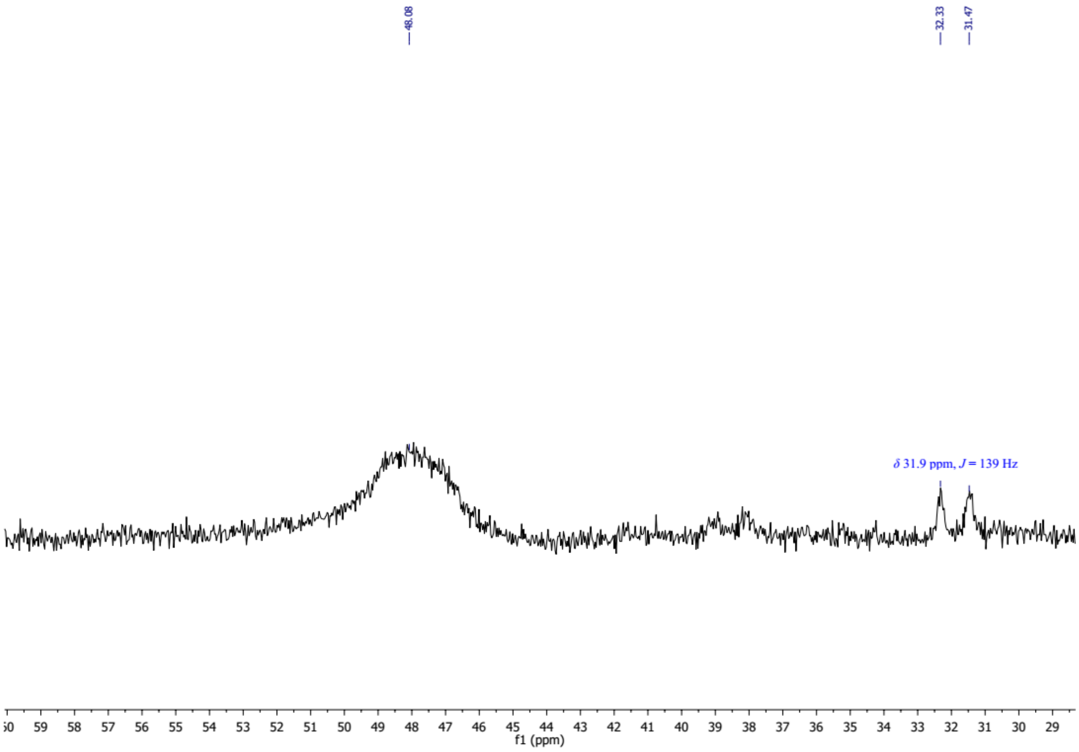

Supplementary Figure 14. ^{31}P NMR spectrum of benzothiophene coordinated Rh(**L4**)-H catalyst

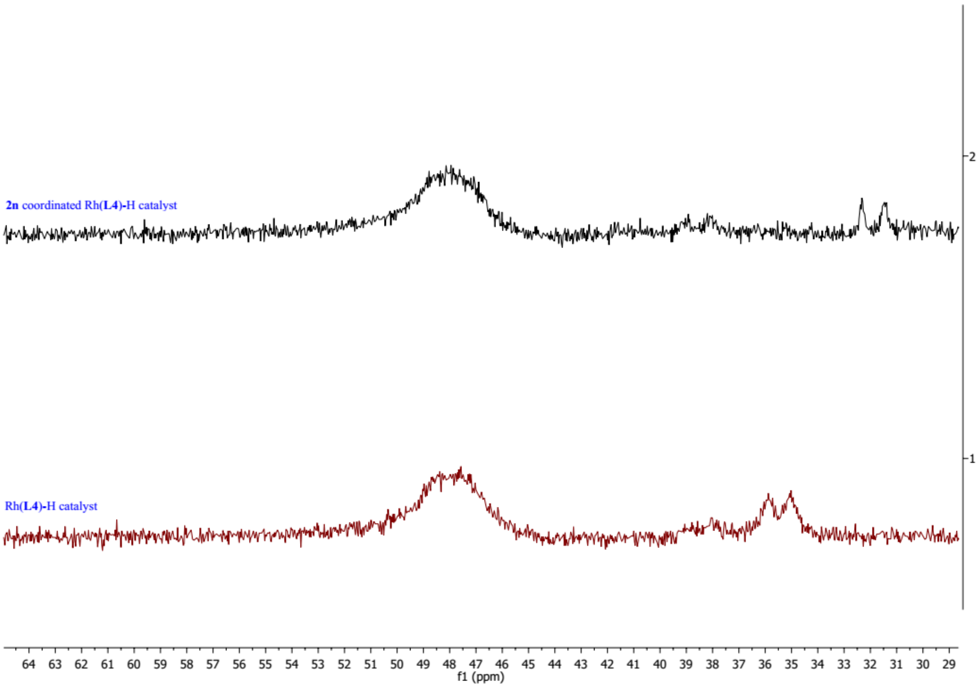

Supplementary Figure 15. ^{31}P NMR monitoring on the coordination of **2n** to

Rh(**L4**)-H catalyst

Interpretation: The thiophene coordinated rhodium hydride species was considered to be the catalyst resting state, as it might affect both enantioselectivity and reactivity of the oxidative addition process of SCB onto Rh^I. For thiophene partner **2f**, both sulfur atom and adjacent oxygen atom of carbonyl group would bond to the rhodium center, giving rise to bidentate coordinated complex. Oxidative addition of Si-C bond onto such rhodium complex might affect enantioselectivity as this process was considered to be enantio-determining based on our previous DFT calculations.[3] For thiophene partner **2l**, the carbonyl group was placed away from the coordinated rhodium center, therefore better enantioselectivity was observed. Besides, we also added 2.0 equiv. of pyridine into the pre-formed **CAT4** stock solution. The ^{31}P NMR signal remained unchanged as opposed to the addition of thiophene, indicating the coordination between soft acid (rhodium) and hard base (nitrogen) didn't match. This also help to explain why only thiophenes were successfully in this reaction, because the initial coordination of thiphenes to the rhodium center make the formal intermolecular silylation into the intramolecular one, thus overcoming the low reactivities of C-H bonds.

3.3 Deuterium Labeling Experiments

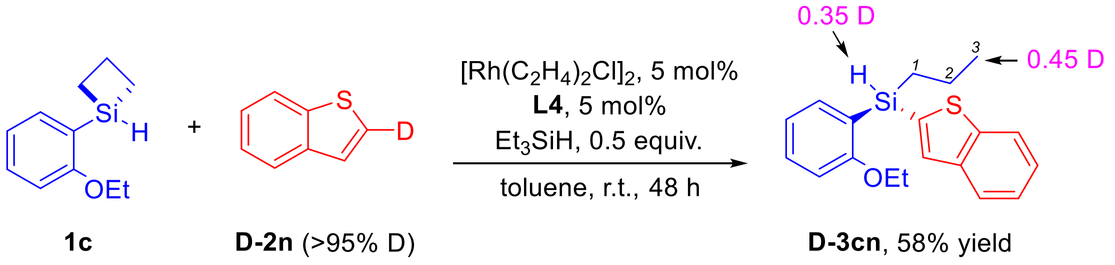

To a sealed tube equipped with magnetic stirring bar were added 0.5 mL of the Rh(**L4**)-H solution, substrate **1c** (19.2 mg, 0.1 mmol, 1.0 equiv.), deuterated benzothiophene **D-2n** (13.5 mg, 0.1 mmol, 1.0 equiv.) and the total volume of toluene solution was adjusted to be 1.0 mL. The reaction mixture was stirred at room temperature for 48 h and diluted with dichloromethane (2.0 mL). The organic layer was concentrated under reduced pressure and the residue was purified by preTLC to give **D-3cn** (58% yield) as a colorless oil. As depicted in Supplementary Figure 16, the deuterium incorporation of **D-3cn** was calculated by ^1H NMR analysis. Moreover, deuteration on the Si-H and

the terminal carbon atom of *n*-propyl group was further identified by ^{2}H NMR analysis (Supplementary Figure 17).

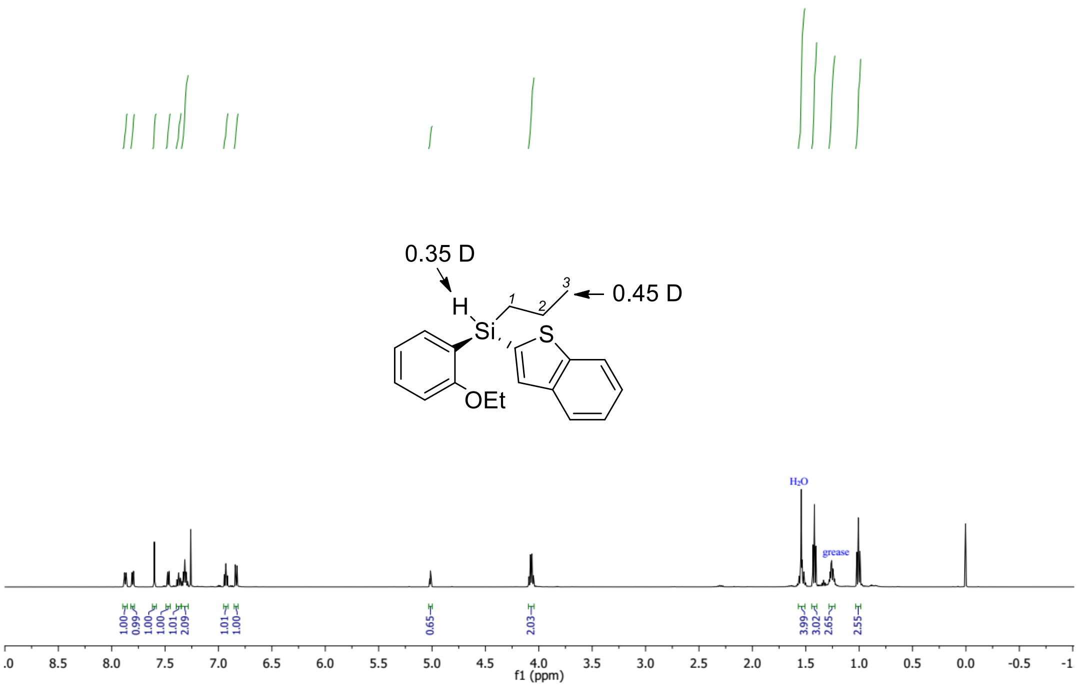

Supplementary Figure 16. ^{1}H NMR spectrum of **D-3cn**

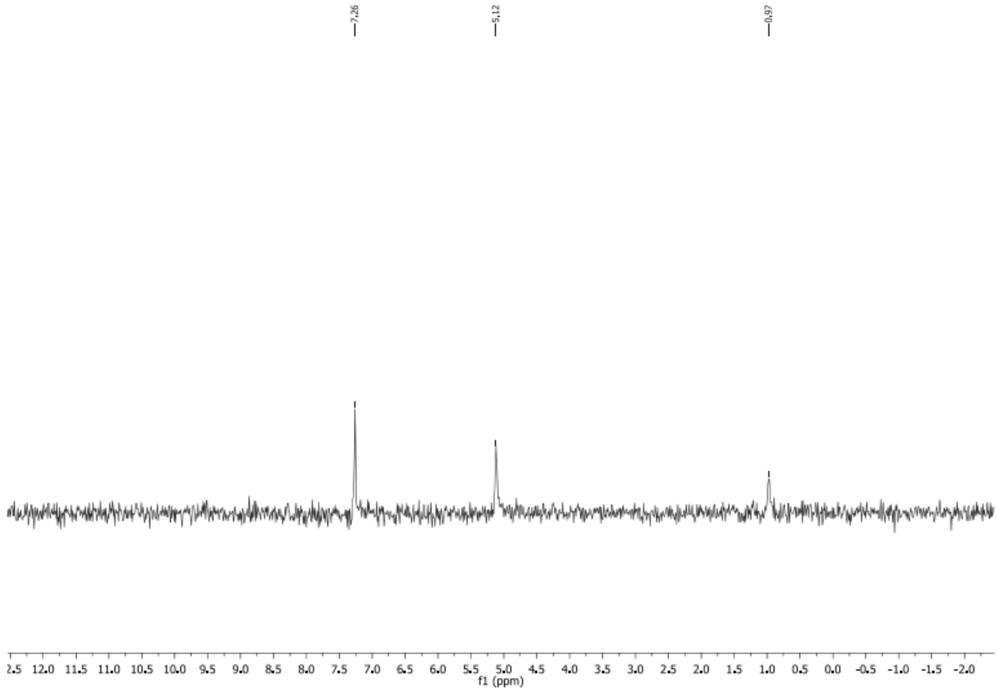

Supplementary Figure 17. ^{2}H NMR spectrum of **D-3cn**

Besides, the unreacted **D-2n** after standard reaction was also recovered. As shown in Supplementary Figure 18, the proton incorporation of recovered **D-2n** (0.34 H) was calculated by ^{1}H NMR analysis (7.44 ppm in $CDCl_3$).

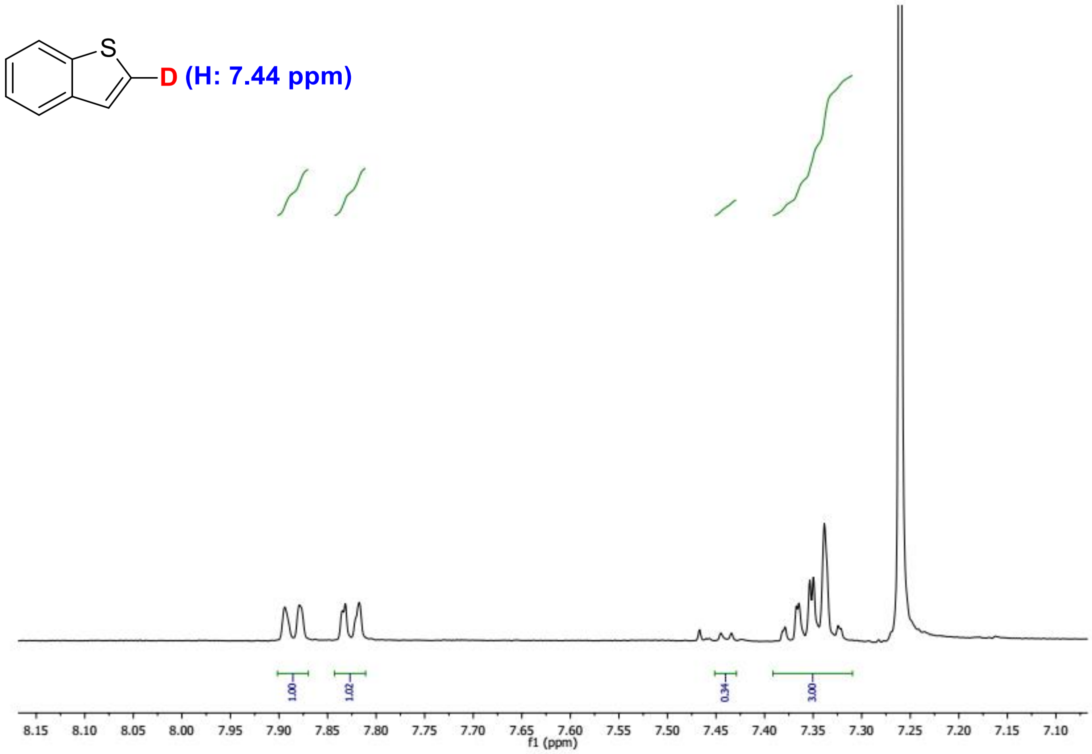

Supplementary Figure 18. ^{1}H NMR spectrum of recovered **D-2n** after standard reaction

To rationalize the observed H/D scrambling, we first carried out control experiment in the absence of [Rh]-H catalyst. To a sealed tube equipped with magnetic stirring bar were added substrate **1c** (19.2 mg, 0.1 mmol, 1.0 equiv.), deuterated benzothiophene **D-2n** (13.5 mg, 0.1 mmol, 1.0 equiv.) and toluene (1.0 mL). The reaction mixture was stirred at room temperature for 24 h and diluted with dichloromethane (2.0 mL). The organic layer was concentrated under reduced pressure and the remaining **D-2n** was recovered by preTLC as a white solid. As shown in Supplementary Figure 19, no proton incorporation of **D-2n** was detected by ^{1}H NMR analysis (7.44 ppm in $CDCl_3$), indicating the observed H/D scrambling of **D-3cn** was facilitated by [Rh]-H catalyst.

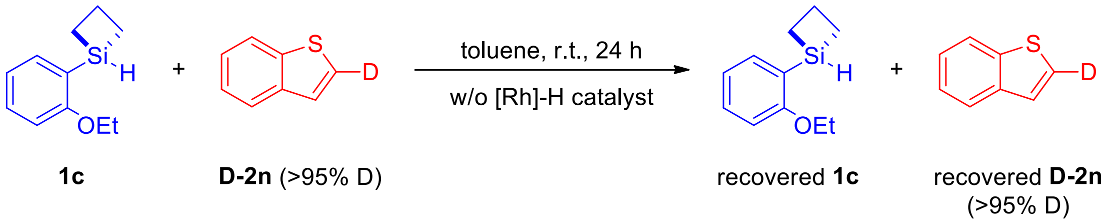

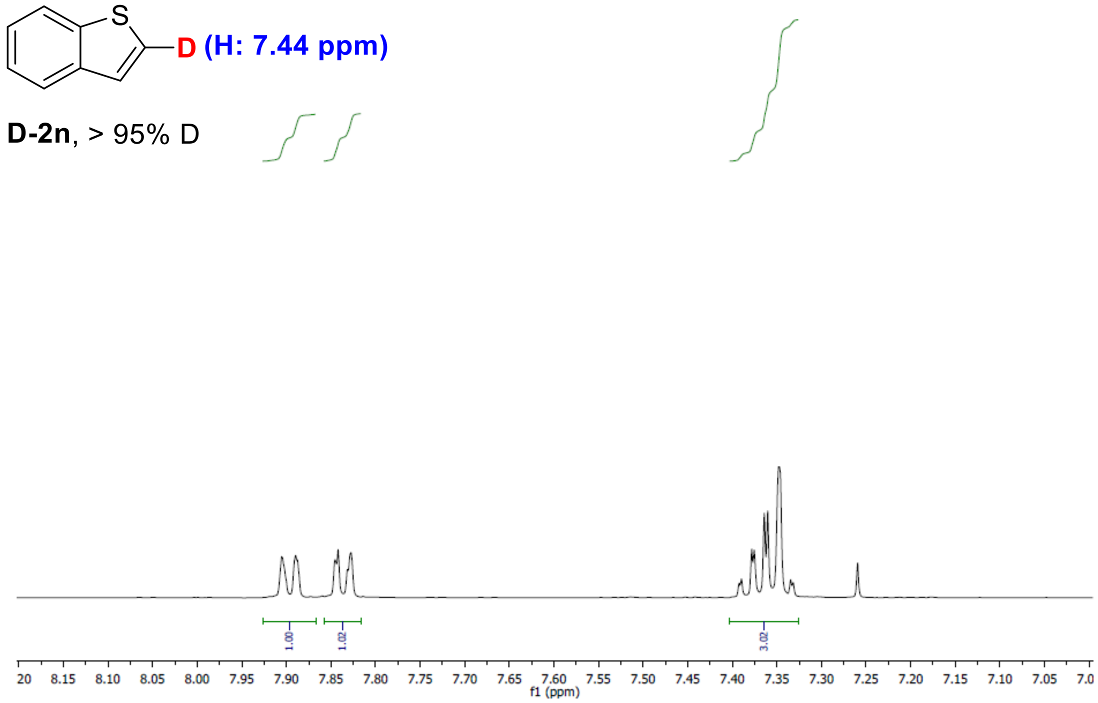

Supplementary Figure 19. ^{1}H NMR spectrum of recovered **D-2n** in the absence of [Rh]-H catalyst

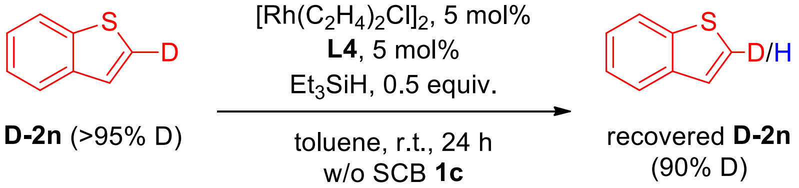

We then carried out control experiment in the absence of silacyclobutane **1c**. To a sealed tube equipped with magnetic stirring bar were added 0.5 mL of the Rh(**L4**)-H solution, deuterated benzothiophene **D-2n** (13.5 mg, 0.1 mmol) and the total volume of toluene solution was adjusted to be 1.0 mL. The reaction mixture was stirred at r.t. for 24 h and diluted with dichloromethane (2.0 mL). The organic layer was concentrated under reduced pressure and the remaining **D-2n** was recovered by preTLC as a white solid. As shown in Supplementary Figure 20, the proton incorporation of **D-2n** (0.10 H) was calculated by ^{1}H NMR analysis (7.44 ppm in $CDCl_3$), indicating the excess Et_3SiH

acted as the H source in the presence of [Rh]-H catalyst.

Interpretation: The observed H/D scrambling between deuterium source (**D-2n**) and proton socure (**1c** or Et_3SiH) occurred outside of the catalytic cycle (ring-opening to generate **D-3cn**) in the presence of [Rh]-H catalyst.

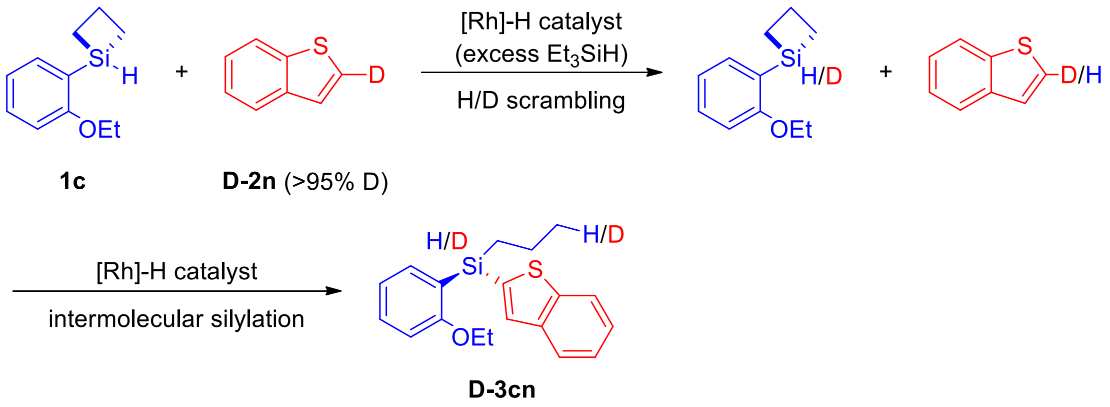

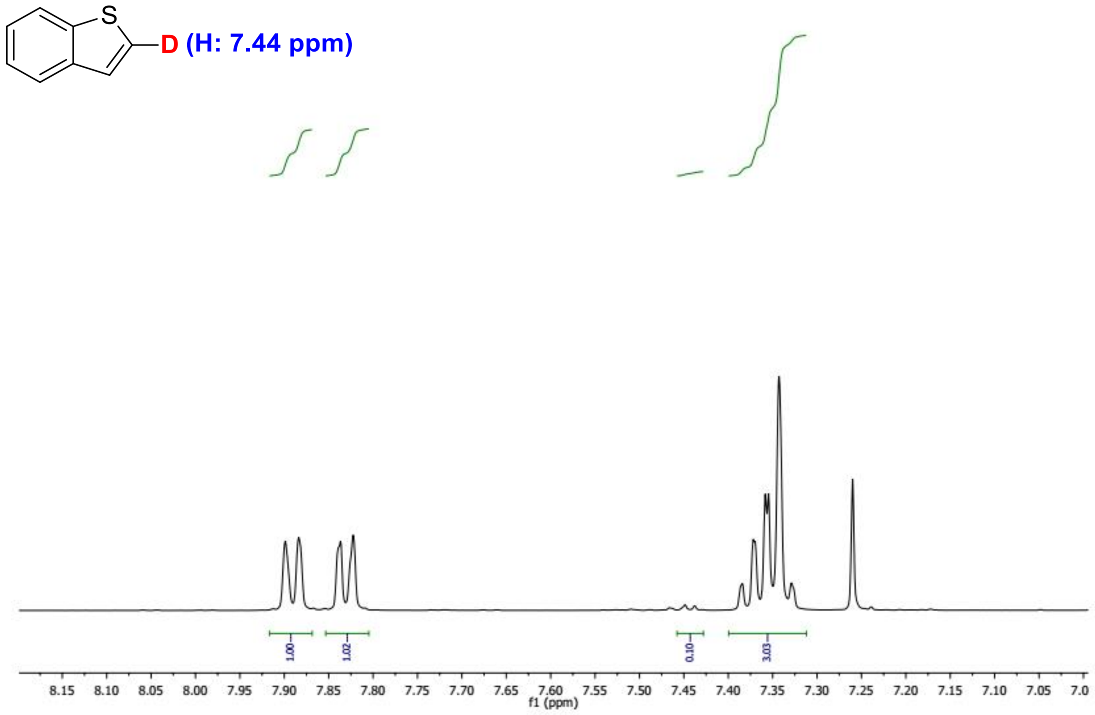

Supplementary Figure 20. ^{1}H NMR spectrum of recovered **D-2n** in the absence of silacylcobutane **1c**

3.4 KIE Experiment

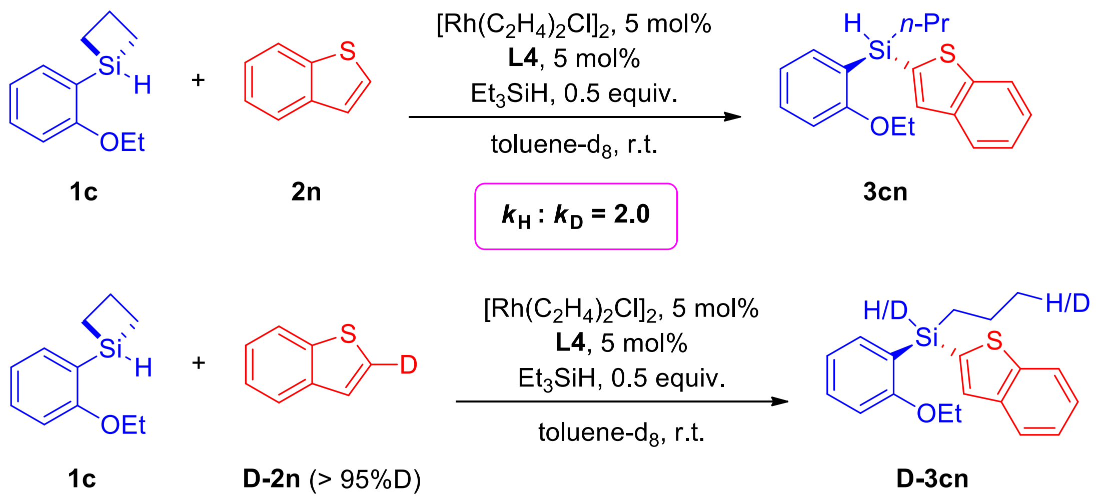

The KIE was determined by the relative ratio of the initial rates of two parallel reactions using **1c** and **2n**, **1c** and **D-2n**, respectively. To two seperate NMR tubes with J. Young valve was added 0.5 mL of the Rh(**L4**)-H solution. To one tube were added substrate **1c** (19.2 mg, 0.1 mmol, 1.0 equiv.), benzothiophene **2n** (13.4 mg, 0.1 mmol, 1.0 equiv.) and to the other tube were added substrate **1c** (19.2 mg, 0.1 mmol, 1.0 equiv.), and deuterated benzothiophene **D-2n** (13.5 mg, 0.1 mmol, 1.0 equiv.). These NMR tubes were then removed from glovebox and subjected to ^{1}H NMR analysis using 1,2-dimethoxyethane as the internal standard.

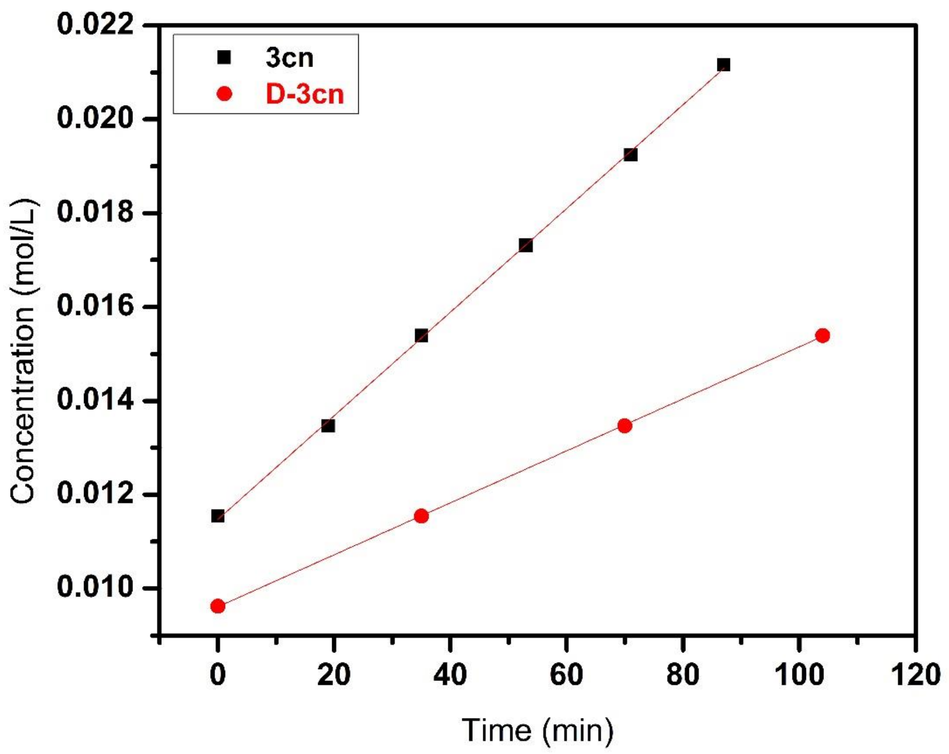

Supplementary Figure 21. Representative plot for the KIE experiment. For the formation of **3cn**, $y = 0.0001105x + 0.01147$, $R^2 = 0.9994$. For the formation of **D-3cn**, $y = 0.00005544x + 0.00961$, $R^2 = 0.99993$. Thus $k_H/k_D = 2.0$.

Interpretation: The first ^{1}H NMR data acquisition was assigned as the beginning of timing. No induction period was detected as the monohydrosilanes of **3cn**/**D-3cn** could be detected after the first ^{1}H NMR sampling. The KIE was determined to be 2.0, indicating C-H bond activation might be involved in the rate-limiting step.

3.5 Intermolecular Competition Experiment

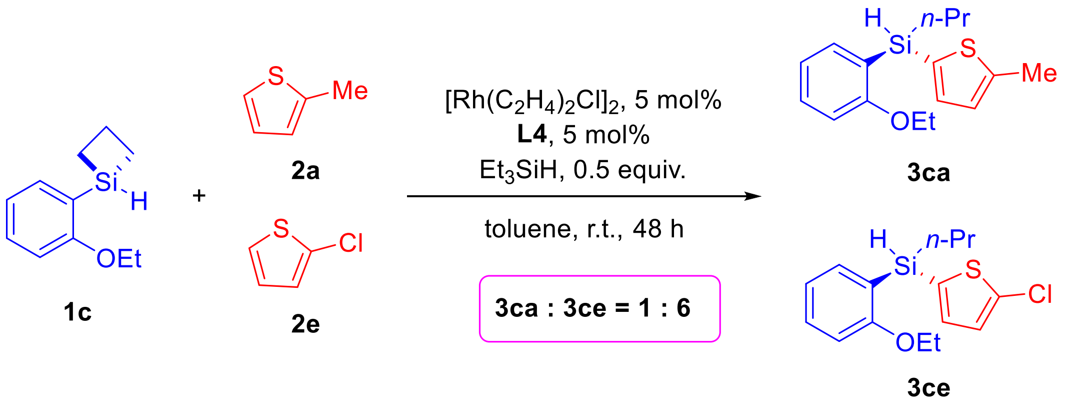

To further demonstrate the rate-limiting step of this reaction we also conducted intermolecular competition experiment. To a sealed tube equipped with magnetic stirring bar were added 0.5 mL of the Rh(**L4**)-H solution, substrate **1c** (19.2 mg, 0.1 mmol, 1.0 equiv.), 2-methylthiophene **2a** (19.6 mg, 0.2 mmol, 2.0 equiv.), 2-chlorothiophene **2e** (23.7 mg, 0.2 mmol, 2.0 equiv.), and the total volume of toluene solution was adjusted to be 1.0 mL. The reaction mixture was stirred at room temperature for 48 h and diluted with dichloromethane (2.0 mL). The organic layer was concentrated under reduced pressure and the ratio of **3ca** to **3ce** was determined by crude ^{1}H NMR analysis. Characterization of **3ce**: ^{1}H NMR (500 MHz, $CDCl_3$) δ 7.42 (d, J = 5.0 Hz, 1H), 7.36 (t, J = 7.5 Hz, 1H), 7.14 (d, J = 5.0 Hz, 1H), 6.95-6.92 (m, 2H), 6.82 (d, J = 10.0 Hz, 1H), 4.86 (t, J = 5.0 Hz, 1H), 4.09-4.04 (m, 2H), 1.50-1.45 (m, 2H), 1.42 (t, J = 7.5 Hz, 3H), 1.18-1.13 (m, 2H), 0.98 (t, J = 7.5 Hz, 3H); ^{13}C NMR (125 MHz, $CDCl_3$) δ 163.6, 136.9, 135.9, 135.4, 134.4, 132.1, 127.4, 122.2, 120.7, 110.3, 63.5, 18.2, 17.8, 15.7, 14.9.

Interpretation: The ratio of **3ca**/**3ce** was determined to be 1:6. The faster reaction of

thiophene bearing electron-withdrawing group suggested a faster oxidative addition process for the cleavage of C-H bond, which further indicated C-H bond activation was involved in the rate-limiting step.

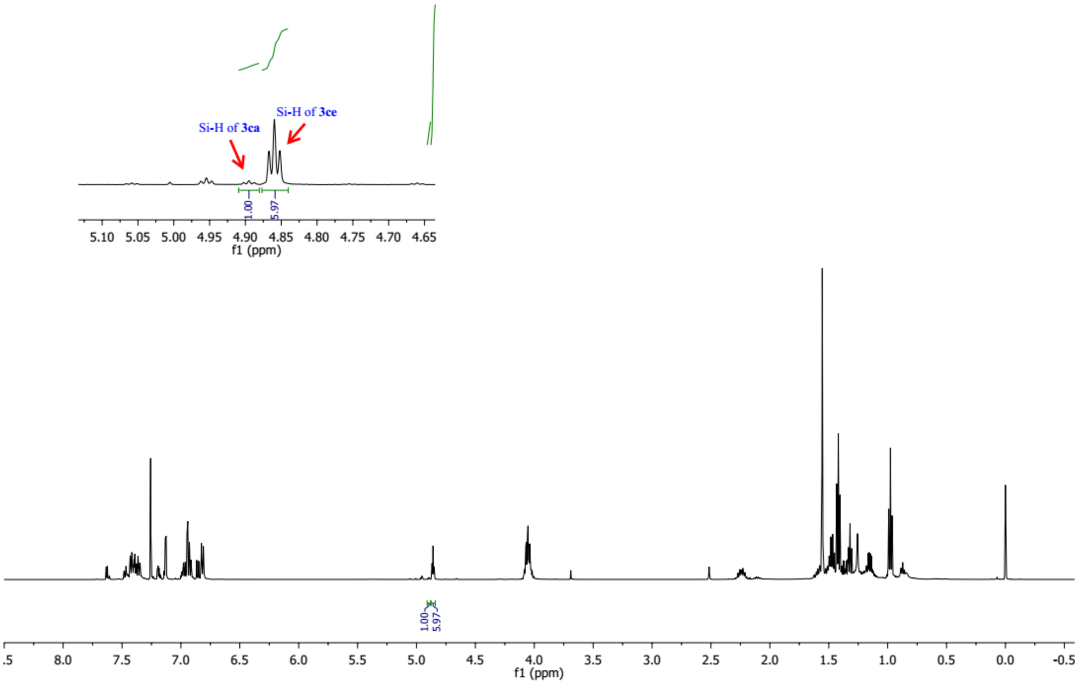

Supplementary Figure 22. Crude ^{1}H NMR spectrum of the mixture of **3ca** and **3ce**

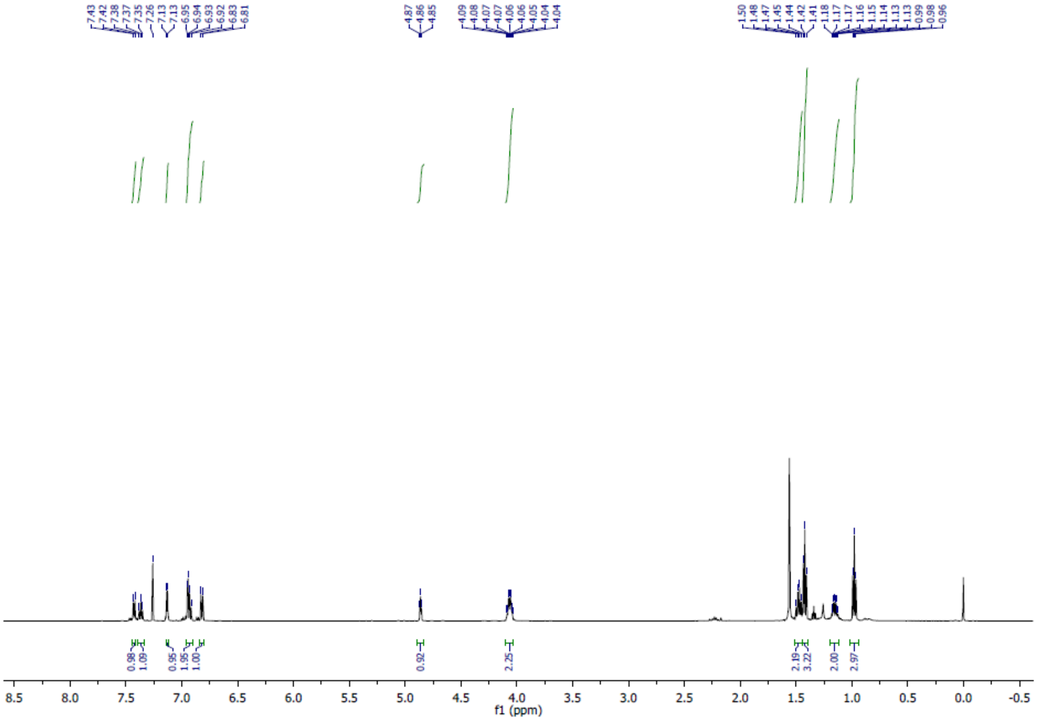

Supplementary Figure S23. ^{1}H NMR spectrum of **3ce**

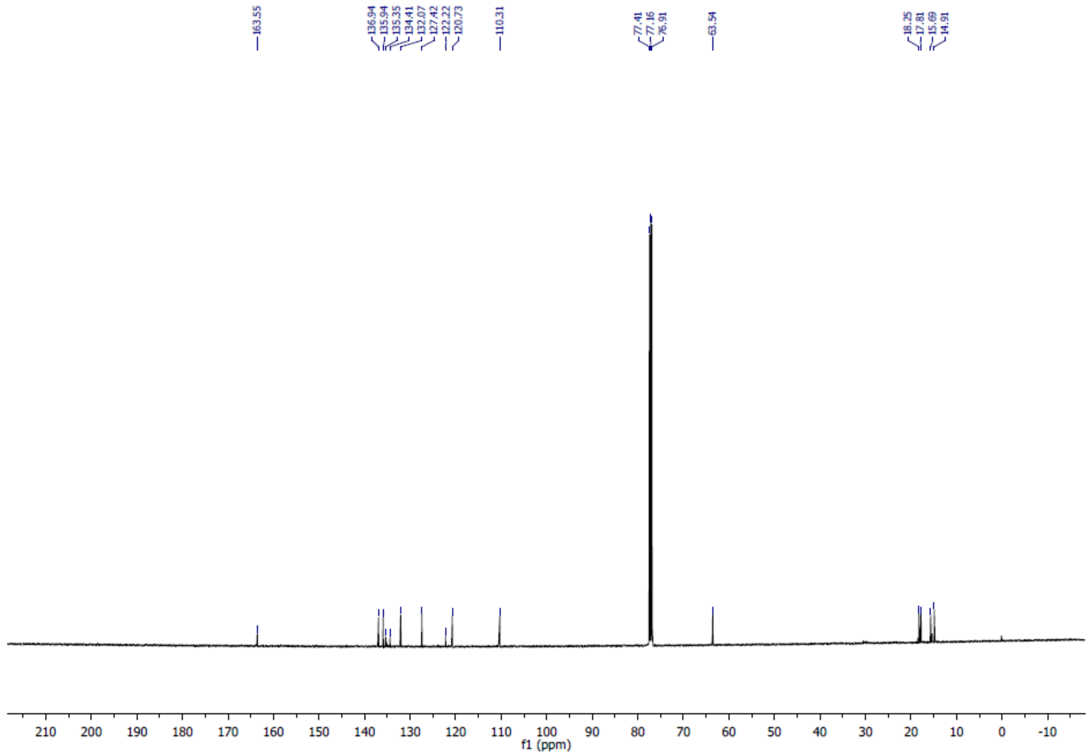

Supplementary Figure 24. ^{13}C NMR spectrum of **3ce**

3.6 Copies of ^1H, ^{13}C, ^{19}F and ^{29}Si NMR Spectra

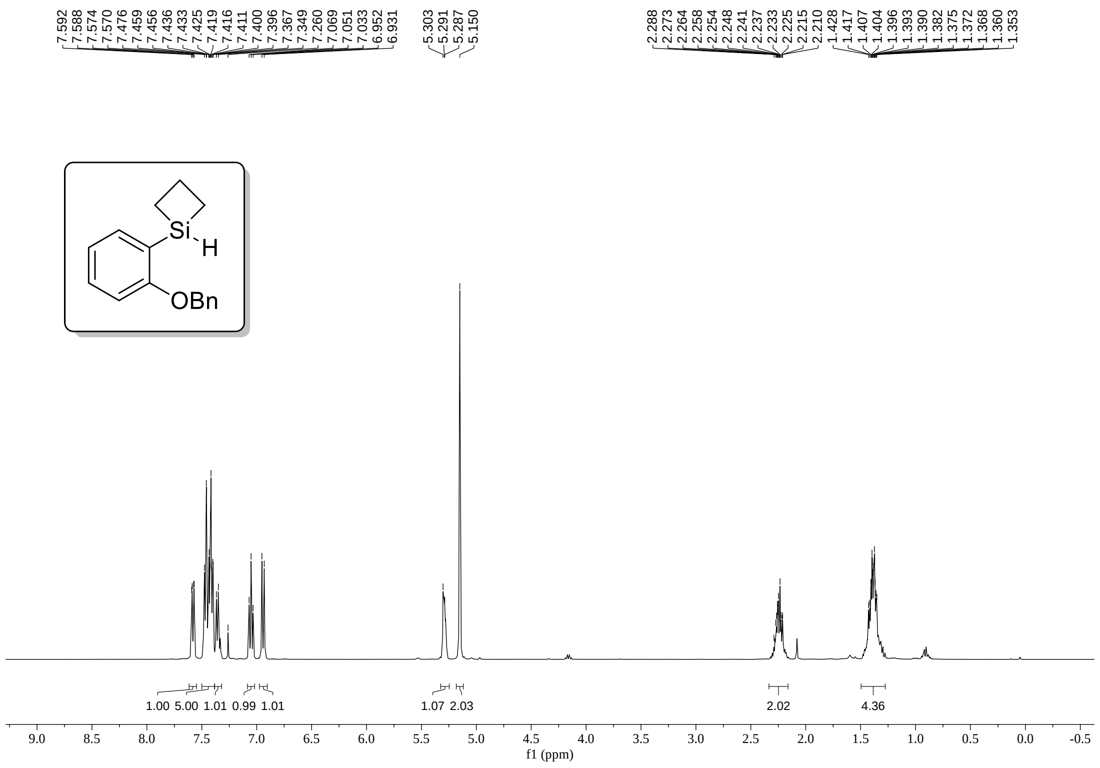

Supplementary Figure 25. ^1H NMR spectrum of **1a**

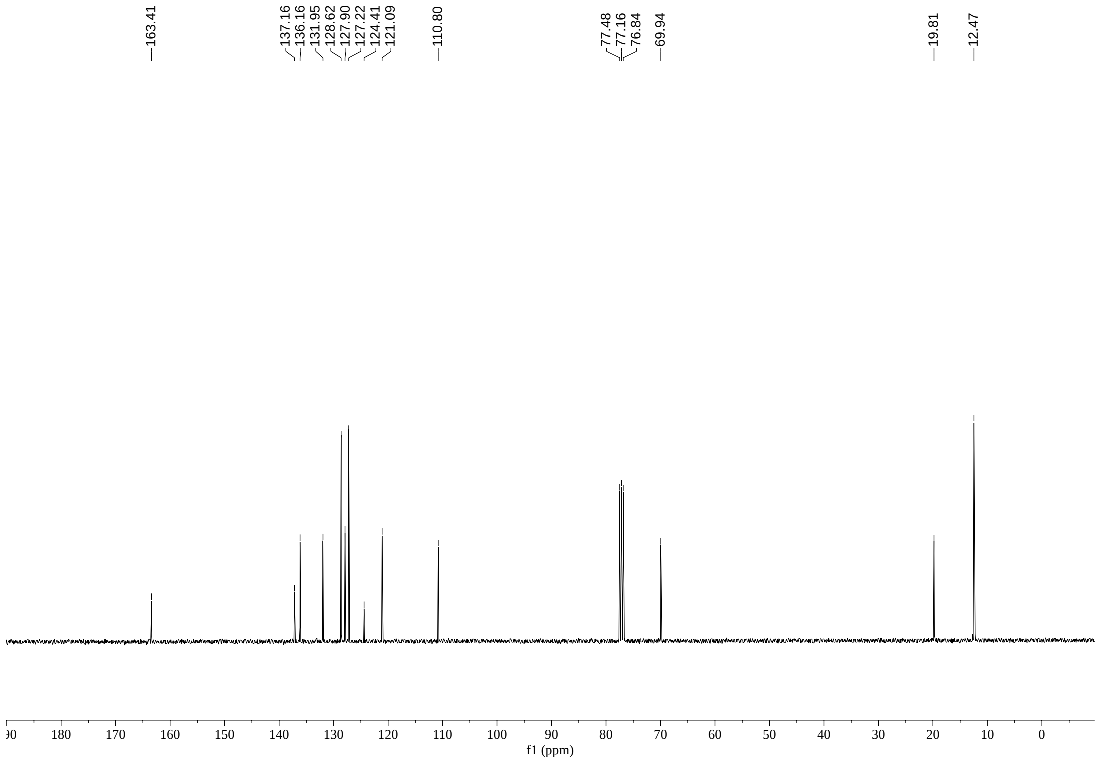

Supplementary Figure 26. ^{13}C NMR spectrum of **1a**

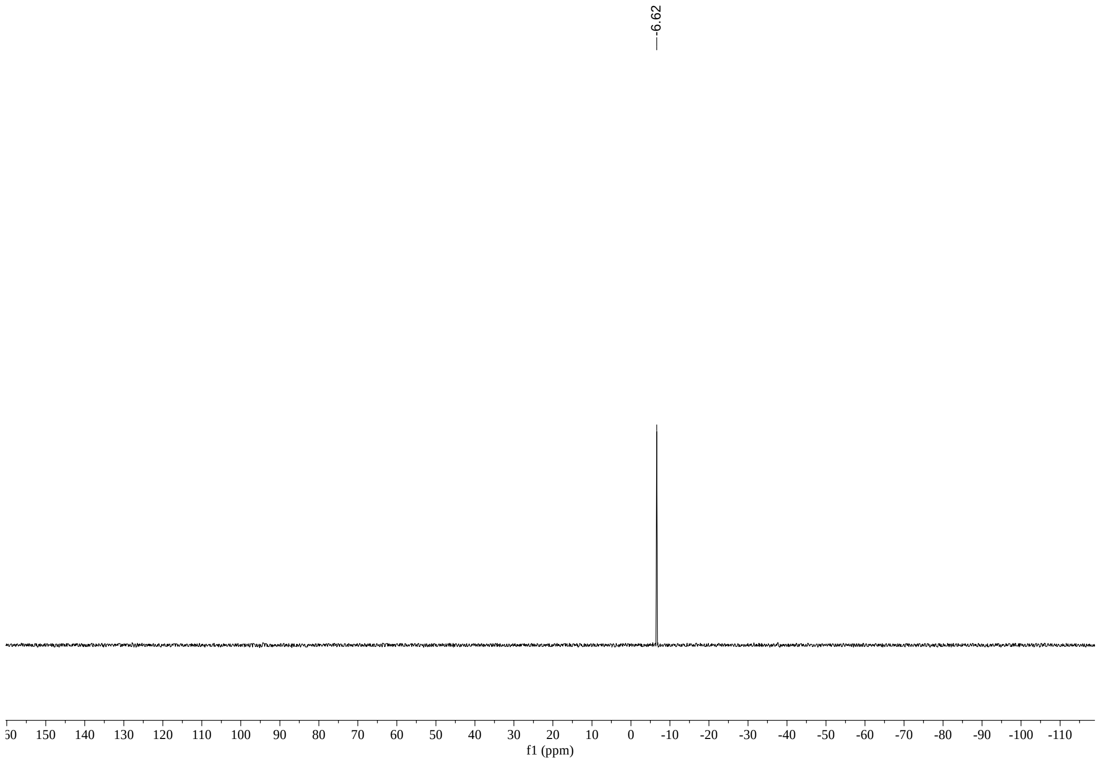

Supplementary Figure 27. ^{29}Si NMR spectrum of **1a**

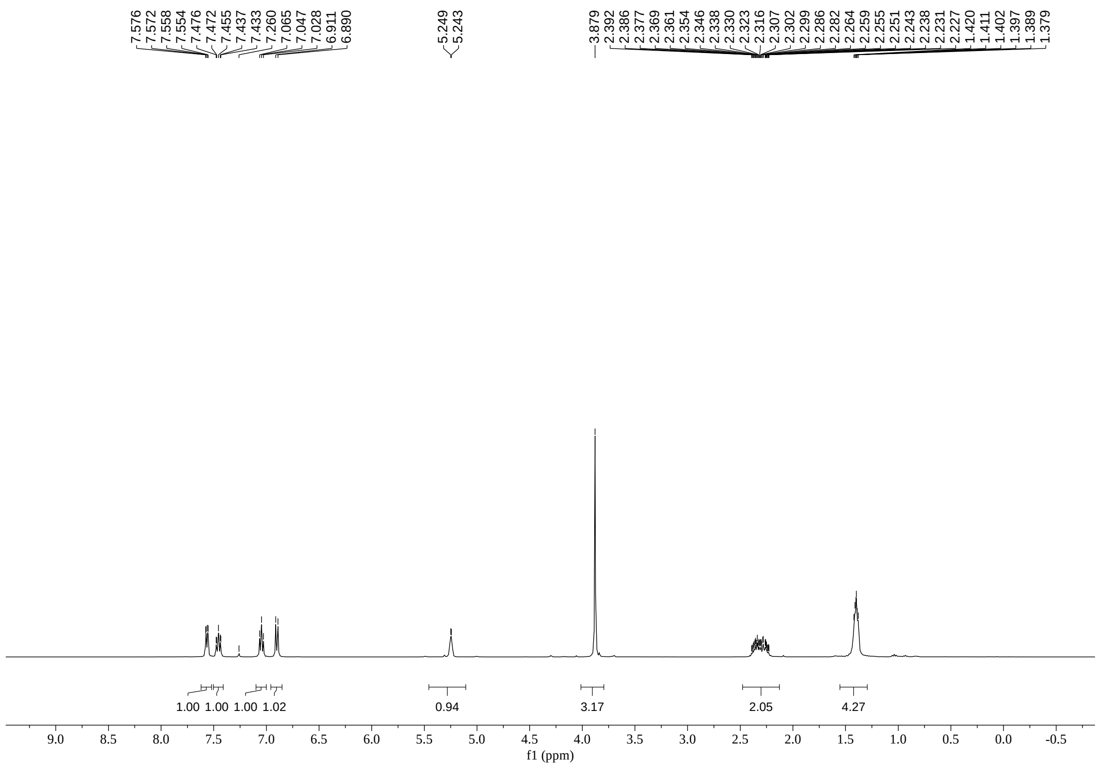

Supplementary Figure 28. ^{1}H NMR spectrum of **1b**

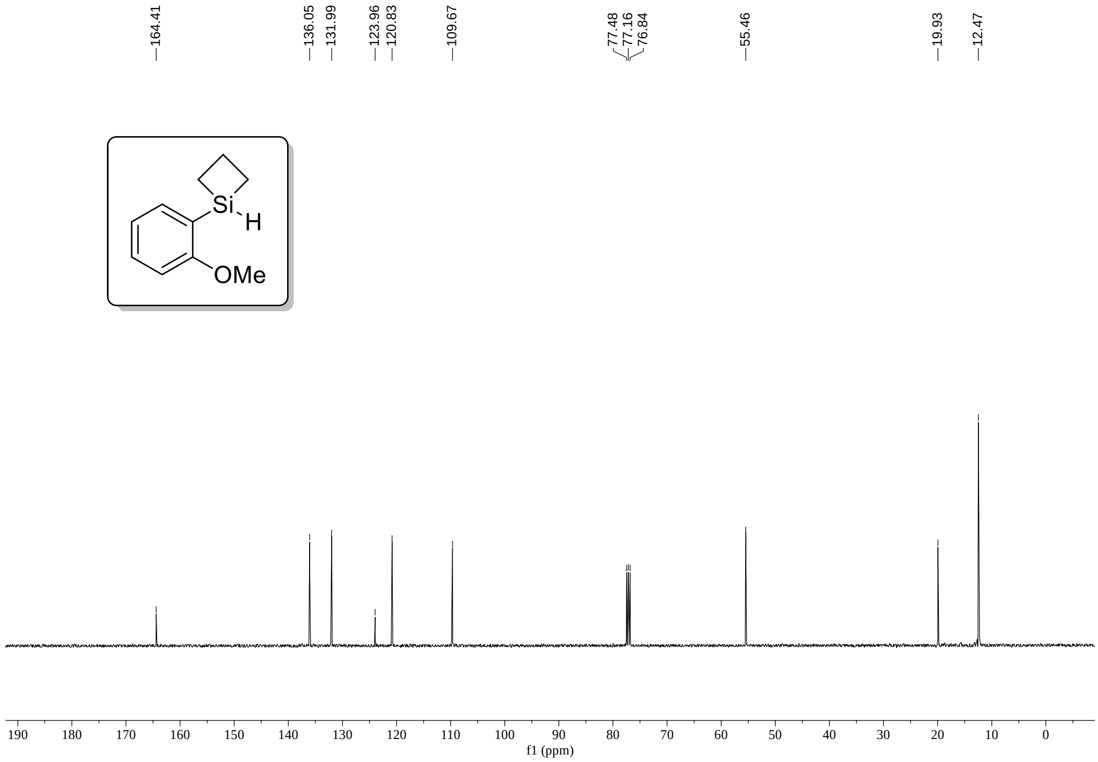

Supplementary Figure 29. ^{13}C NMR spectrum of **1b**

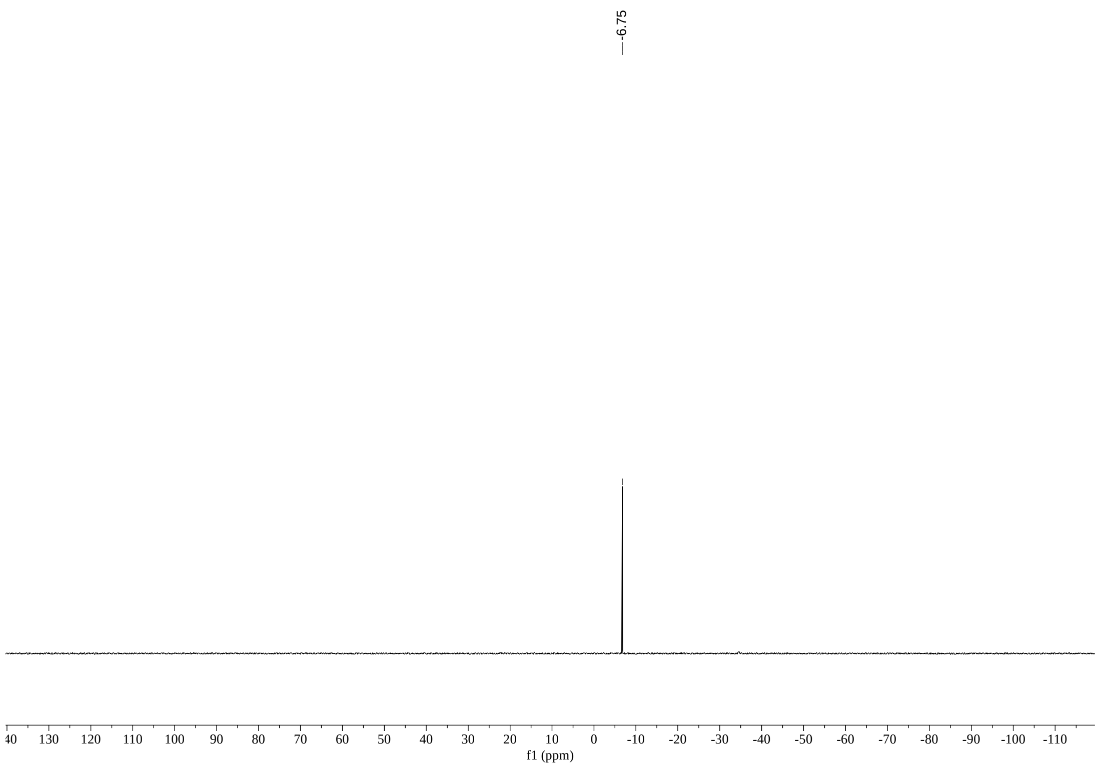

Supplementary Figure 30. ^{29}Si NMR spectrum of **1b**

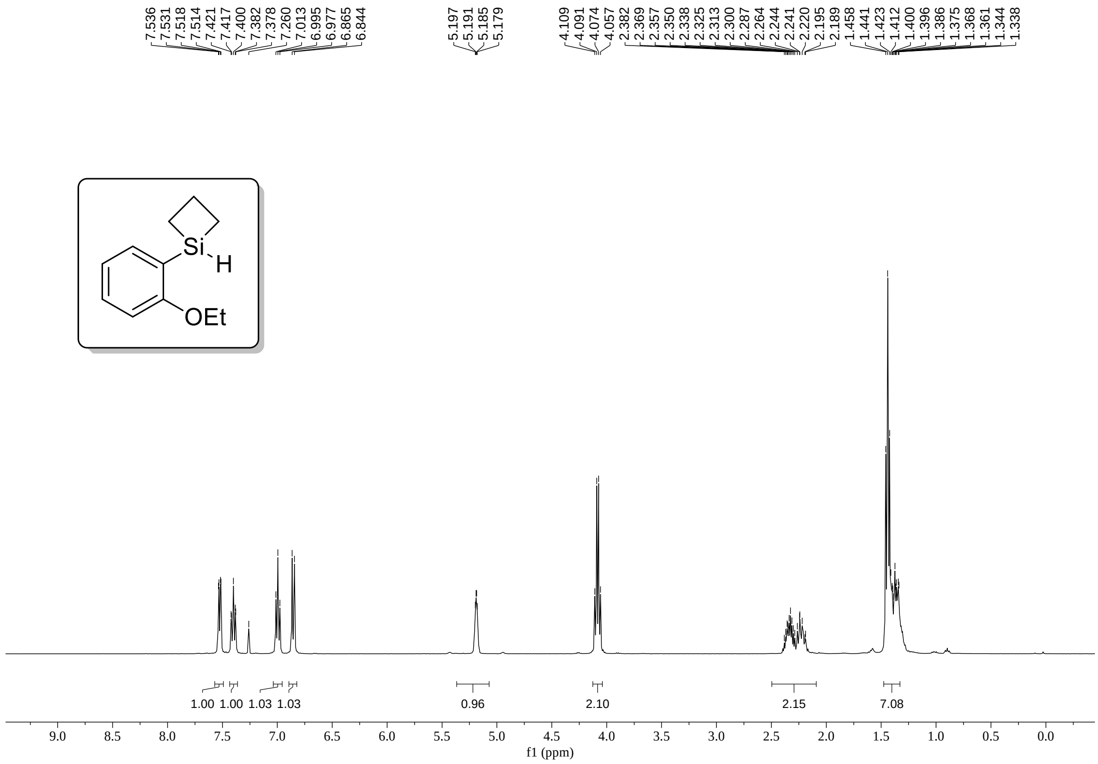

Supplementary Figure 31. ^{1}H NMR spectrum of **1c**

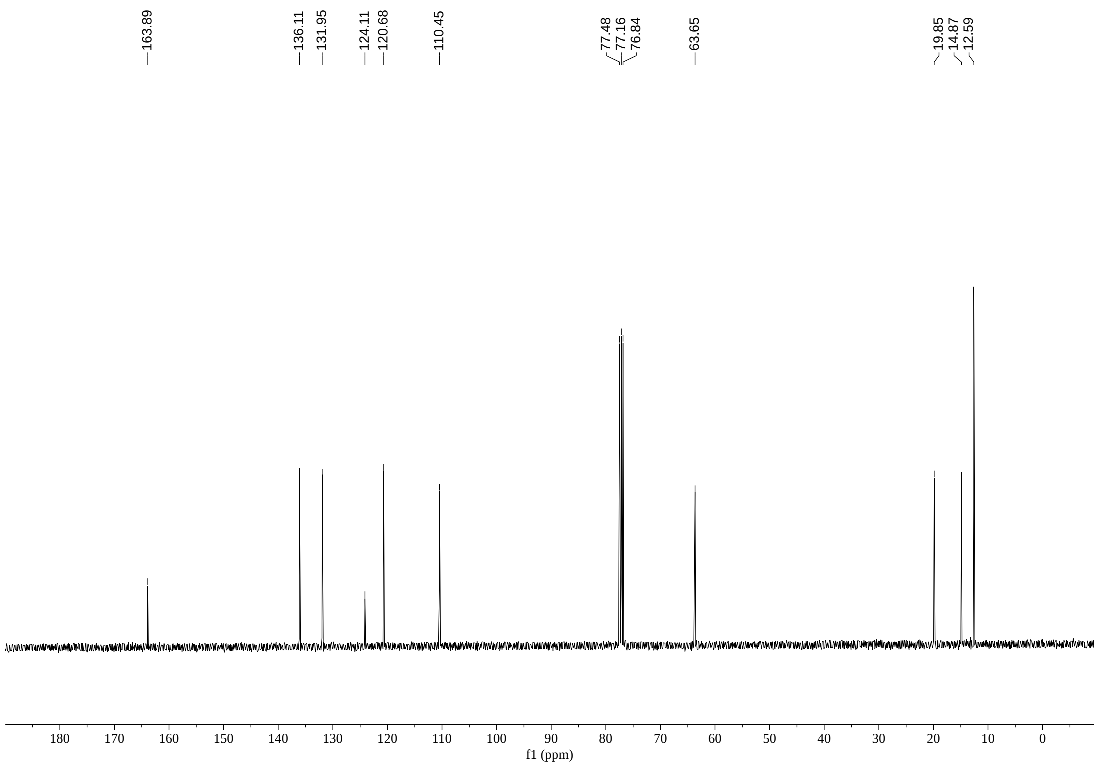

Supplementary Figure 32. ^{13}C NMR spectrum of **1c**

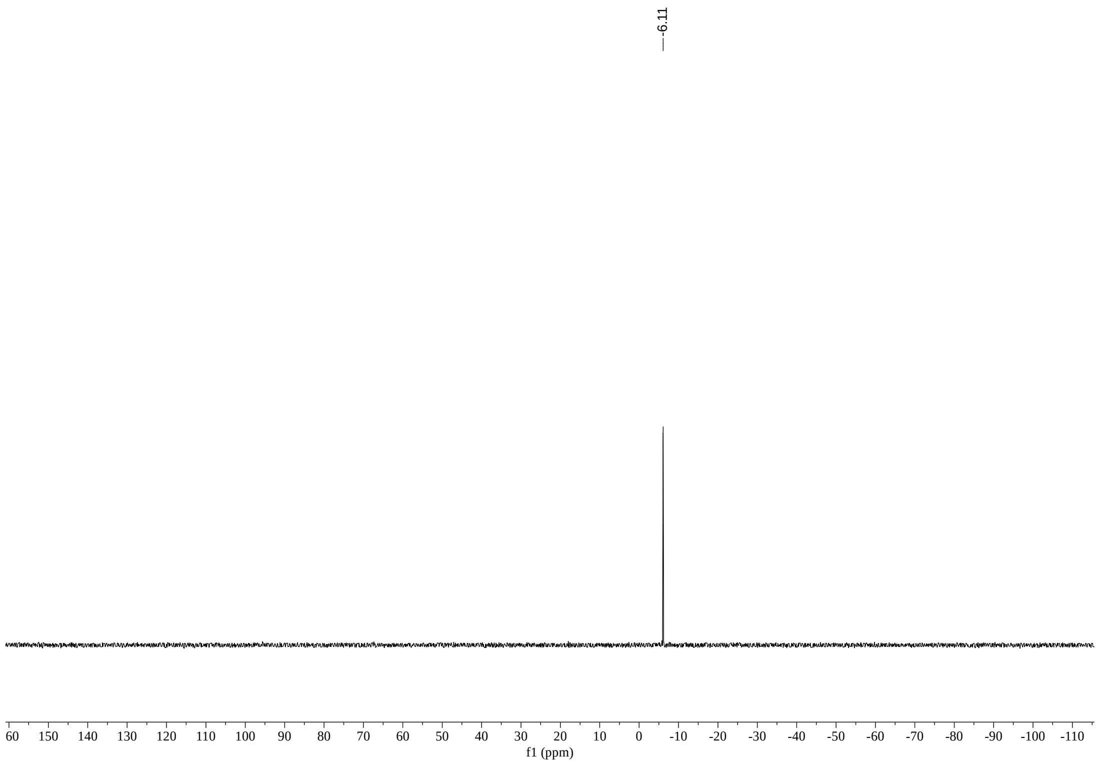

Supplementary Figure 33. ^{29}Si NMR spectrum of **1c**

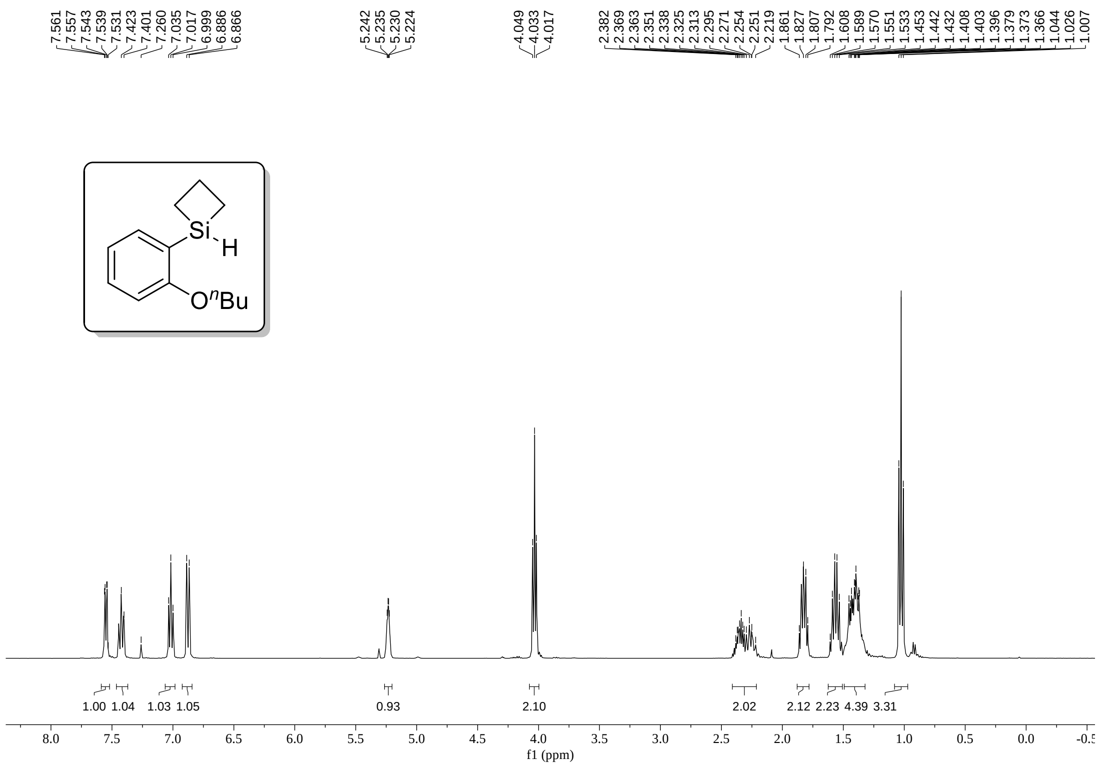

Supplementary Figure 34. ^{1}H NMR spectrum of **1d**

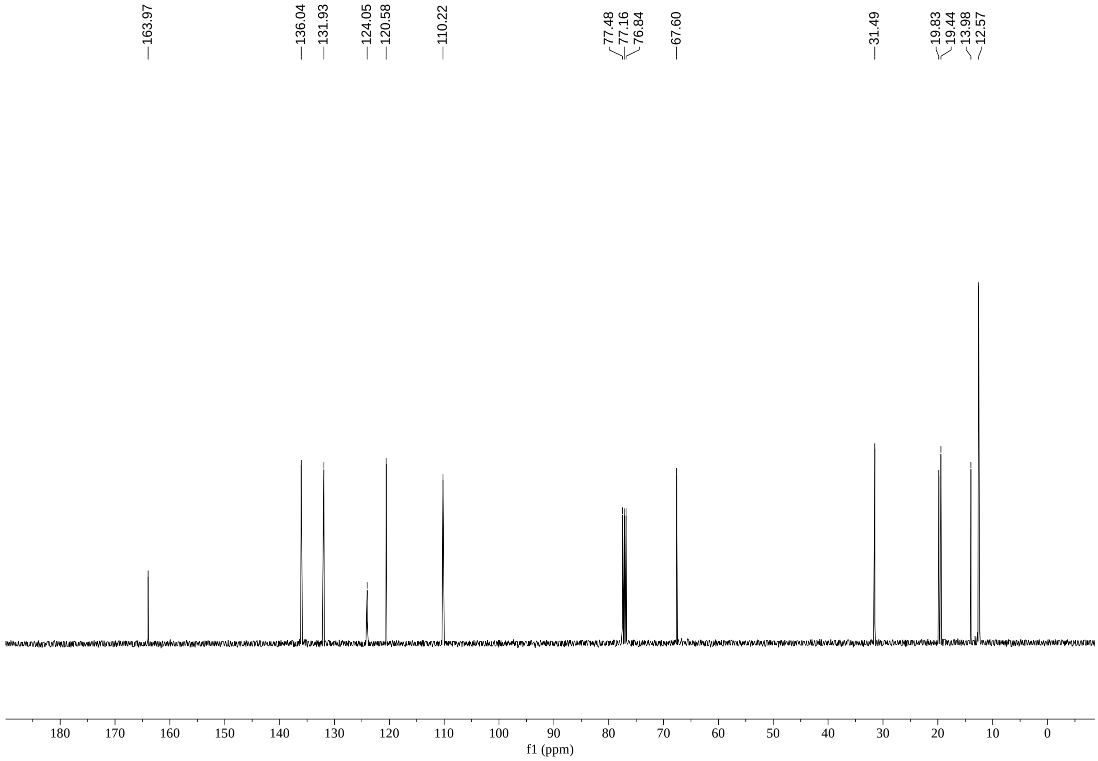

Supplementary Figure 35. ^{13}C NMR spectrum of **1d**

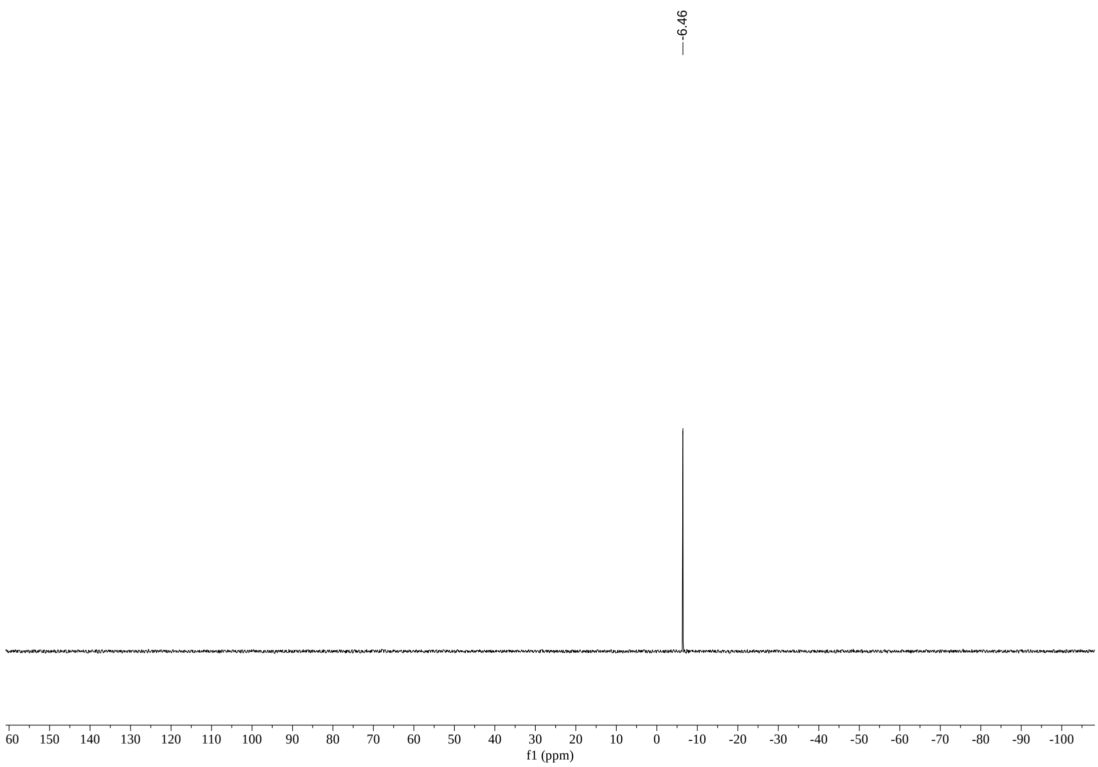

Supplementary Figure 36. ^{29}Si NMR spectrum of **1d**

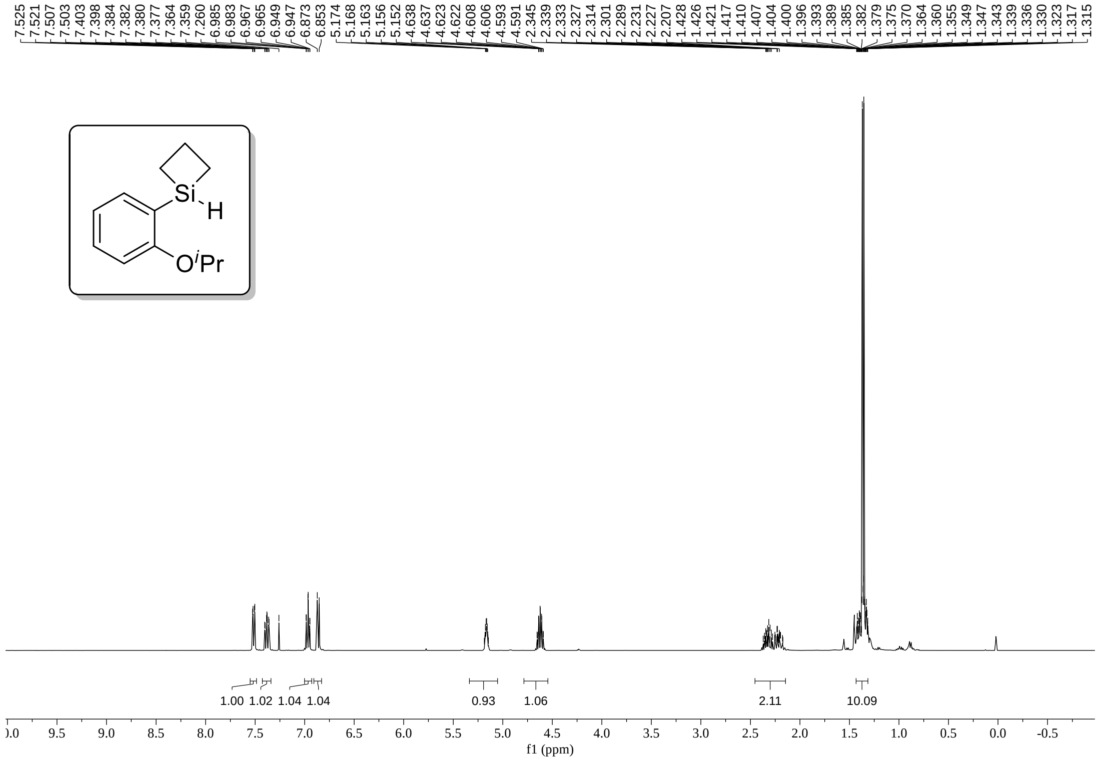

Supplementary Figure 37. ^{1}H NMR spectrum of **1e**

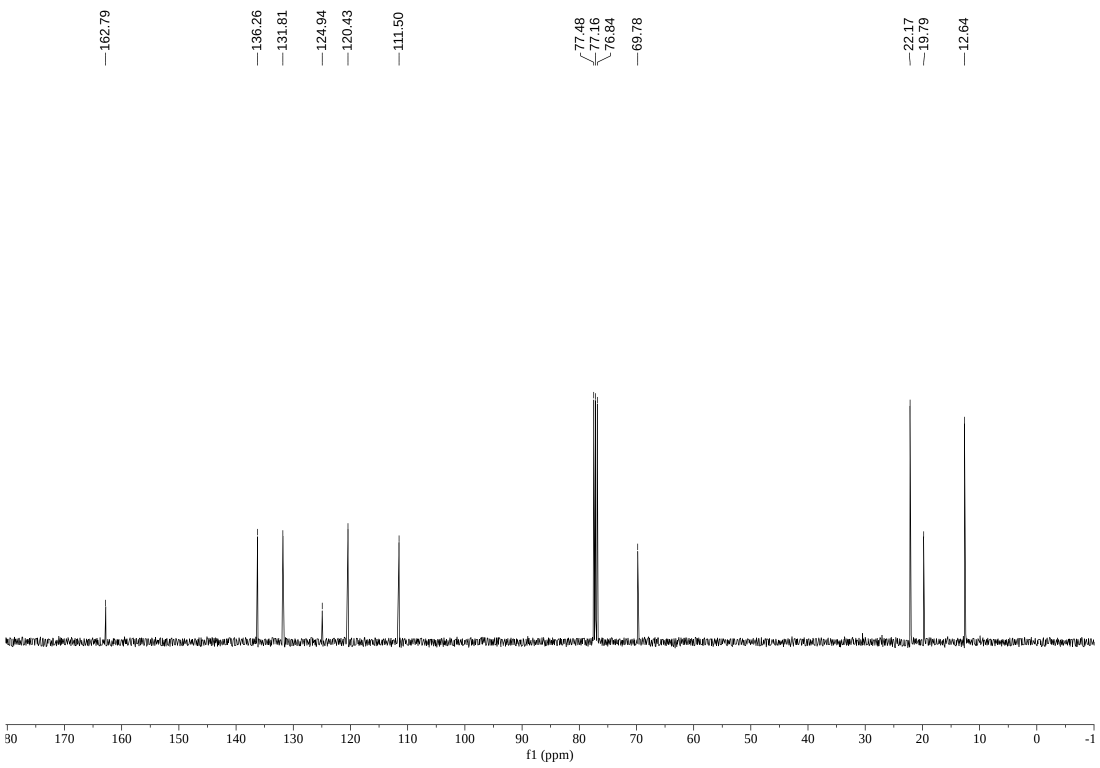

Supplementary Figure 38. ^{13}C NMR spectrum of **1e**

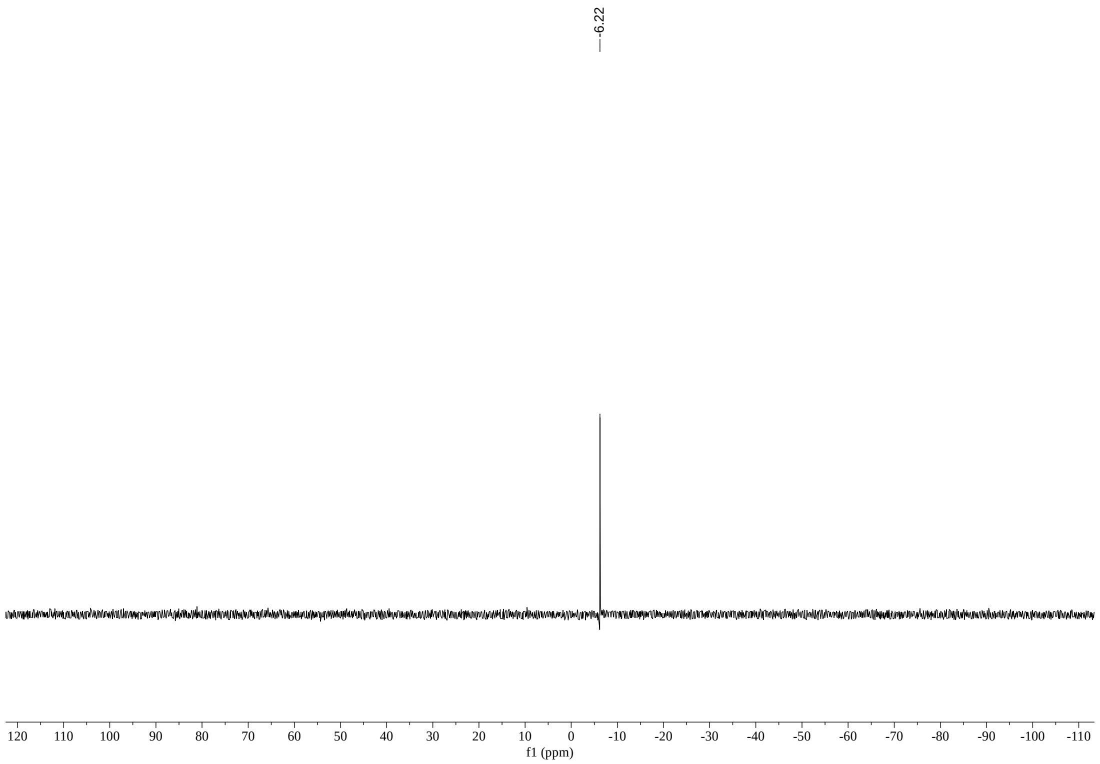

Supplementary Figure 39. ^{29}Si NMR spectrum of **1e**

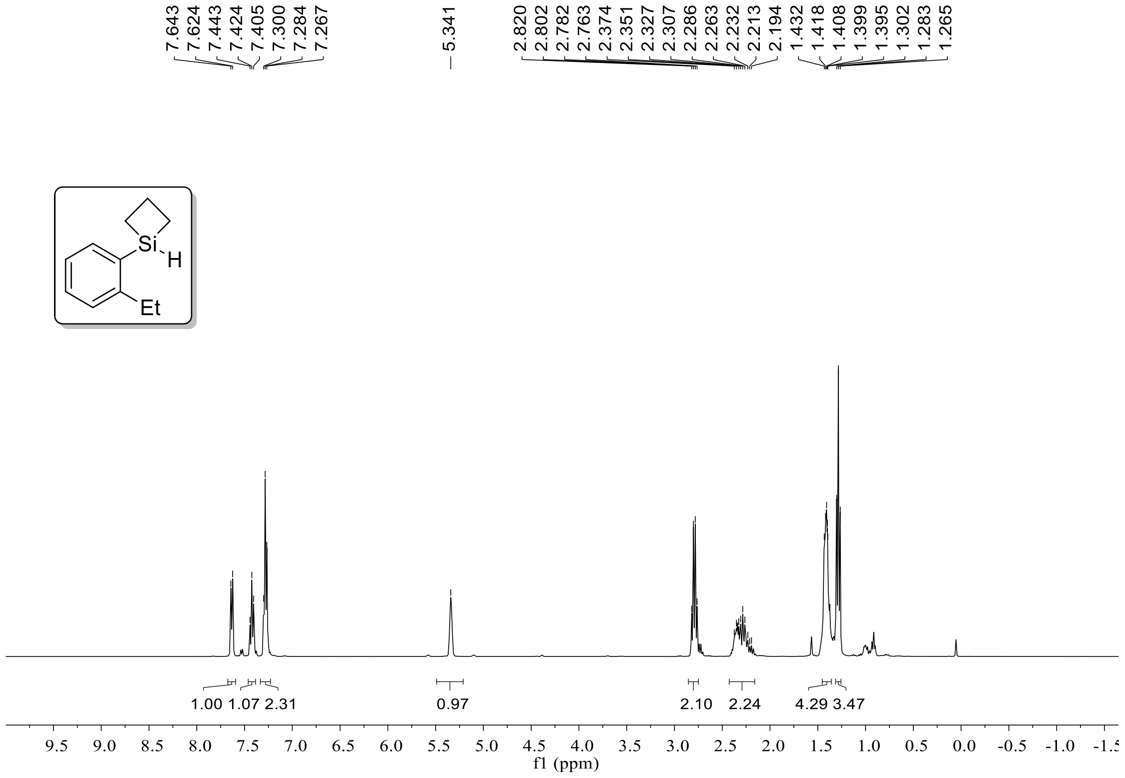

Supplementary Figure 40. ^{1}H NMR spectrum of **1f**

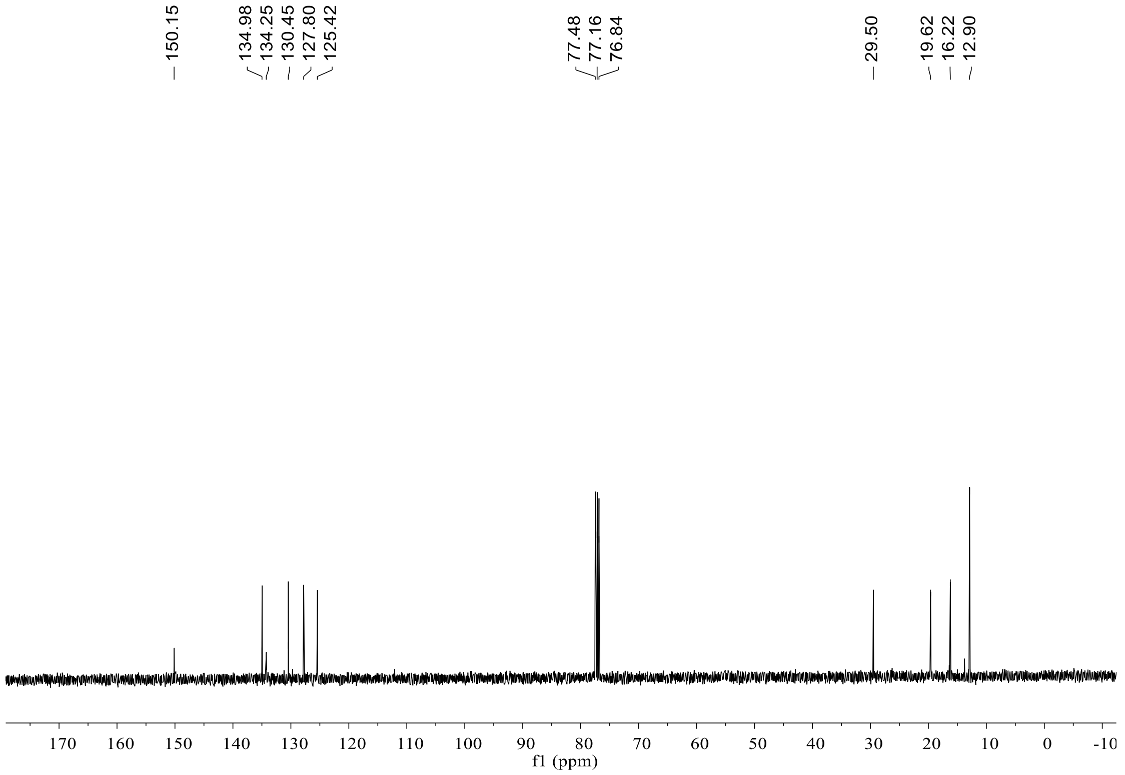

Supplementary Figure 41. ^{13}C NMR spectrum of **1f**

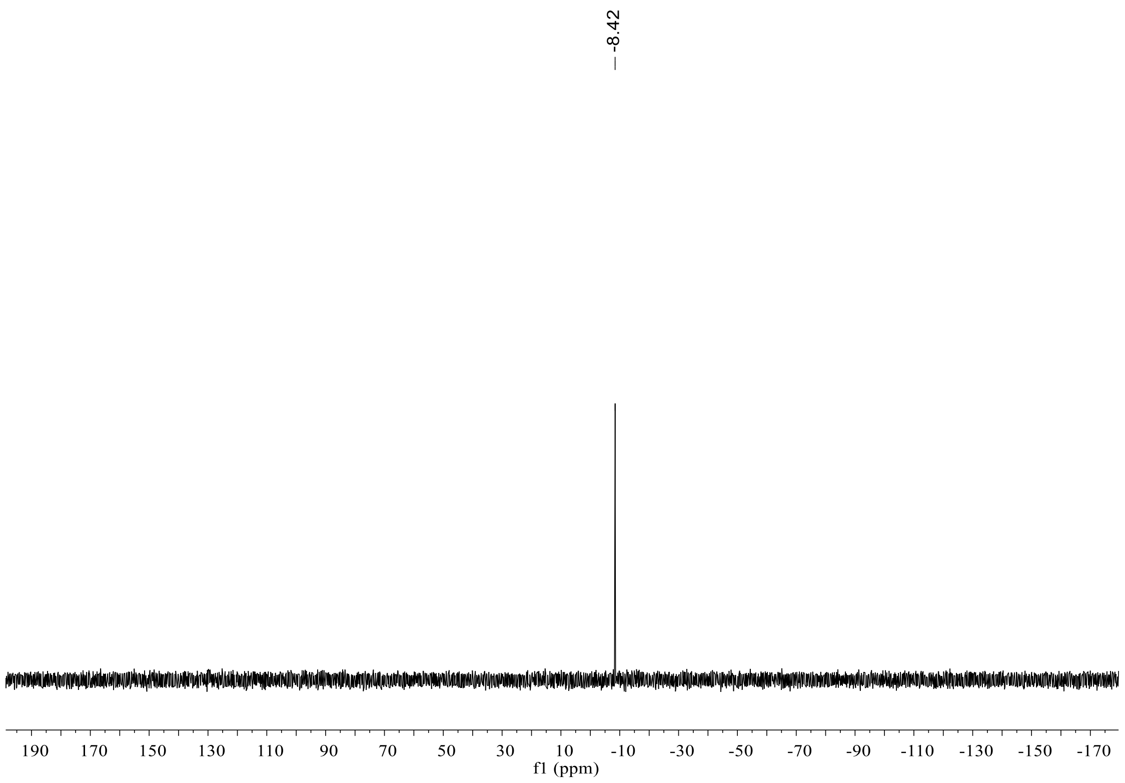

Supplementary Figure 42. ^{29}Si NMR spectrum of **1f**

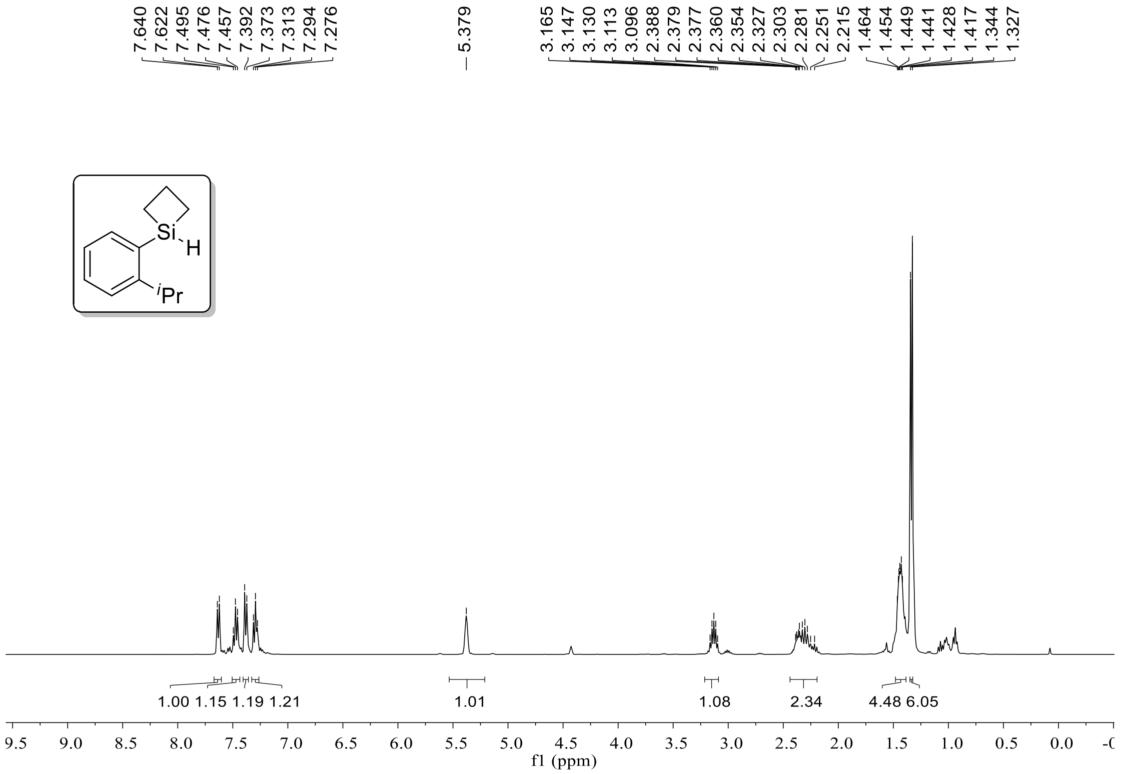

Supplementary Figure 43. ^{1}H NMR spectrum of **1g**

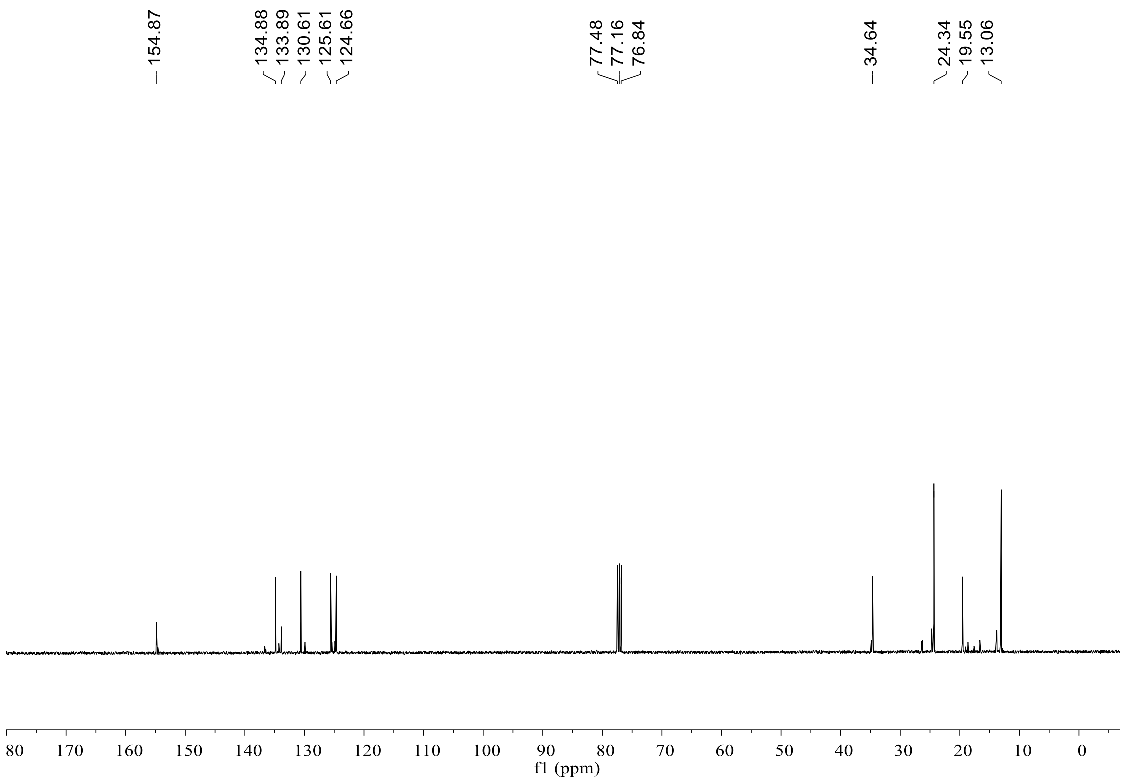

Supplementary Figure 44. ^{13}C NMR spectrum of **1g**

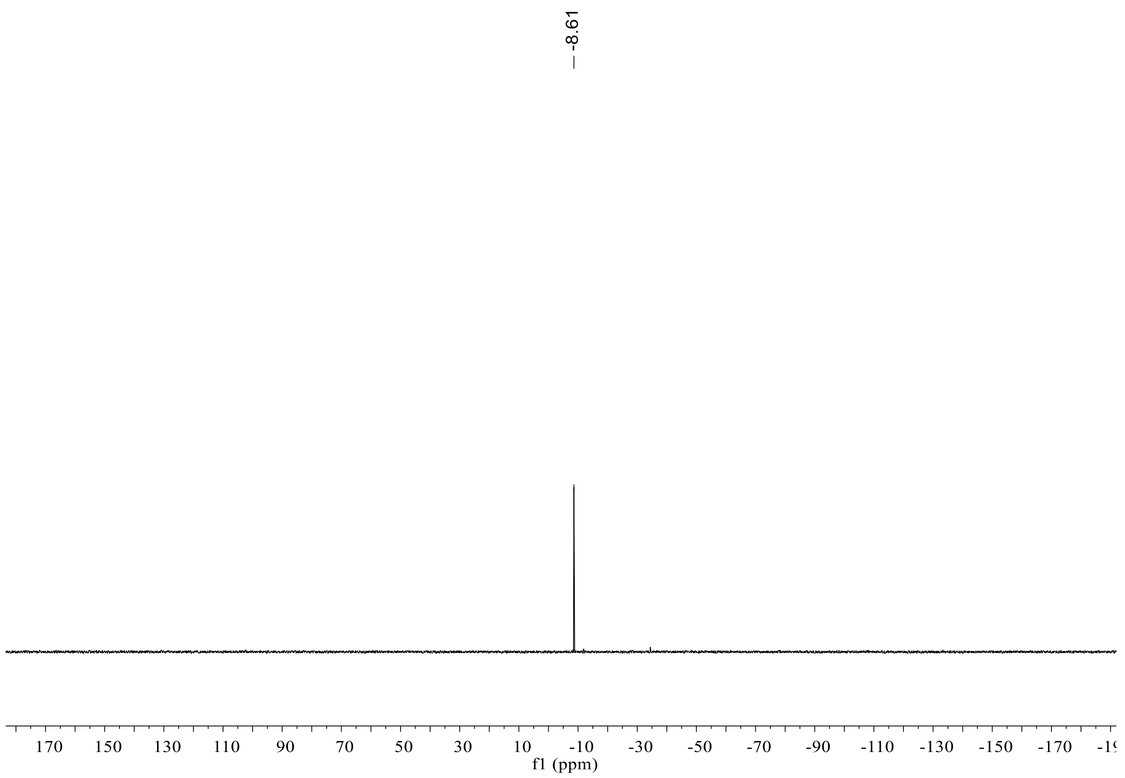

Supplementary Figure 45. ^{29}Si NMR spectrum of **1g**

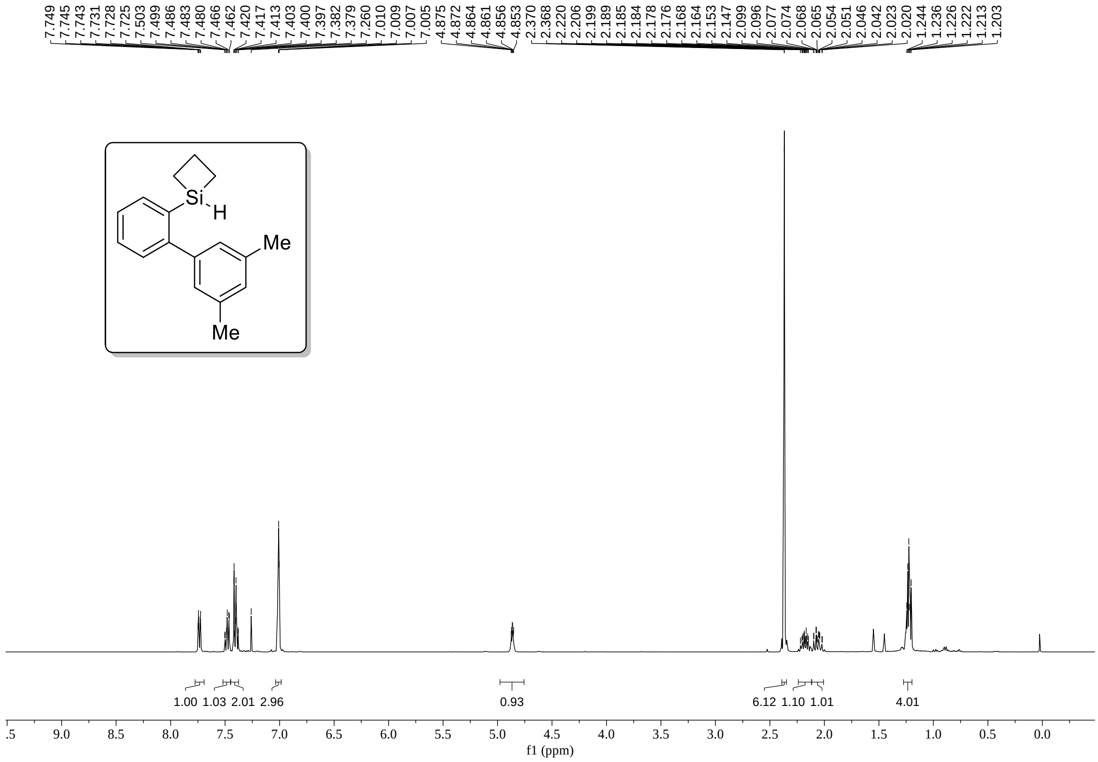

Supplementary Figure 46. ^{1}H NMR spectrum of **1h**

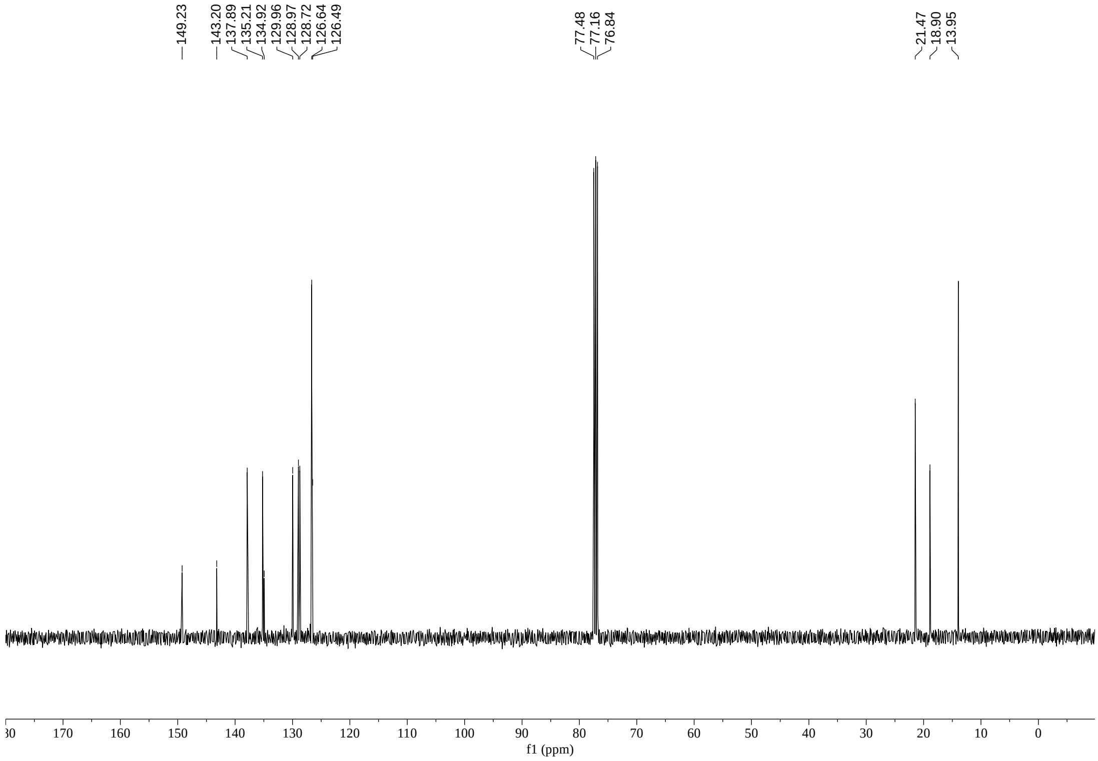

Supplementary Figure 47. ^{13}C NMR spectrum of **1h**

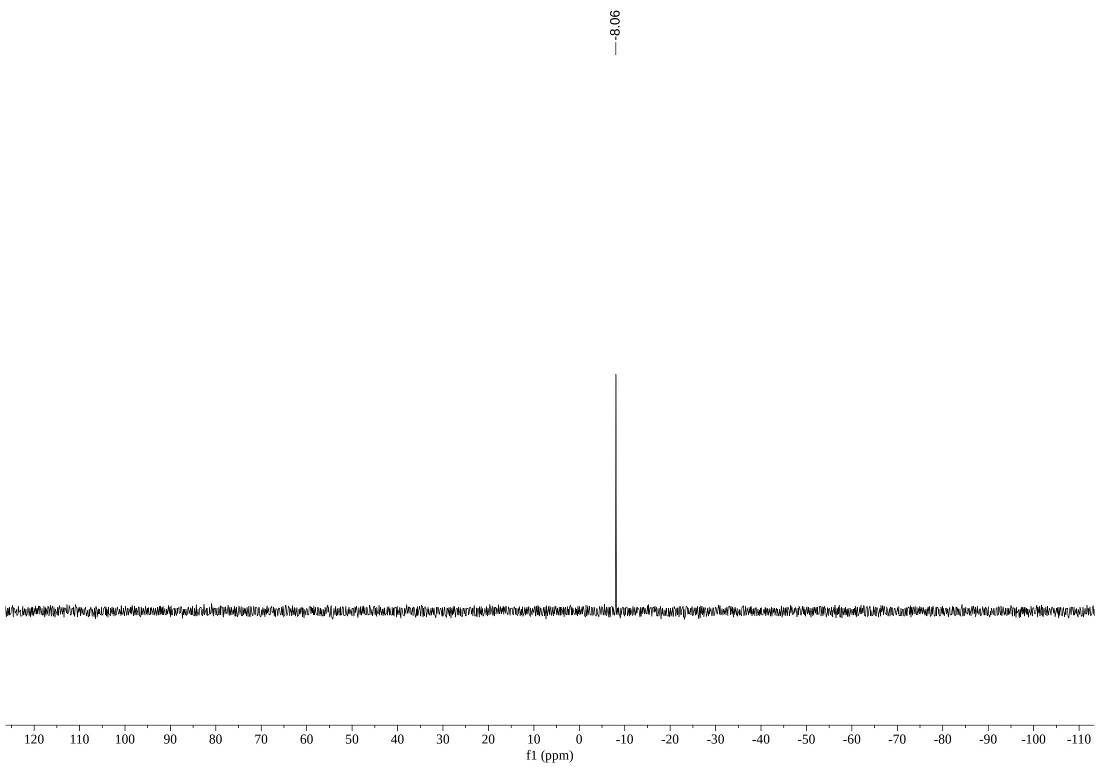

Supplementary Figure 48. ^{29}Si NMR spectrum of **1h**

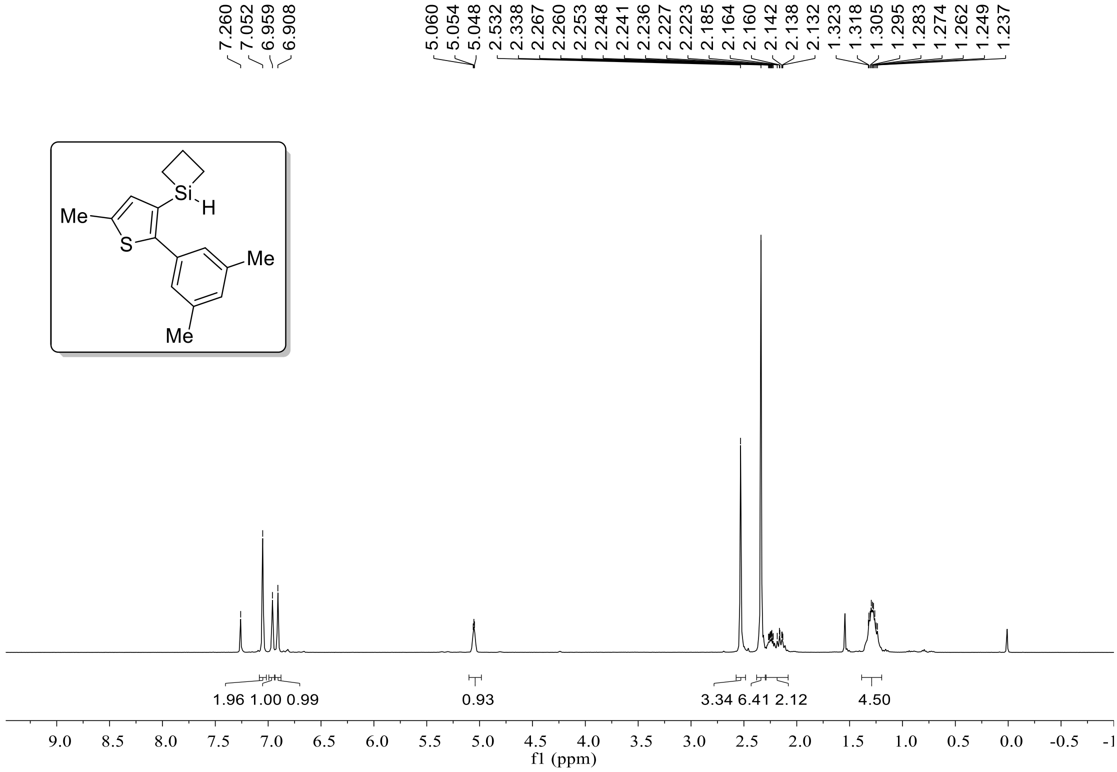

Supplementary Figure 49. ^{1}H NMR spectrum of **1i**

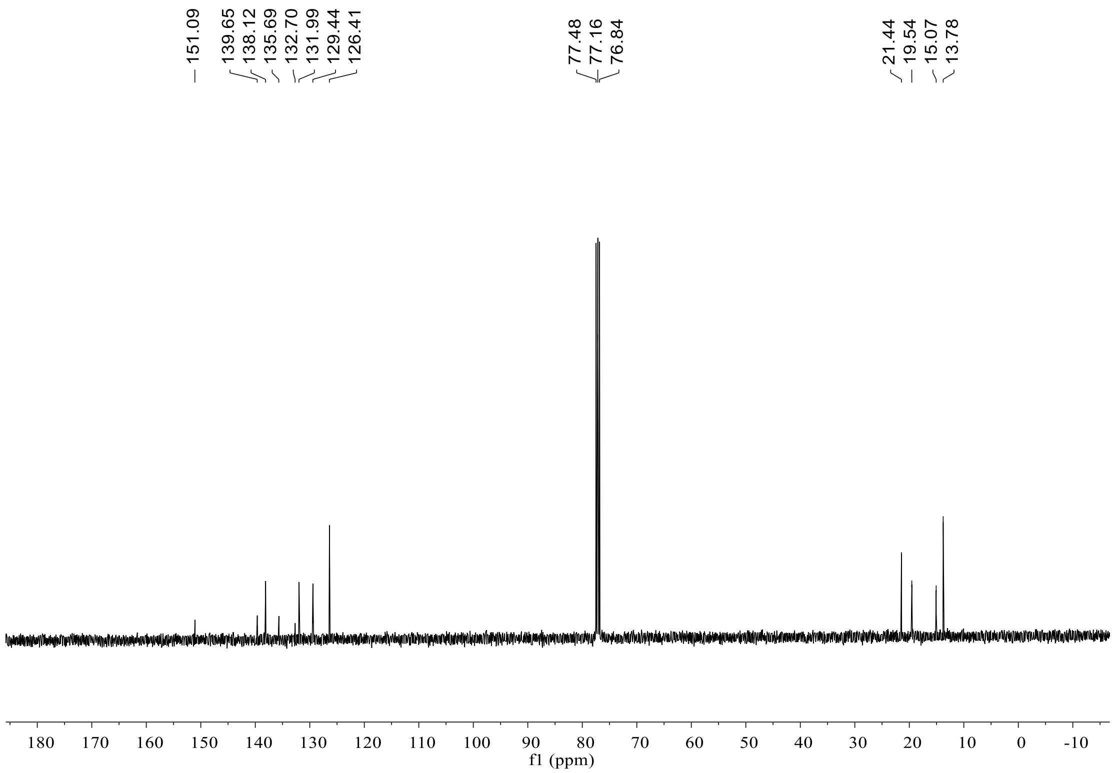

Supplementary Figure 50. ^{13}C NMR spectrum of **1i**

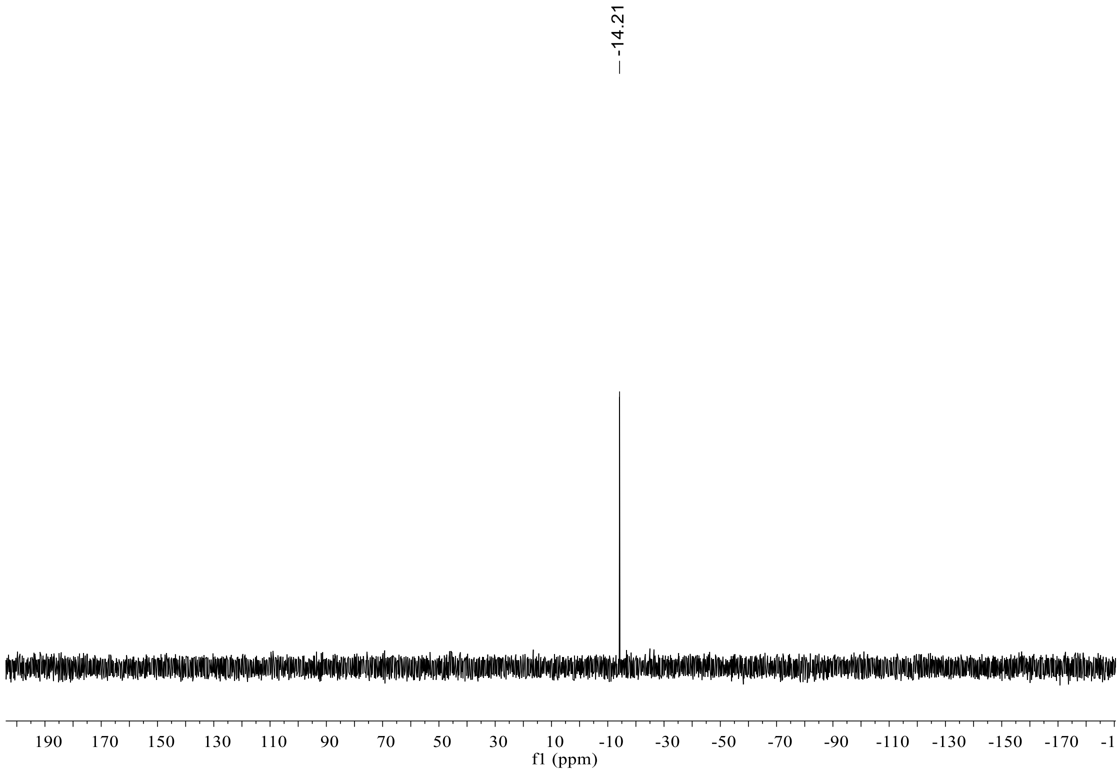

Supplementary Figure 51. ^{29}Si NMR spectrum of **1i**

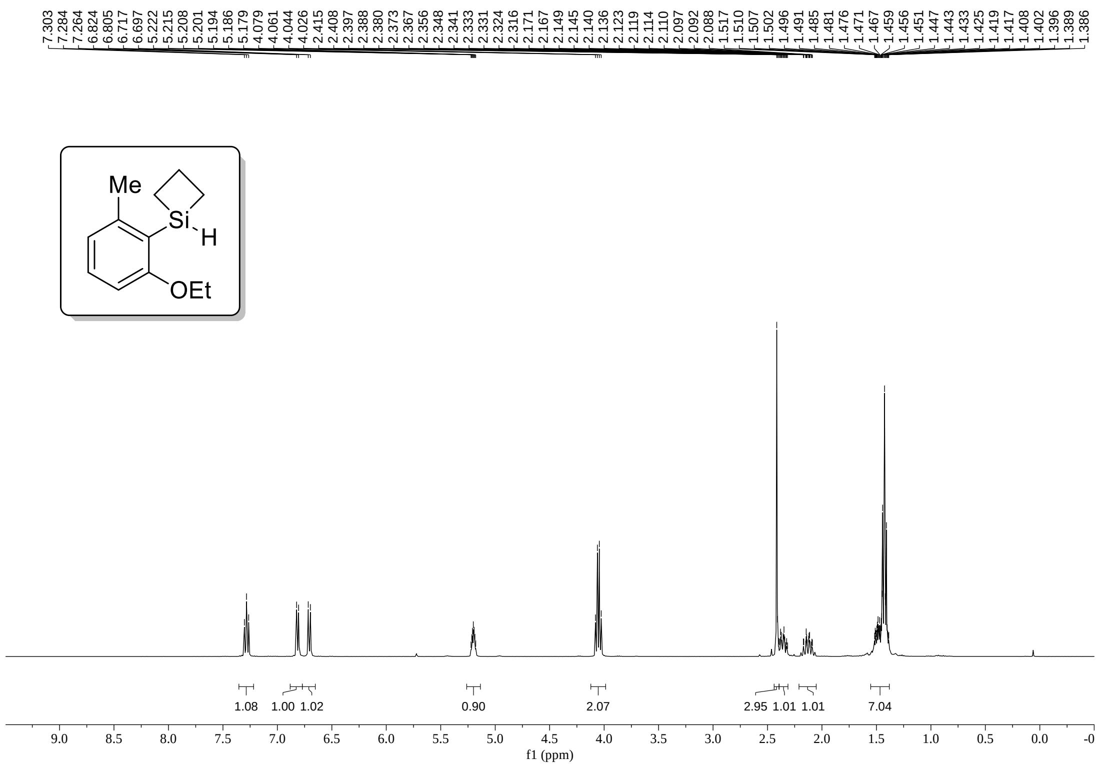

Supplementary Figure 52. ^{1}H NMR spectrum of **1j**

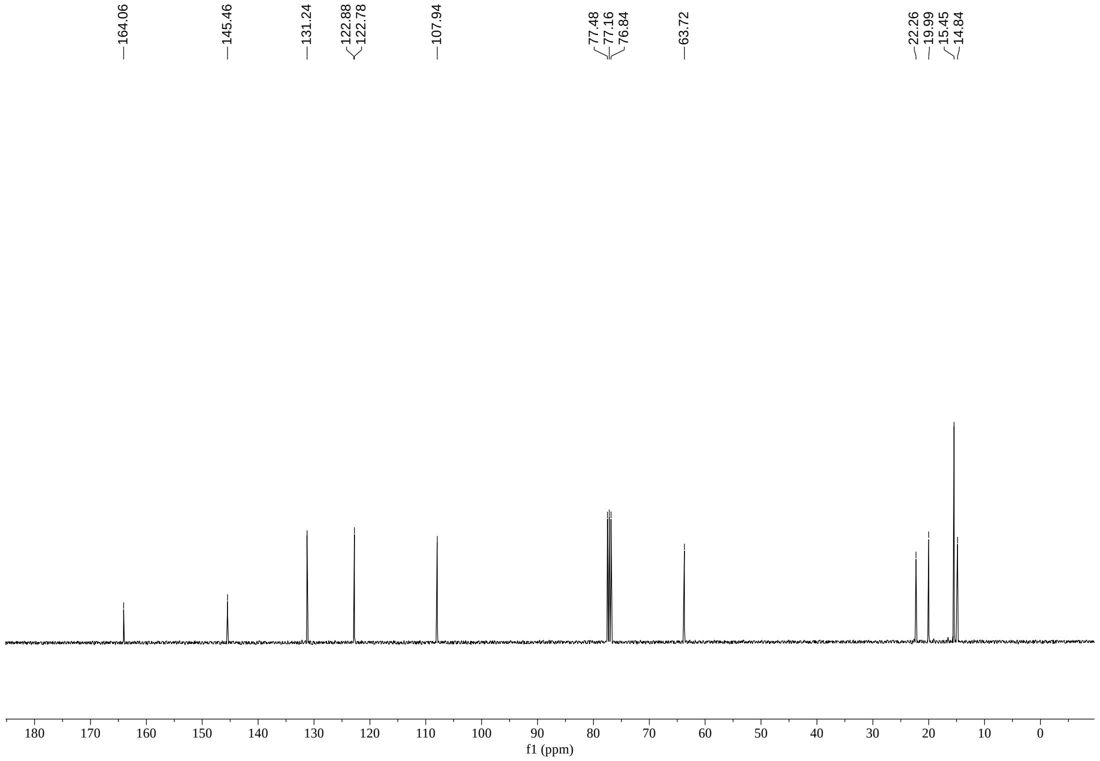

Supplementary Figure 53. ^{13}C NMR spectrum of **1j**

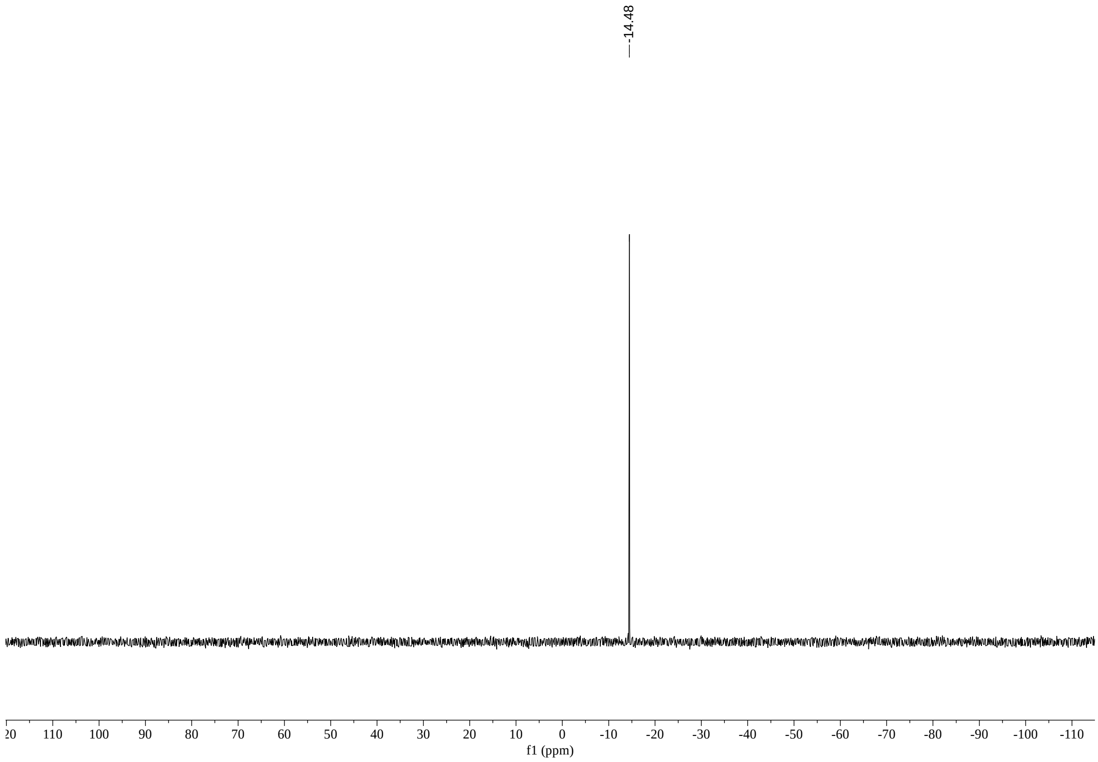

Supplementary Figure 54. ^{29}Si NMR spectrum of **1j**

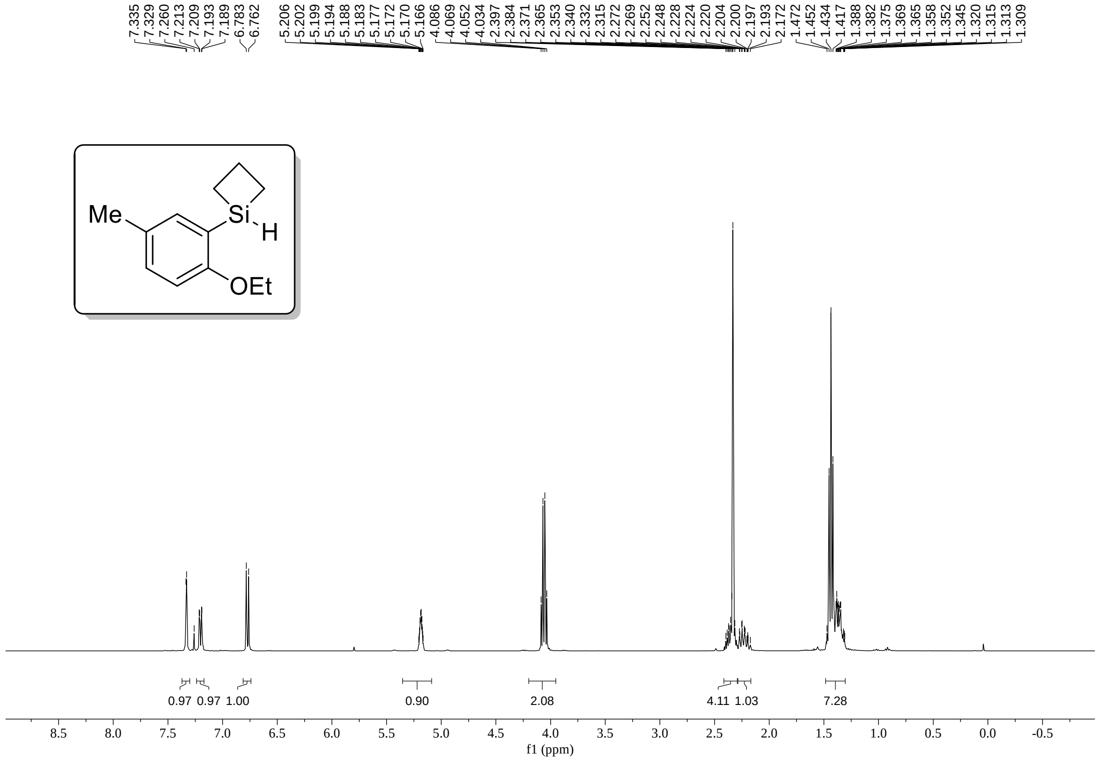

Supplementary Figure 55. ^{1}H NMR spectrum of **1k**

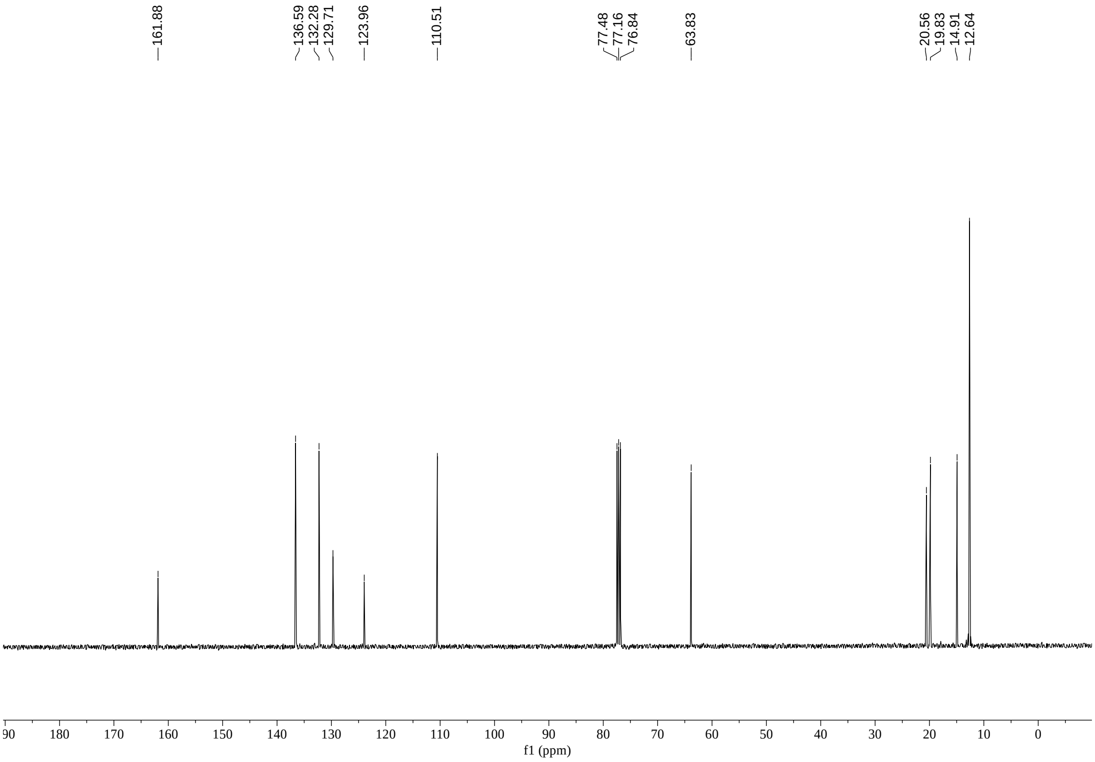

Supplementary Figure 56. ^{13}C NMR spectrum of **1k**

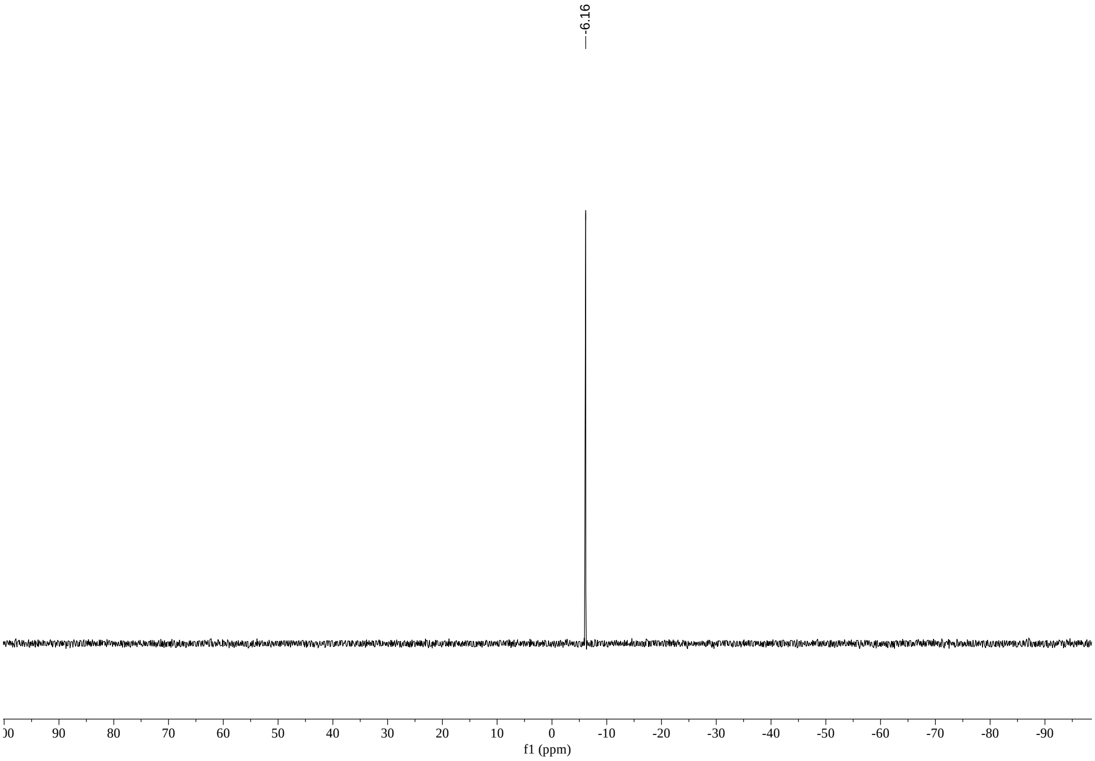

Supplementary Figure 57. ^{29}Si NMR spectrum of **1k**

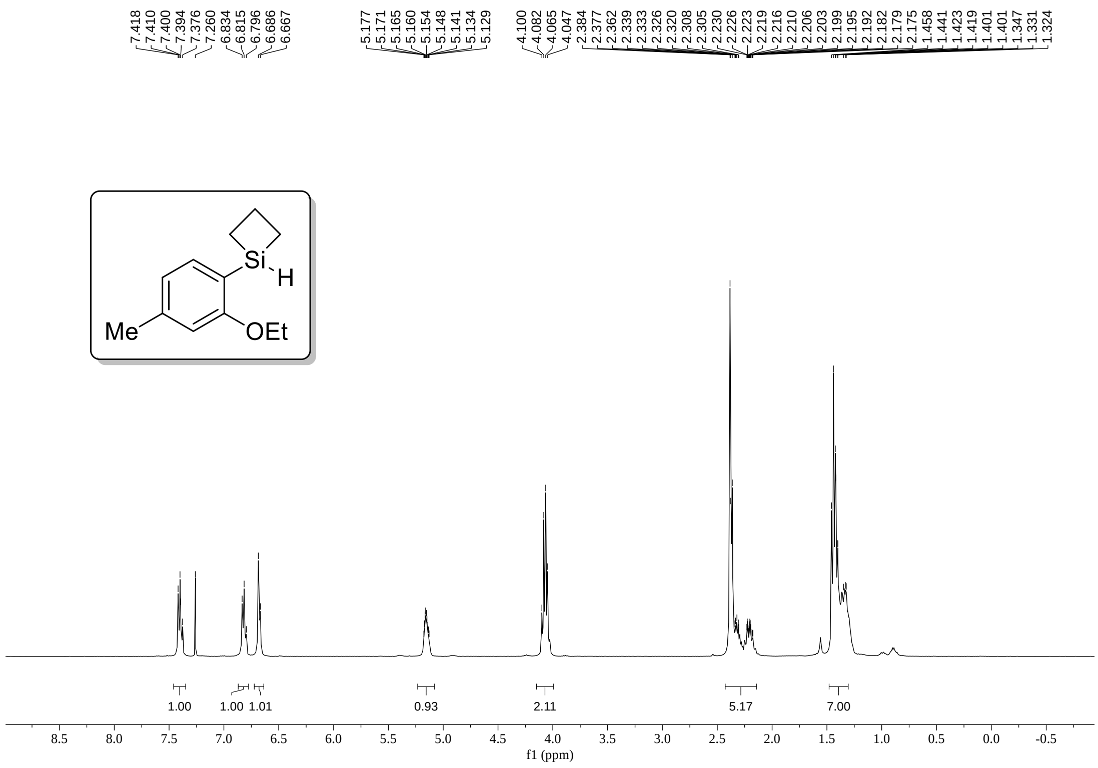

Supplementary Figure 58. ^{1}H NMR spectrum of **1l**

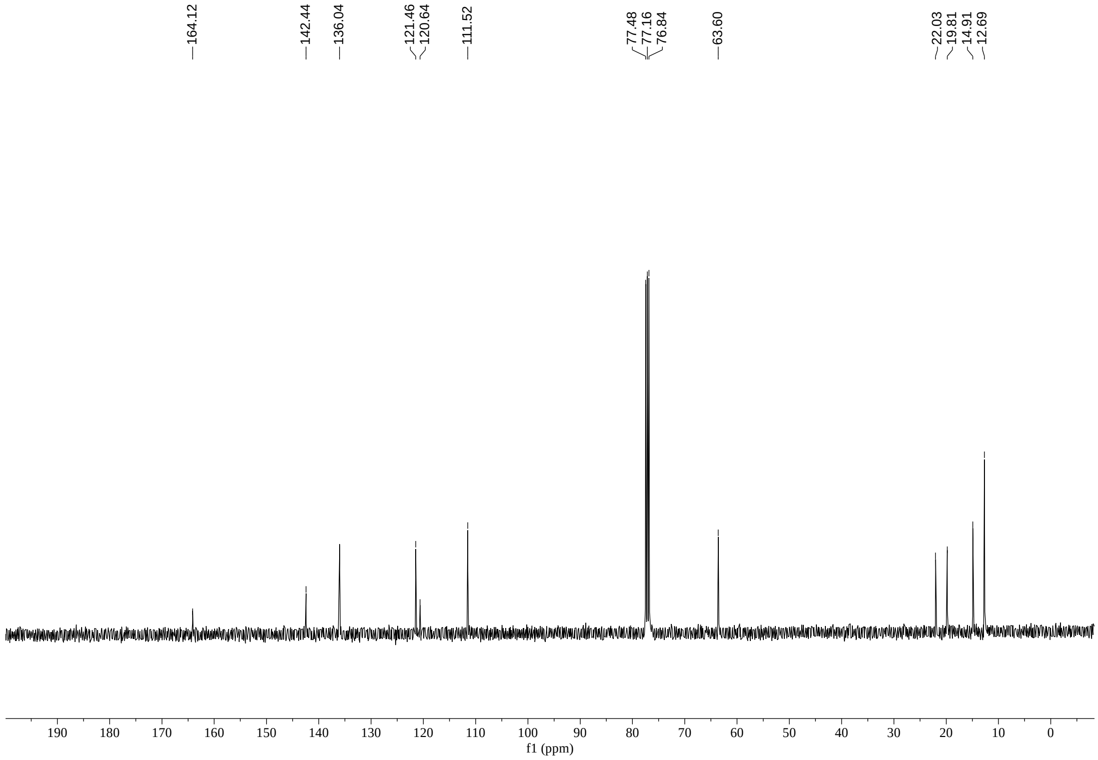

Supplementary Figure 59. ^{13}C NMR spectrum of **1l**

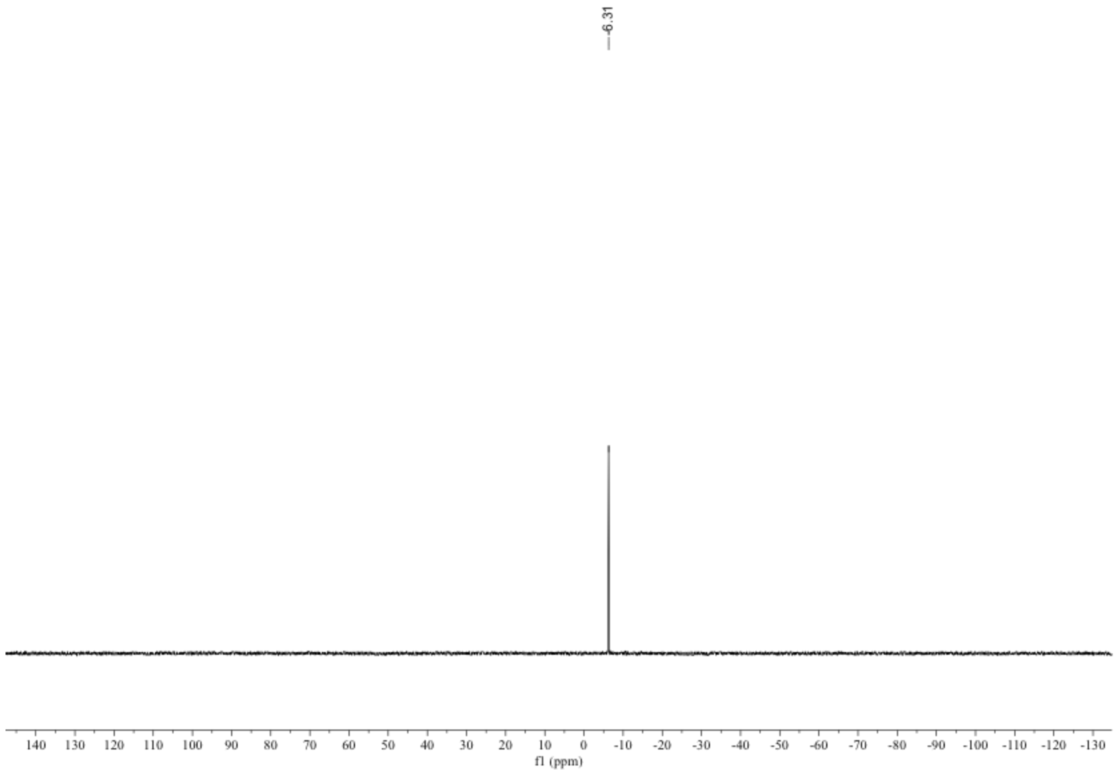

Supplementary Figure 60. ^{29}Si NMR spectrum of **1l**

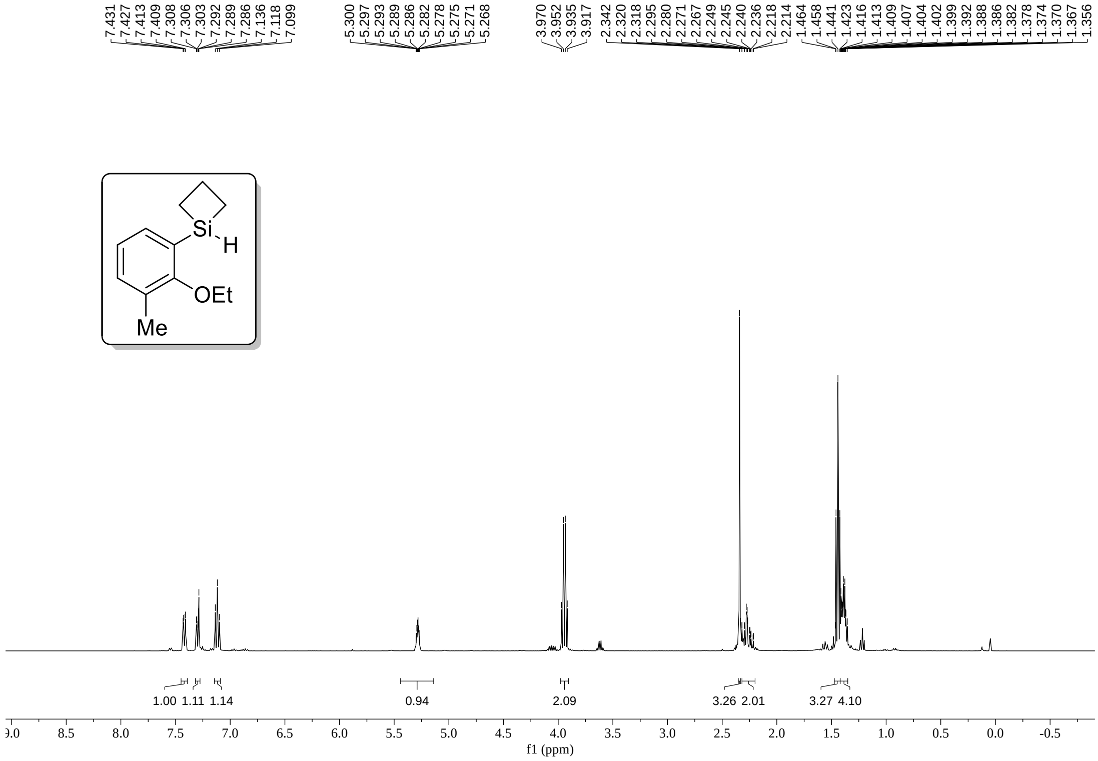

Supplementary Figure 61. ^{1}H NMR spectrum of **1m**

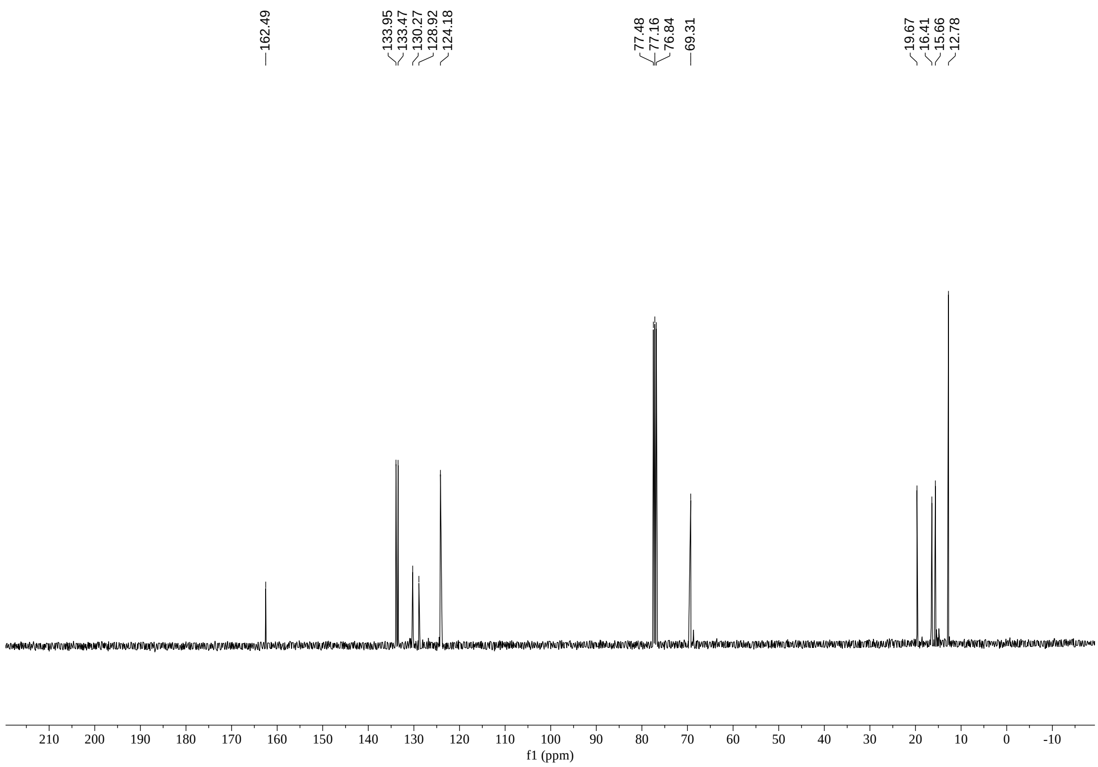

Supplementary Figure 62. ^{13}C NMR spectrum of **1m**

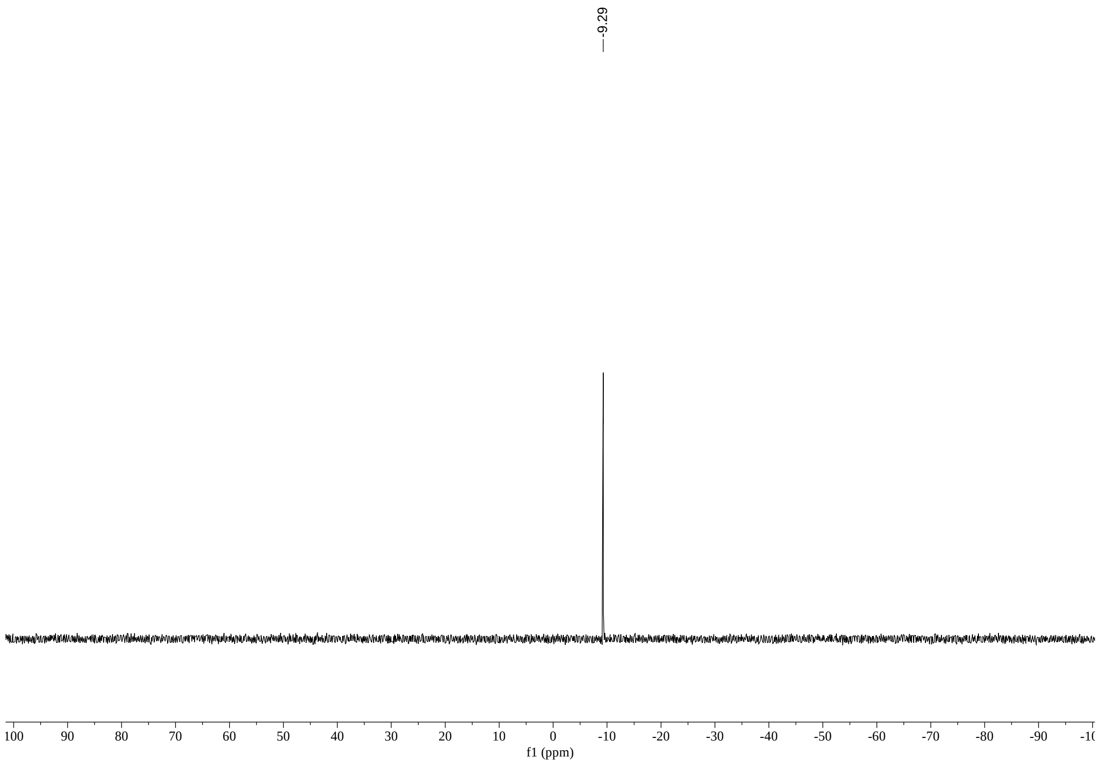

Supplementary Figure 63. ^{29}Si NMR spectrum of **1m**

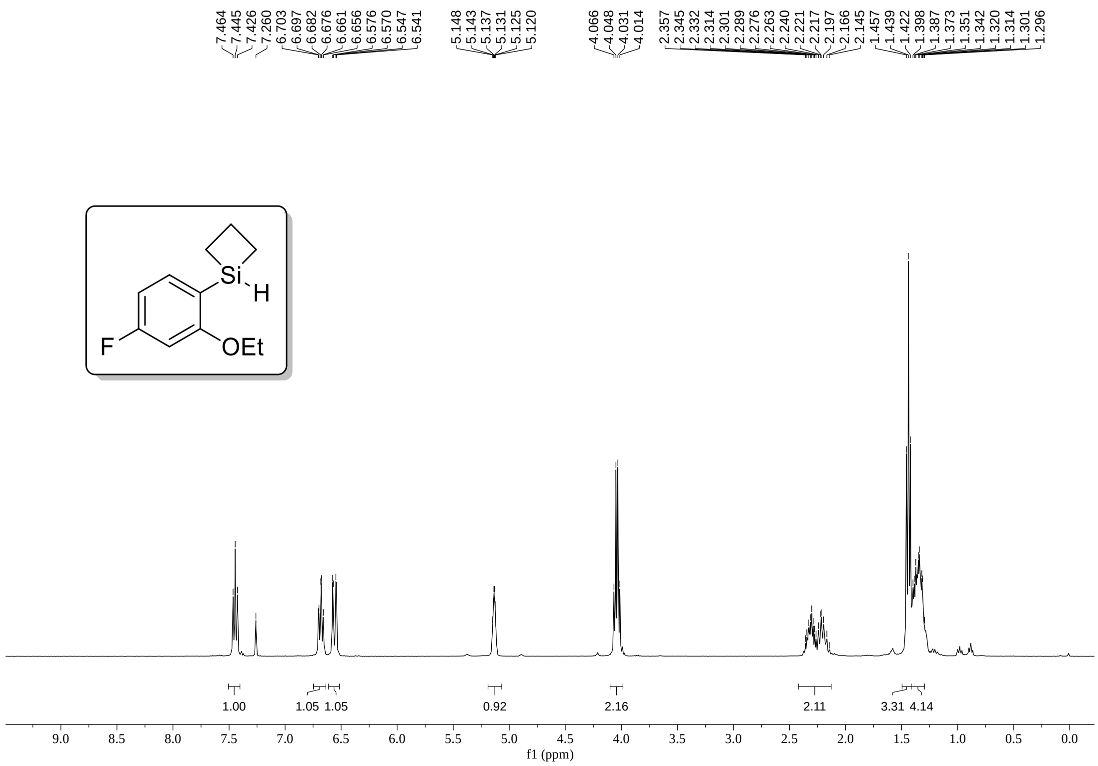

Supplementary Figure 64. ^{1}H NMR spectrum of **1n**

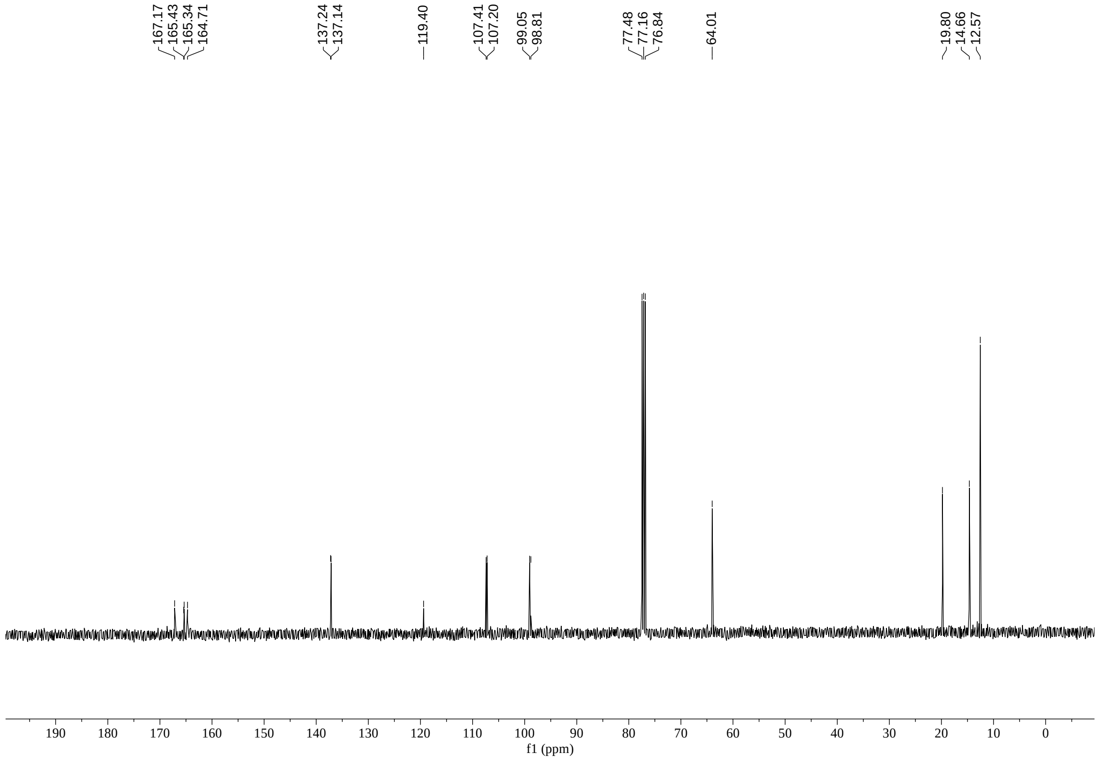

Supplementary Figure 65. ^{13}C NMR spectrum of **1n**

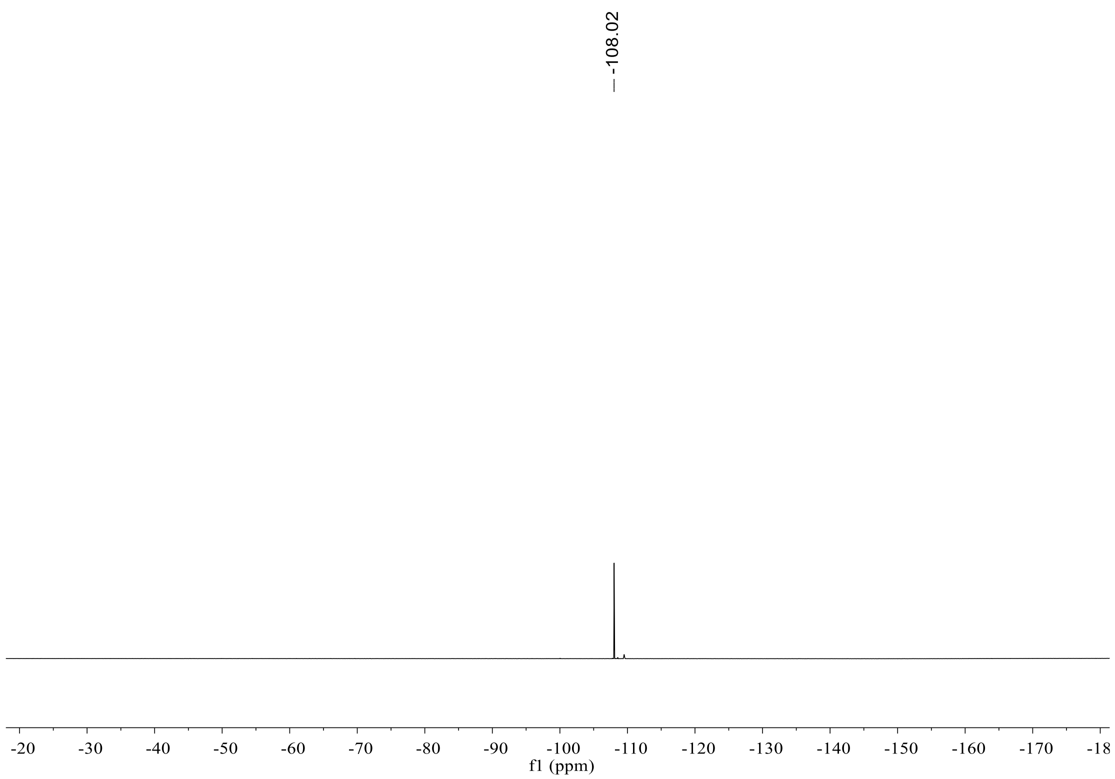

Supplementary Figure 66. ^{19}F NMR spectrum of **1n**

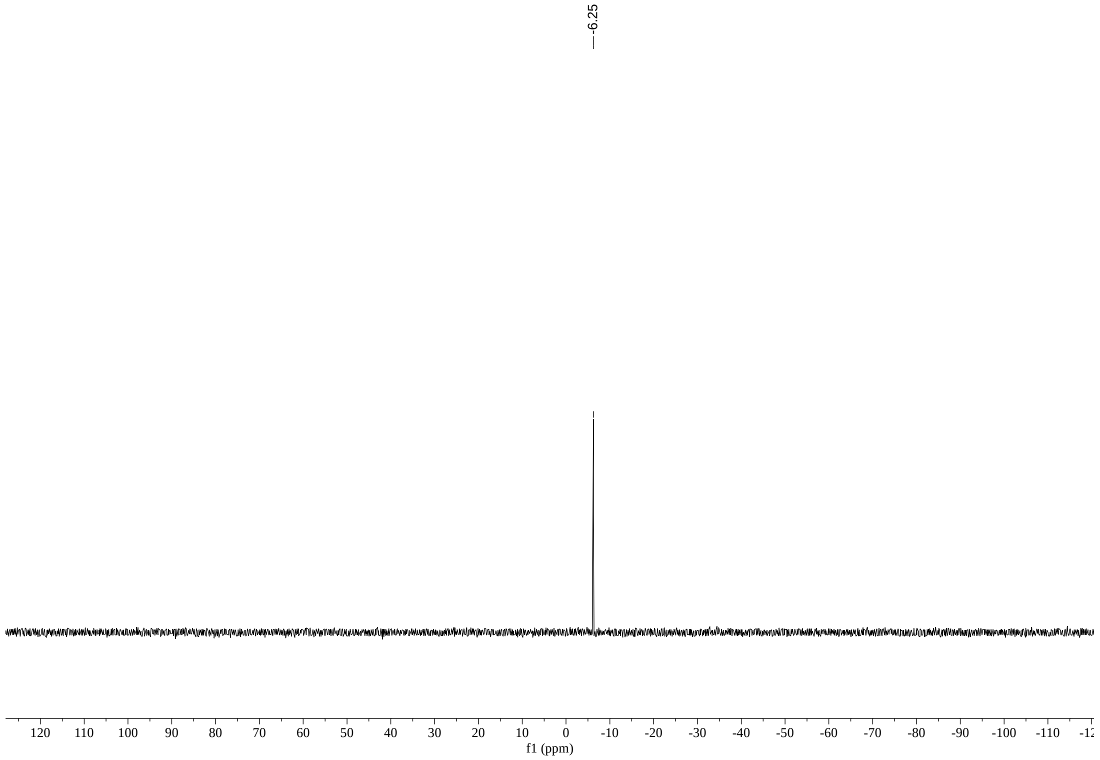

Supplementary Figure 67. ^{29}Si NMR spectrum of **1n**

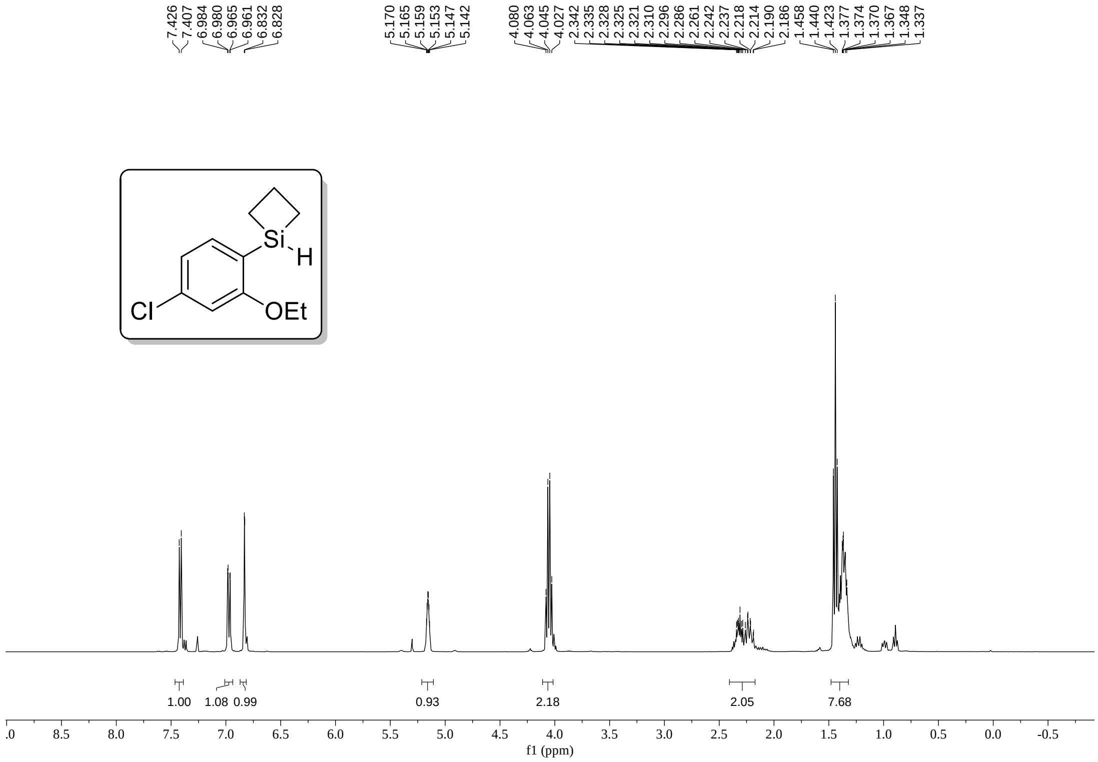

Supplementary Figure 68. ^{1}H NMR spectrum of **1o**

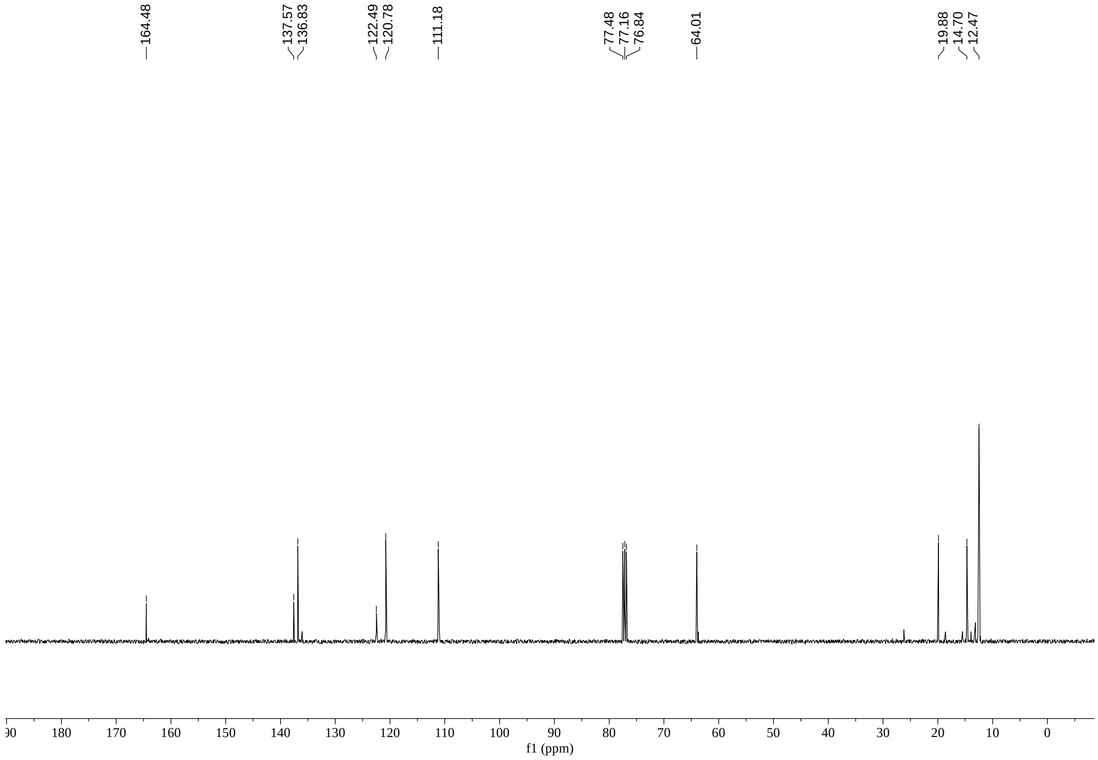

Supplementary Figure 69. ^{13}C NMR spectrum of **1o**

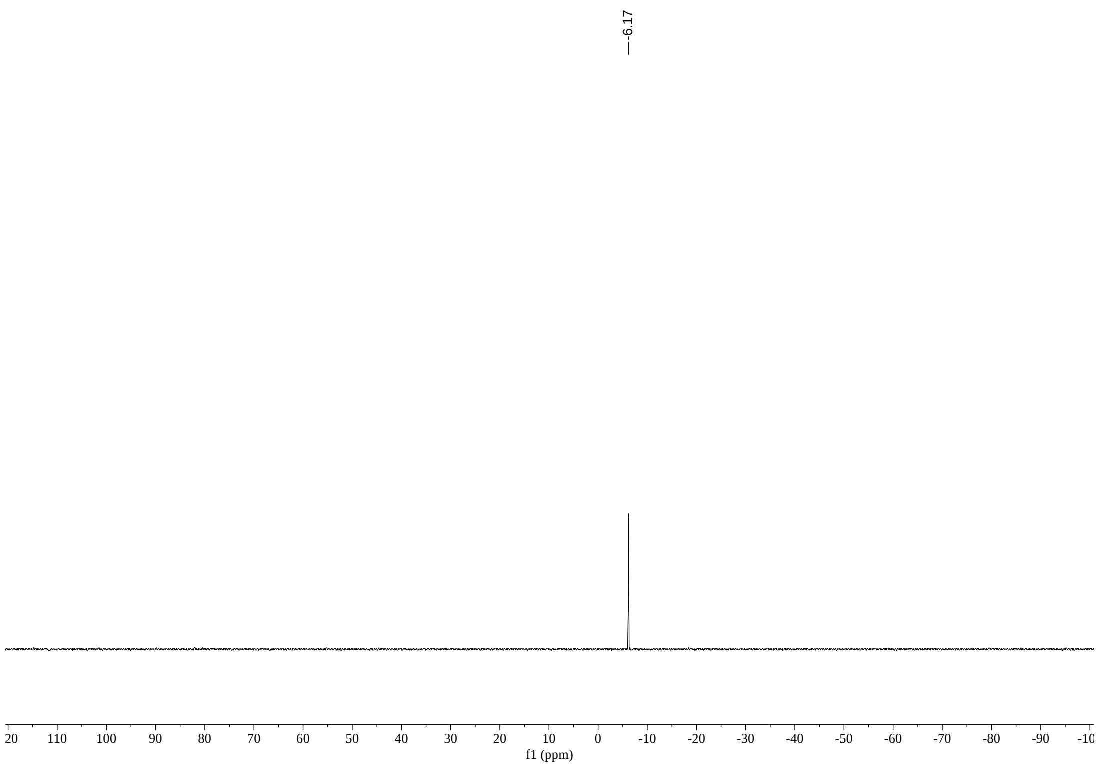

Supplementary Figure 70. ^{29}Si NMR spectrum of **1o**

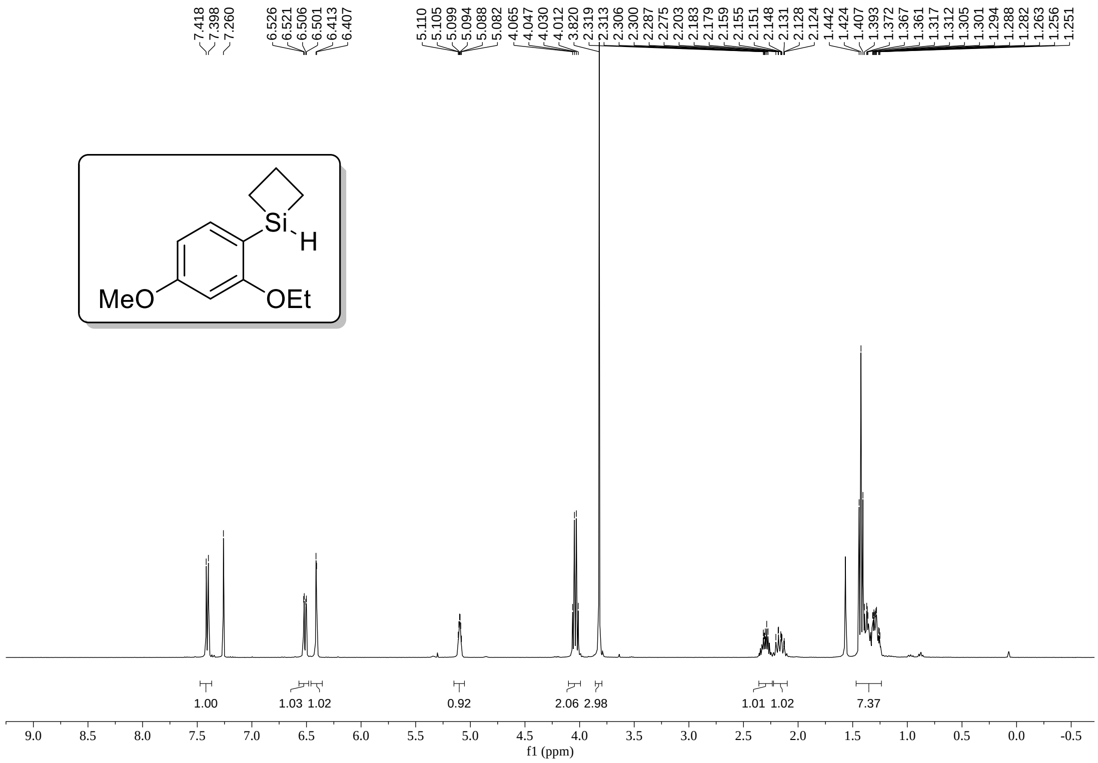

Supplementary Figure 71. ^{1}H NMR spectrum of **1p**

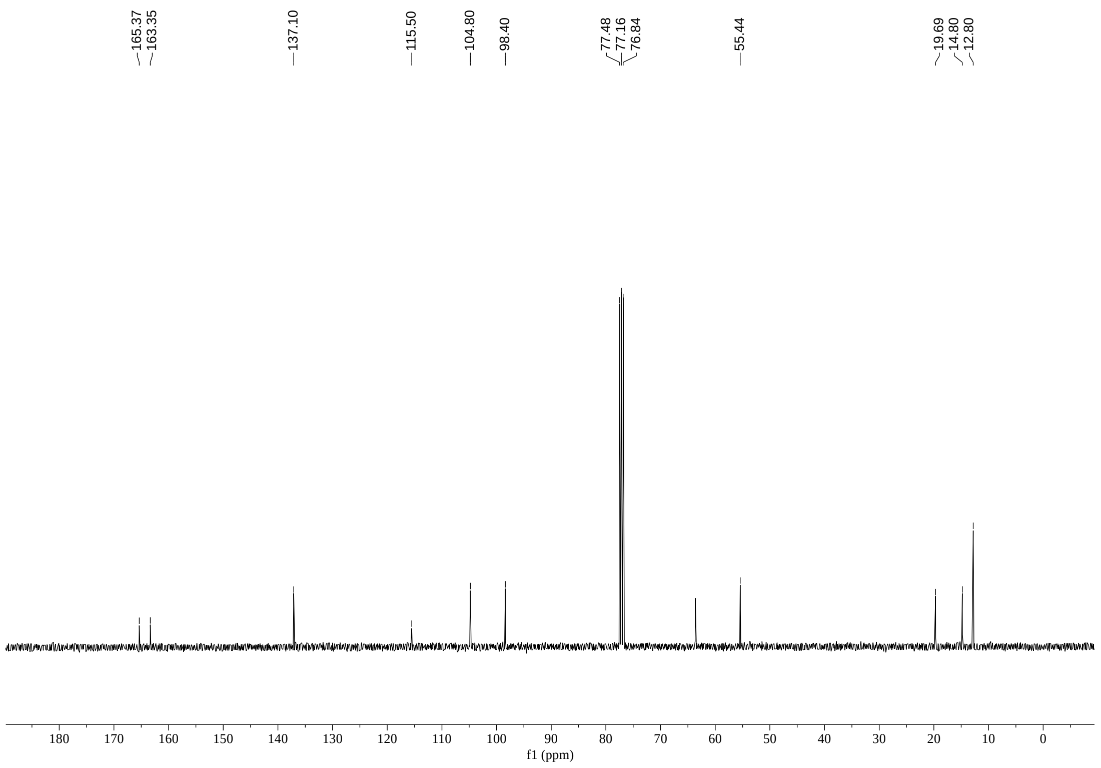

Supplementary Figure 72. ^{13}C NMR spectrum of **1p**

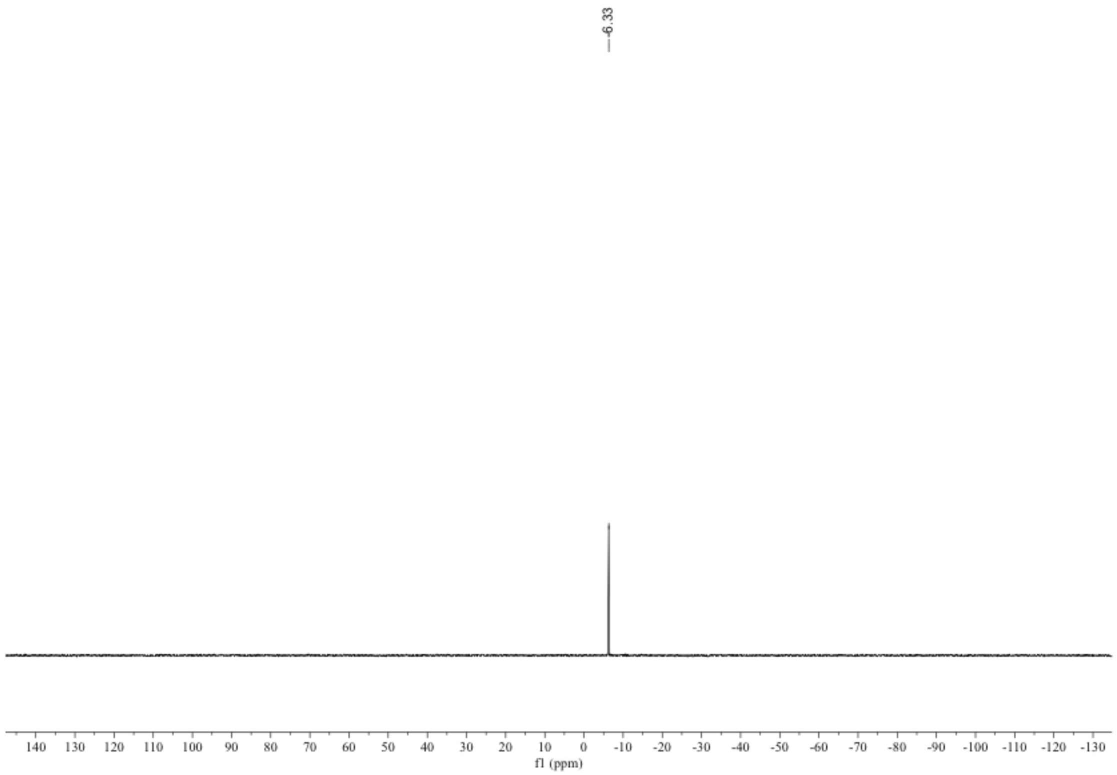

Supplementary Figure 73. ^{29}Si NMR spectrum of **1p**

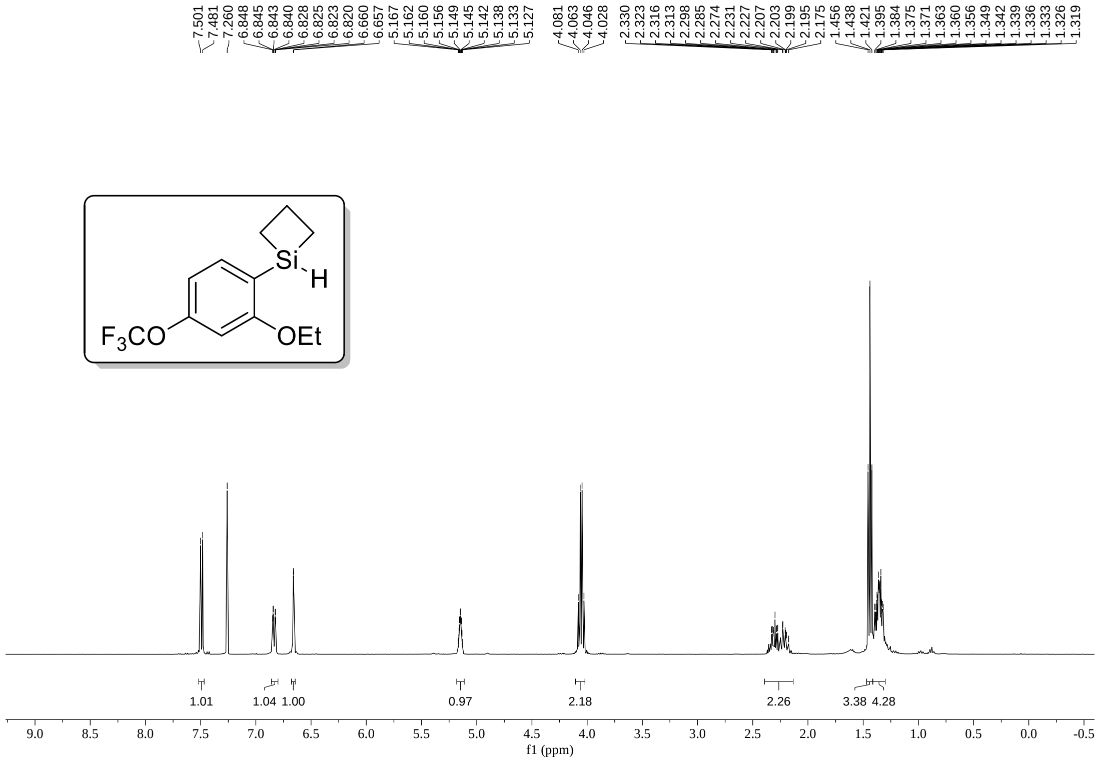

Supplementary Figure 74. ^{1}H NMR spectrum of **1q**

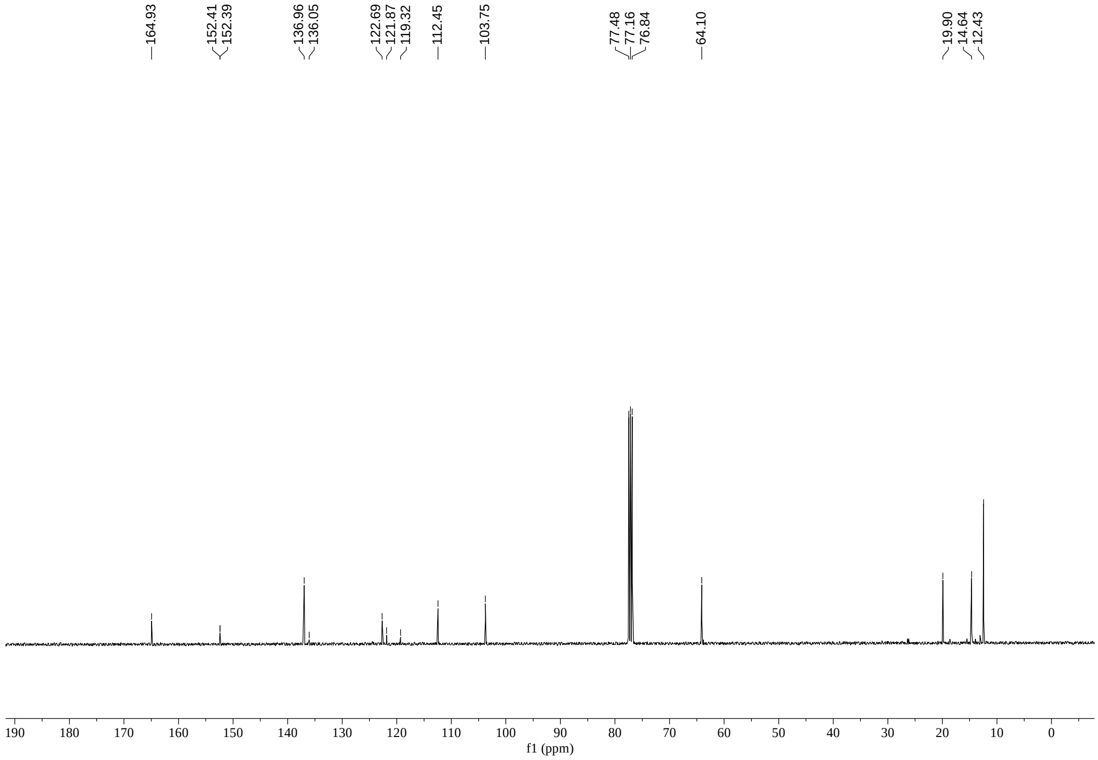

Supplementary Figure 75. ^{13}C NMR spectrum of **1q**

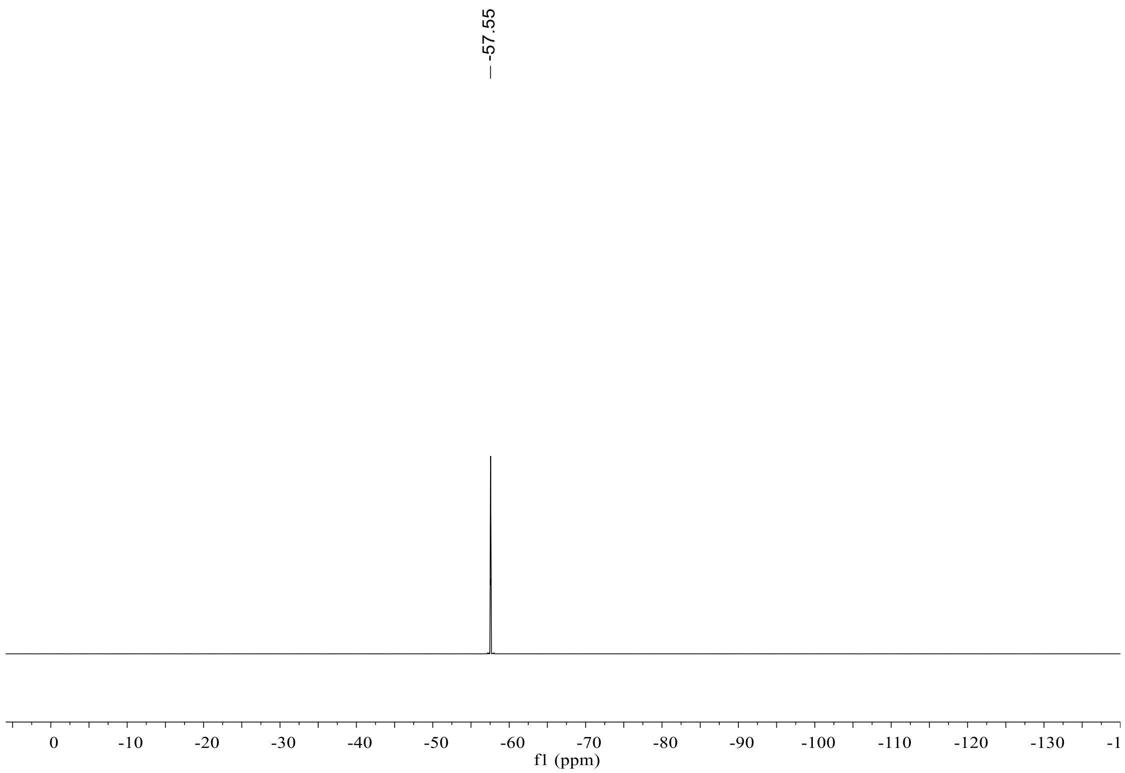

Supplementary Figure 76. ^{19}F NMR spectrum of **1q**

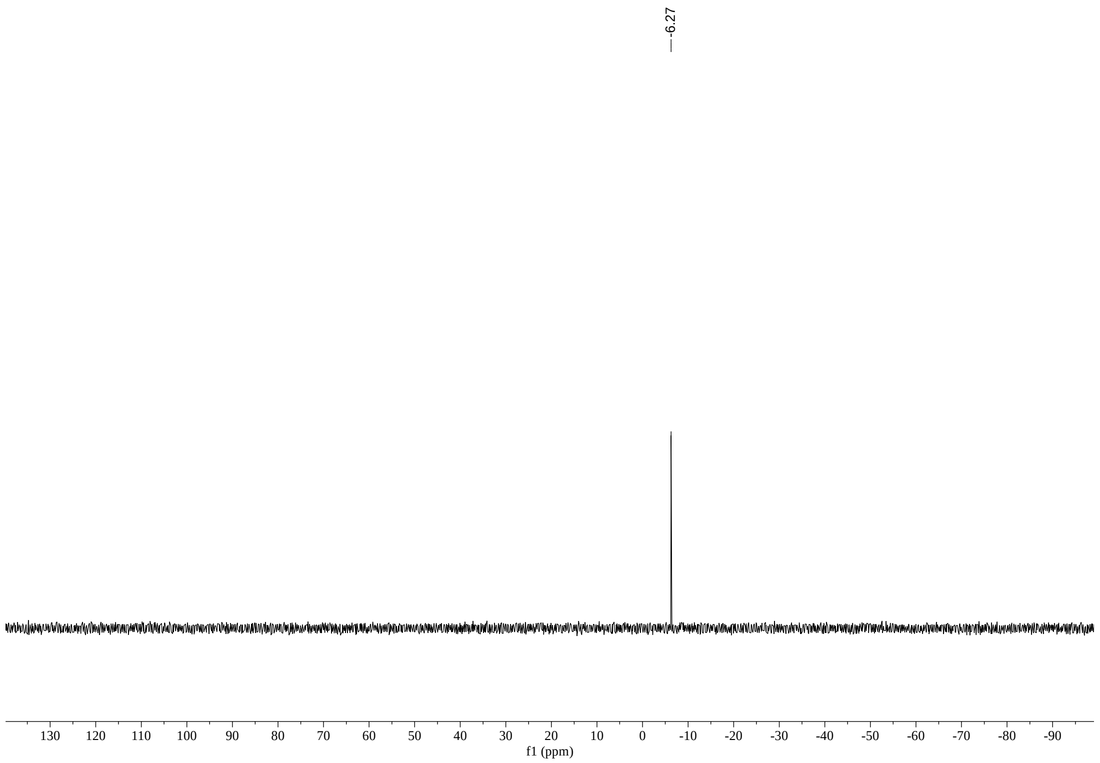

Supplementary Figure 77. ^{29}Si NMR spectrum of **1q**

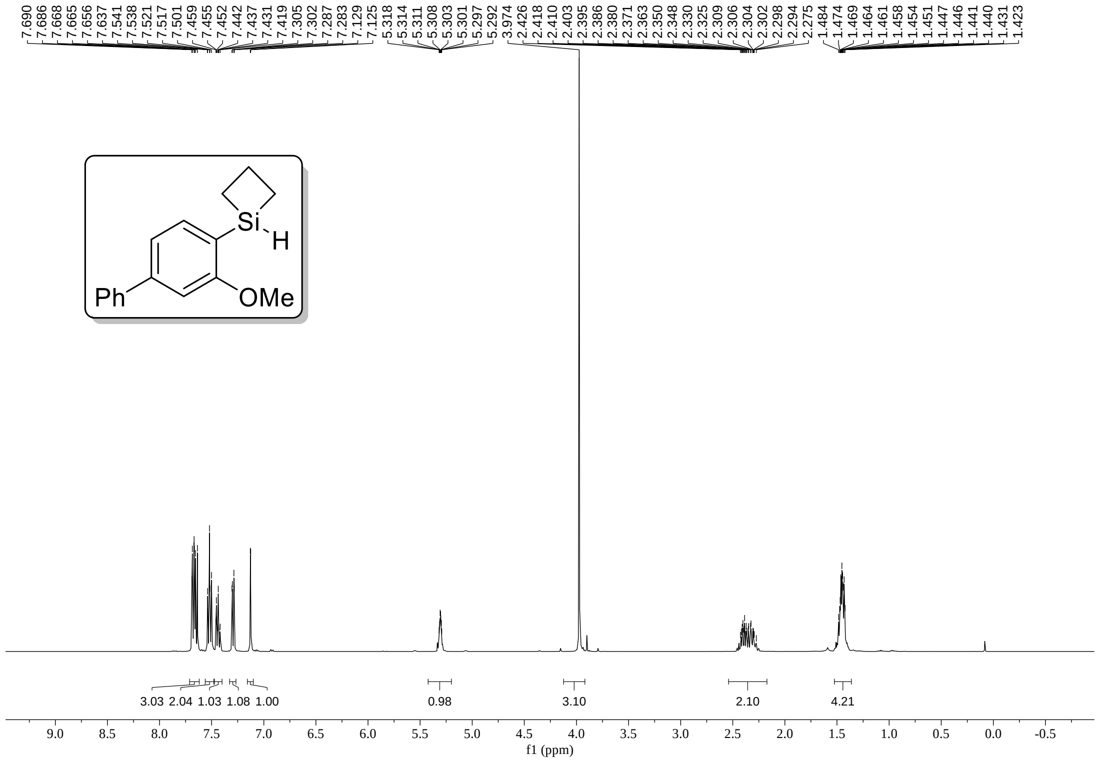

Supplementary Figure 78. ^{1}H NMR spectrum of **1r**

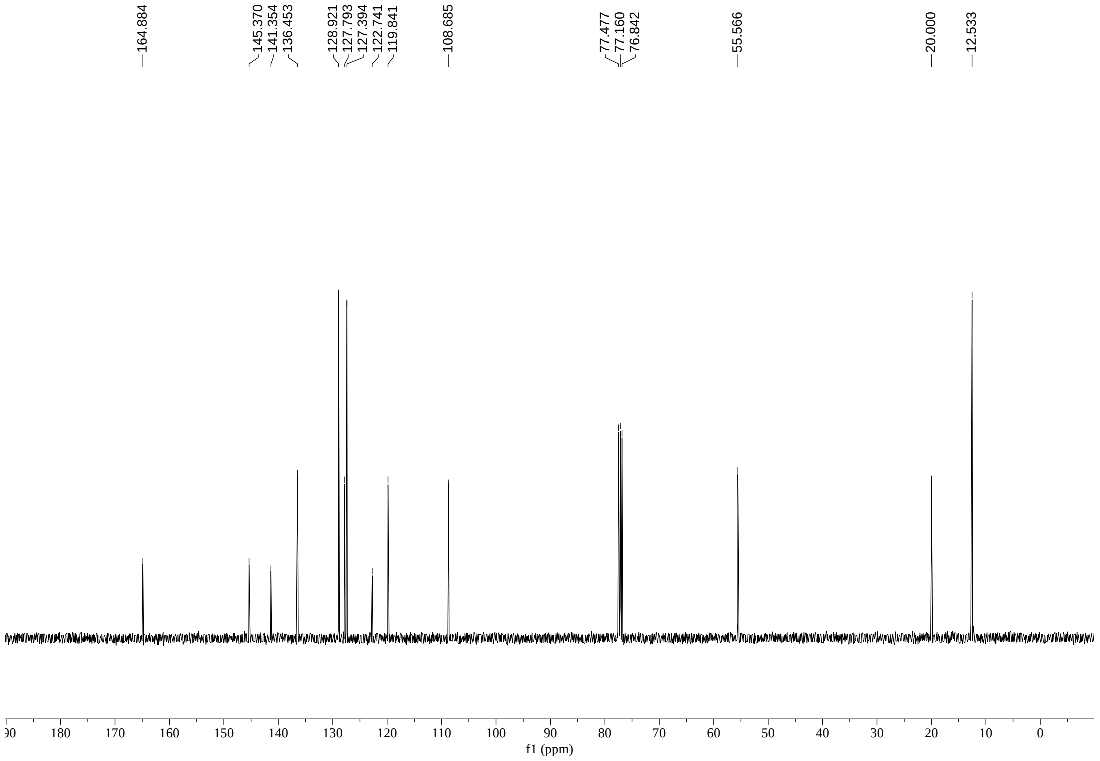

Supplementary Figure 79. ^{13}C NMR spectrum of **1r**

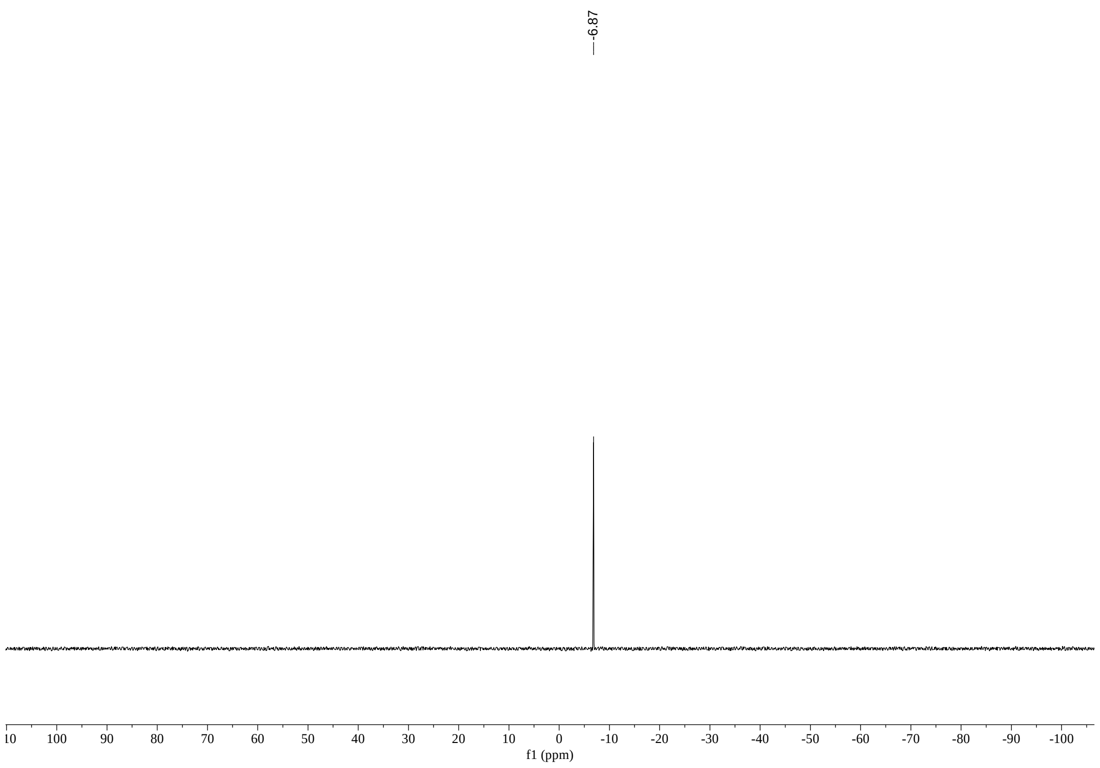

Supplementary Figure 80. ^{29}Si NMR spectrum of **1r**

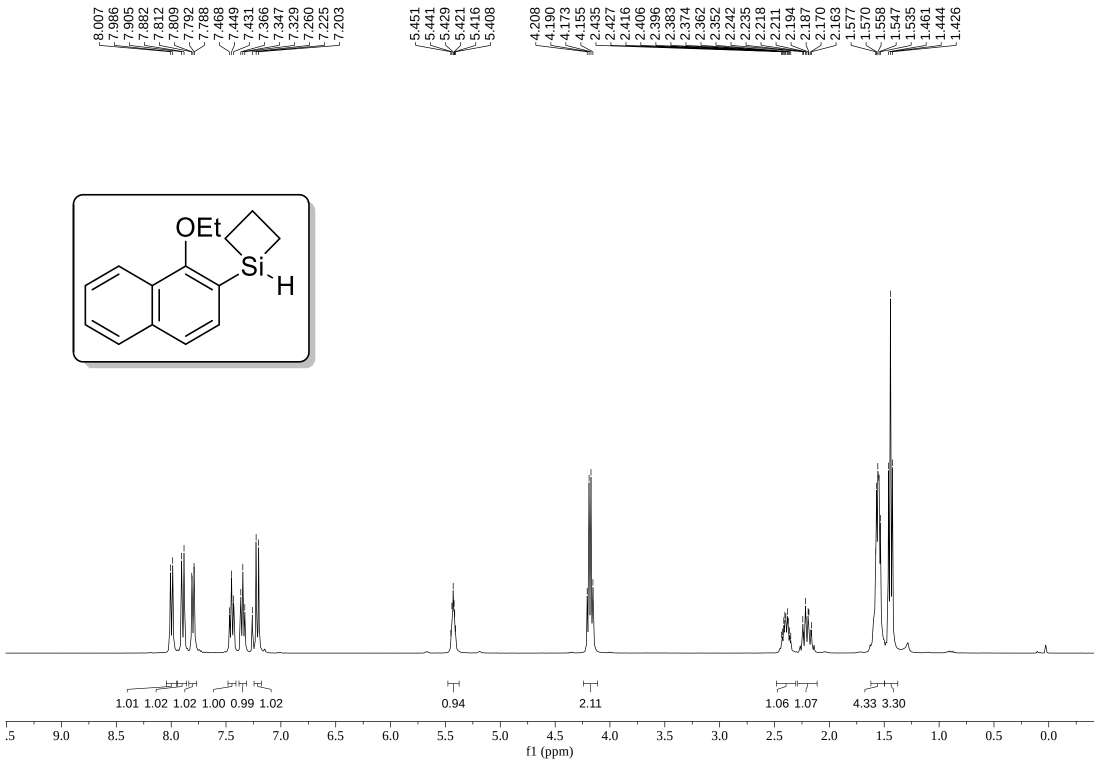

Supplementary Figure 81. ^{1}H NMR spectrum of **1s**

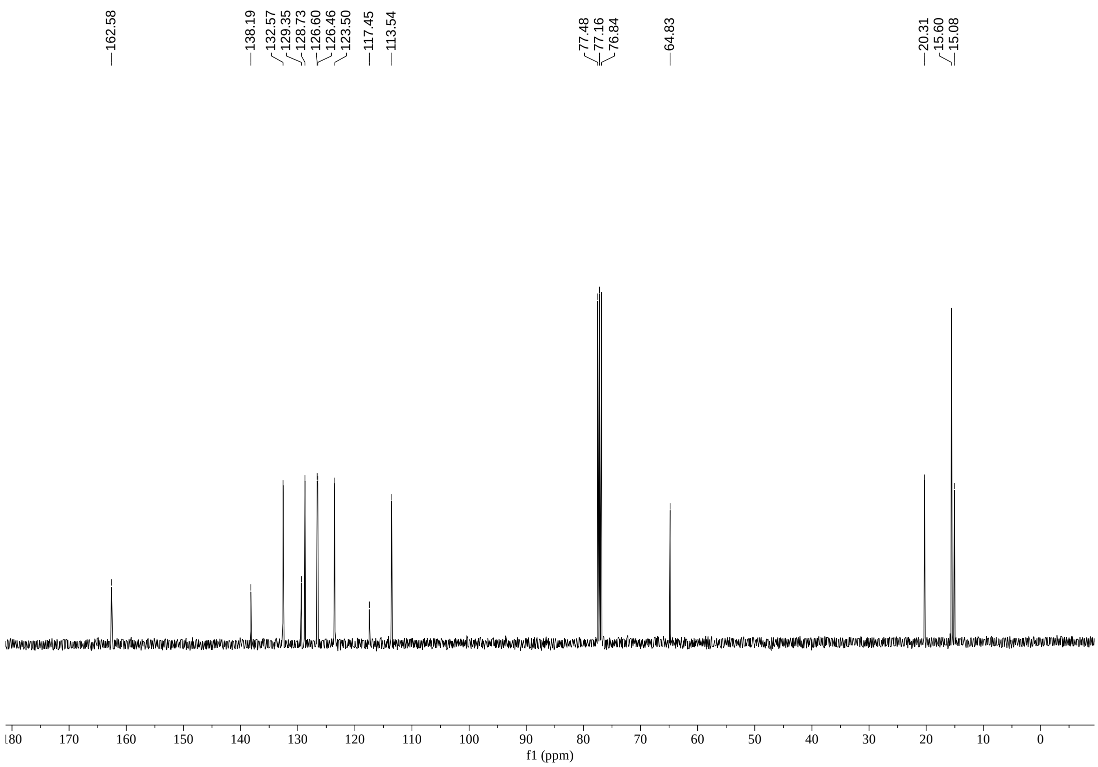

Supplementary Figure 82. ^{13}C NMR spectrum of **1s**

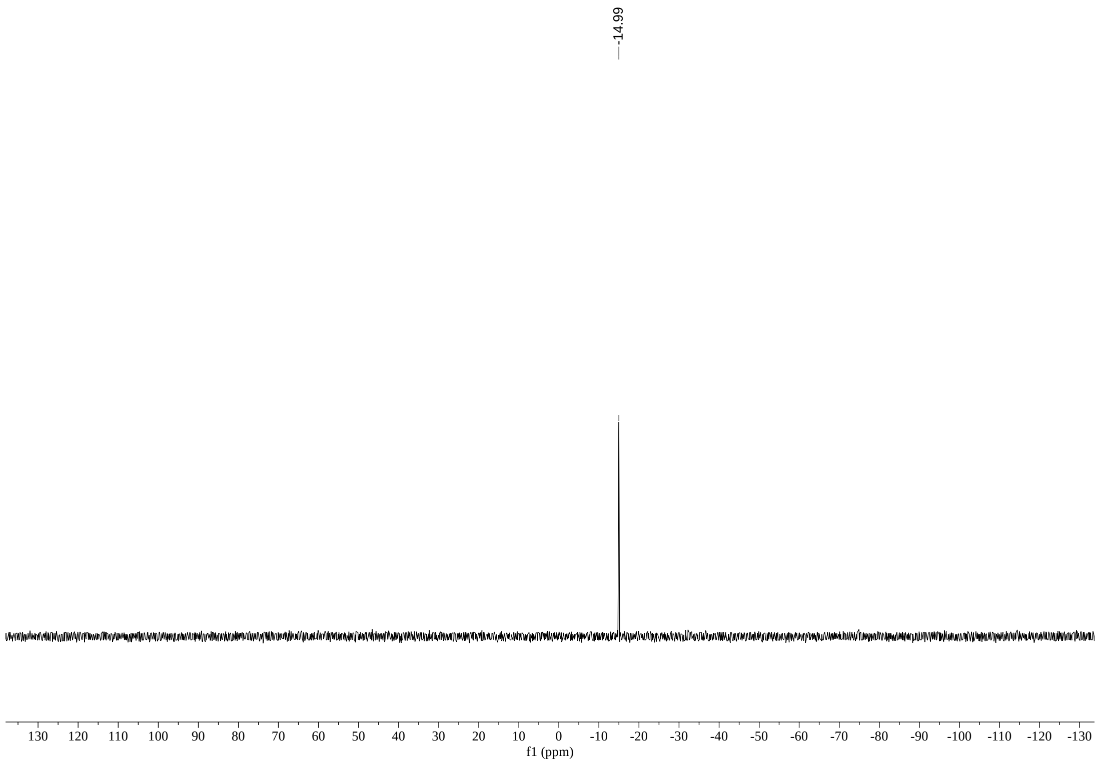

Supplementary Figure 83. ^{29}Si NMR spectrum of **1s**

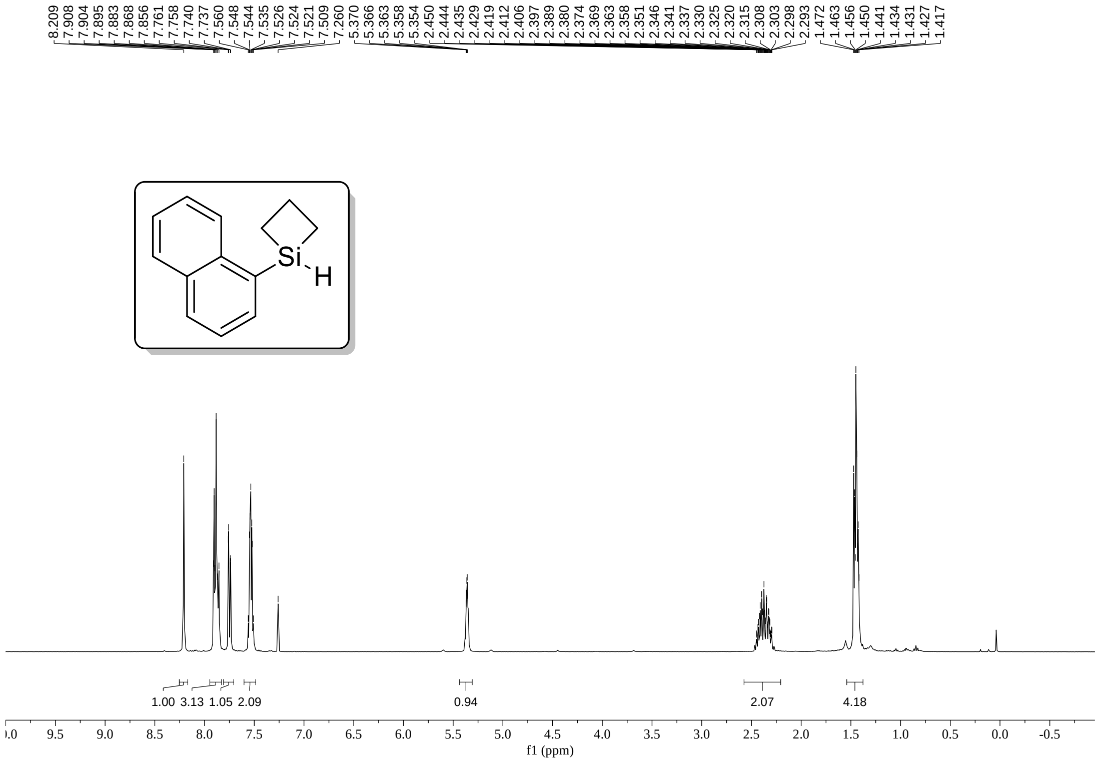

Supplementary Figure 84. ^{1}H NMR spectrum of **1t**

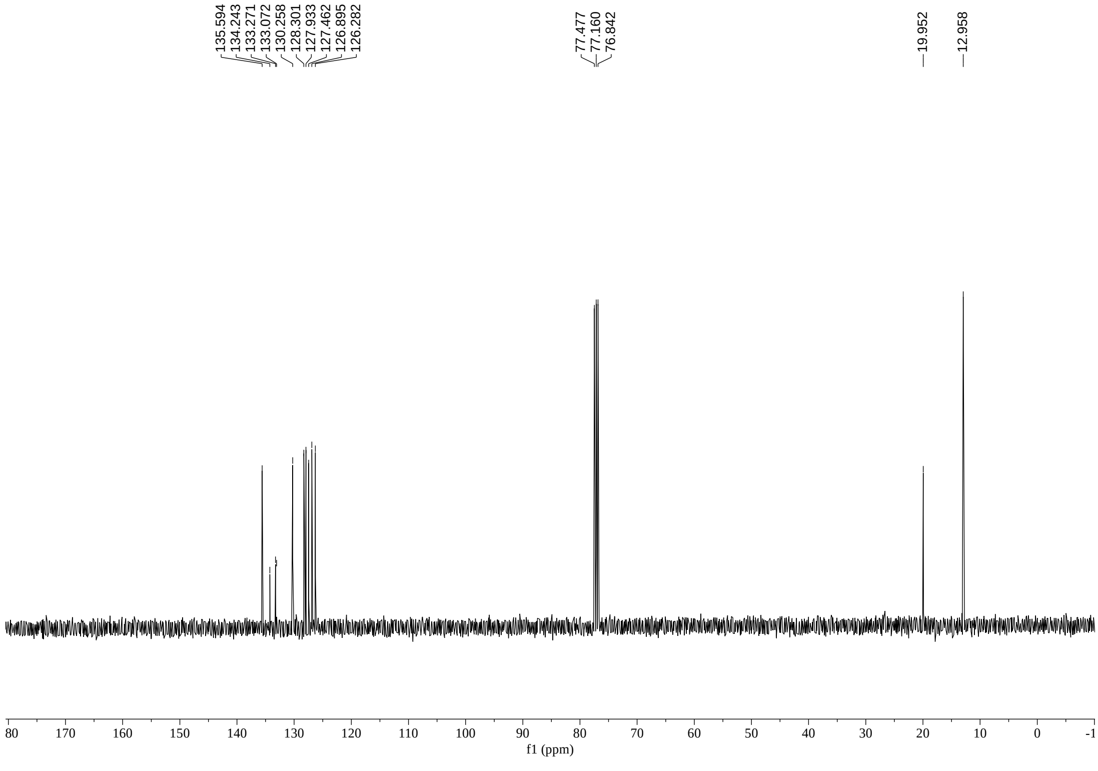

Supplementary Figure 85. ^{13}C NMR spectrum of **1t**

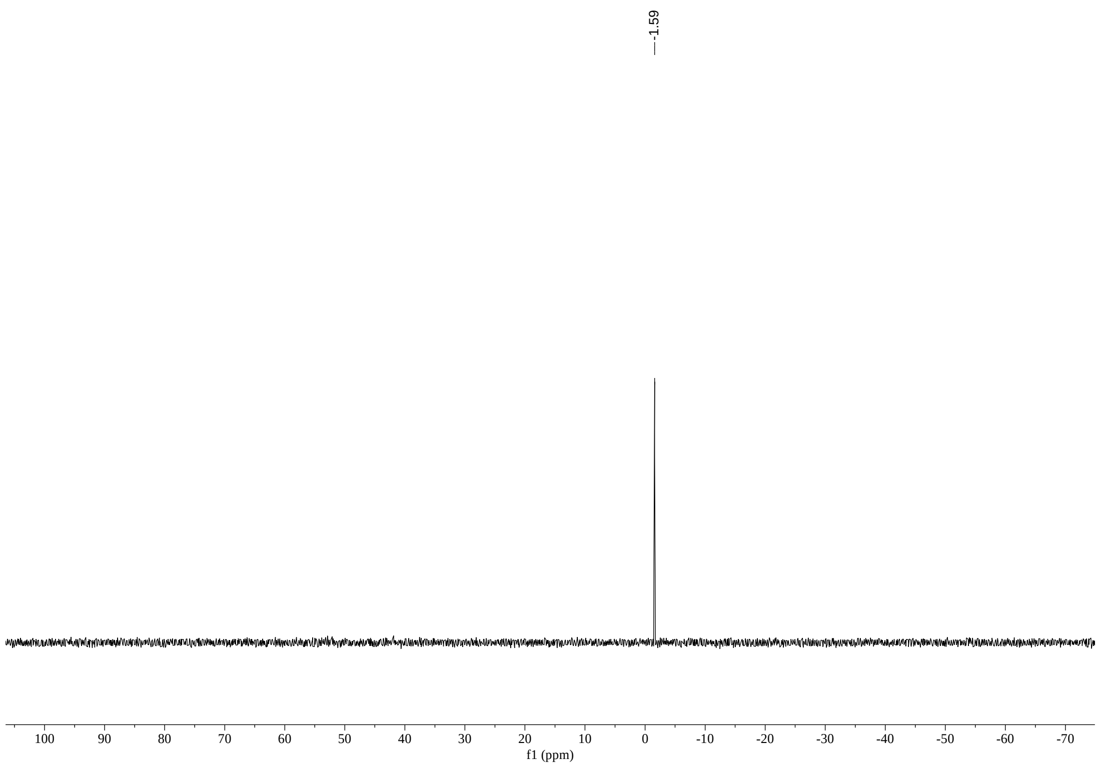

Supplementary Figure 86. ^{29}Si NMR spectrum of **1t**

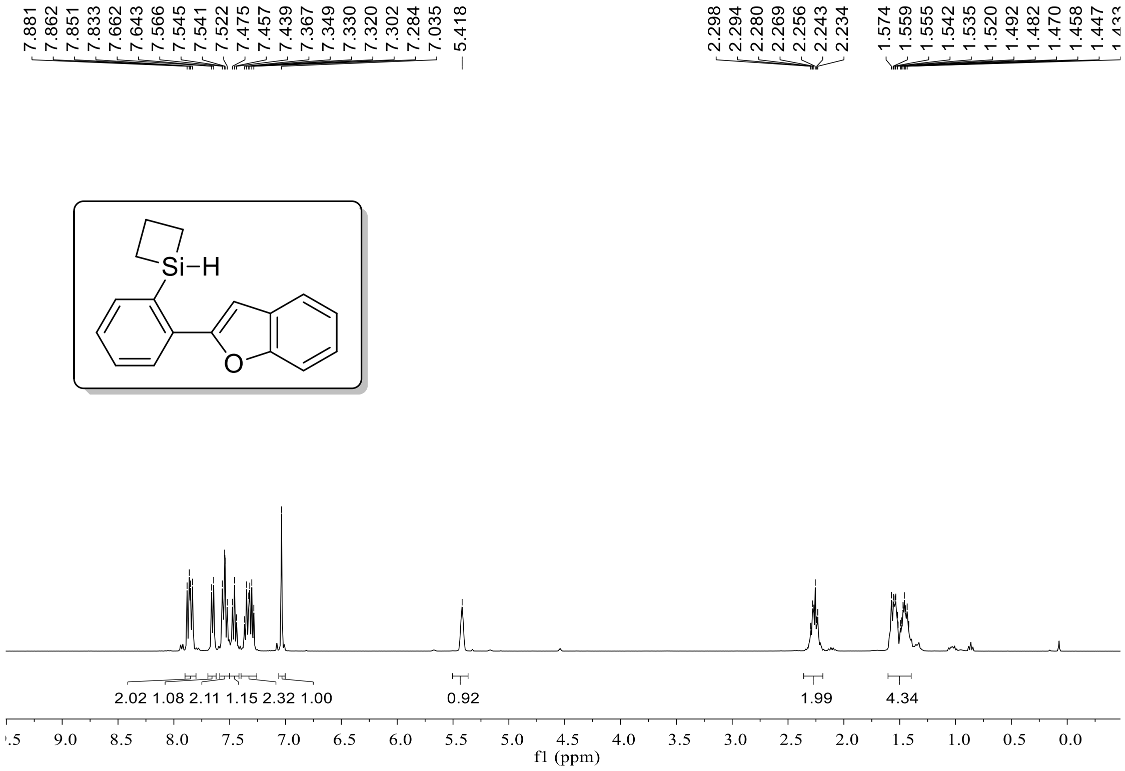

Supplementary Figure 87. ^{1}H NMR spectrum of **1v**

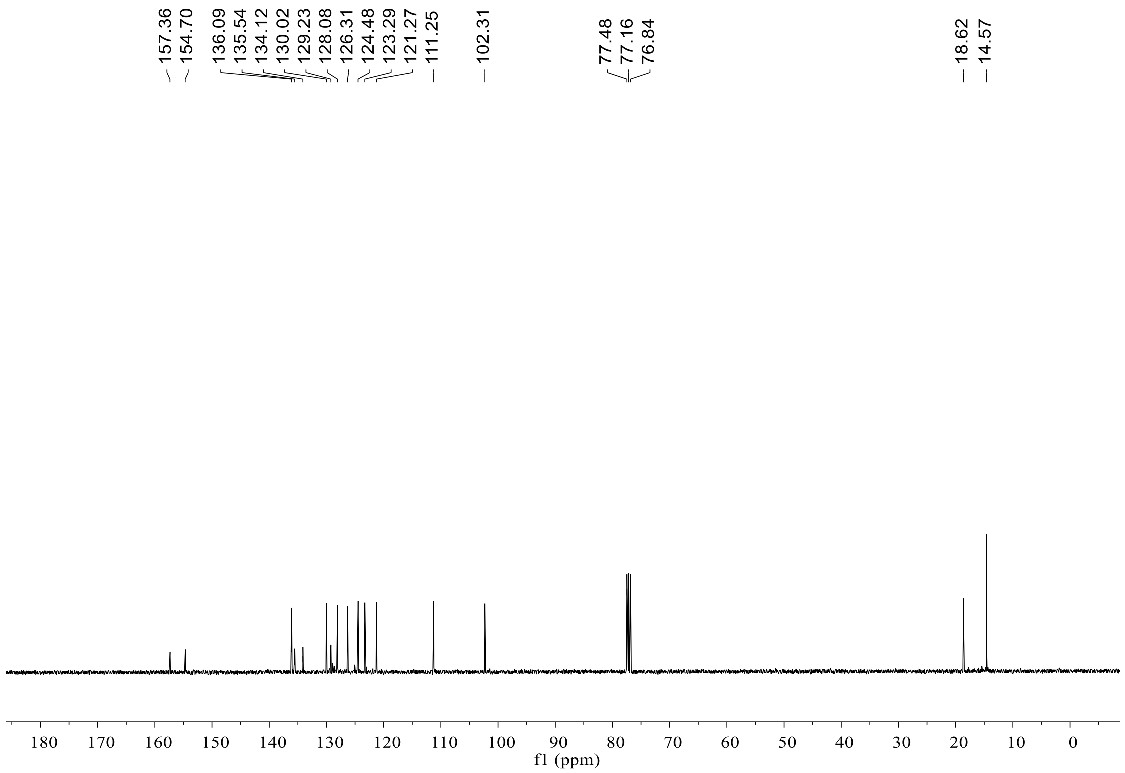

Supplementary Figure 88. ^{13}C NMR spectrum of **1v**

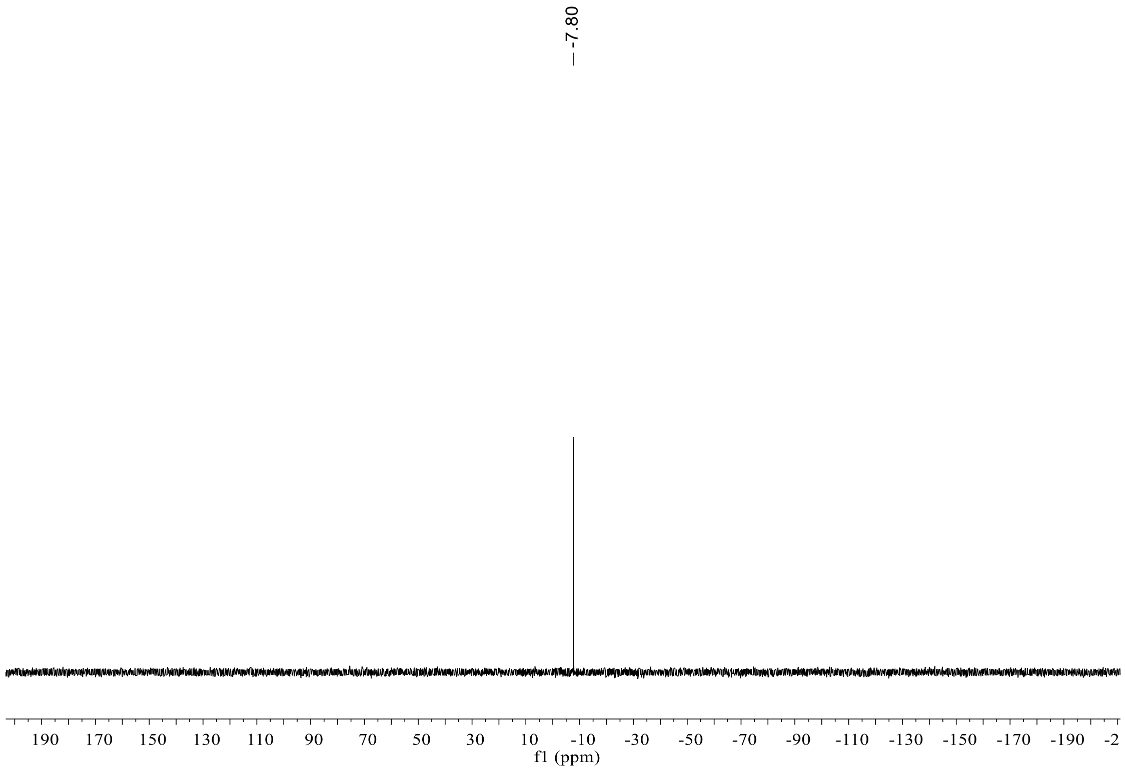

Supplementary Figure 89. ^{29}Si NMR spectrum of **1v**

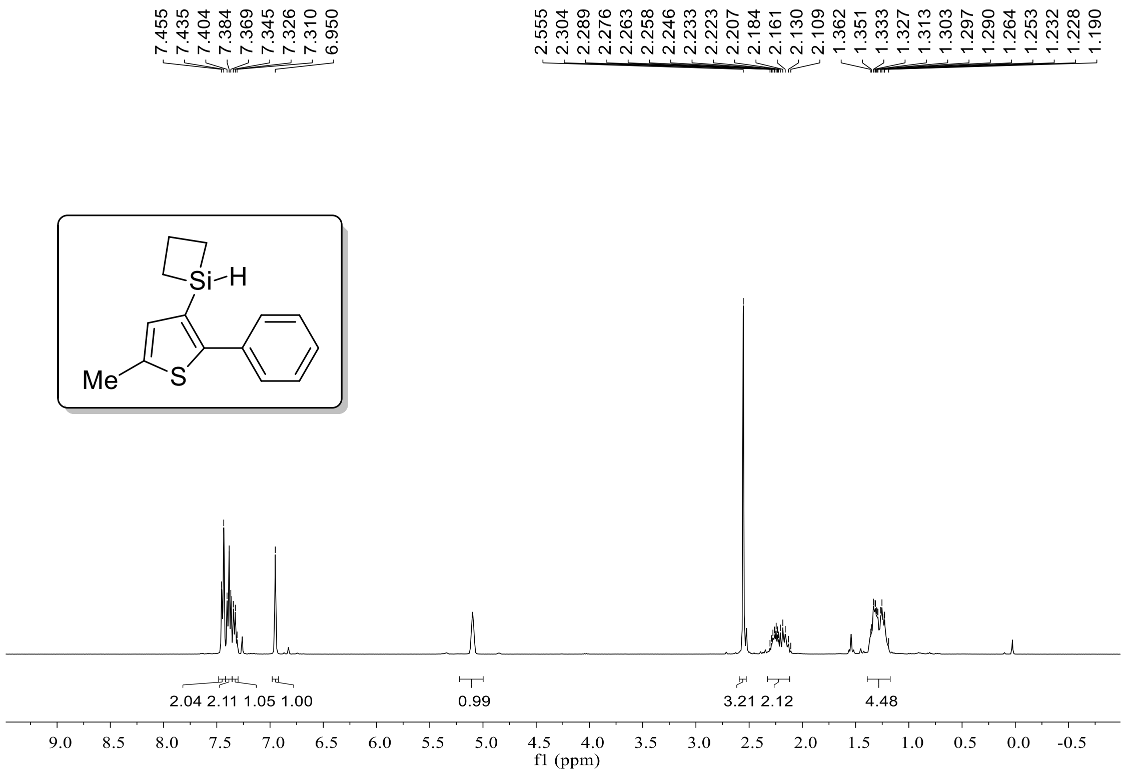

Supplementary Figure 90. ^{1}H NMR spectrum of **1w**

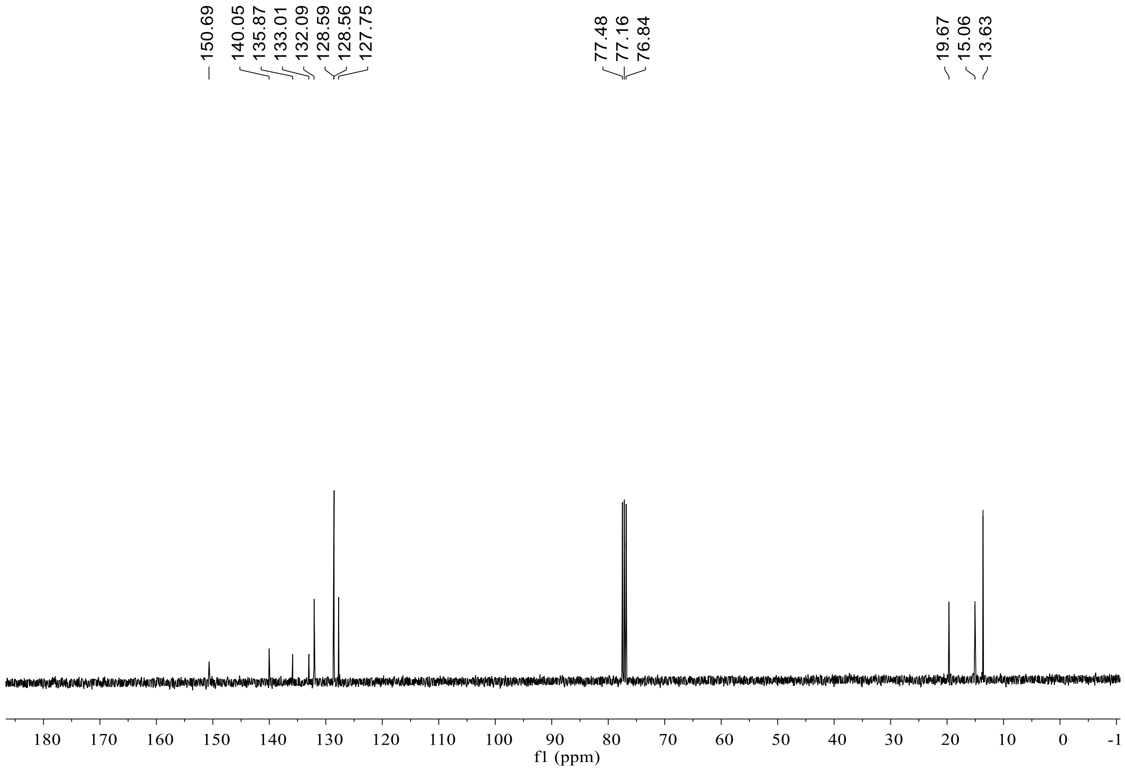

Supplementary Figure 91. ^{13}C NMR spectrum of **1w**

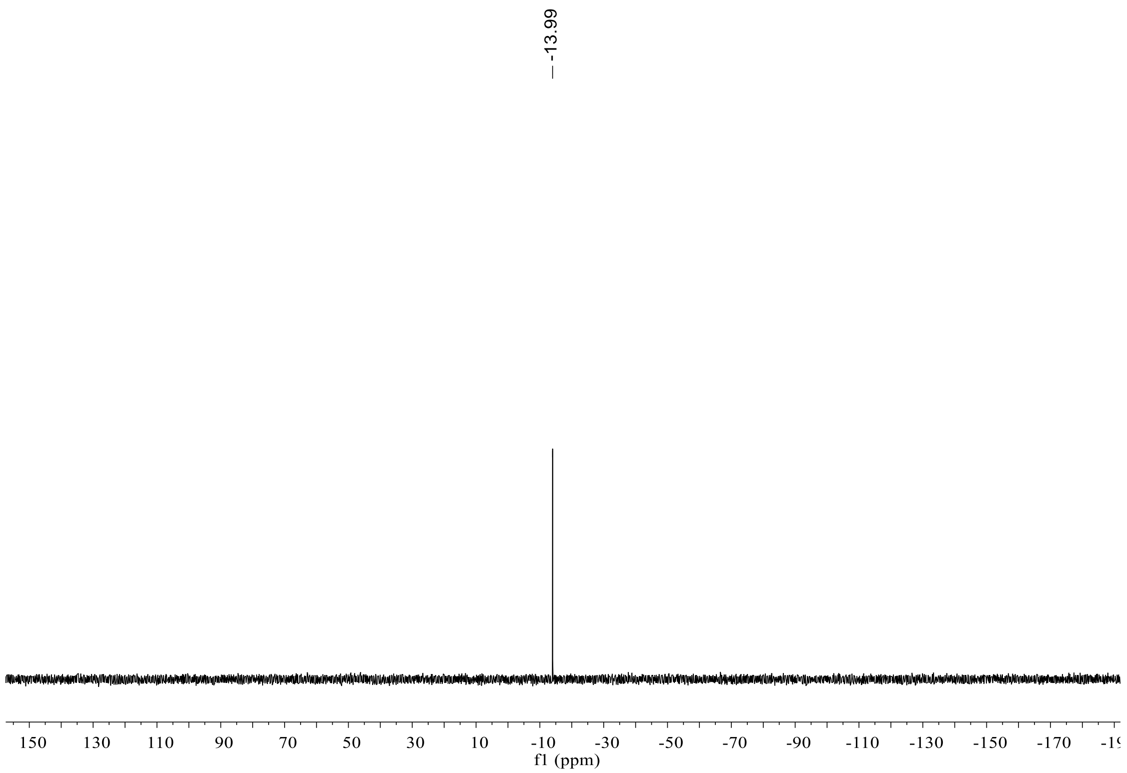

Supplementary Figure 92. ^{29}Si NMR spectrum of **1w**

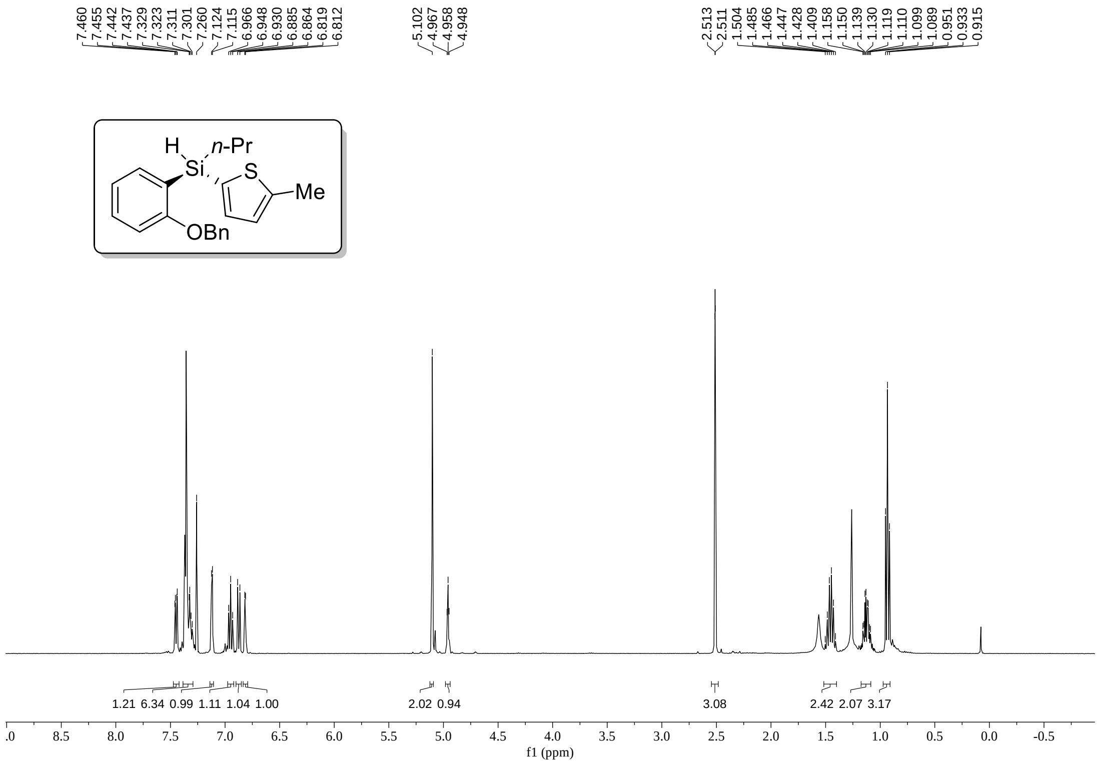

Supplementary Figure 93. ^{1}H NMR spectrum of **3aa**

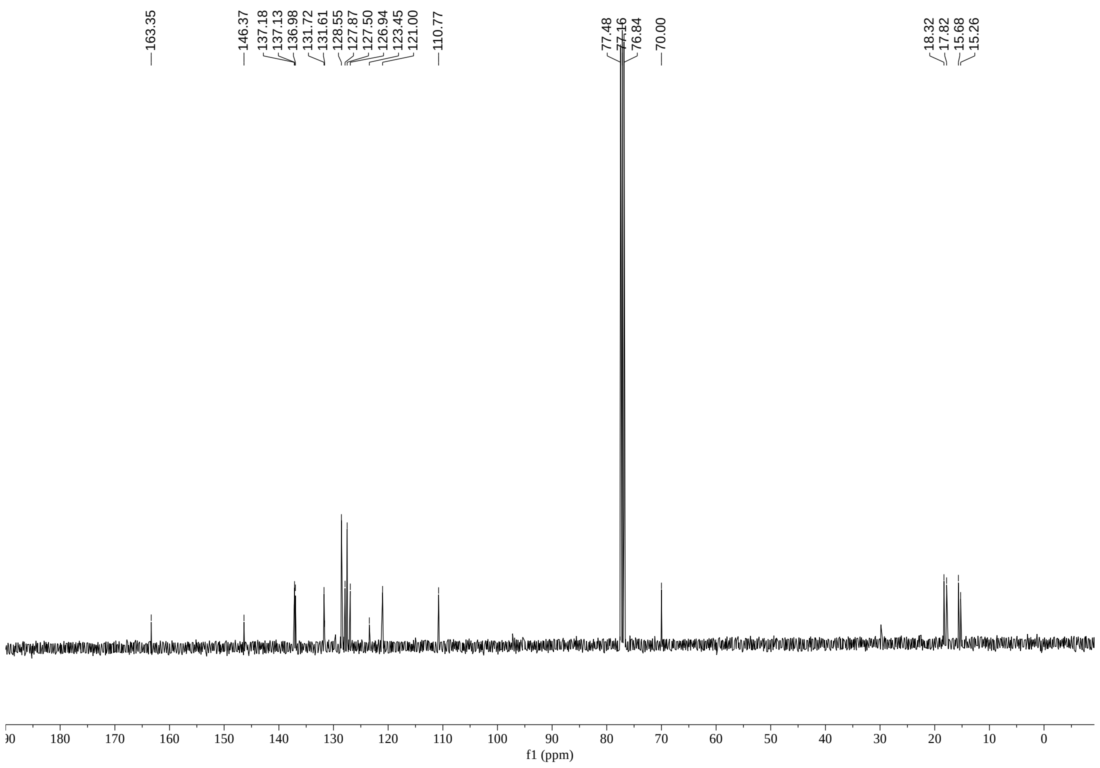

Supplementary Figure 94. ^{13}C NMR spectrum of **3aa**

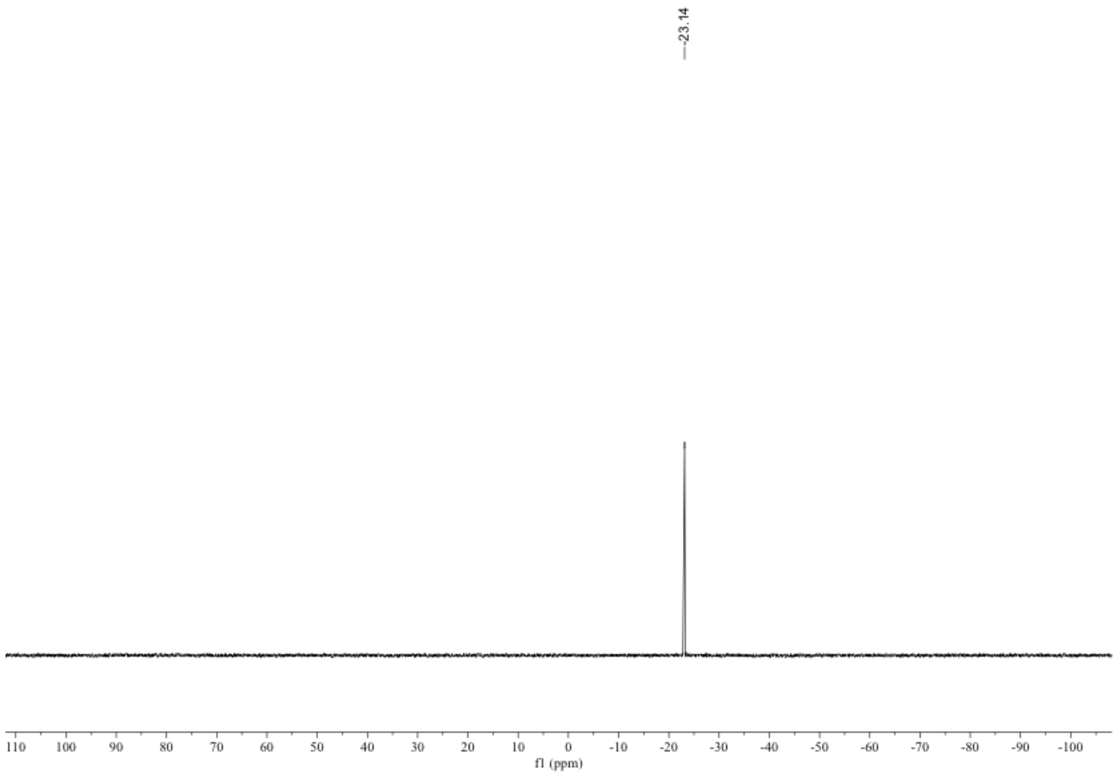

Supplementary Figure 95. ^{29}Si NMR spectrum of **3aa**

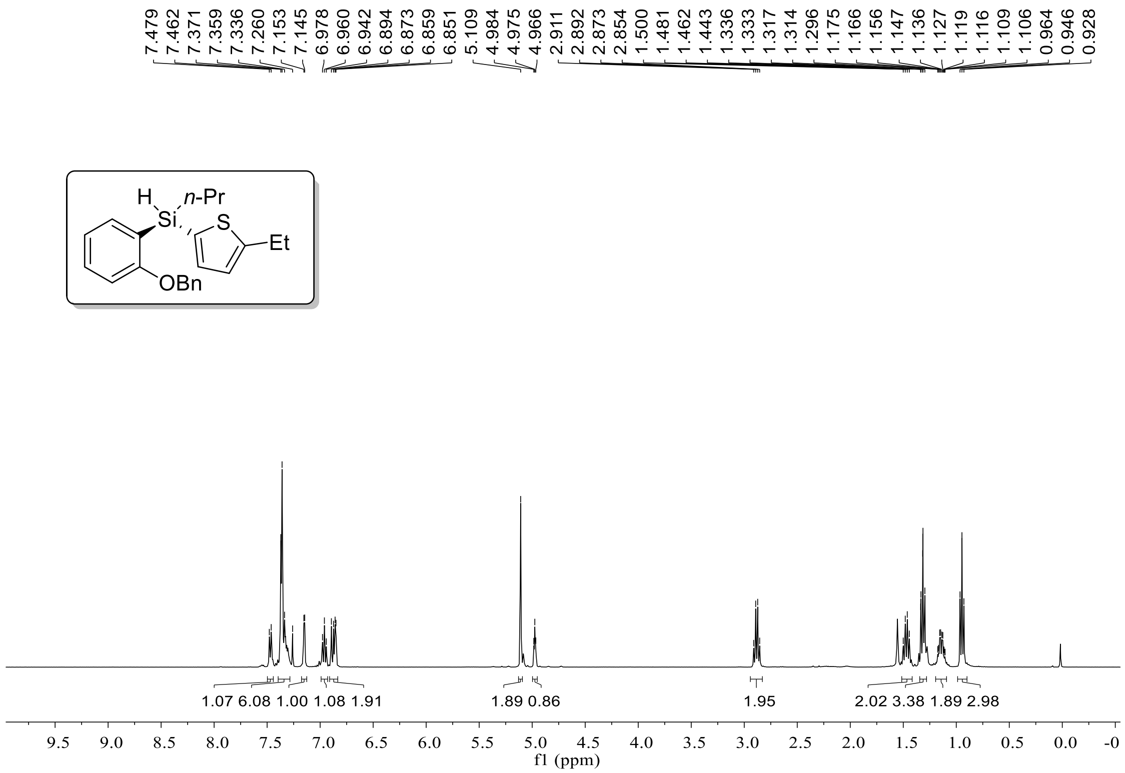

Supplementary Figure 96. ^{1}H NMR spectrum of **3ab**

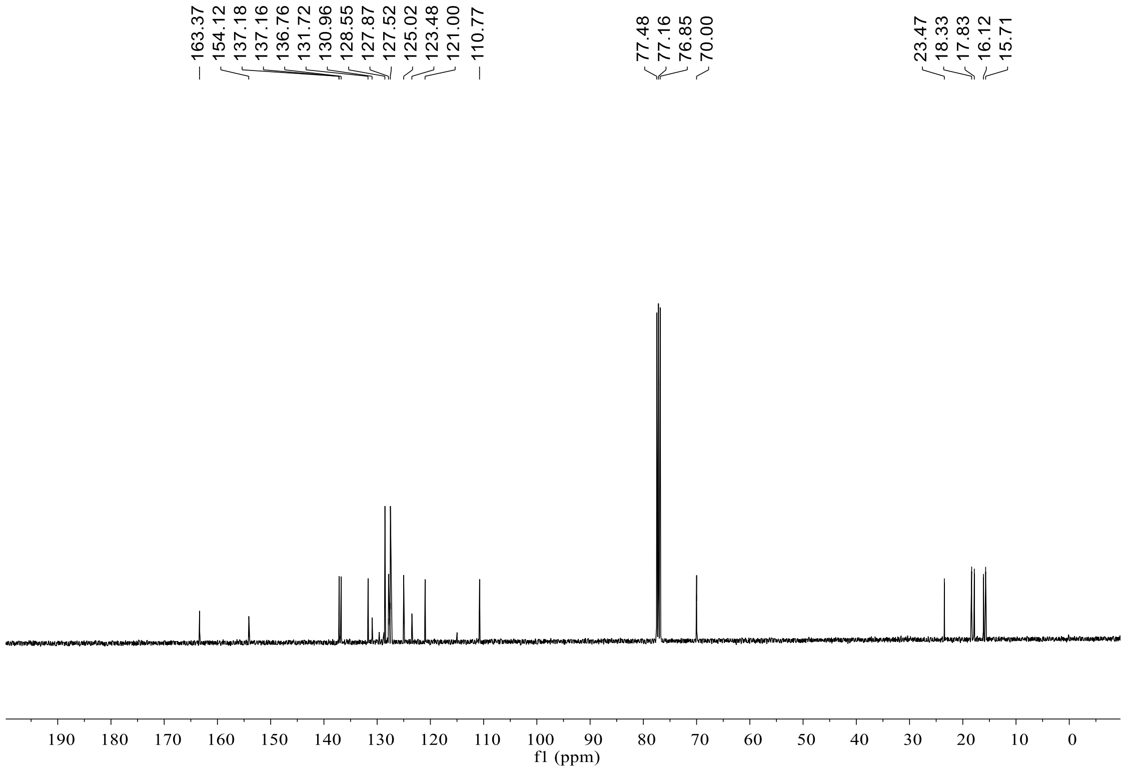

Supplementary Figure 97. ^{13}C NMR spectrum of **3ab**

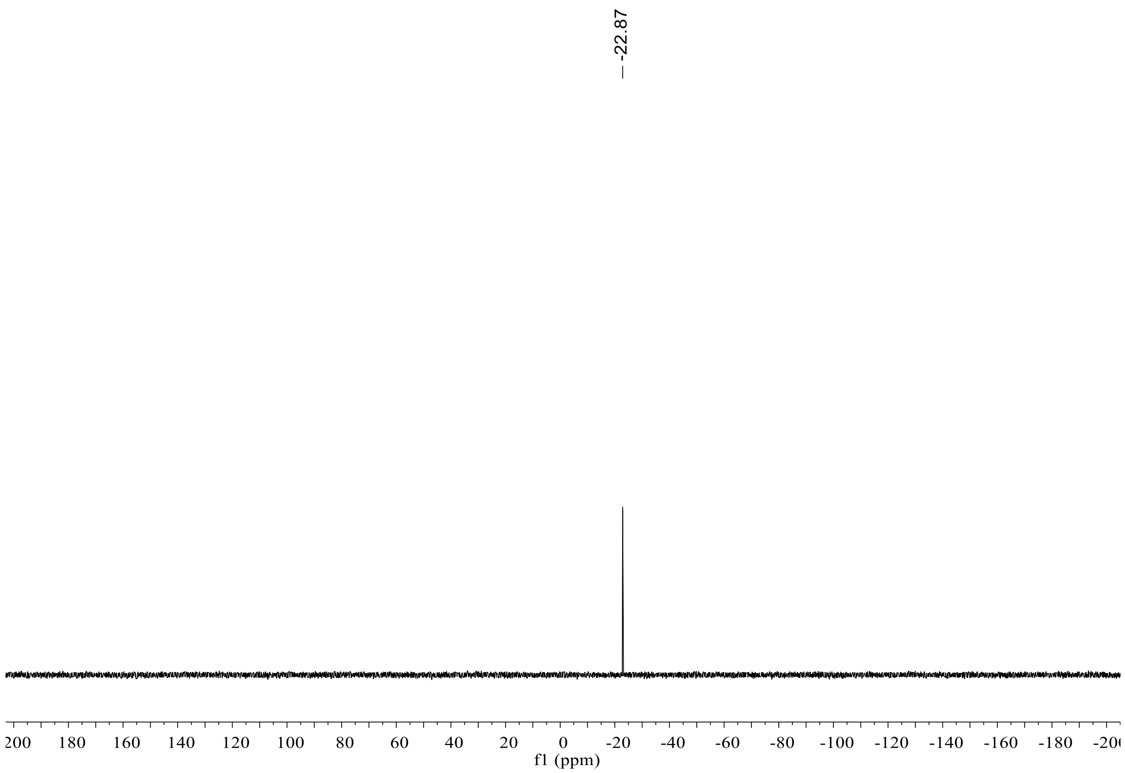

Supplementary Figure 98. ^{29}Si NMR spectrum of **3ab**

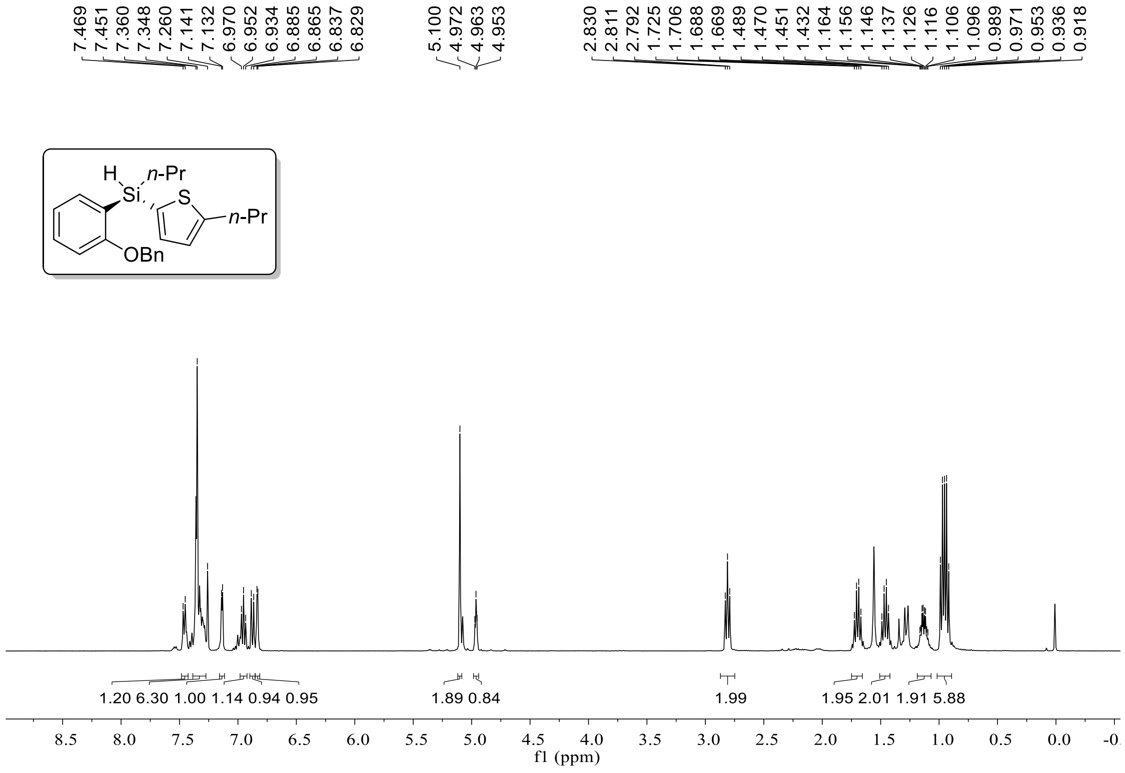

Supplementary Figure 99. ^{1}H NMR spectrum of **3ac**

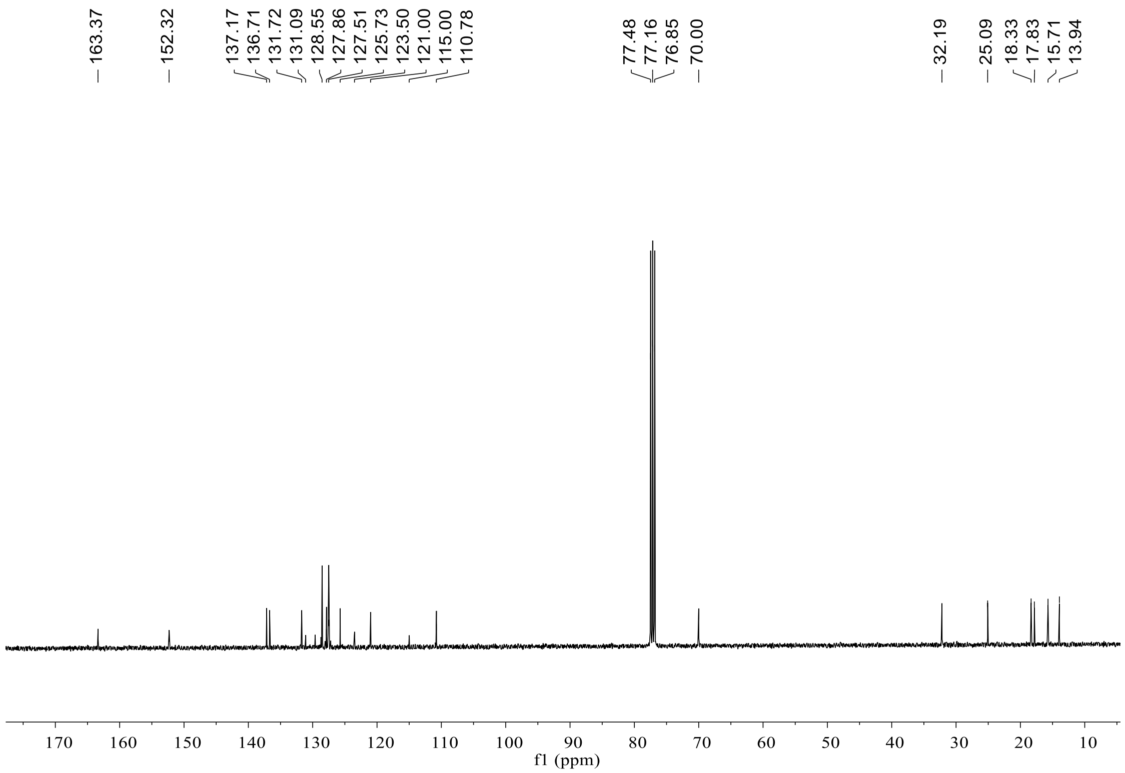

Supplementary Figure 100. ^{13}C NMR spectrum of **3ac**

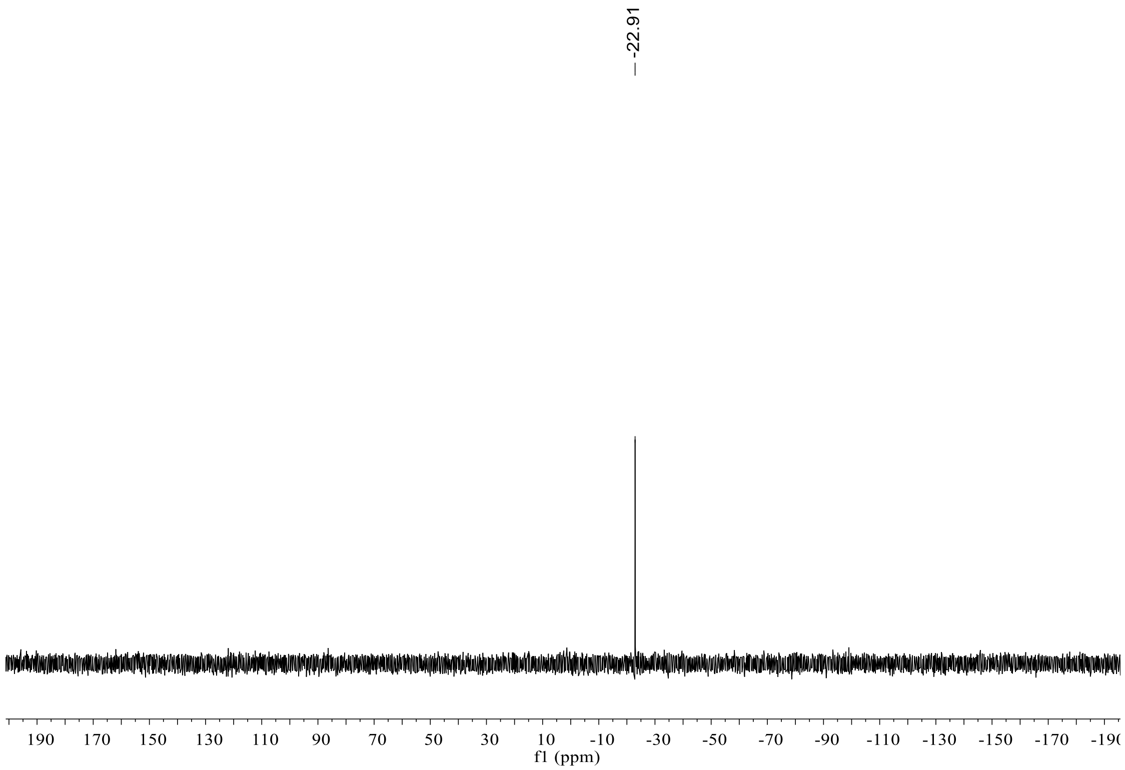

Supplementary Figure 101. ^{29}Si NMR spectrum of **3ac**

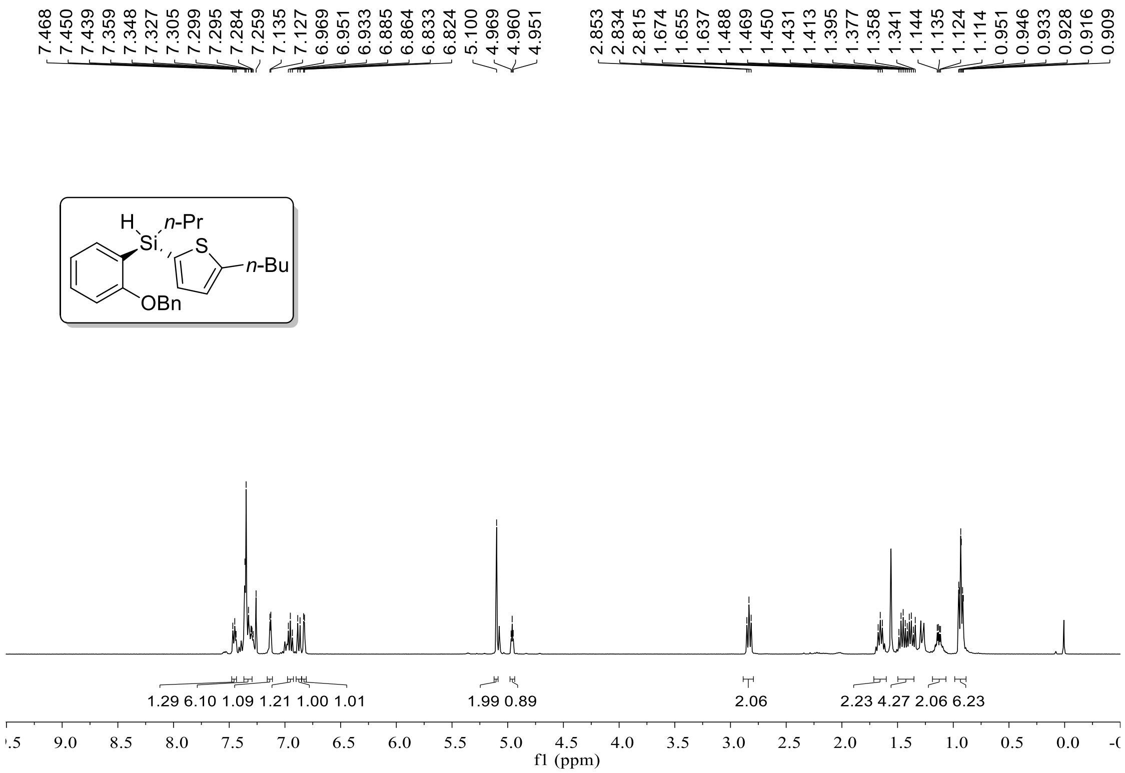

Supplementary Figure 102. ^{1}H NMR spectrum of **3ad**

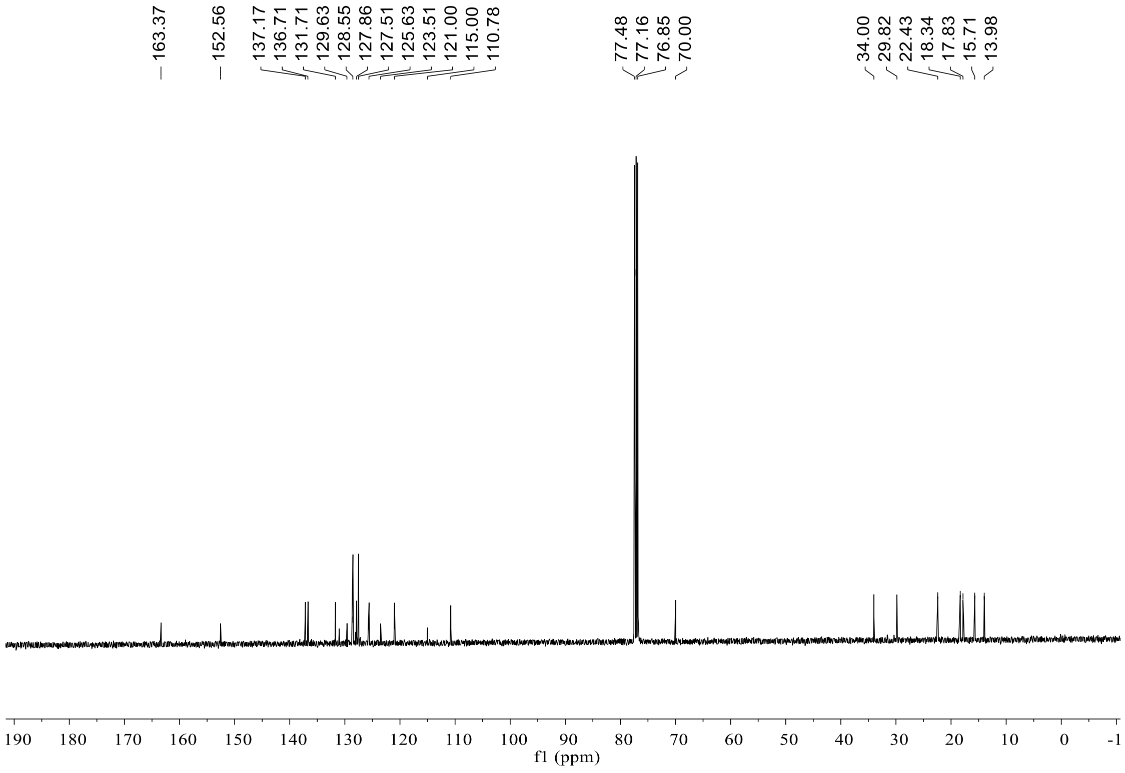

Supplementary Figure 103. ^{13}C NMR spectrum of **3ad**

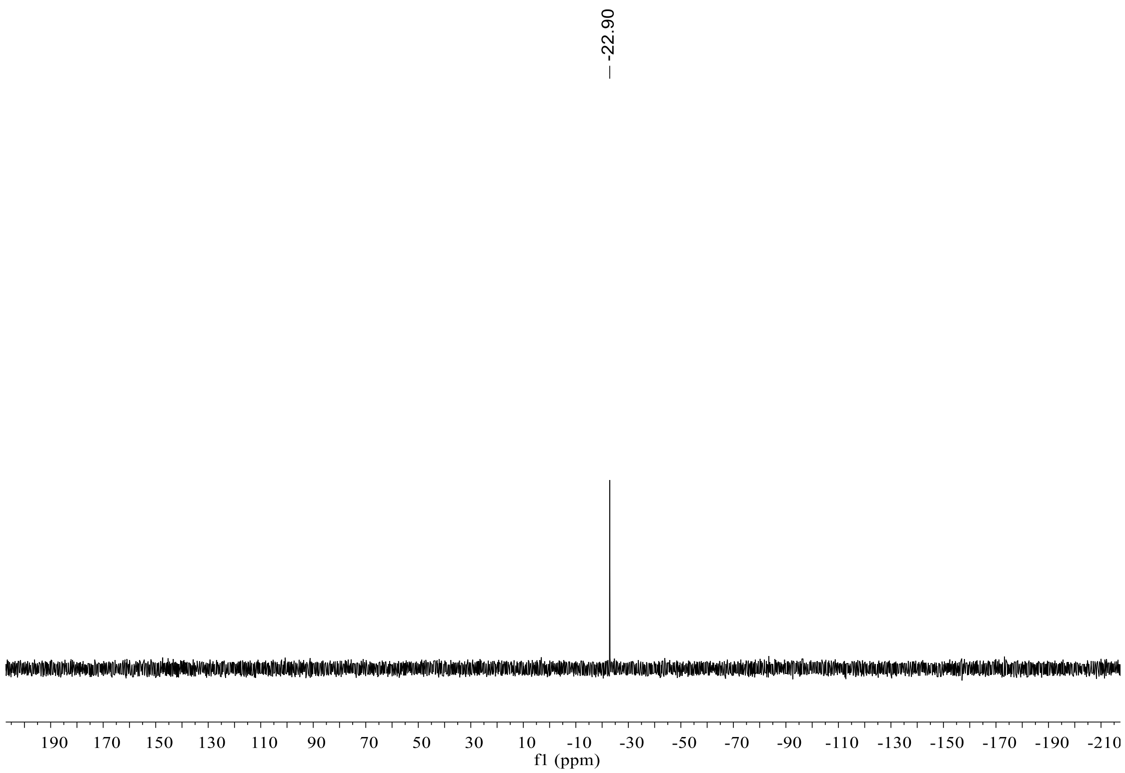

Supplementary Figure 104. ^{29}Si NMR spectrum of **3ad**

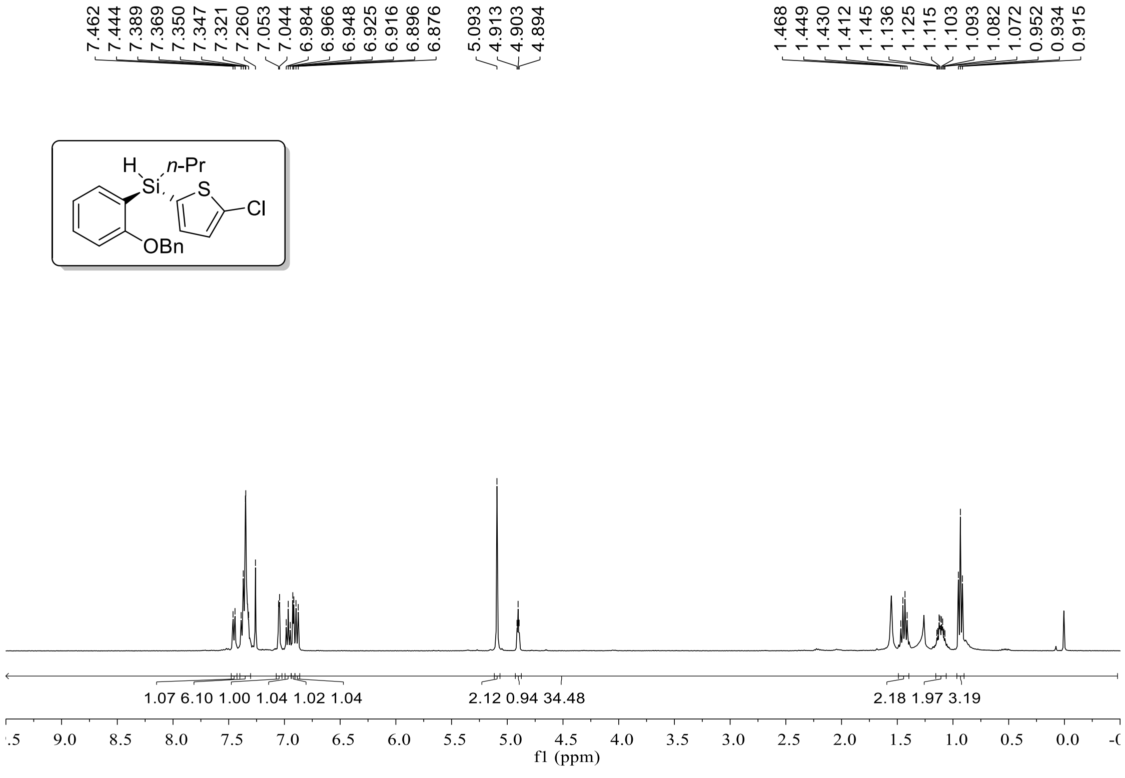

Supplementary Figure 105. ^{1}H NMR spectrum of **3ae**

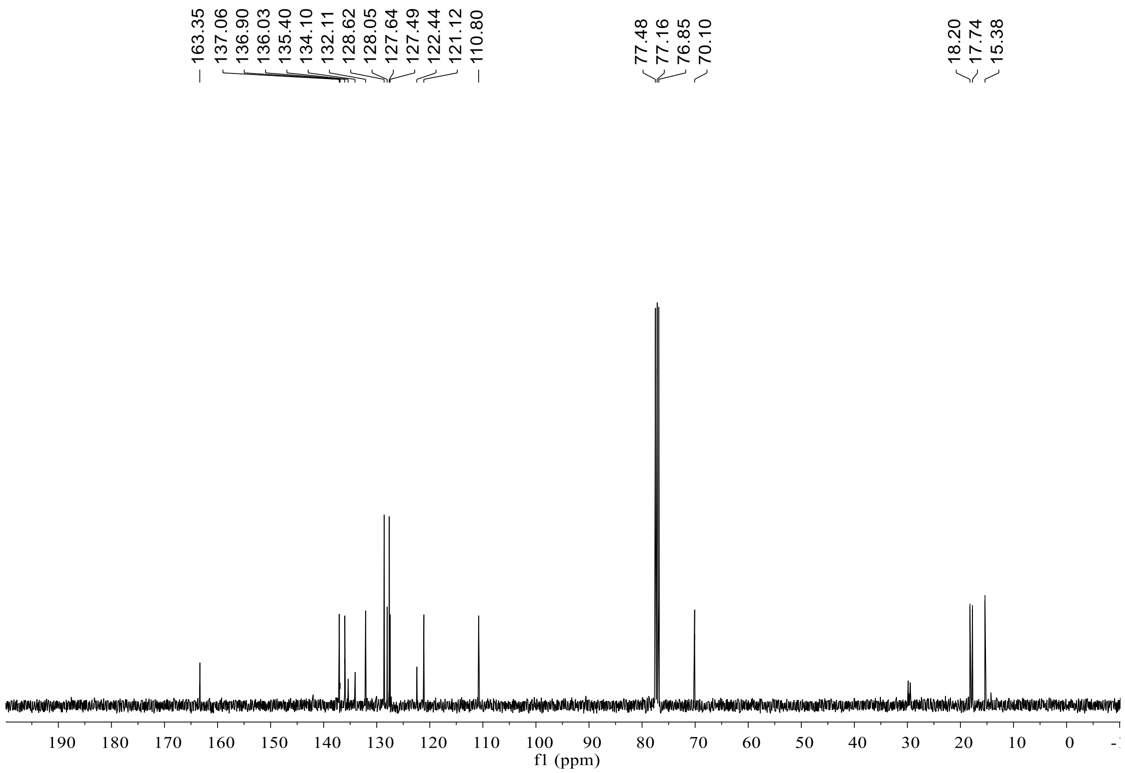

Supplementary Figure 106. ^{13}C NMR spectrum of **3ae**

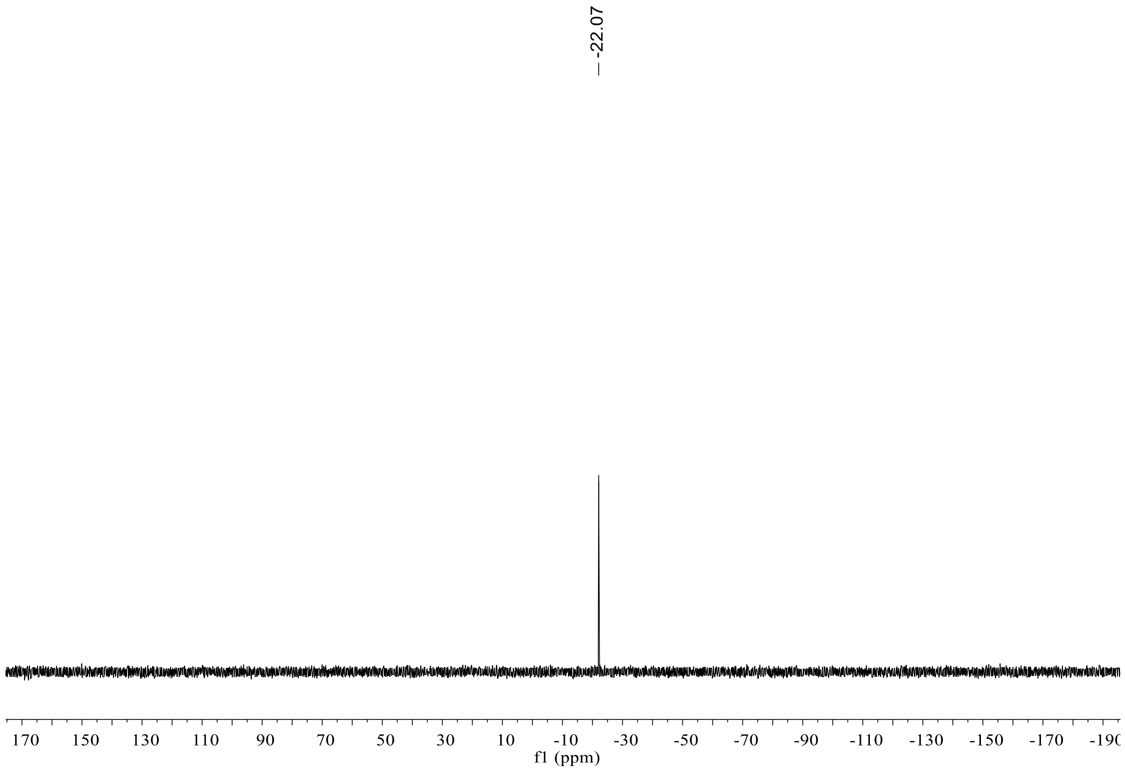

Supplementary Figure 107. ^{29}Si NMR spectrum of **3ae**

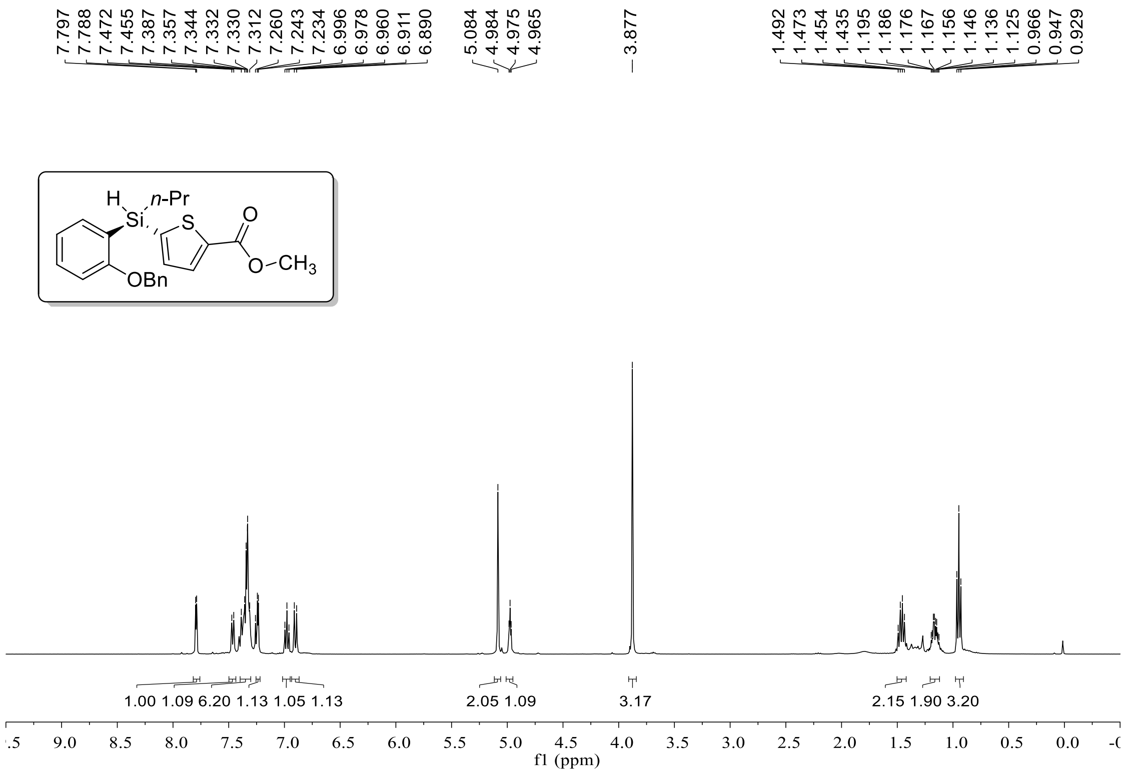

Supplementary Figure 108. ^{1}H NMR spectrum of **3af**

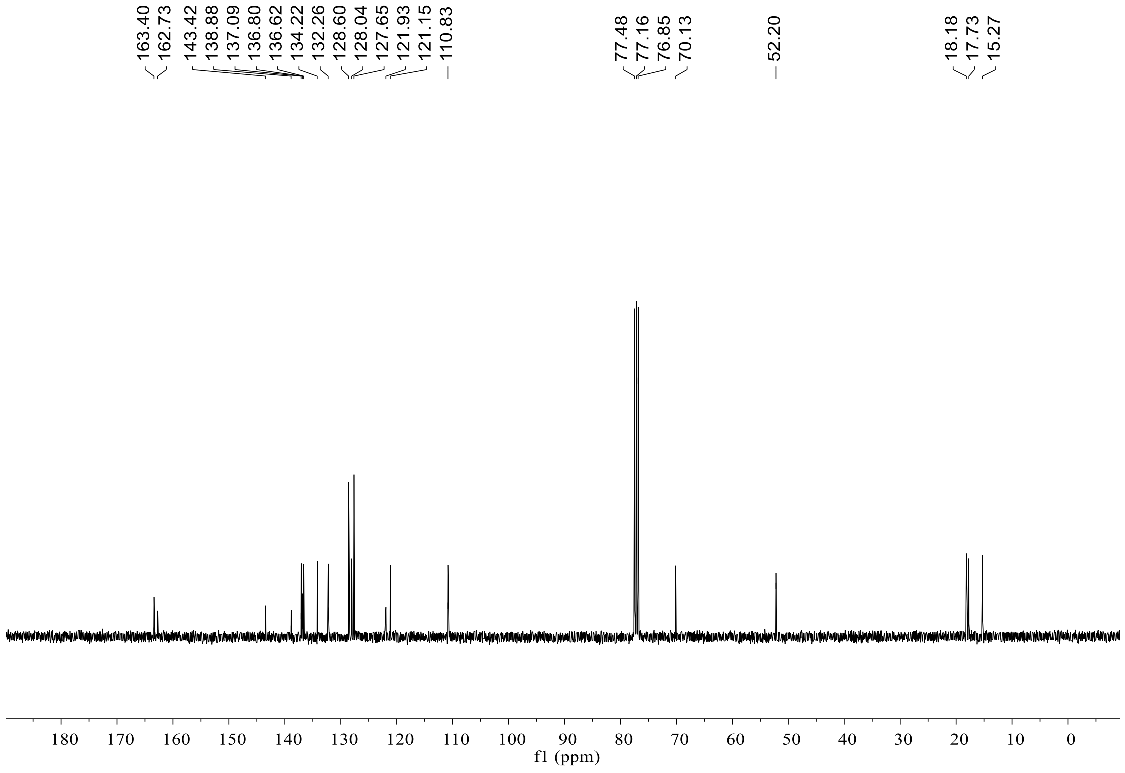

Supplementary Figure 109. ^{13}C NMR spectrum of **3af**

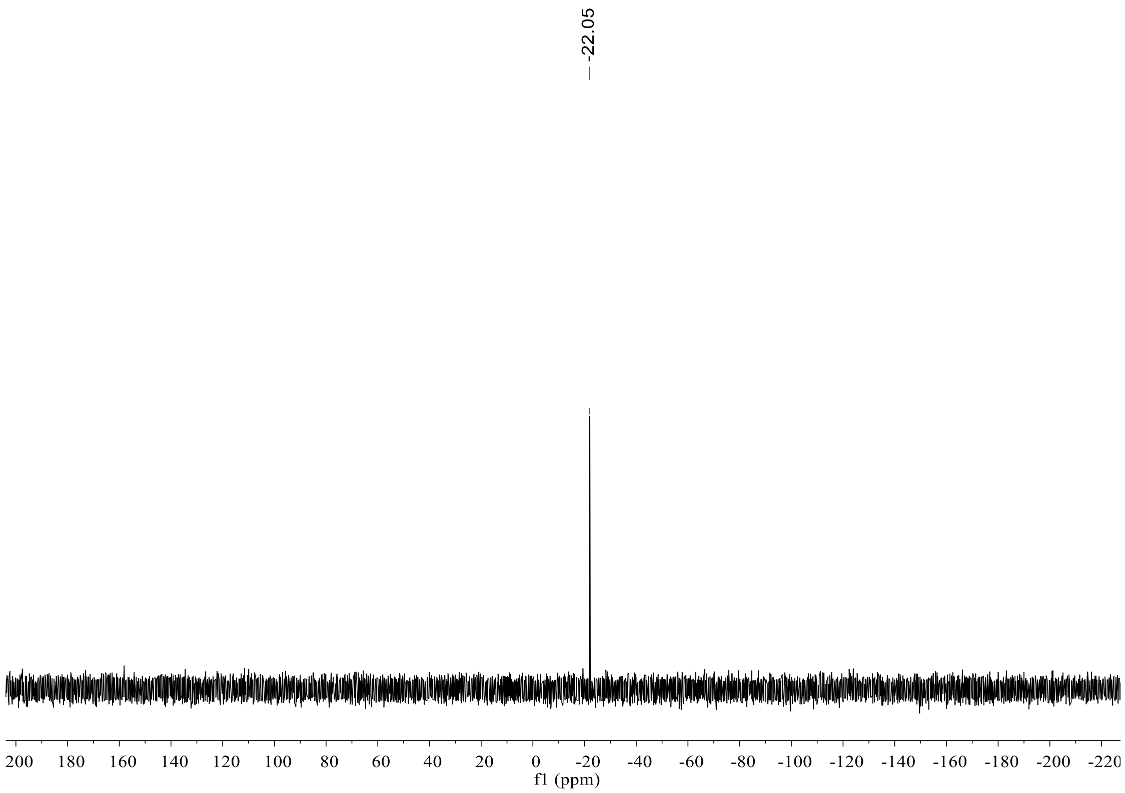

Supplementary Figure 110. ^{29}Si NMR spectrum of **3af**

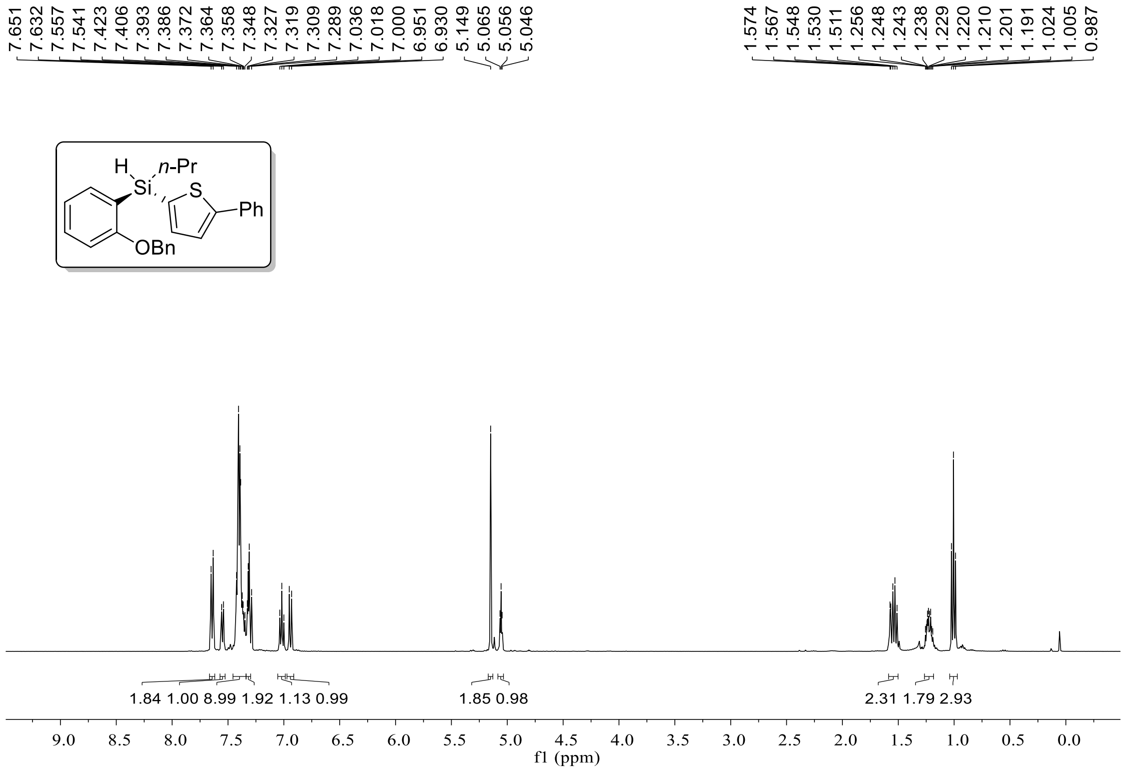

Supplementary Figure 111. ^{1}H NMR spectrum of **3ag**

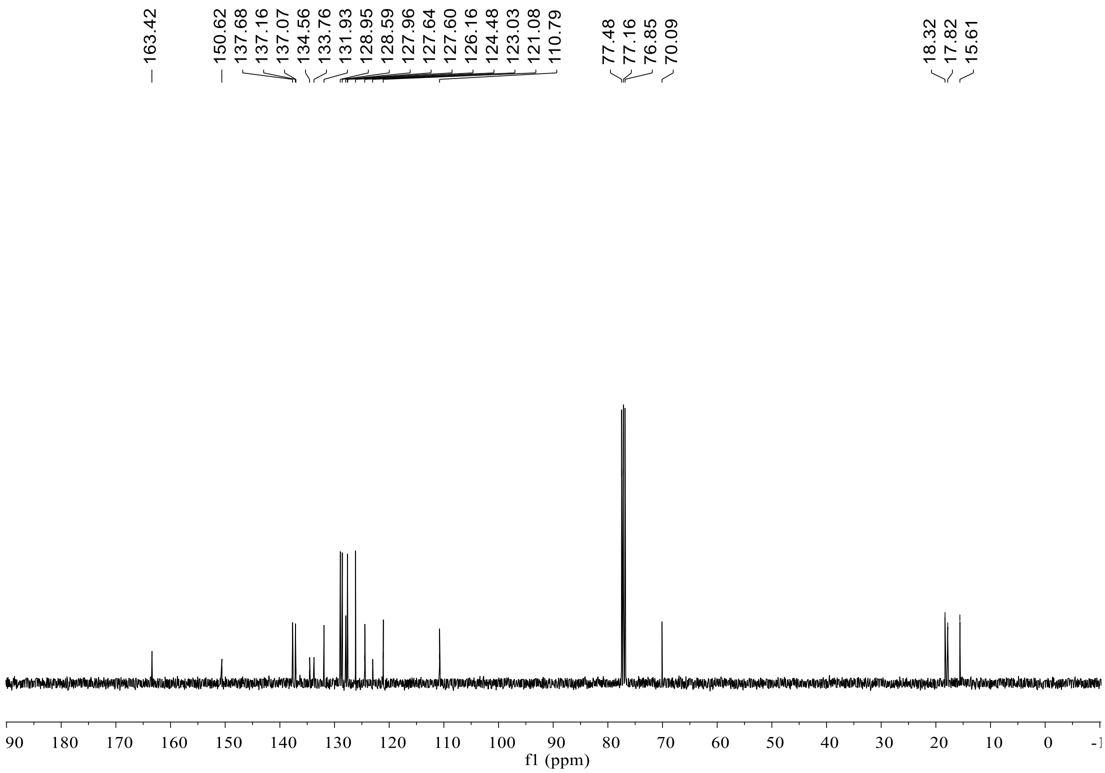

Supplementary Figure 112. ^{13}C NMR spectrum of **3ag**

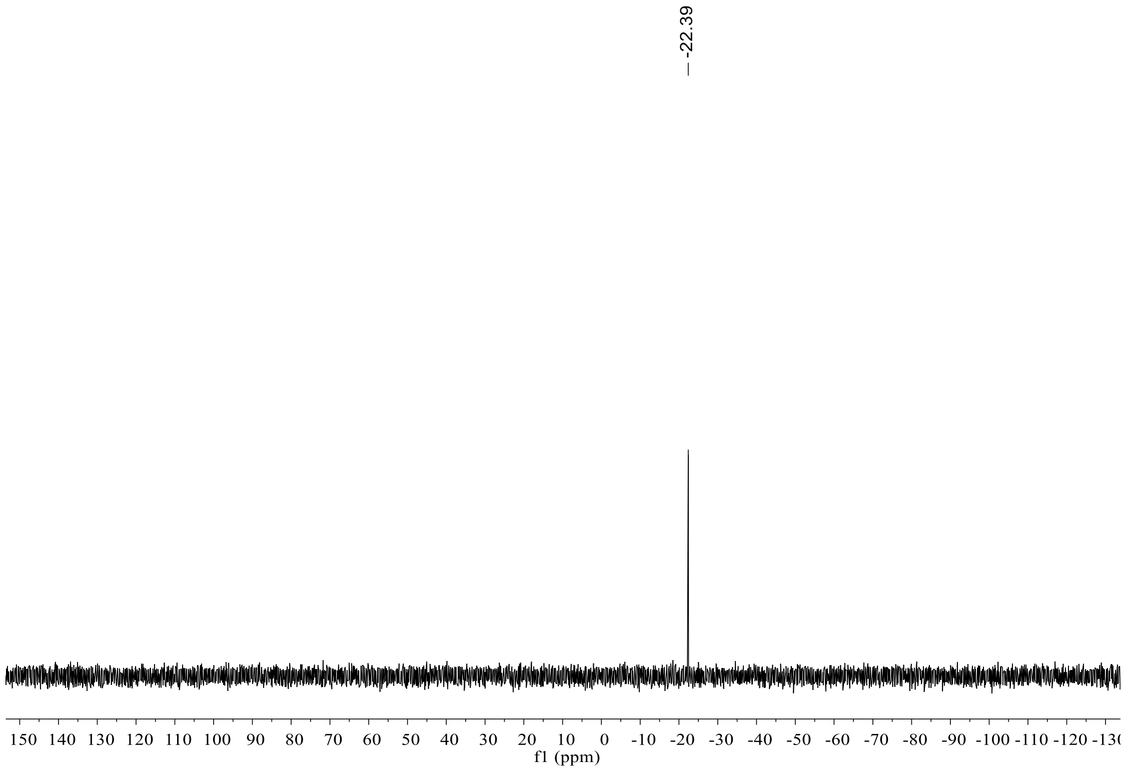

Supplementary Figure 113. ^{29}Si NMR spectrum of **3ag**

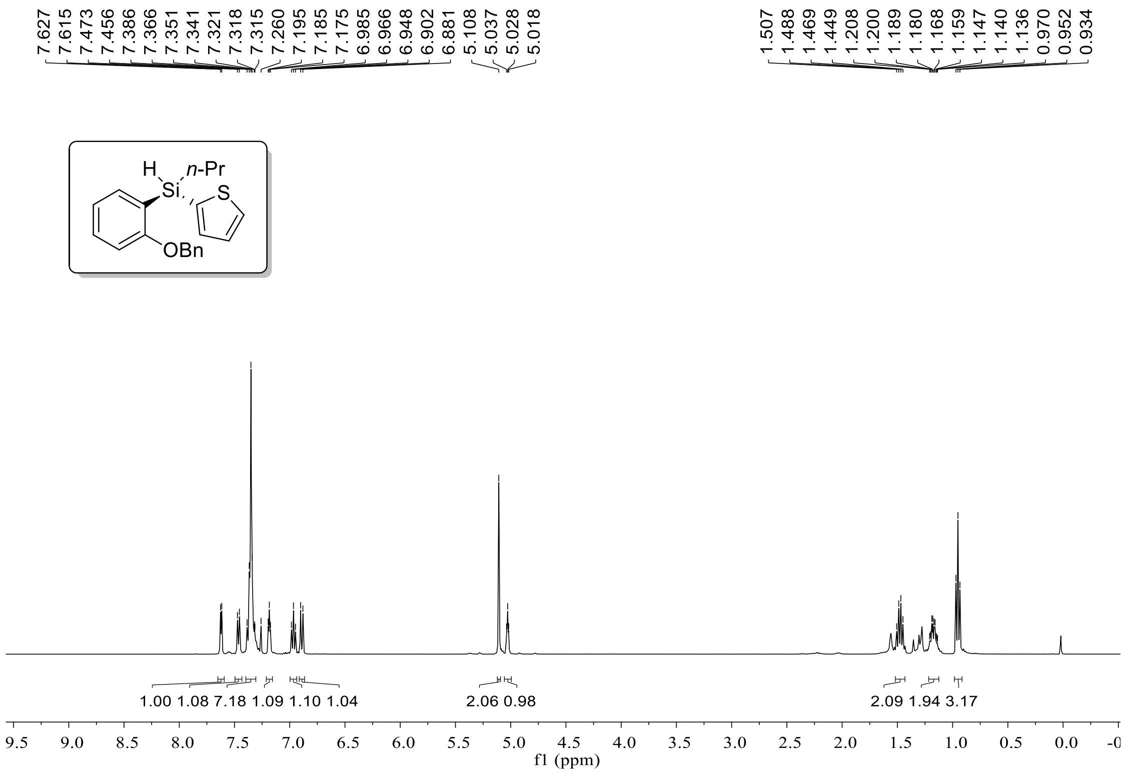

Supplementary Figure 114. ^{1}H NMR spectrum of **3ah**

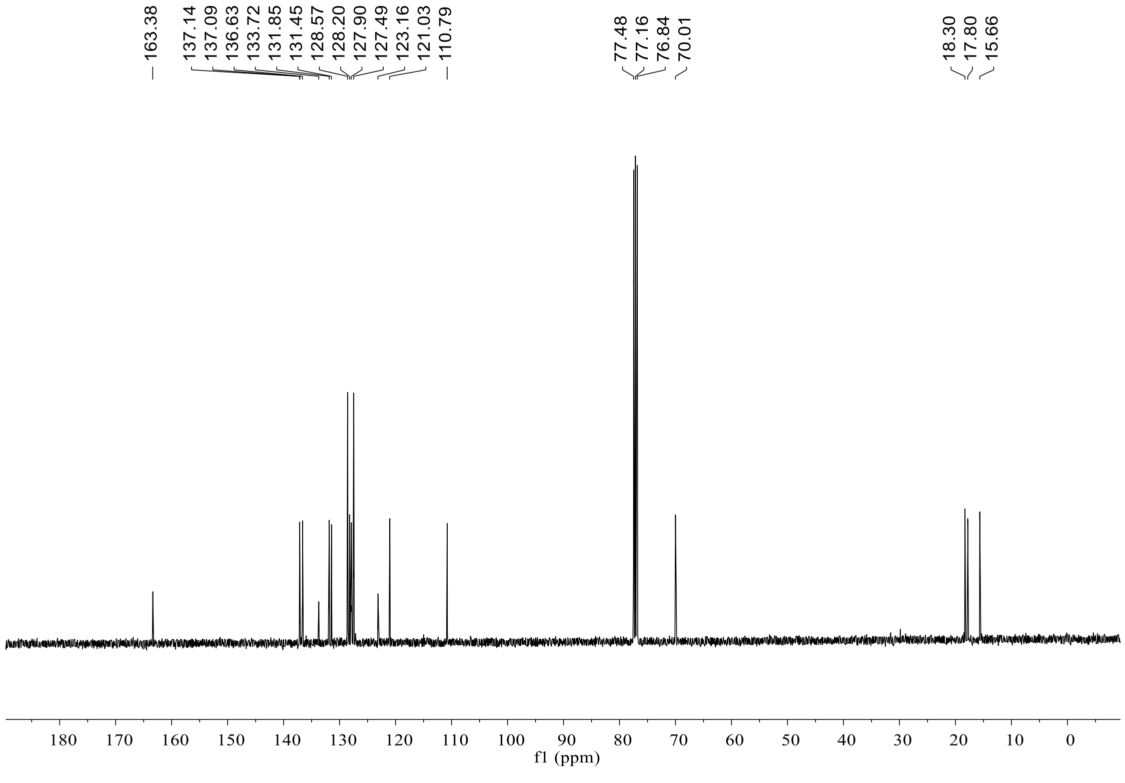

Supplementary Figure 115. ^{13}C NMR spectrum of **3ah**

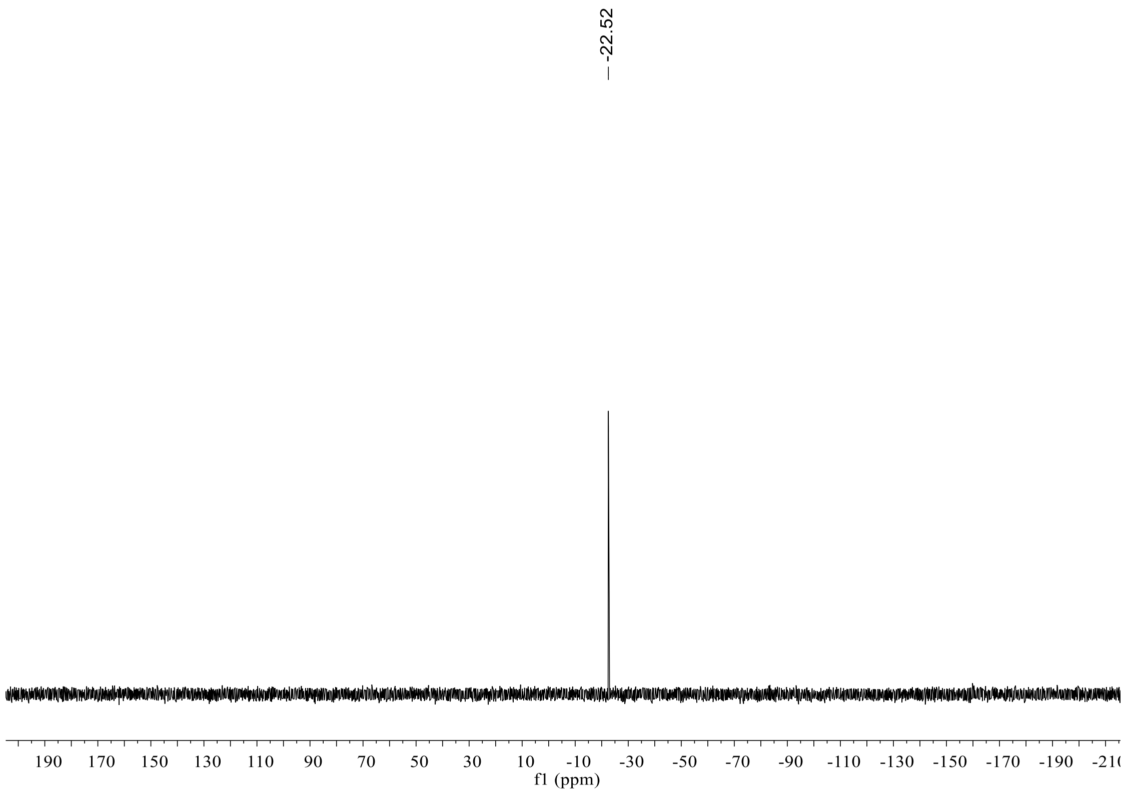

Supplementary Figure 116. ^{29}Si NMR spectrum of **3ah**

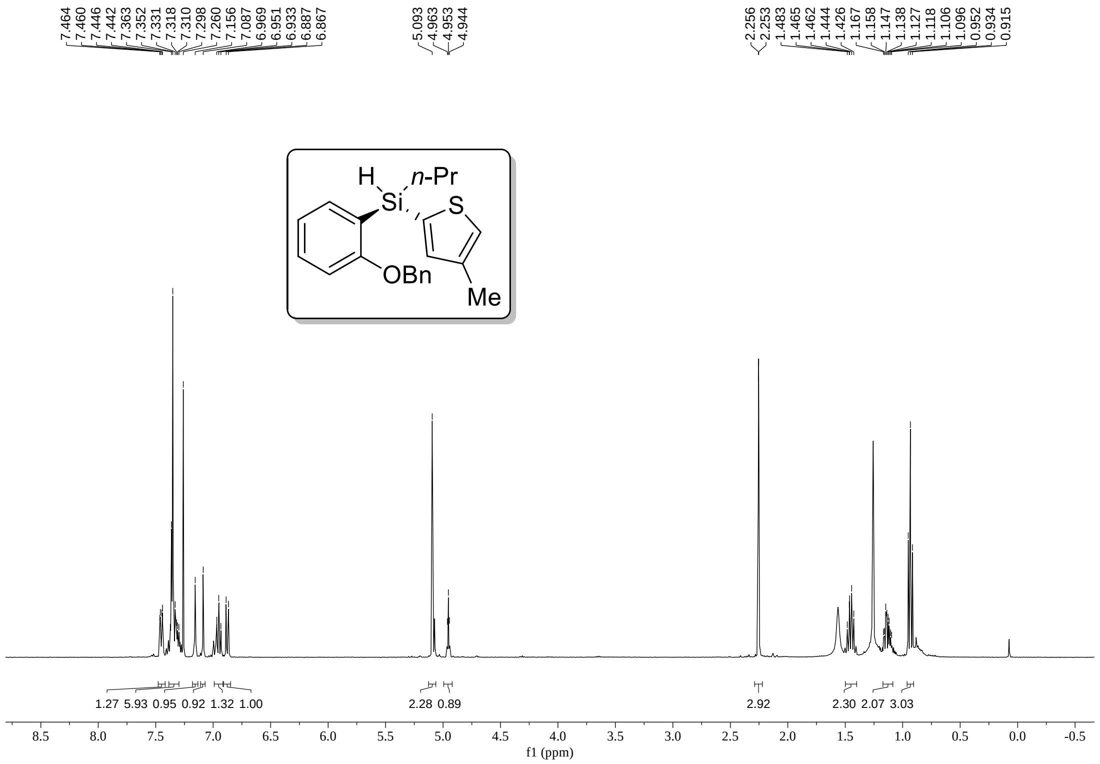

Supplementary Figure 117. ^{1}H NMR spectrum of **3ai**

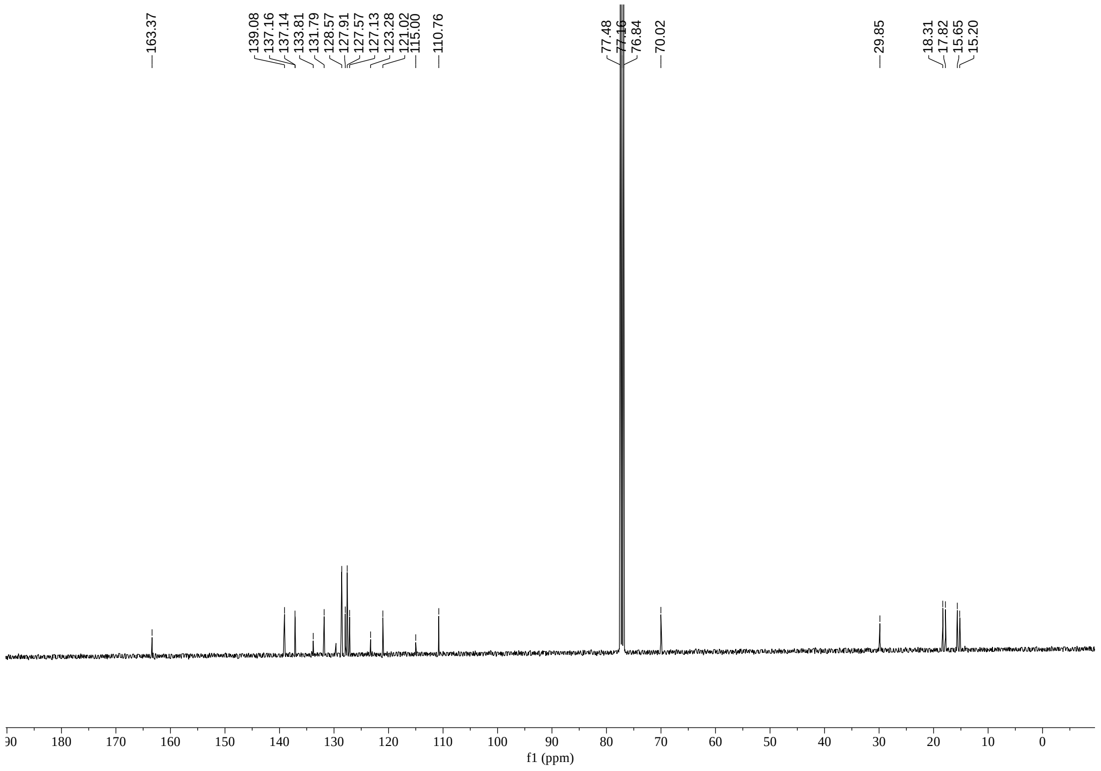

Supplementary Figure 118. ^{13}C NMR spectrum of **3ai**

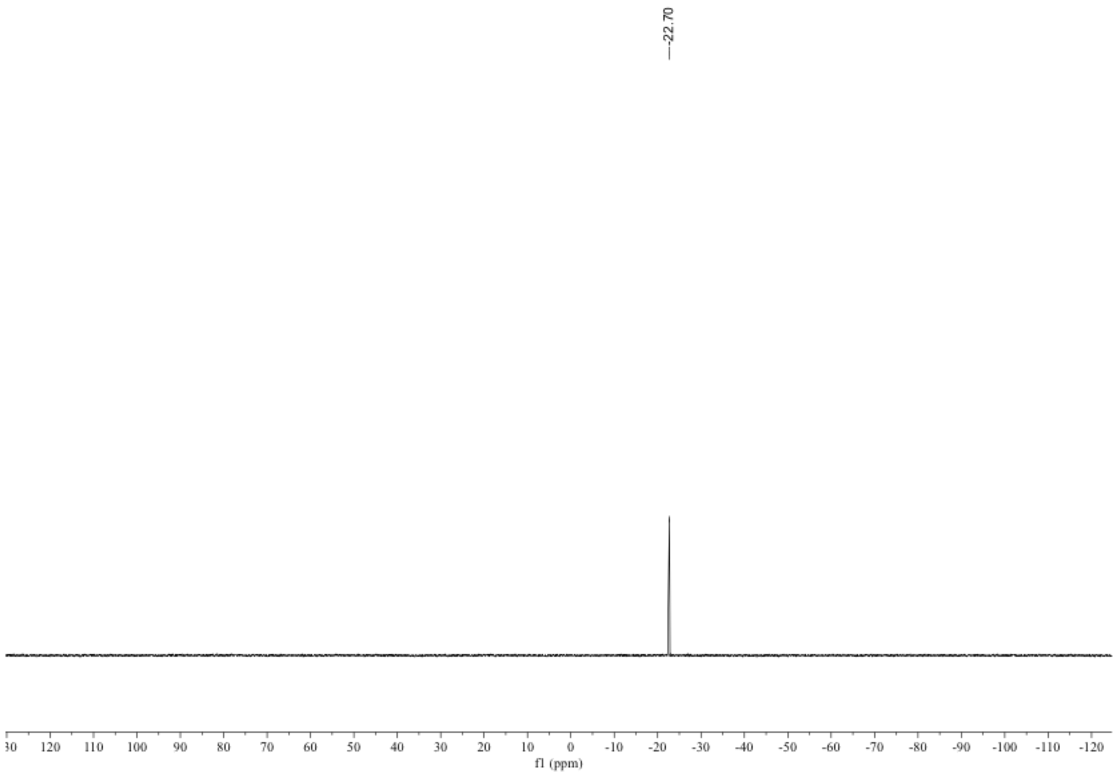

Supplementary Figure 119. ^{29}Si NMR spectrum of **3ai**

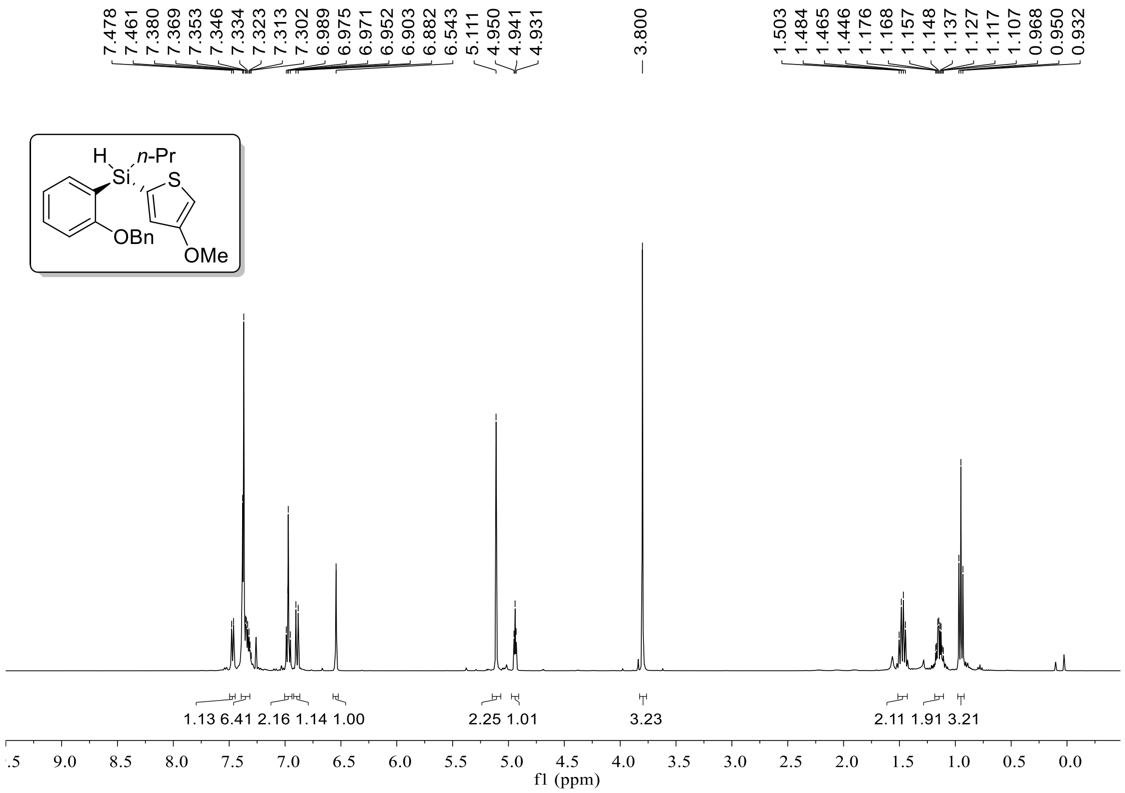

Supplementary Figure 120. ^{1}H NMR spectrum of **3aj**

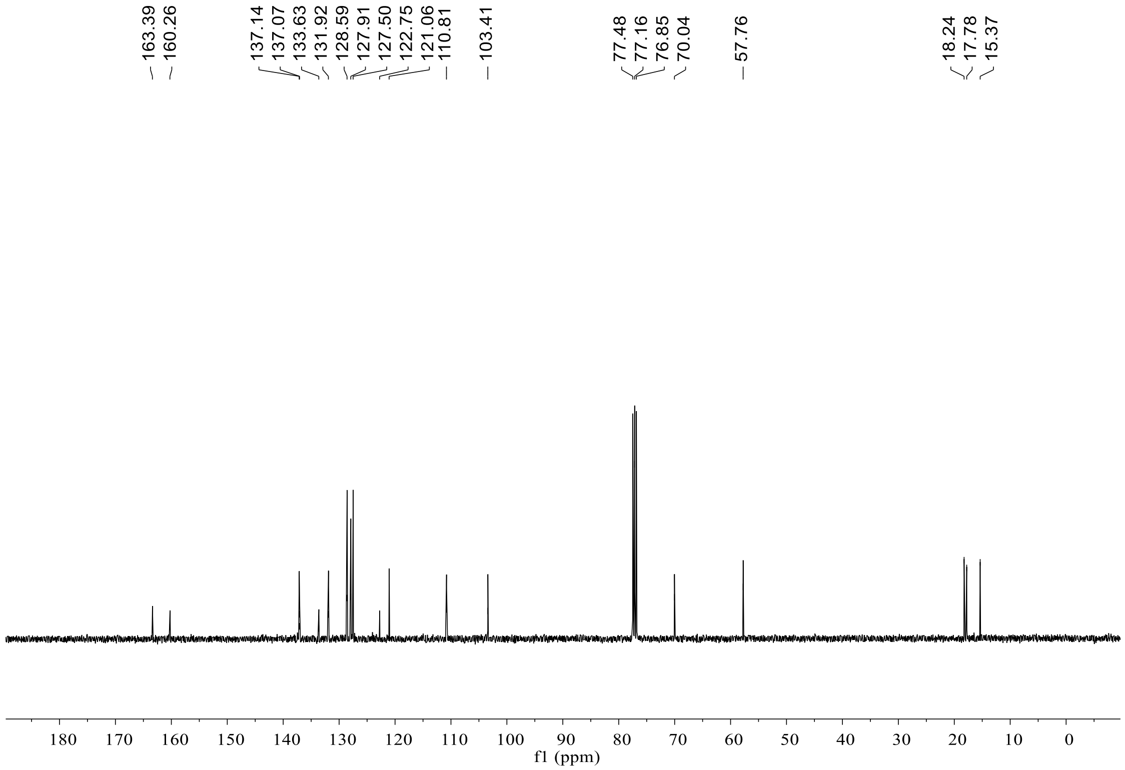

Supplementary Figure 121. ^{13}C NMR spectrum of **3aj**

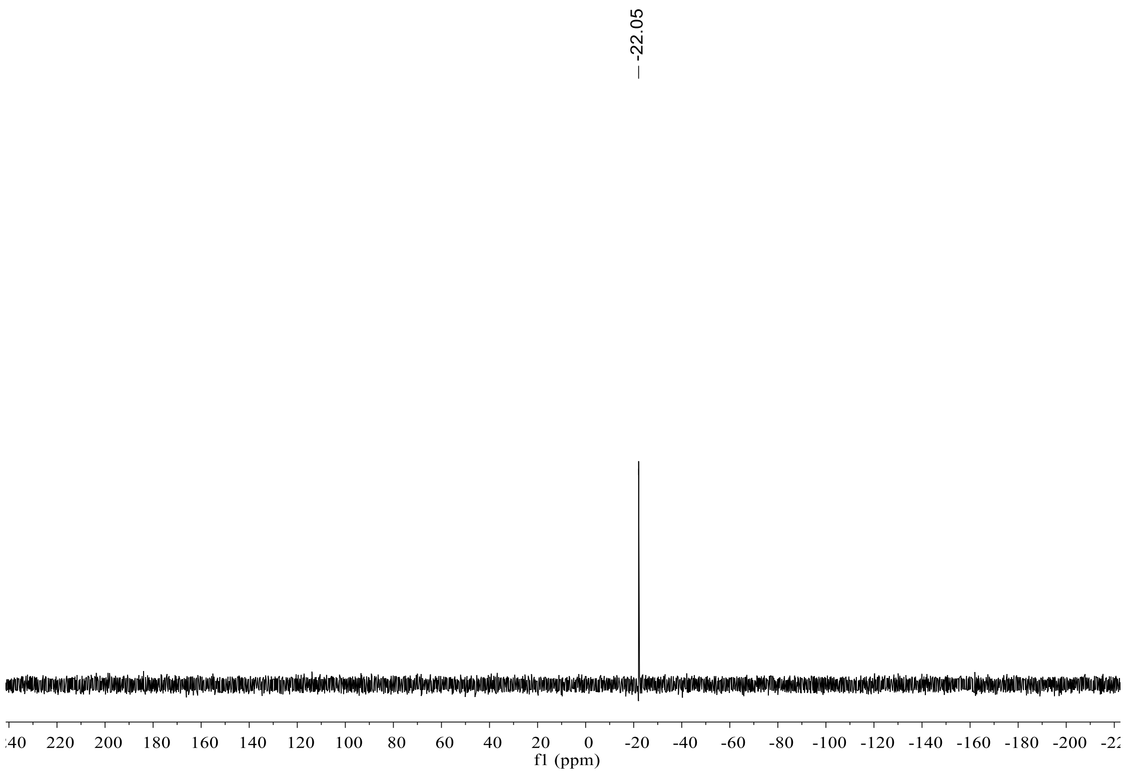

Supplementary Figure 122. ^{29}Si NMR spectrum of **3aj**

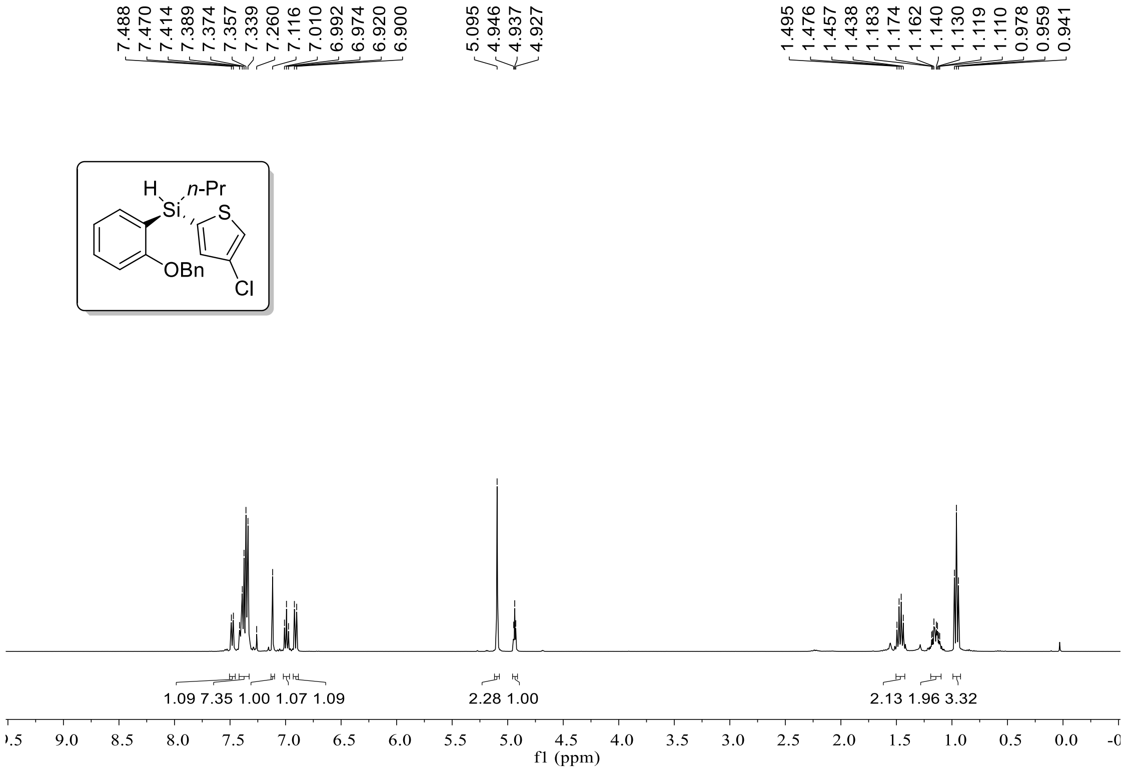

Supplementary Figure 123. ^{1}H NMR spectrum of **3ak**

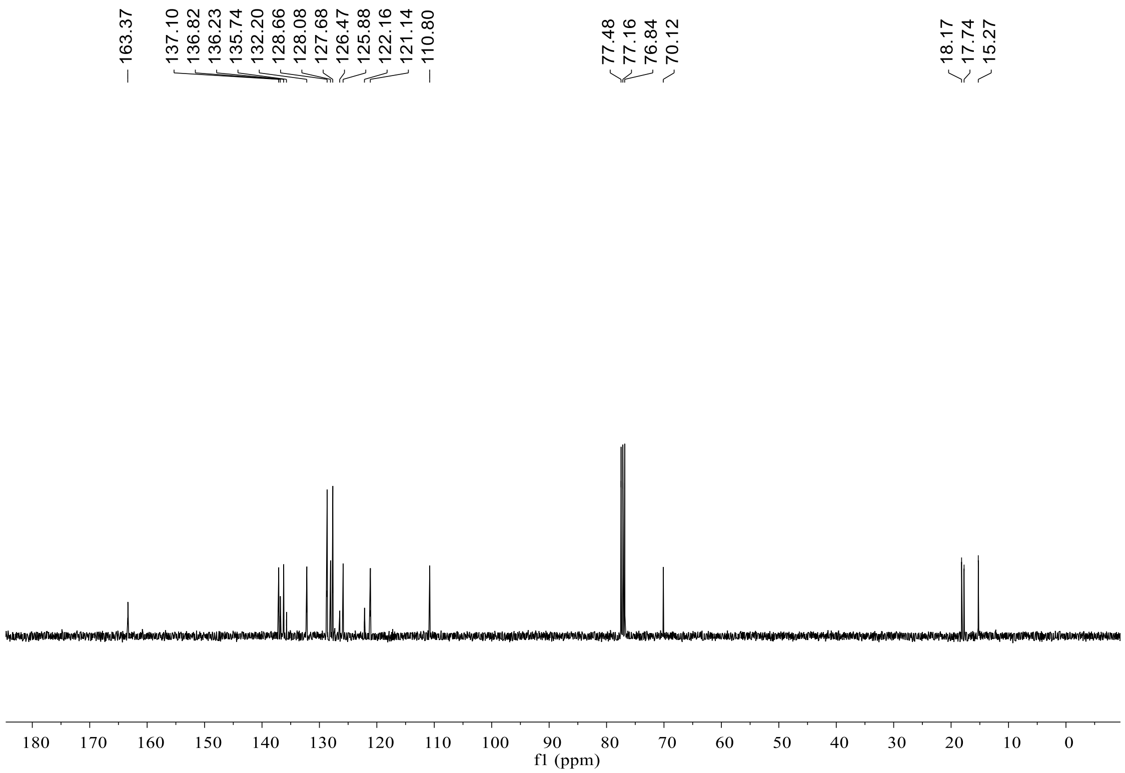

Supplementary Figure 124. ^{13}C NMR spectrum of **3ak**

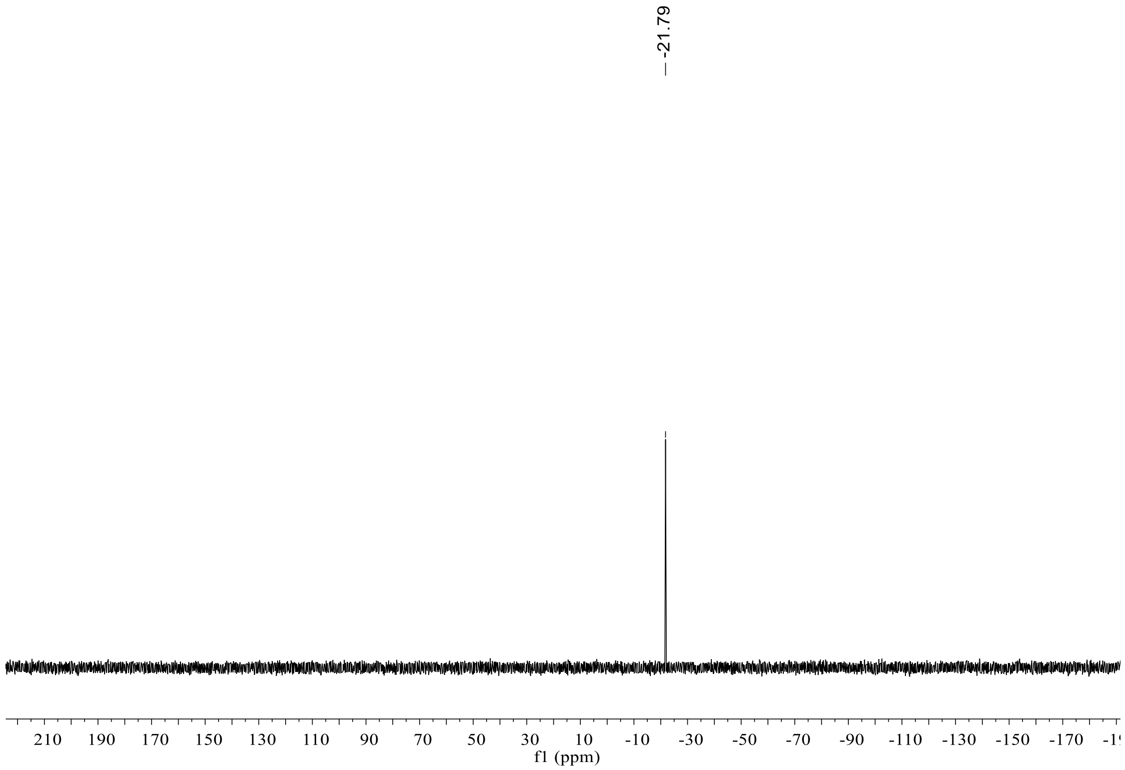

Supplementary Figure 125. ^{29}Si NMR spectrum of **3ak**

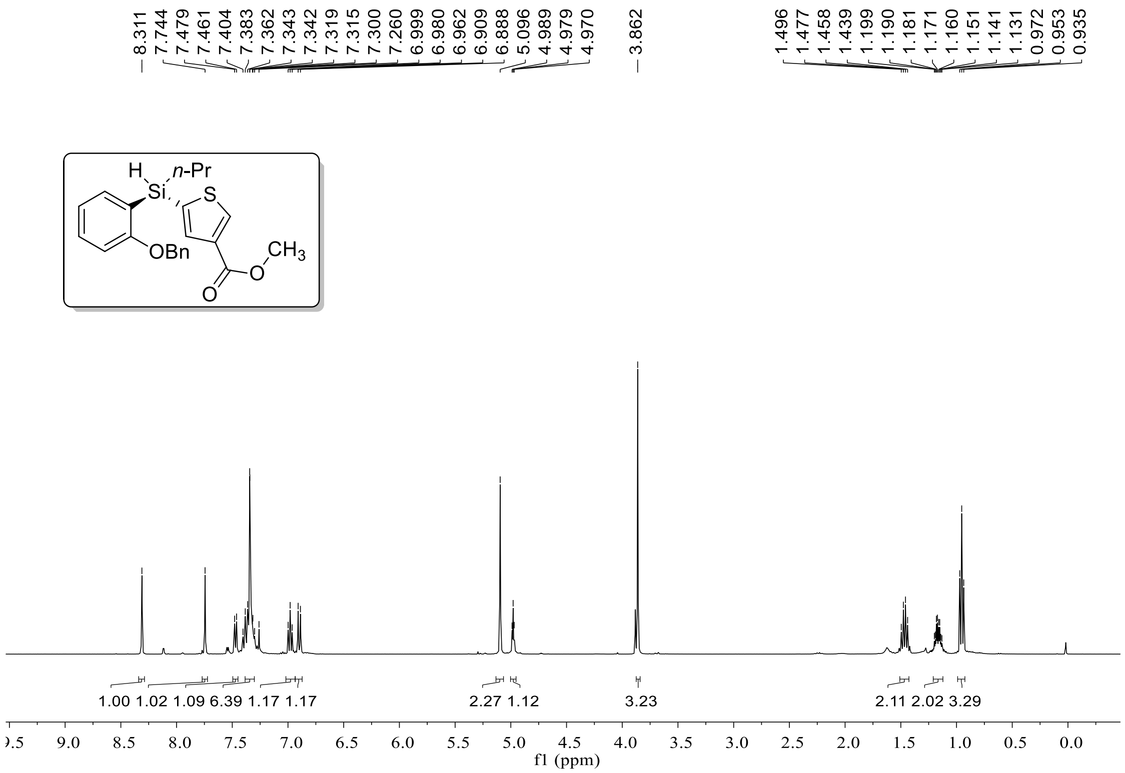

Supplementary Figure 126. ^{1}H NMR spectrum of **3al**

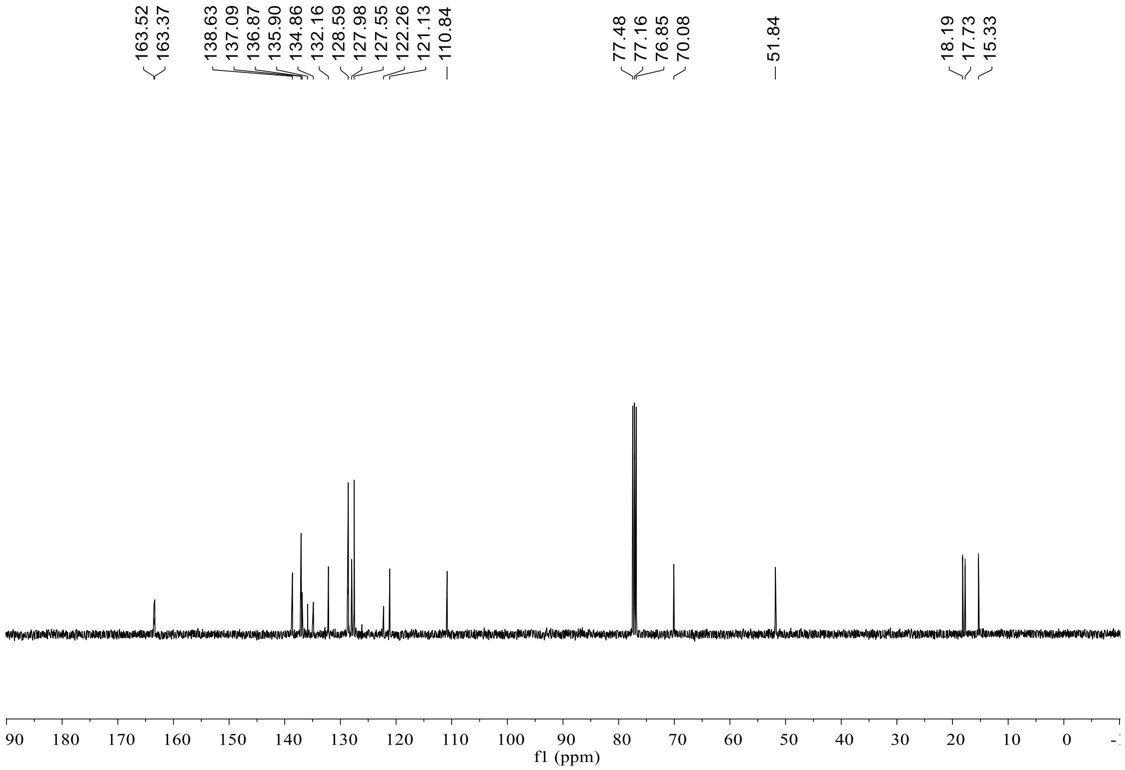

Supplementary Figure 127. ^{13}C NMR spectrum of **3al**

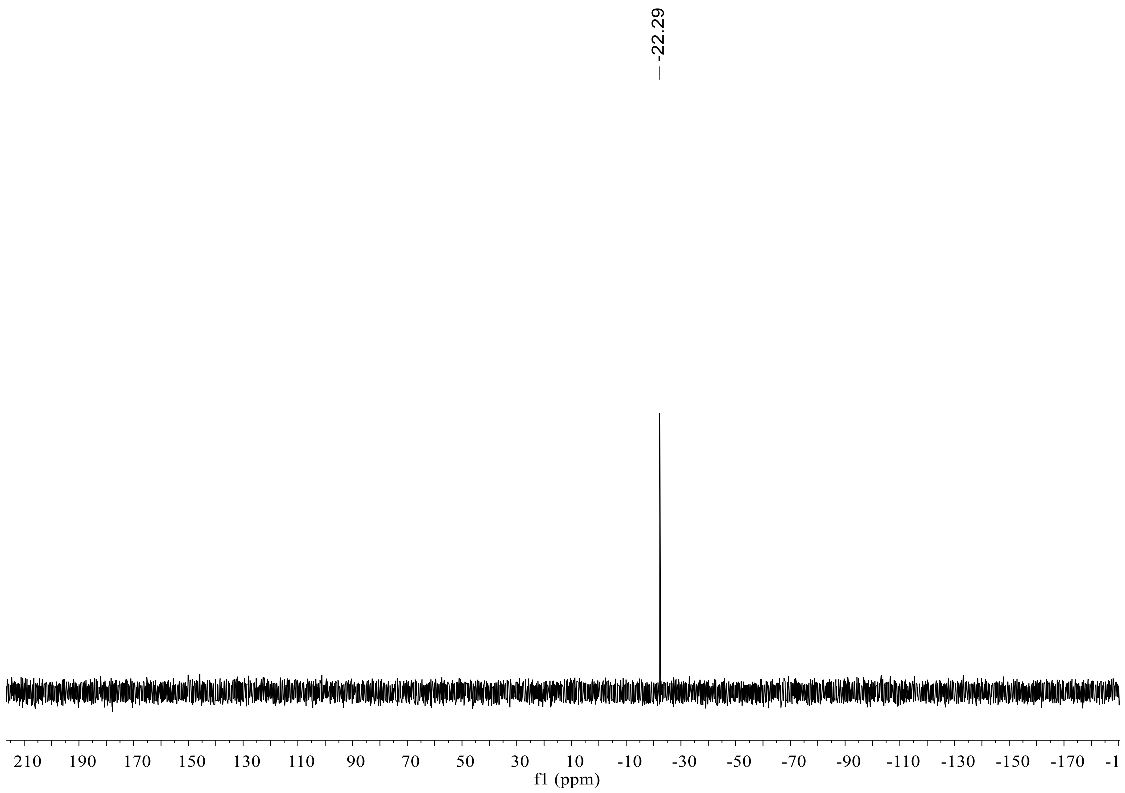

Supplementary Figure 128. ^{29}Si NMR spectrum of **3al**

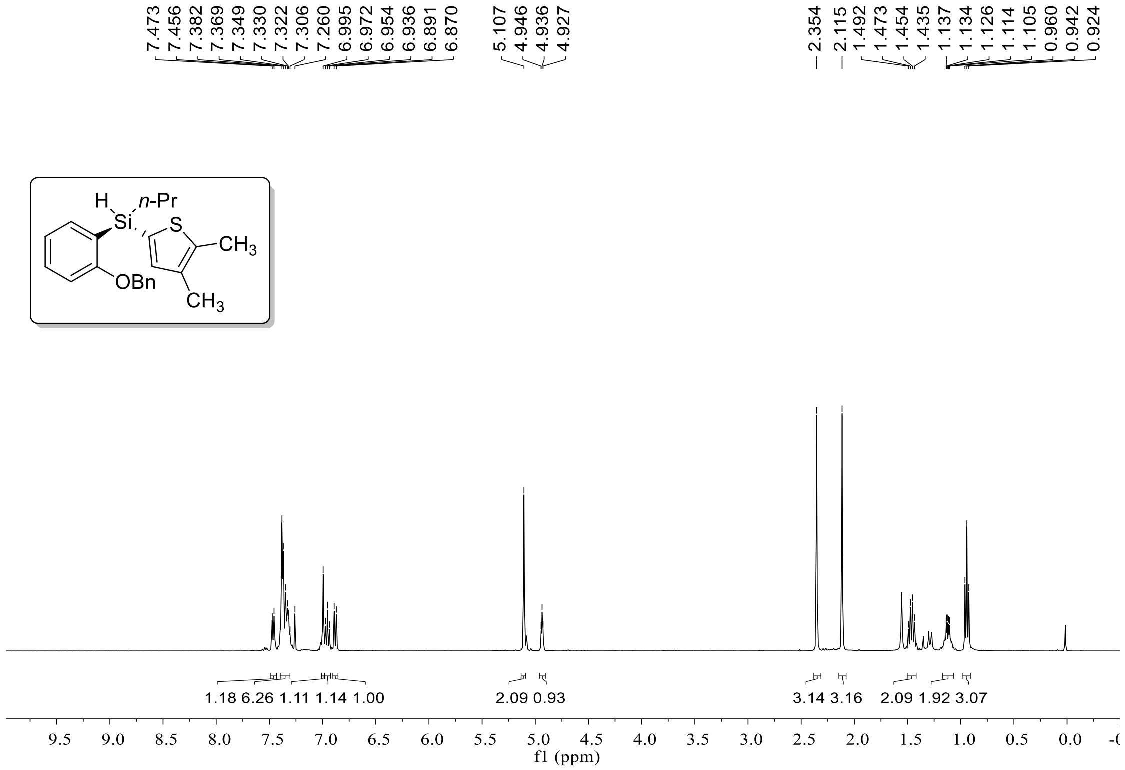

Supplementary Figure 129. ^{1}H NMR spectrum of **3am**

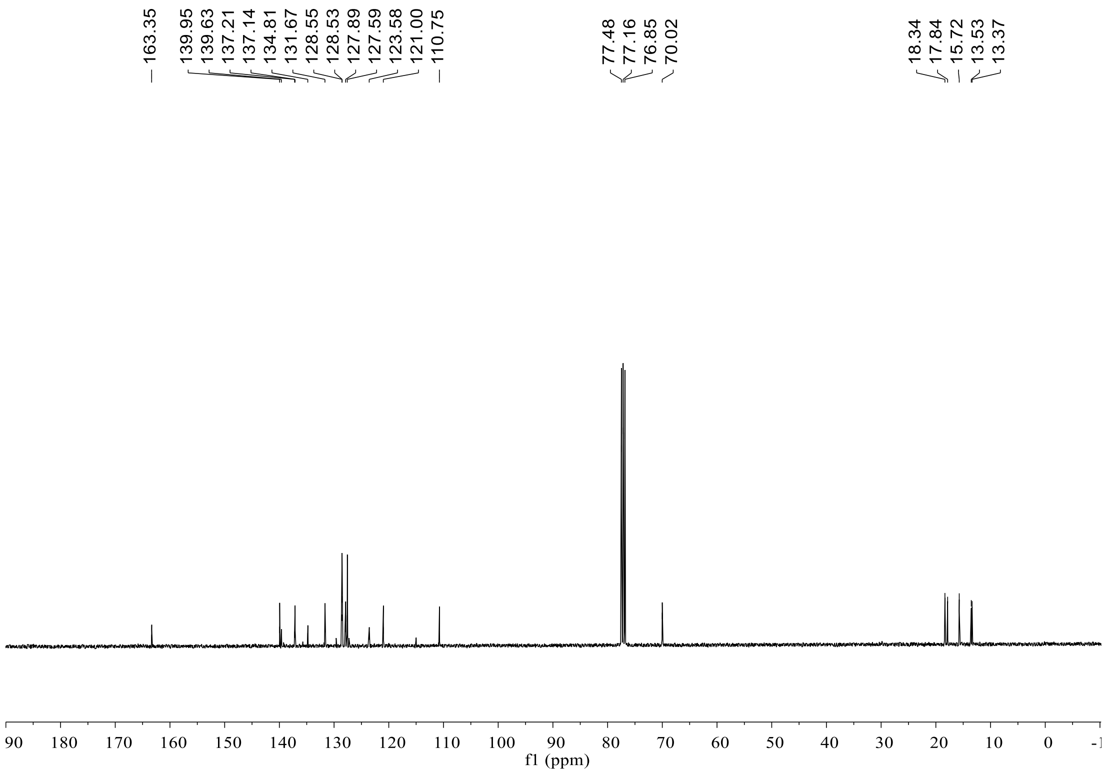

Supplementary Figure 130. ^{13}C NMR spectrum of **3am**

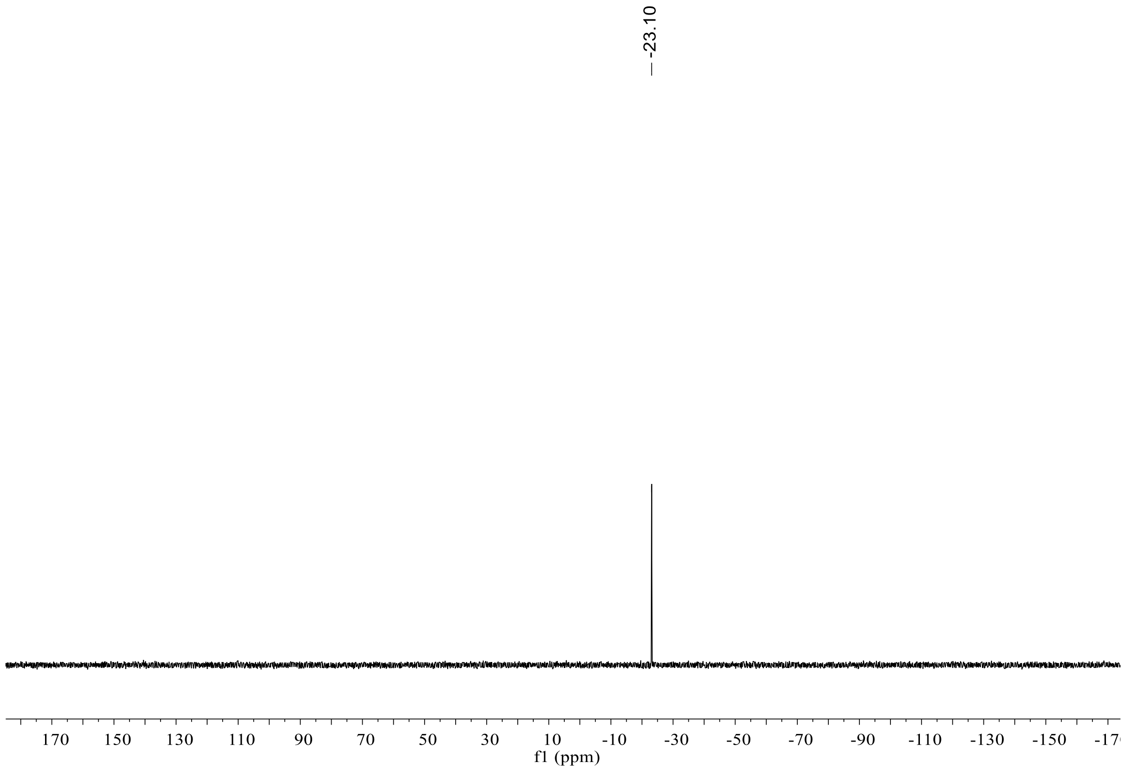

Supplementary Figure 131. ^{29}Si NMR spectrum of **3am**

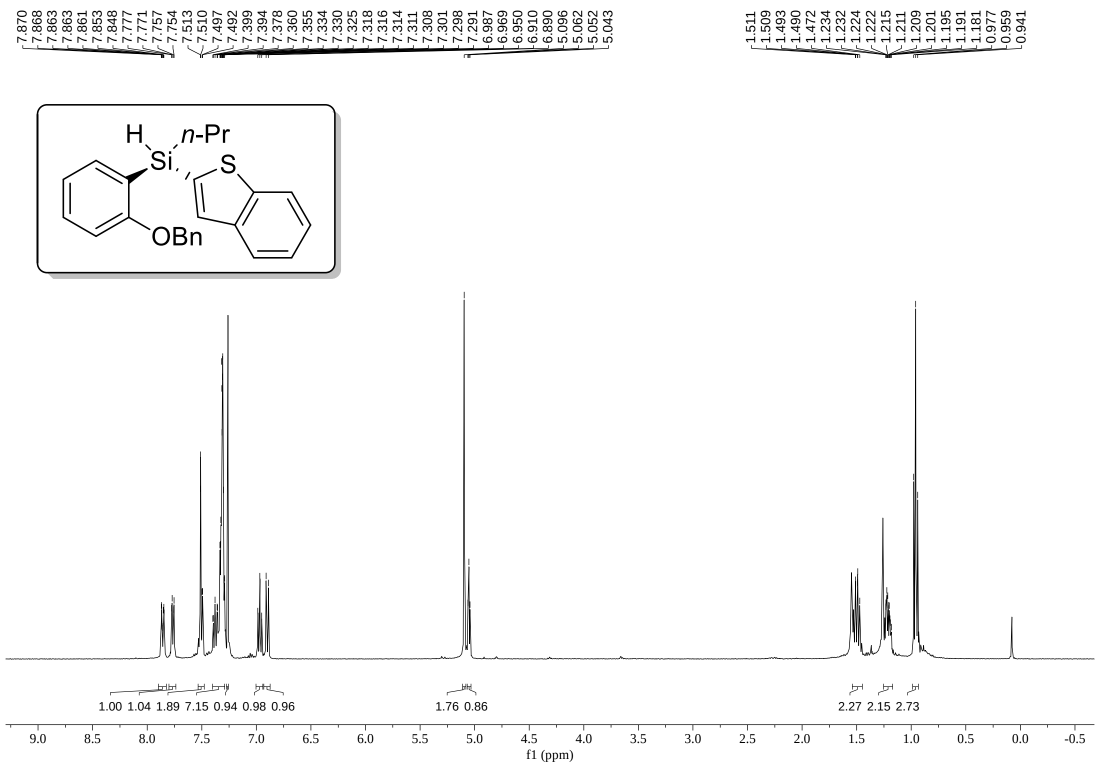

Supplementary Figure 132. ^{1}H NMR spectrum of **3an**

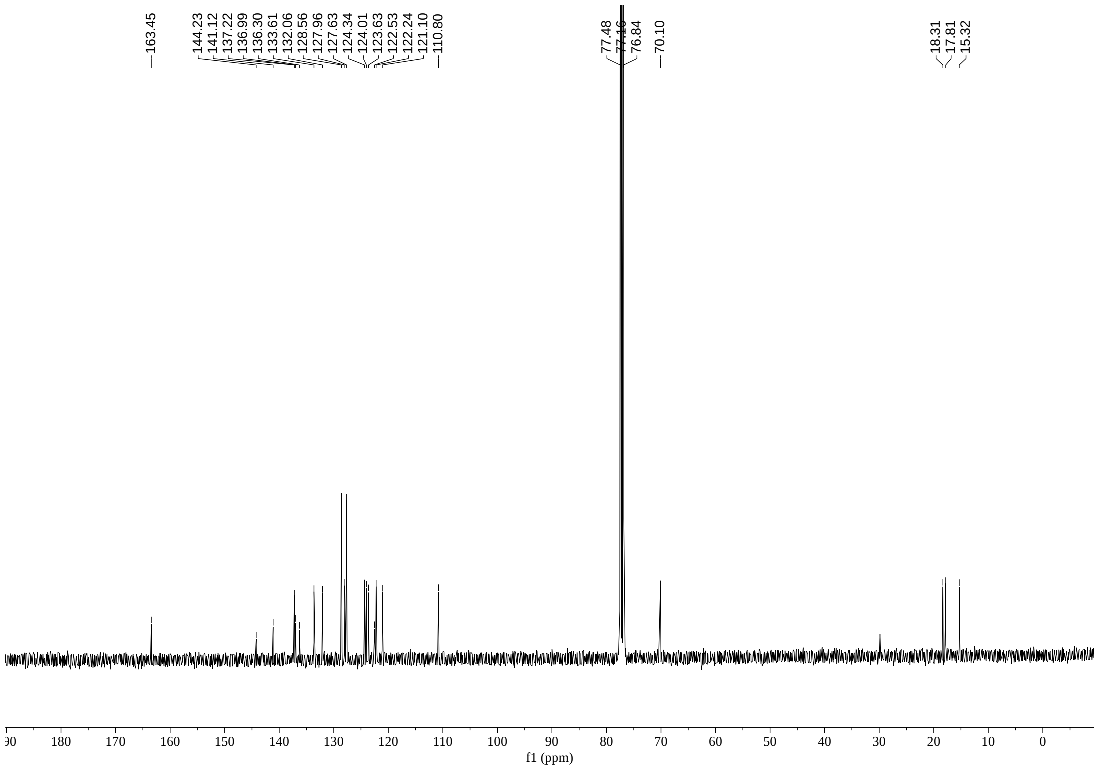

Supplementary Figure 133. ^{13}C NMR spectrum of **3an**

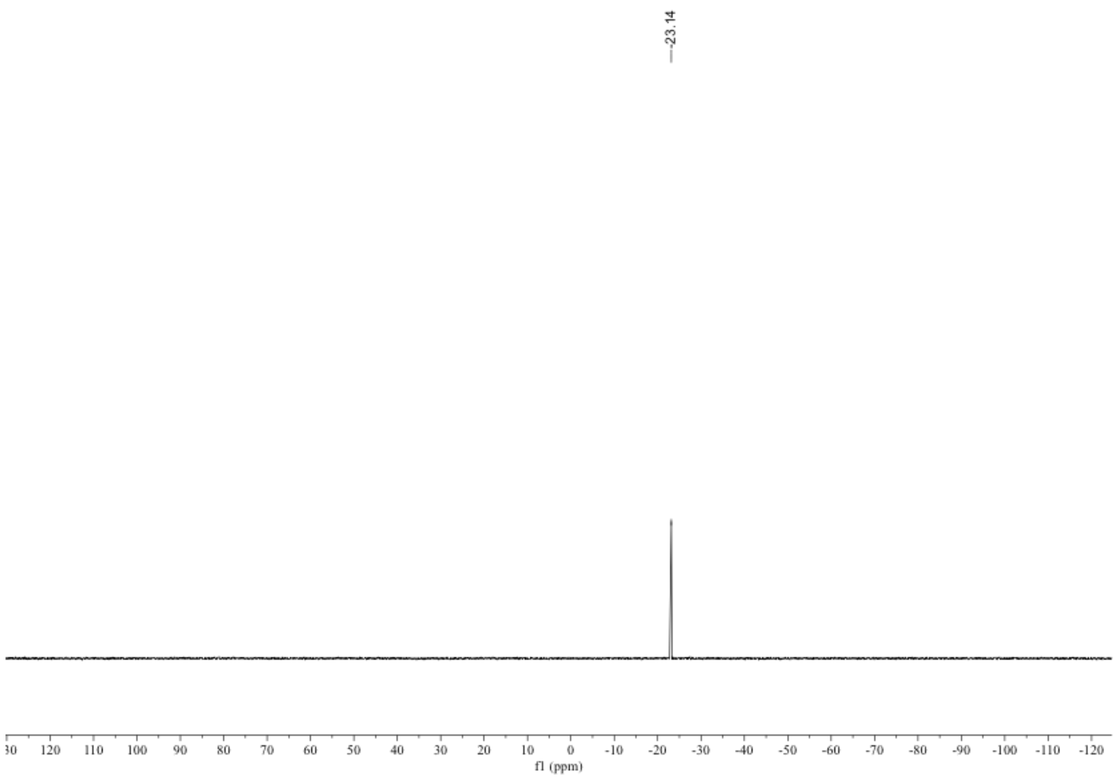

Supplementary Figure 134. ^{29}Si NMR spectrum of **3an**

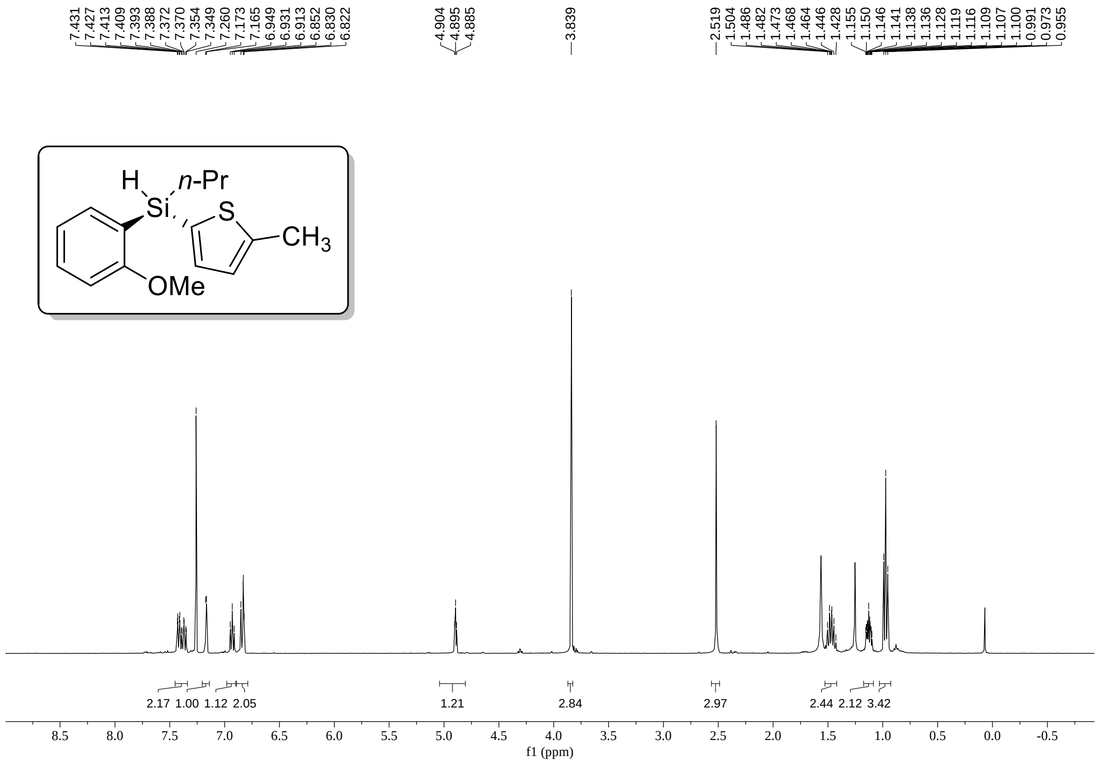

Supplementary Figure 135. ^{1}H NMR spectrum of **3ba**

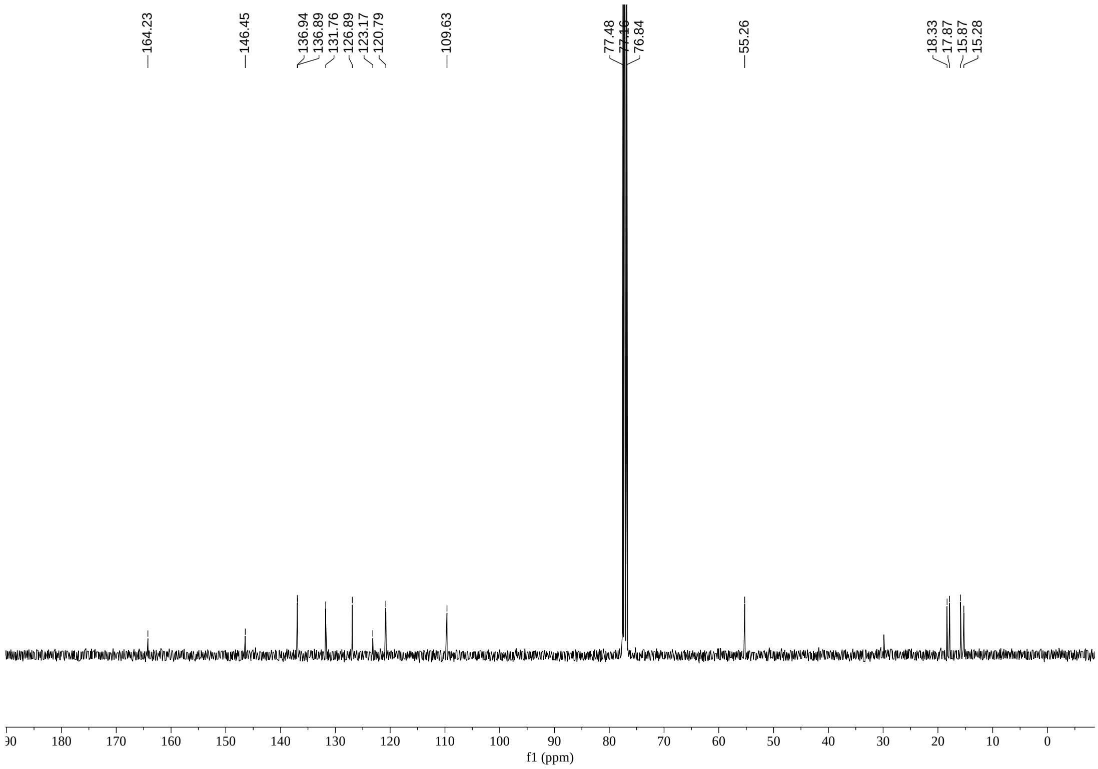

Supplementary Figure 136. ^{13}C NMR spectrum of **3ba**

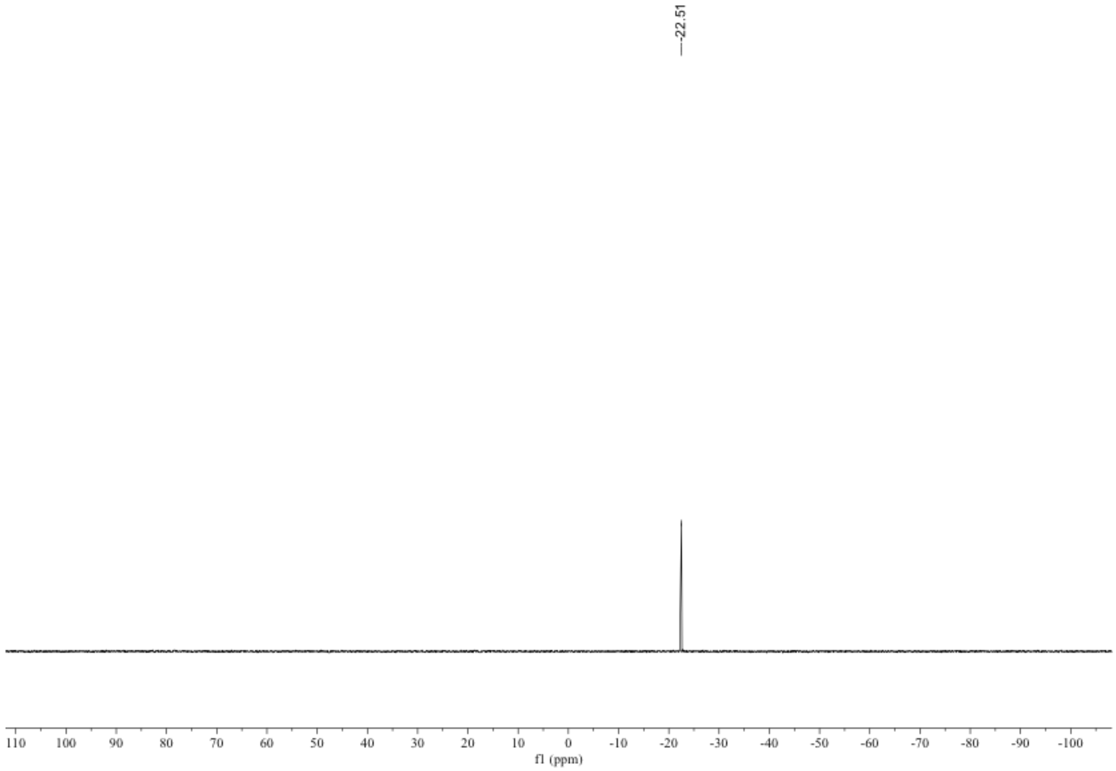

Supplementary Figure 137. ^{29}Si NMR spectrum of **3ba**

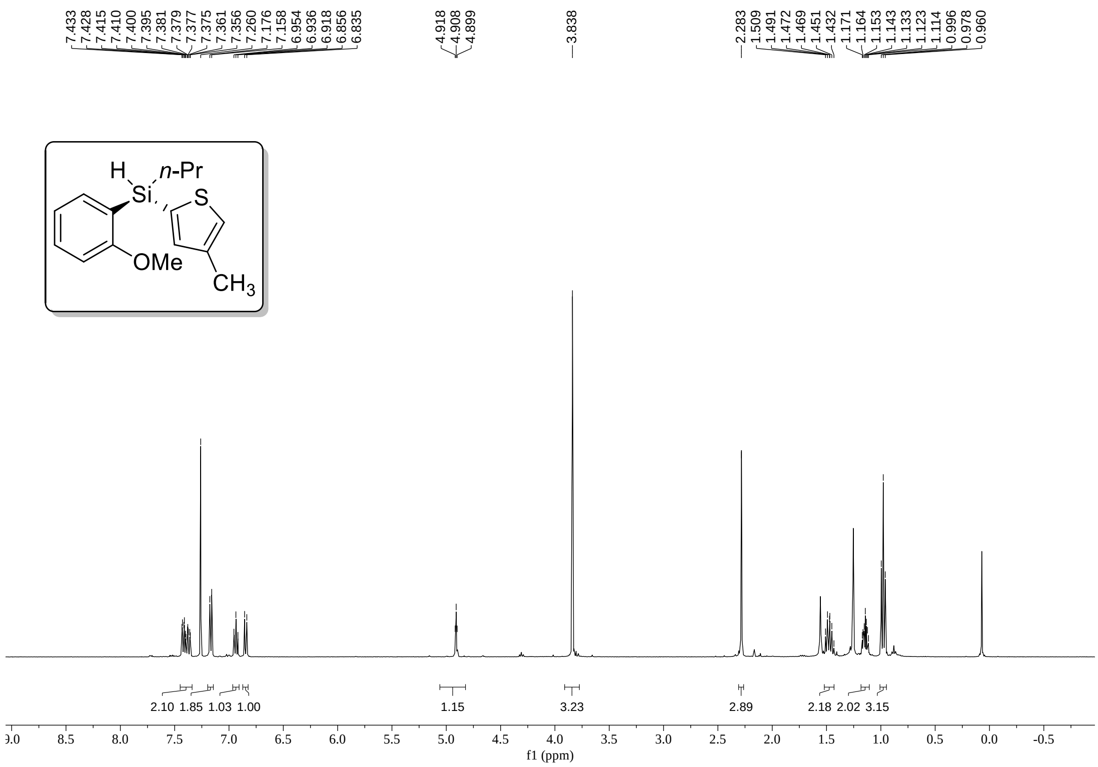

Supplementary Figure 138. ^{1}H NMR spectrum of **3bi**

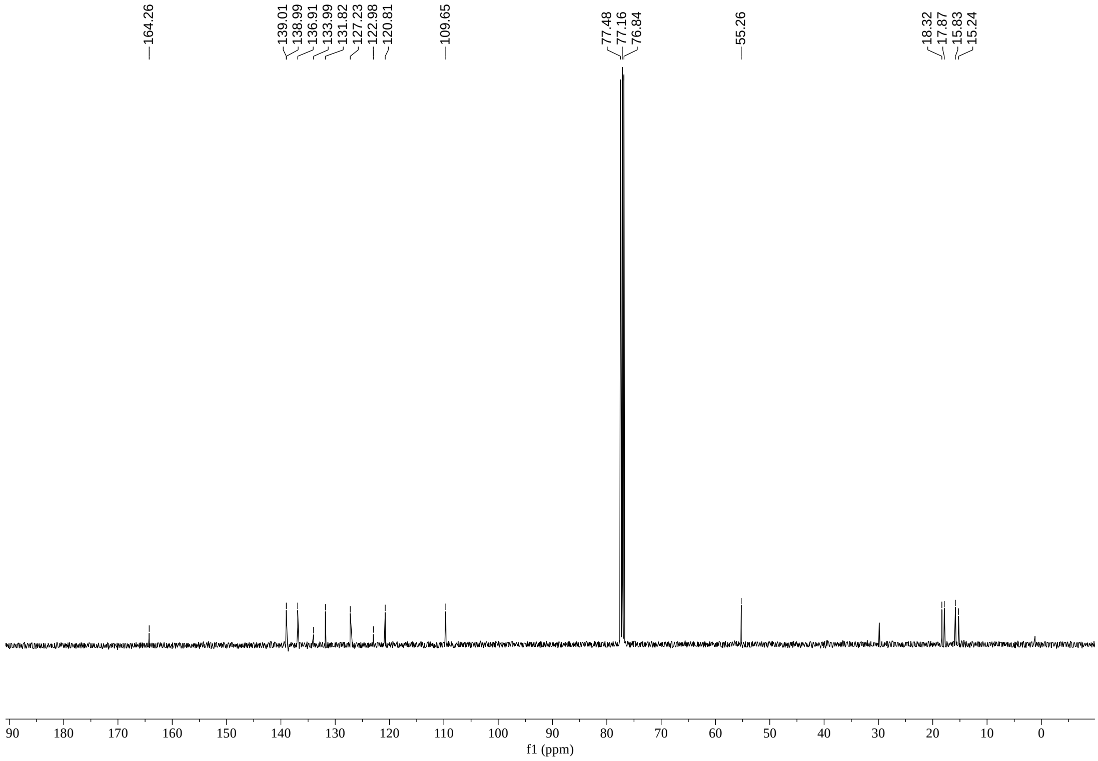

Supplementary Figure 139. ^{13}C NMR spectrum of **3bi**

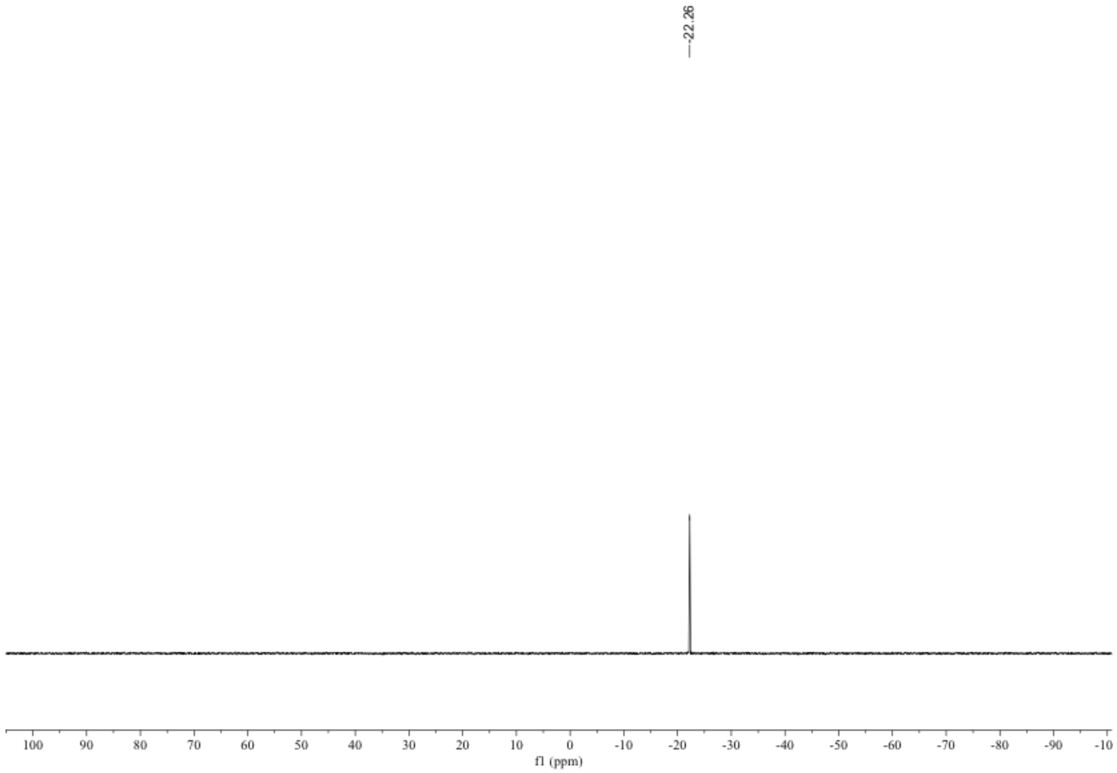

Supplementary Figure 140. ^{29}Si NMR spectrum of **3bi**

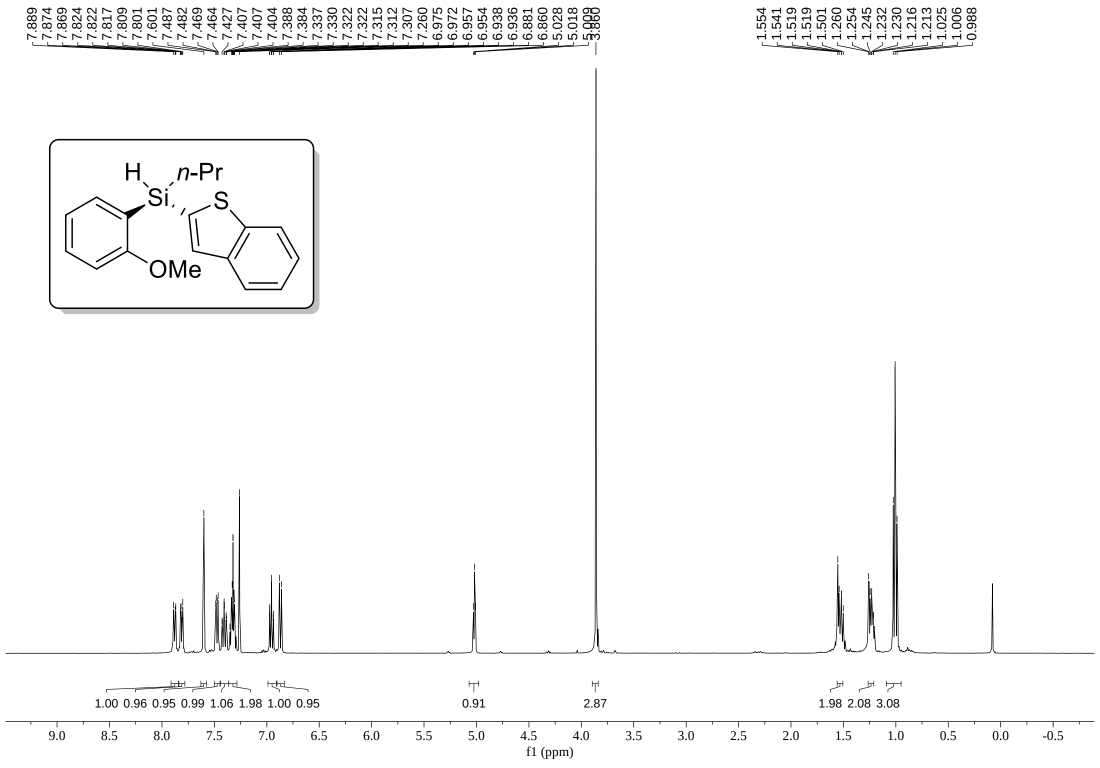

Supplementary Figure 141. ^{1}H NMR spectrum of **3bn**

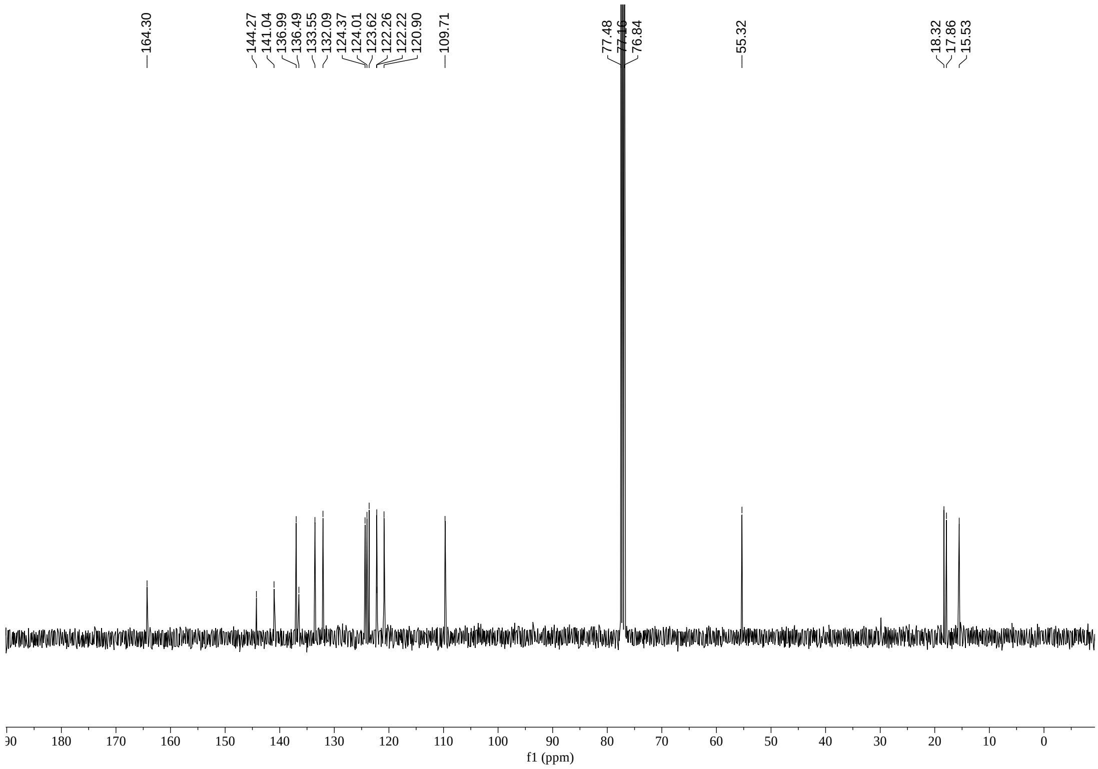

Supplementary Figure 142. ^{13}C NMR spectrum of **3bn**

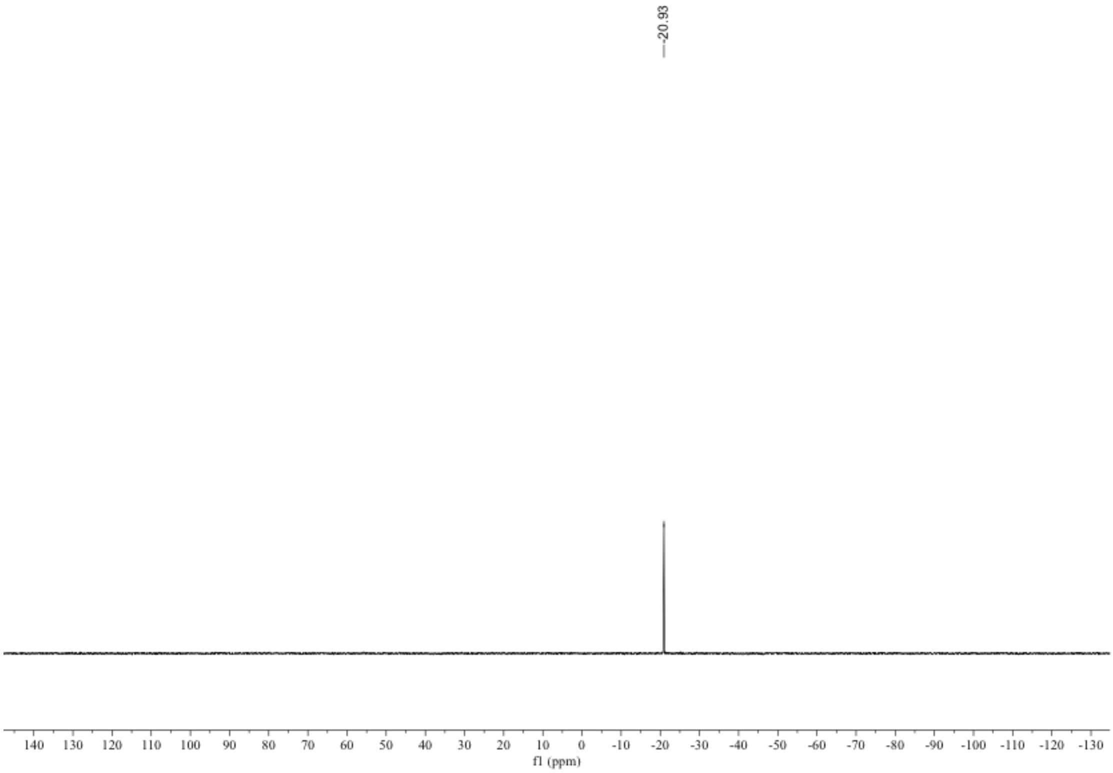

Supplementary Figure 143. ^{29}Si NMR spectrum of **3bn**

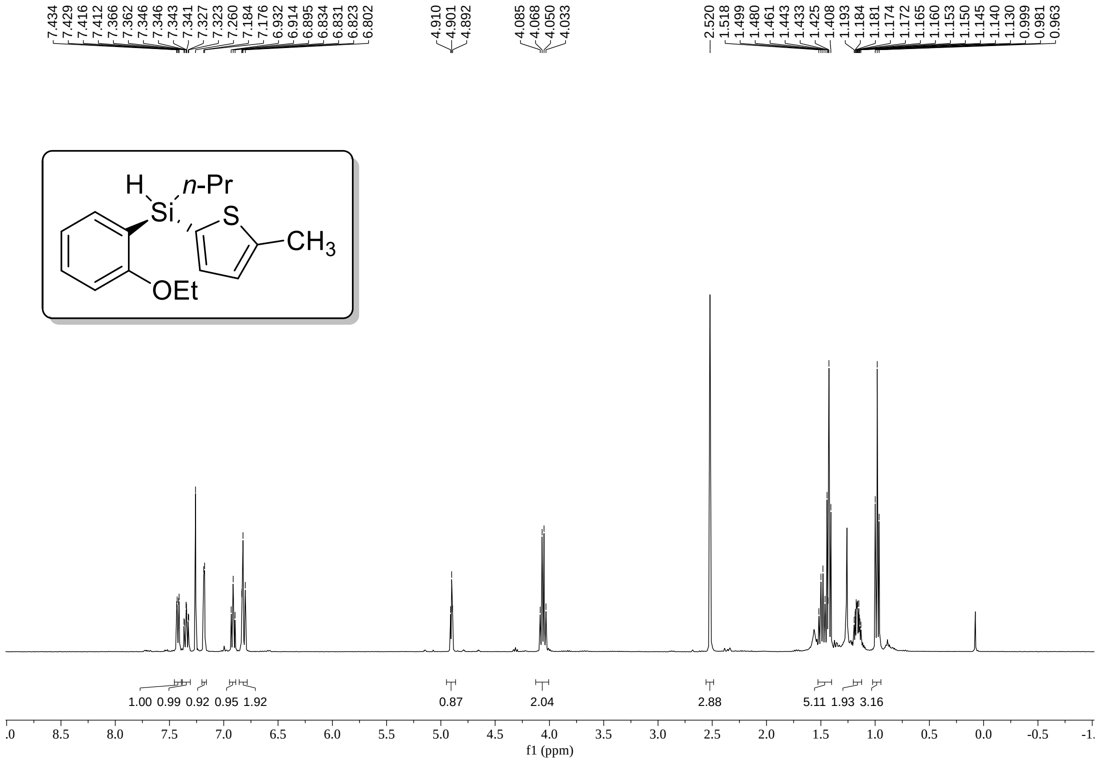

Supplementary Figure 144. ^{1}H NMR spectrum of **3ca**

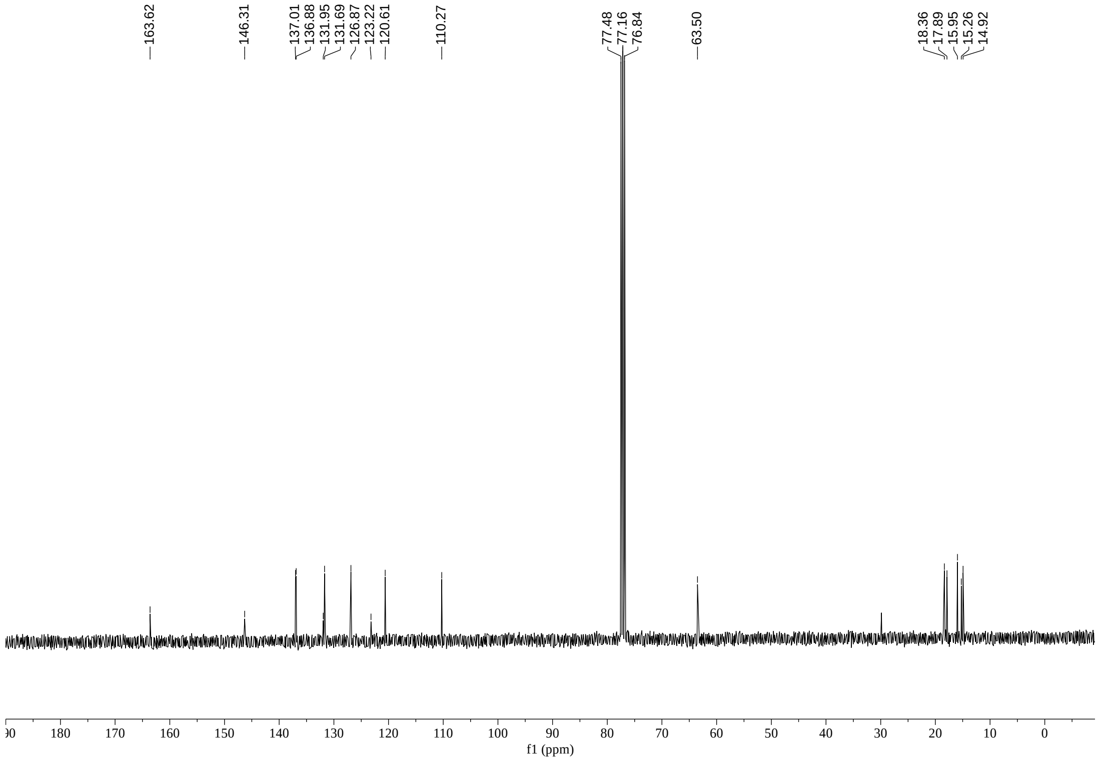

Supplementary Figure 145. ^{13}C NMR spectrum of **3ca**

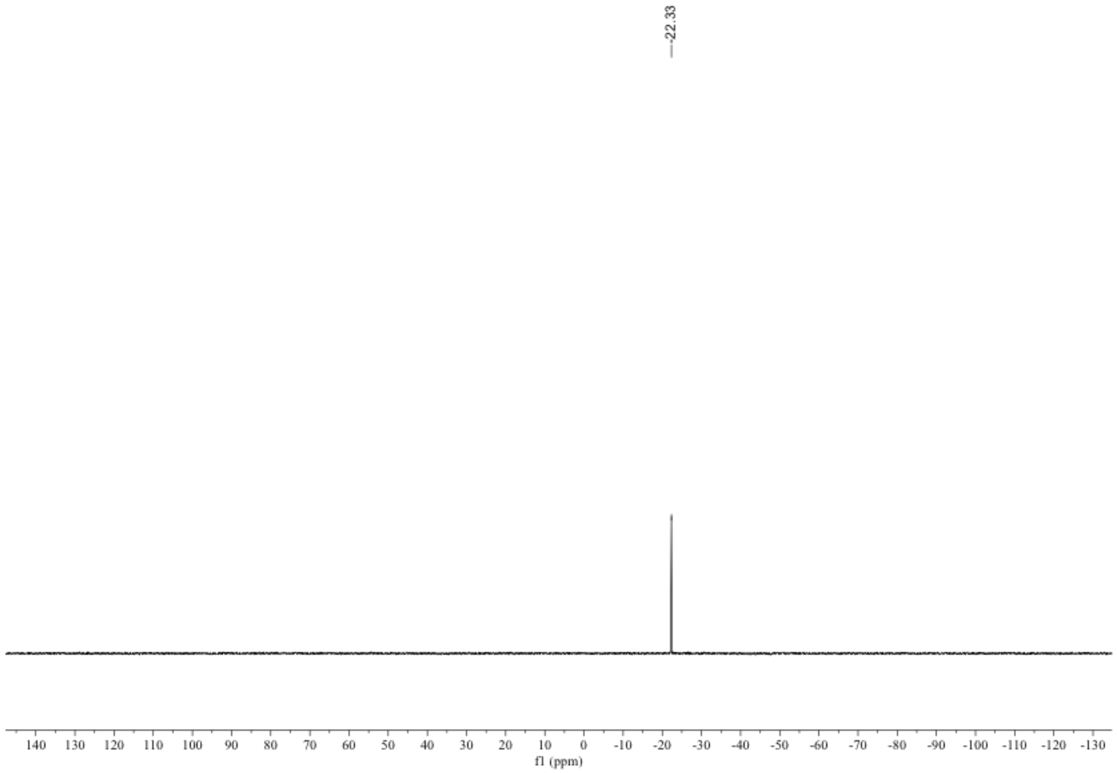

Supplementary Figure 146. ^{29}Si NMR spectrum of **3ca**

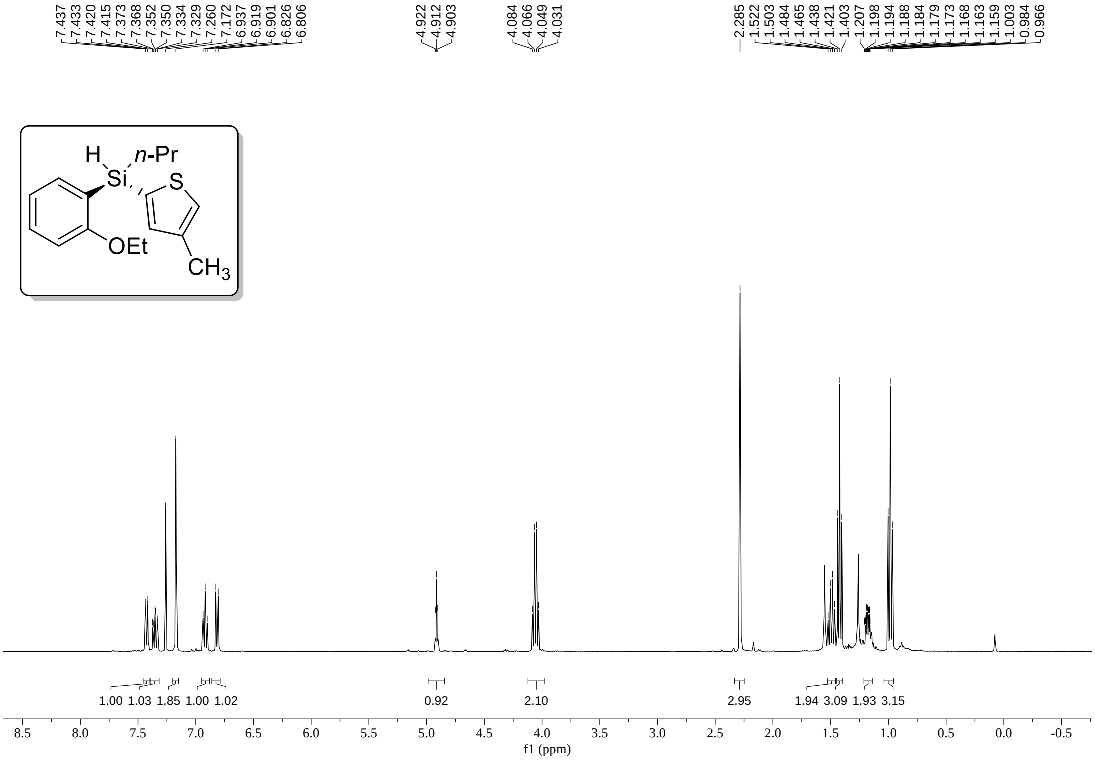

Supplementary Figure 147. ^{1}H NMR spectrum of **3ci**

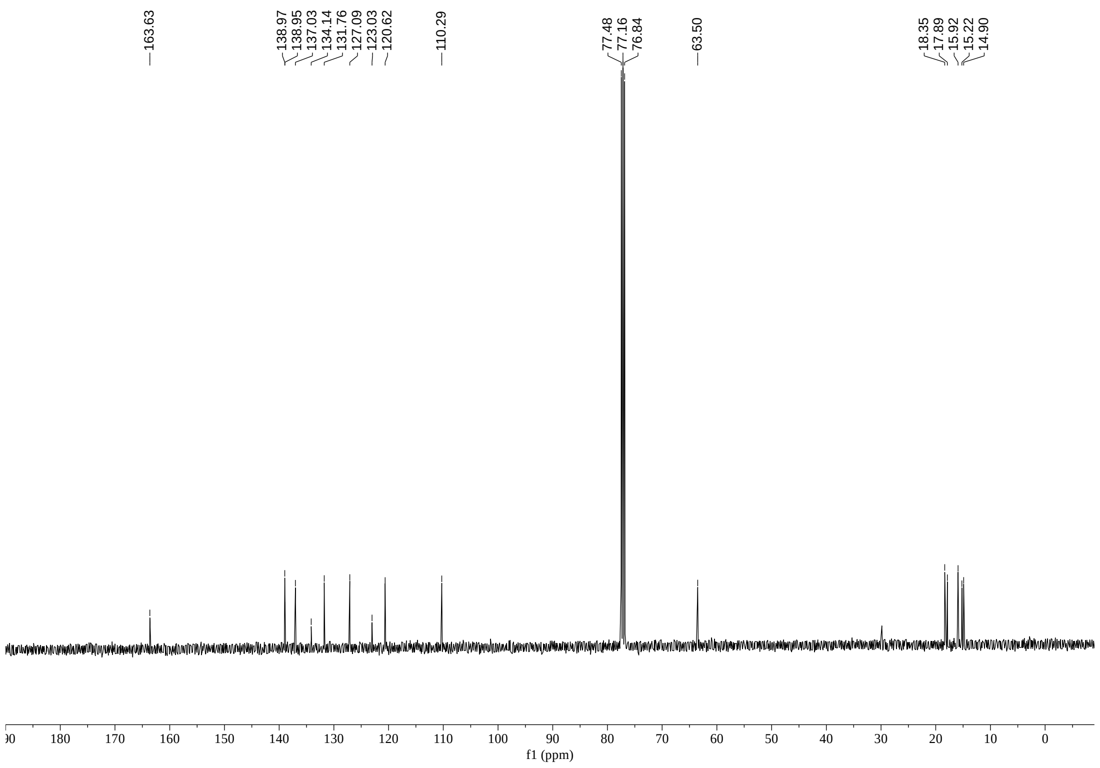

Supplementary Figure 148. ^{13}C NMR spectrum of **3ci**

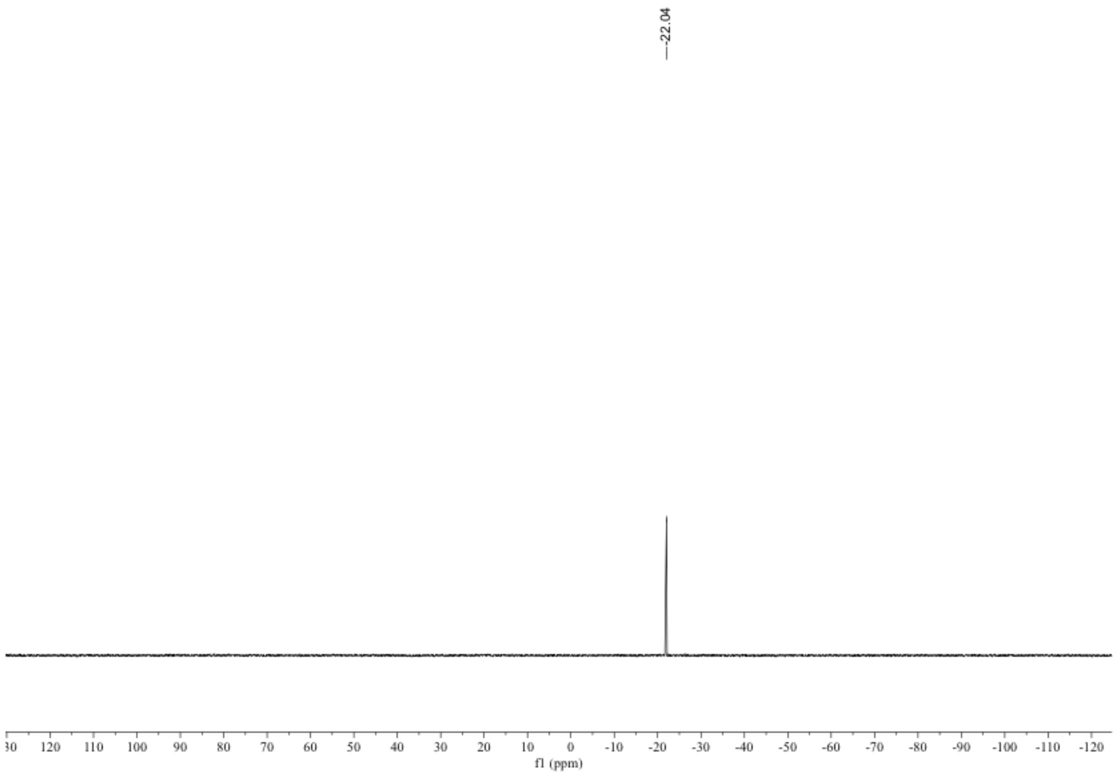

Supplementary Figure 149. ^{29}Si NMR spectrum of **3ci**

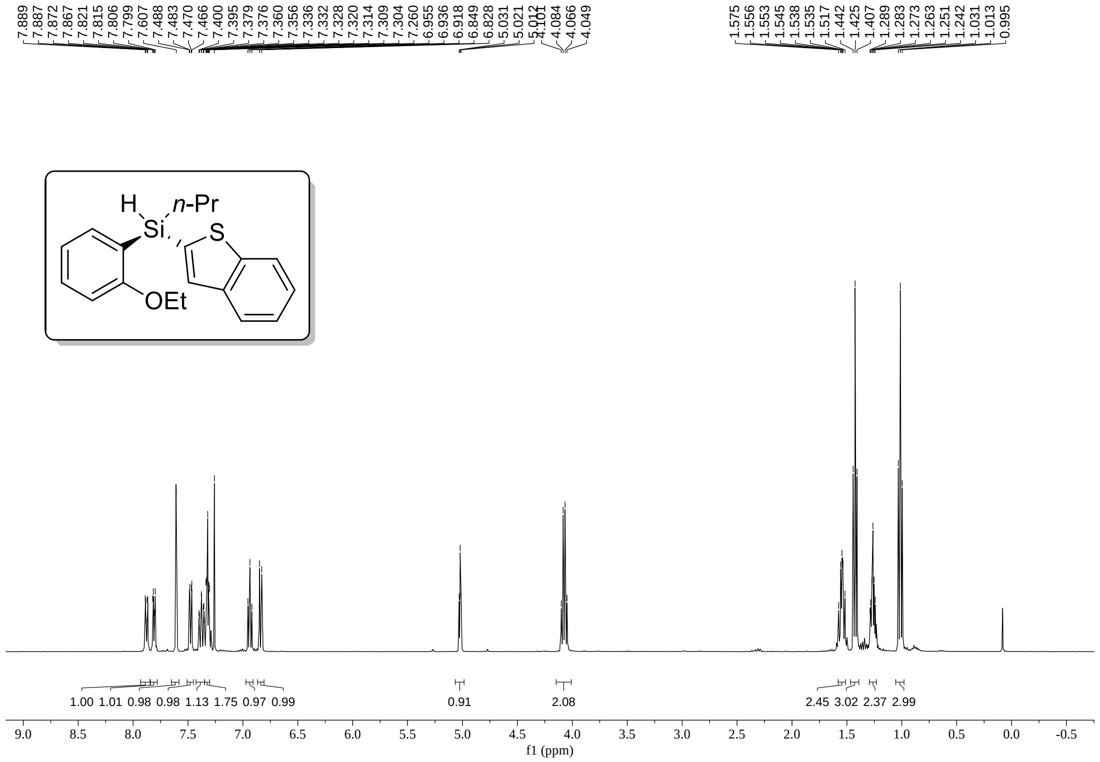

Supplementary Figure 150. ^{1}H NMR spectrum of **3cn**

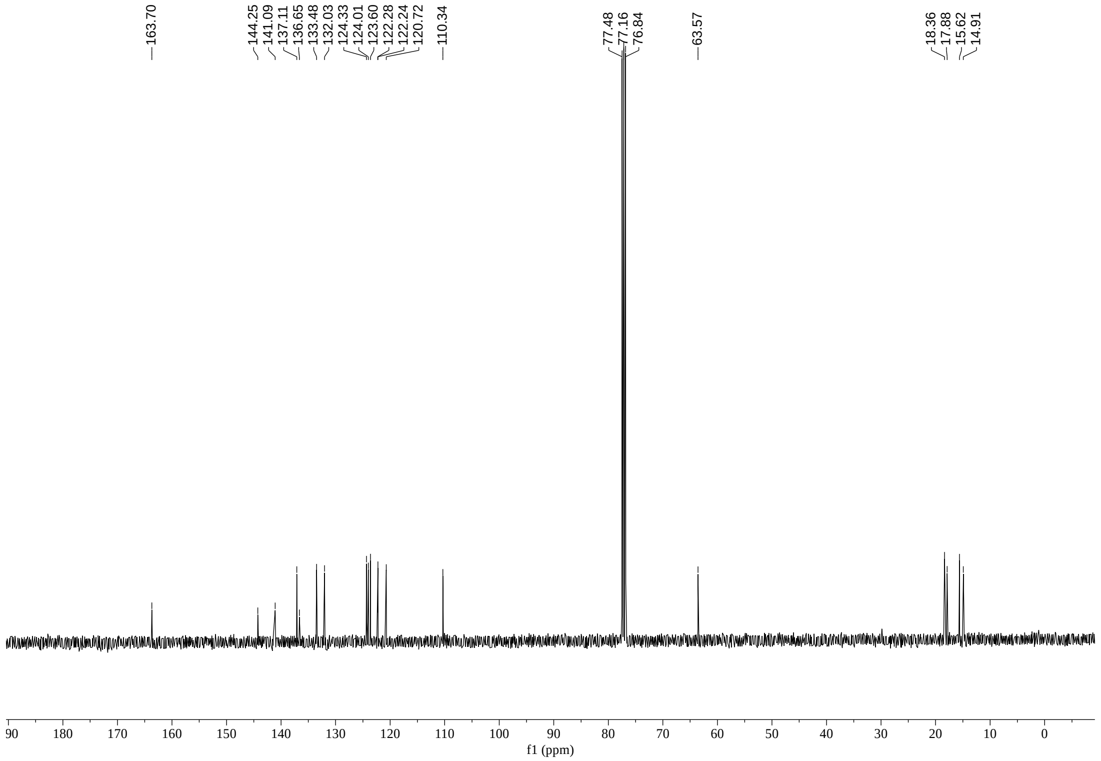

Supplementary Figure 151. ^{13}C NMR spectrum of **3cn**

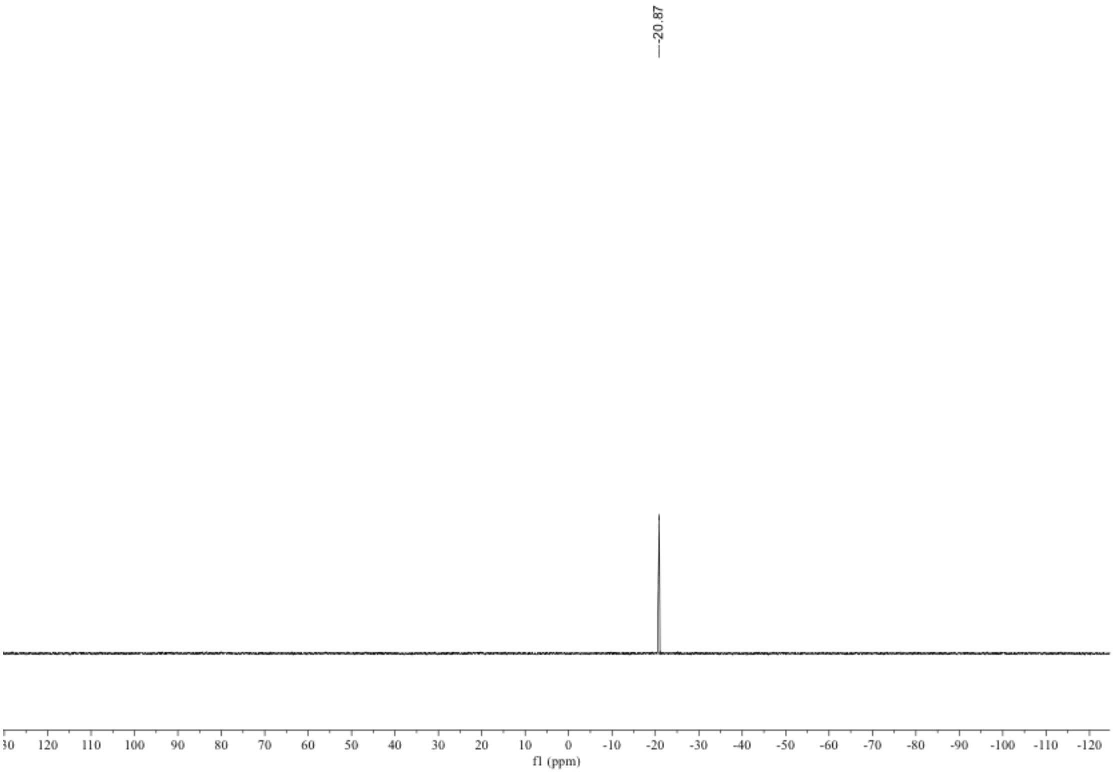

Supplementary Figure 152. ^{29}Si NMR spectrum of **3cn**

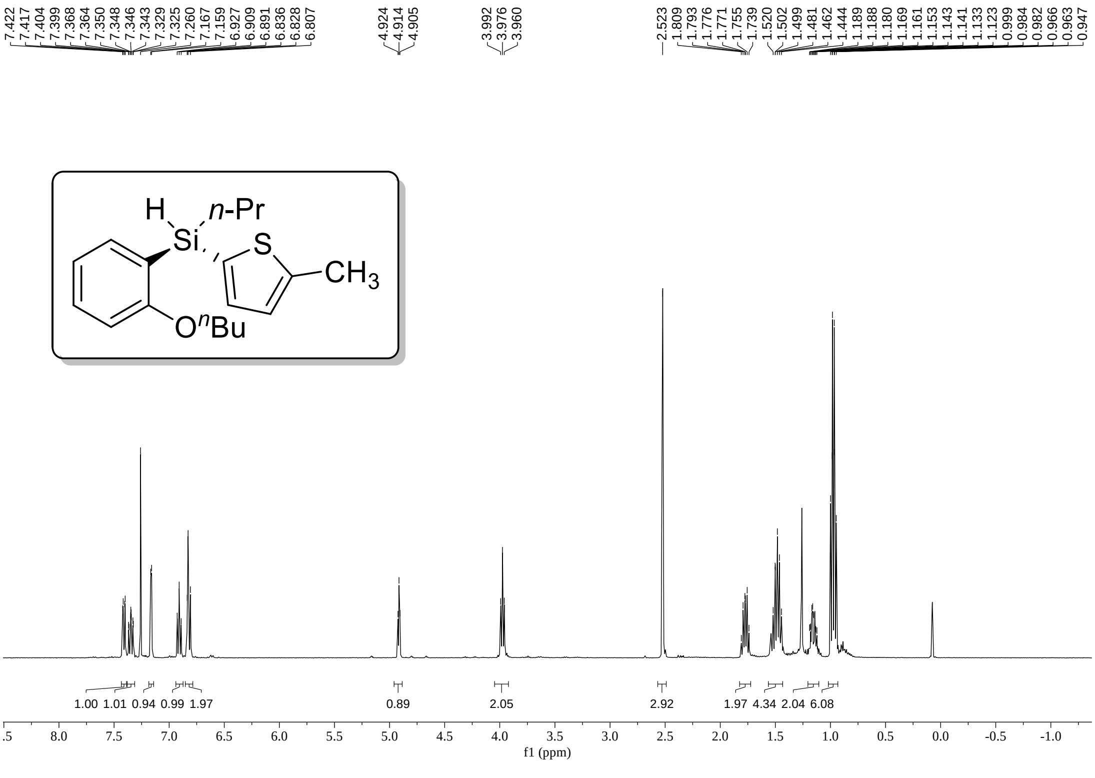

Supplementary Figure 153. ^{1}H NMR spectrum of **3da**

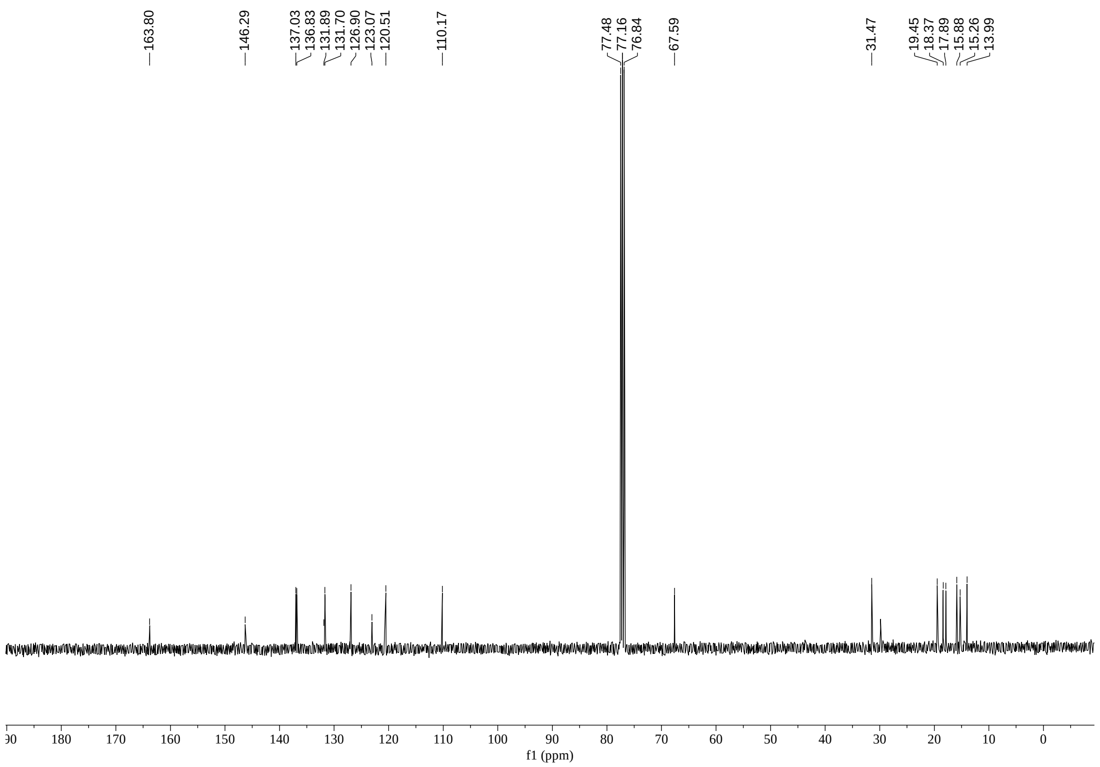

Supplementary Figure 154. ^{13}C NMR spectrum of **3da**

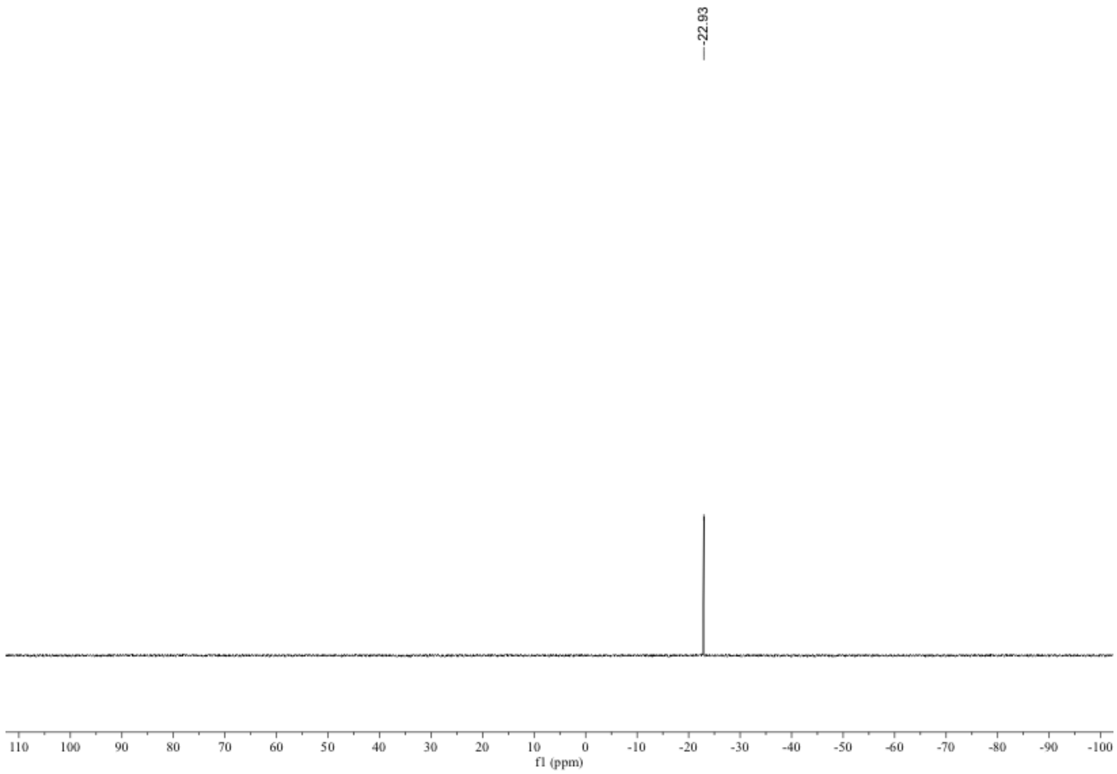

Supplementary Figure 155. ^{29}Si NMR spectrum of **3da**

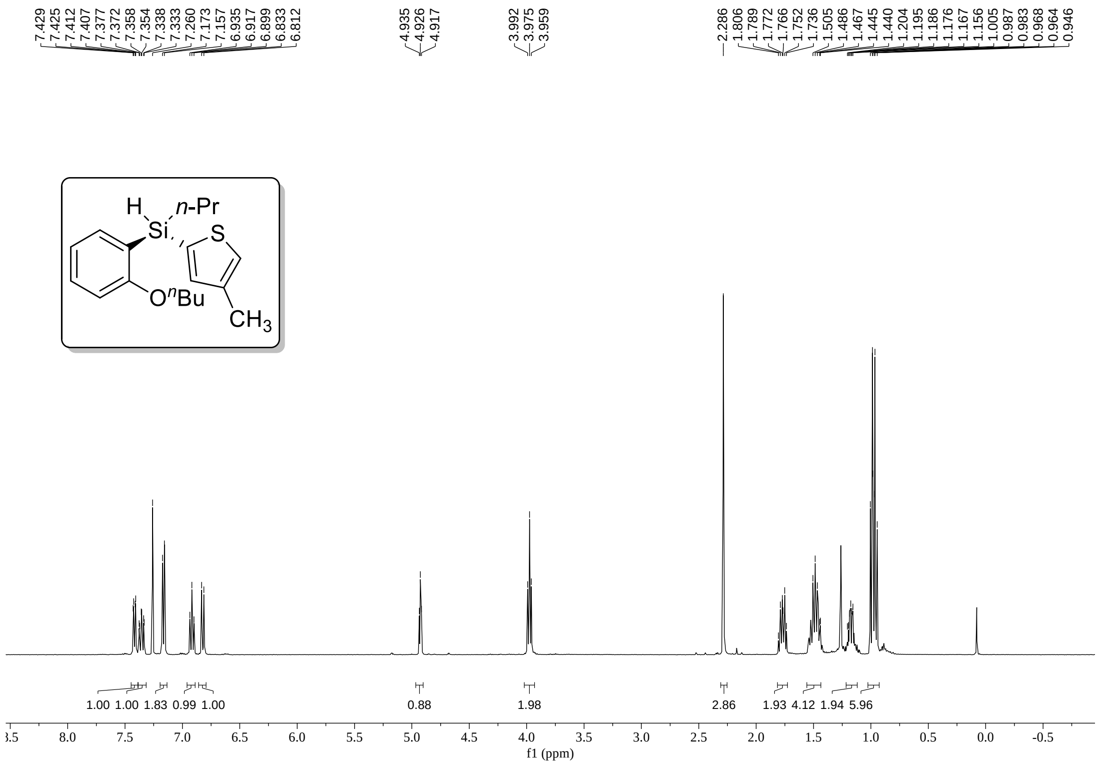

Supplementary Figure 156. ^{1}H NMR spectrum of **3di**

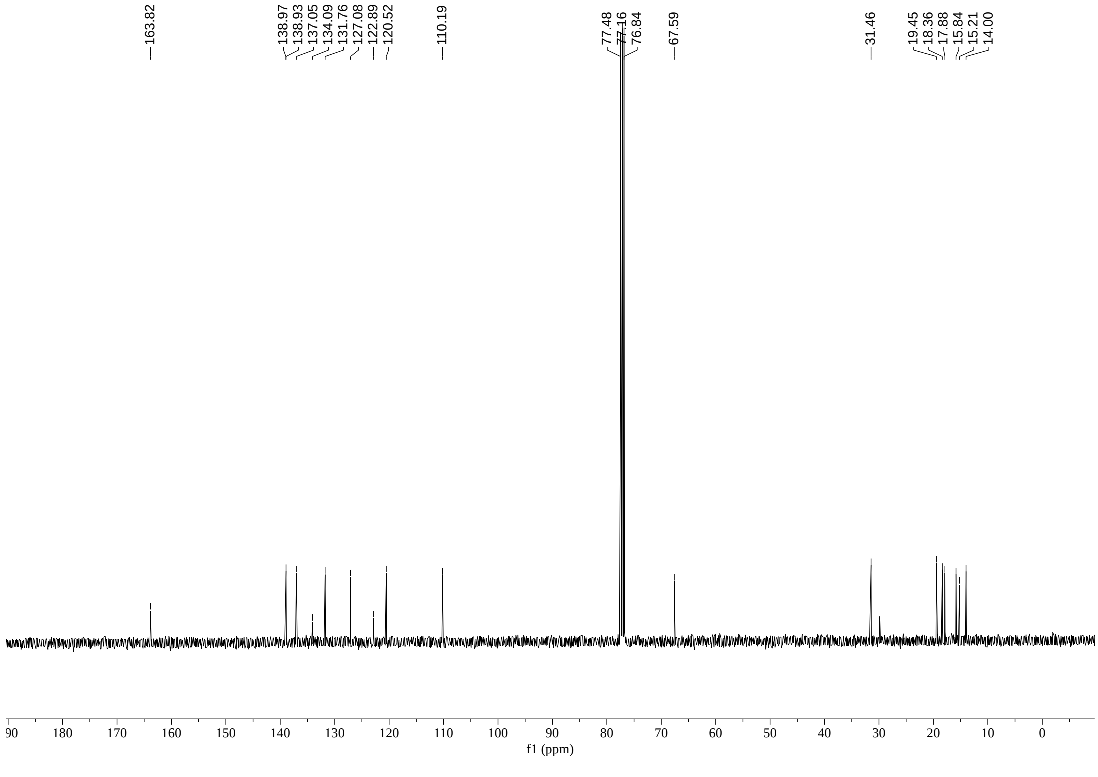

Supplementary Figure 157. ^{13}C NMR spectrum of **3di**

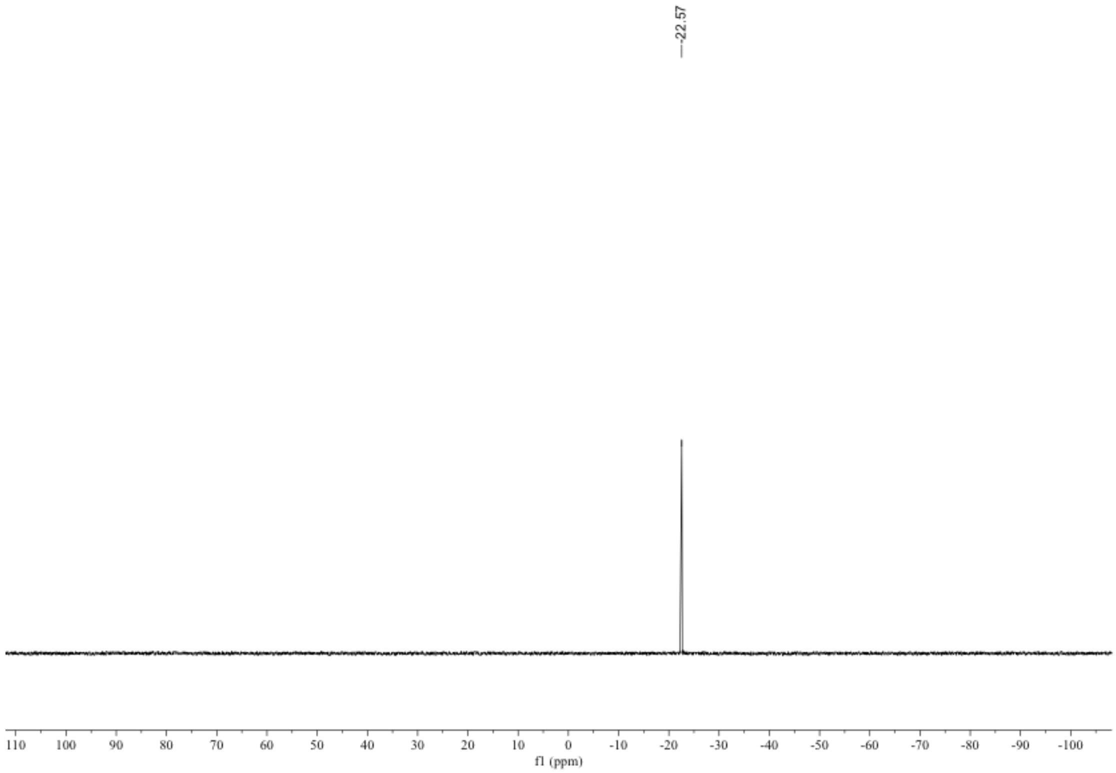

Supplementary Figure 158. ^{29}Si NMR spectrum of **3di**

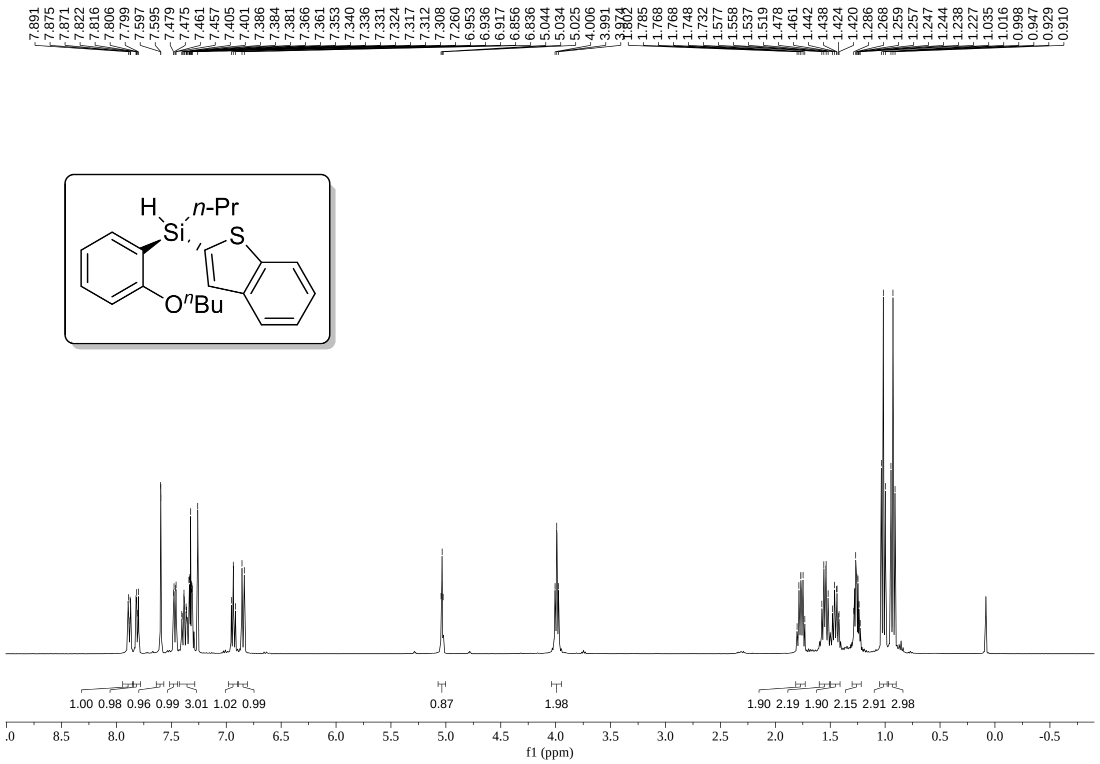

Supplementary Figure 159. ^{1}H NMR spectrum of **3dn**

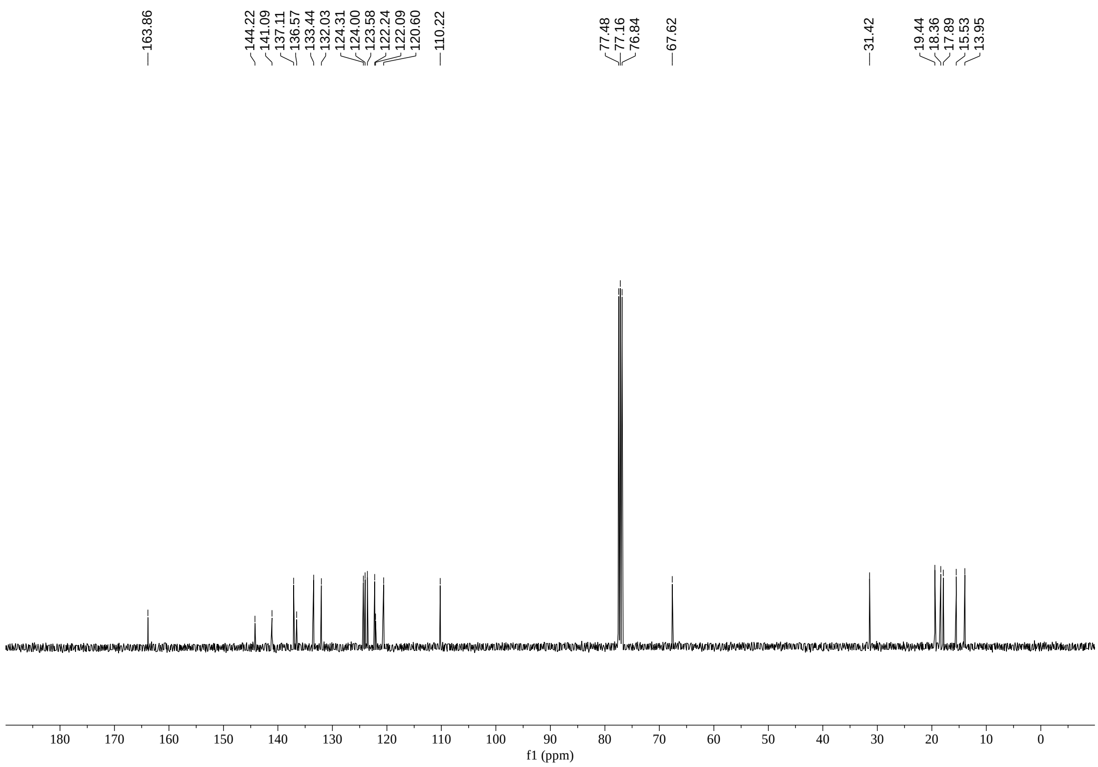

Supplementary Figure 160. ^{13}C NMR spectrum of **3dn**

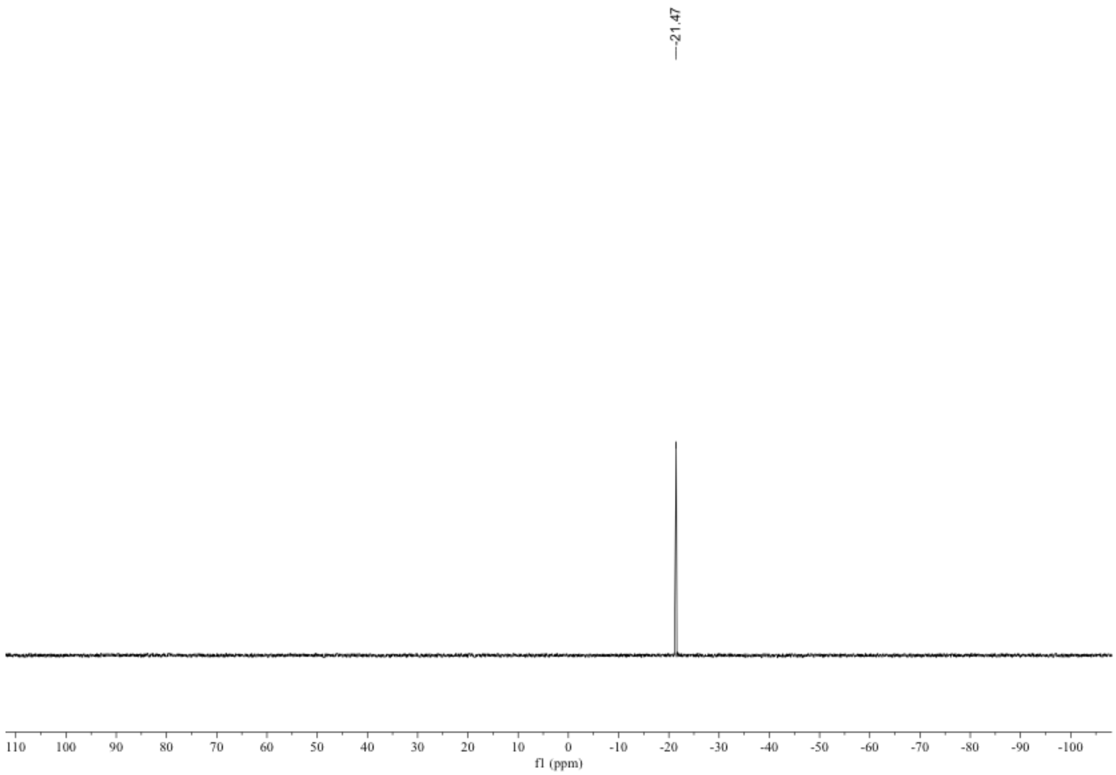

Supplementary Figure 161. ^{29}Si NMR spectrum of **3dn**

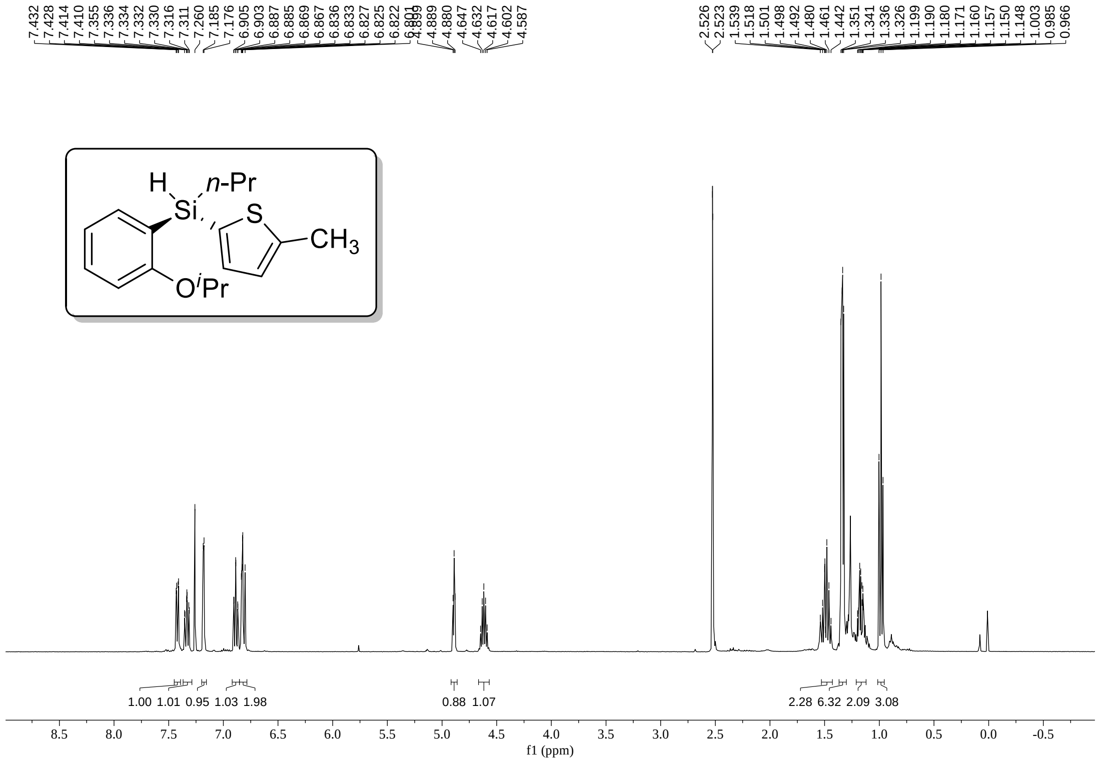

Supplementary Figure 162. ^{1}H NMR spectrum of **3ea**

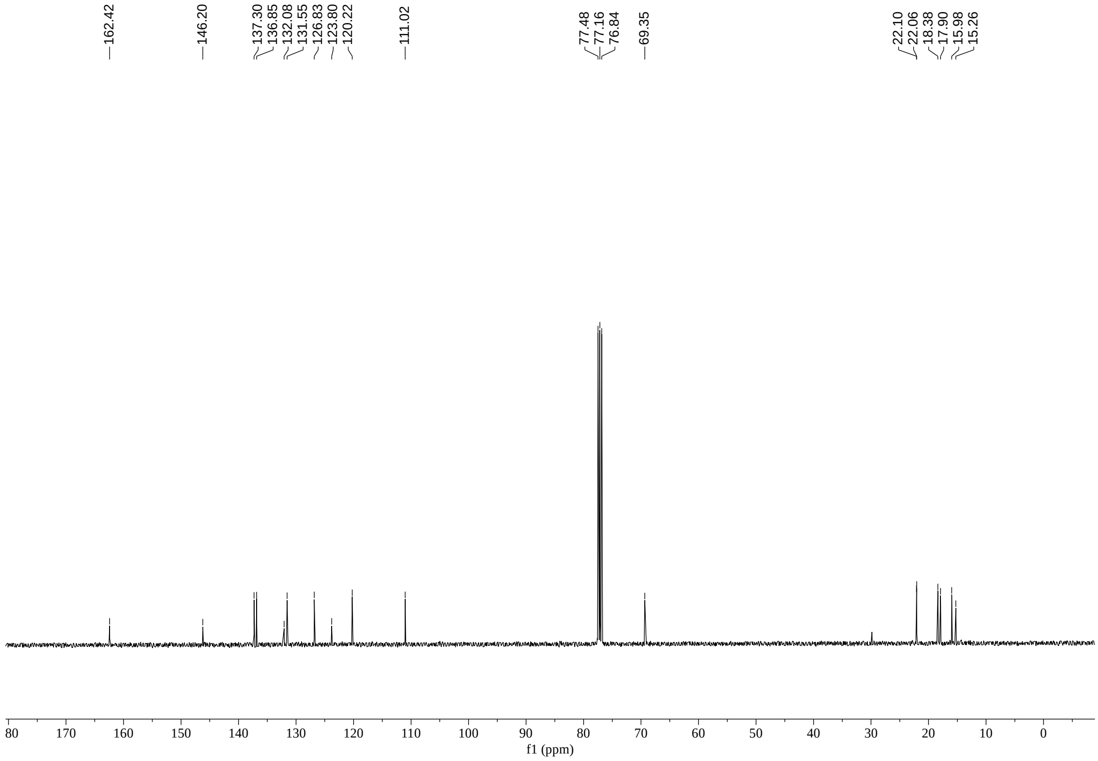

Supplementary Figure 163. ^{13}C NMR spectrum of **3ea**

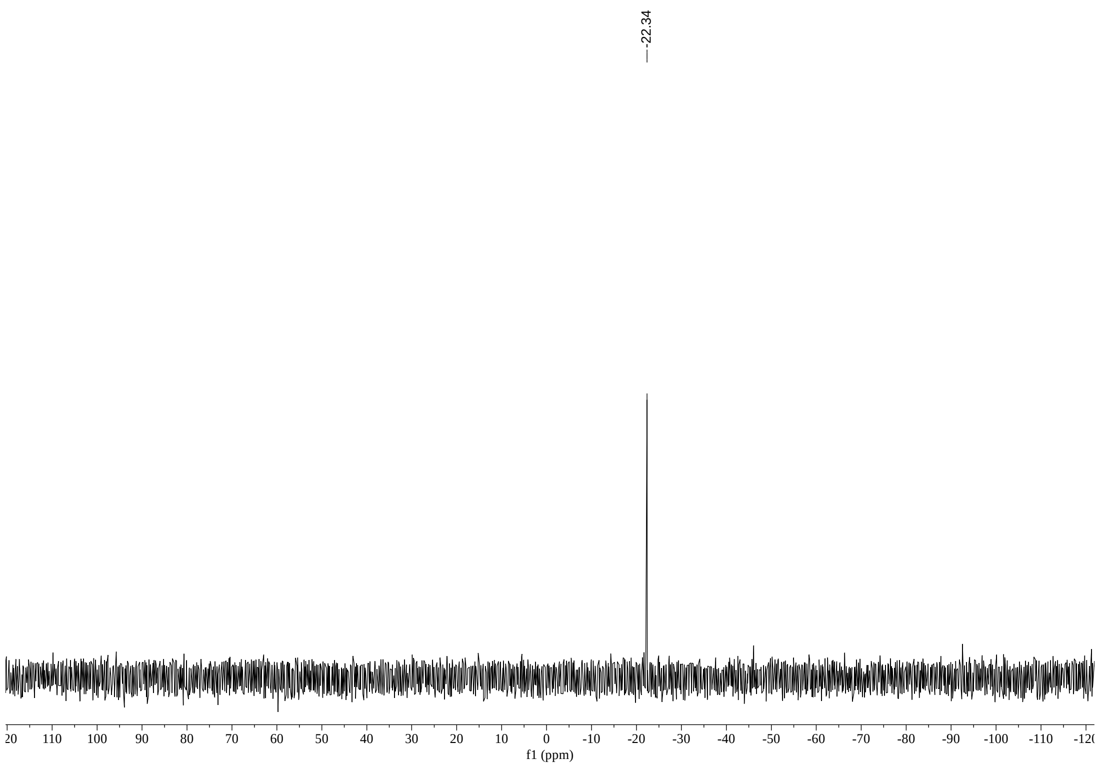

Supplementary Figure 164. ^{29}Si NMR spectrum of **3ea**

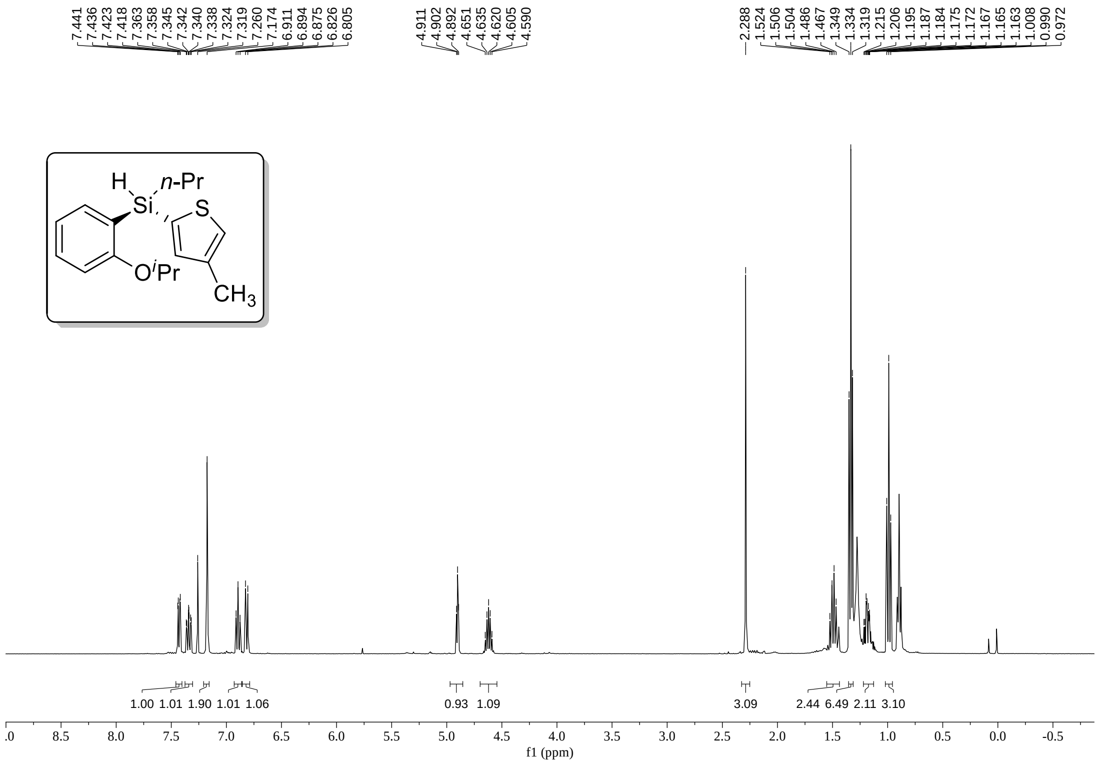

Supplementary Figure 165. ^{1}H NMR spectrum of **3ei**

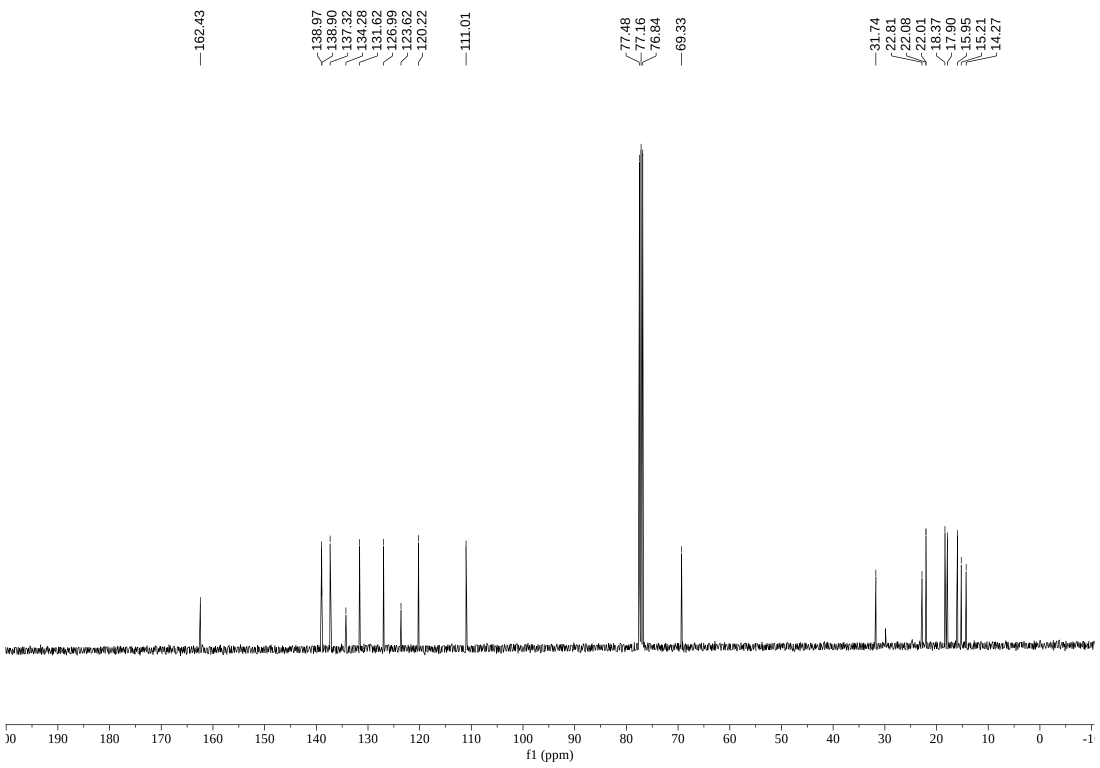

Supplementary Figure 166. ^{13}C NMR spectrum of **3ei**

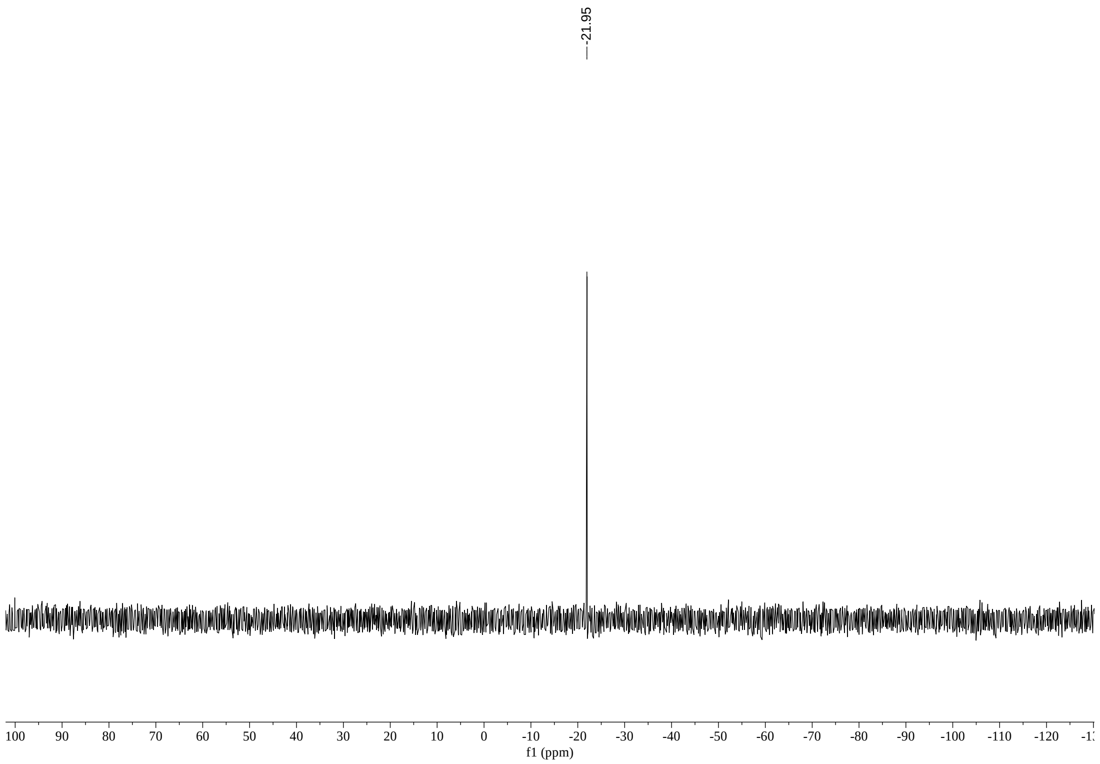

Supplementary Figure 167. ^{29}Si NMR spectrum of **3ei**

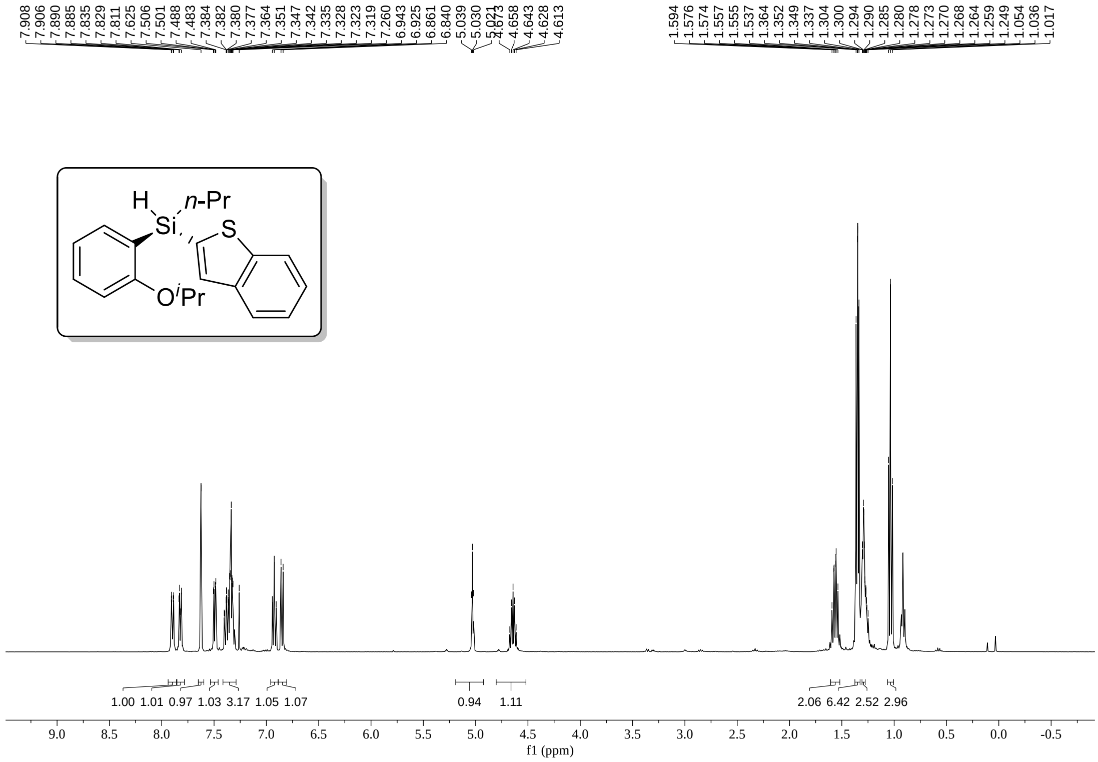

Supplementary Figure 168. ^{1}H NMR spectrum of **3en**

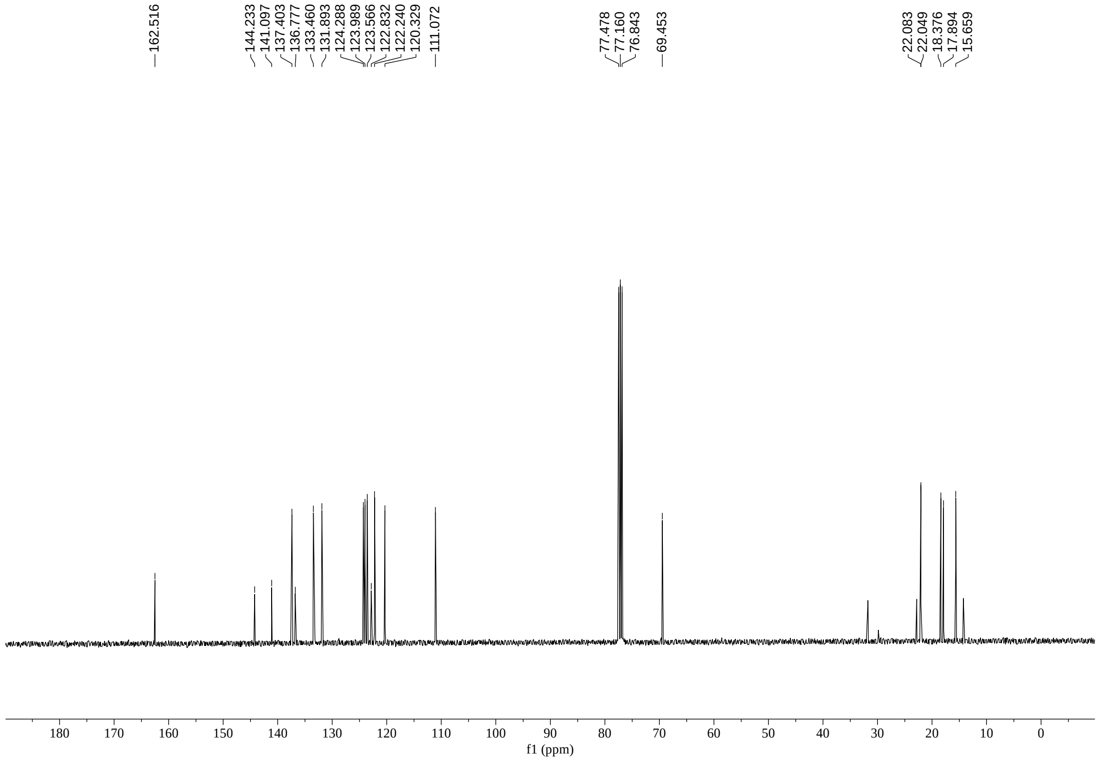

Supplementary Figure 169. ^{13}C NMR spectrum of **3en**

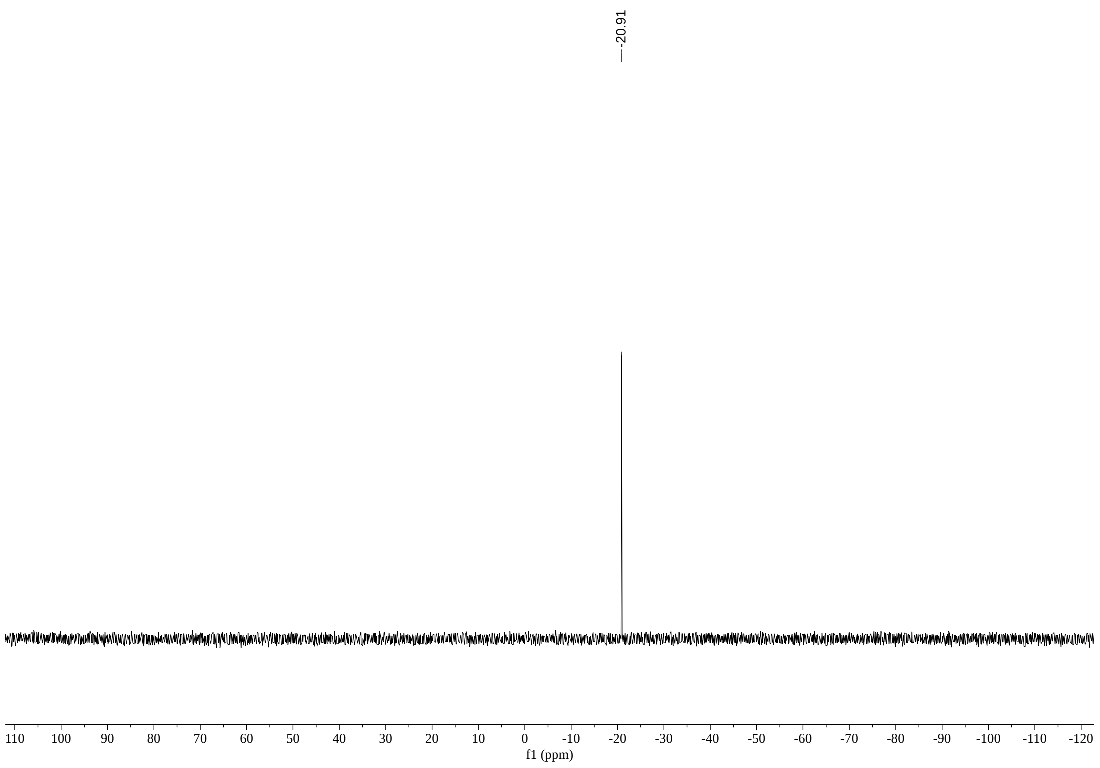

Supplementary Figure 170. ^{29}Si NMR spectrum of **3en**

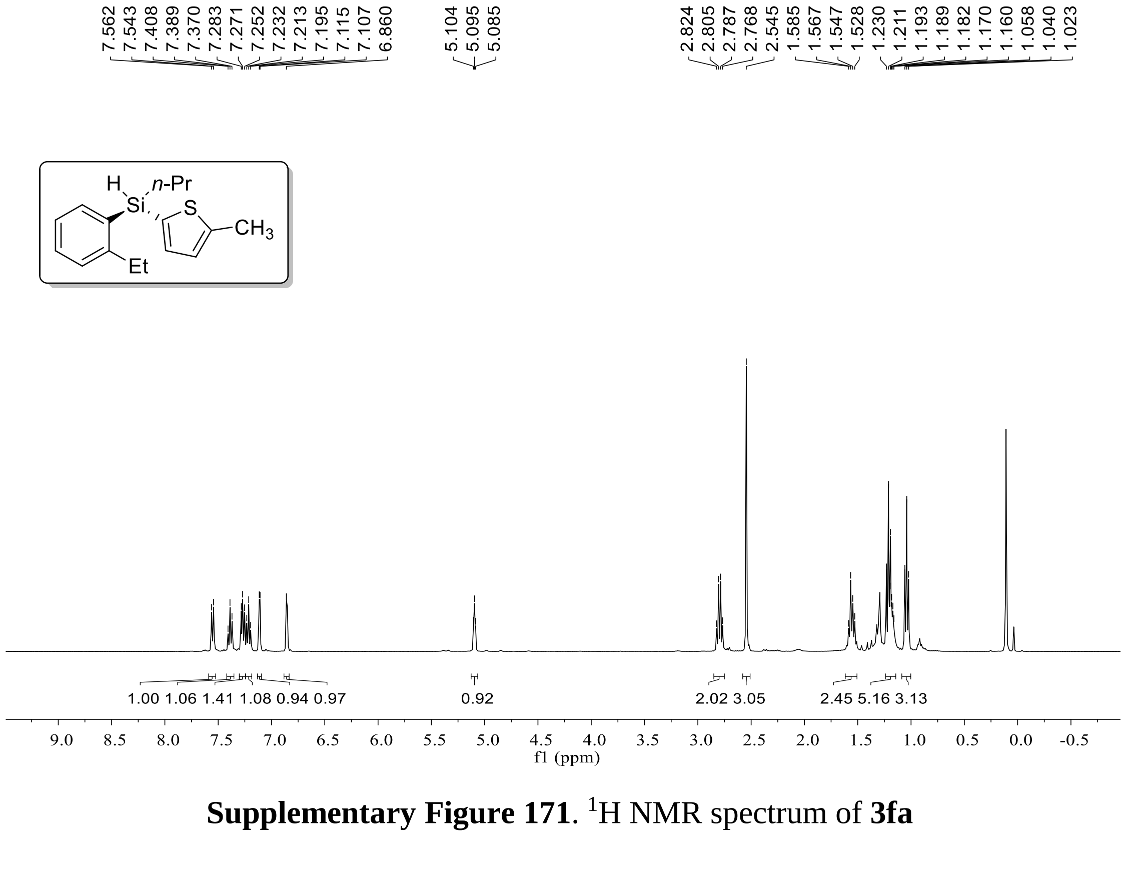

Supplementary Figure 171. ^{1}H NMR spectrum of **3fa**

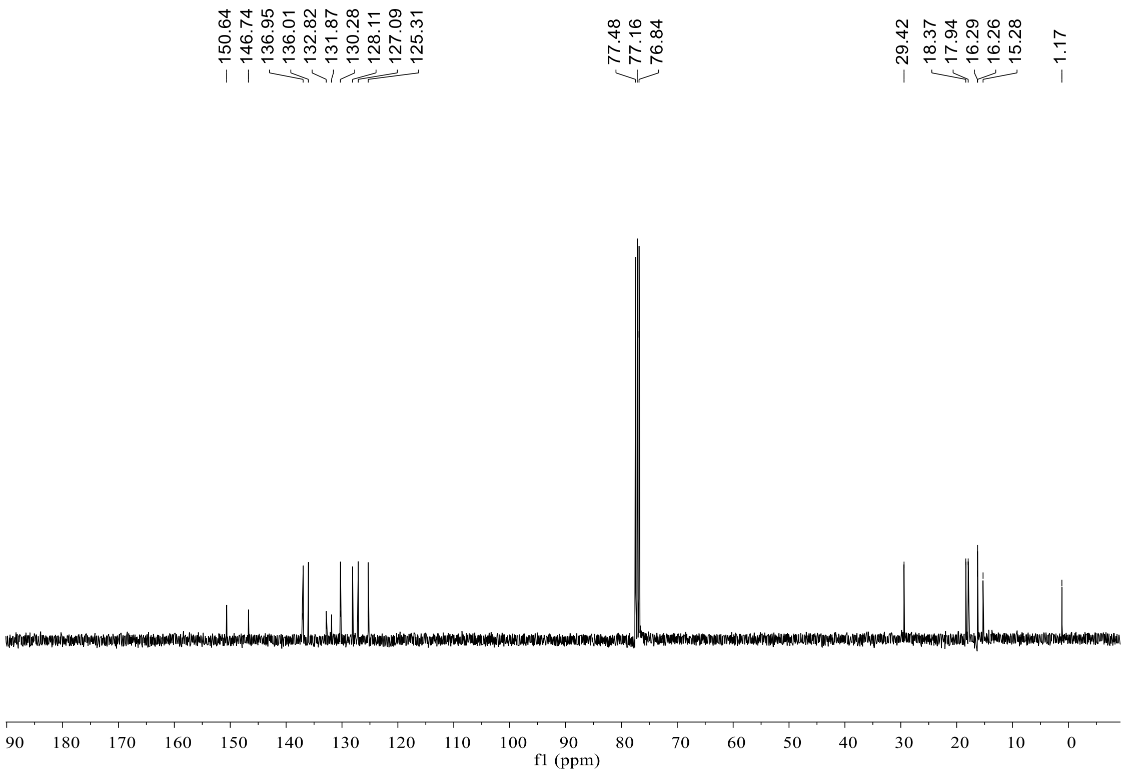

Supplementary Figure 172. ^{13}C NMR spectrum of **3fa**

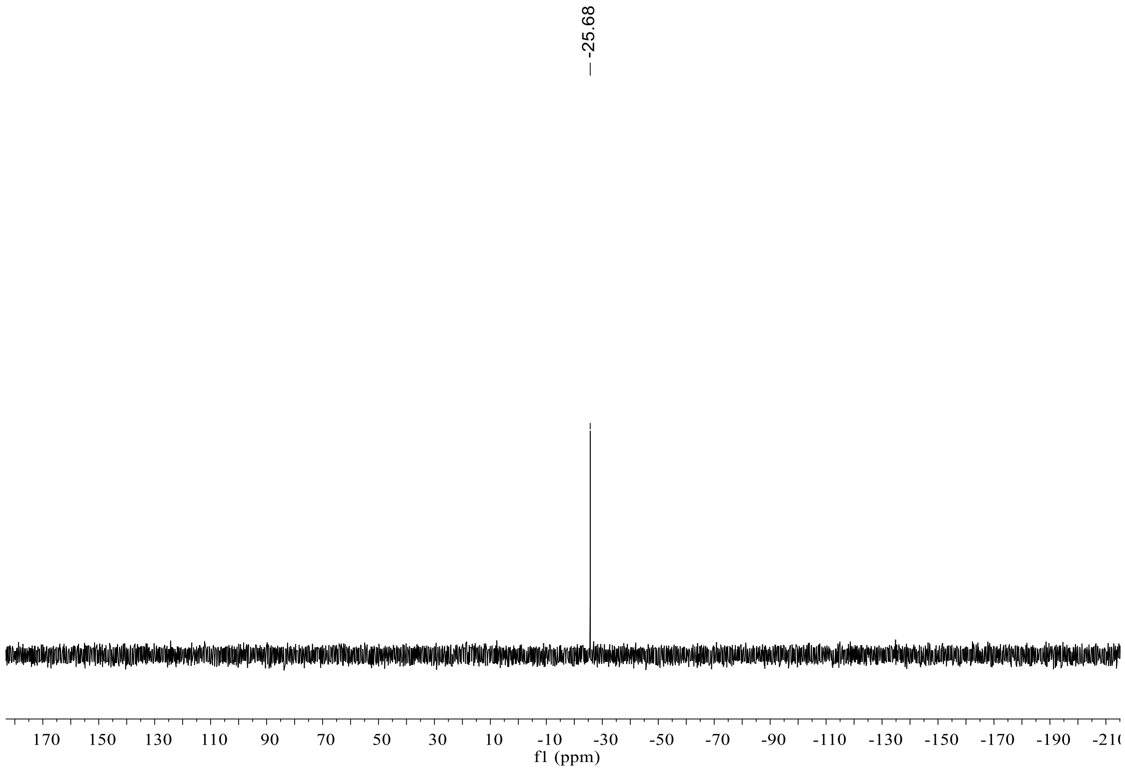

Supplementary Figure 173. ^{29}Si NMR spectrum of **3fa**

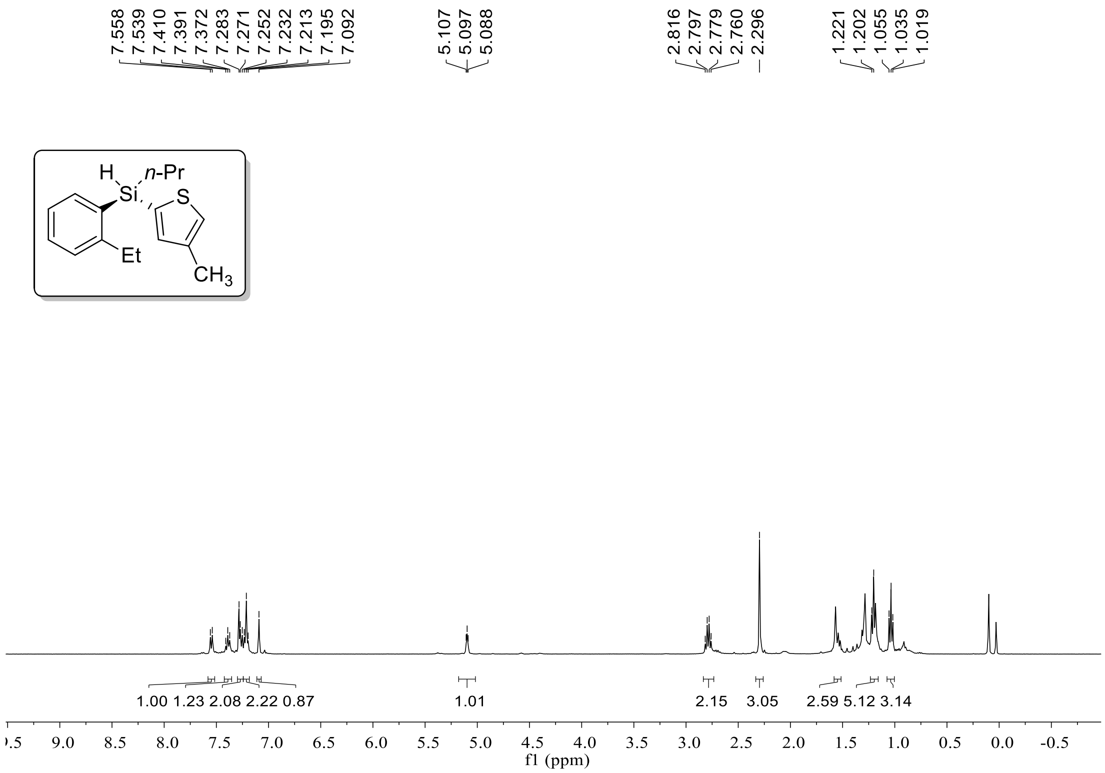

Supplementary Figure 174. ^{1}H NMR spectrum of **3fi**

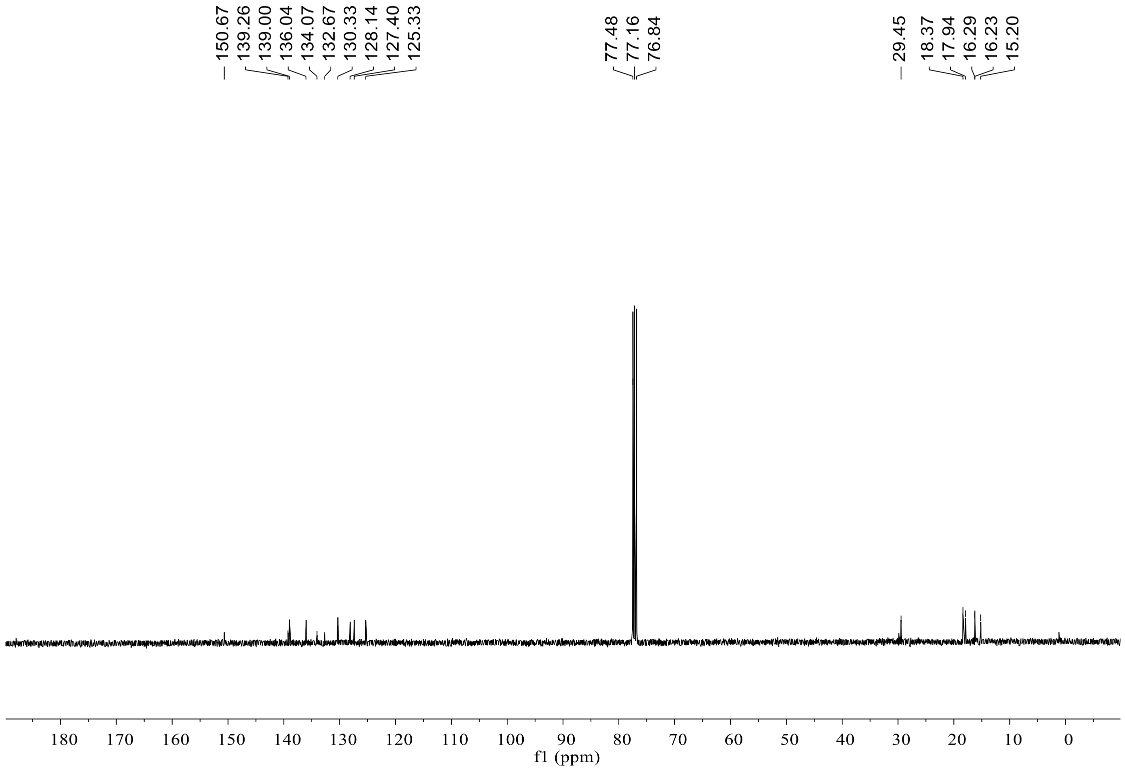

Supplementary Figure 175. ^{13}C NMR spectrum of **3fi**

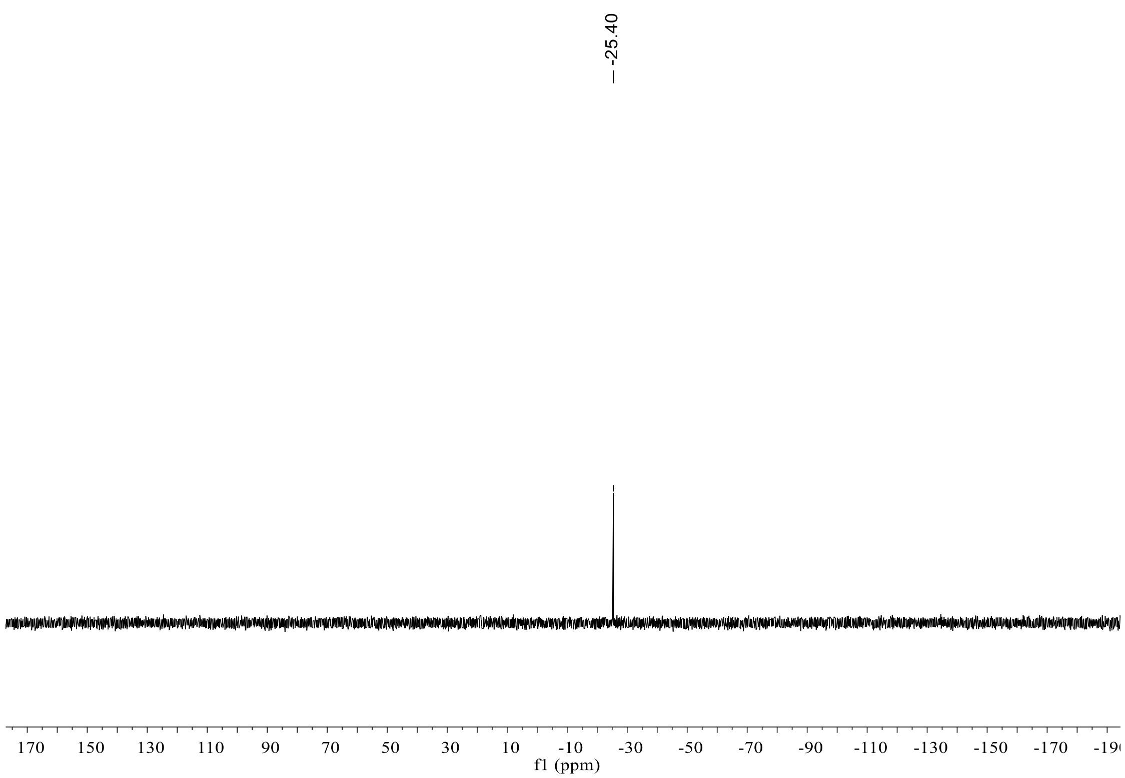

Supplementary Figure 176. ^{29}Si NMR spectrum of **3fi**

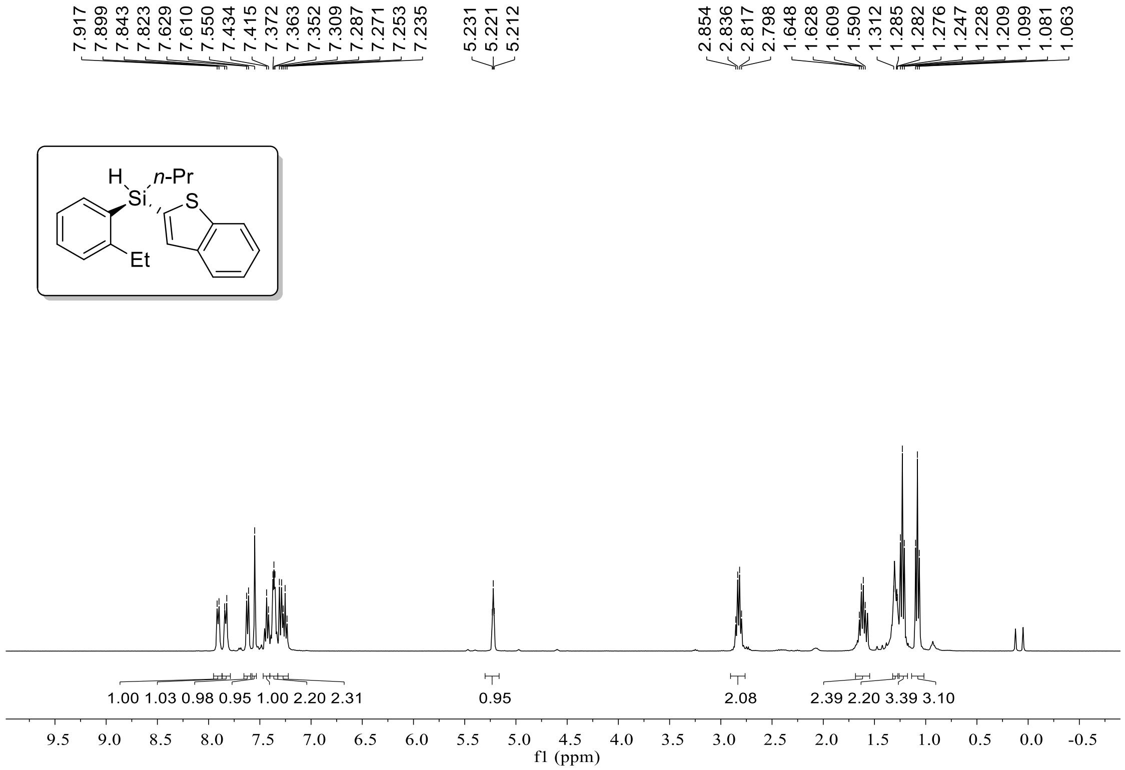

Supplementary Figure 177. ^{1}H NMR spectrum of **3fn**

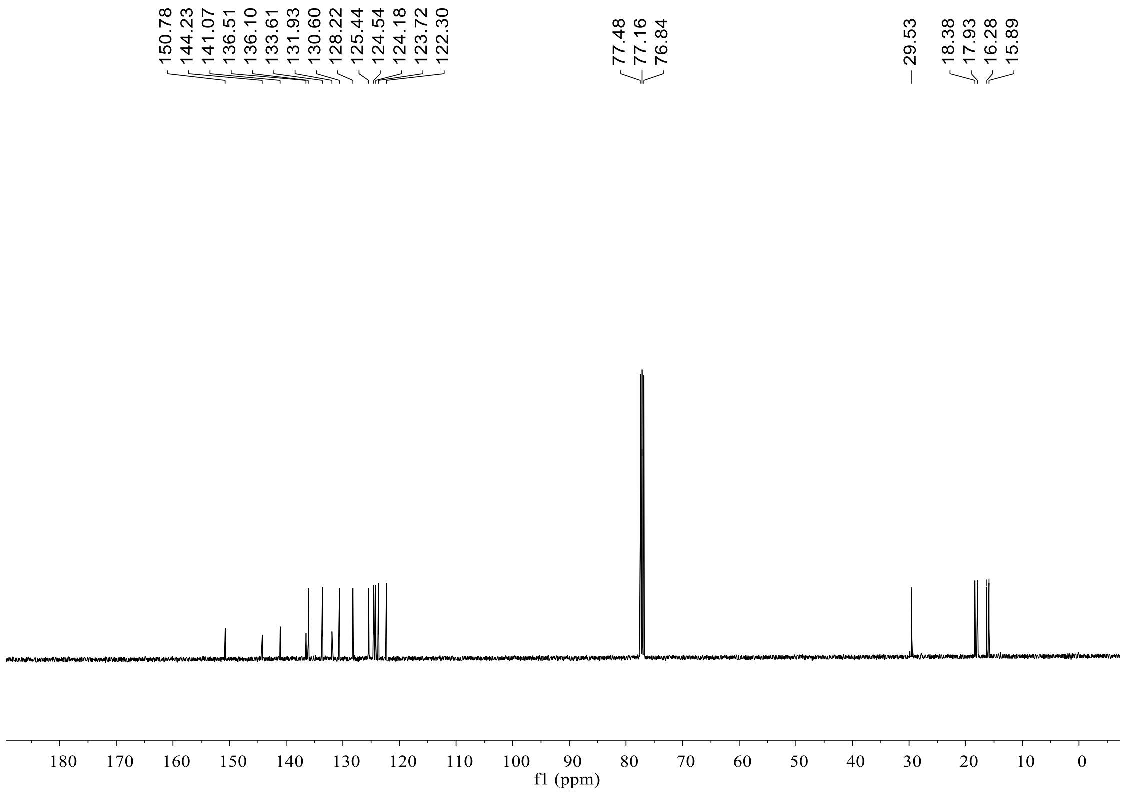

Supplementary Figure 178. ^{13}C NMR spectrum of **3fn**

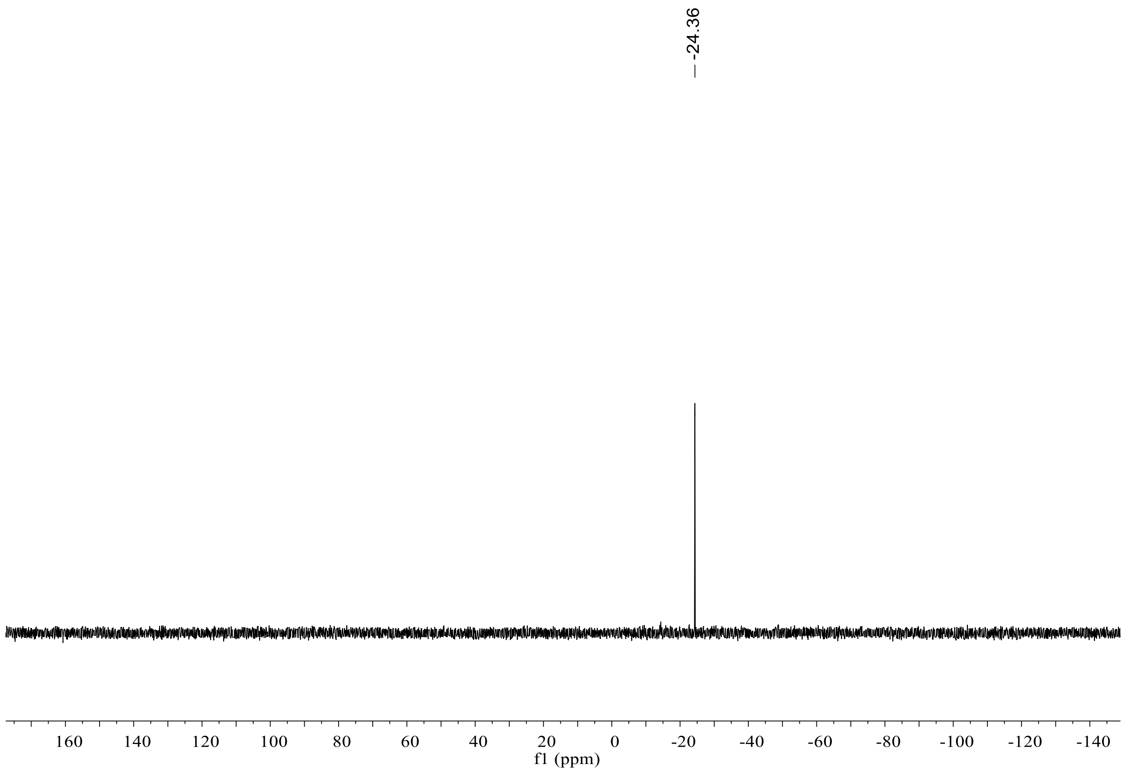

Supplementary Figure 179. ^{29}Si NMR spectrum of **3fn**

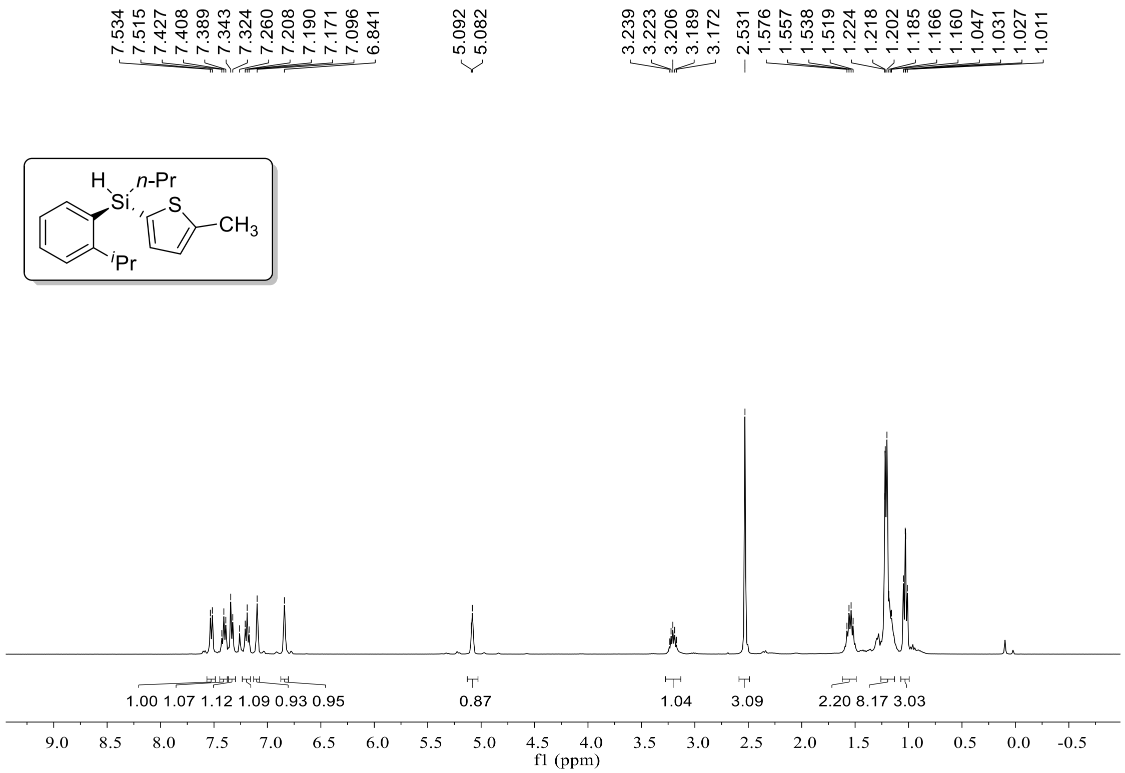

Supplementary Figure 180. ^{1}H NMR spectrum of **3ga**

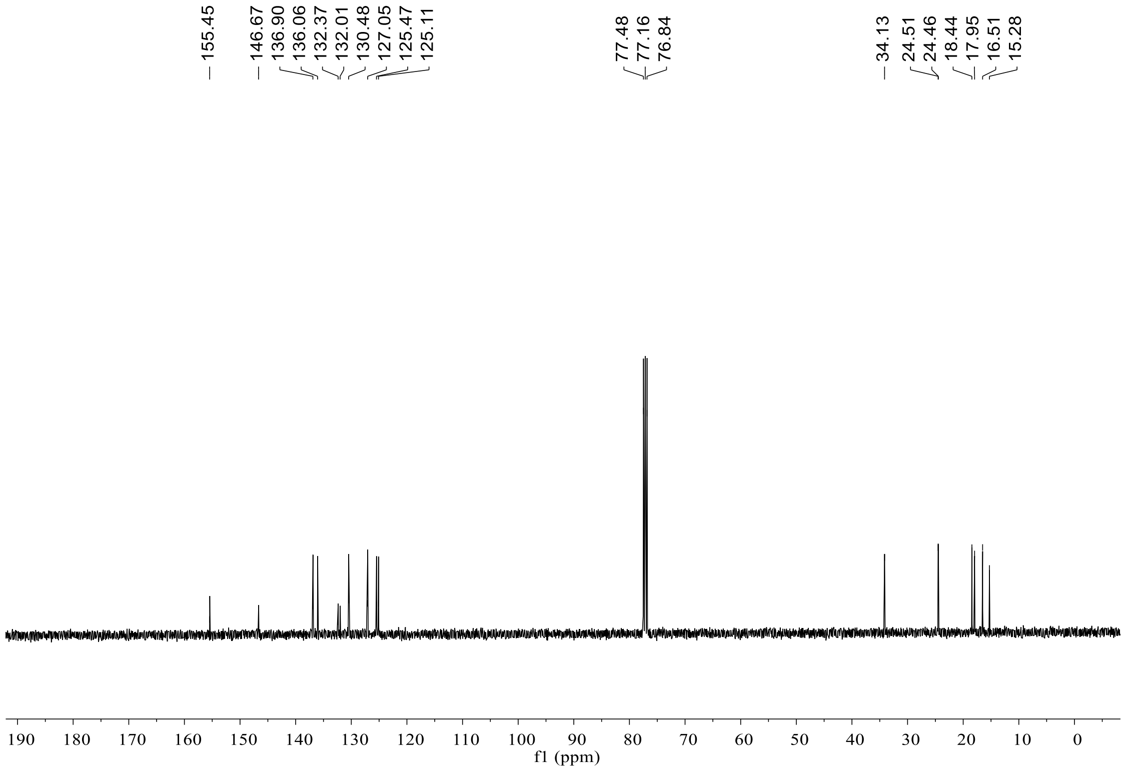

Supplementary Figure 181. ^{13}C NMR spectrum of **3ga**

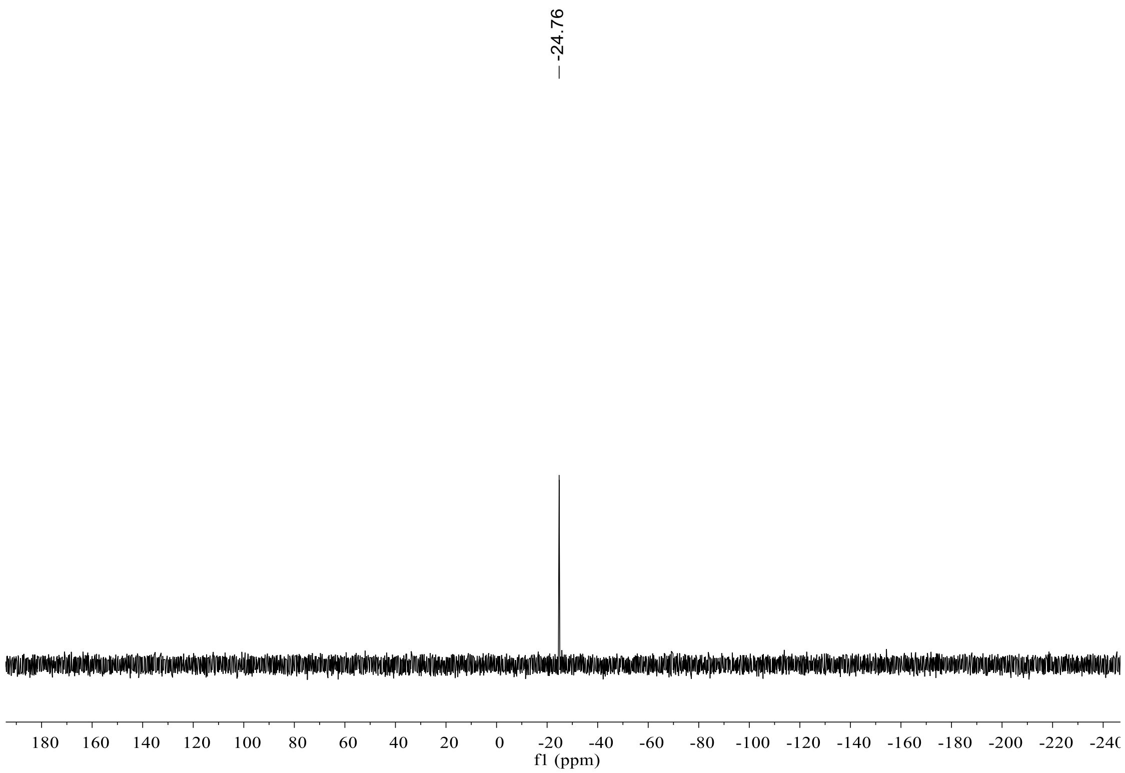

Supplementary Figure 182. ^{29}Si NMR spectrum of **3ga**

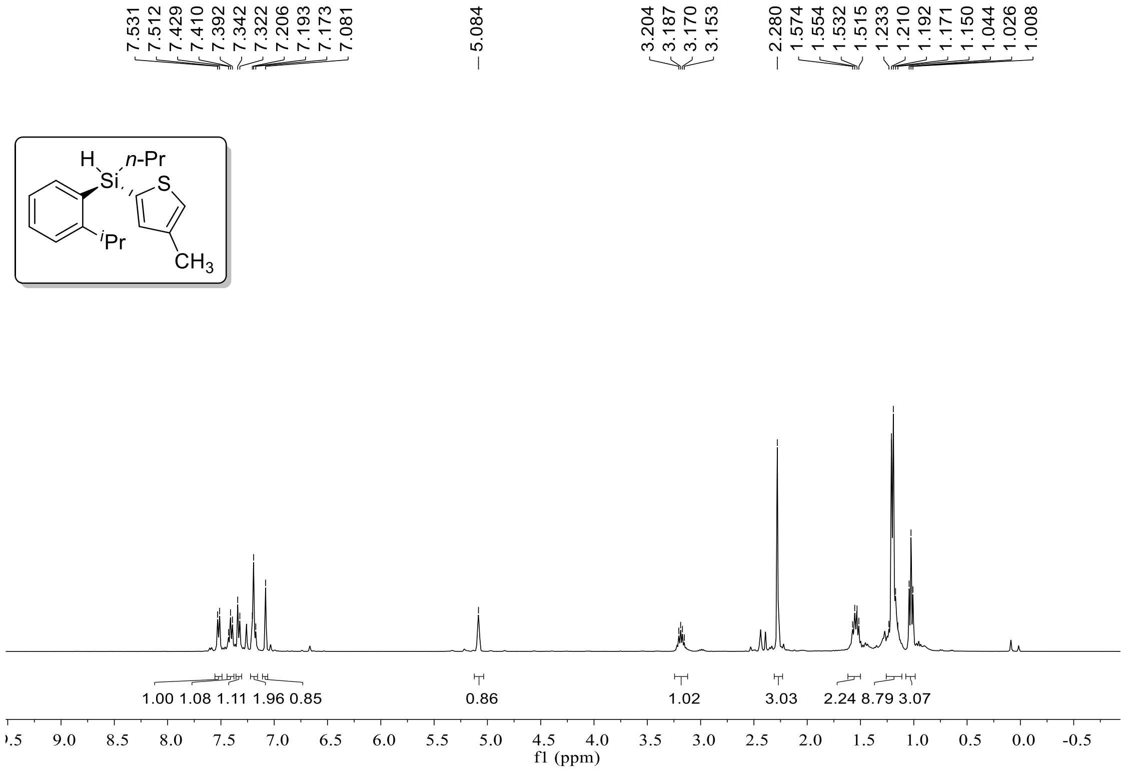

Supplementary Figure 183. ^{1}H NMR spectrum of **3gi**

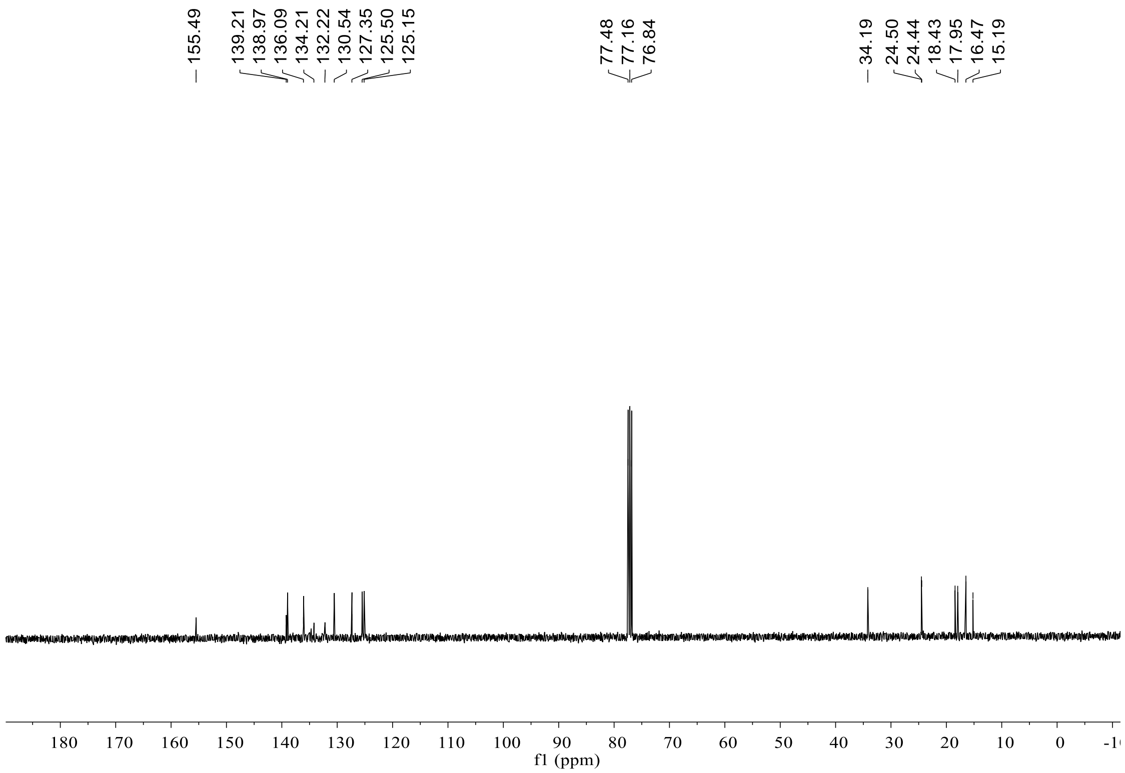

Supplementary Figure 184. ^{13}C NMR spectrum of **3gi**

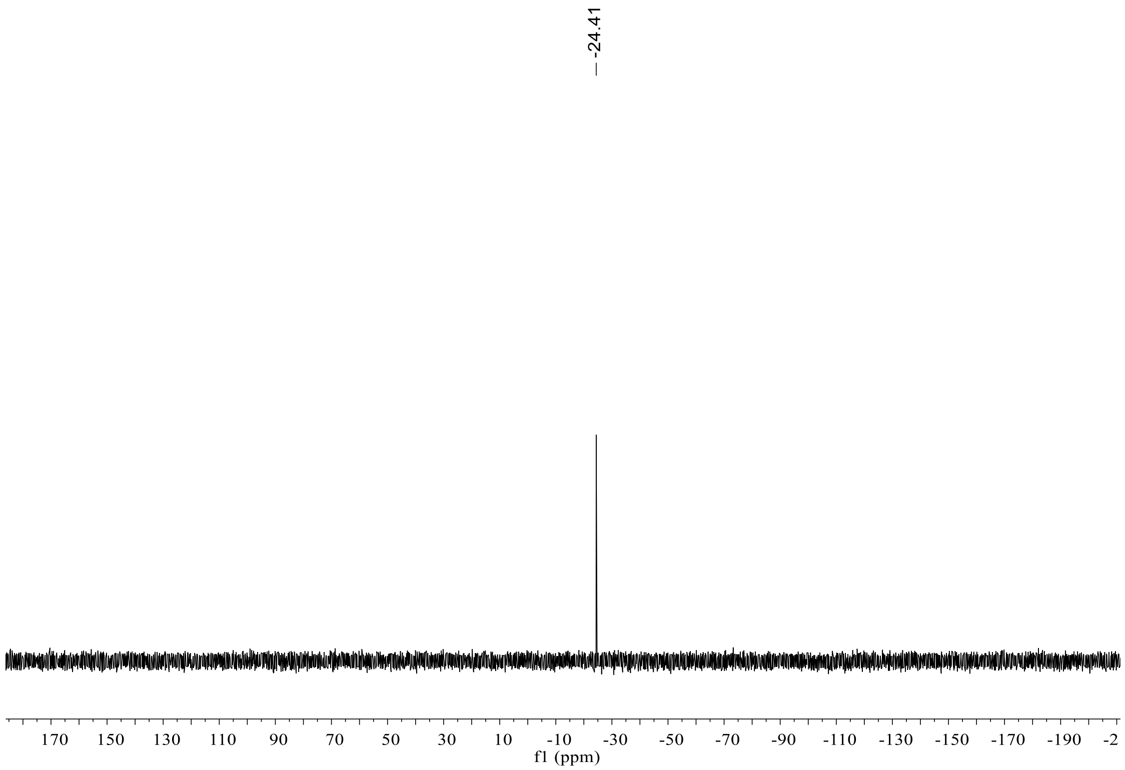

Supplementary Figure 185. ^{29}Si NMR spectrum of **3gi**

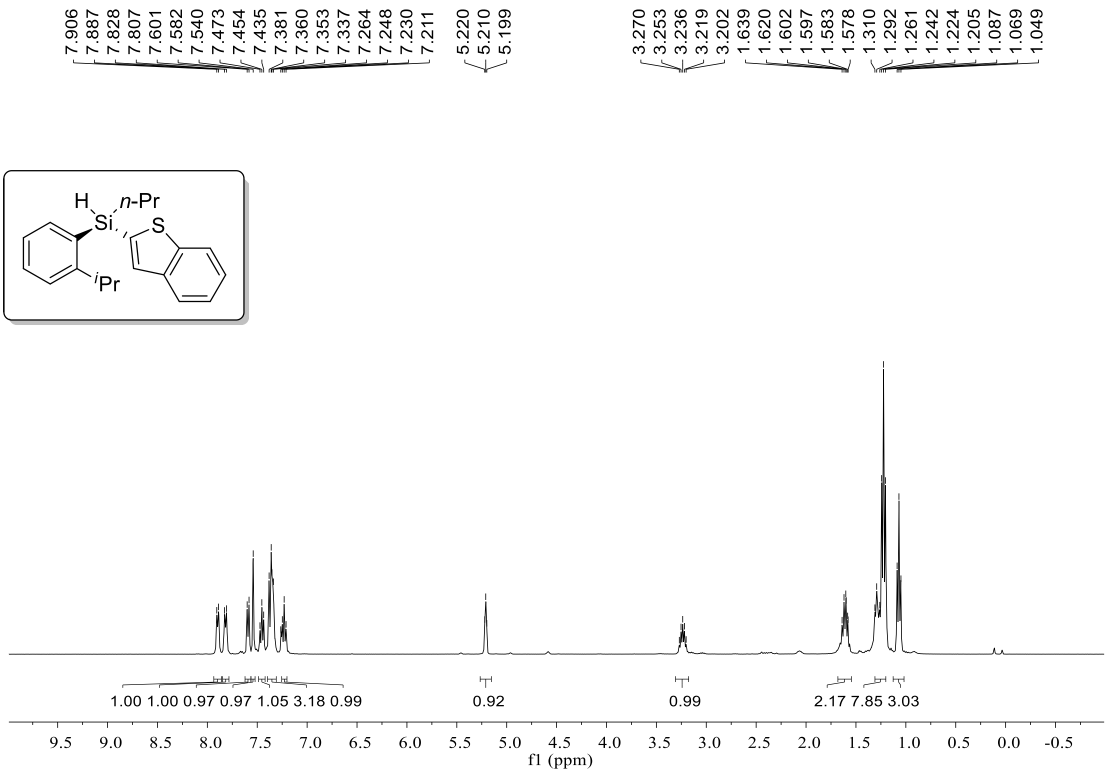

Supplementary Figure 186. ^{1}H NMR spectrum of **3gn**

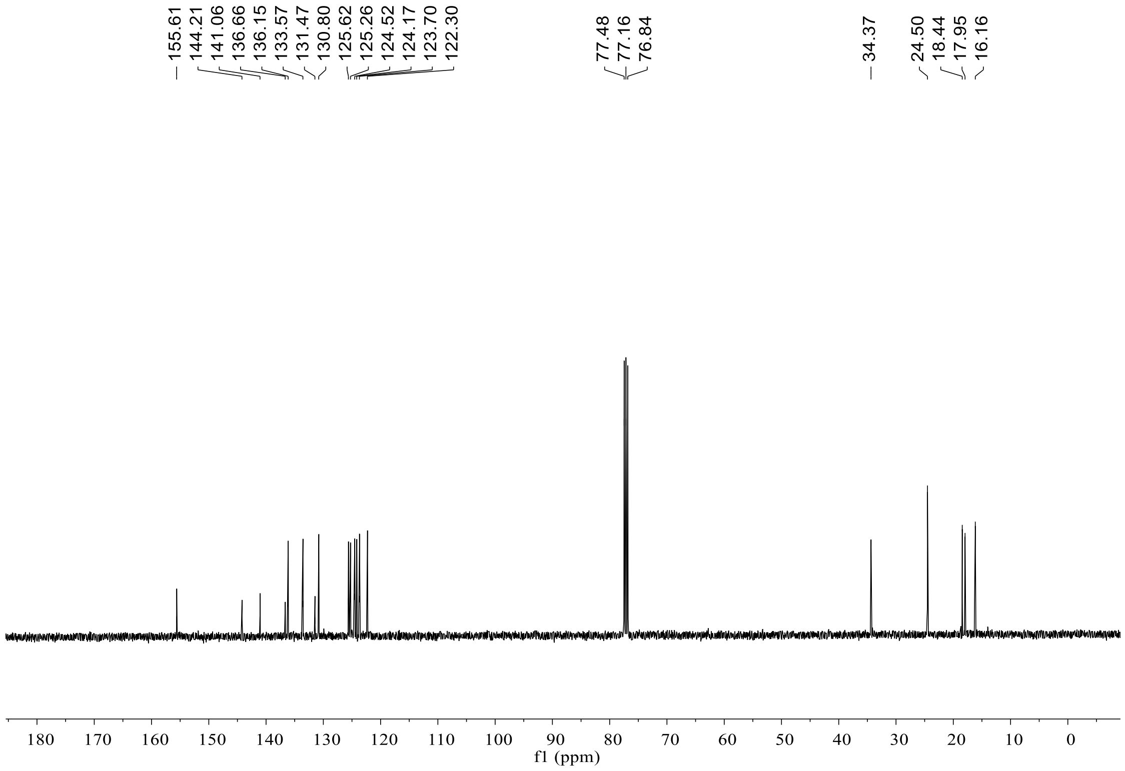

Supplementary Figure 187. ^{13}C NMR spectrum of **3gn**

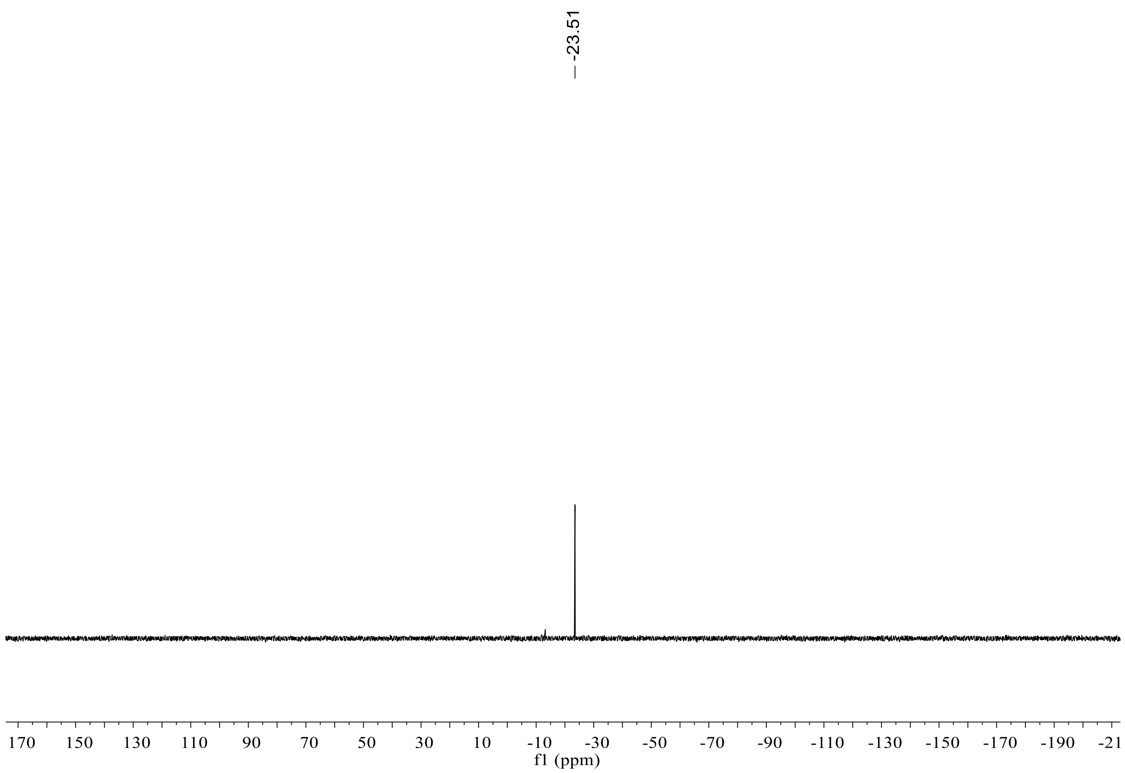

Supplementary Figure 188. ^{29}Si NMR spectrum of **3gn**

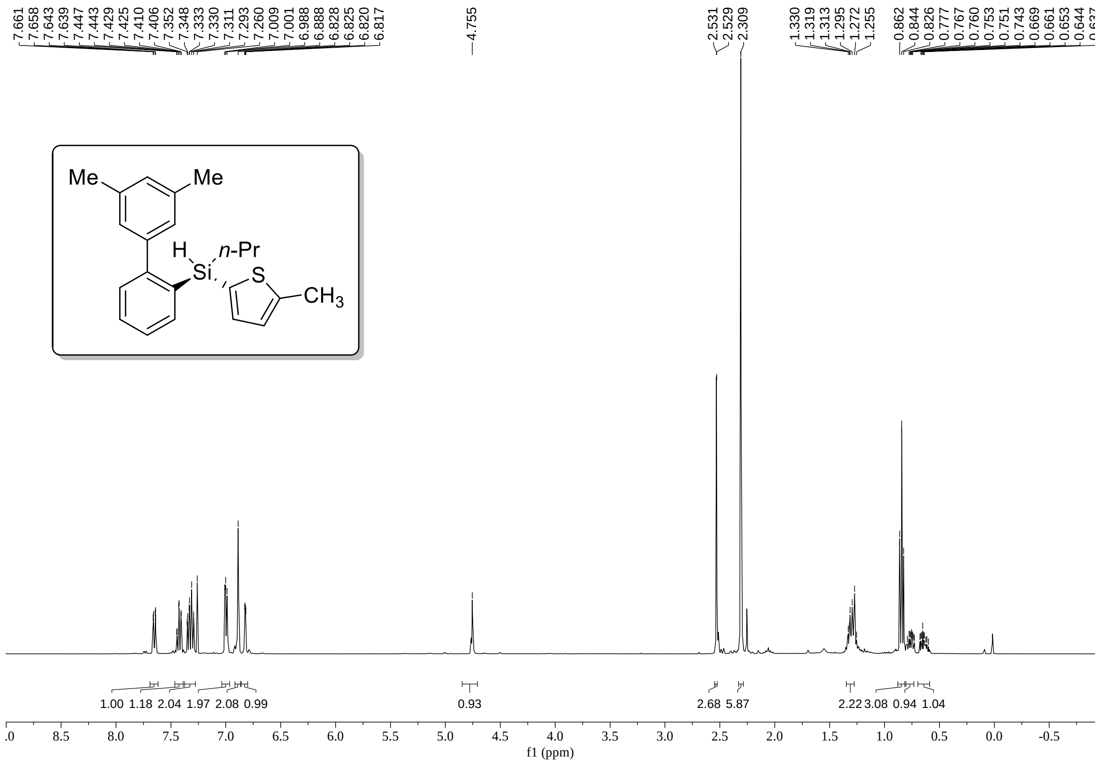

Supplementary Figure 189. ^{1}H NMR spectrum of **3ha**

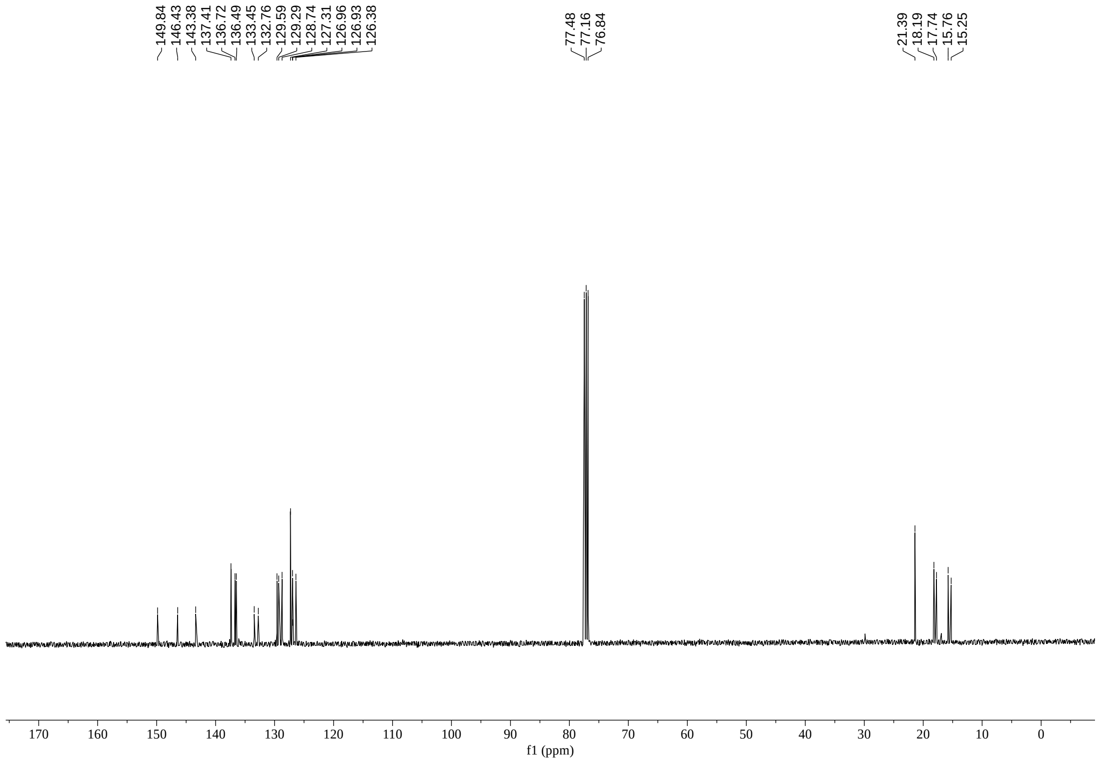

Supplementary Figure 190. ^{13}C NMR spectrum of **3ha**

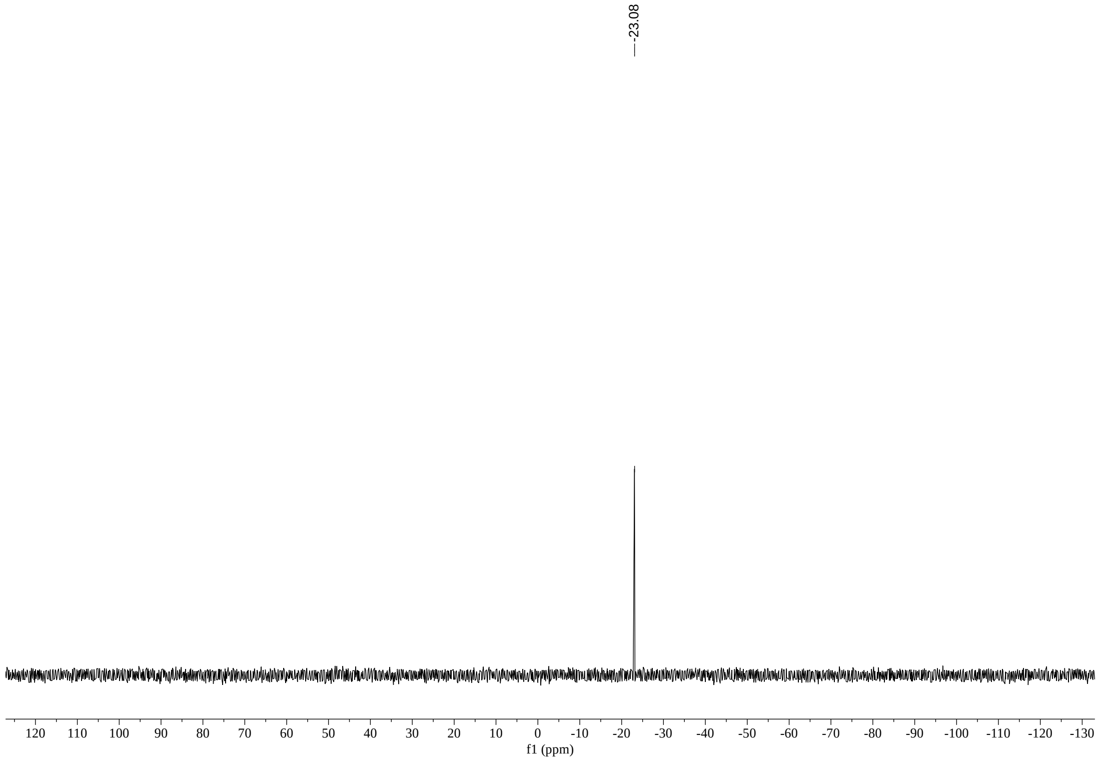

Supplementary Figure 191. ^{29}Si NMR spectrum of **3ha**

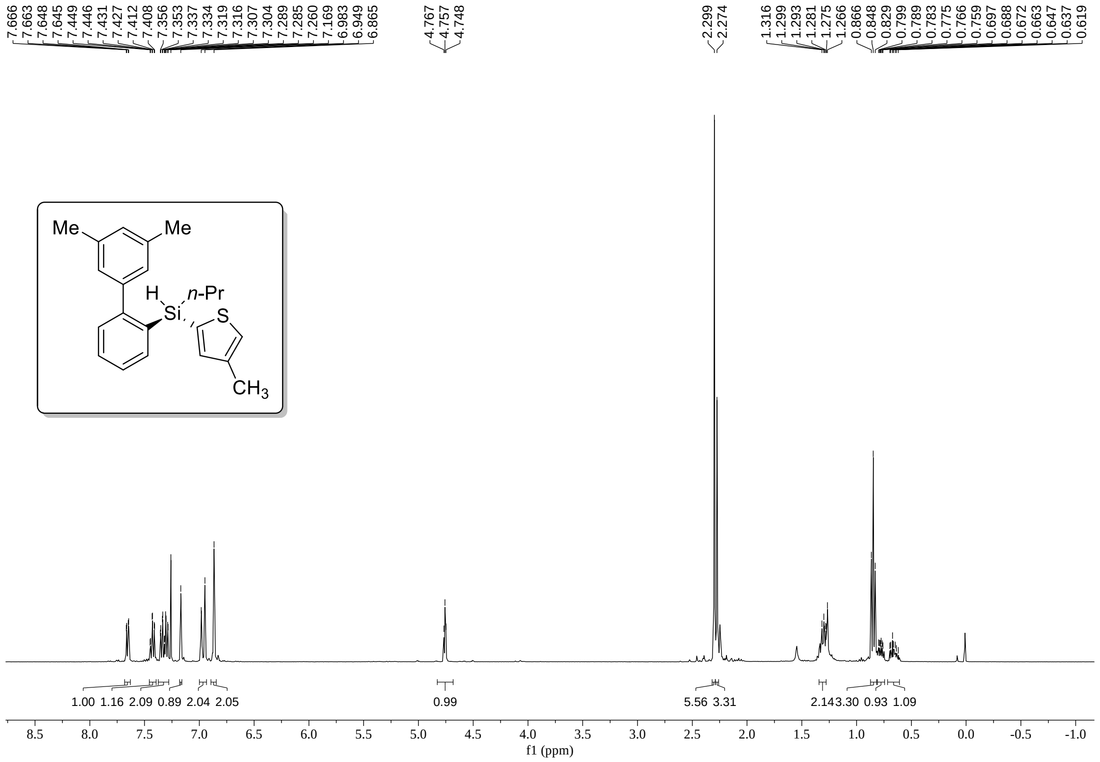

Supplementary Figure 192. ^{1}H NMR spectrum of **3hi**

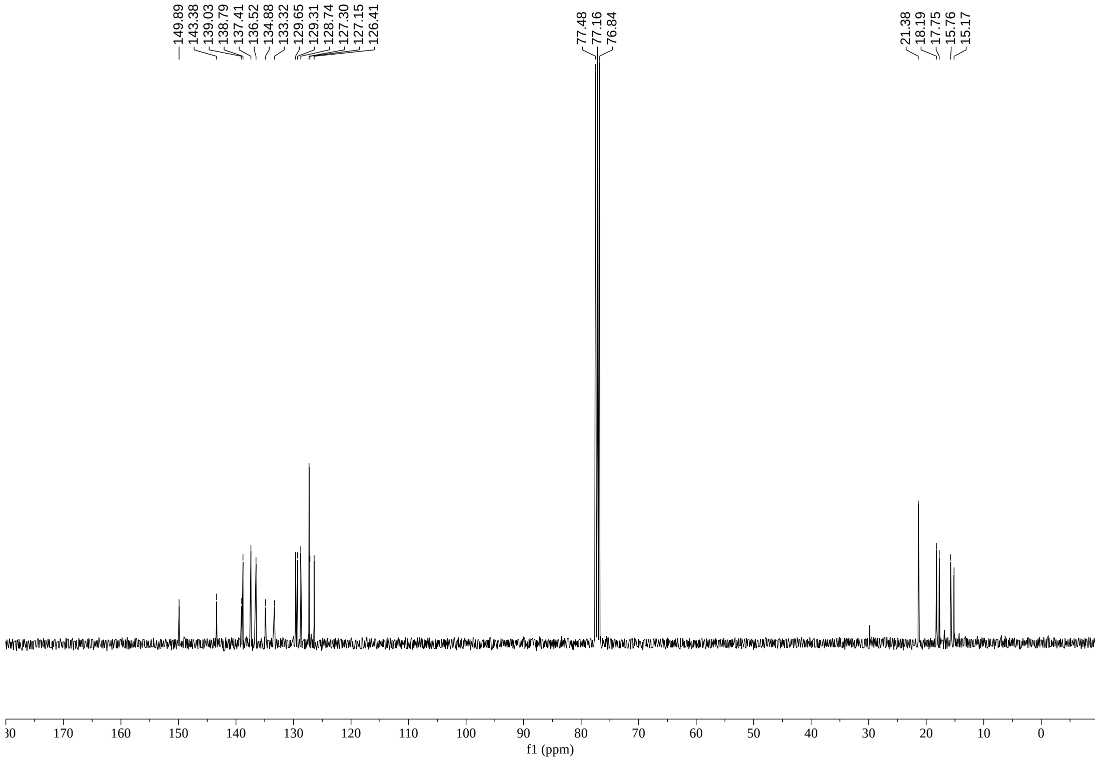

Supplementary Figure 193. ^{13}C NMR spectrum of **3hi**

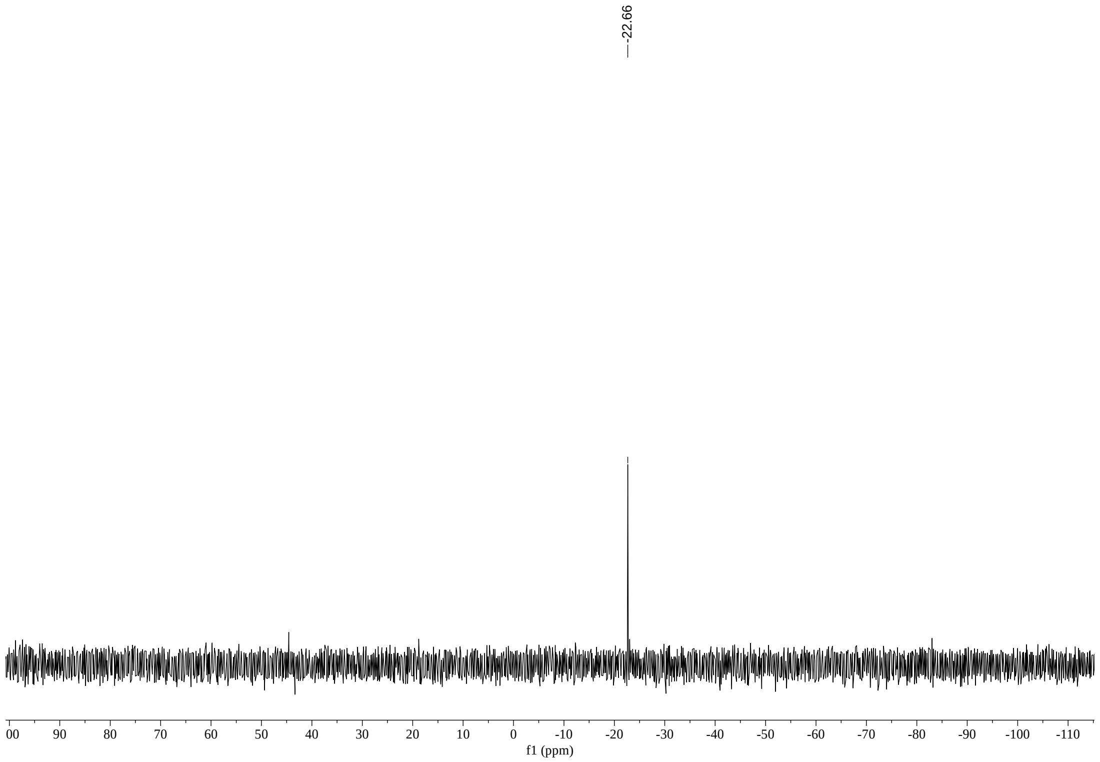

Supplementary Figure 194. ^{29}Si NMR spectrum of **3hi**

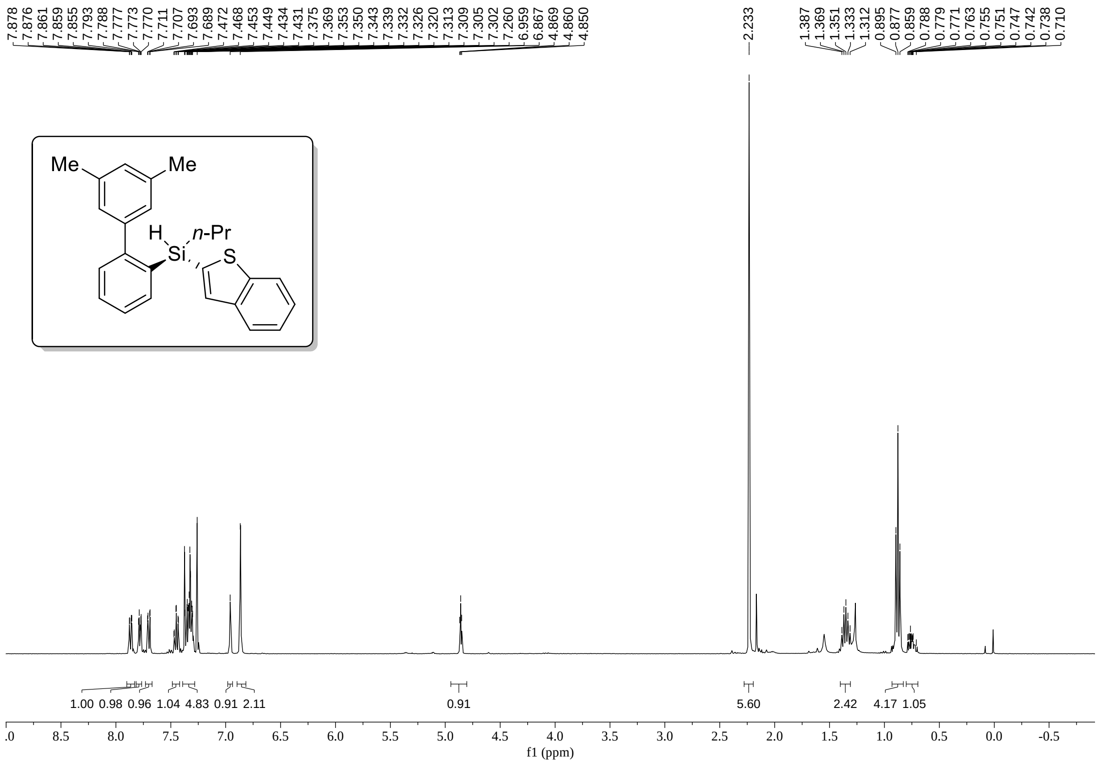

Supplementary Figure 195. ^{1}H NMR spectrum of **3hn**

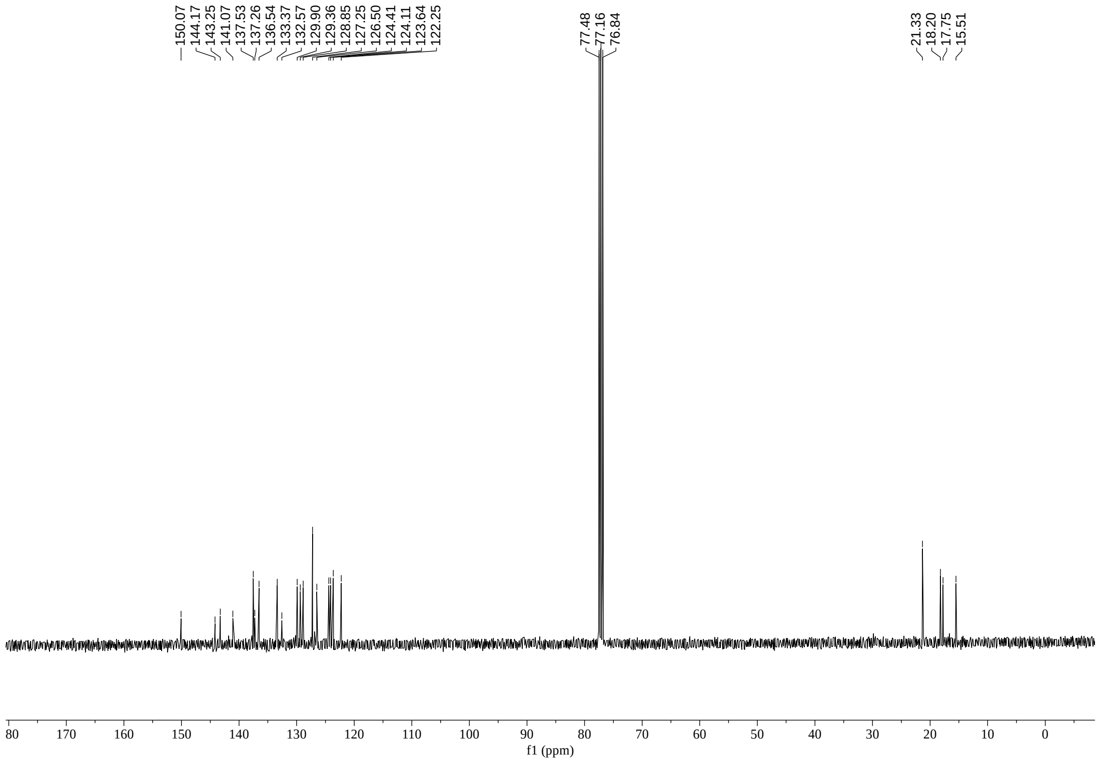

Supplementary Figure 196. ^{13}C NMR spectrum of **3hn**

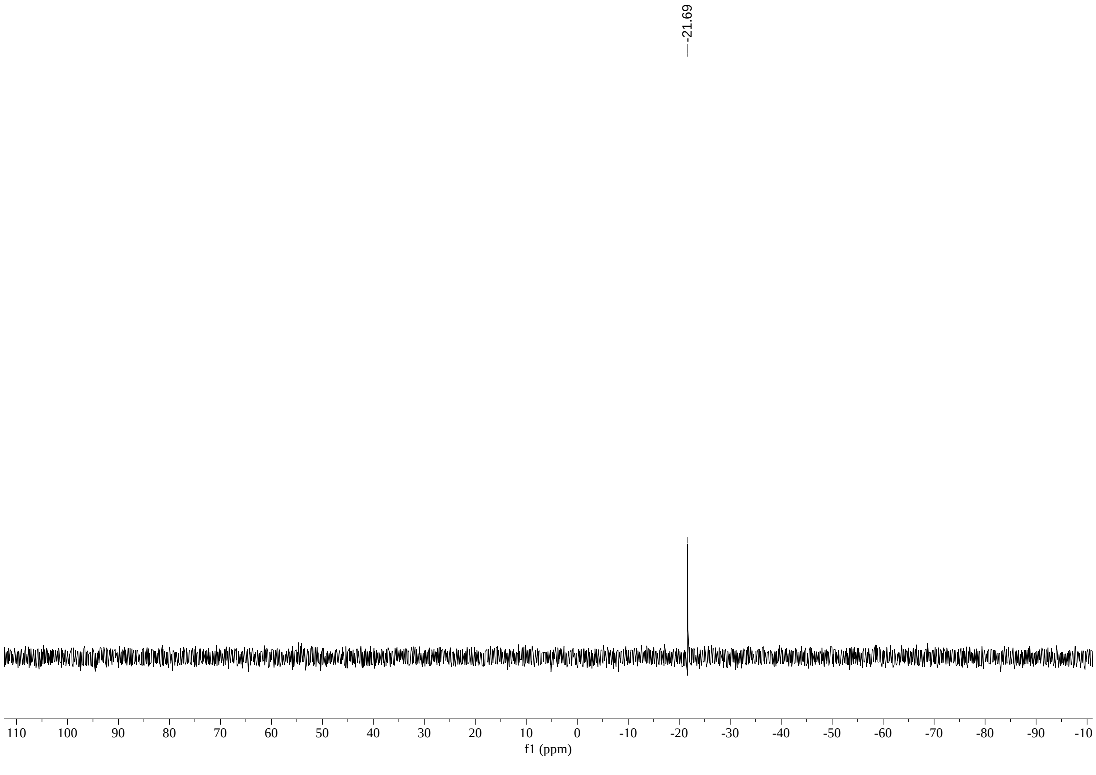

Supplementary Figure 197. ^{29}Si NMR spectrum of **3hn**

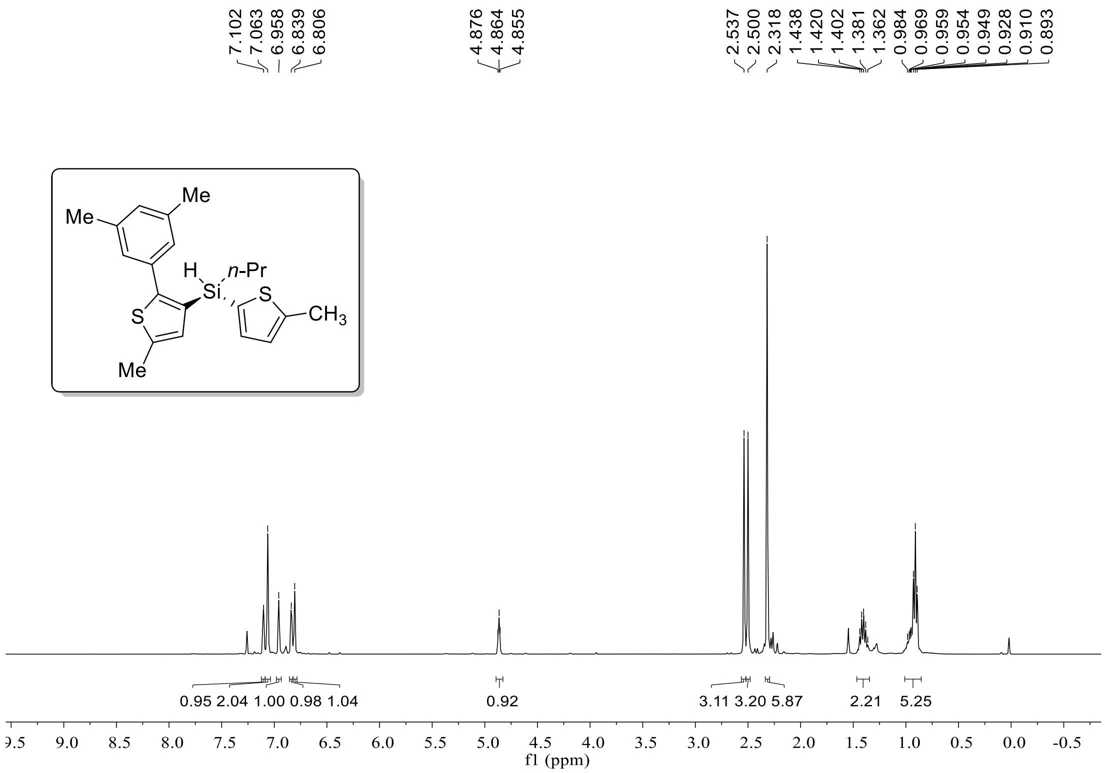

Supplementary Figure 198. ^{1}H NMR spectrum of **3ia**

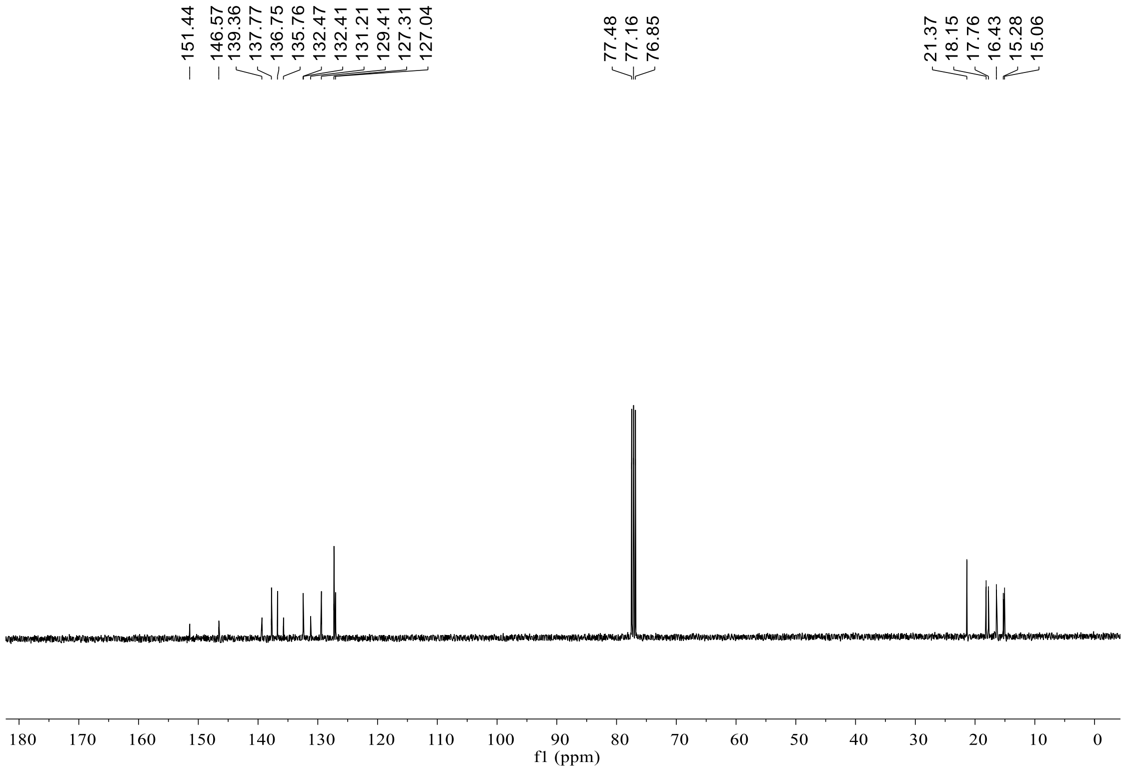

Supplementary Figure 199. ^{13}C NMR spectrum of **3ia**

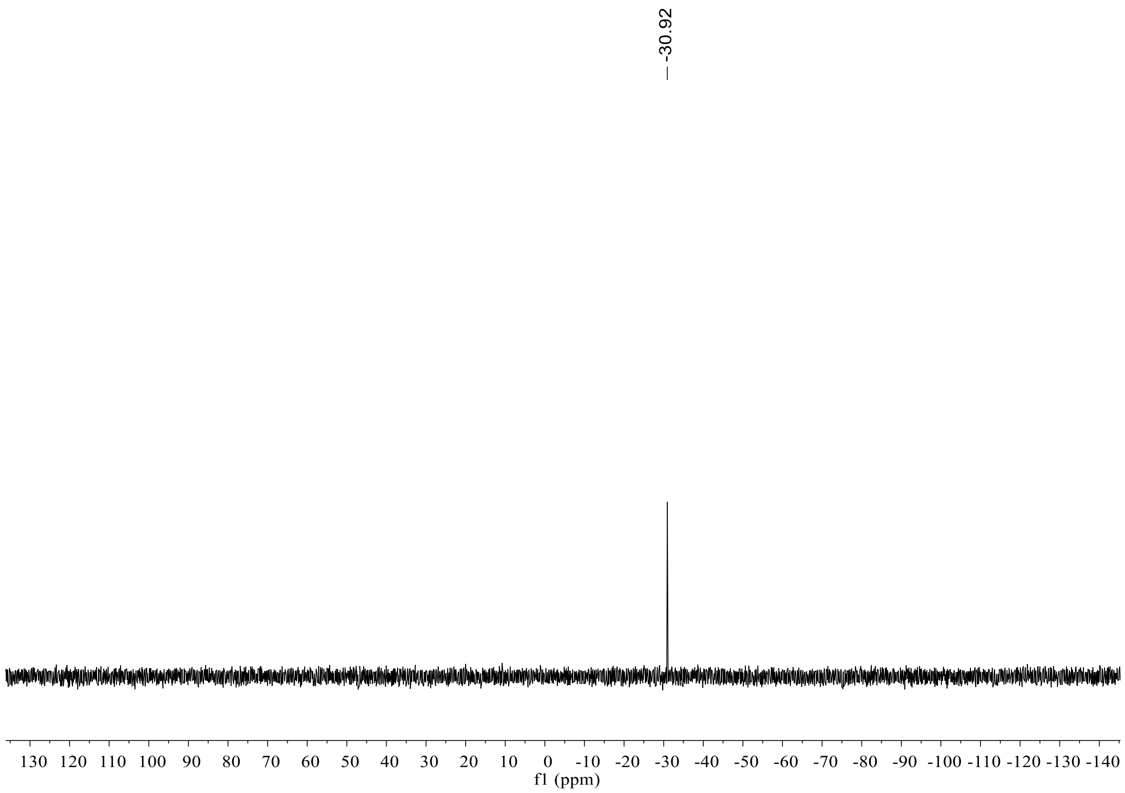

Supplementary Figure 200. ^{29}Si NMR spectrum of **3ia**

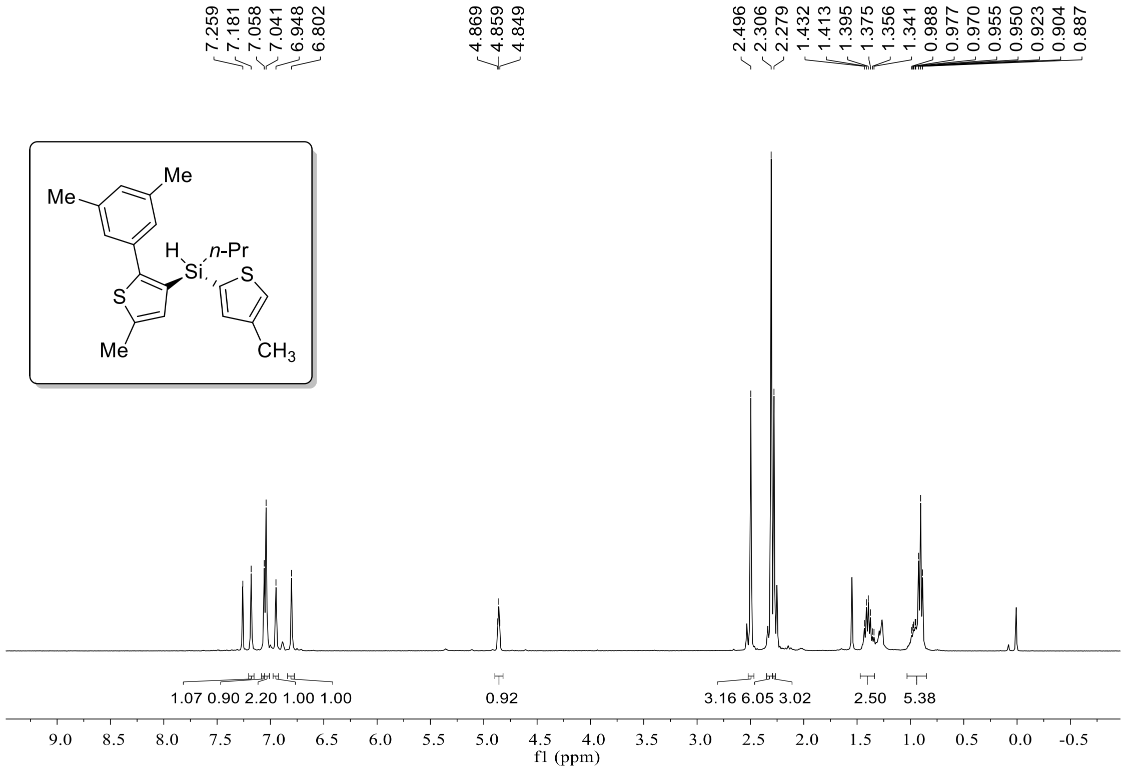

Supplementary Figure 201. ^{1}H NMR spectrum of **3ii**

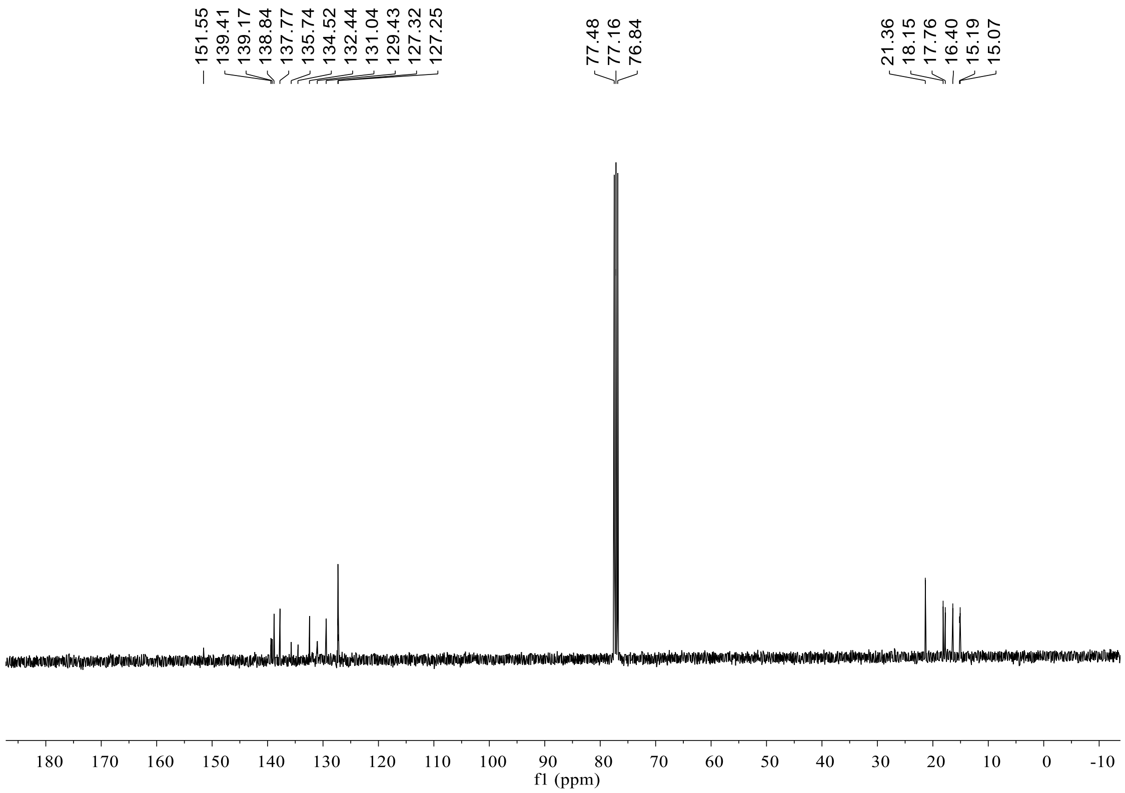

Supplementary Figure 202. ^{13}C NMR spectrum of **3ii**

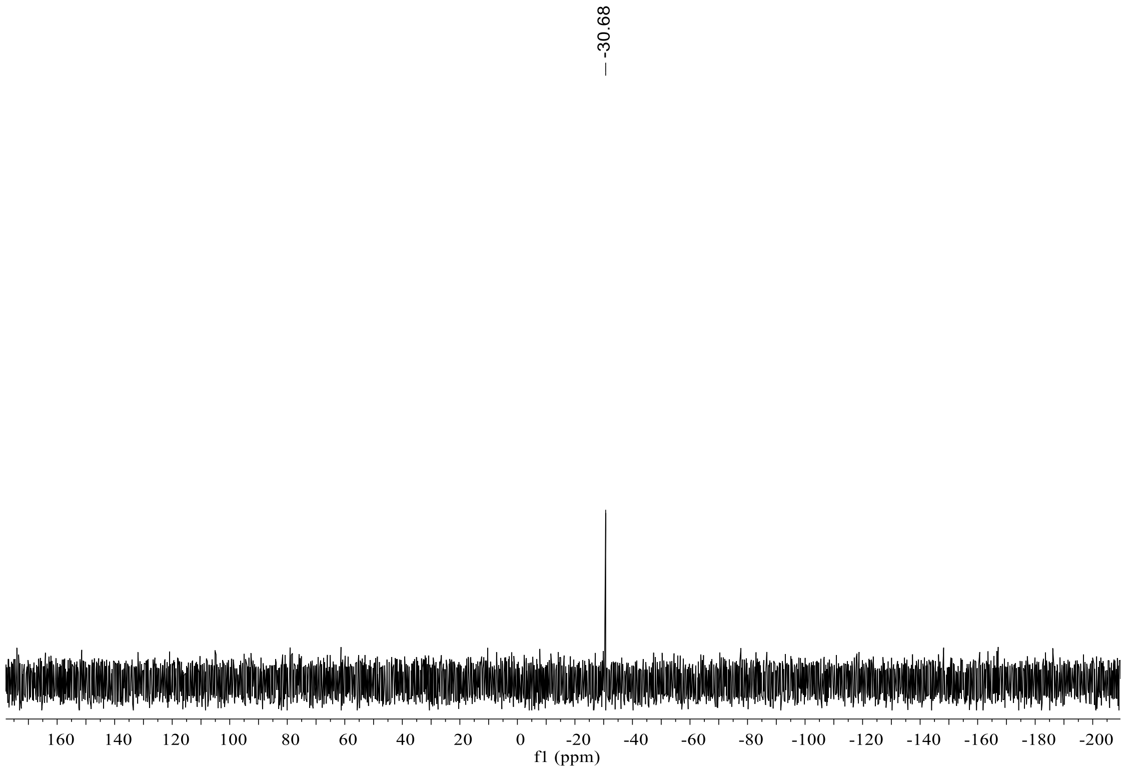

Supplementary Figure 203. ^{29}Si NMR spectrum of **3ii**

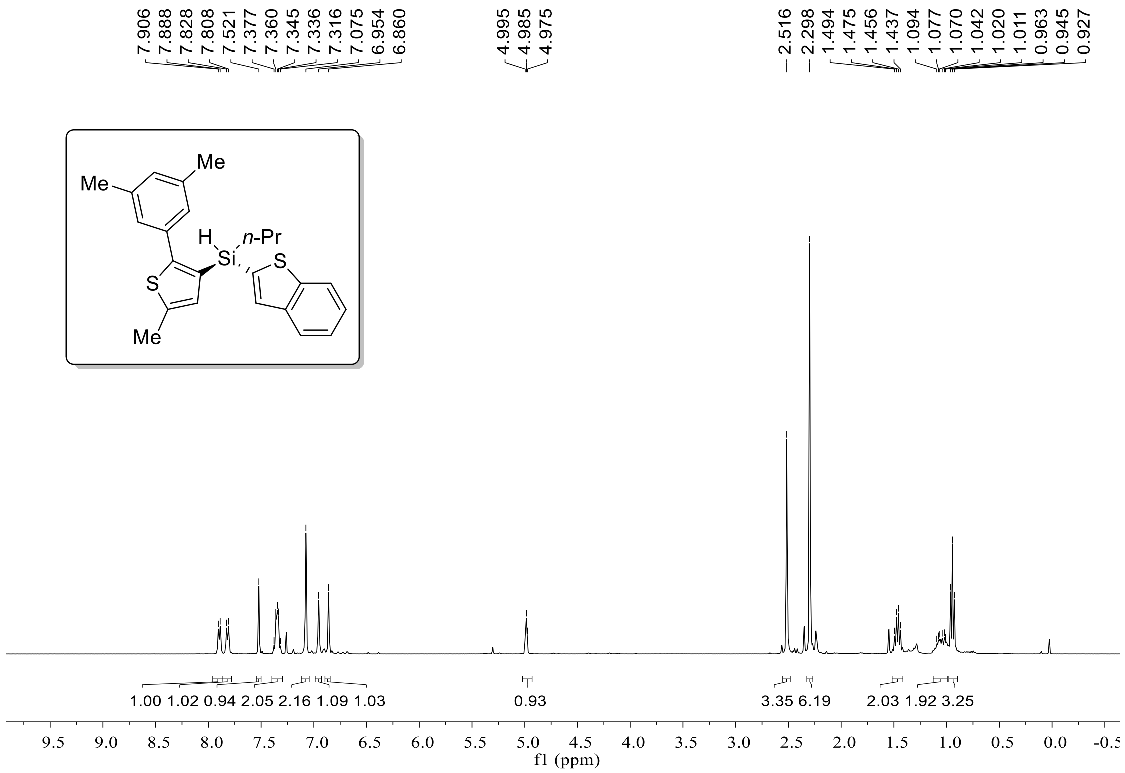

Supplementary Figure 204. ^{1}H NMR spectrum of **3in**

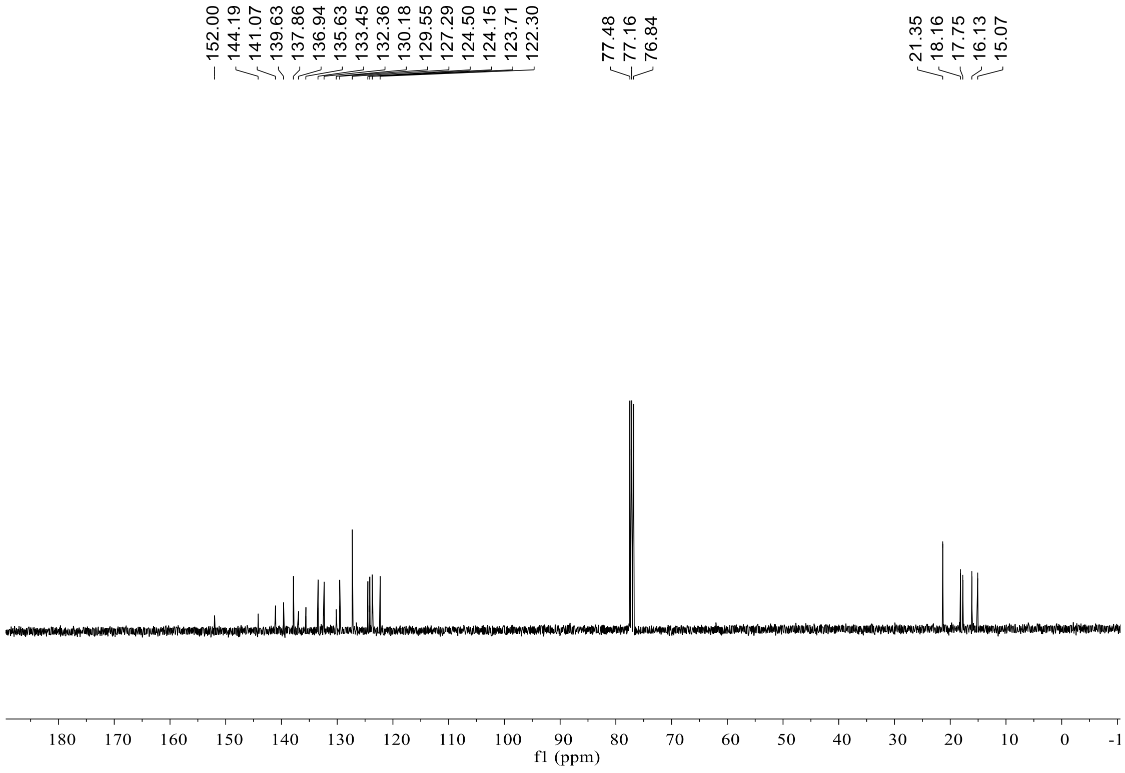

Supplementary Figure 205. ^{13}C NMR spectrum of **3in**

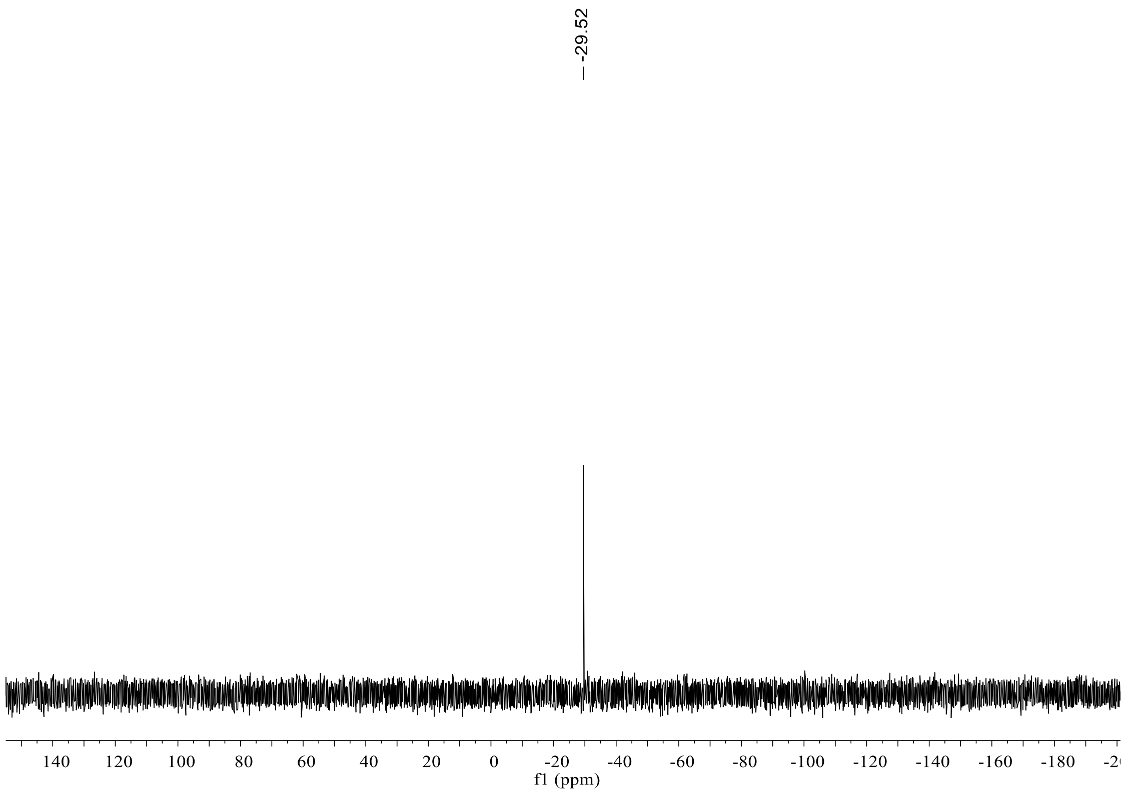

Supplementary Figure 206. ^{29}Si NMR spectrum of **3in**

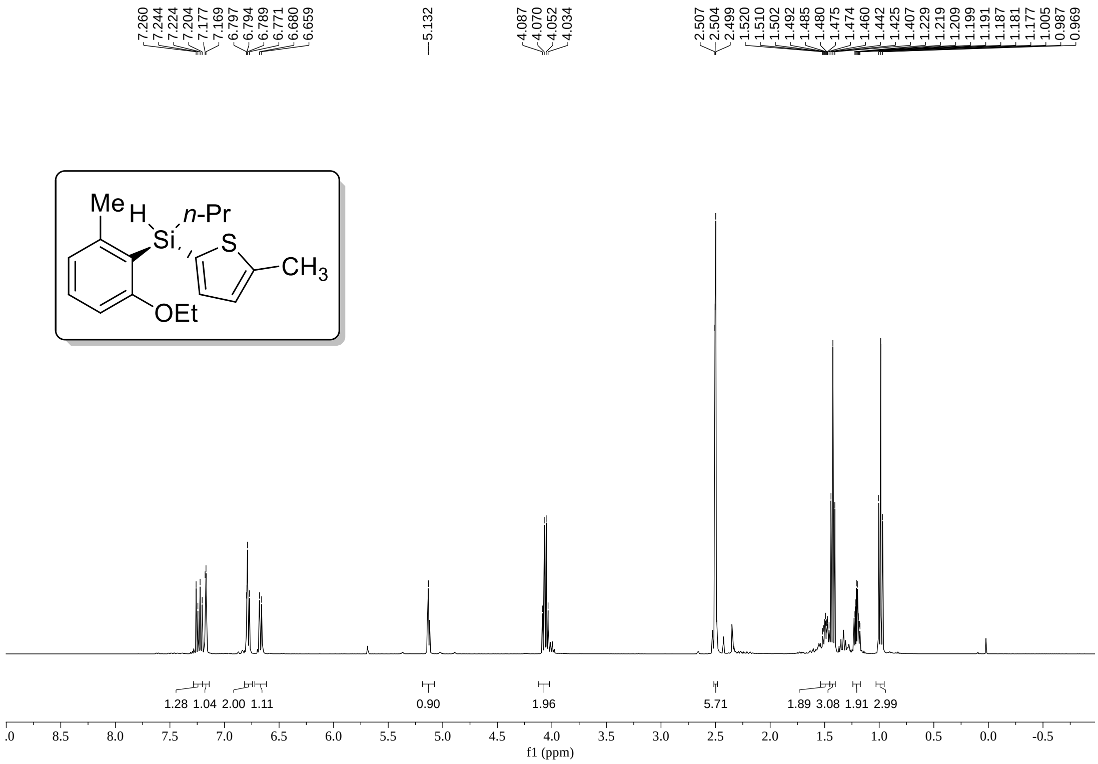

Supplementary Figure 207. ^{1}H NMR spectrum of **3ja**

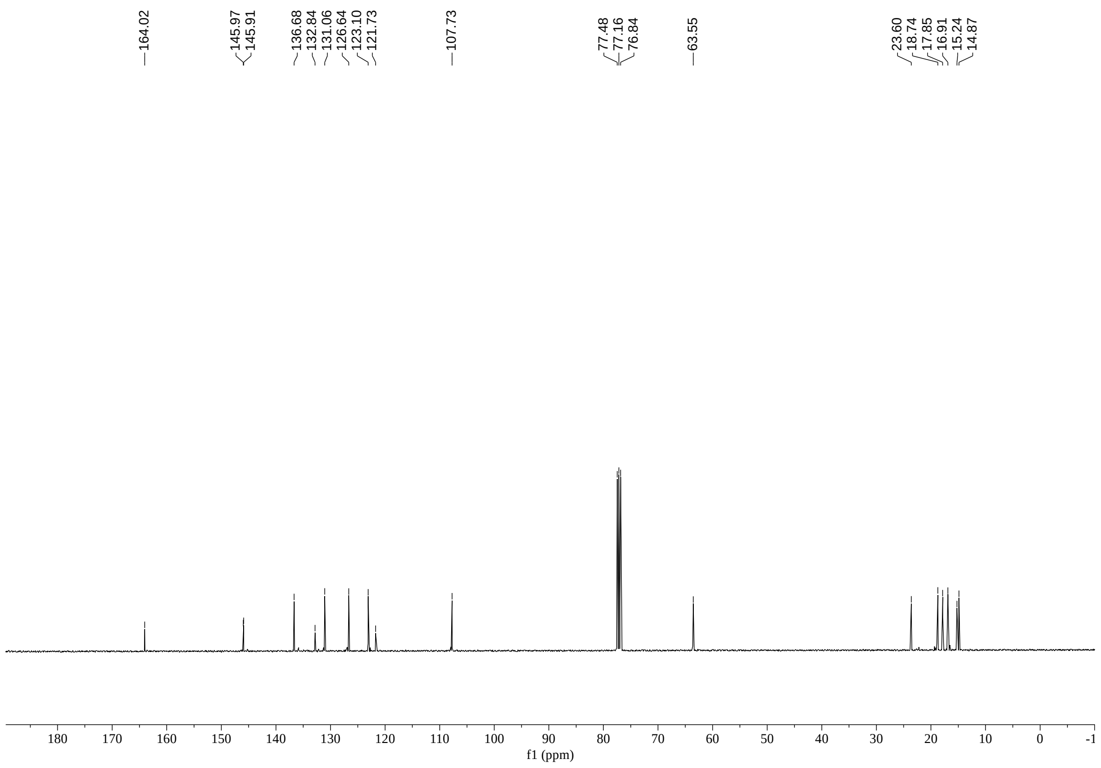

Supplementary Figure 208. ^{13}C NMR spectrum of **3ja**

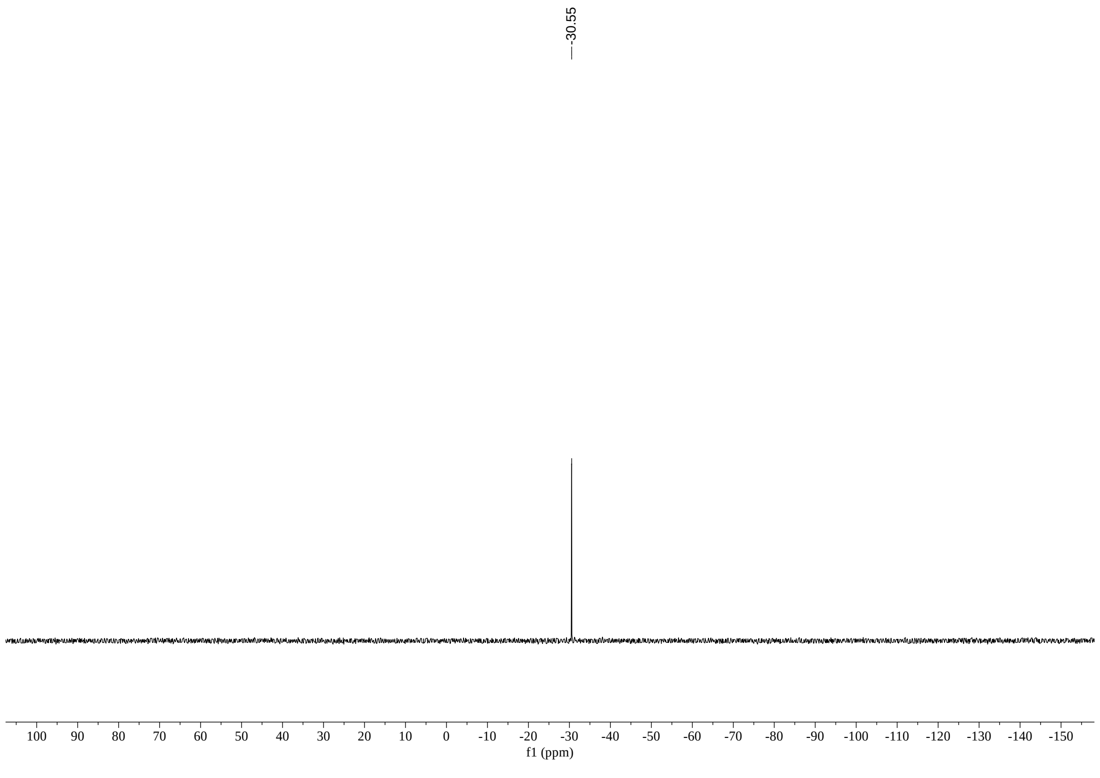

Supplementary Figure 209. ^{29}Si NMR spectrum of **3ja**

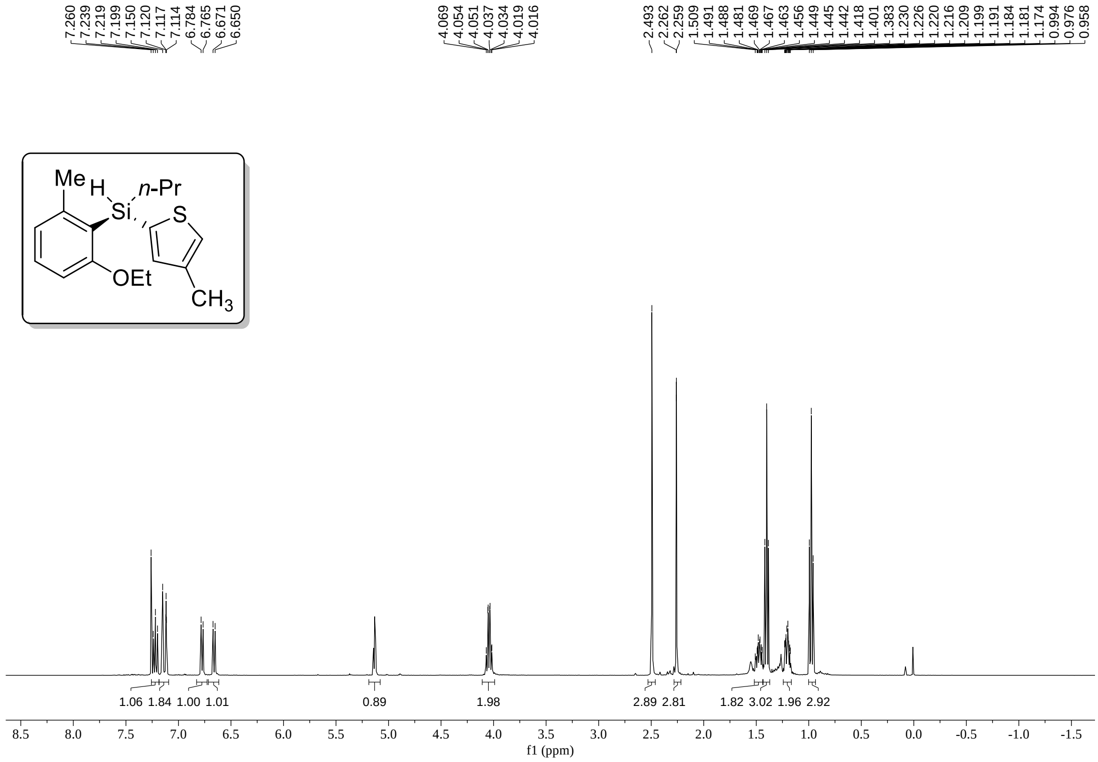

Supplementary Figure 210. ^{1}H NMR spectrum of **3ji**

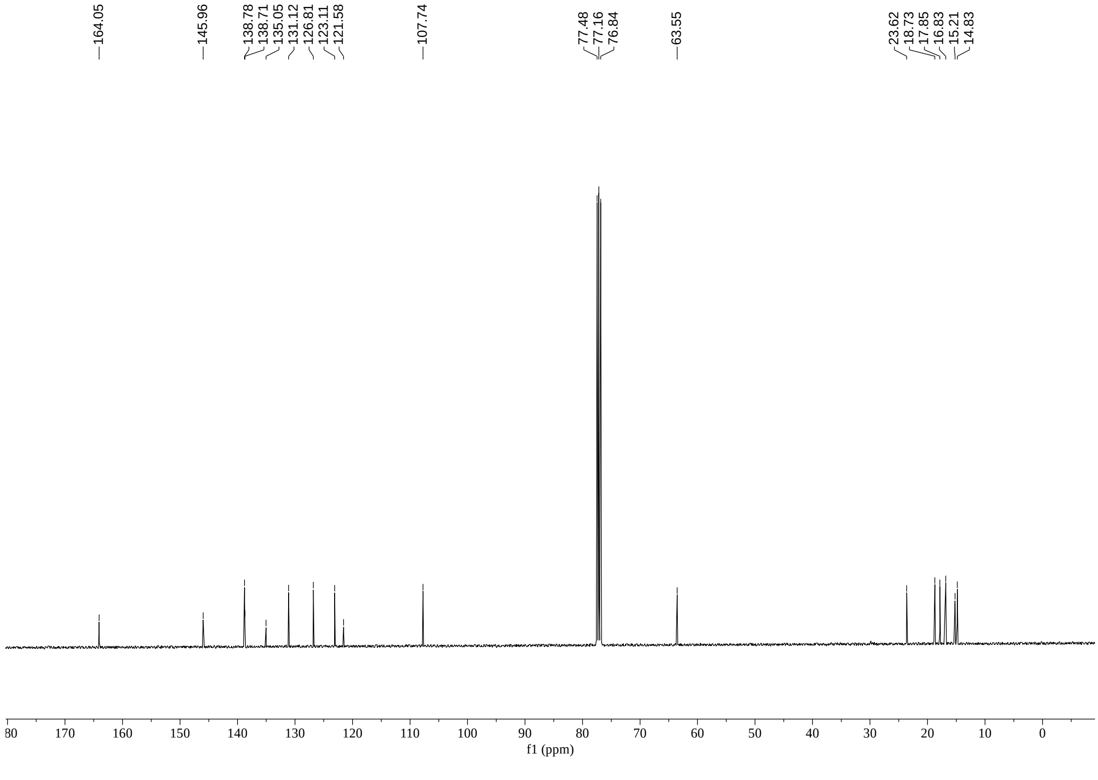

Supplementary Figure 211. ^{13}C NMR spectrum of **3ji**

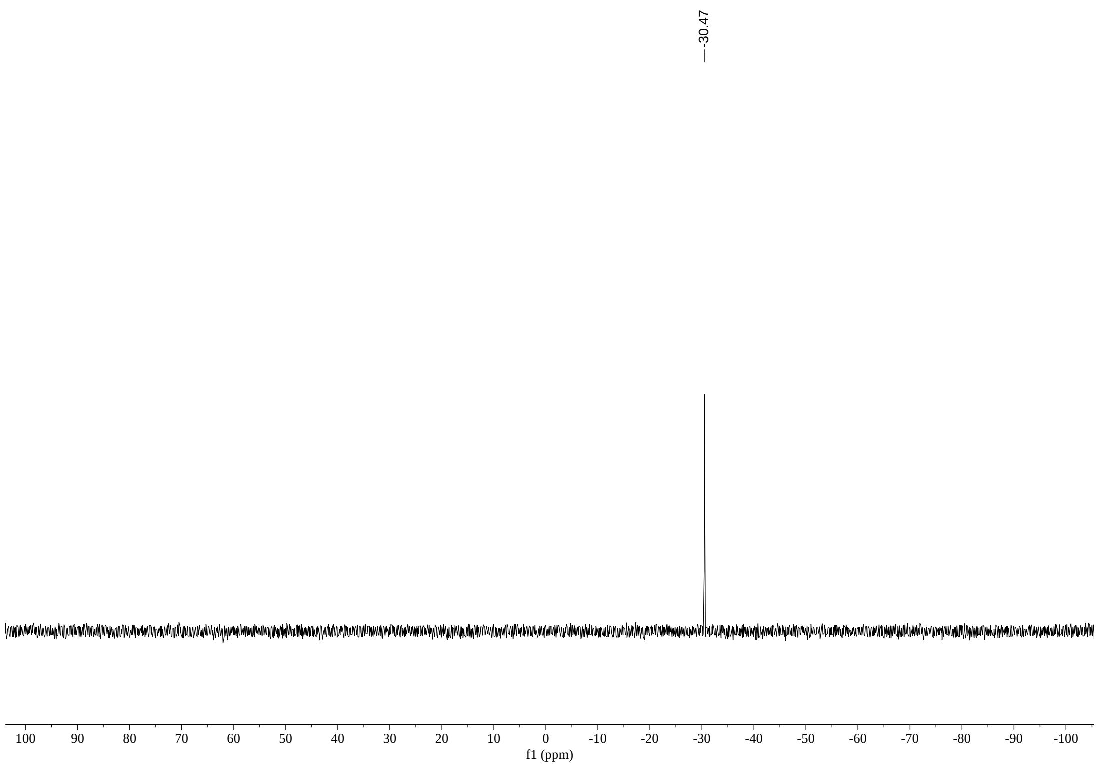

Supplementary Figure 212. ^{29}Si NMR spectrum of **3ji**

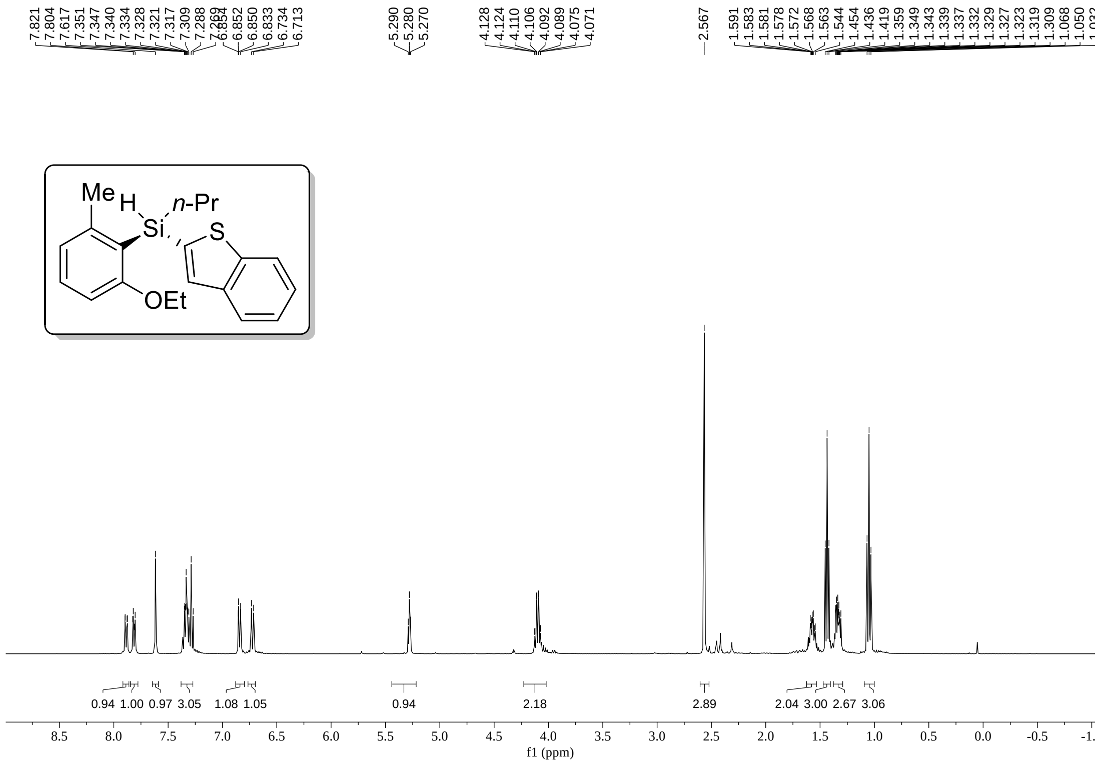

Supplementary Figure 213. ^{1}H NMR spectrum of **3jn**

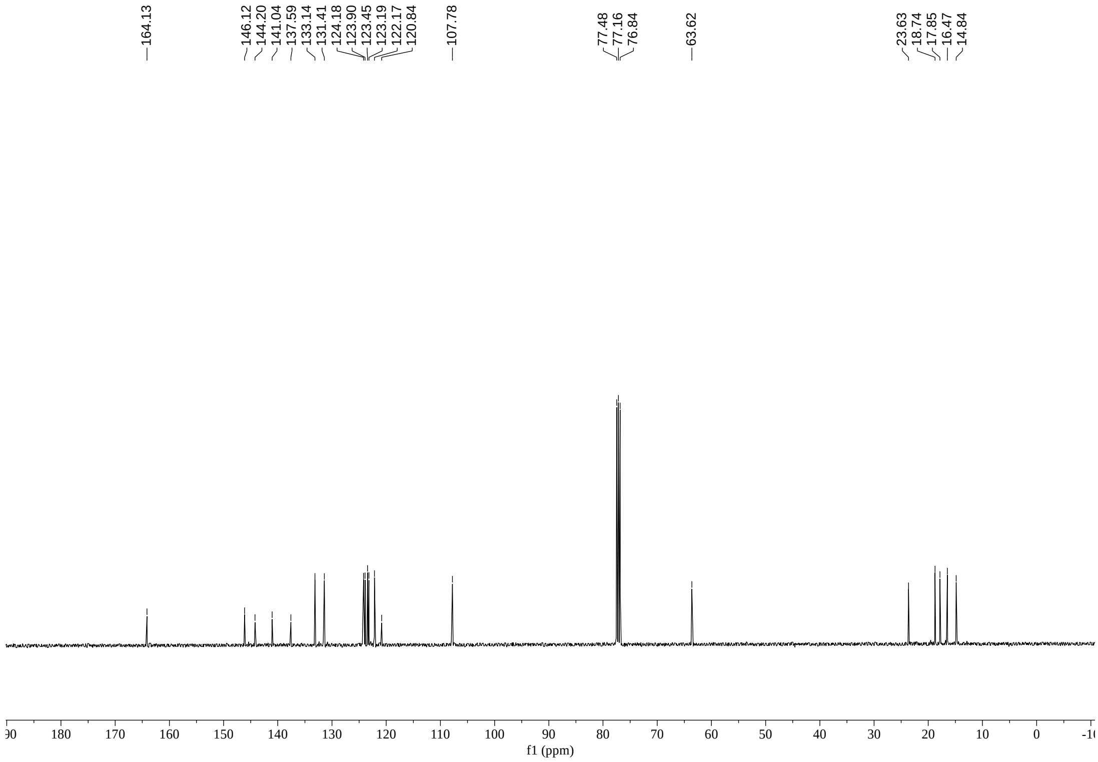

Supplementary Figure 214. ^{13}C NMR spectrum of **3jn**

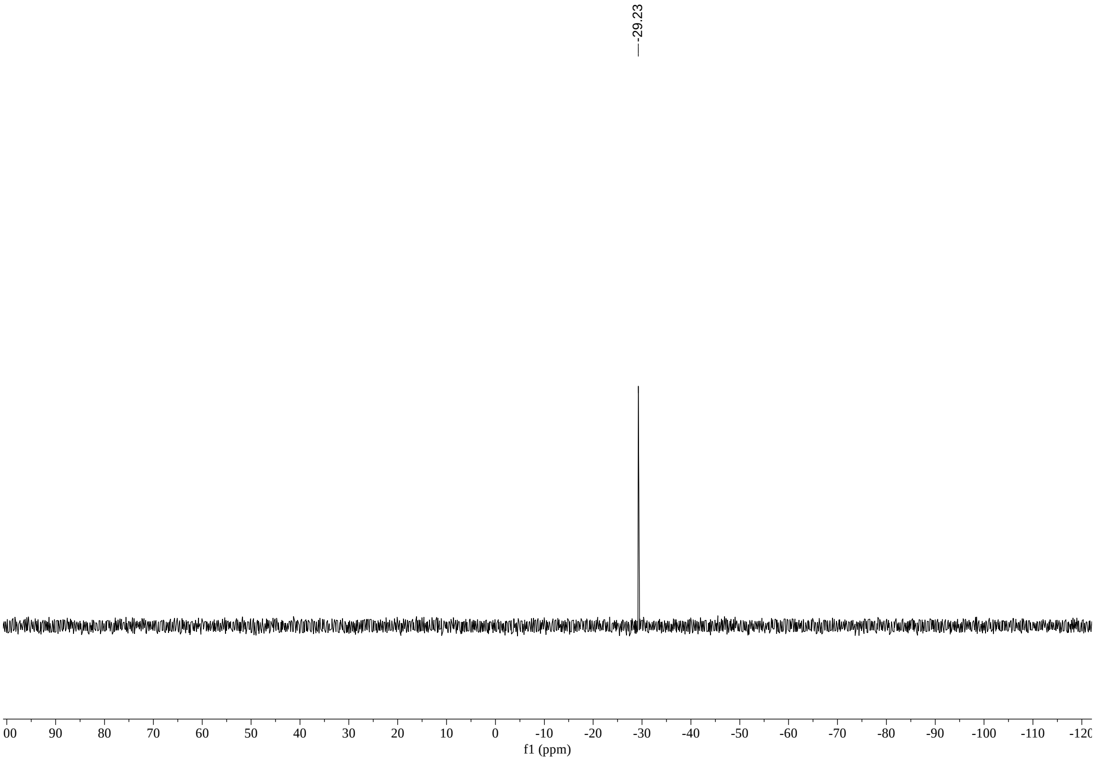

Supplementary Figure 215. ^{29}Si NMR spectrum of **3jn**

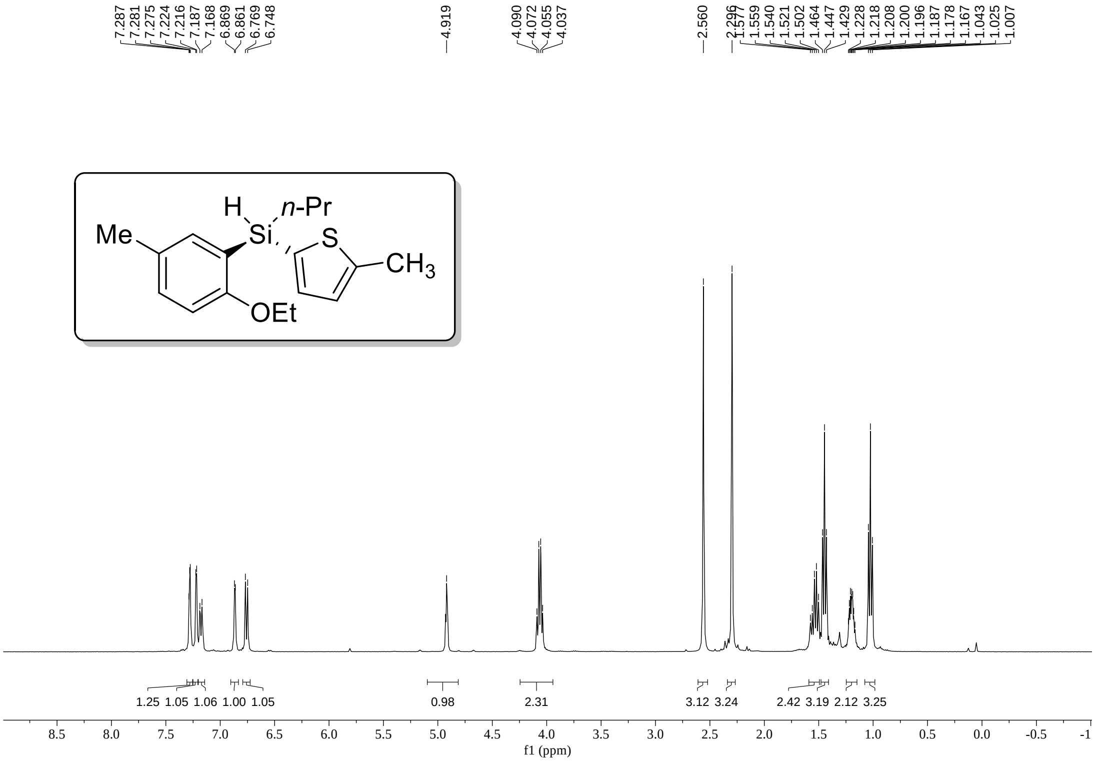

Supplementary Figure 216. ^{1}H NMR spectrum of **3ka**

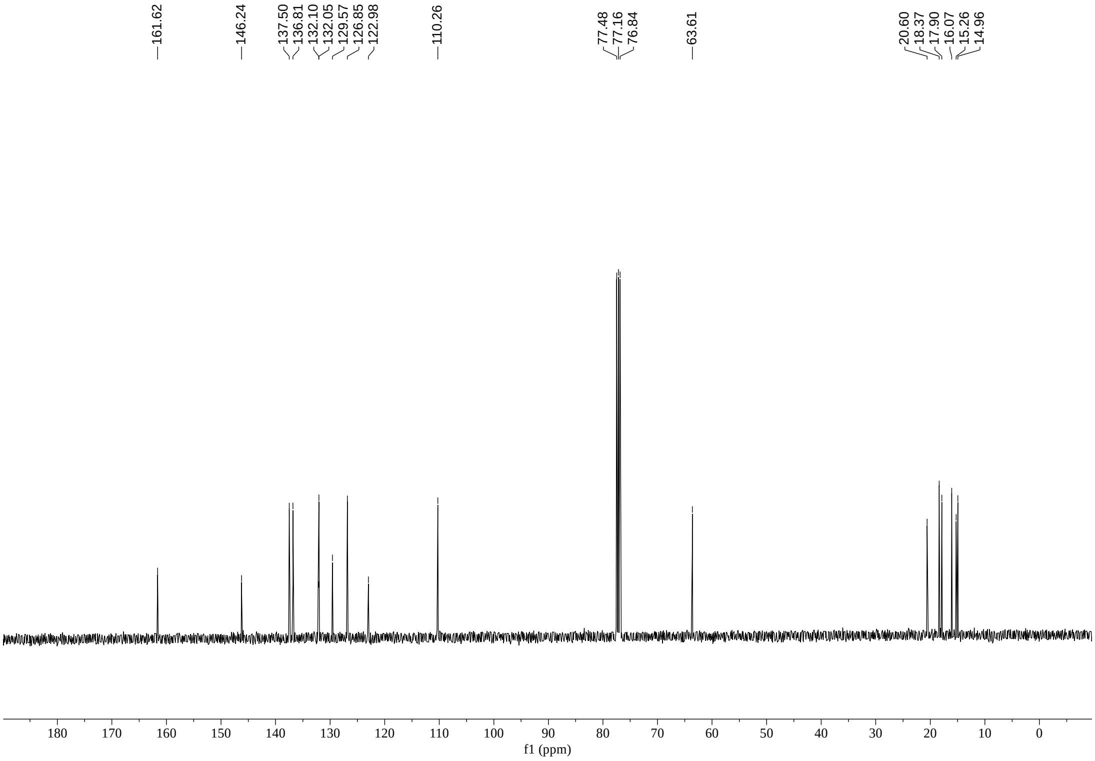

Supplementary Figure 217. ^{13}C NMR spectrum of **3ka**

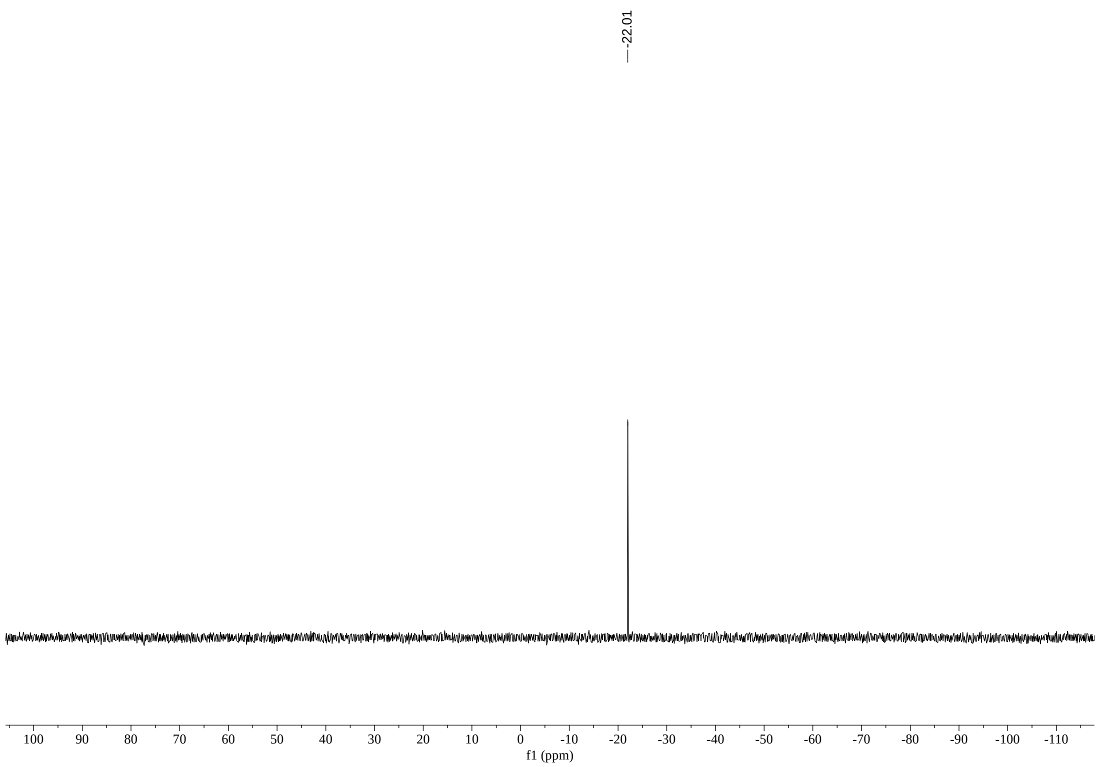

Supplementary Figure 218. ^{29}Si NMR spectrum of **3ka**

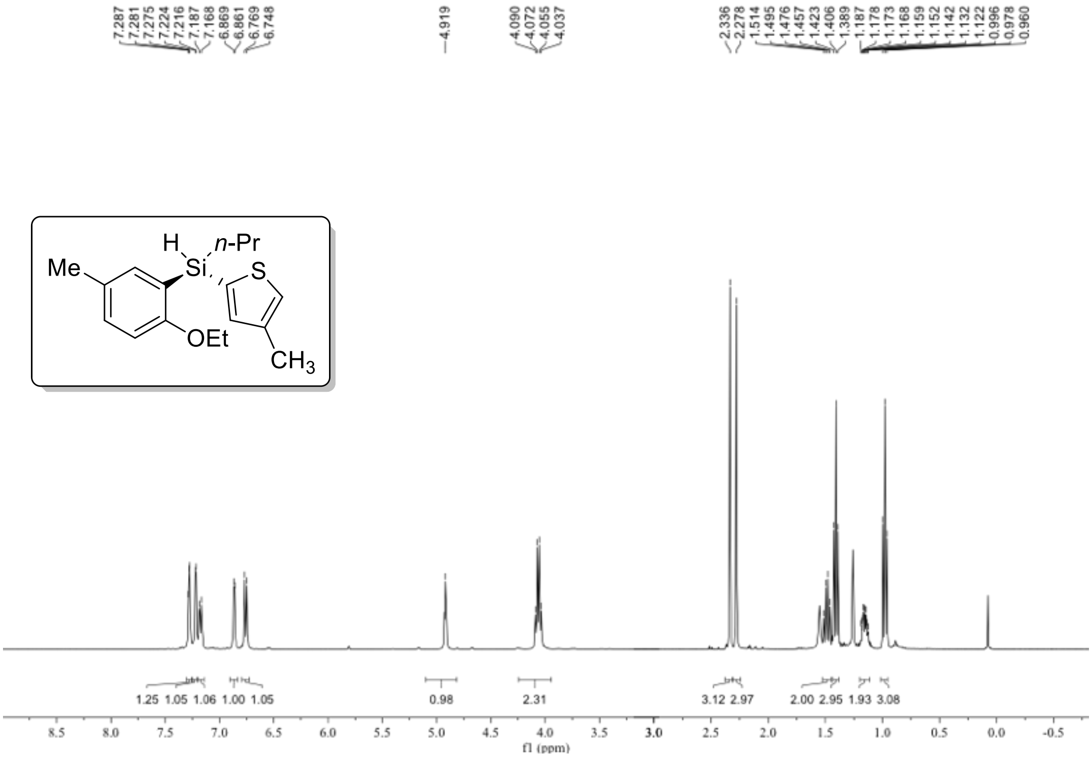

Supplementary Figure 219. ^{1}H NMR spectrum of **3ki**

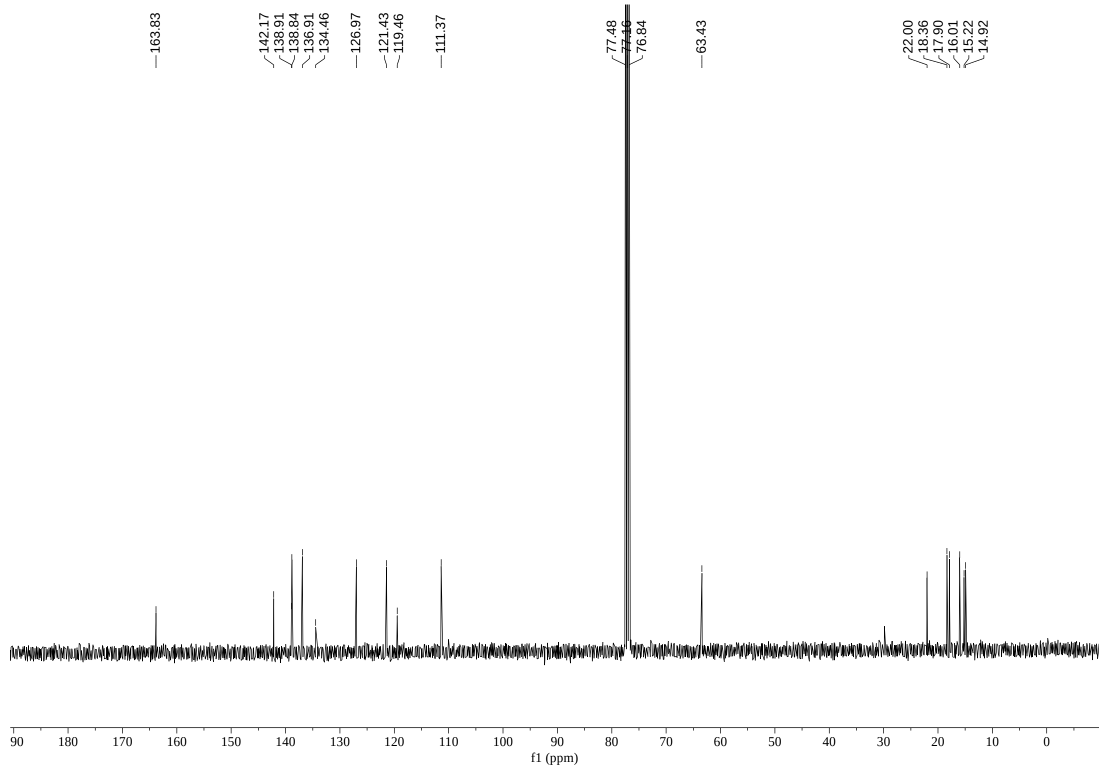

Supplementary Figure 220. ^{13}C NMR spectrum of **3ki**

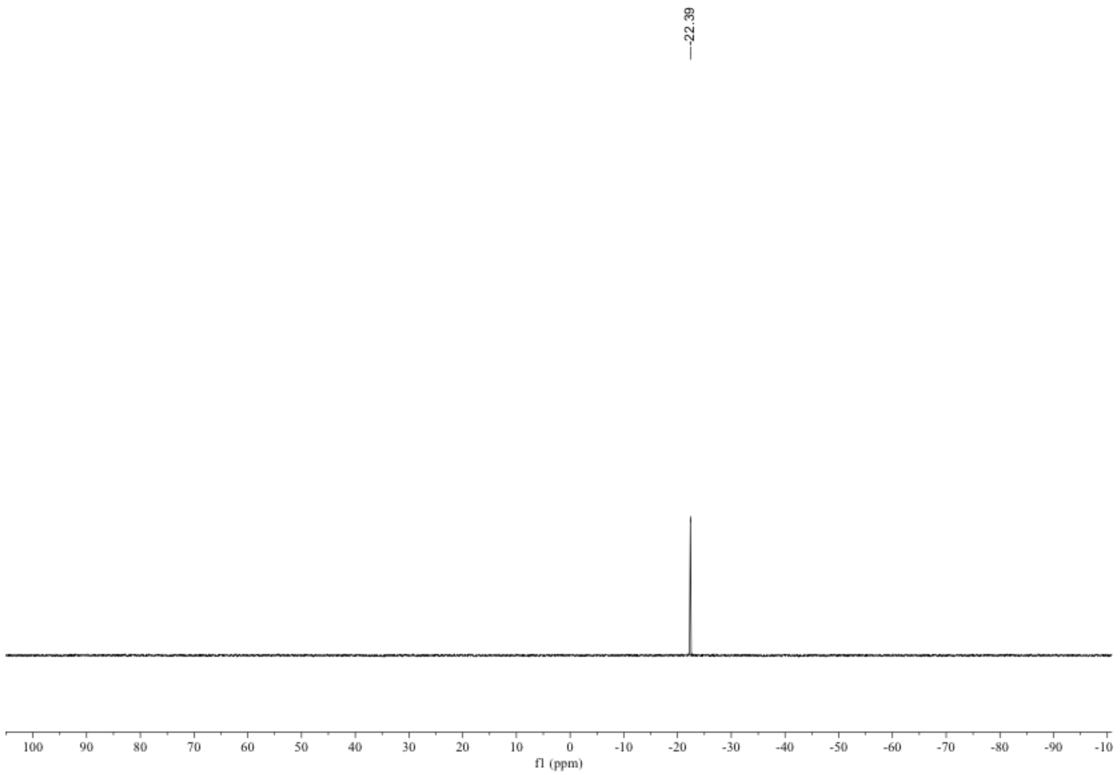

Supplementary Figure 221. ^{29}Si NMR spectrum of **3ki**

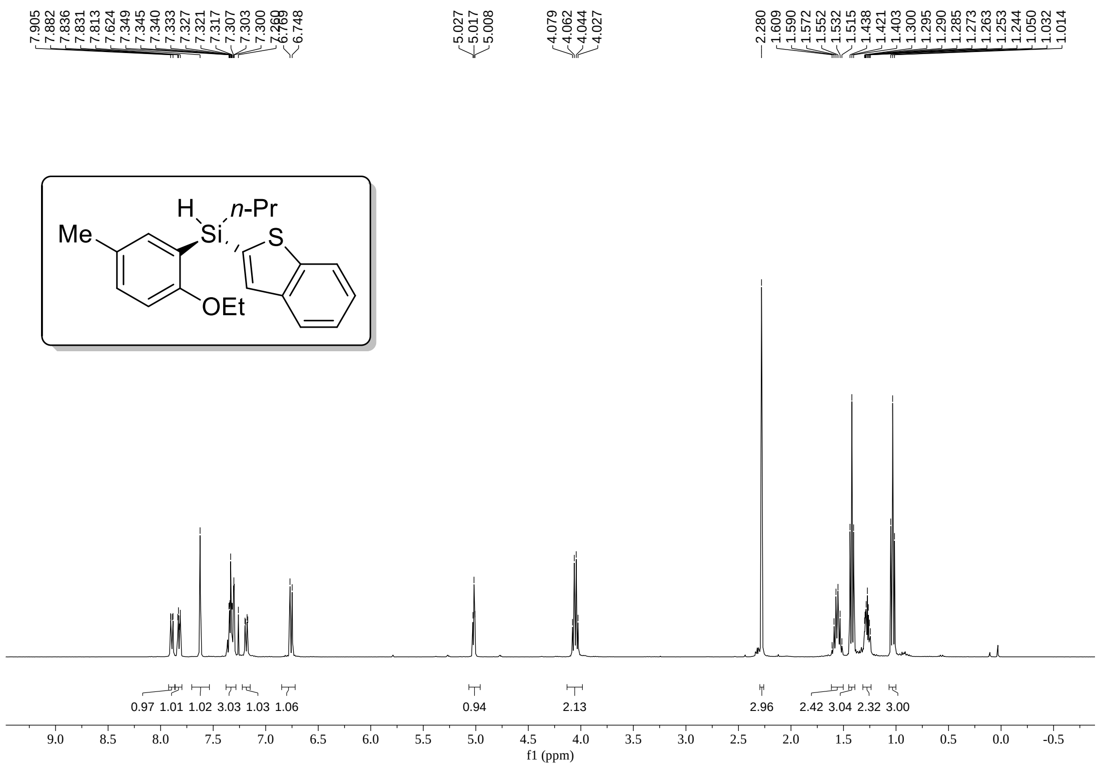

Supplementary Figure 222. ^{1}H NMR spectrum of **3kn**

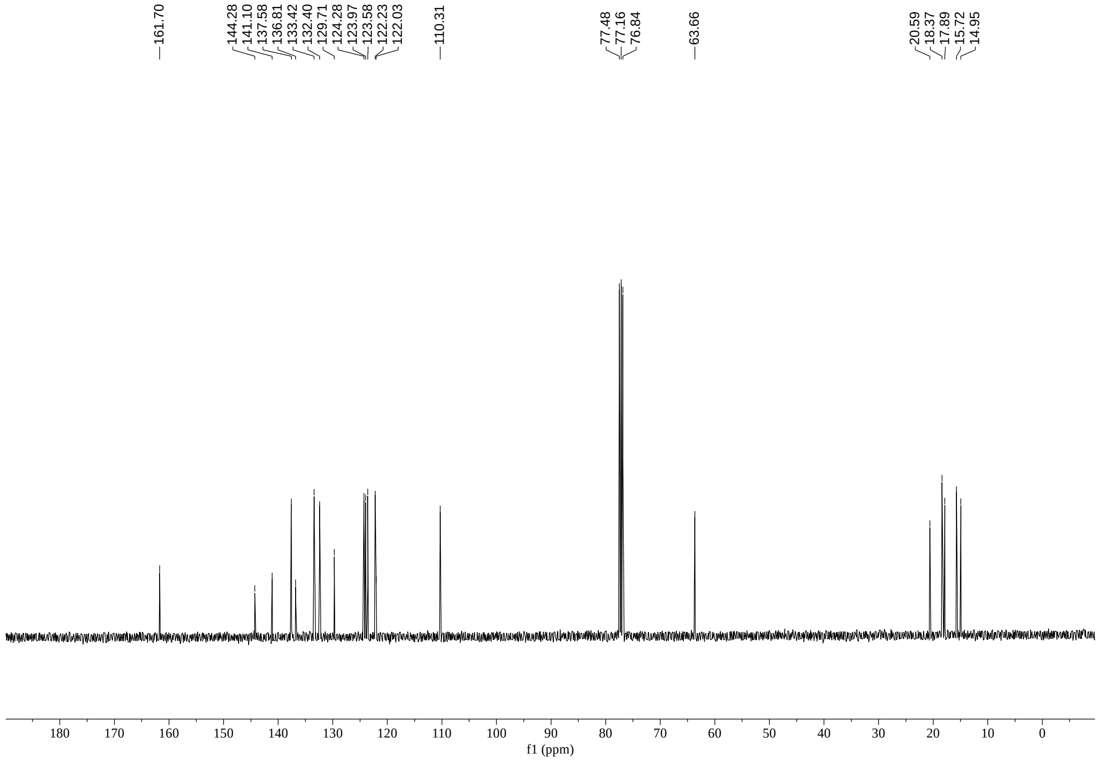

Supplementary Figure 223. ^{13}C NMR spectrum of **3kn**

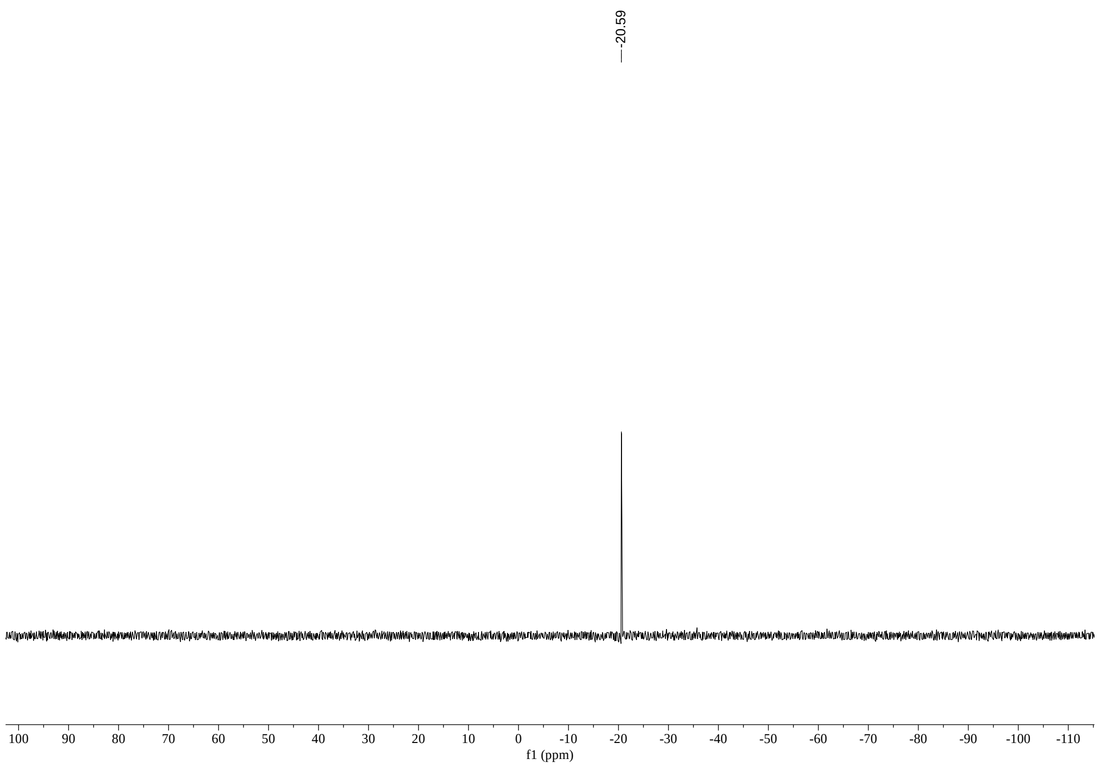

Supplementary Figure 224. ^{29}Si NMR spectrum of **3kn**

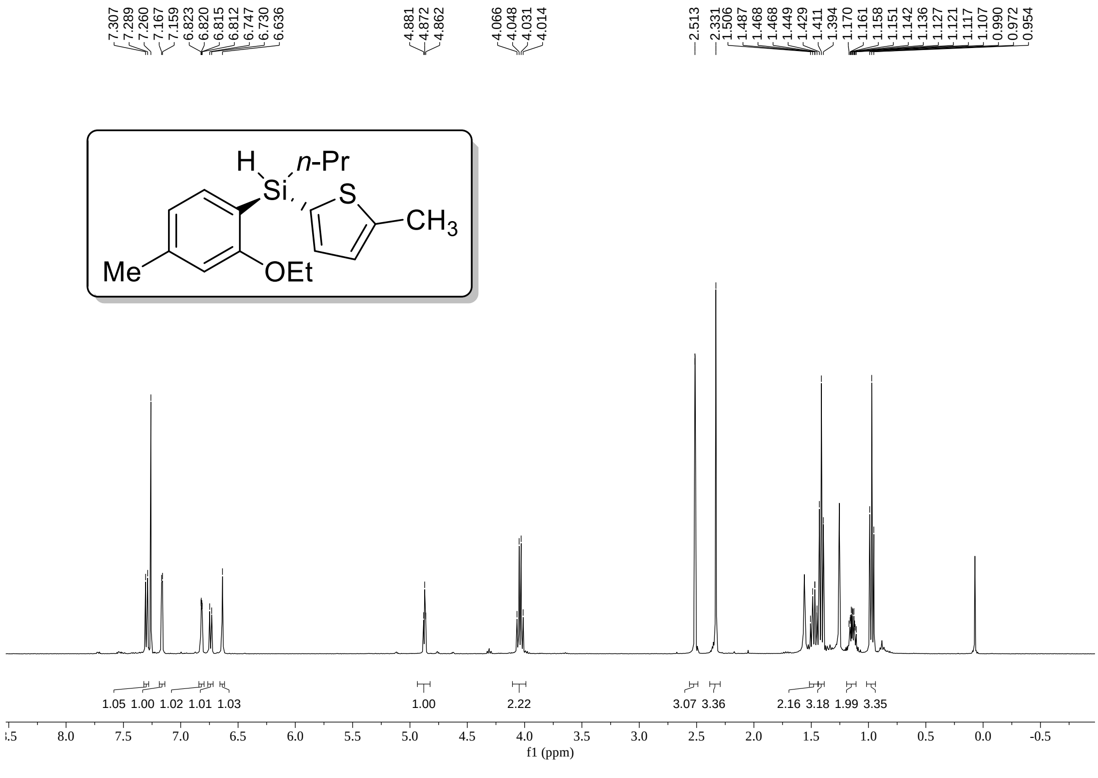

Supplementary Figure 225. ^{1}H NMR spectrum of **3la**

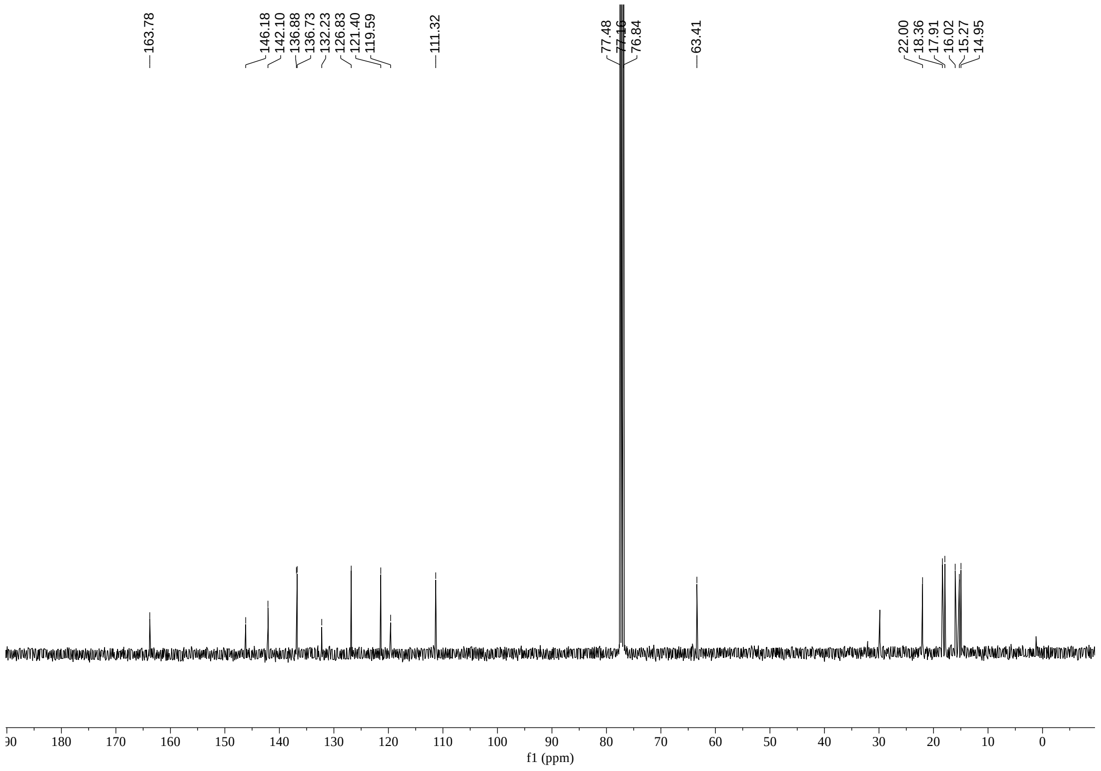

Supplementary Figure 226. ^{13}C NMR spectrum of **3la**

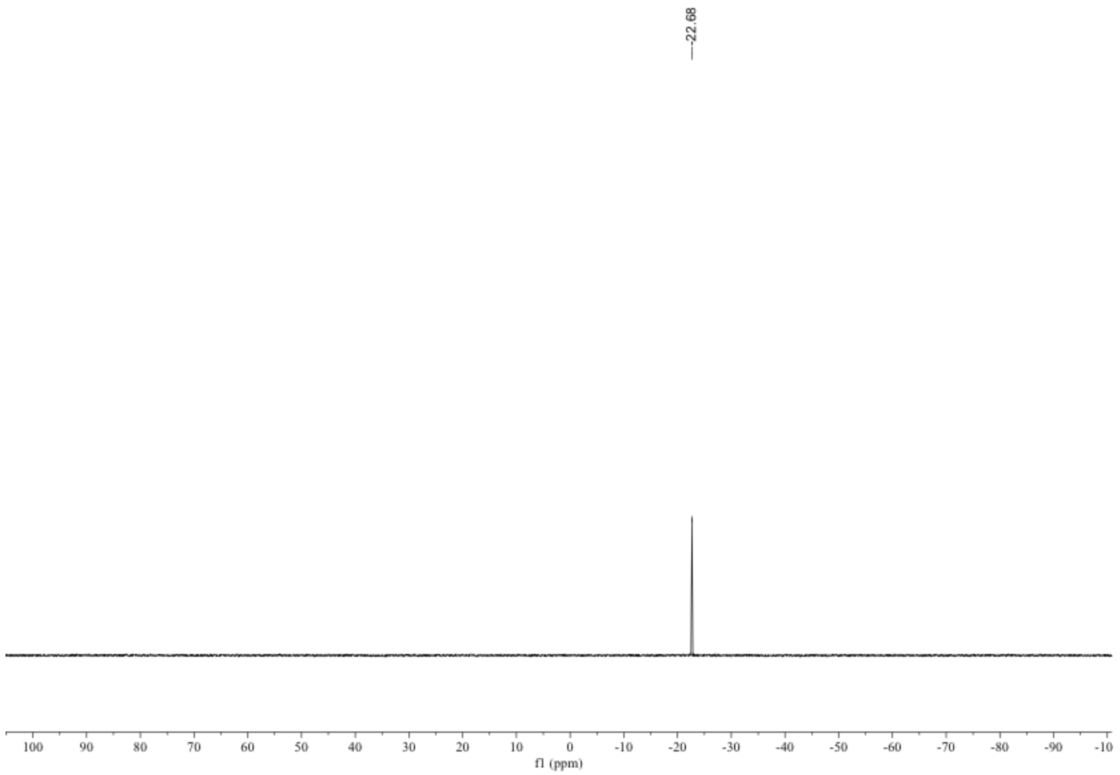

Supplementary Figure 227. ^{29}Si NMR spectrum of **3la**

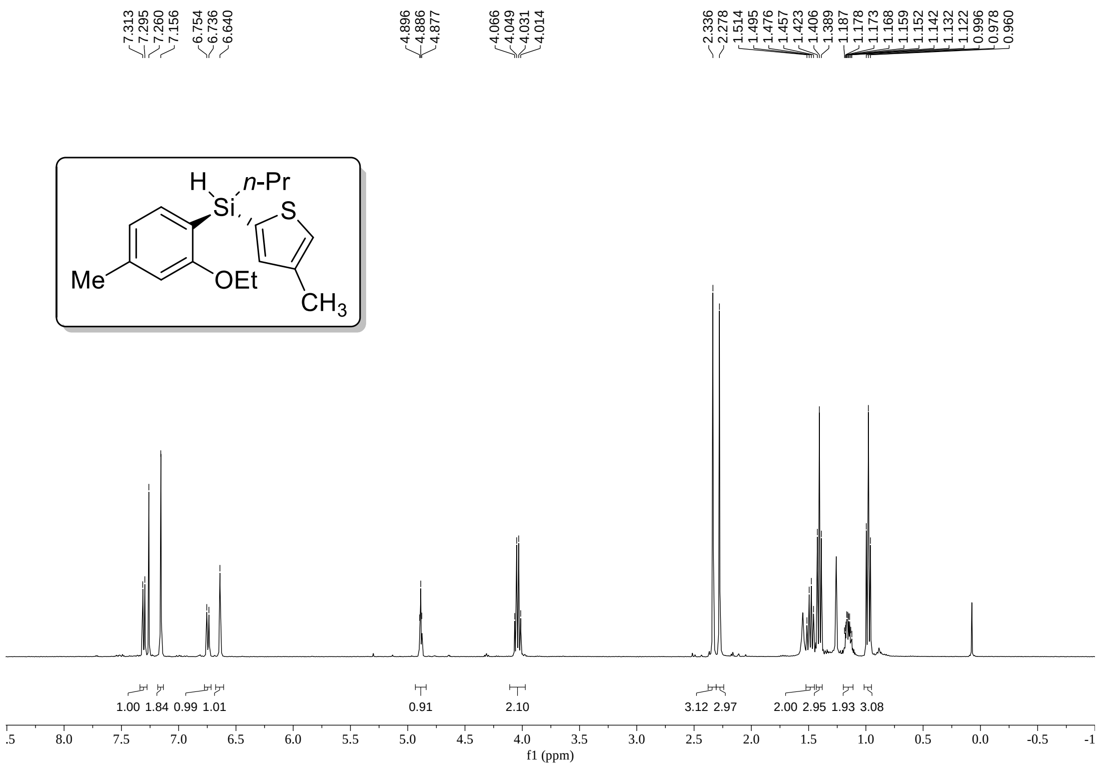

Supplementary Figure 228. ^{1}H NMR spectrum of **3li**

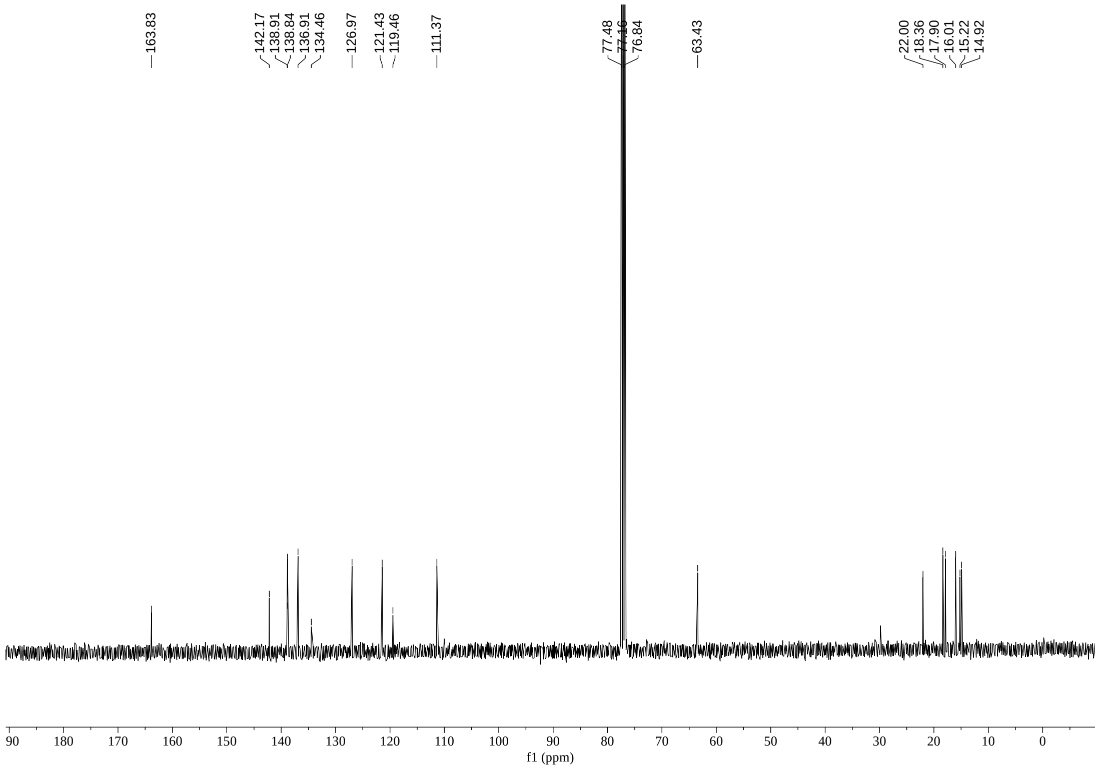

Supplementary Figure 229. ^{13}C NMR spectrum of **3li**

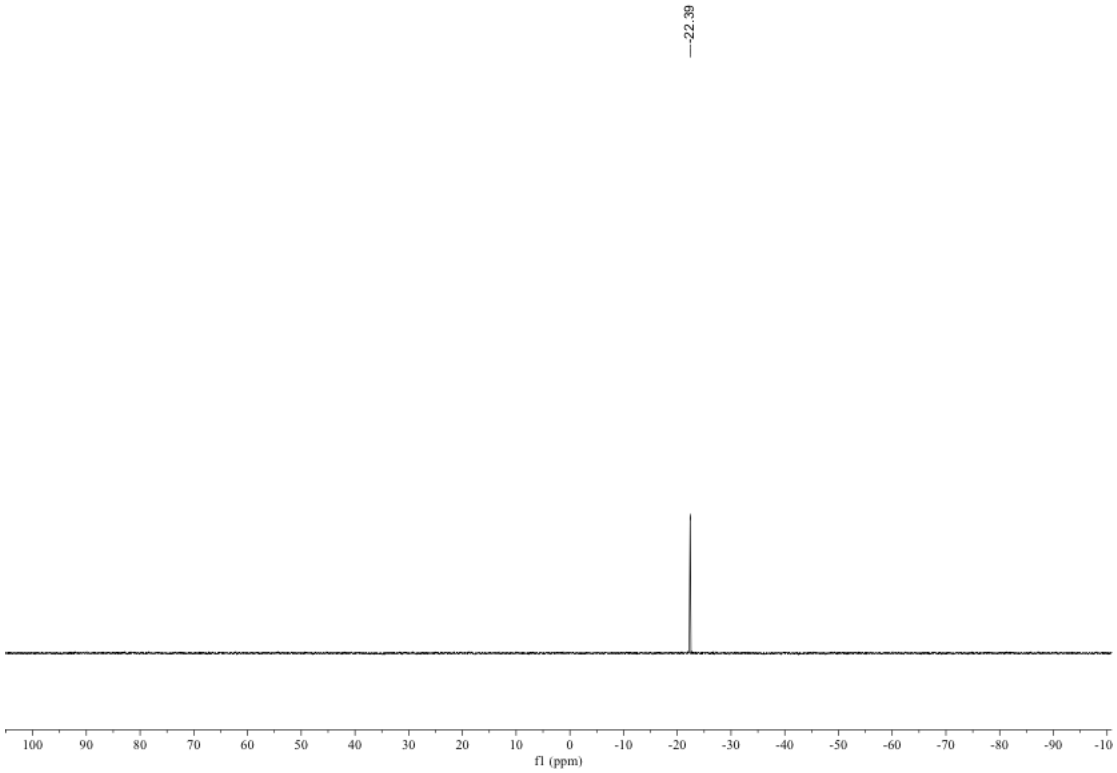

Supplementary Figure 230. ^{29}Si NMR spectrum of **3li**

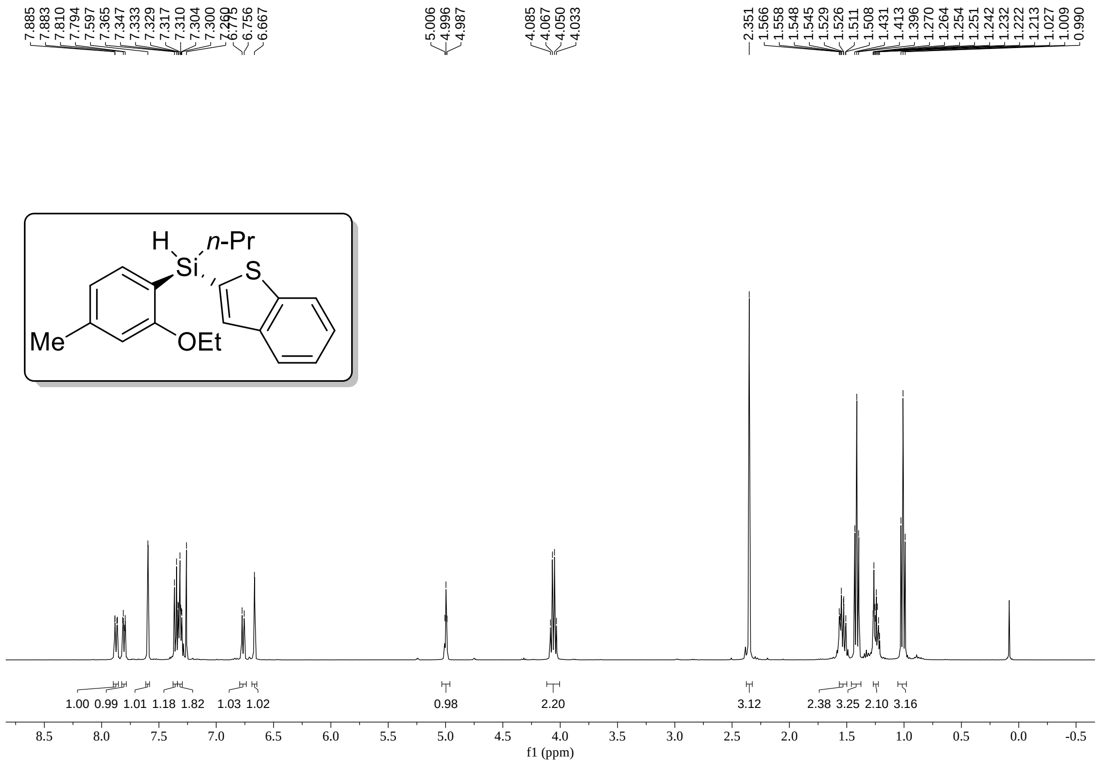

Supplementary Figure 231. ^{1}H NMR spectrum of **3ln**

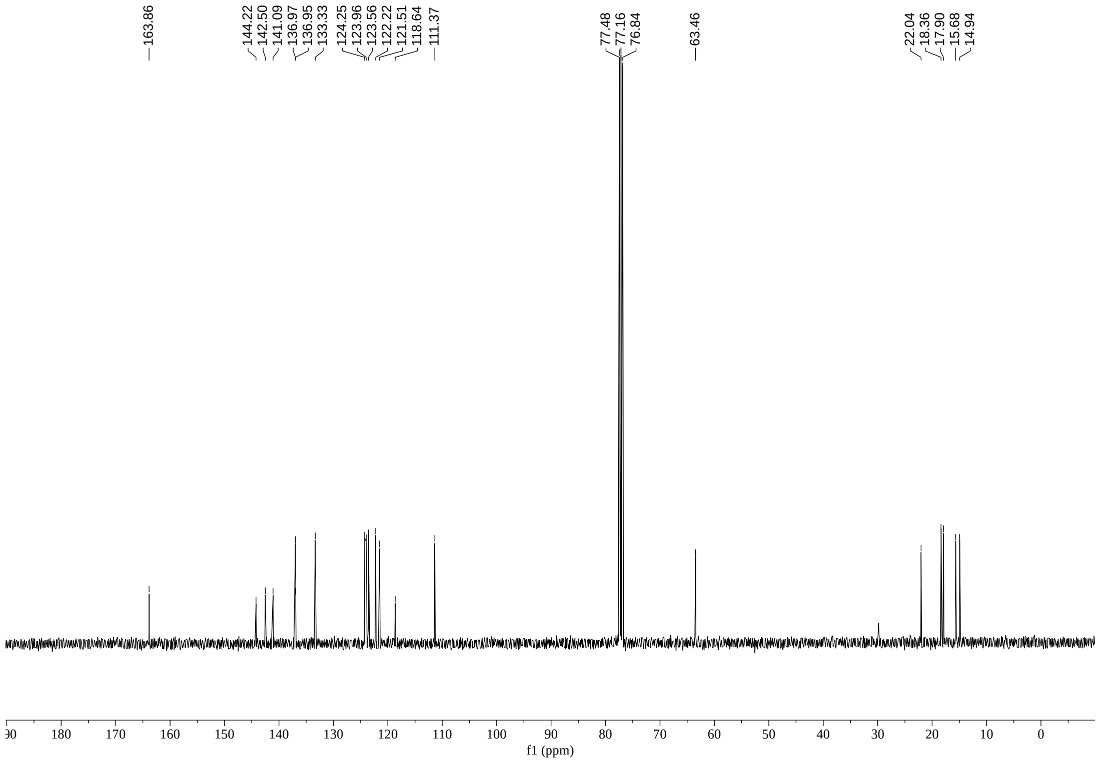

Supplementary Figure 232. ^{13}C NMR spectrum of **3ln**

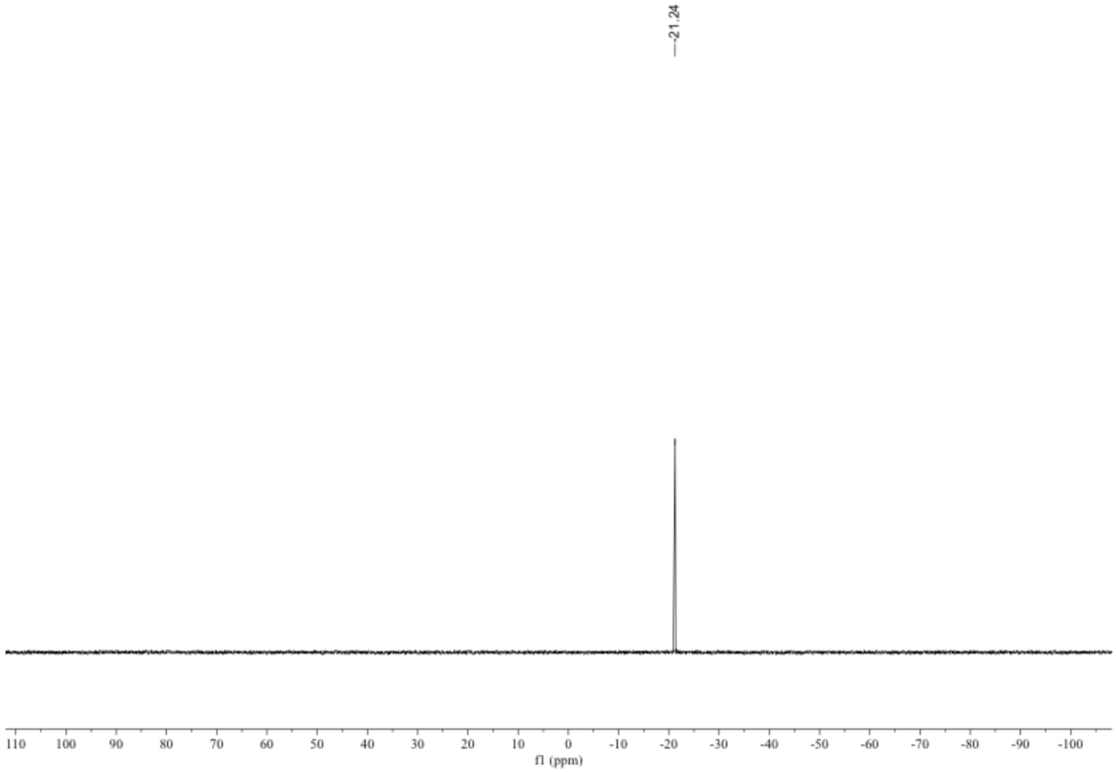

Supplementary Figure 233. ^{29}Si NMR spectrum of **3ln**

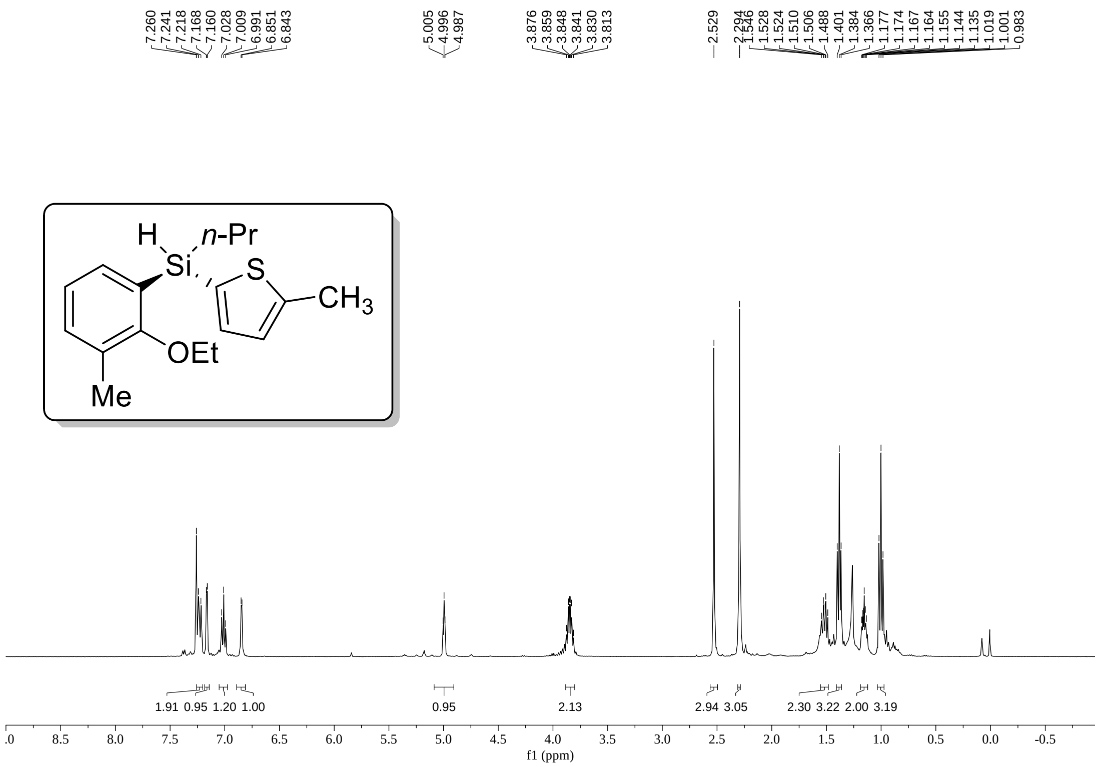

Supplementary Figure 234. ^{1}H NMR spectrum of **3ma**

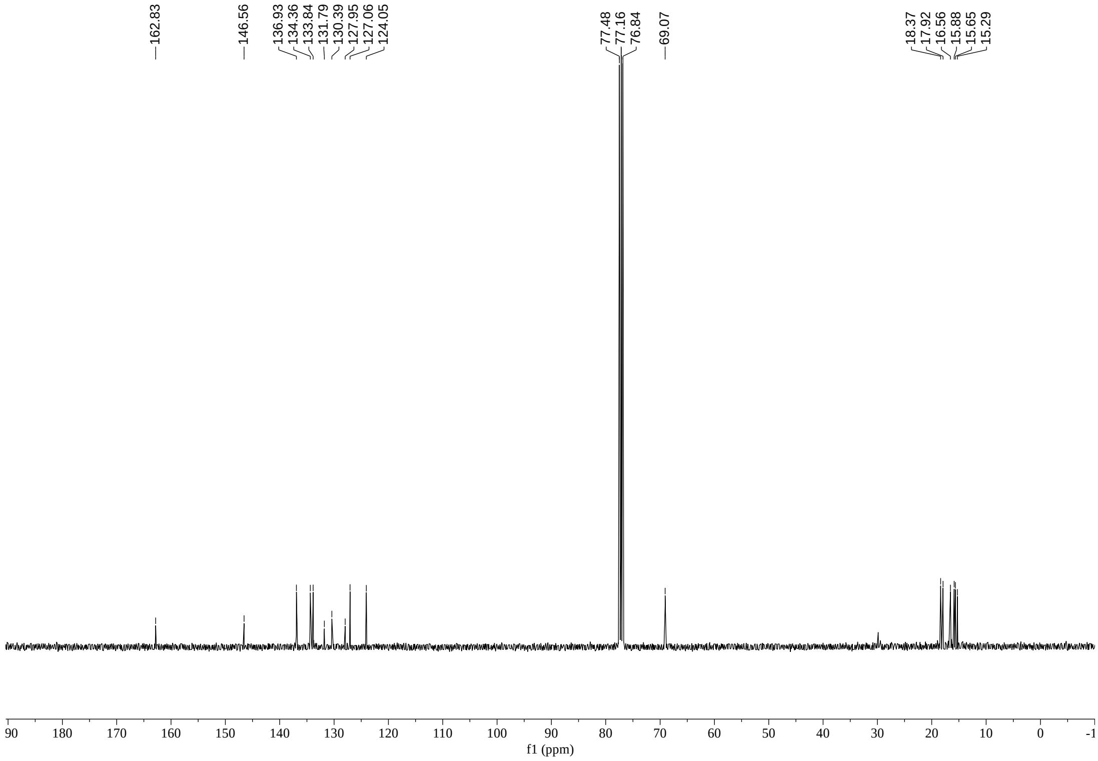

Supplementary Figure 235. ^{13}C NMR spectrum of **3ma**

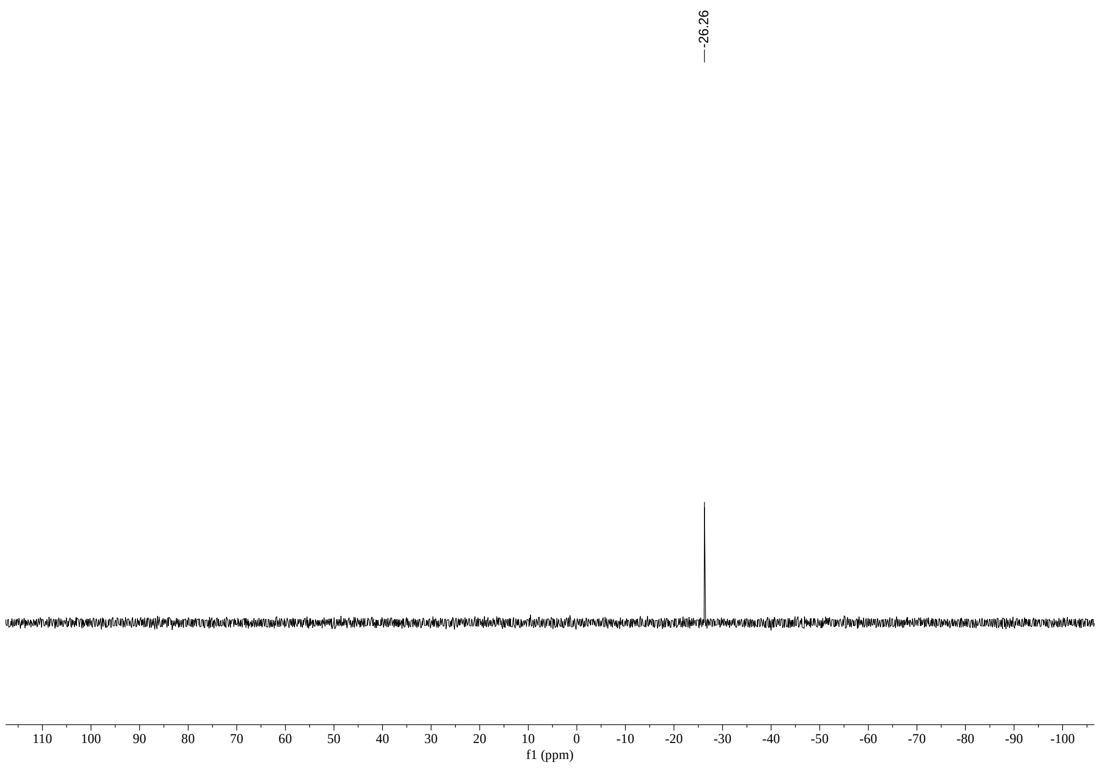

Supplementary Figure 236. ^{29}Si NMR spectrum of **3ma**

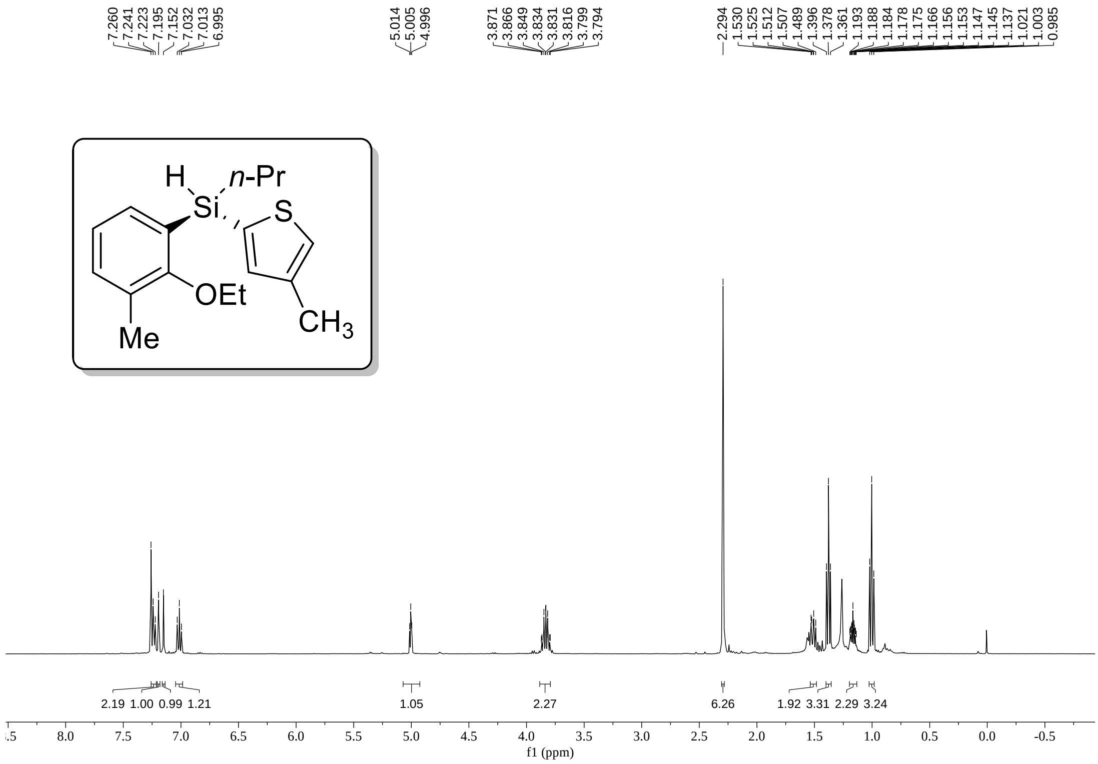

Supplementary Figure 237. ^{1}H NMR spectrum of **3mi**

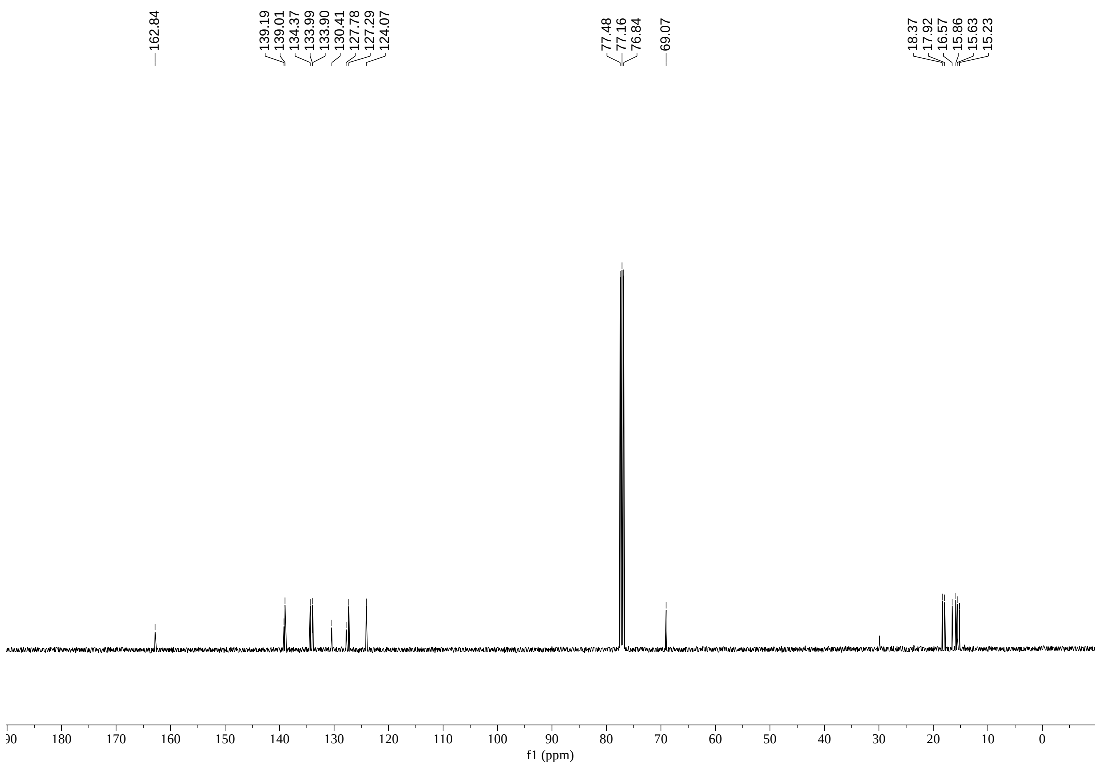

Supplementary Figure 238. ^{13}C NMR spectrum of **3mi**

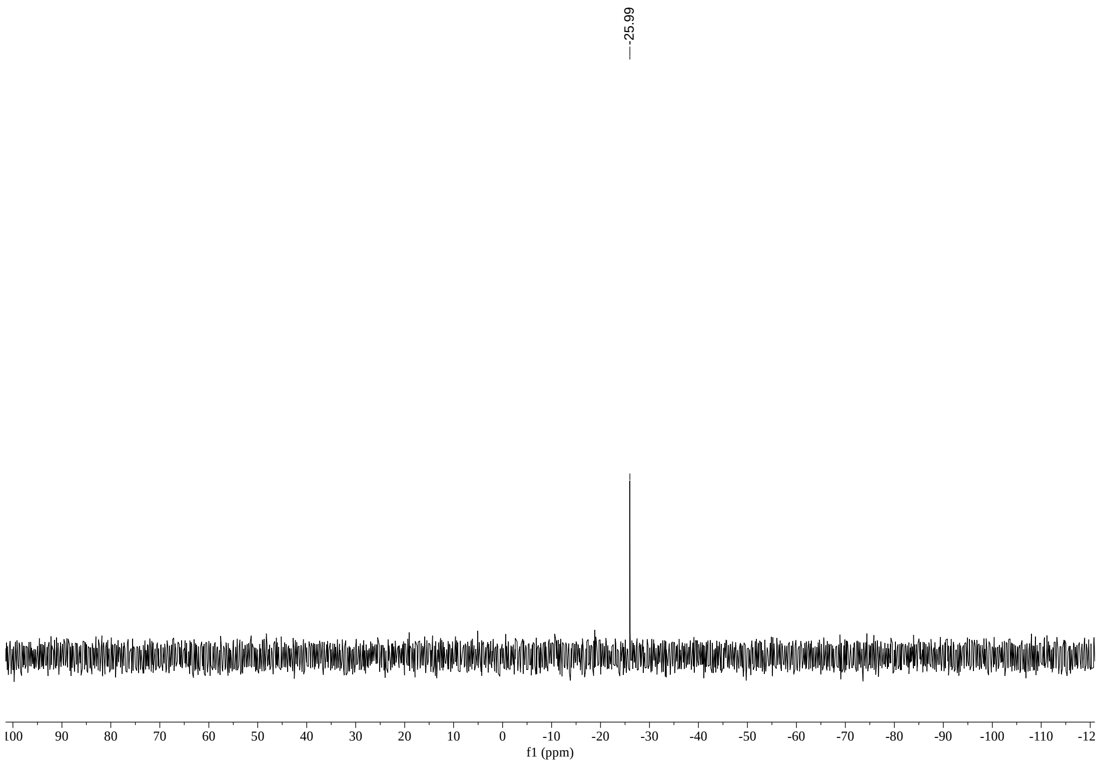

Supplementary Figure 239. ^{29}Si NMR spectrum of **3mi**

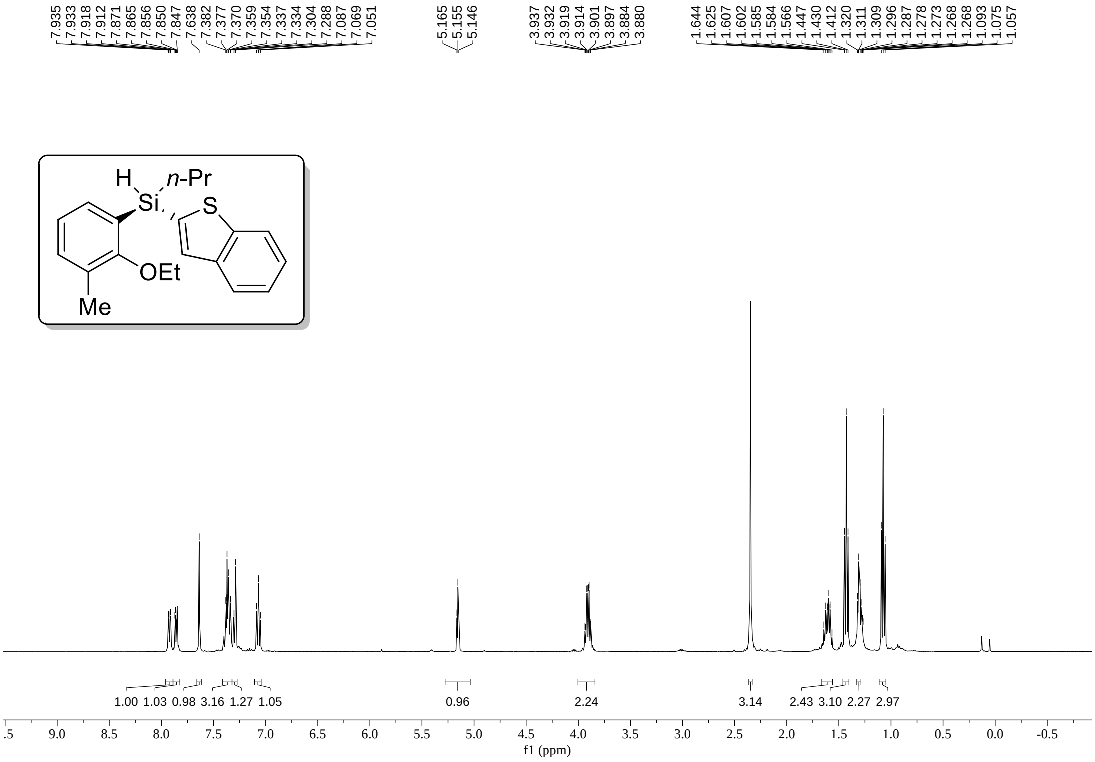

Supplementary Figure 240. ^{1}H NMR spectrum of **3mn**

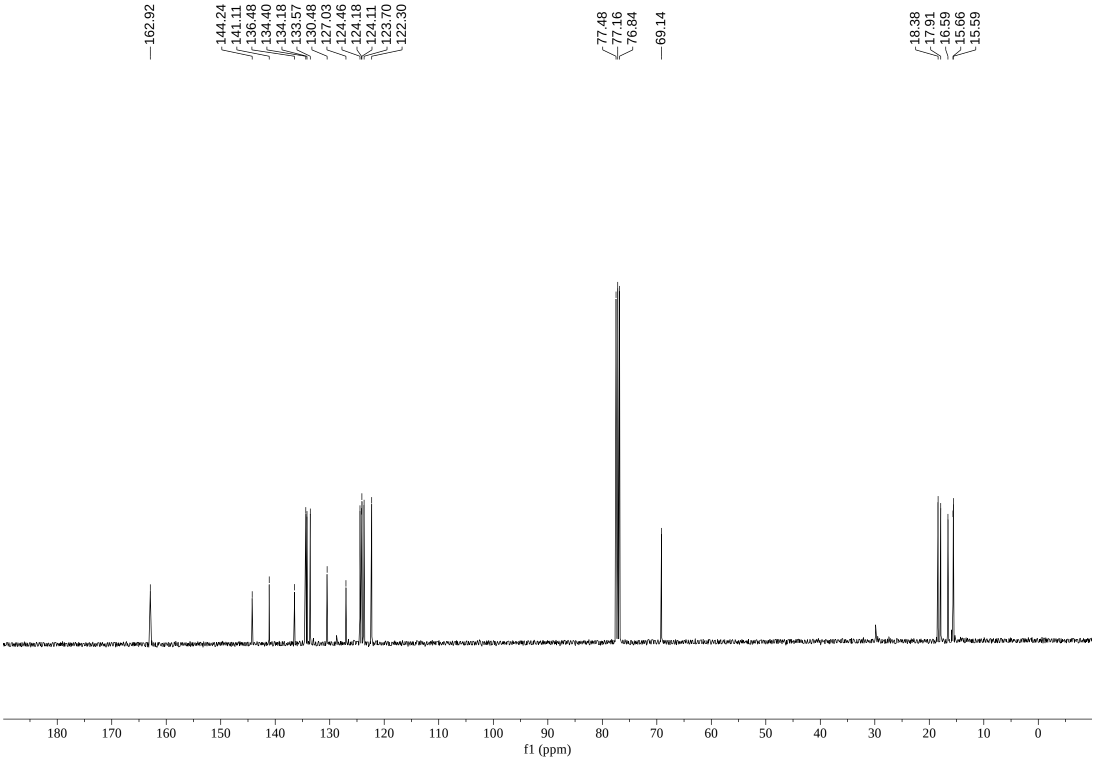

Supplementary Figure 241. ^{13}C NMR spectrum of **3mn**

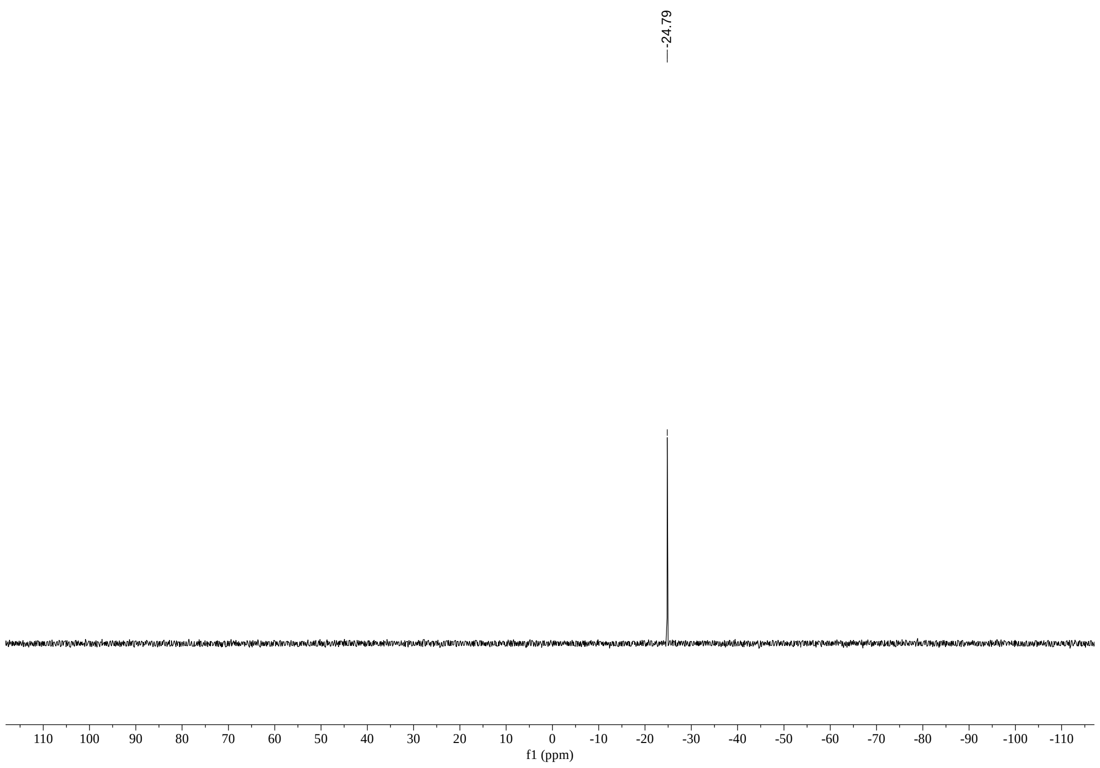

Supplementary Figure 242. ^{29}Si NMR spectrum of **3mn**

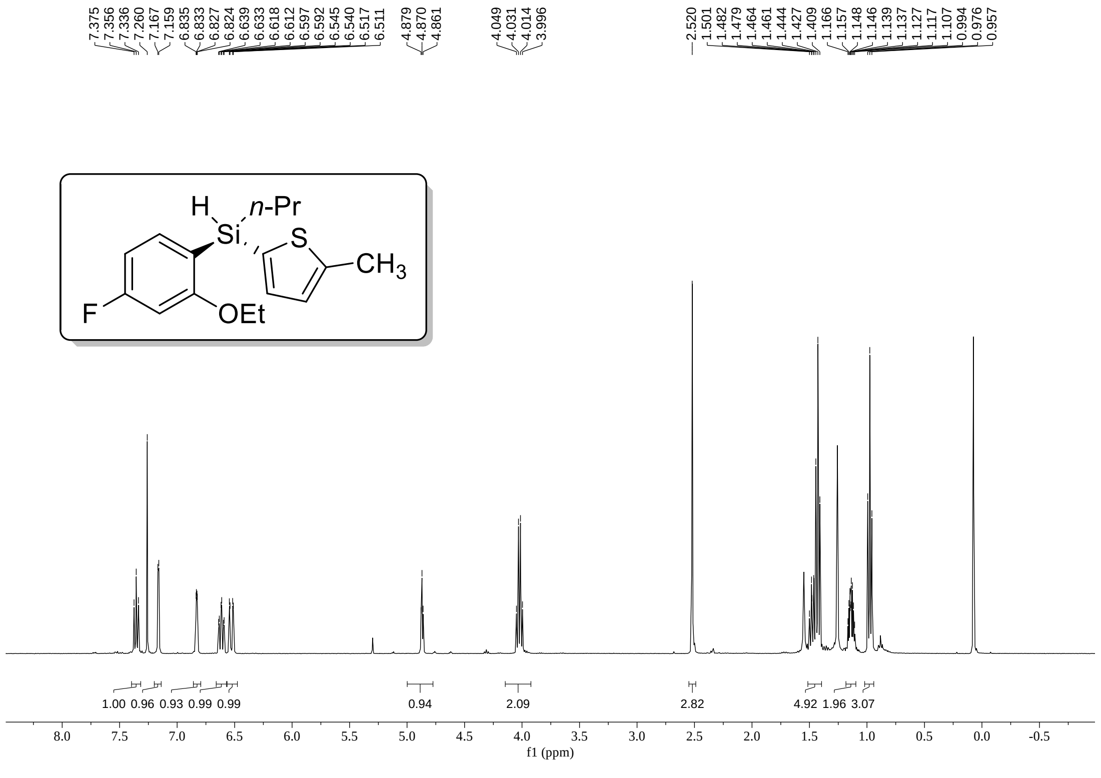

Supplementary Figure 243. ^{1}H NMR spectrum of **3na**

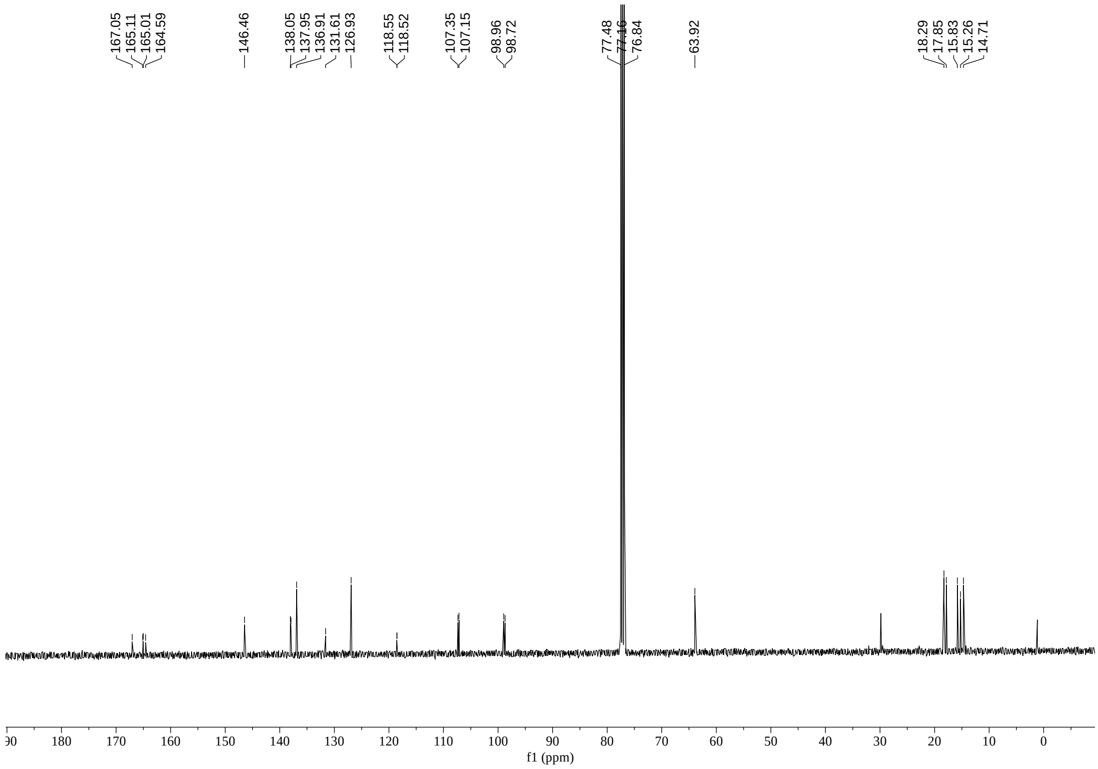

Supplementary Figure 244. ^{13}C NMR spectrum of **3na**

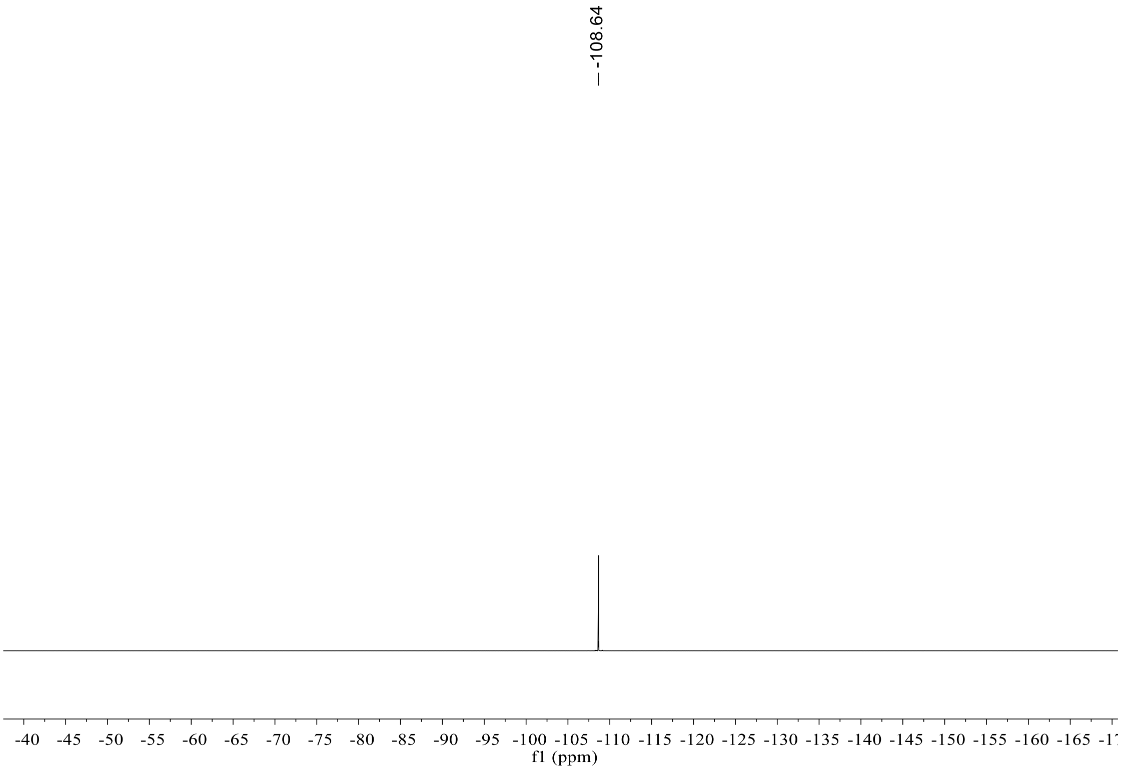

Supplementary Figure 245. ^{19}F NMR spectrum of **3na**

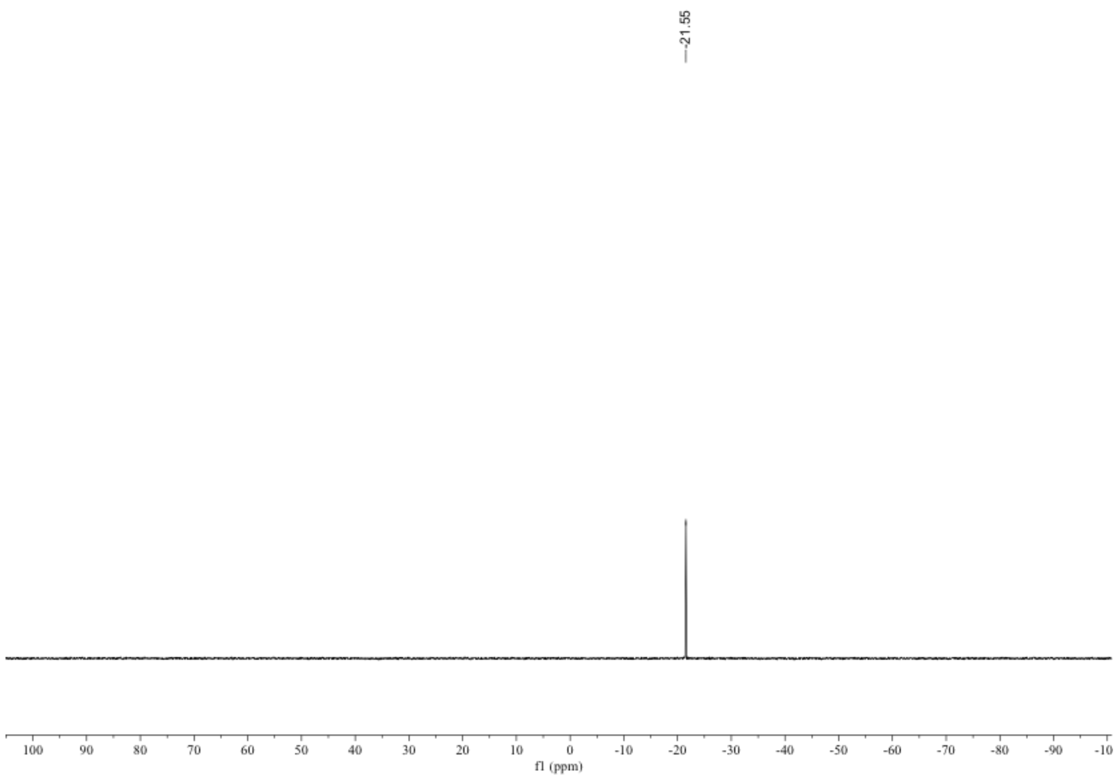

Supplementary Figure 246. ^{29}Si NMR spectrum of **3na**

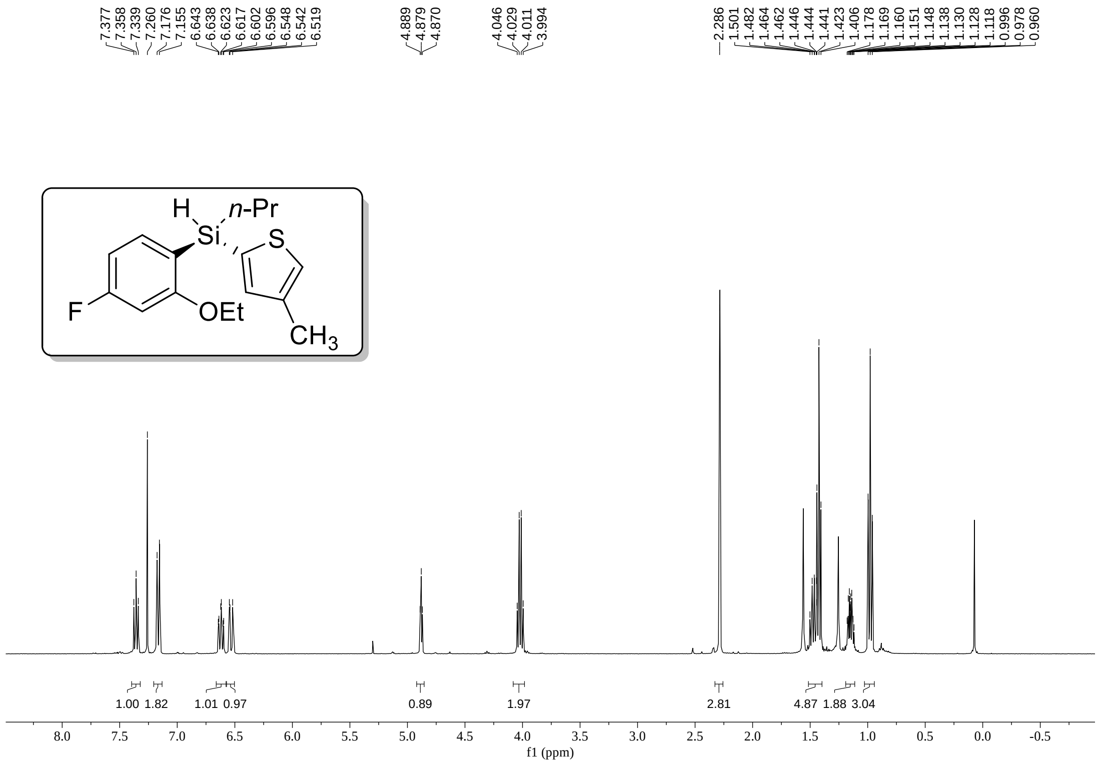

Supplementary Figure 247. ^{1}H NMR spectrum of **3ni**

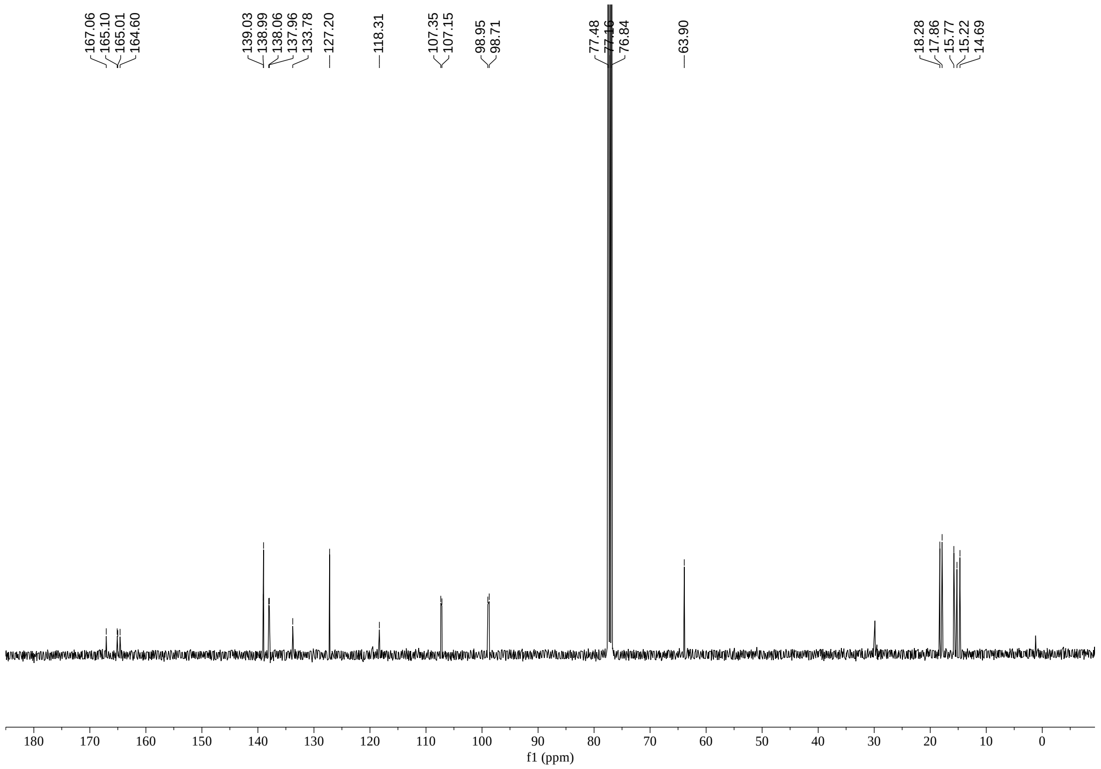

Supplementary Figure 248. ^{13}C NMR spectrum of **3ni**

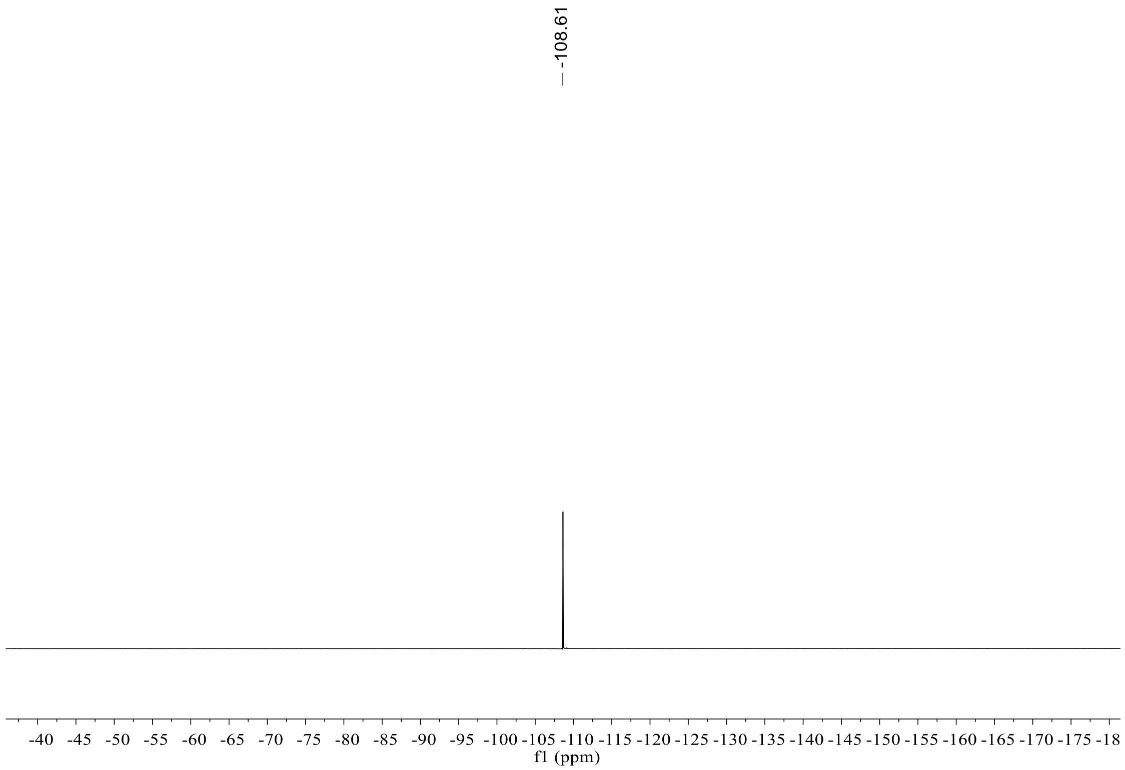

Supplementary Figure 249. ^{19}F NMR spectrum of **3ni**

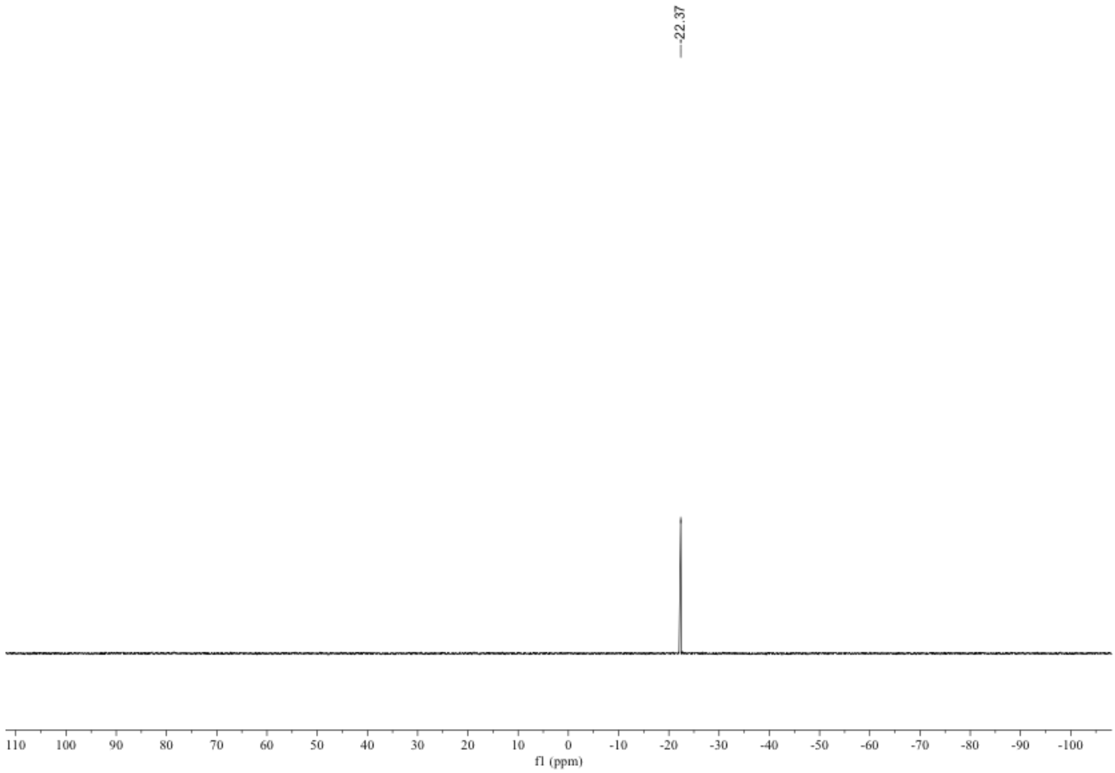

Supplementary Figure 250. ^{29}Si NMR spectrum of **3ni**

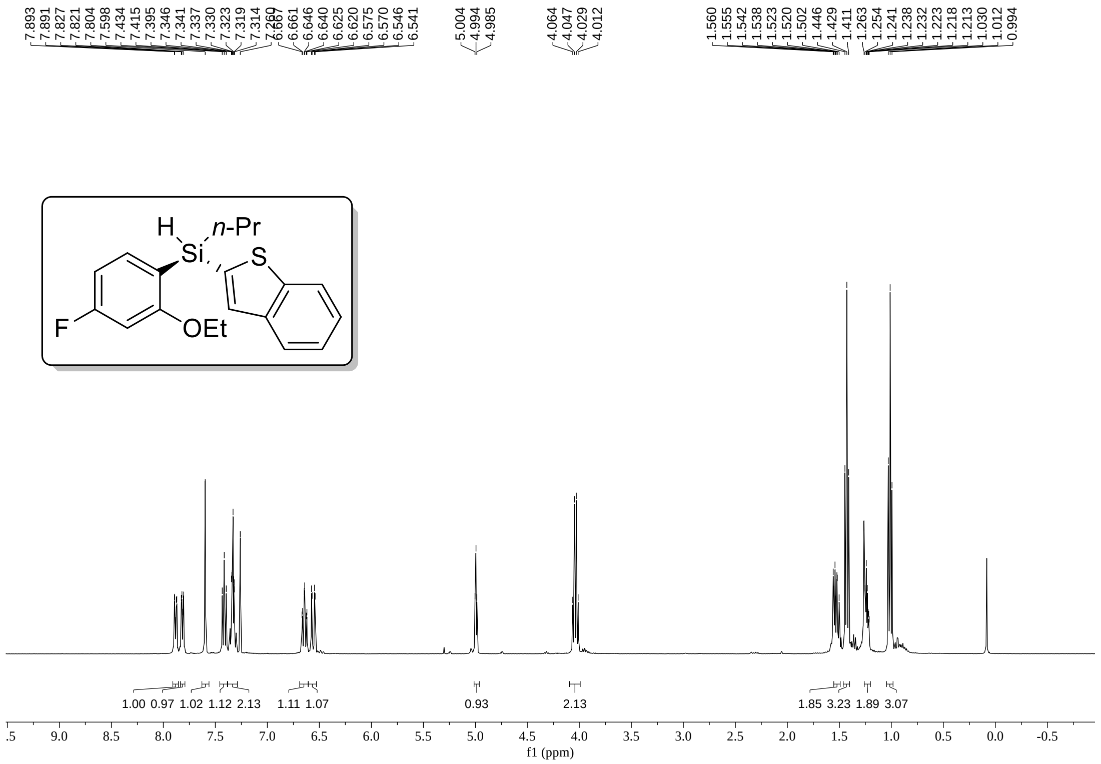

Supplementary Figure 251. ^{1}H NMR spectrum of **3nn**

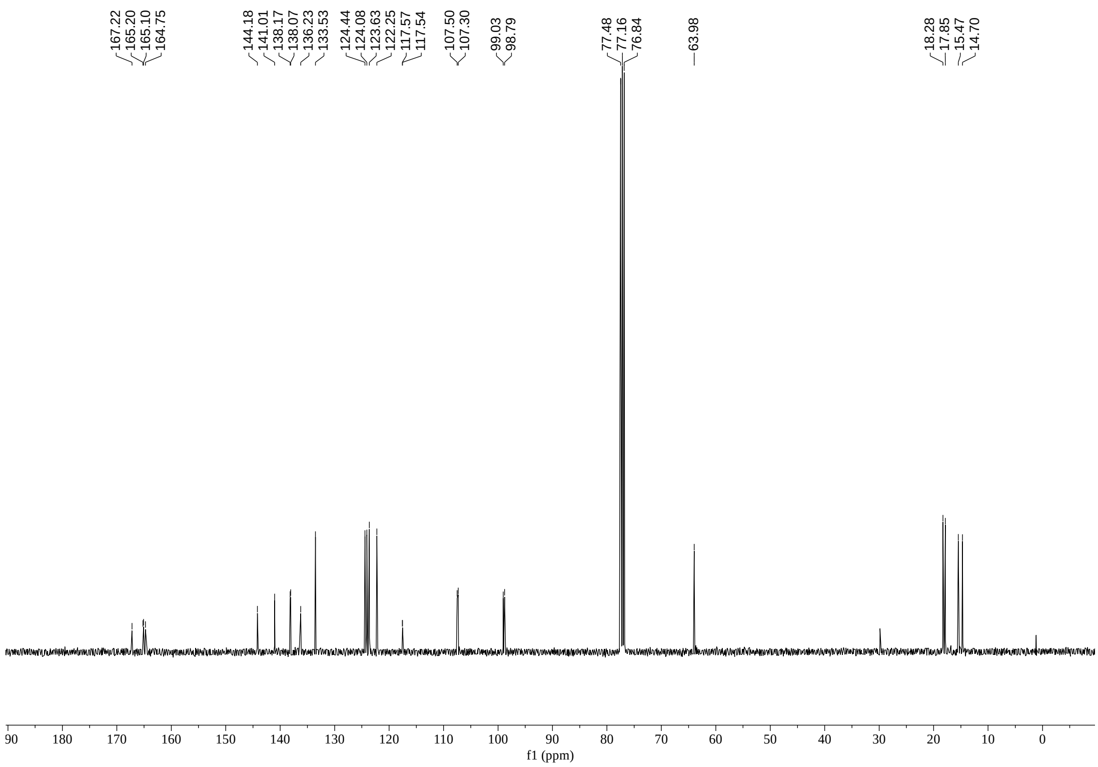

Supplementary Figure 252. ^{13}C NMR spectrum of **3nn**

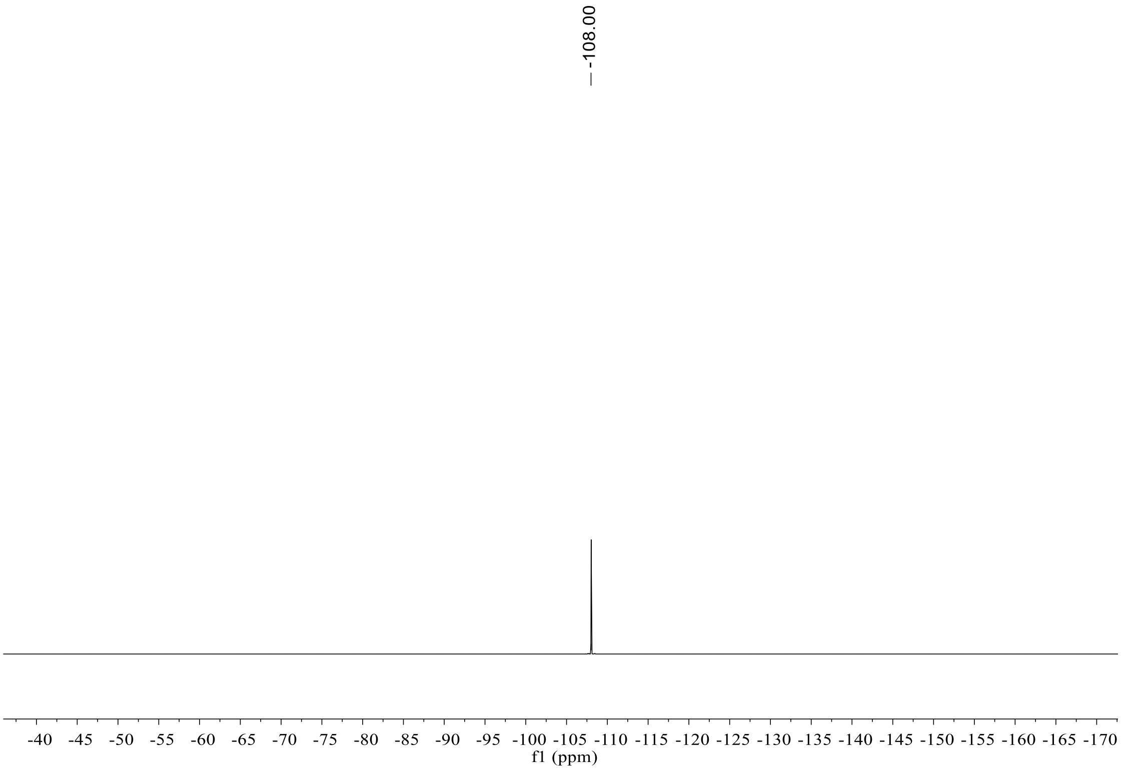

Supplementary Figure 253. ^{19}F NMR spectrum of **3nn**

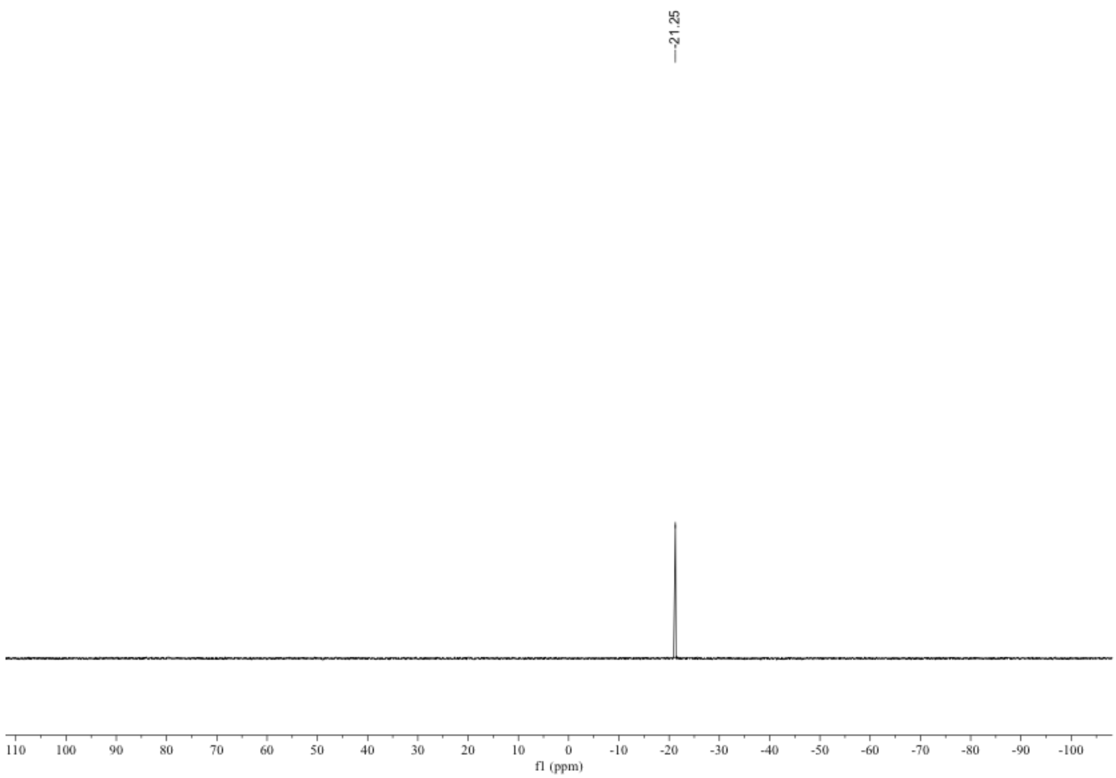

Supplementary Figure 254. ^{29}Si NMR spectrum of **3nn**

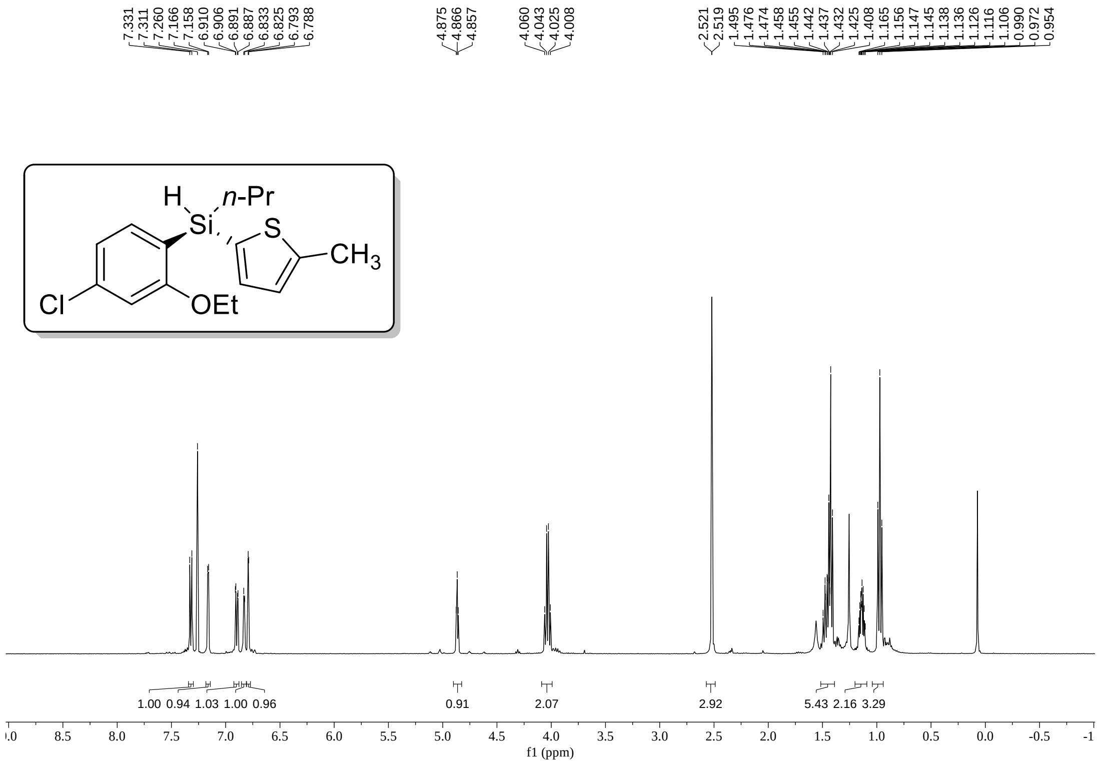

Supplementary Figure 255. ^{1}H NMR spectrum of **3oa**

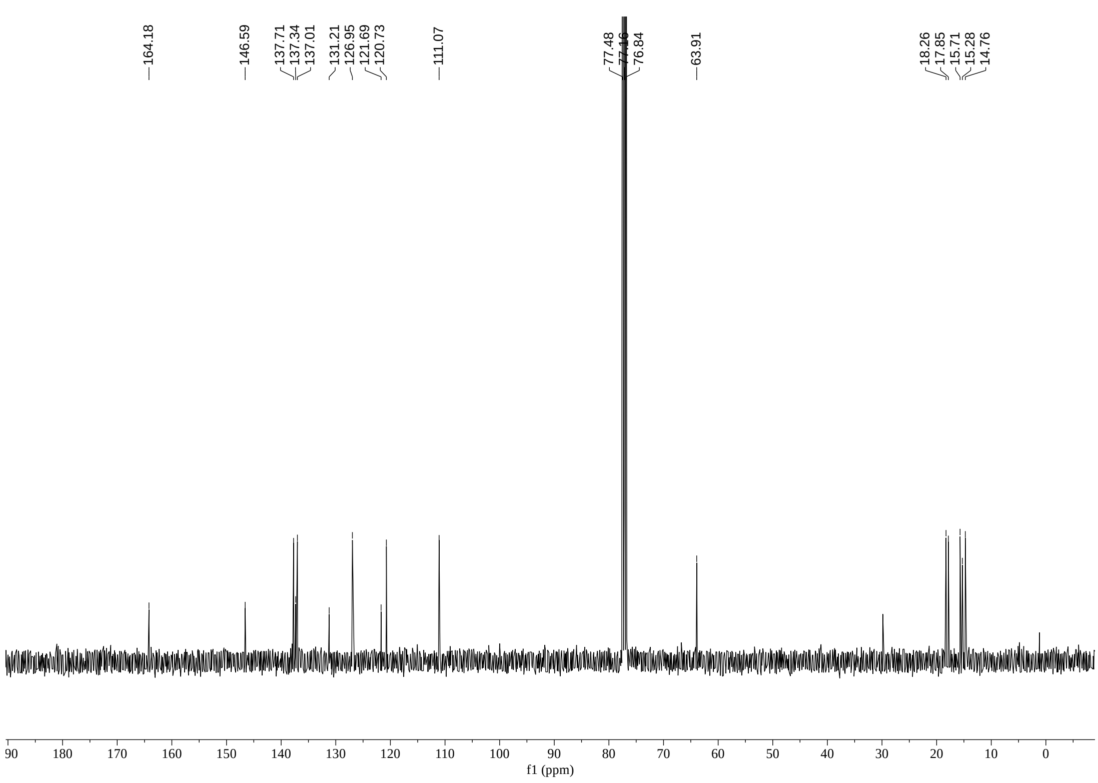

Supplementary Figure 256. ^{13}C NMR spectrum of **3oa**

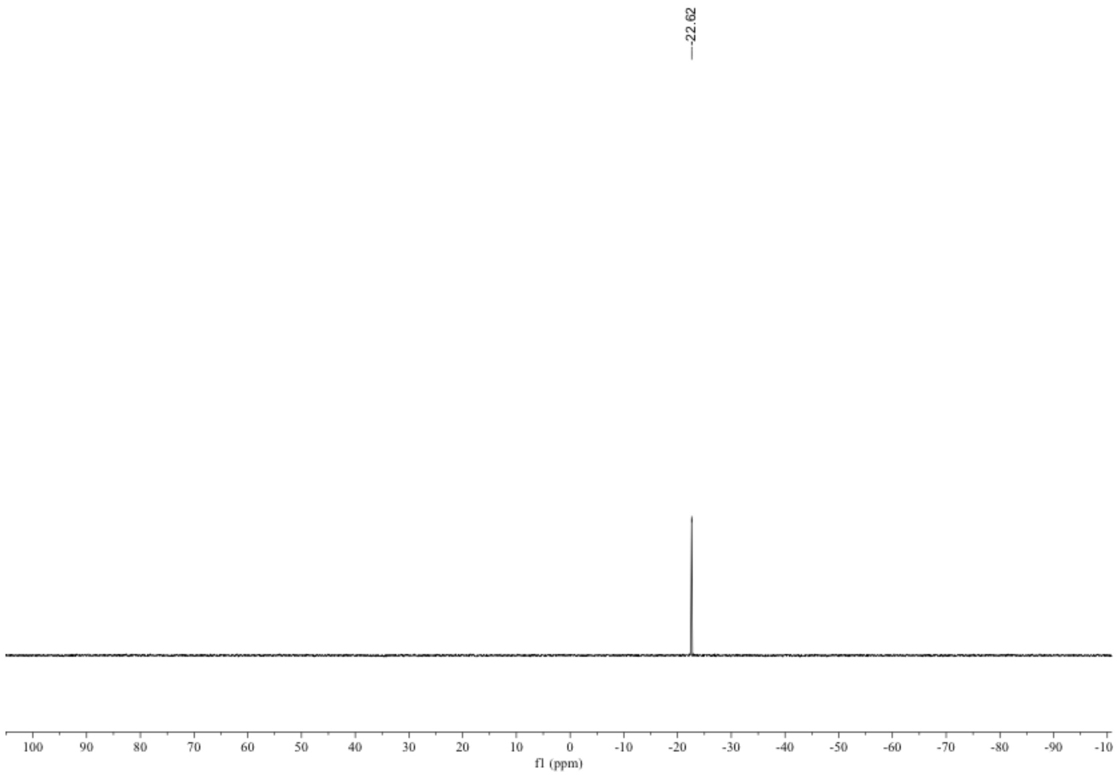

Supplementary Figure 257. ^{29}Si NMR spectrum of **3oa**

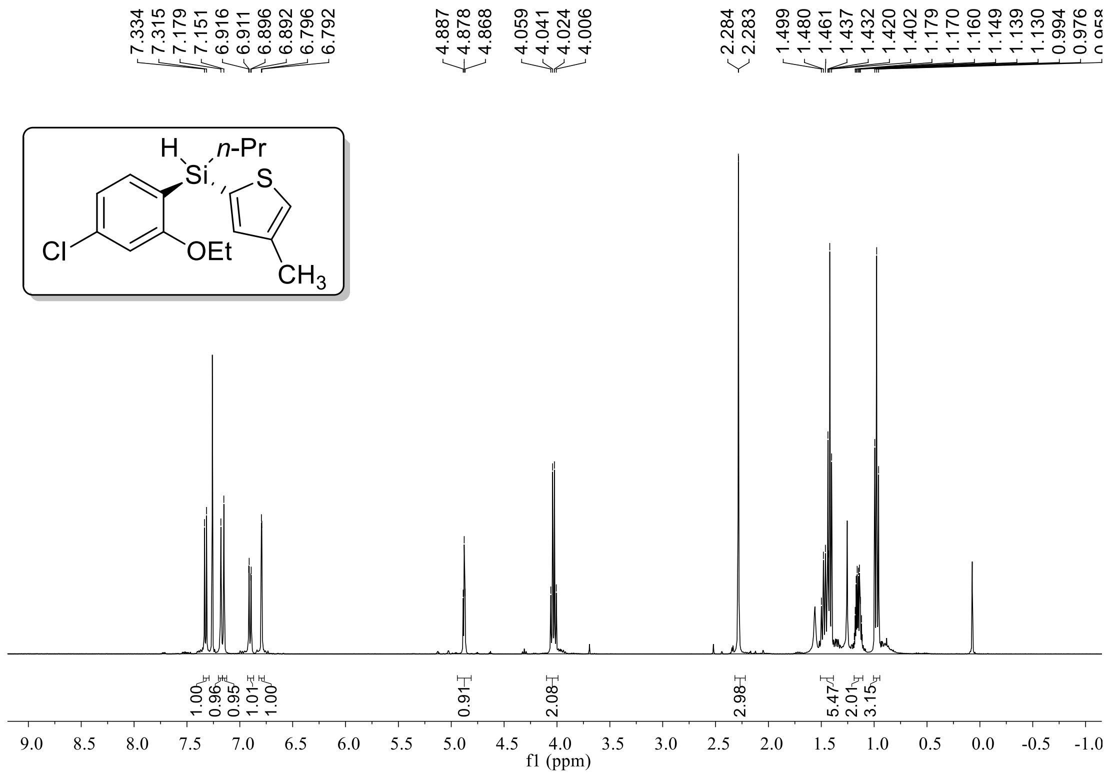

Supplementary Figure 258. ^{1}H NMR spectrum of **3oi**

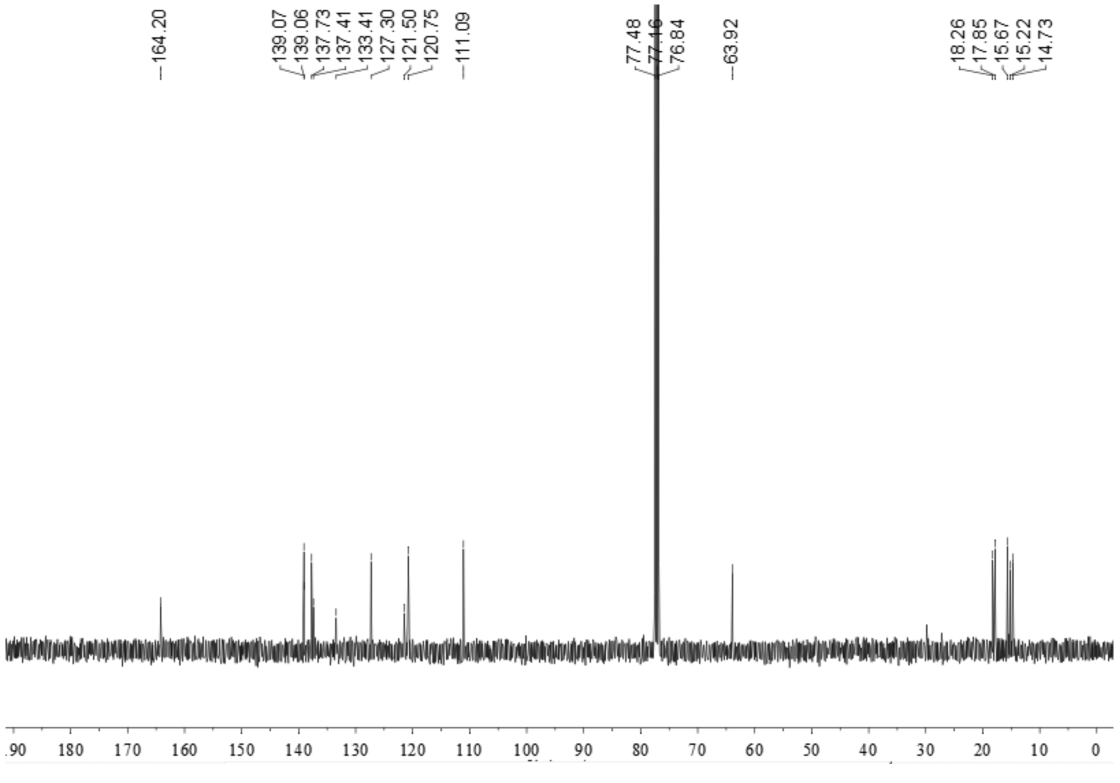

Supplementary Figure 259. ^{13}C NMR spectrum of **3oi**

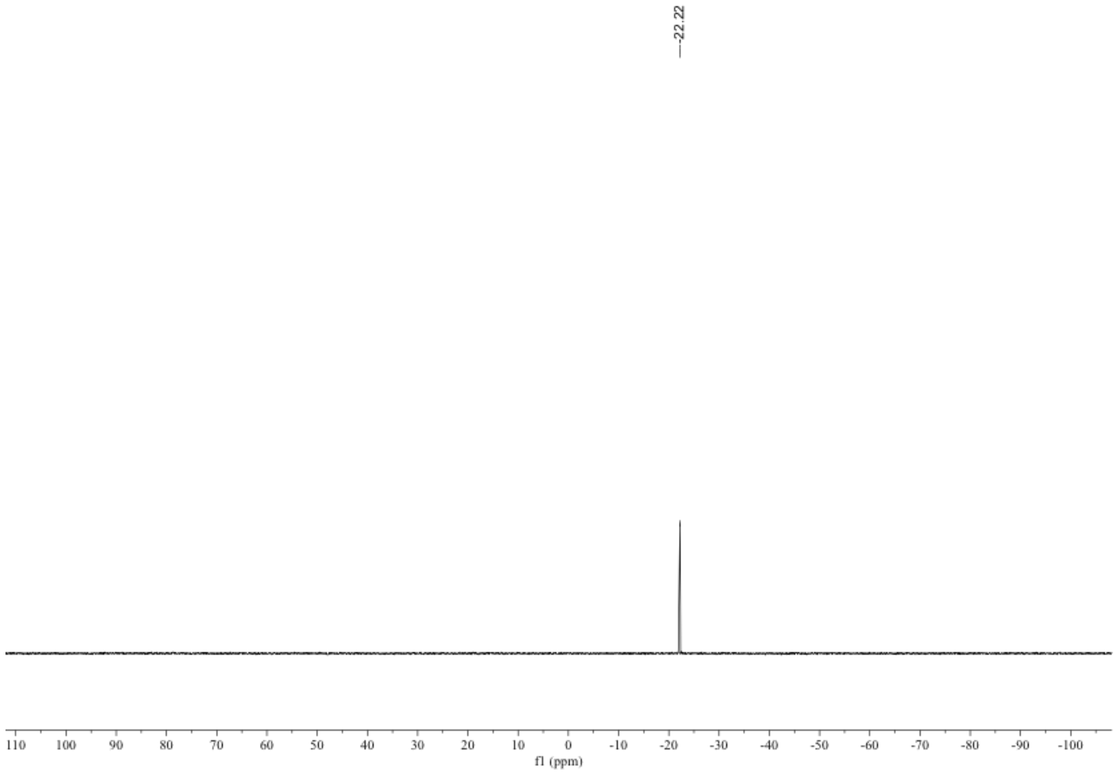

Supplementary Figure 260. ^{29}Si NMR spectrum of **3oi**

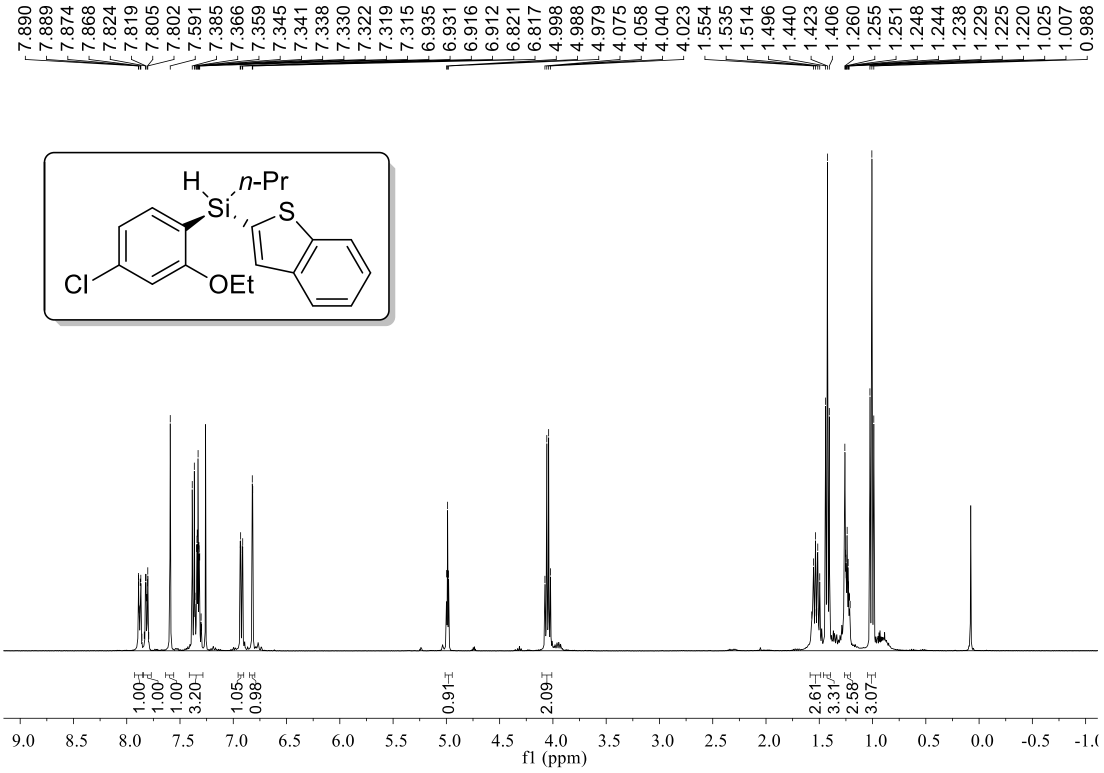

Supplementary Figure 261. ^{1}H NMR spectrum of **3on**

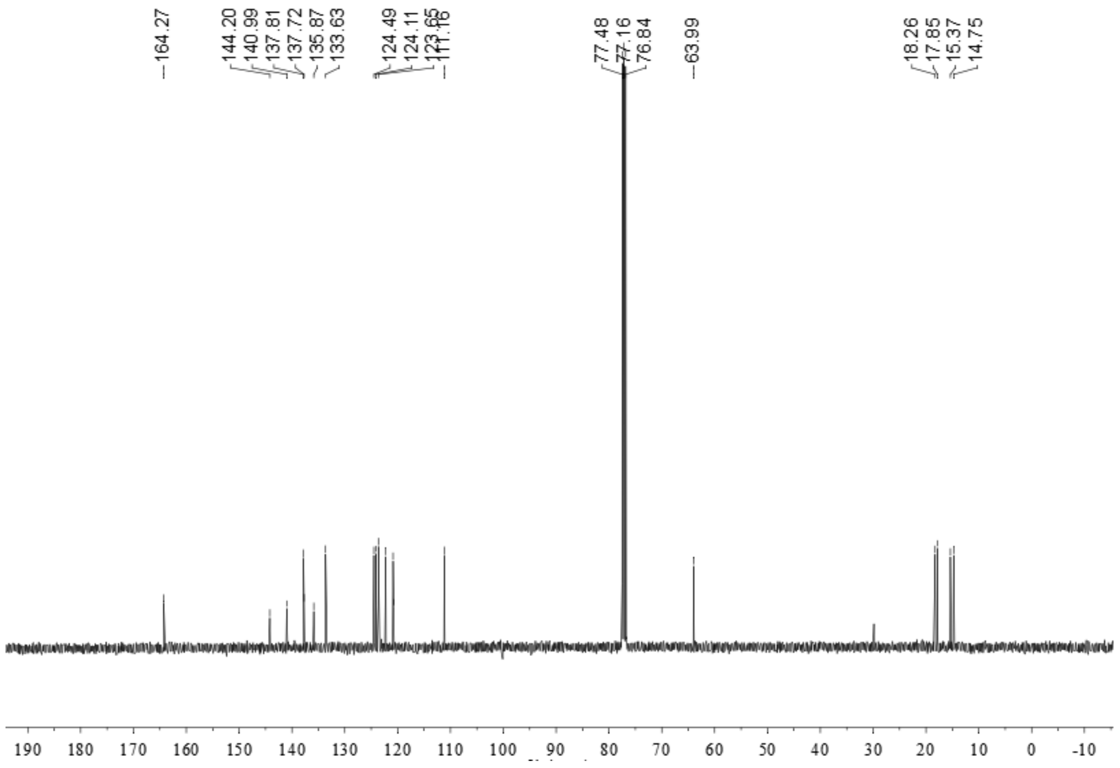

Supplementary Figure 262. ^{13}C NMR spectrum of **3on**

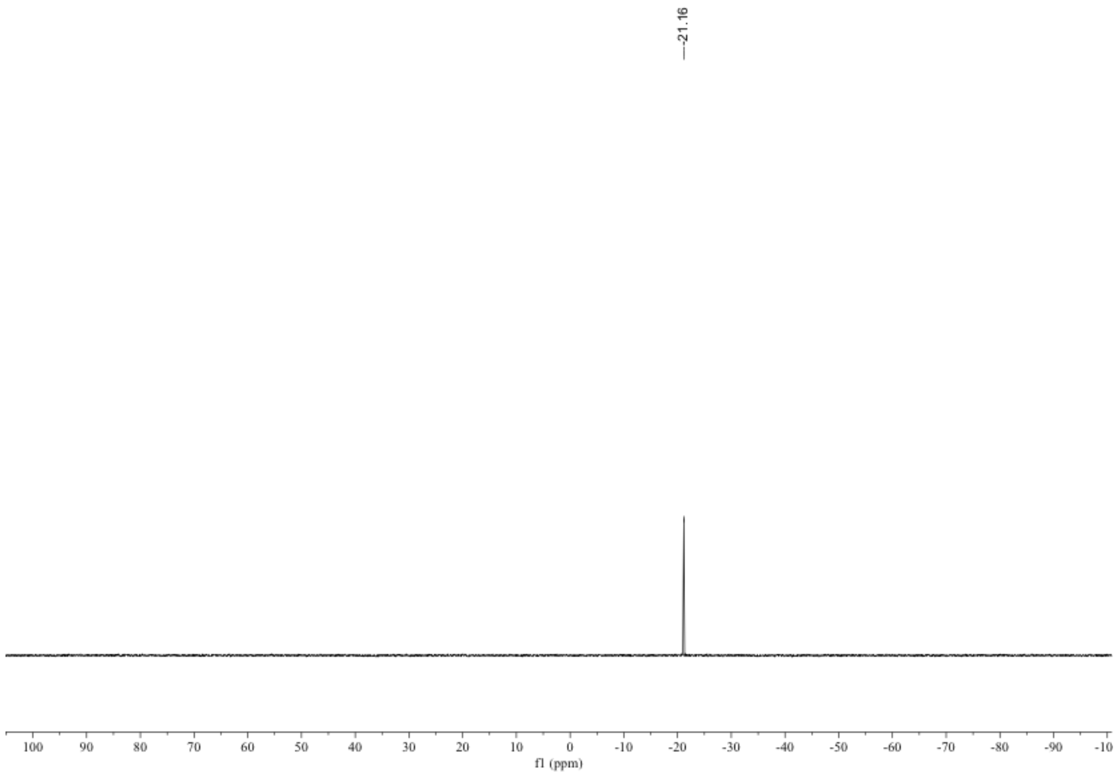

Supplementary Figure 263. ^{29}Si NMR spectrum of **3on**

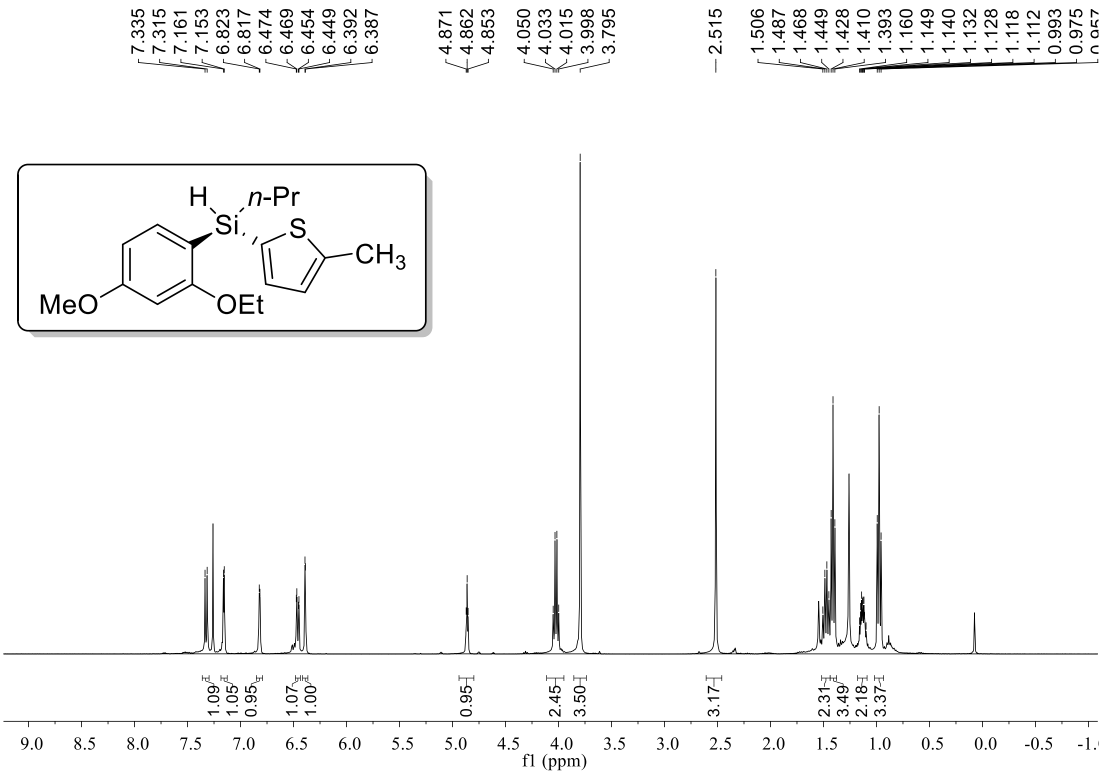

Supplementary Figure 264. ^{1}H NMR spectrum of **3pa**

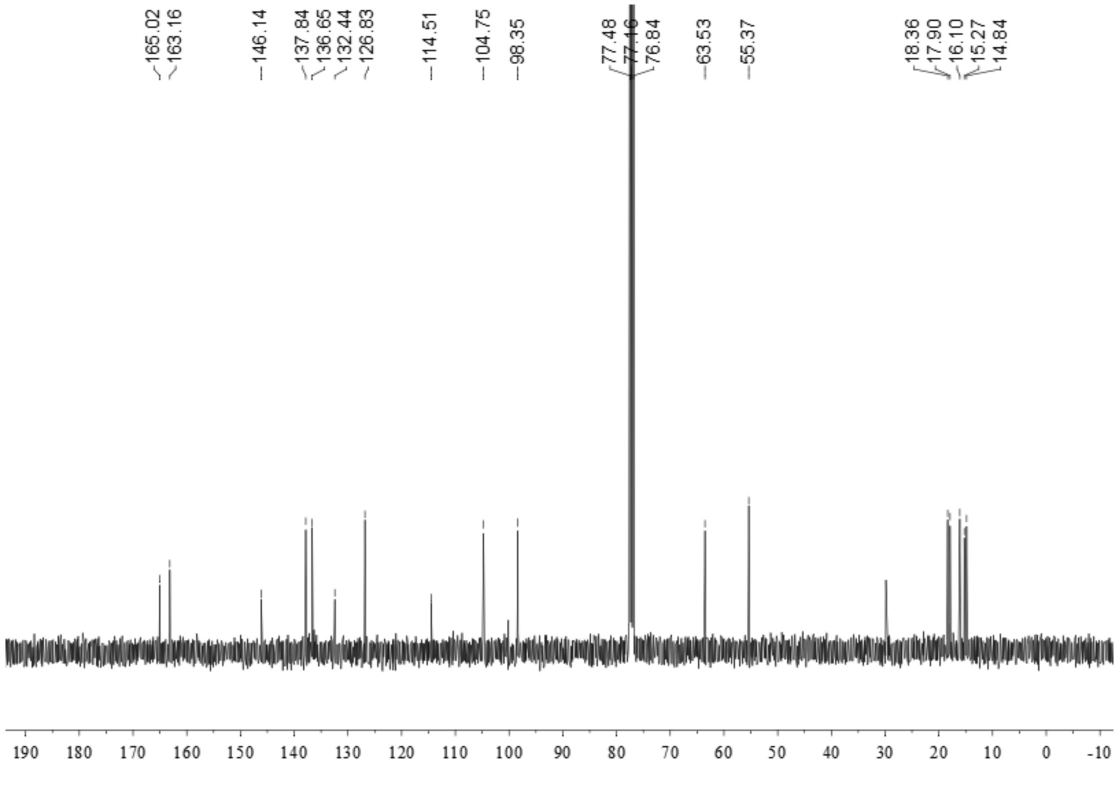

Supplementary Figure 265. ^{13}C NMR spectrum of **3pa**

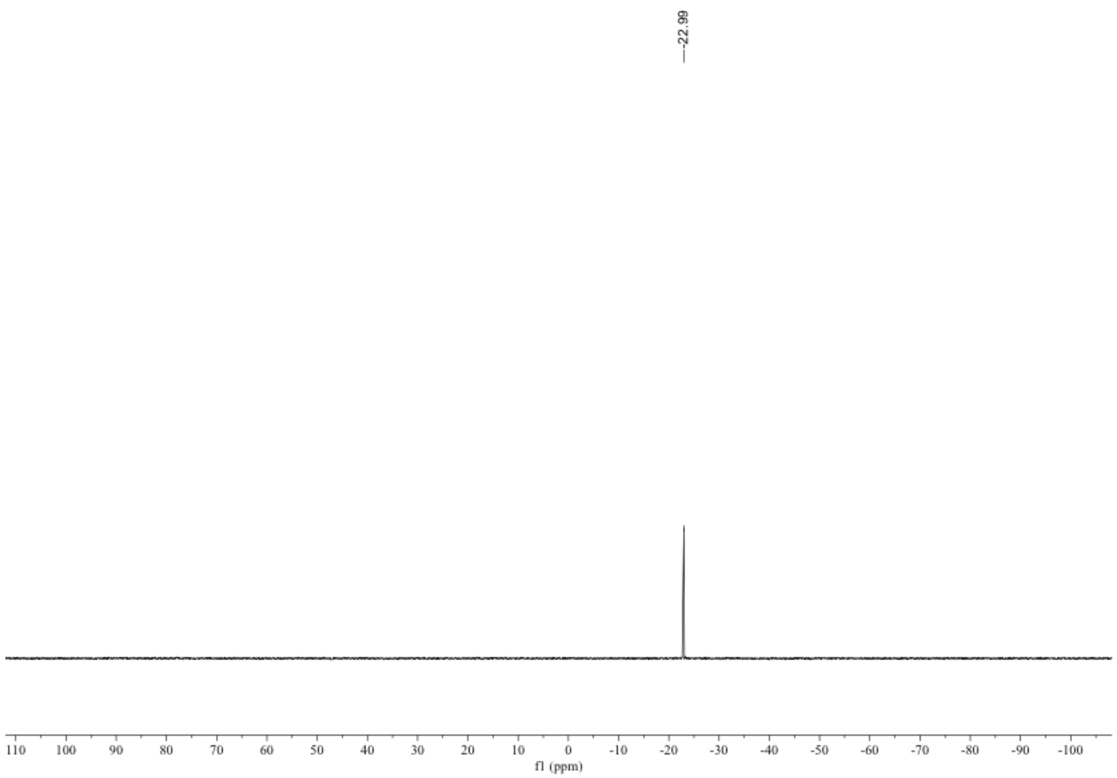

Supplementary Figure 266. ^{29}Si NMR spectrum of **3pa**

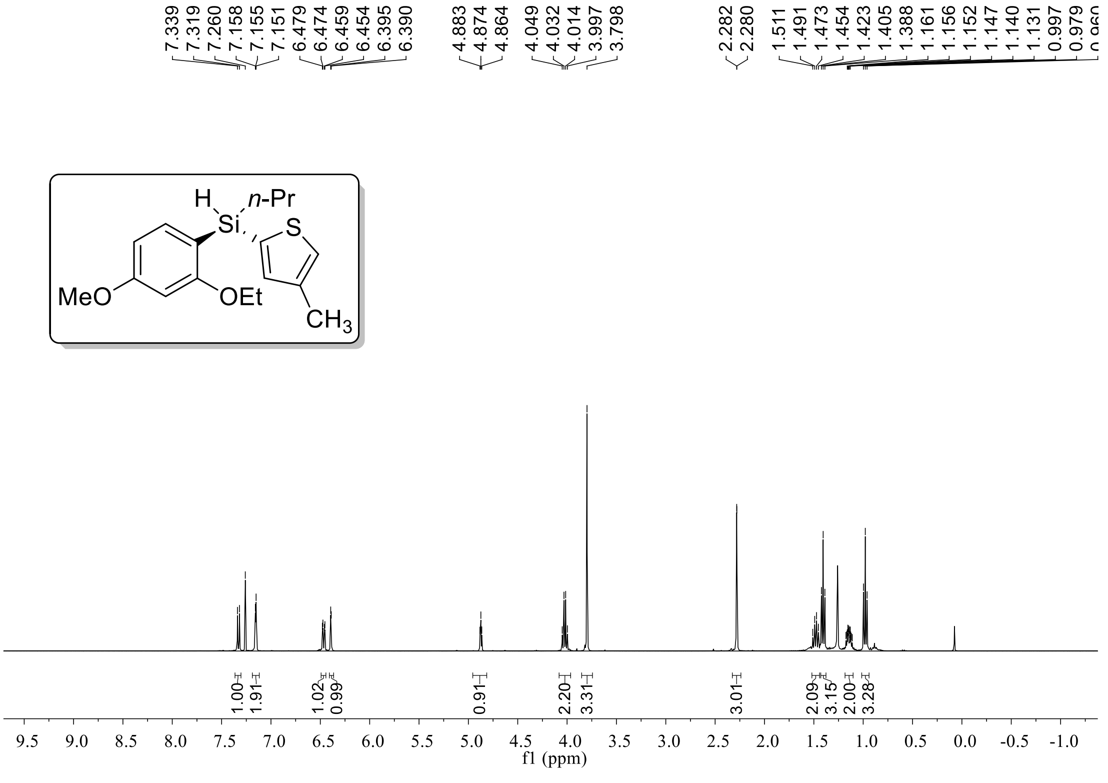

Supplementary Figure 267. ^{1}H NMR spectrum of **3pi**

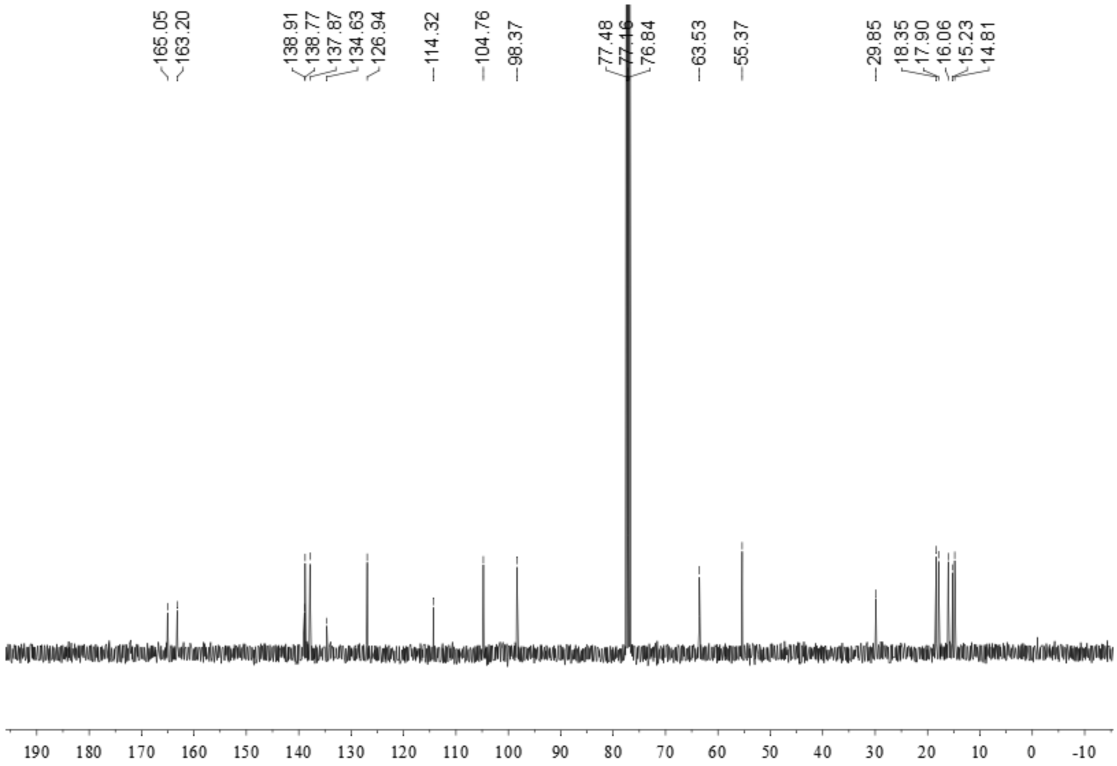

Supplementary Figure 268. ^{13}C NMR spectrum of **3pi**

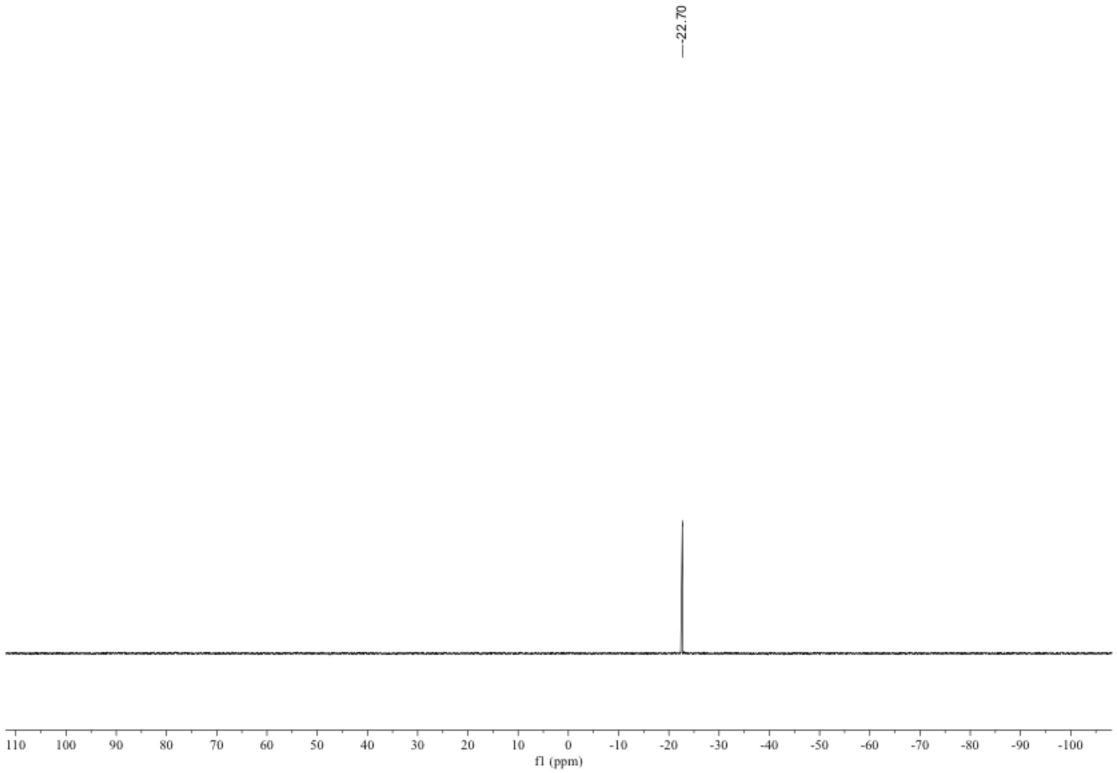

Supplementary Figure 269. ^{29}Si NMR spectrum of **3pi**

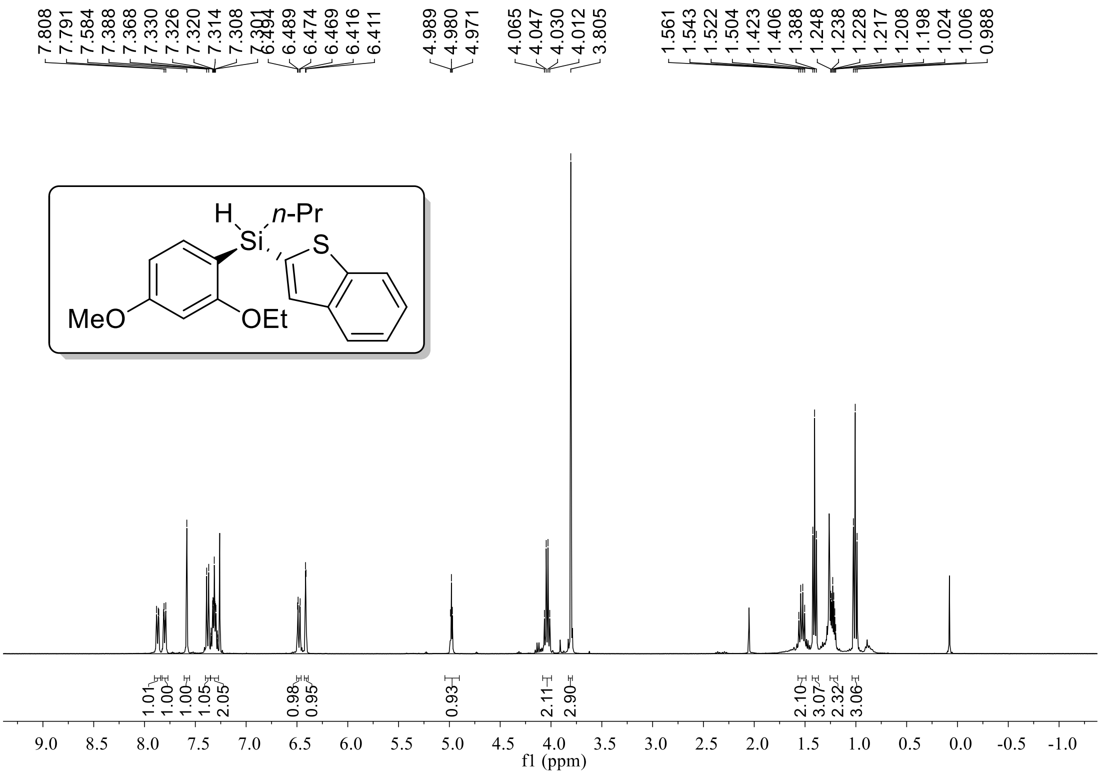

Supplementary Figure 270. ^{1}H NMR spectrum of **3pn**

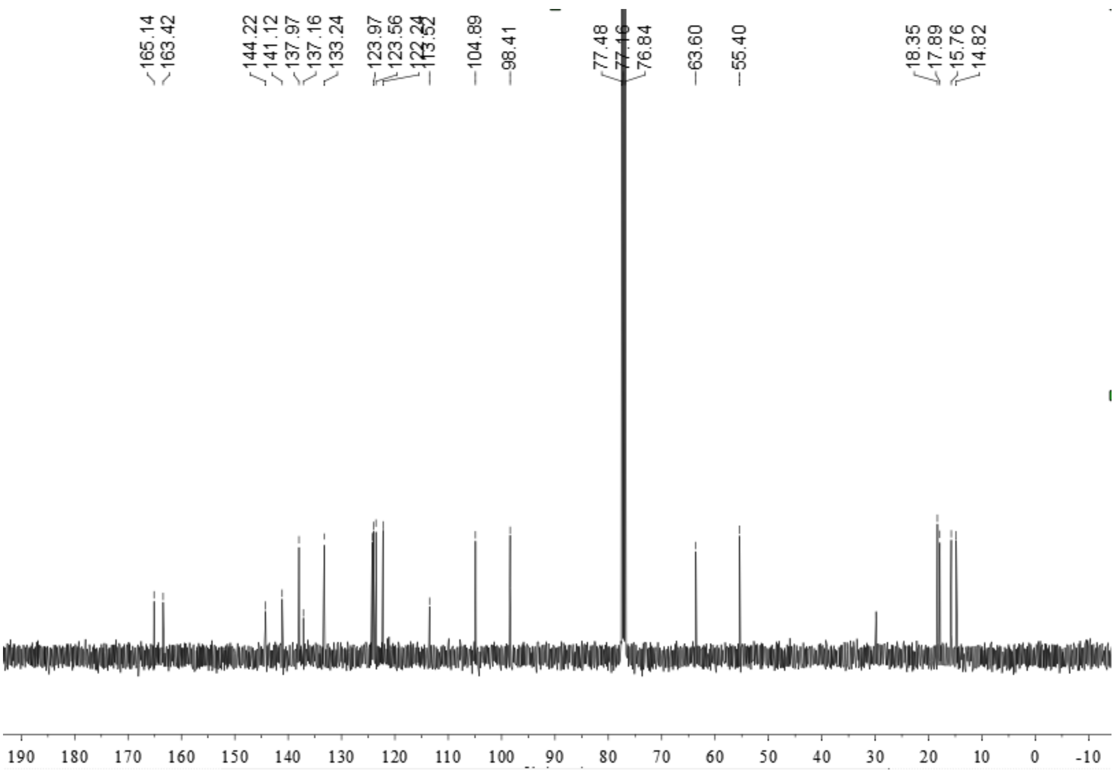

Supplementary Figure 271. ^{13}C NMR spectrum of **3pn**

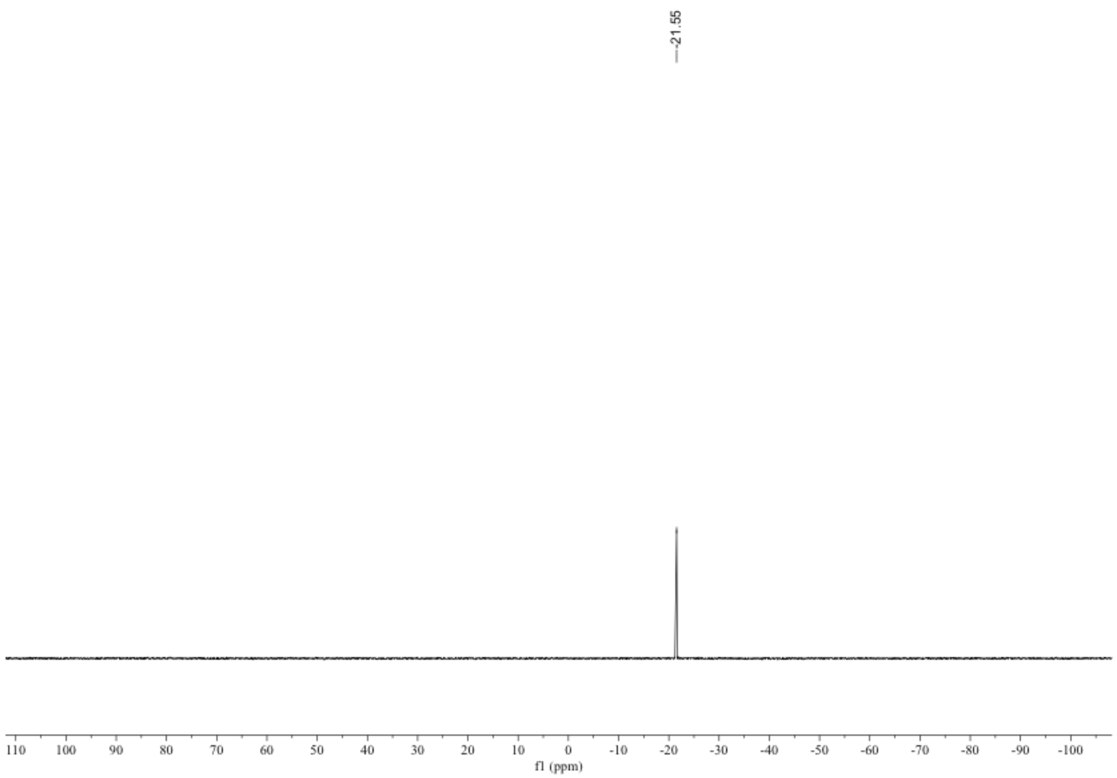

Supplementary Figure 272. ^{29}Si NMR spectrum of **3pn**

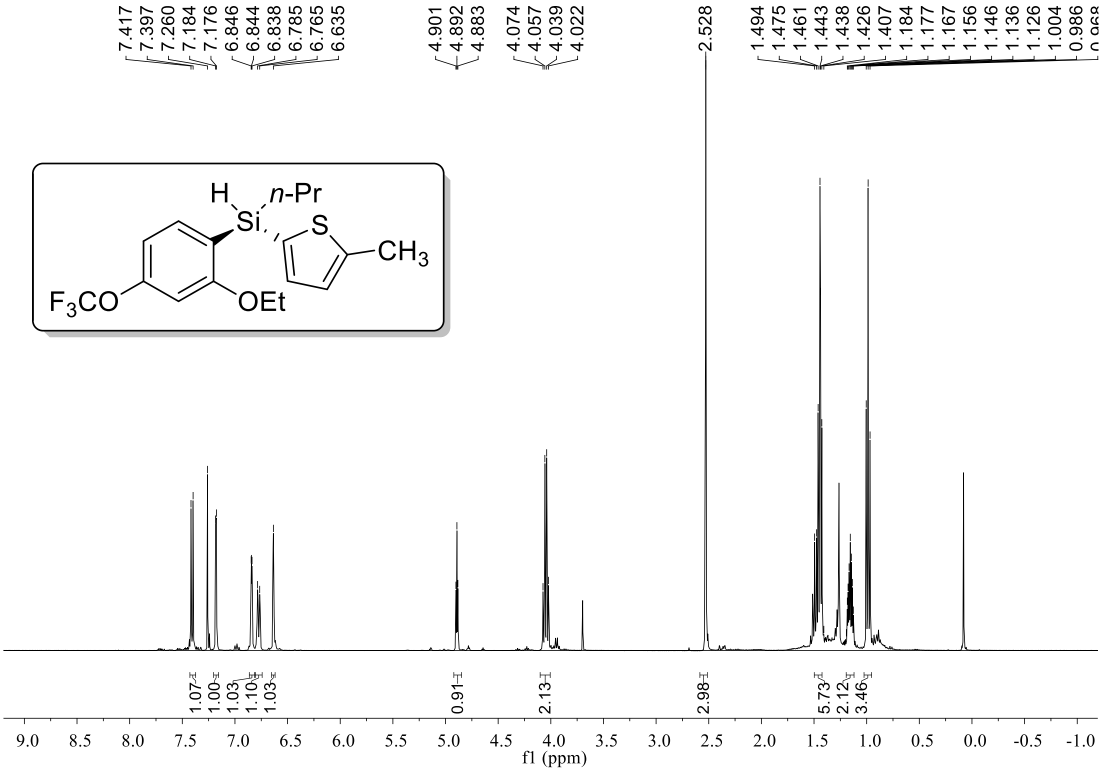

Supplementary Figure 273. ^{1}H NMR spectrum of **3qa**

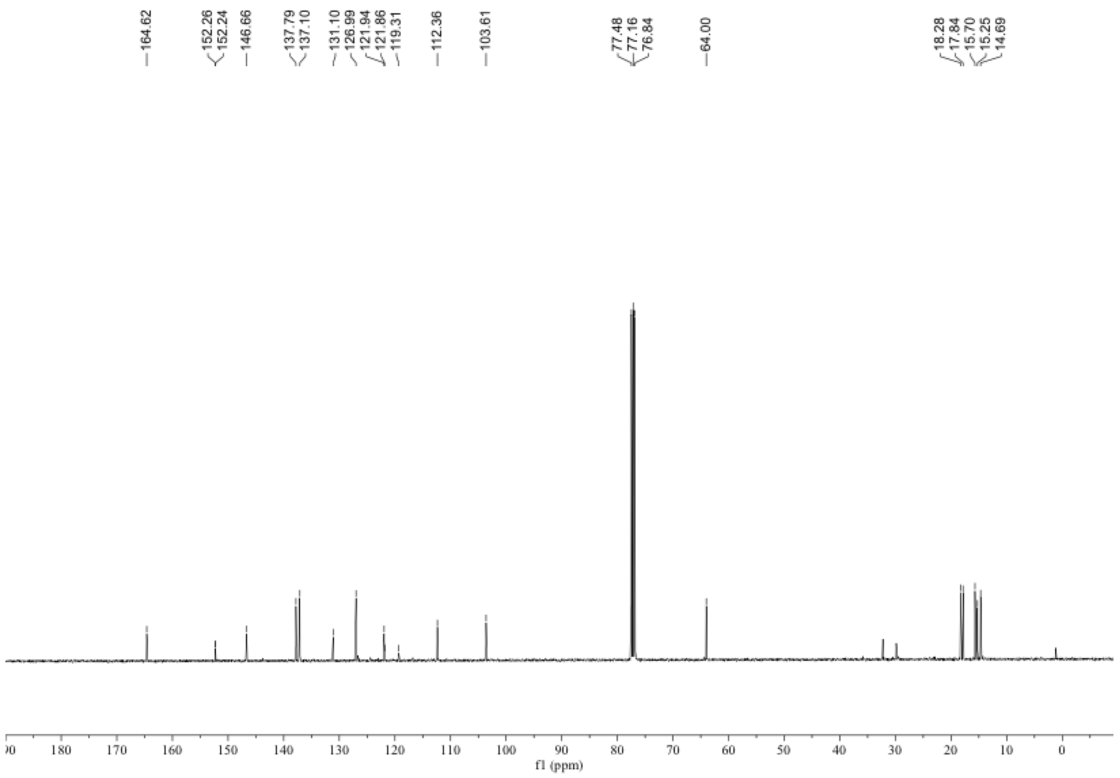

Supplementary Figure 274. ^{13}C NMR spectrum of **3qa**

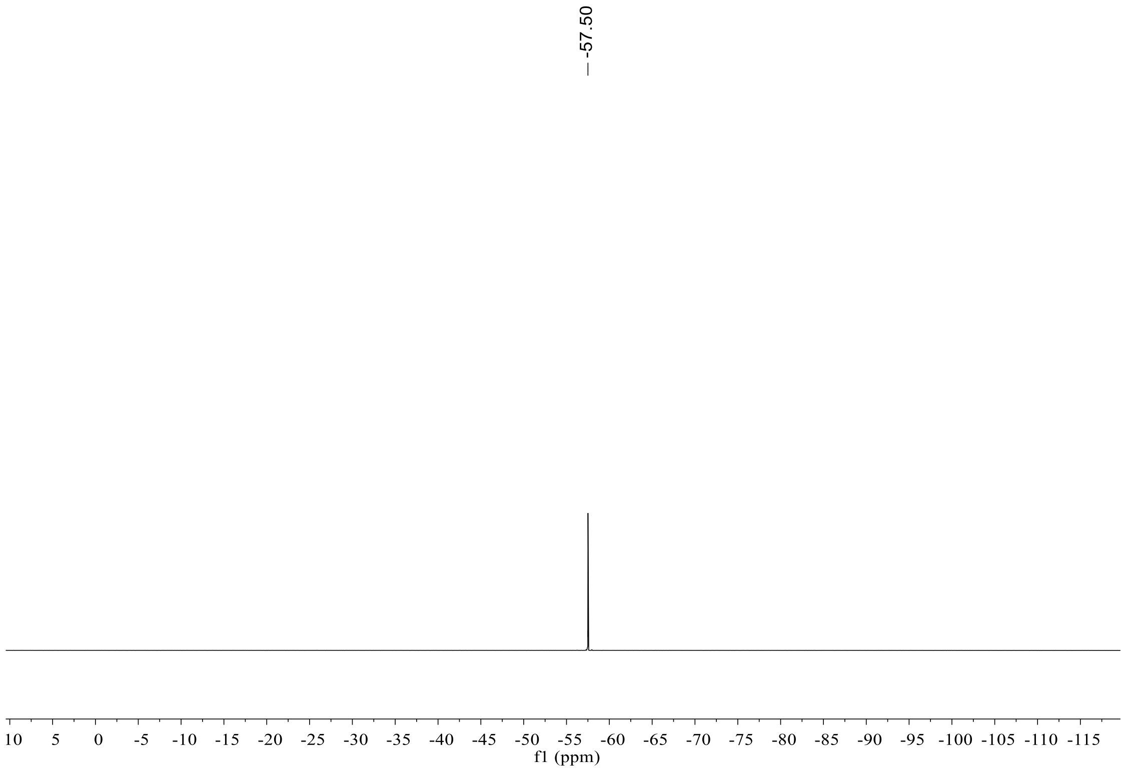

Supplementary Figure 275. ^{19}F NMR spectrum of **3qa**

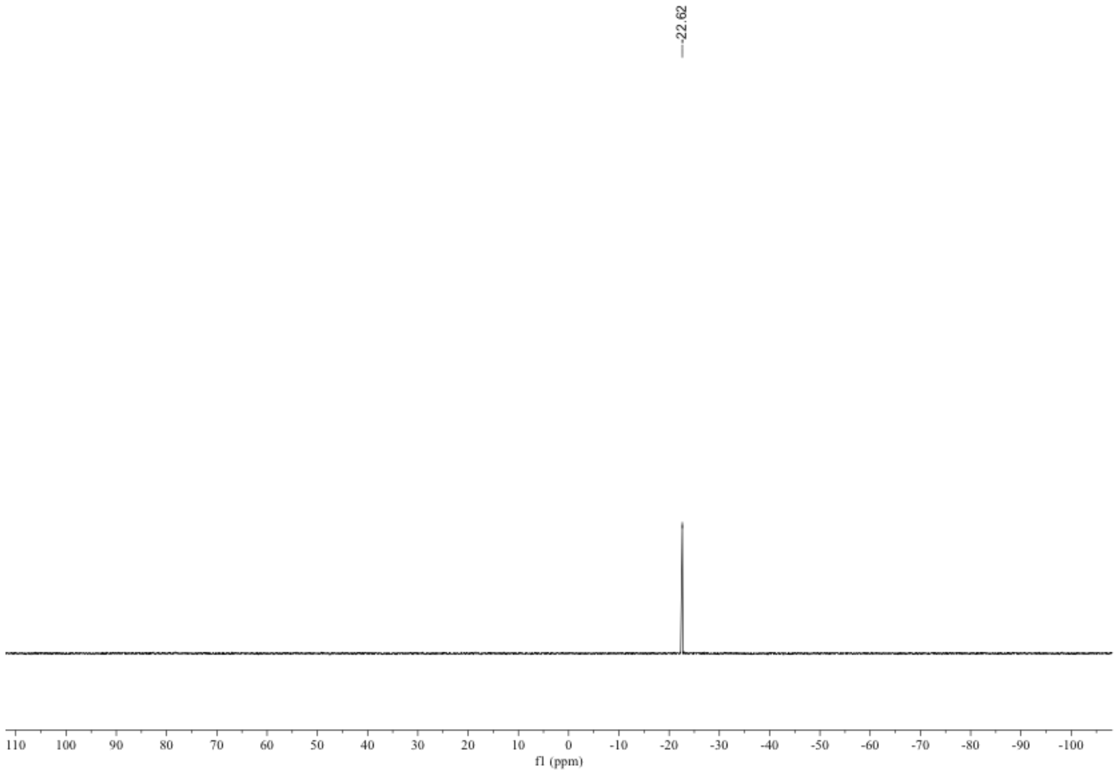

Supplementary Figure 276. ^{29}Si NMR spectrum of **3qa**

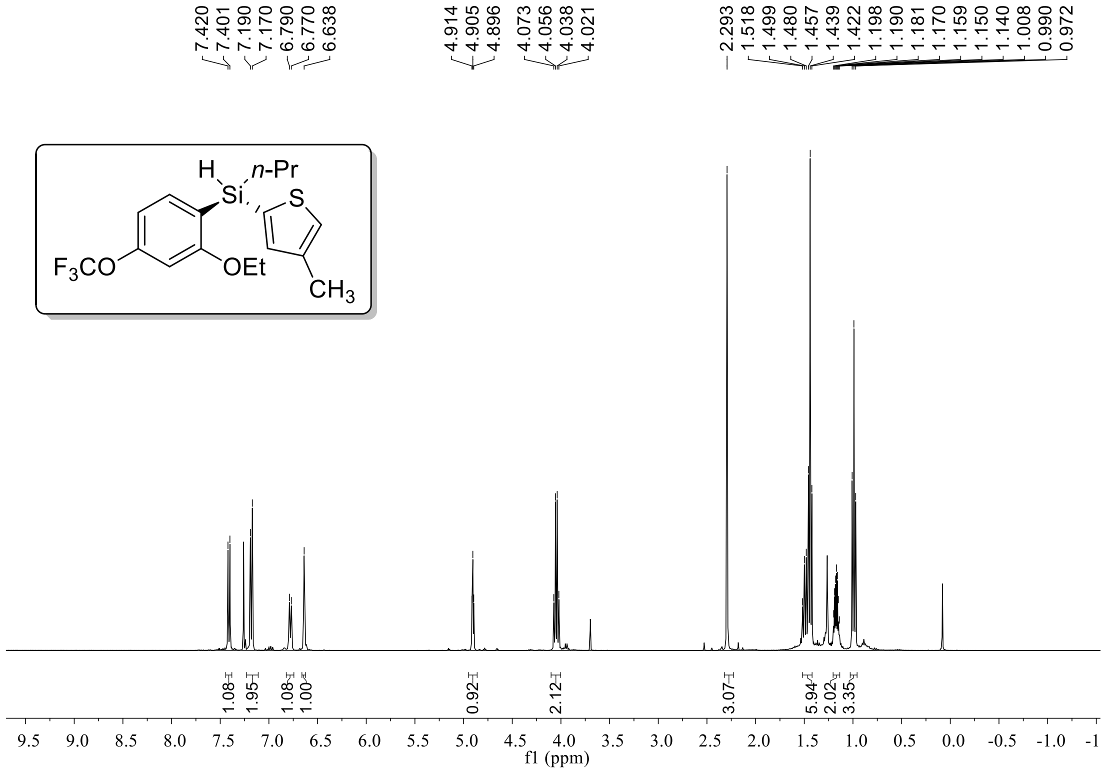

Supplementary Figure 277. ^{1}H NMR spectrum of **3qi**

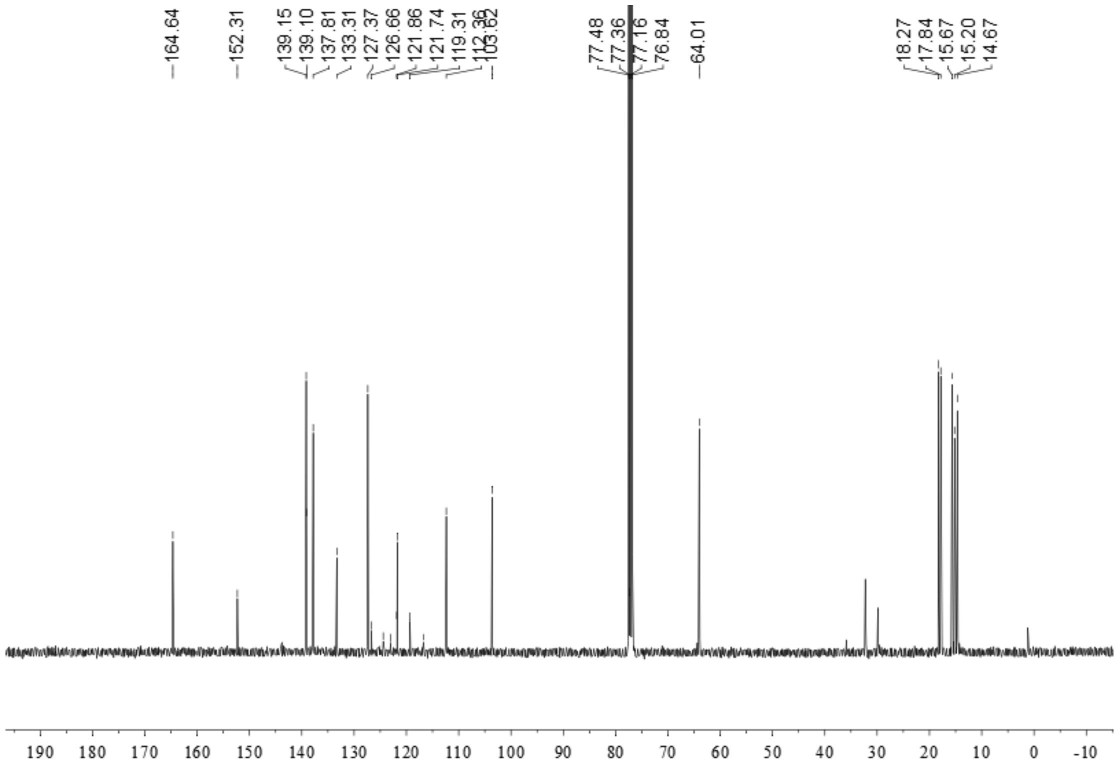

Supplementary Figure 278. ^{13}C NMR spectrum of **3qi**

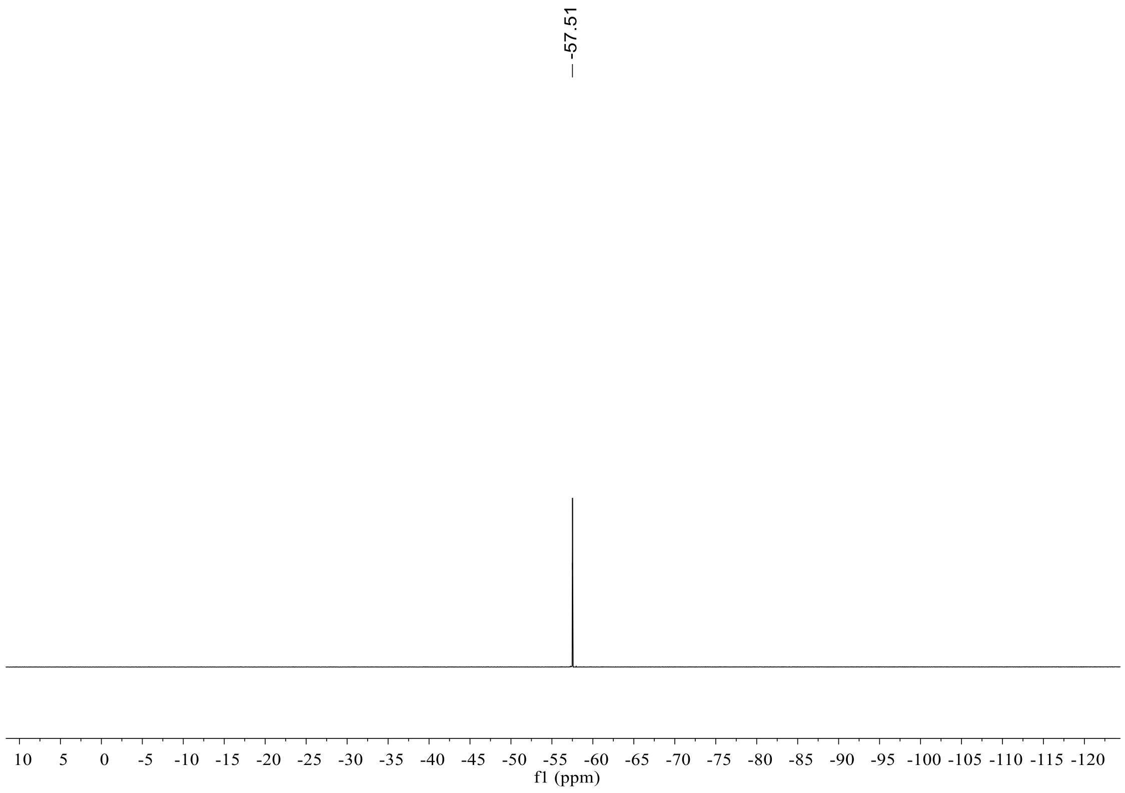

Supplementary Figure 279. ^{19}F NMR spectrum of **3qi**

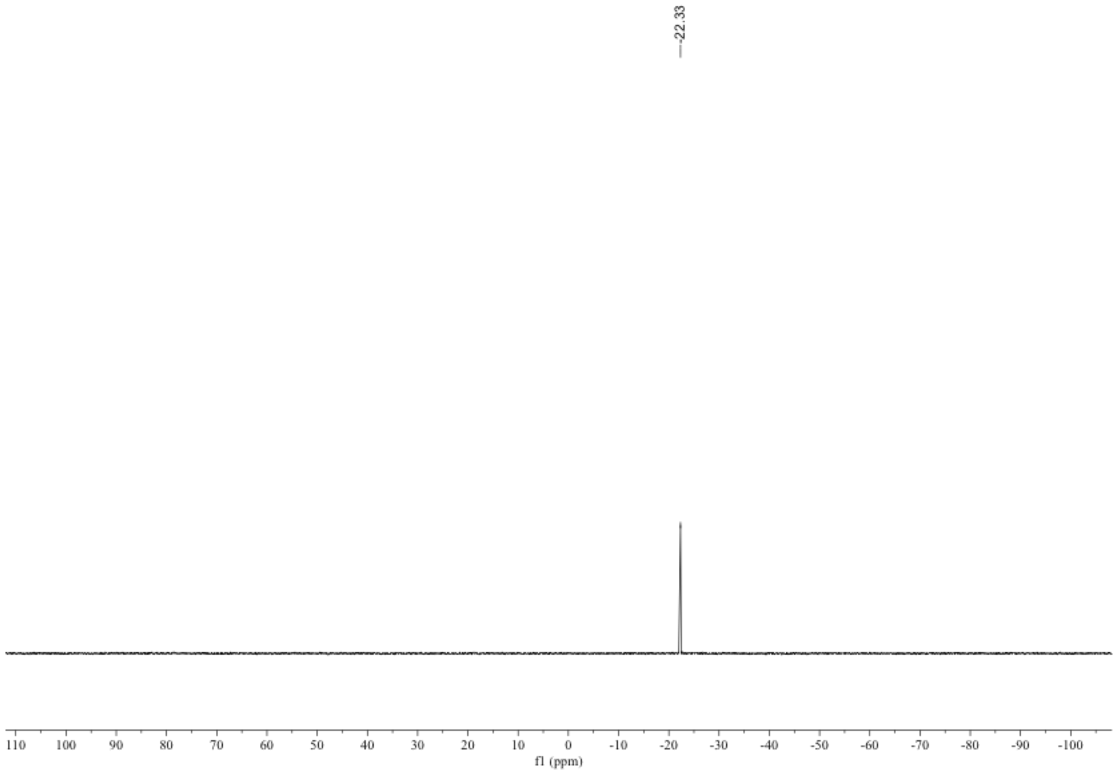

Supplementary Figure 280. ^{29}Si NMR spectrum of **3qi**

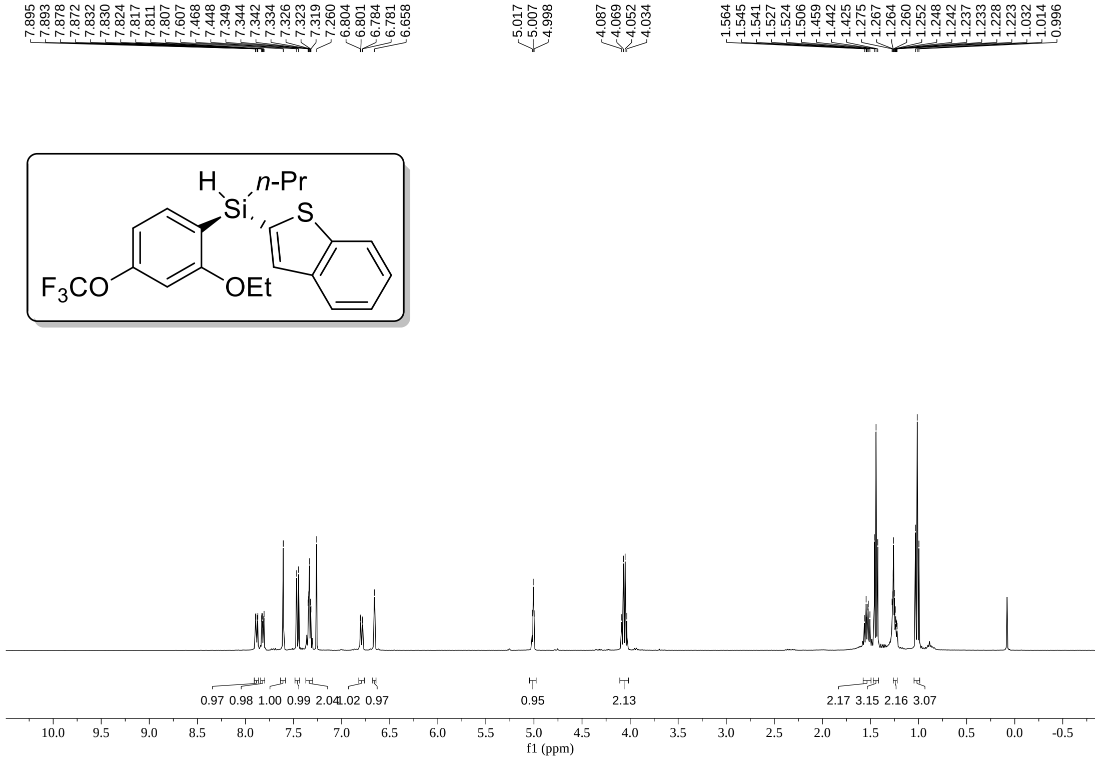

Supplementary Figure 281. ^{1}H NMR spectrum of **3qn**

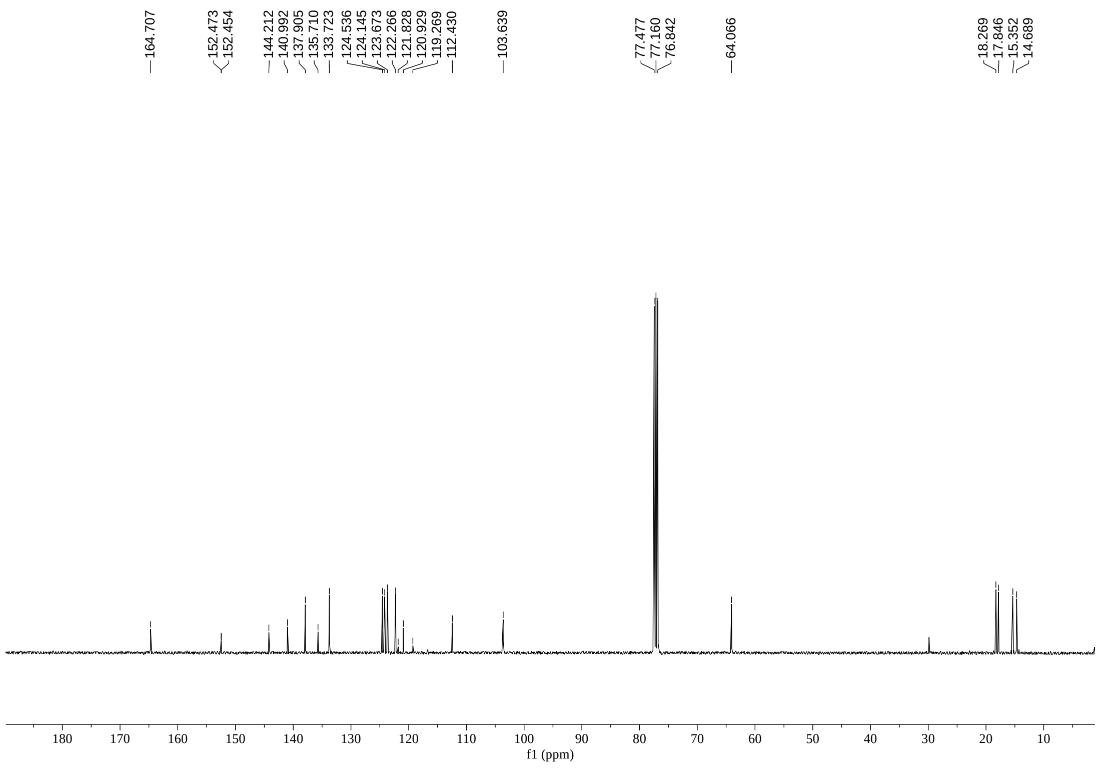

Supplementary Figure 282. ^{13}C NMR spectrum of **3qn**

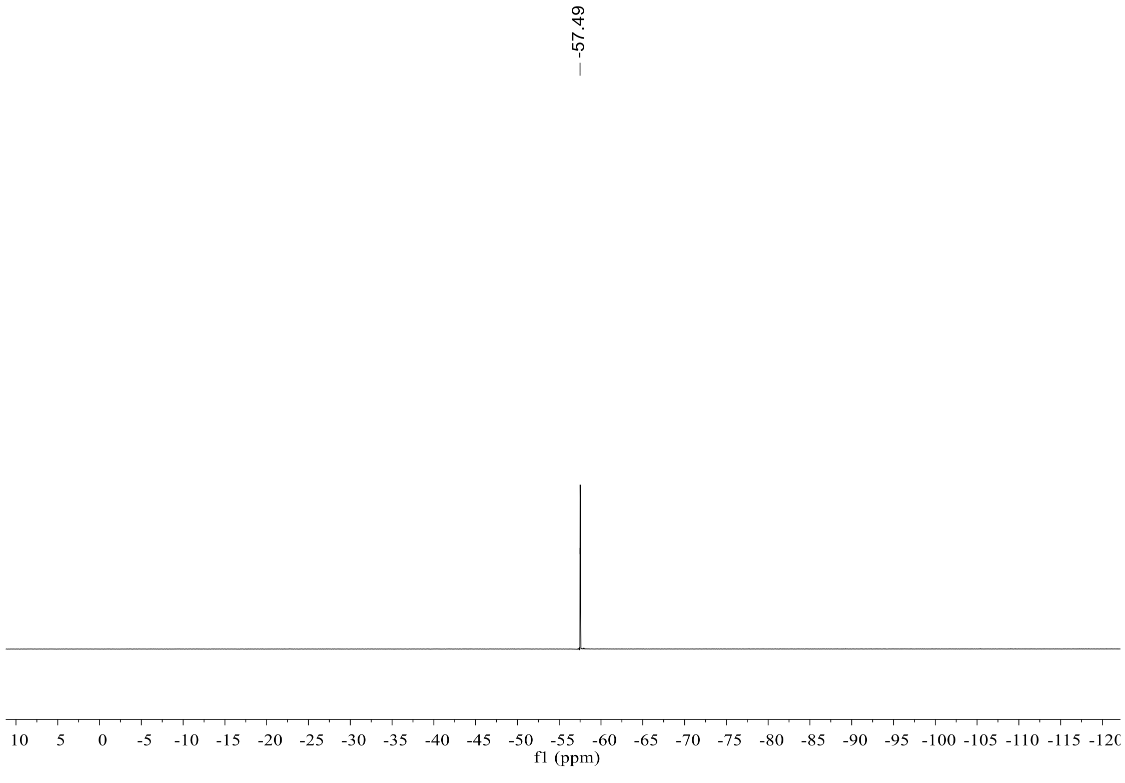

Supplementary Figure 283. ^{19}F NMR spectrum of **3qn**

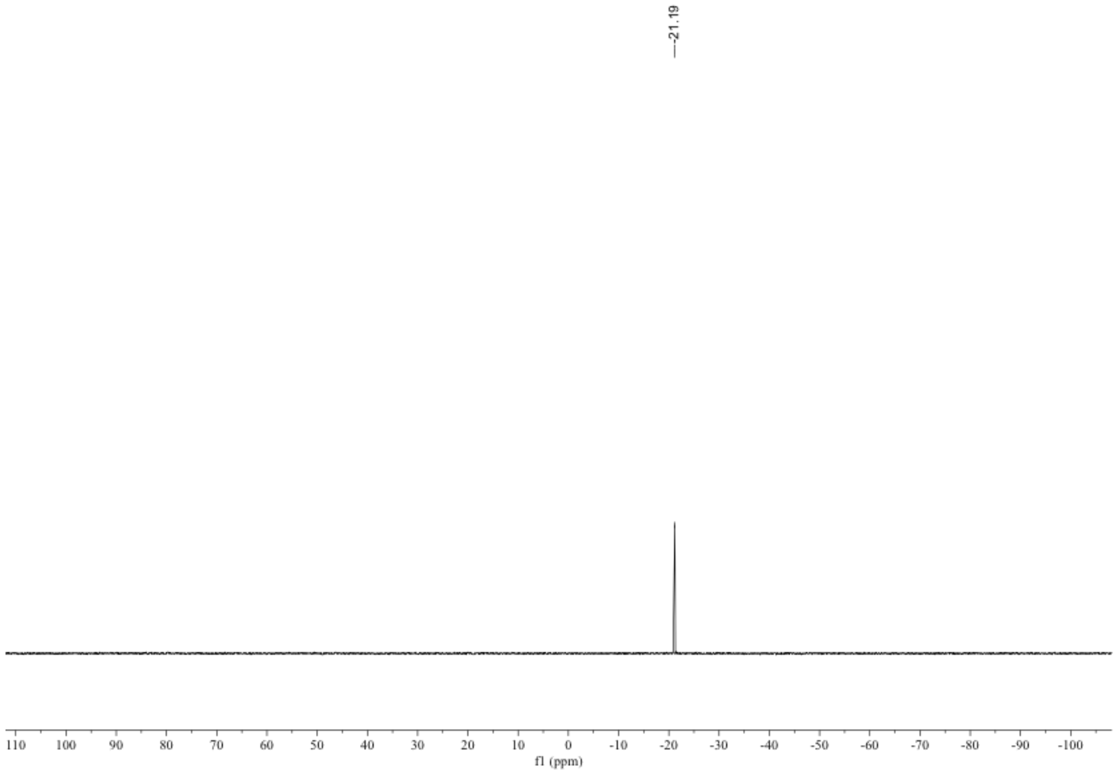

Supplementary Figure 284. ^{29}Si NMR spectrum of **3qn**

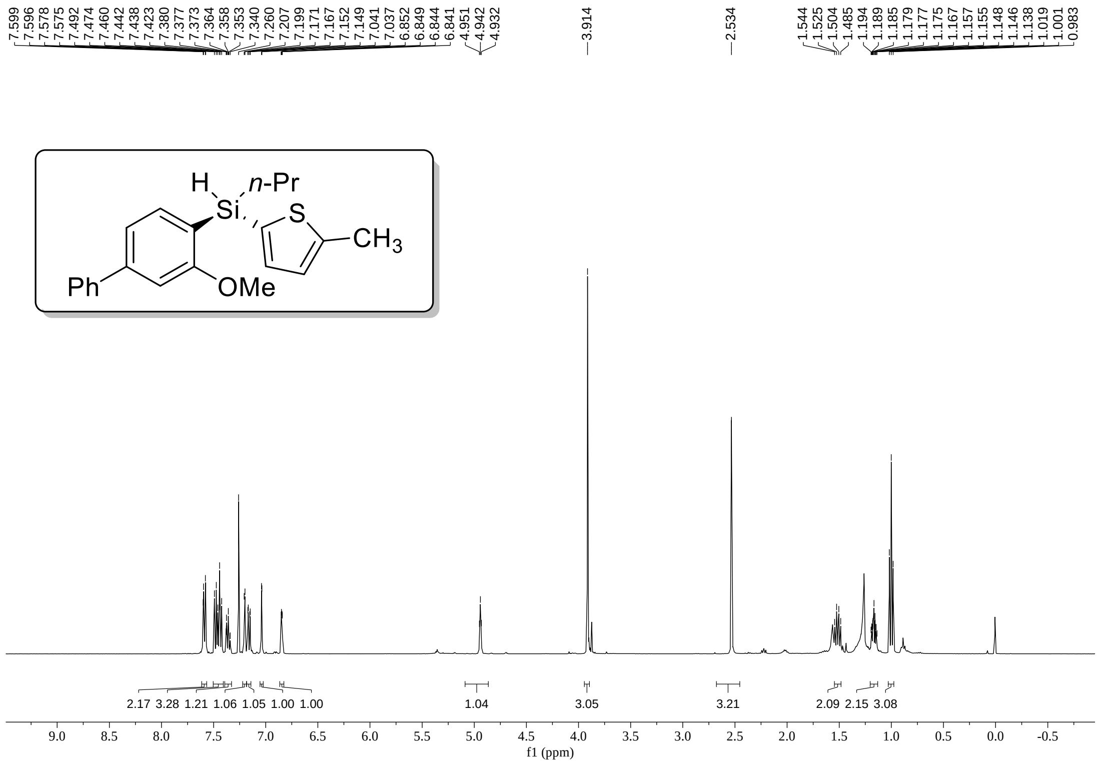

Supplementary Figure 285. ^{1}H NMR spectrum of **3ra**

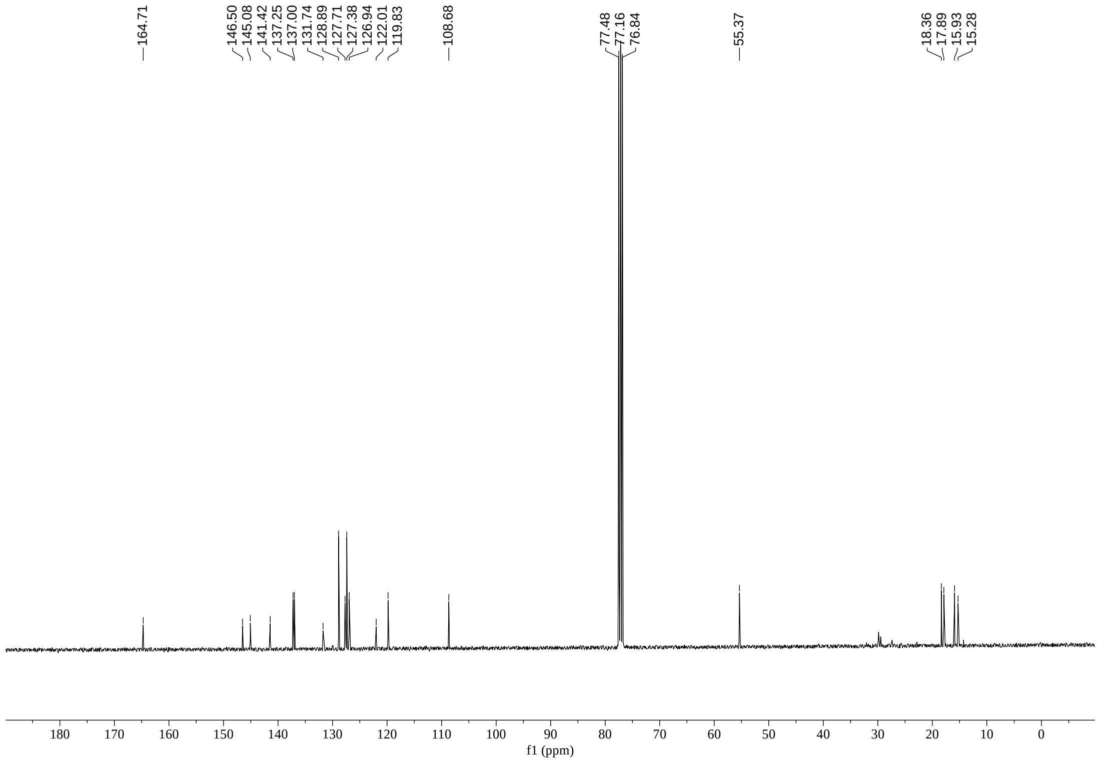

Supplementary Figure 286. ^{13}C NMR spectrum of **3ra**

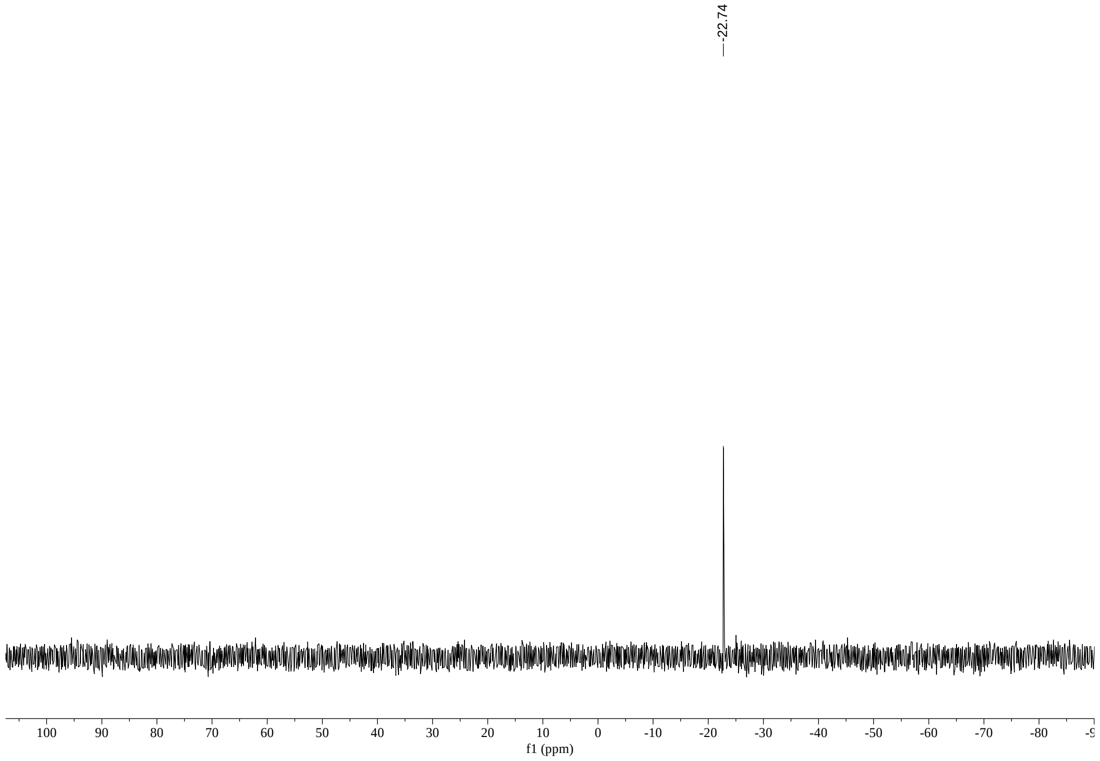

Supplementary Figure 287. ^{29}Si NMR spectrum of **3ra**

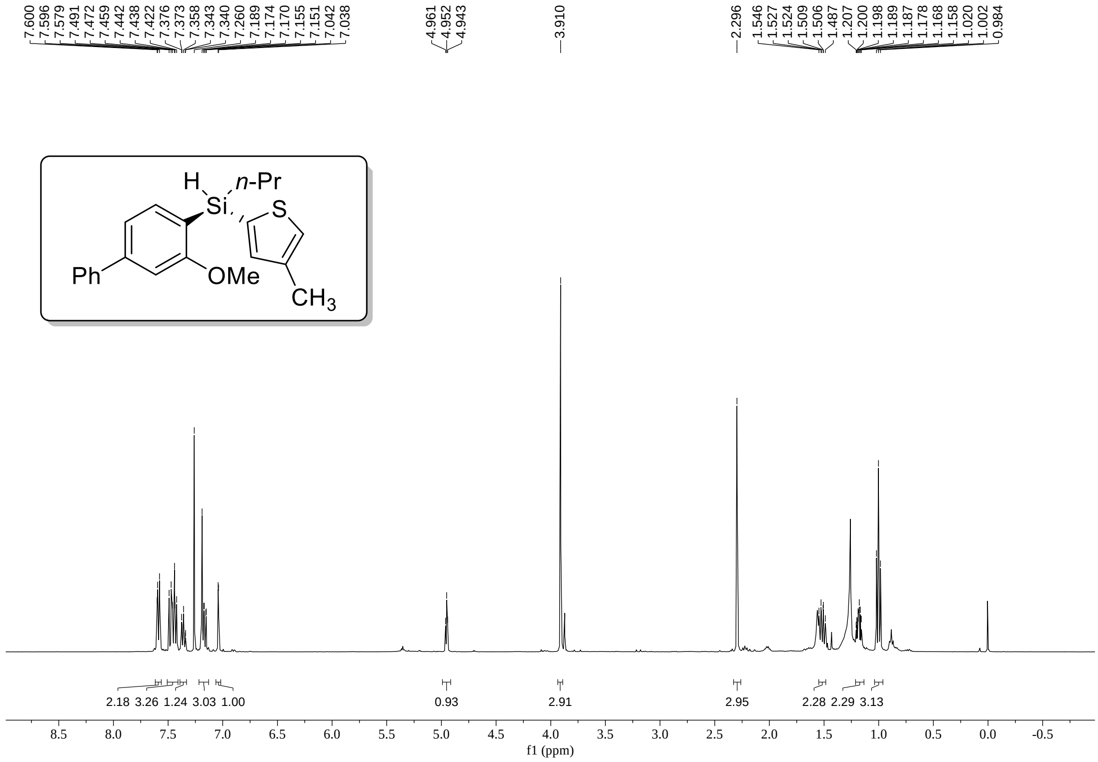

Supplementary Figure 288. ^{1}H NMR spectrum of **3ri**

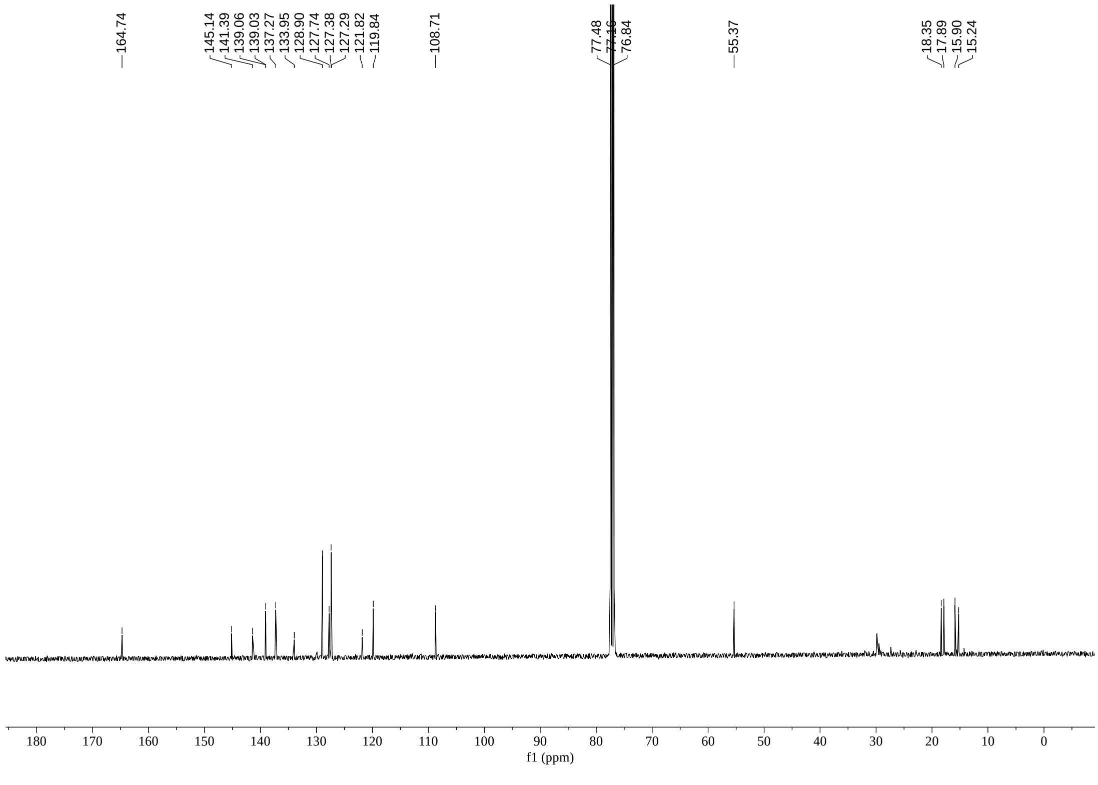

Supplementary Figure 289. ^{13}C NMR spectrum of **3ri**

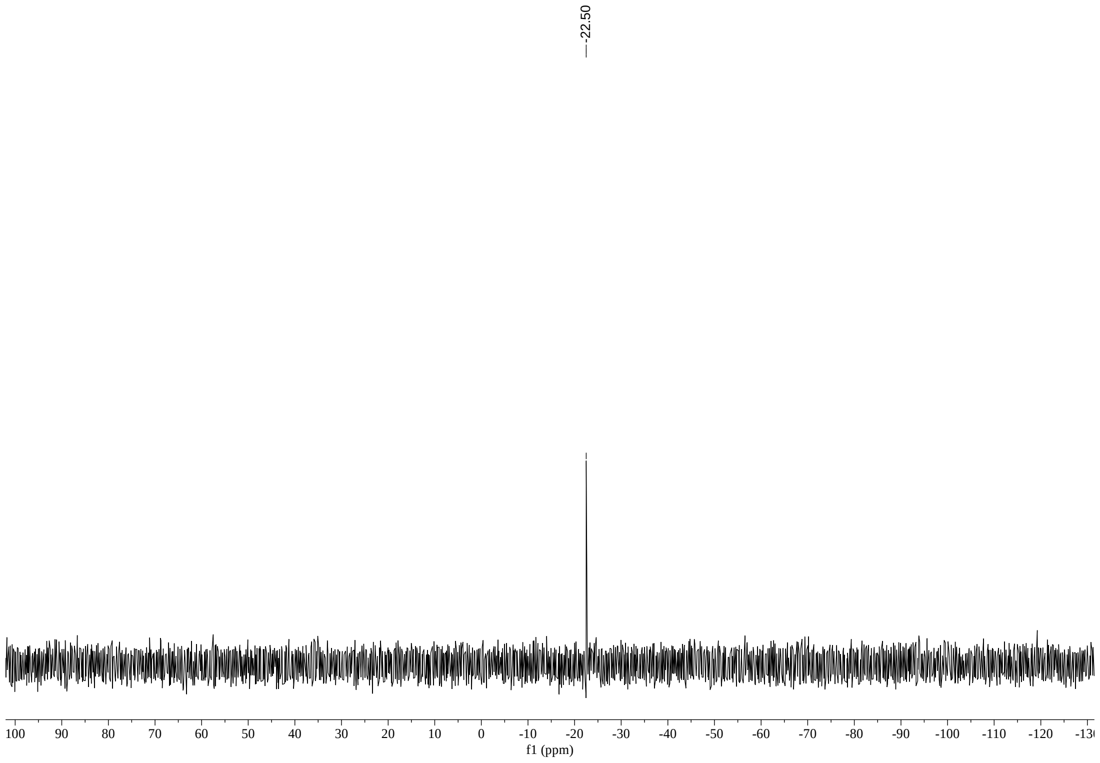

Supplementary Figure 290. ^{29}Si NMR spectrum of **3ri**

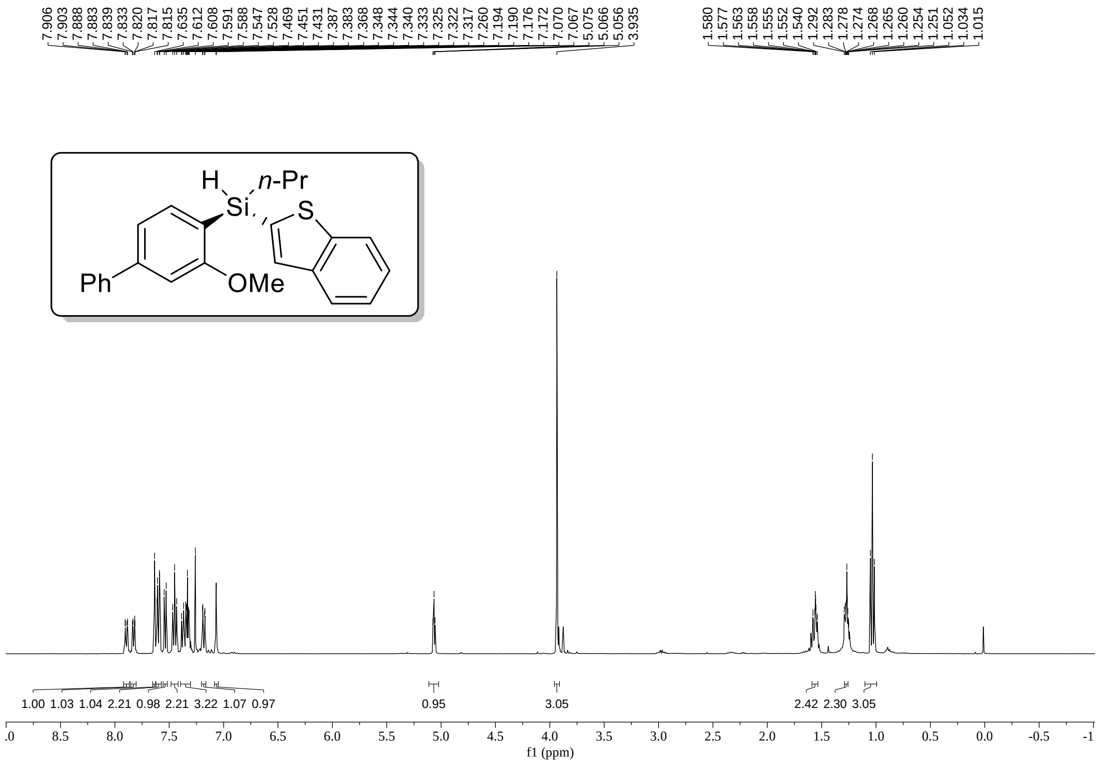

Supplementary Figure 291. ^{1}H NMR spectrum of **3rn**

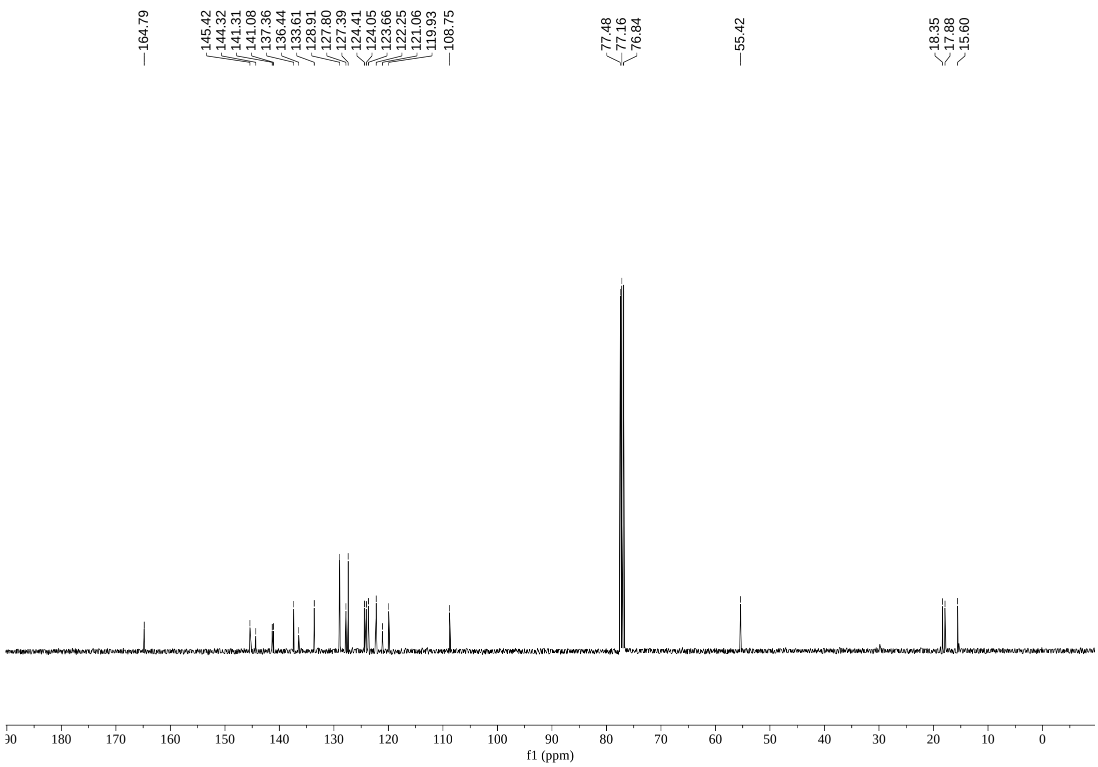

Supplementary Figure 292. ^{13}C NMR spectrum of **3rn**

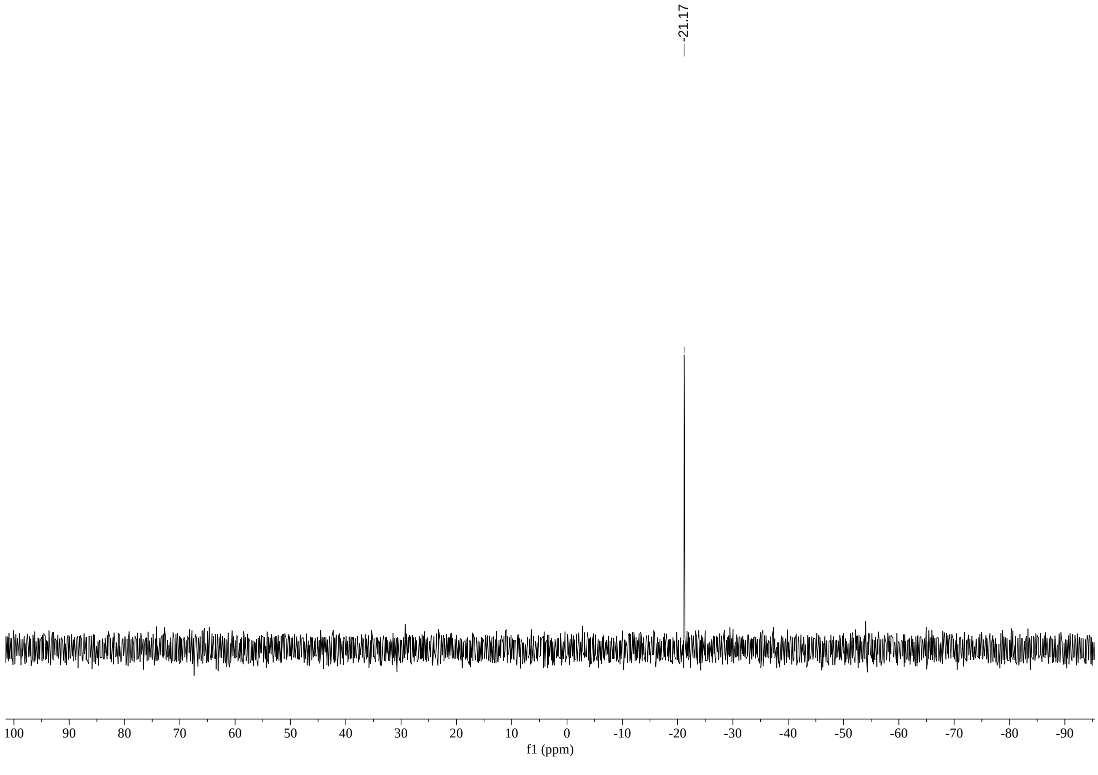

Supplementary Figure 293. ^{29}Si NMR spectrum of **3rn**

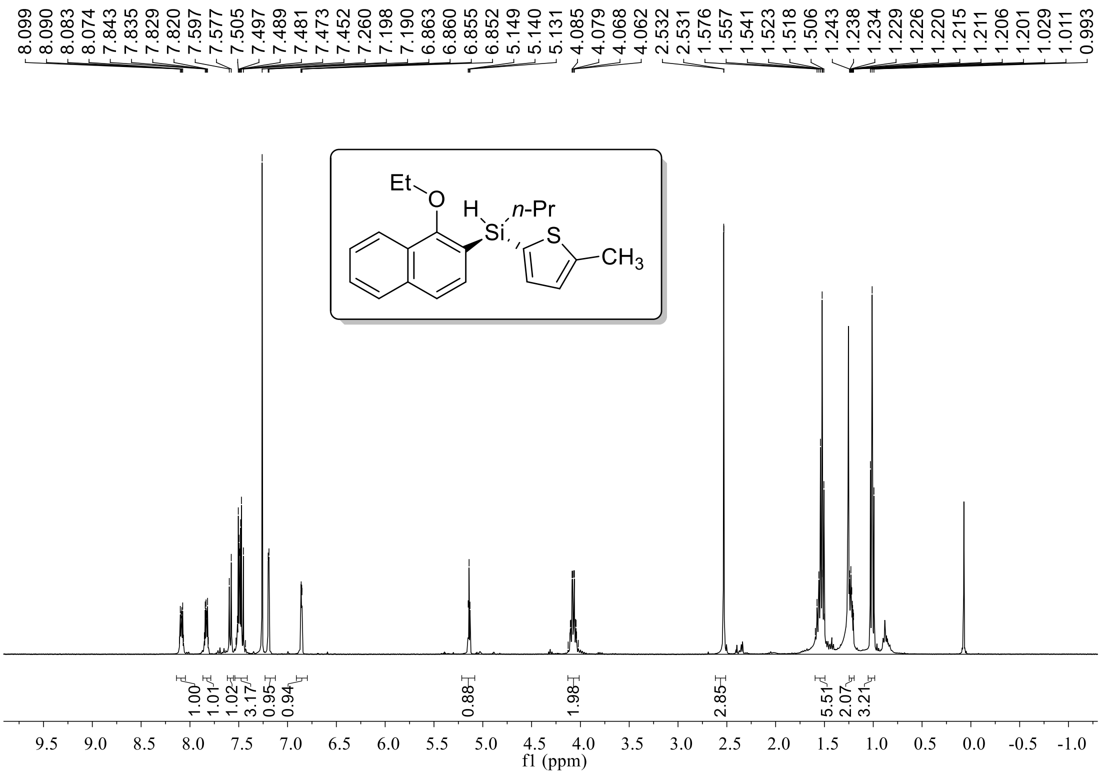

Supplementary Figure 294. ^{1}H NMR spectrum of **3sa**

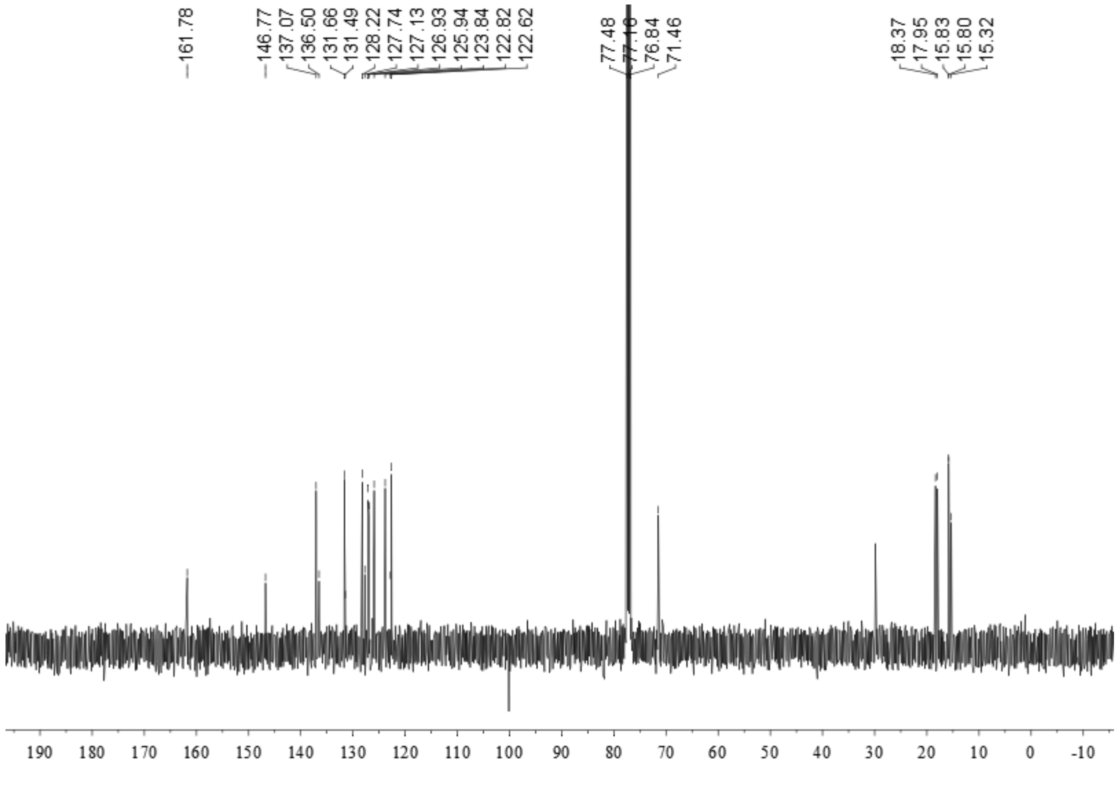

Supplementary Figure 295. ^{13}C NMR spectrum of **3sa**

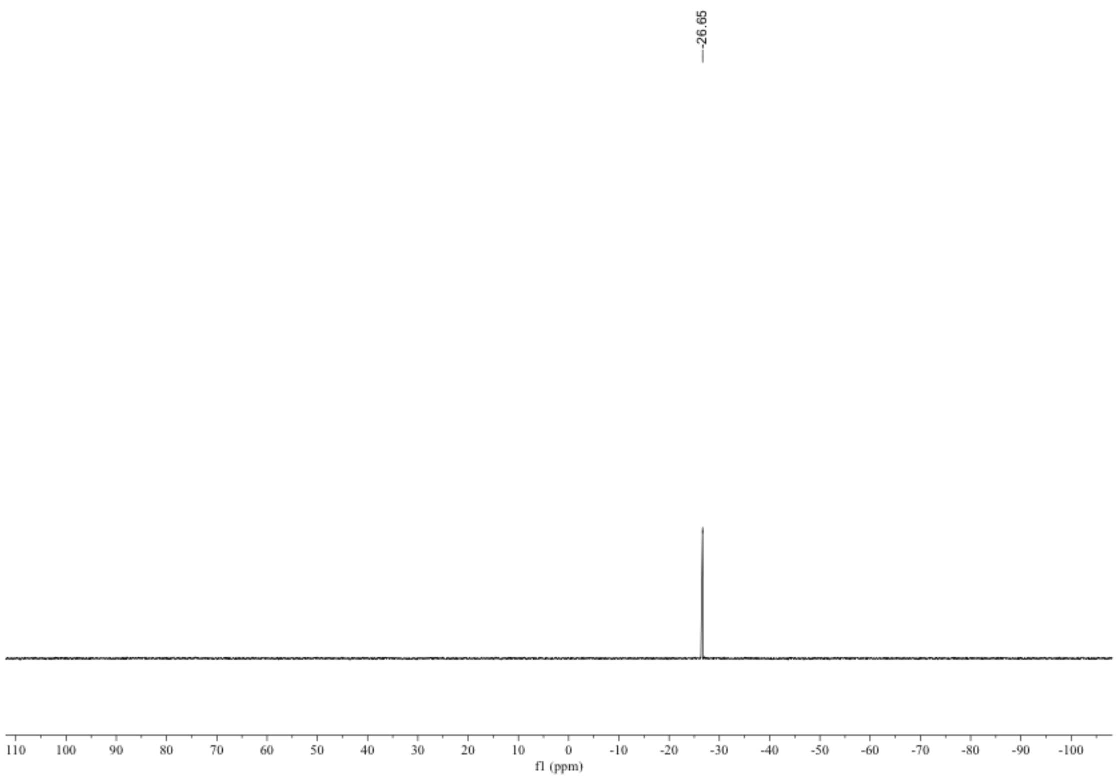

Supplementary Figure 296. ^{29}Si NMR spectrum of **3sa**

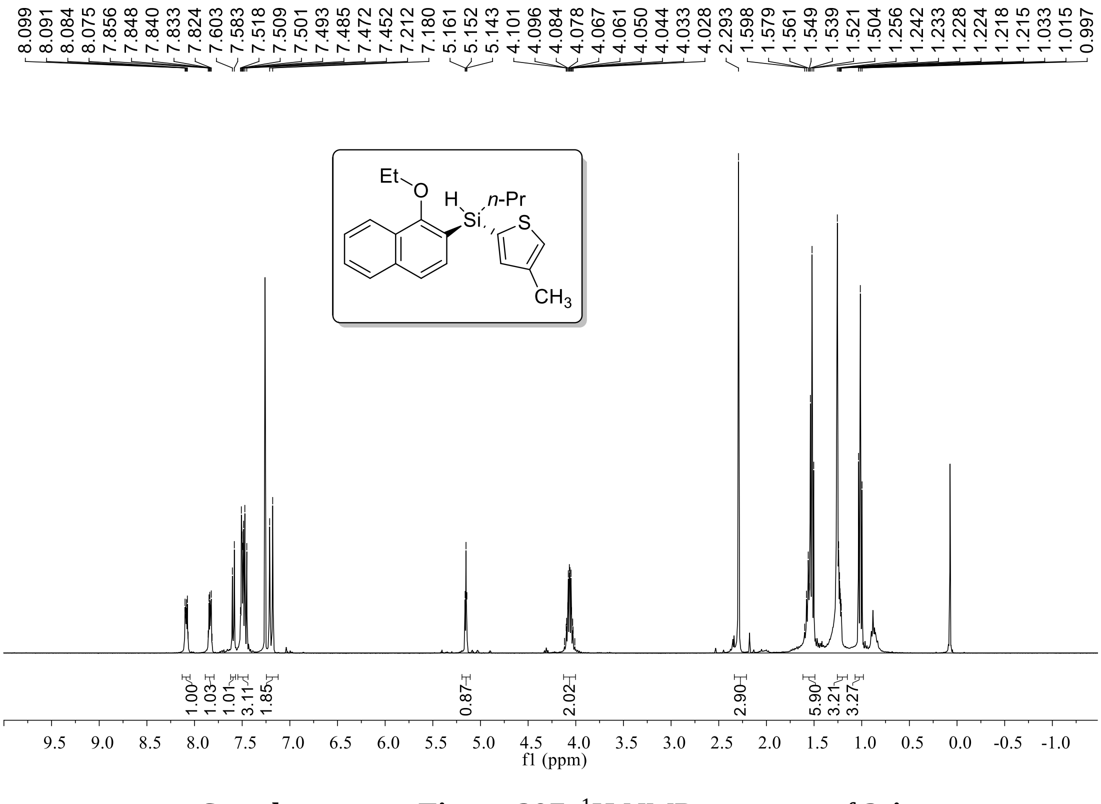

Supplementary Figure 297. ^{1}H NMR spectrum of **3si**

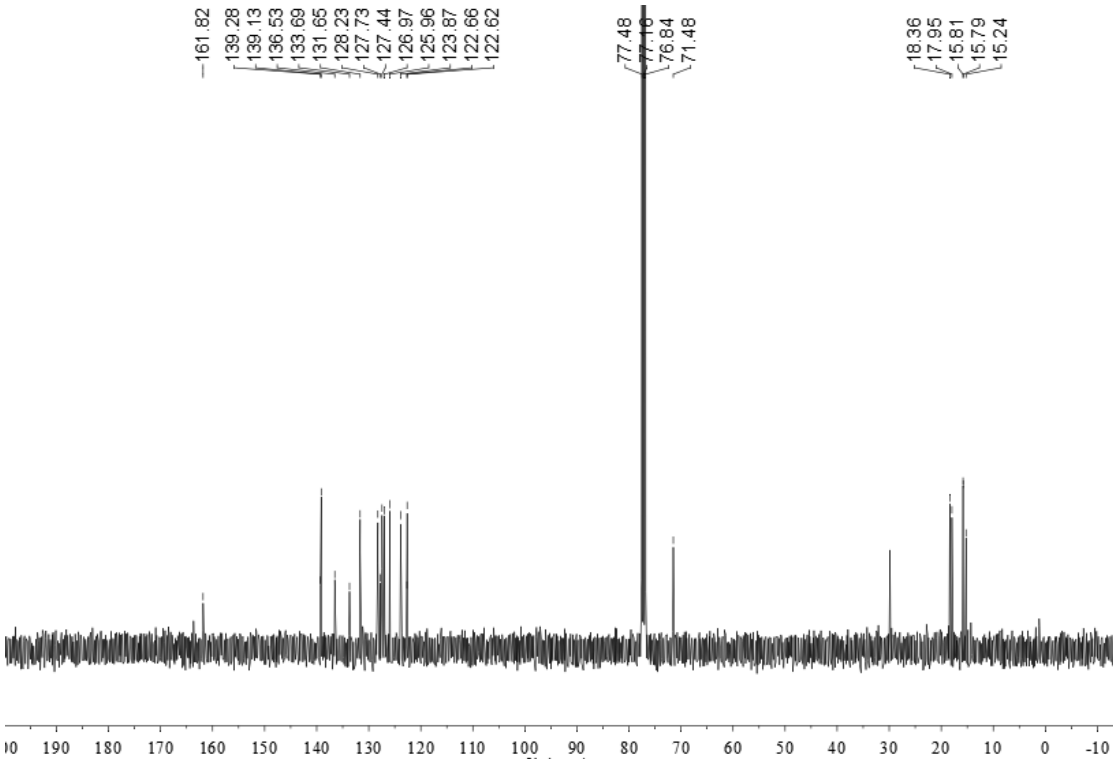

Supplementary Figure 298. ^{13}C NMR spectrum of **3si**

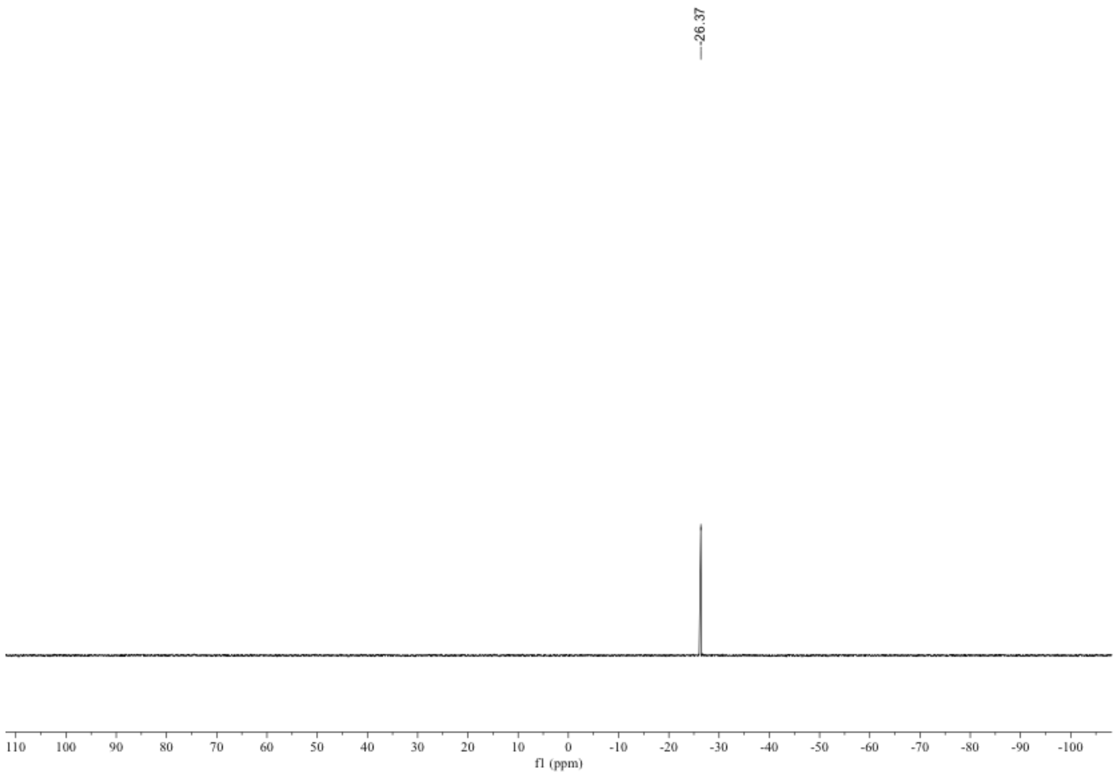

Supplementary Figure 299. ^{29}Si NMR spectrum of **3si**

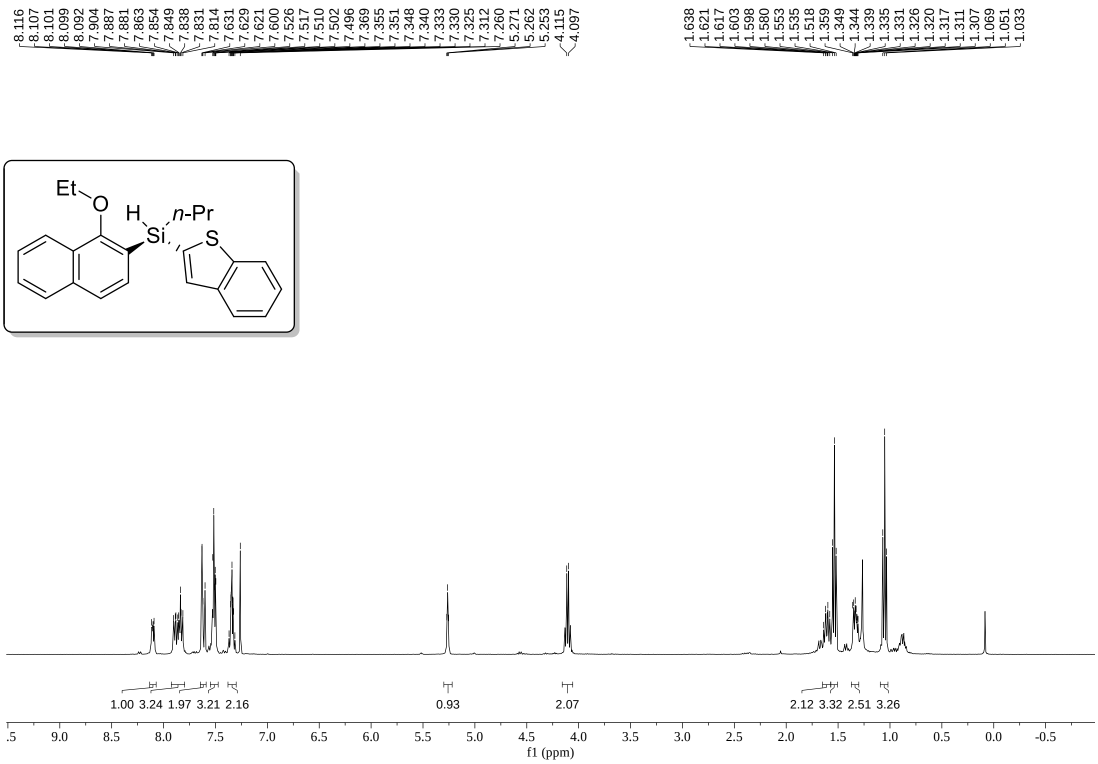

Supplementary Figure 300. ^{1}H NMR spectrum of **3sn**

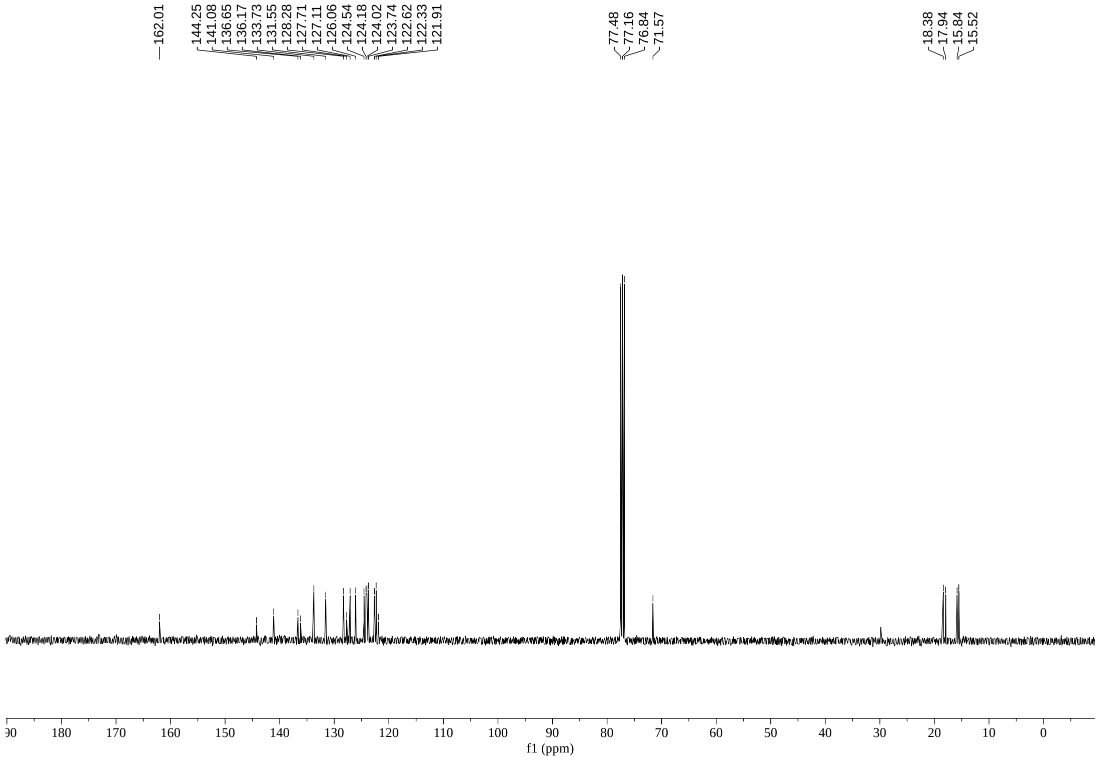

Supplementary Figure 301. ^{13}C NMR spectrum of **3sn**

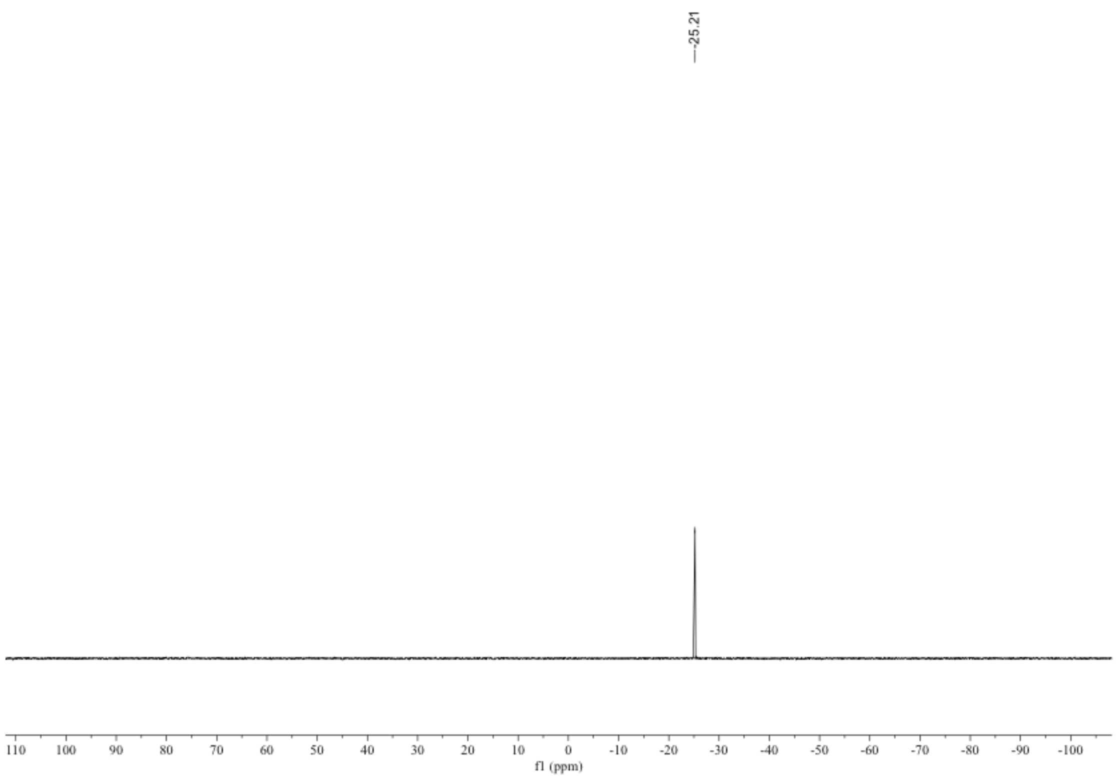

Supplementary Figure 302. ^{29}Si NMR spectrum of **3sn**

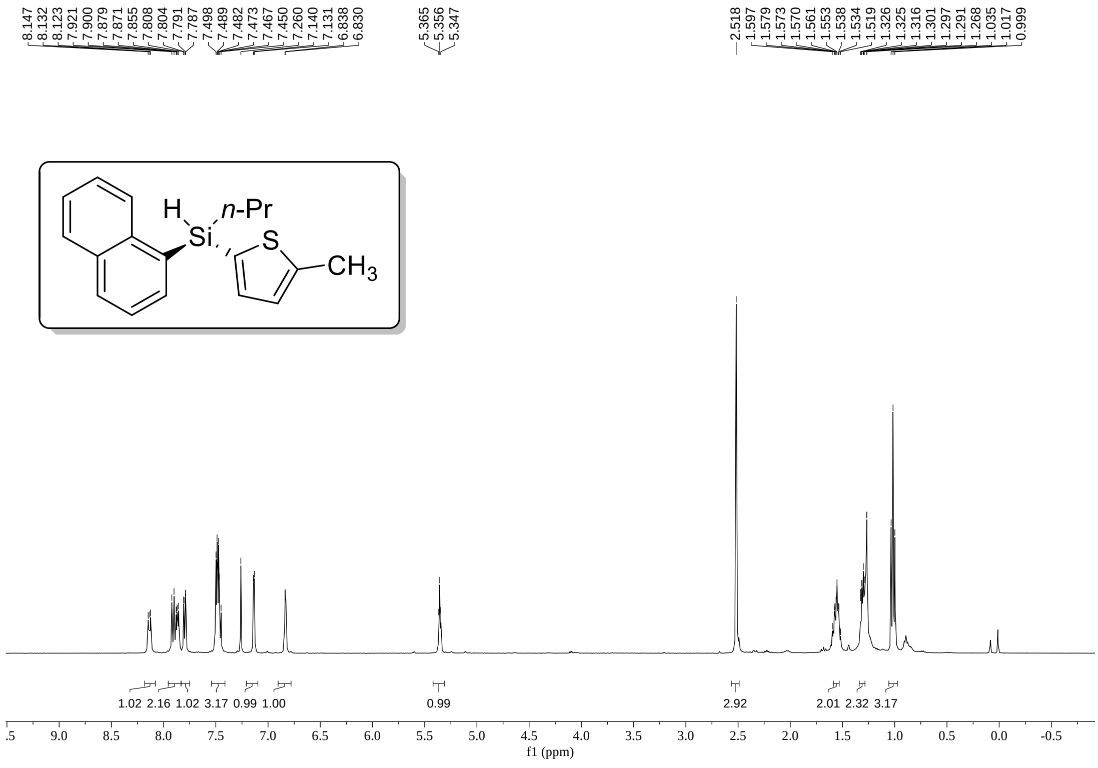

Supplementary Figure 303. ^{1}H NMR spectrum of **3ta**

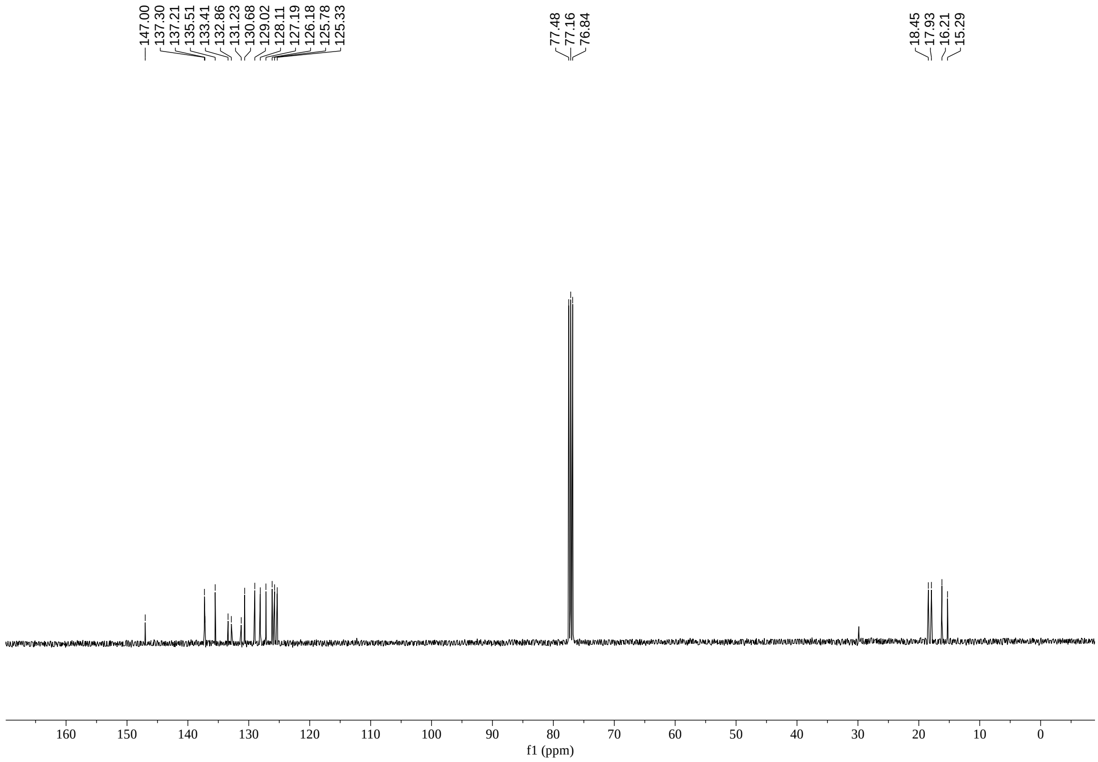

Supplementary Figure 304. ^{13}C NMR spectrum of **3ta**

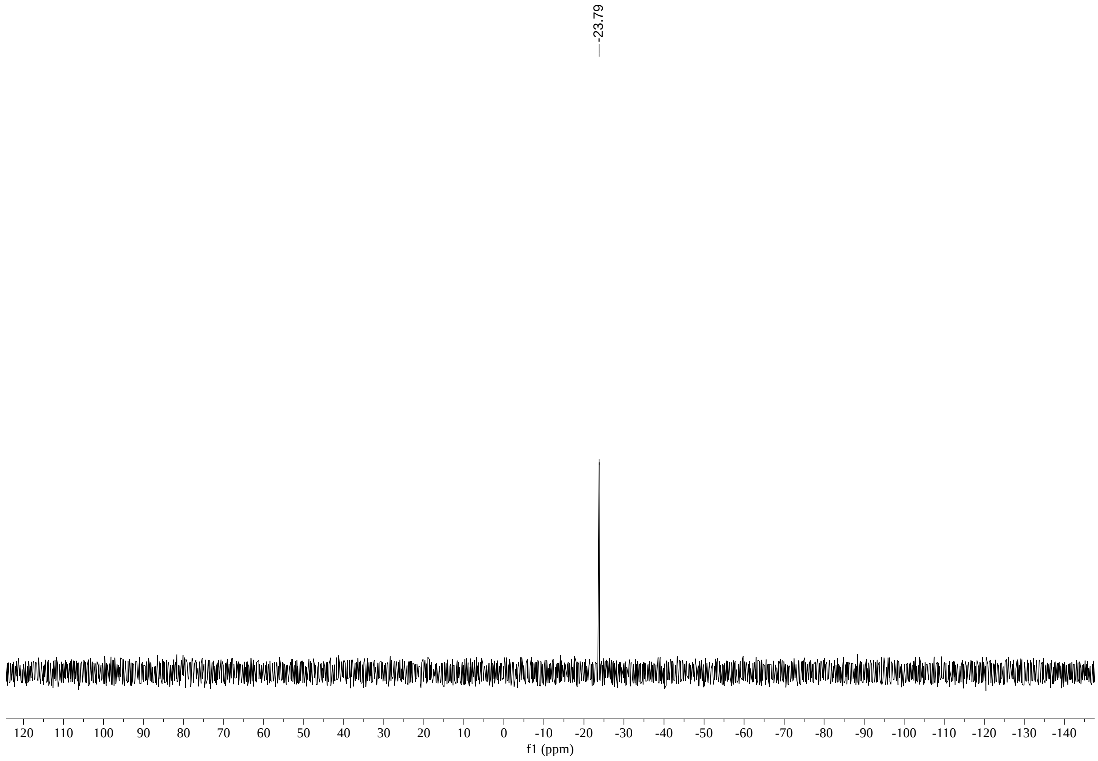

Supplementary Figure 305. ^{29}Si NMR spectrum of **3ta**

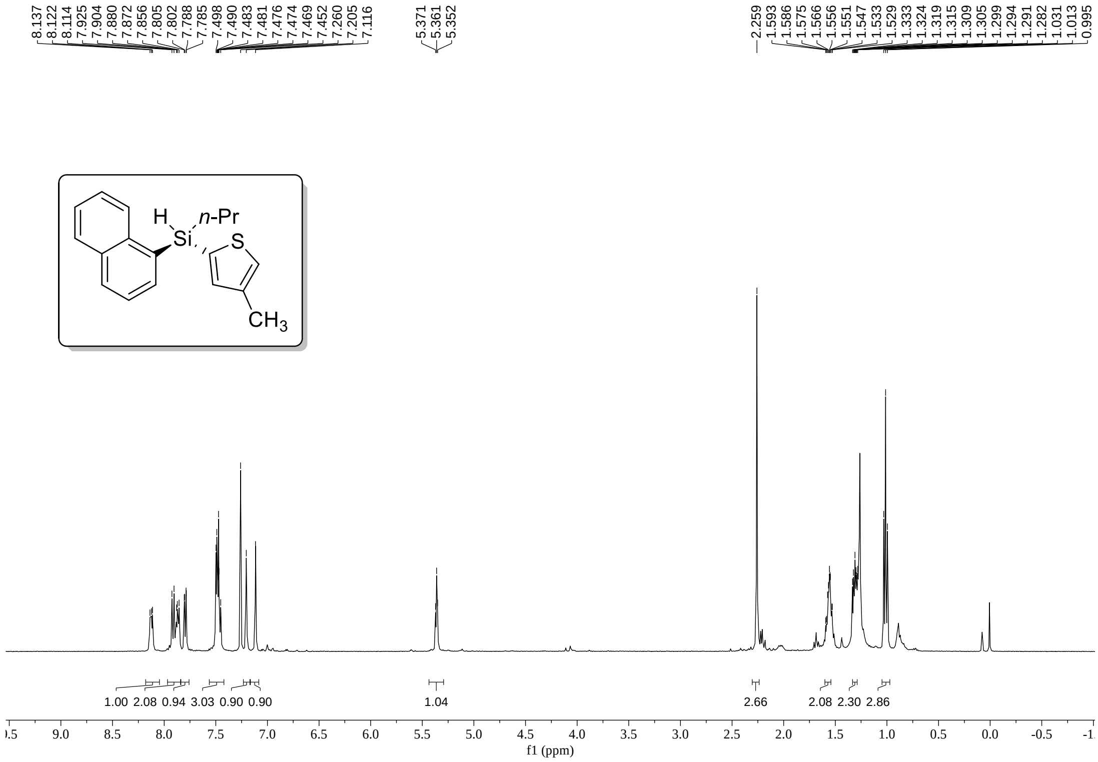

Supplementary Figure 306. ^{1}H NMR spectrum of **3ti**

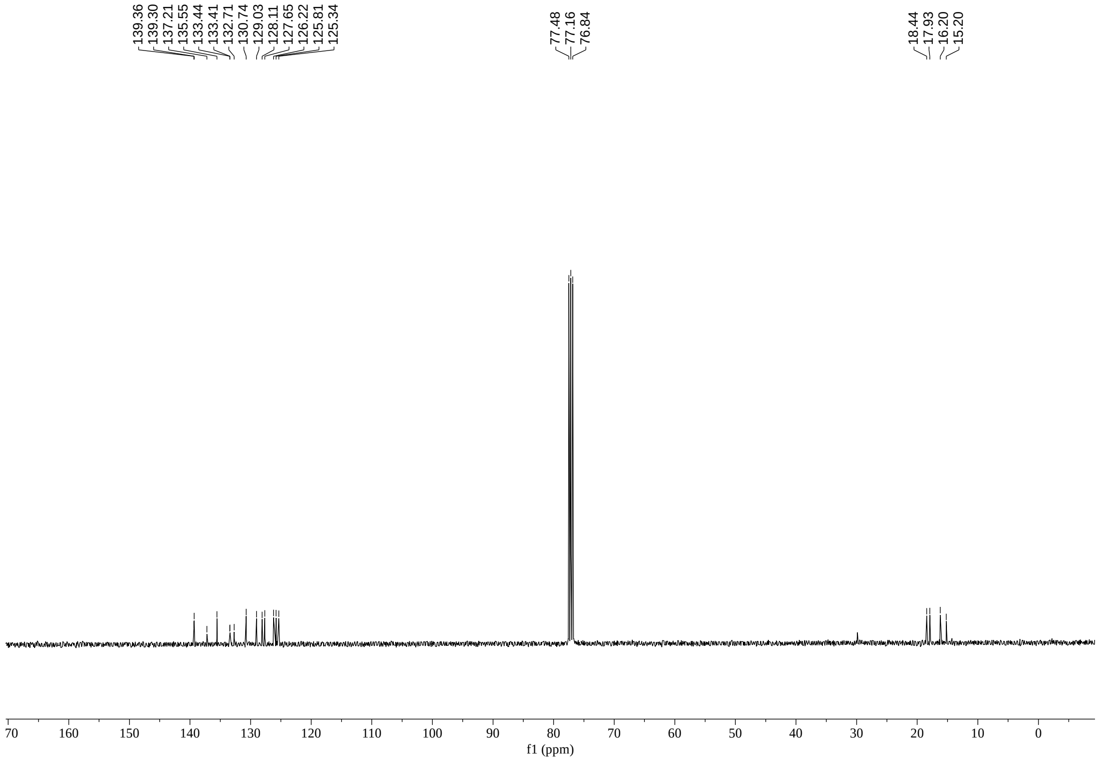

Supplementary Figure 307. ^{13}C NMR spectrum of **3ti**

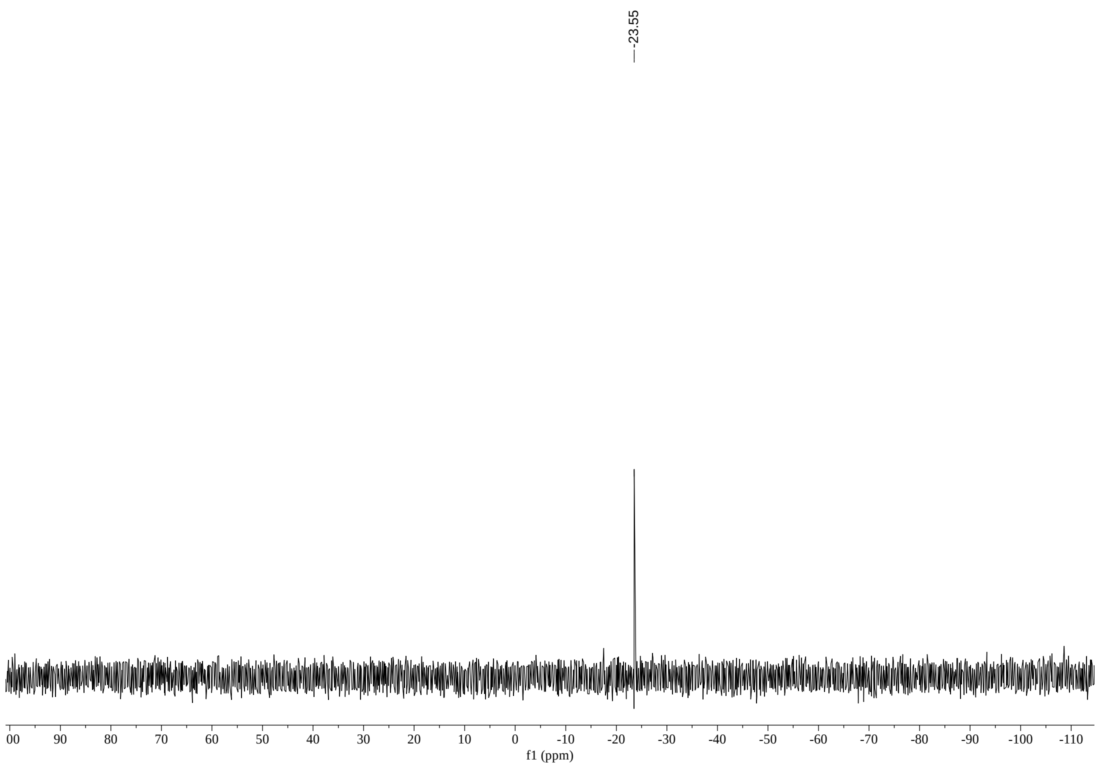

Supplementary Figure 308. ^{29}Si NMR spectrum of **3ti**

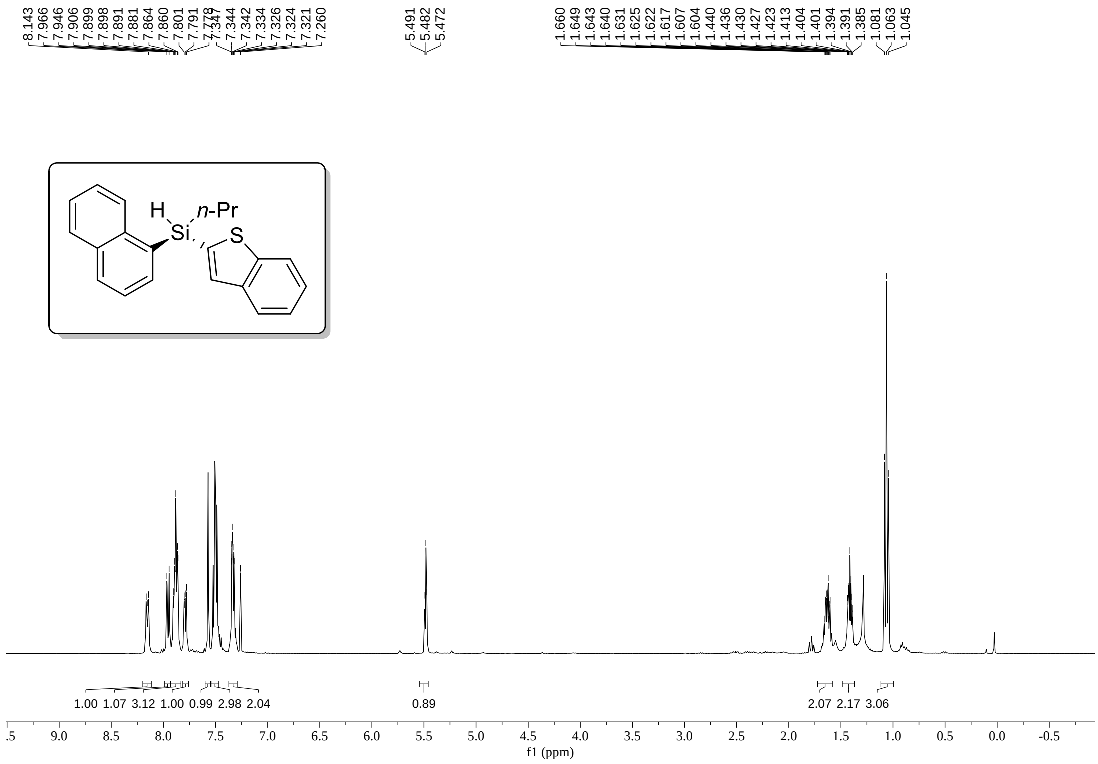

Supplementary Figure 309. ^{1}H NMR spectrum of **3tn**

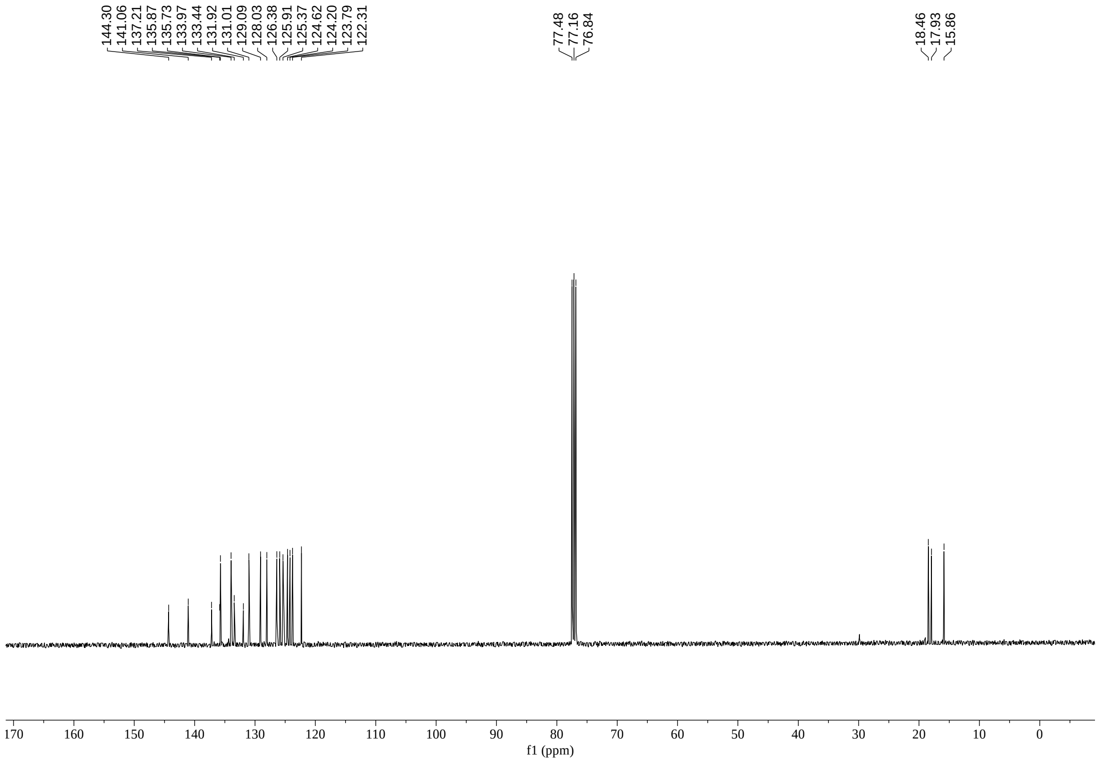

Supplementary Figure 310. ^{13}C NMR spectrum of **3tn**

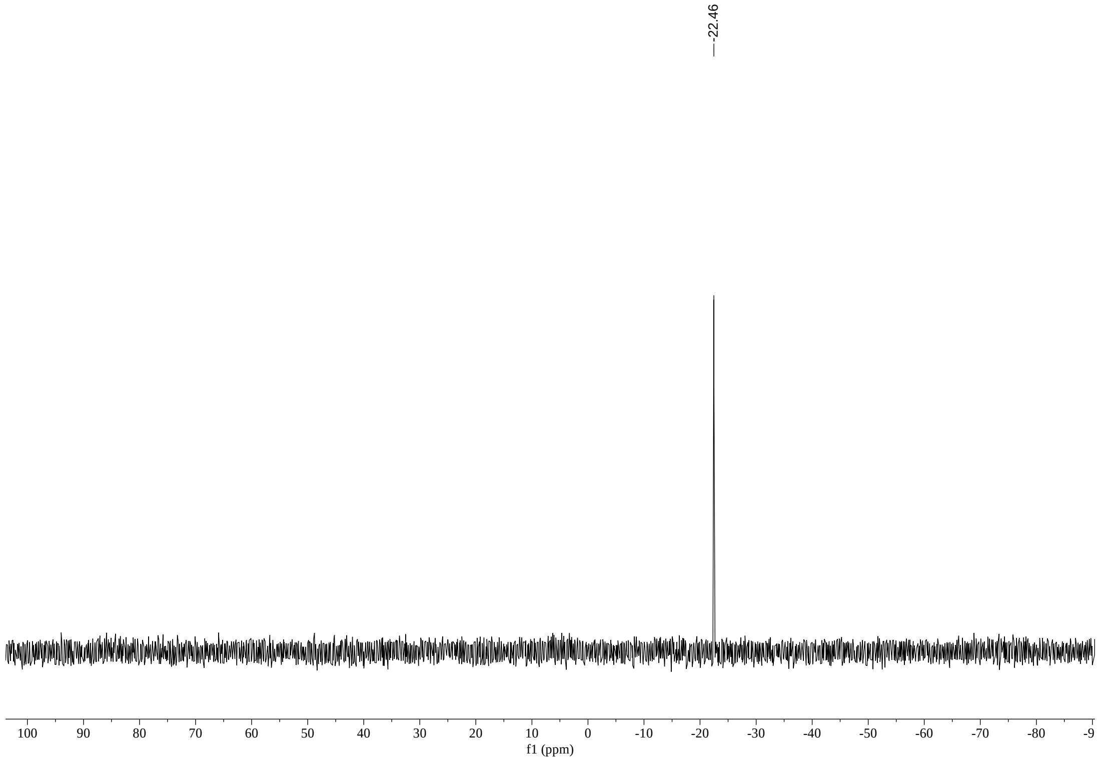

Supplementary Figure 311. ^{29}Si NMR spectrum of **3tn**

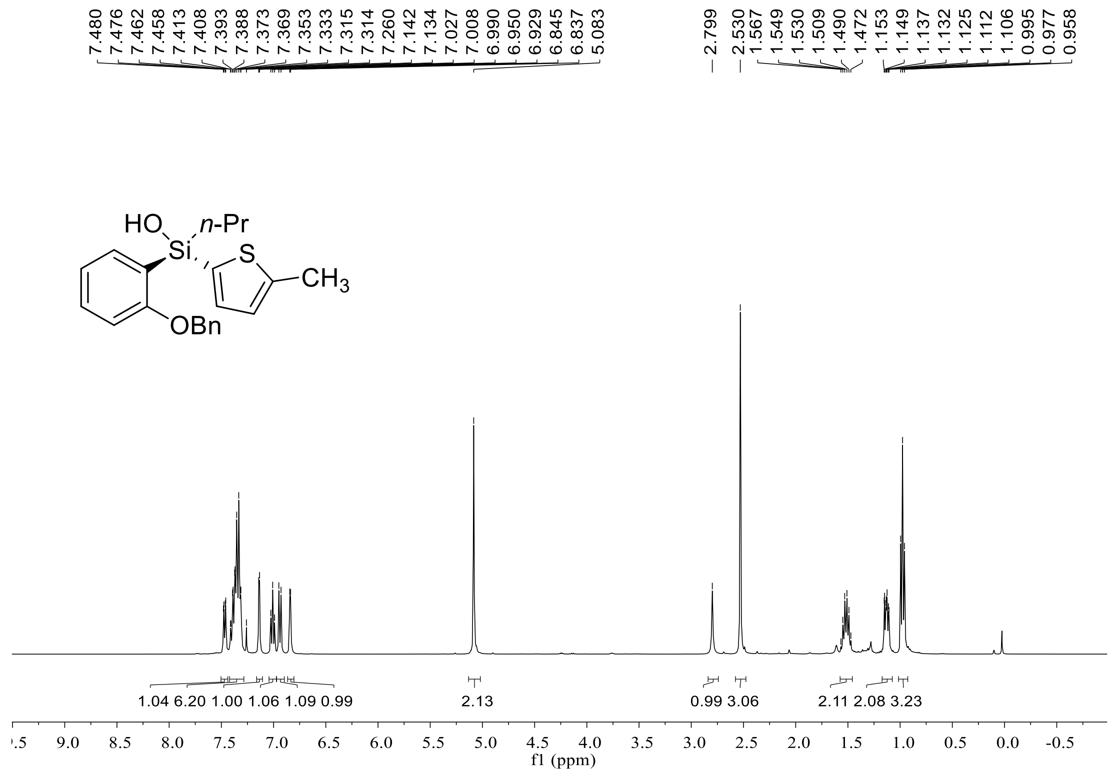

Supplementary Figure 312. ^{1}H NMR spectrum of **3aa-1**

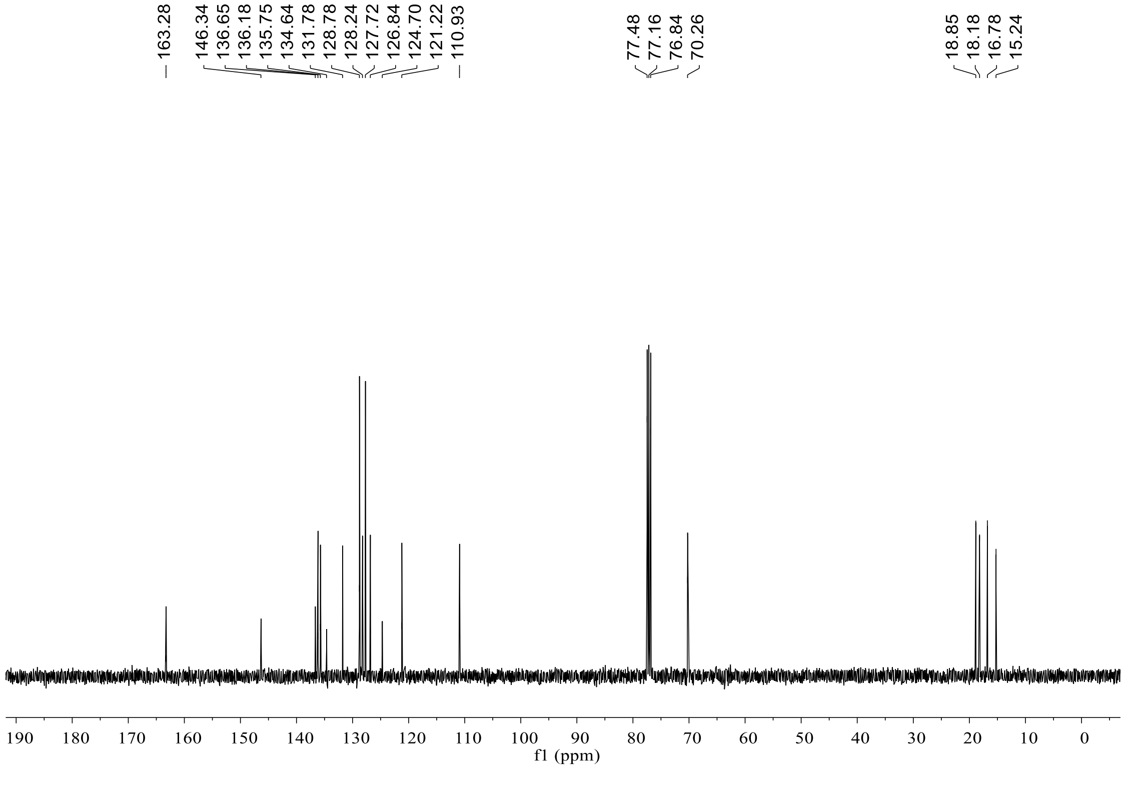

Supplementary Figure 313. ^{13}C NMR spectrum of **3aa-1**

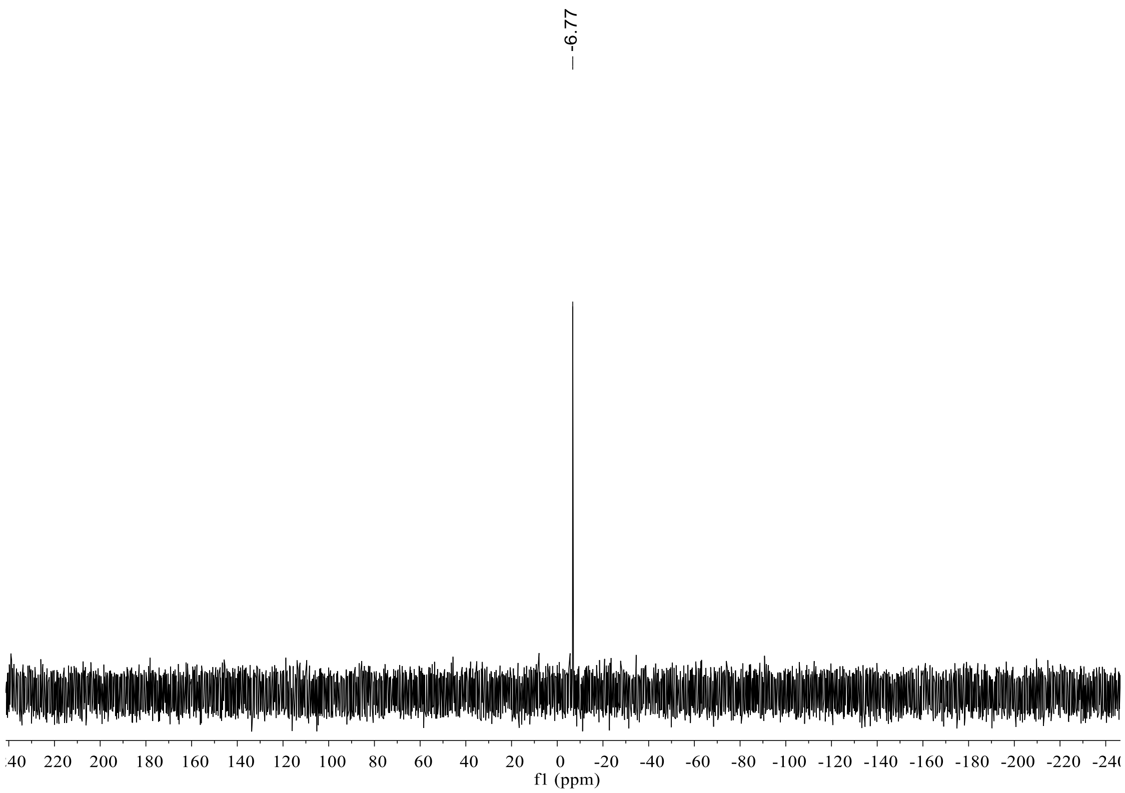

Supplementary Figure 314. ^{29}Si NMR spectrum of **3aa-1**

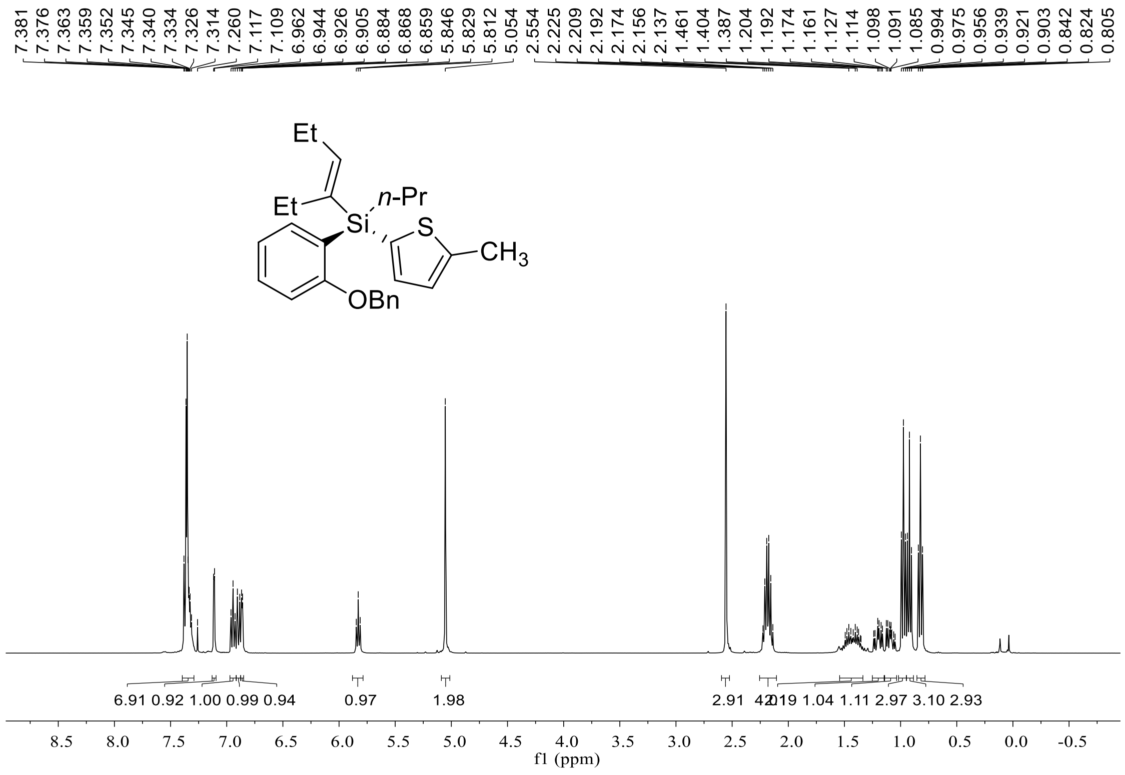

Supplementary Figure 315. ^{1}H NMR spectrum of **3aa-2**

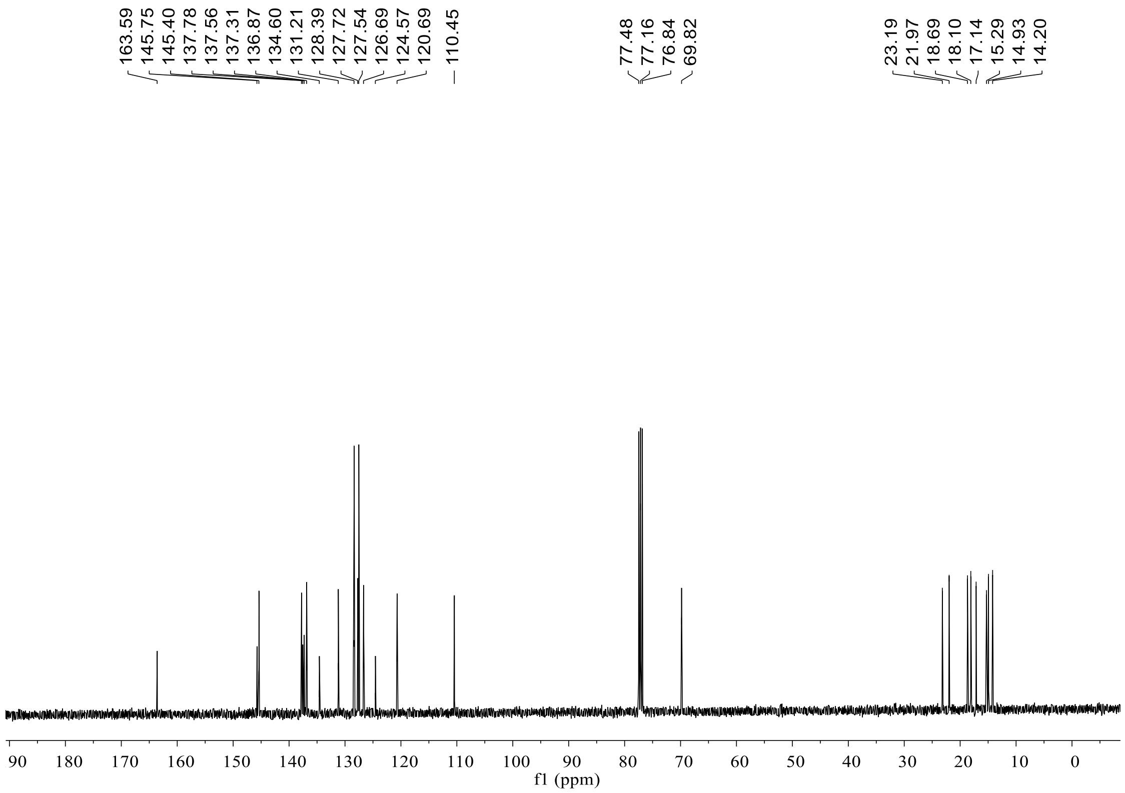

Supplementary Figure 316. ^{13}C NMR spectrum of **3aa-2**

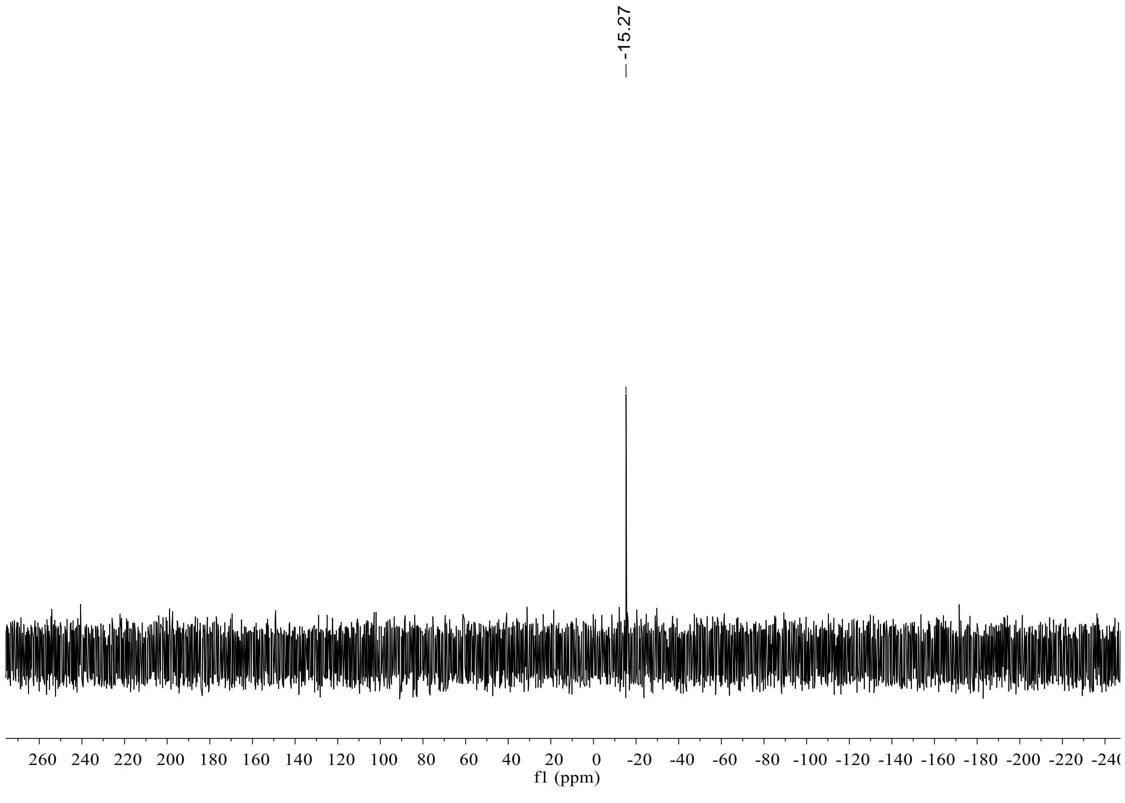

Supplementary Figure 317. ^{29}Si NMR spectrum of **3aa-2**

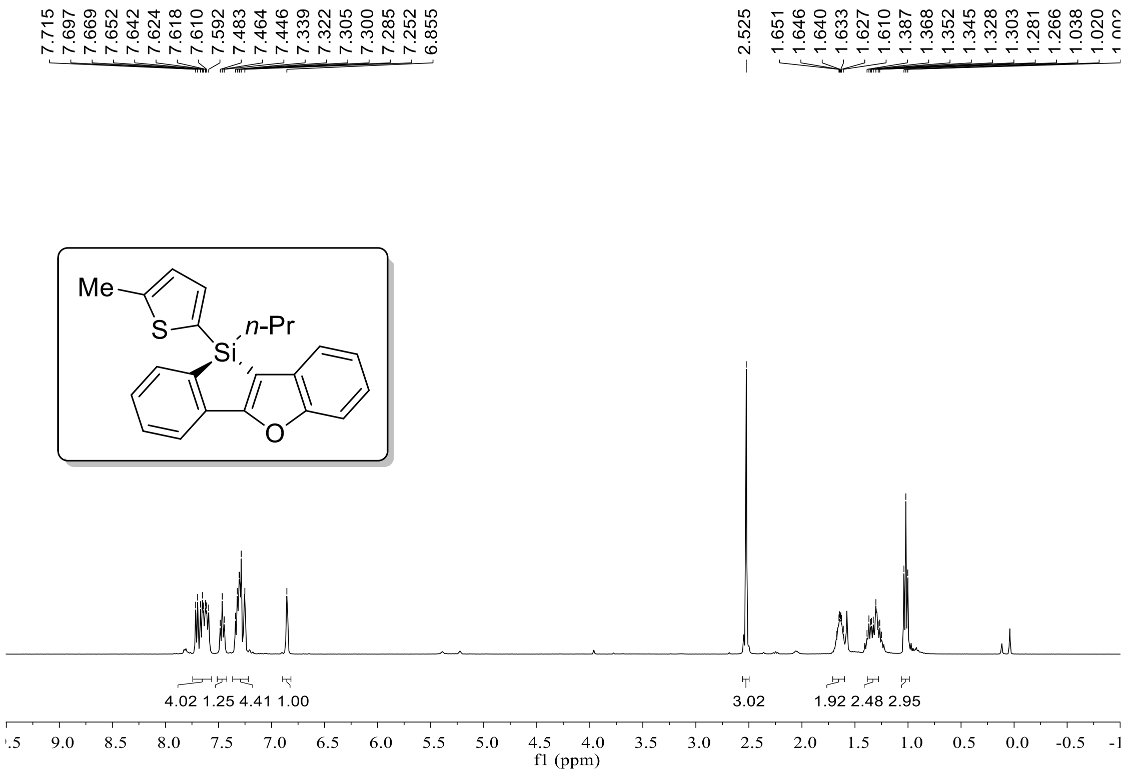

Supplementary Figure 318. ^{1}H NMR spectrum of **3va**

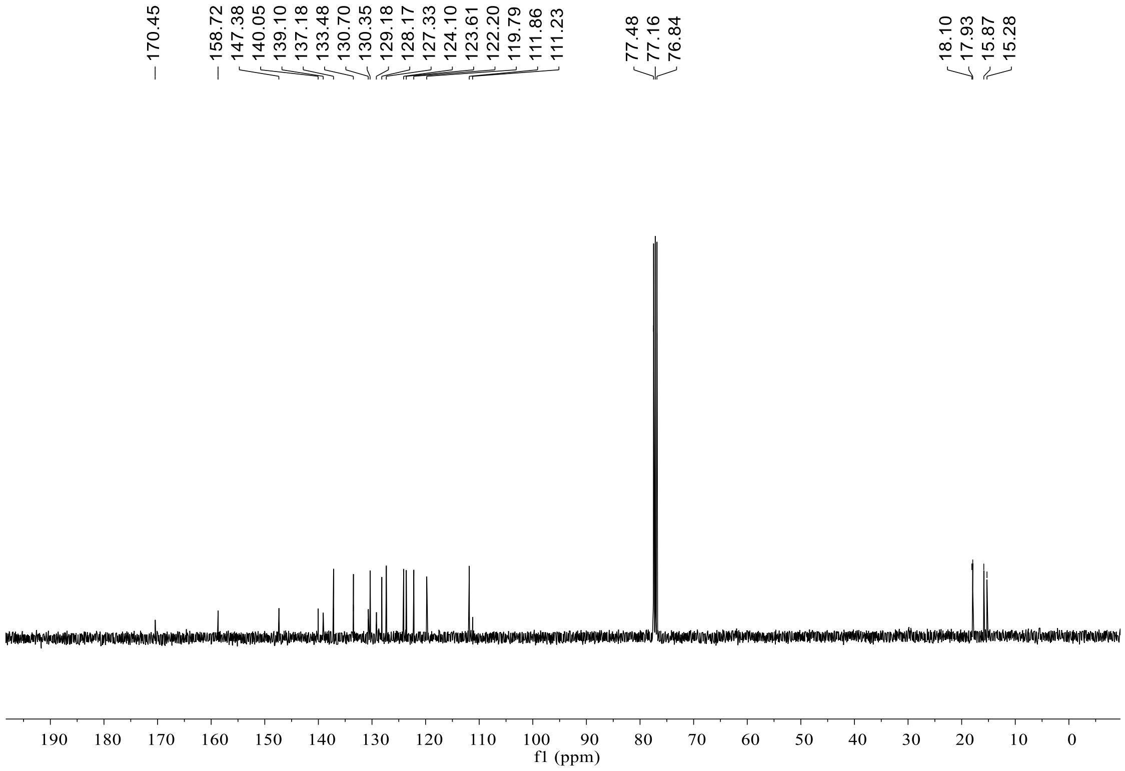

Supplementary Figure 319. ^{13}C NMR spectrum of **3va**

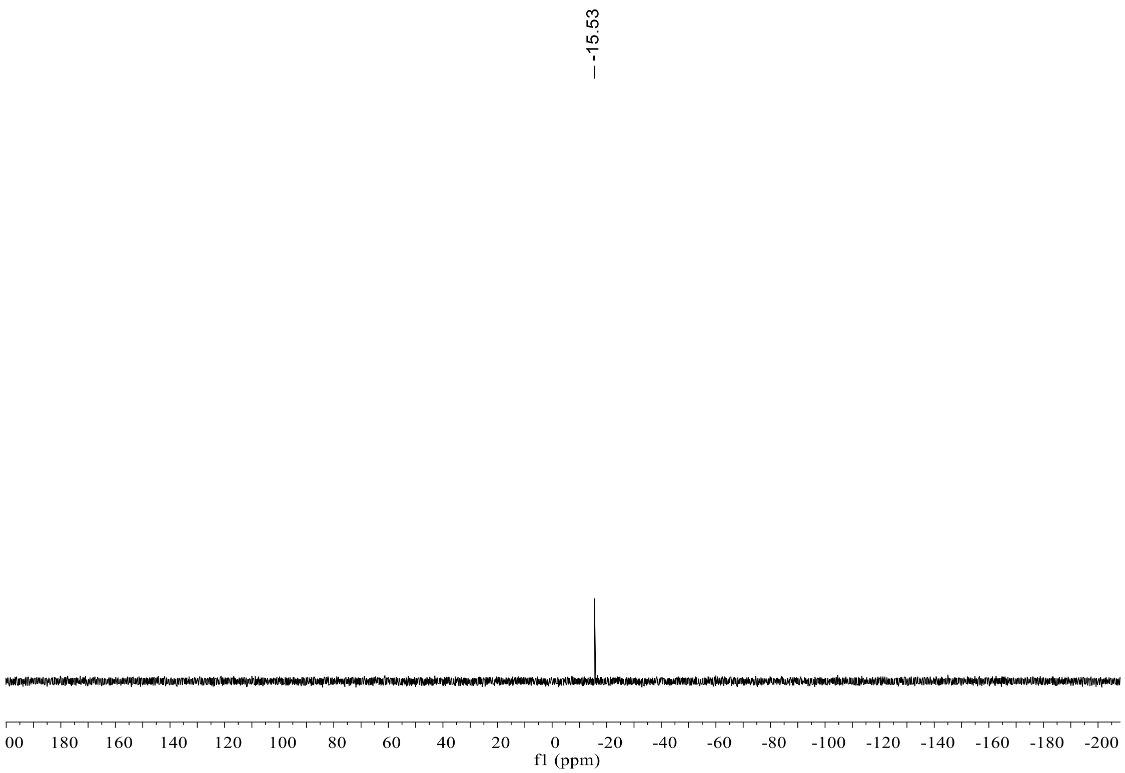

Supplementary Figure 320. ^{29}Si NMR spectrum of **3va**

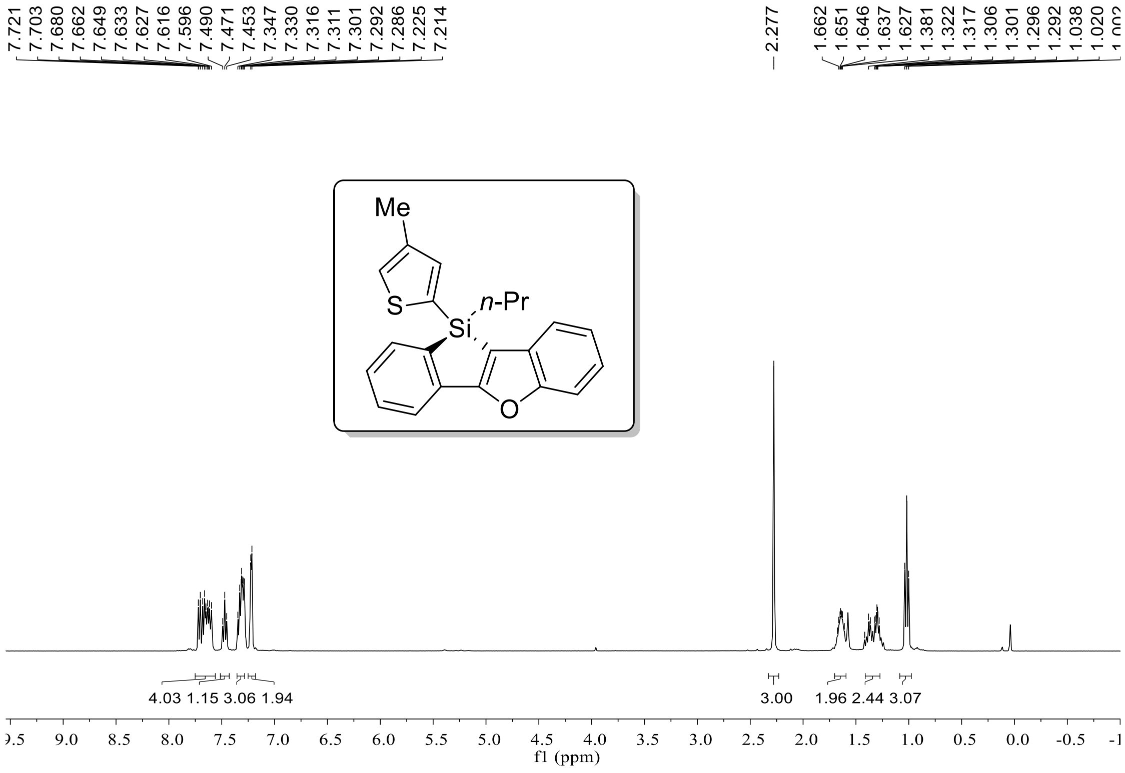

Supplementary Figure 321. ^{1}H NMR spectrum of **3vi**

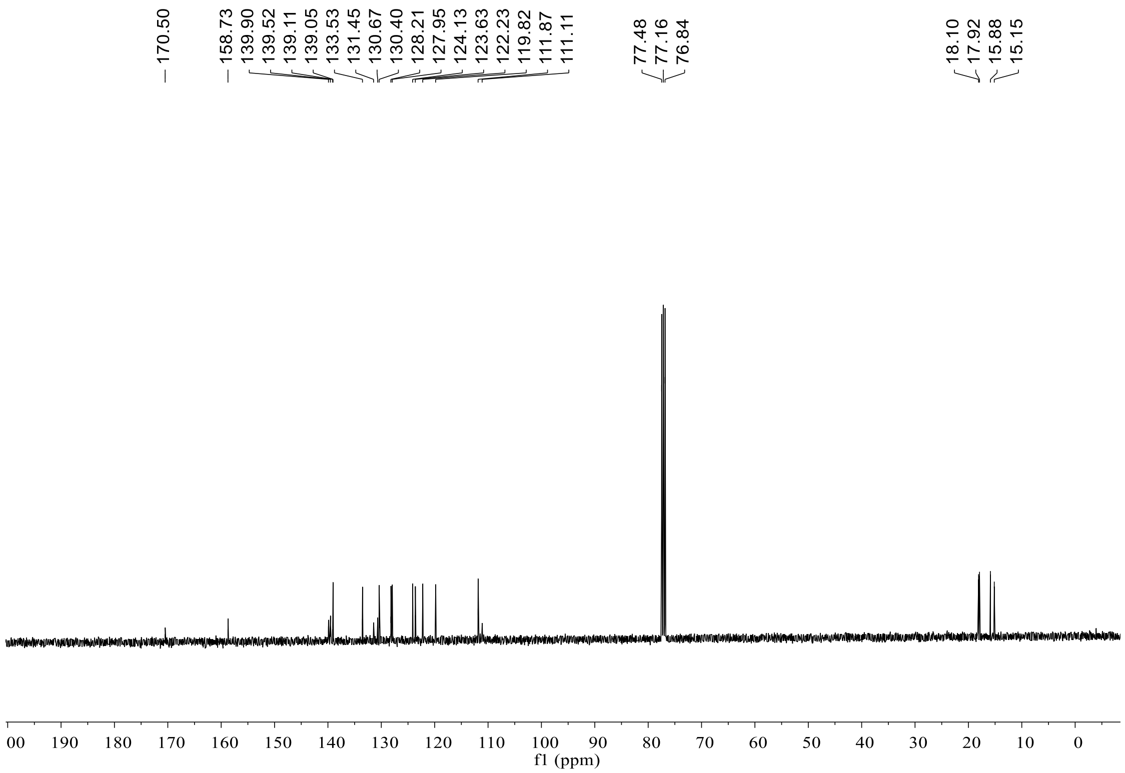

Supplementary Figure 322. ^{13}C NMR spectrum of **3vi**

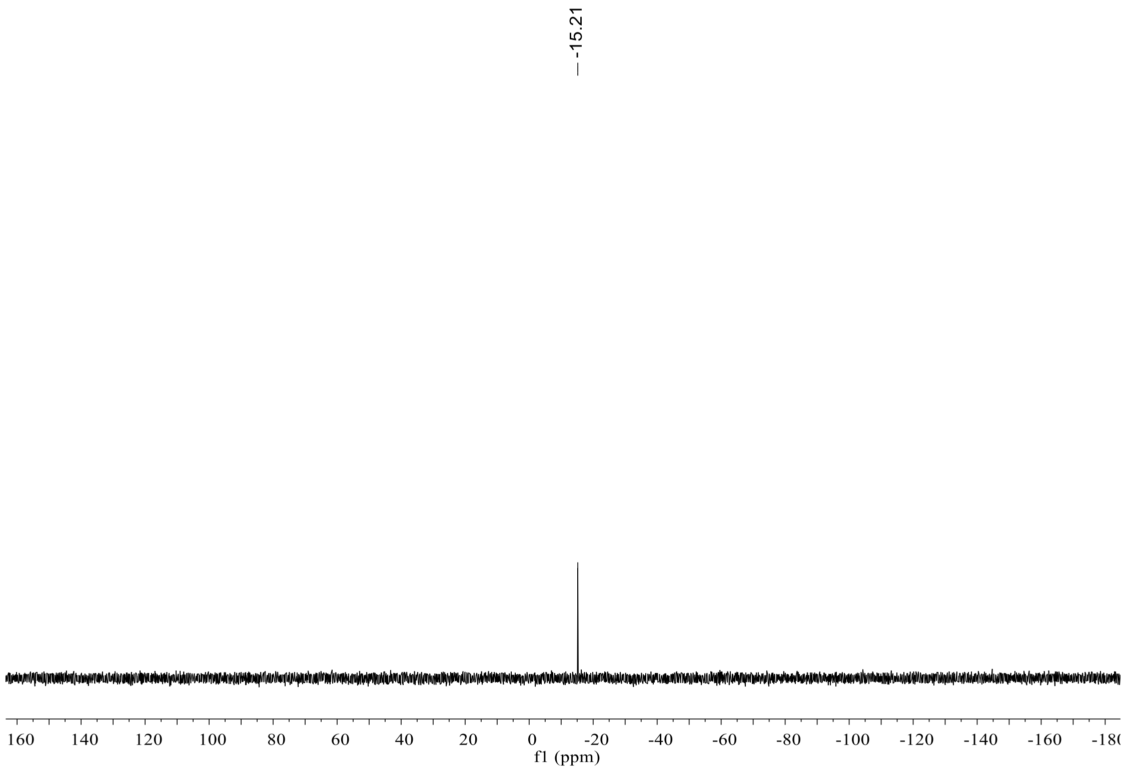

Supplementary Figure 323. ^{29}Si NMR spectrum of **3vi**

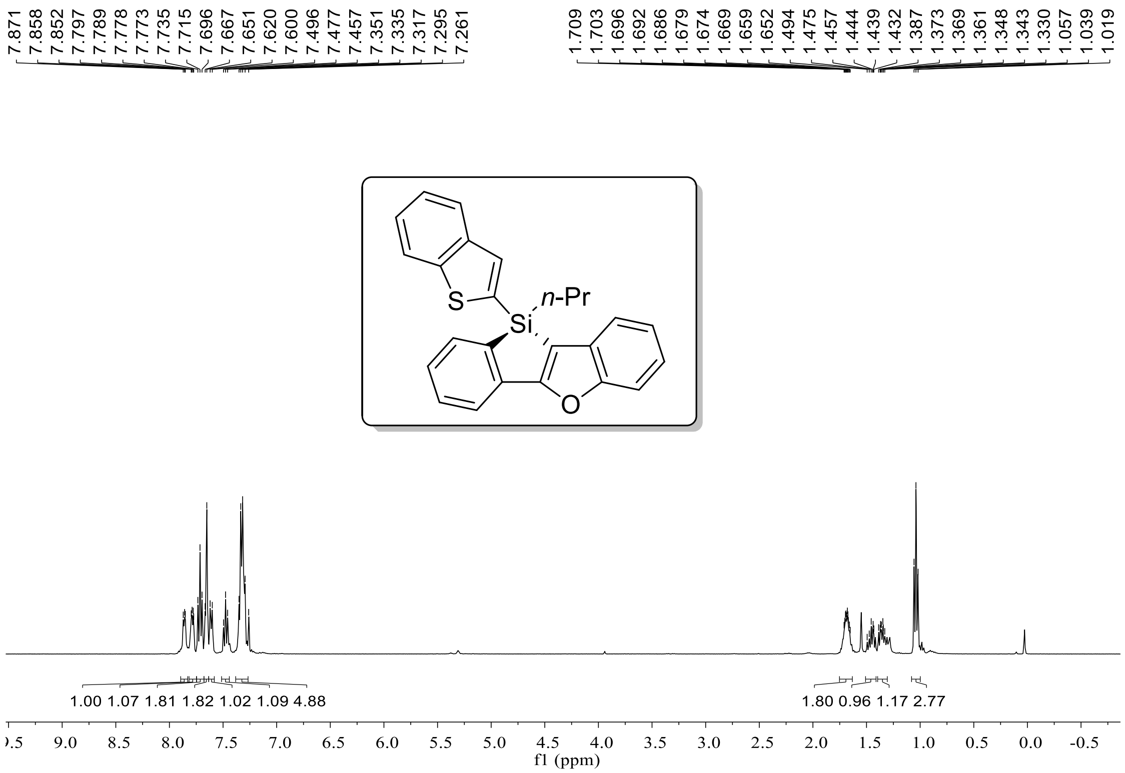

Supplementary Figure 324. ^{1}H NMR spectrum of **3vn**

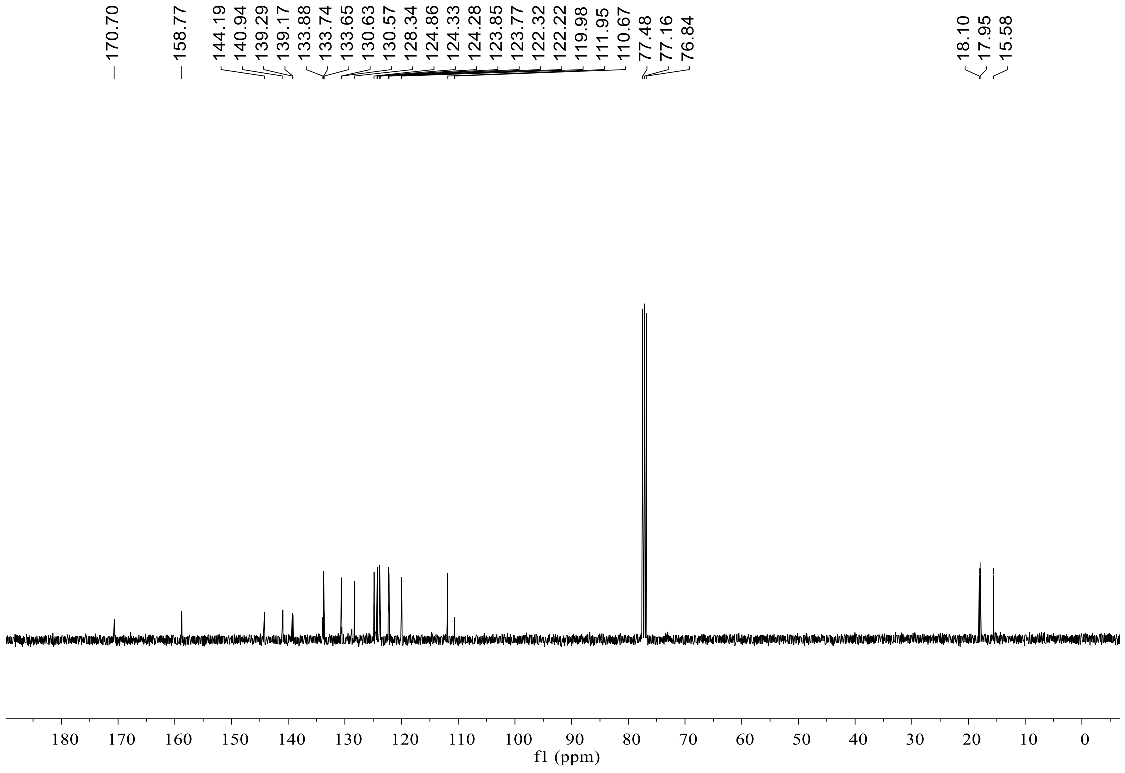

Supplementary Figure 325. ^{13}C NMR spectrum of **3vn**

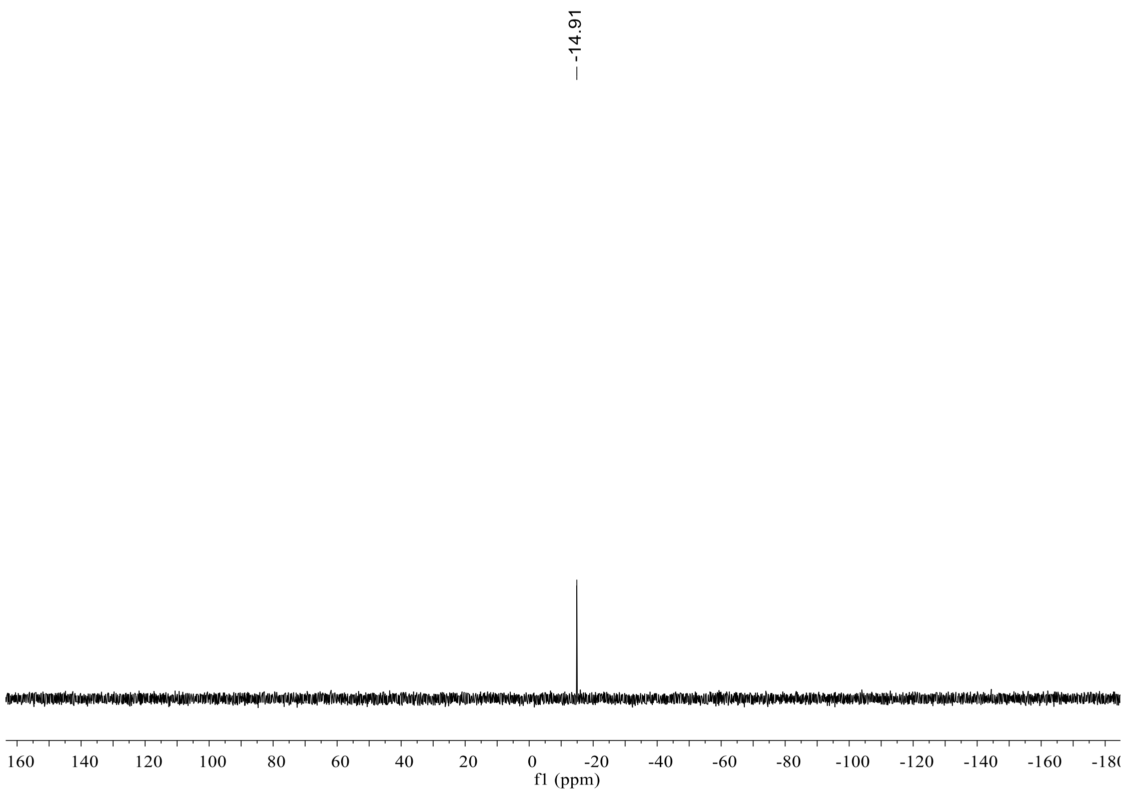

Supplementary Figure 326. ^{29}Si NMR spectrum of **3vn**

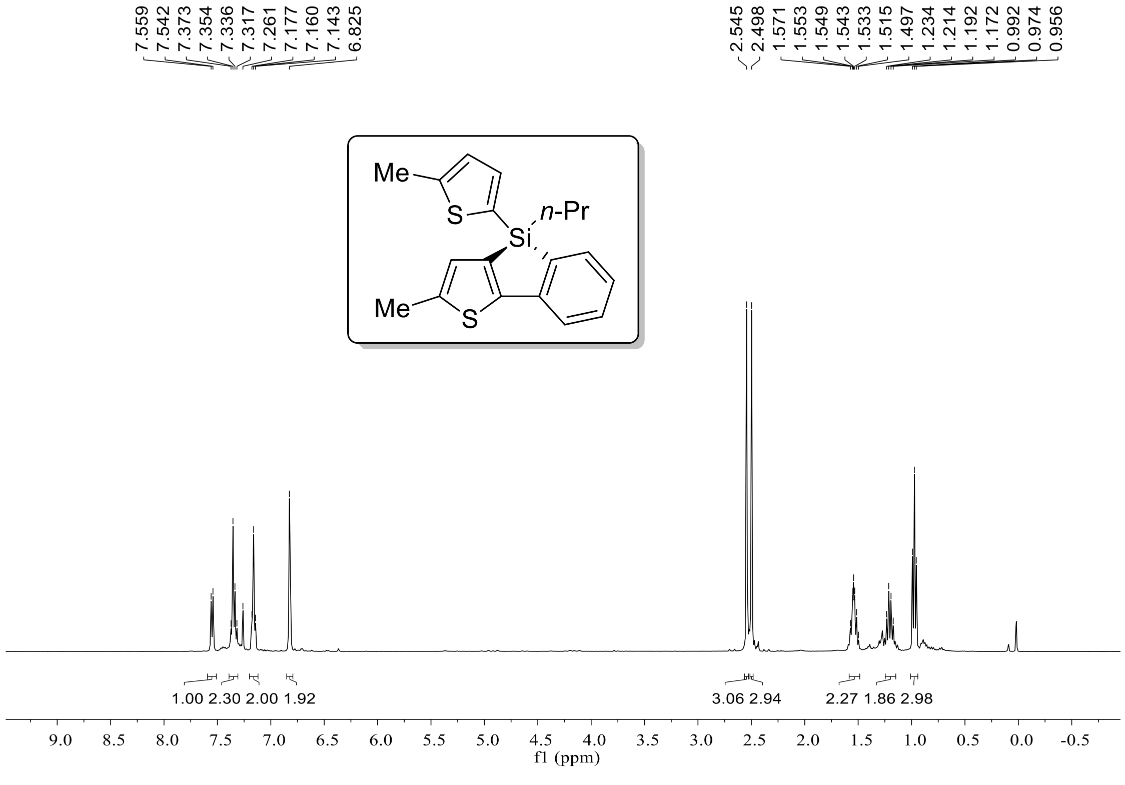

Supplementary Figure 327. ^{1}H NMR spectrum of **3wa**

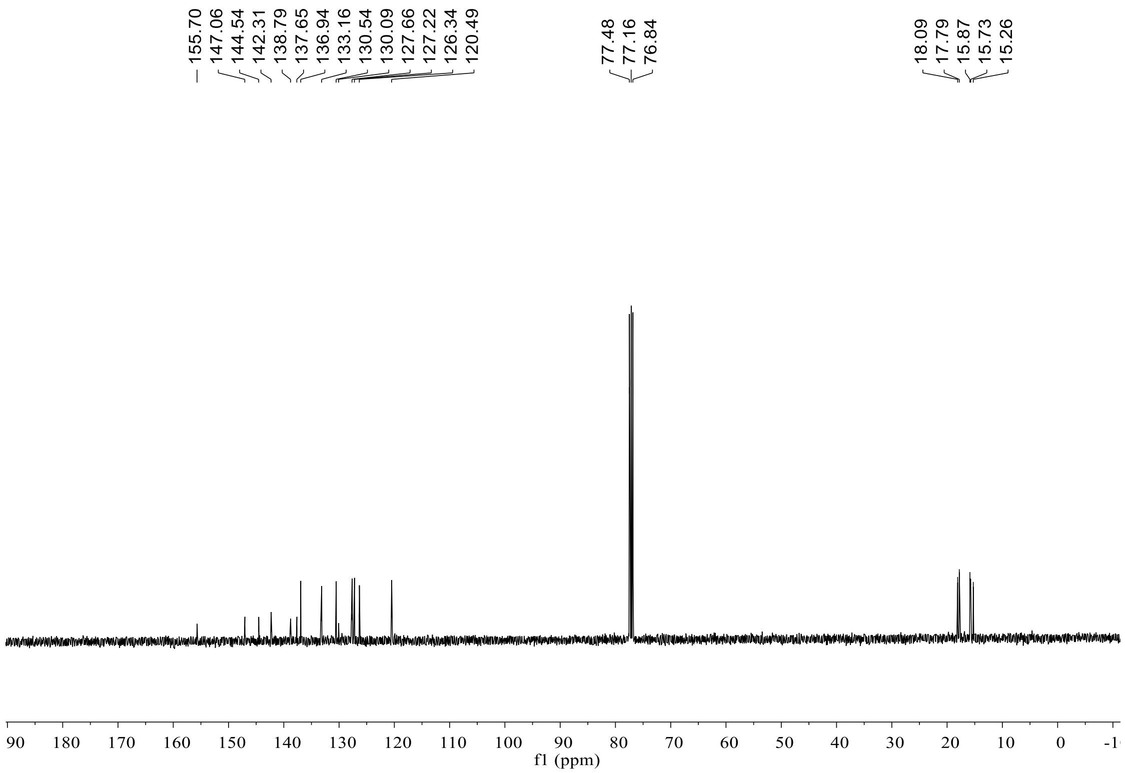

Supplementary Figure 328. ^{13}C NMR spectrum of **3wa**

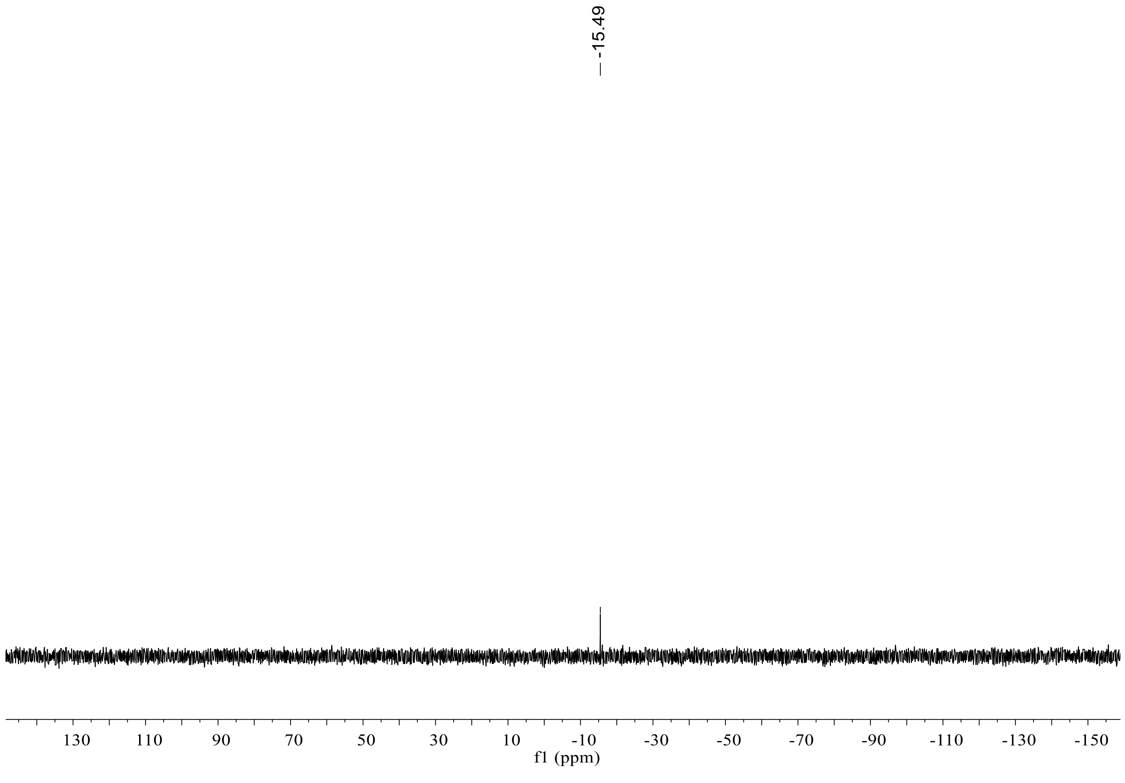

Supplementary Figure 329. ^{29}Si NMR spectrum of **3wa**

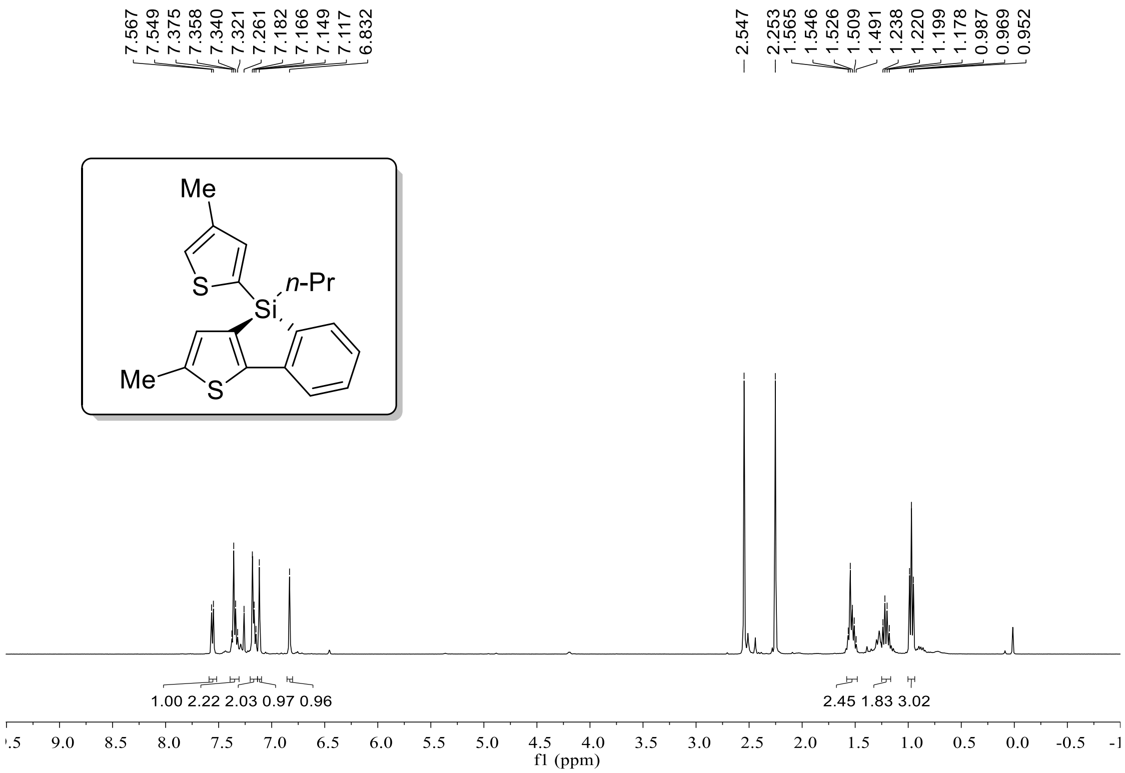

Supplementary Figure 330. ^{1}H NMR spectrum of **3wi**

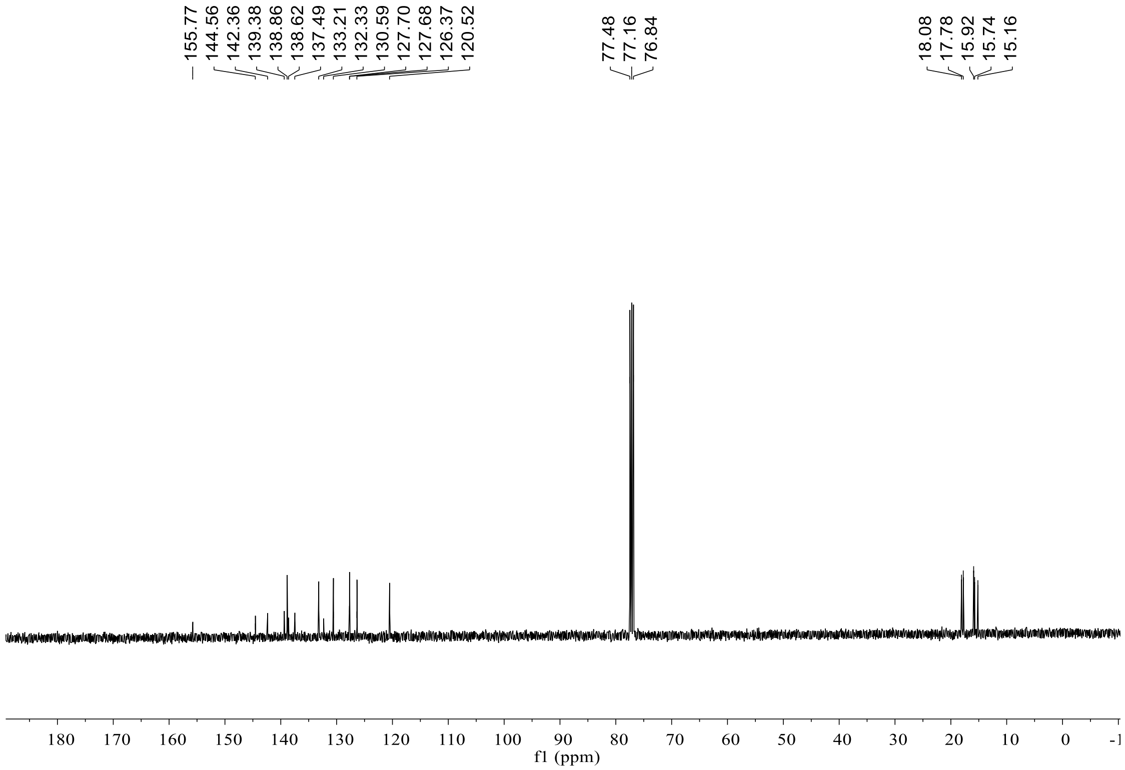

Supplementary Figure 331. ^{13}C NMR spectrum of **3wi**

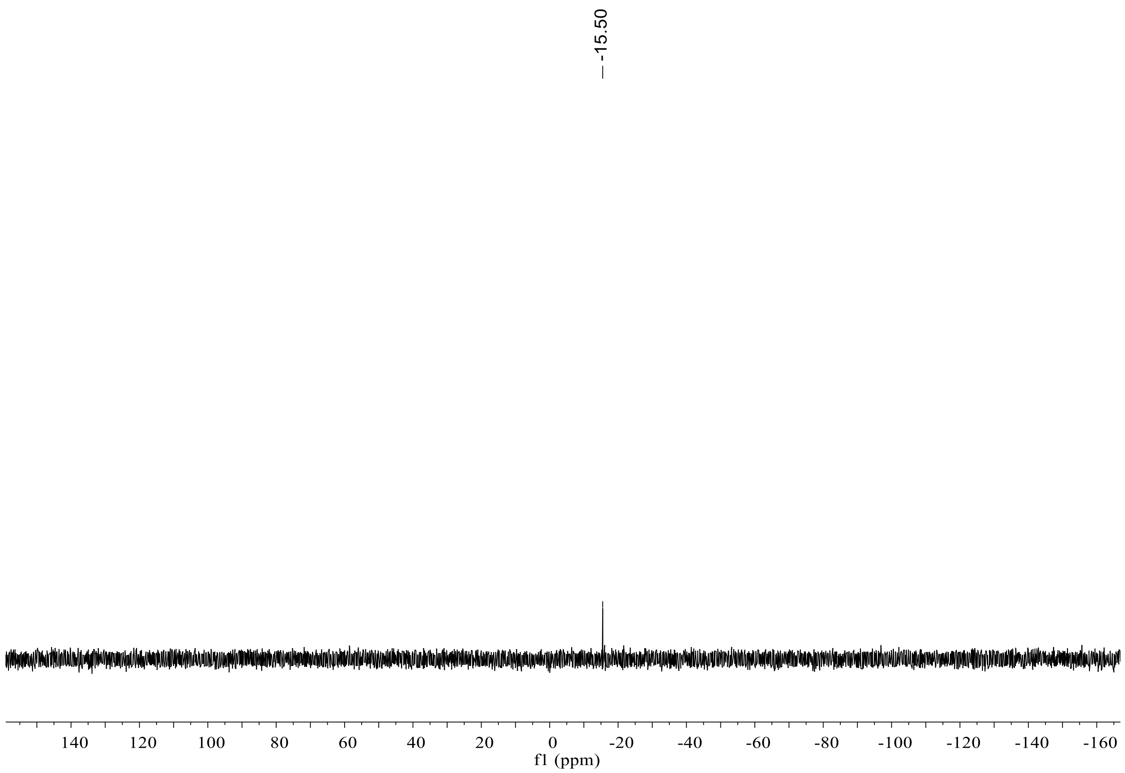

Supplementary Figure 332. ^{29}Si NMR spectrum of **3wi**

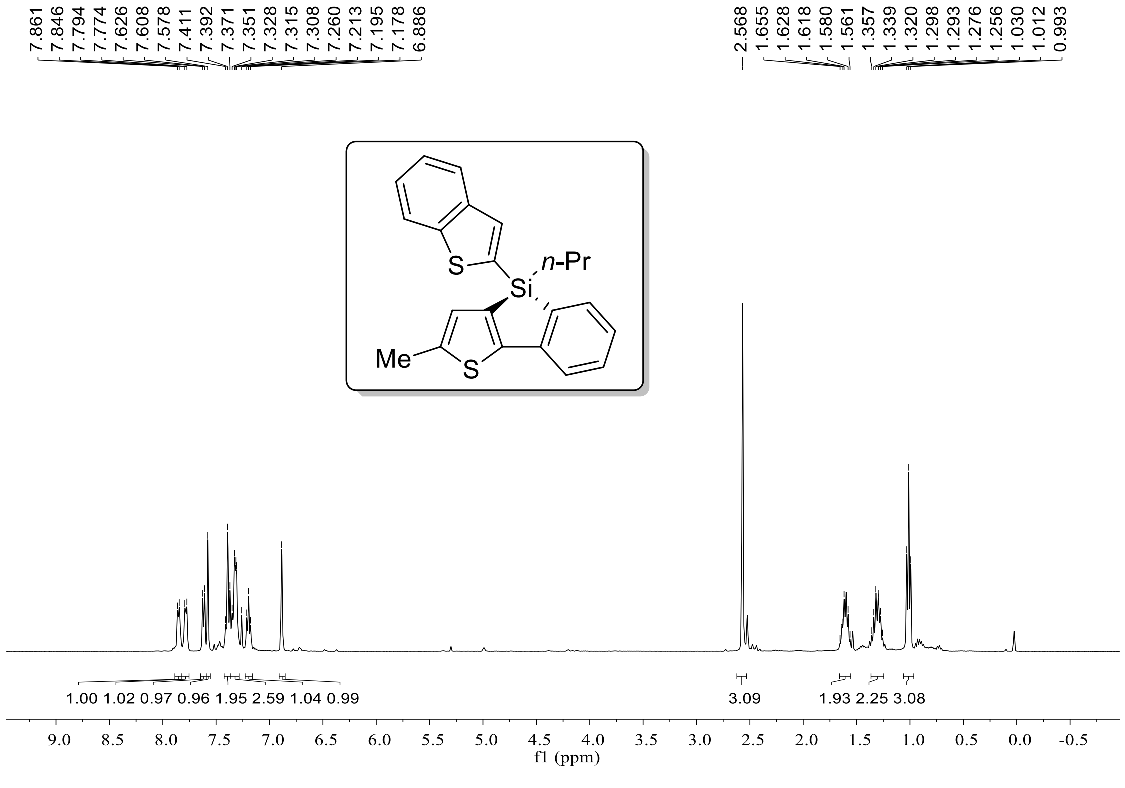

Supplementary Figure 333. ^{1}H NMR spectrum of **3wn**

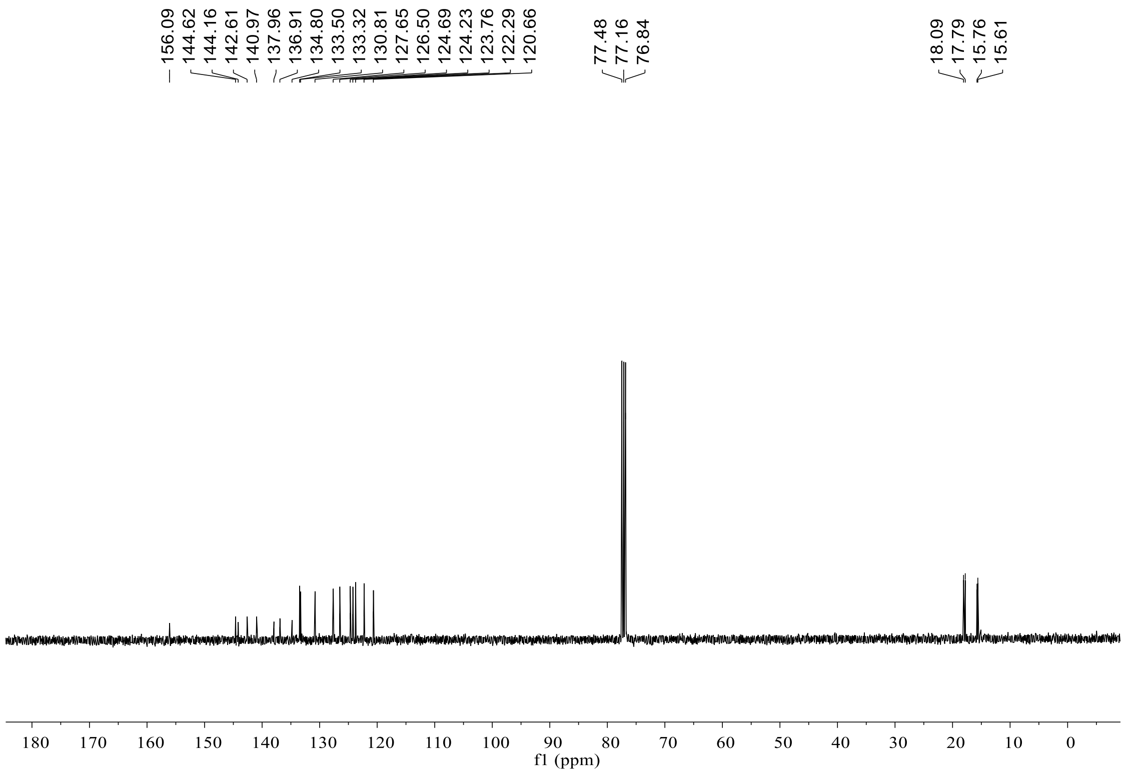

Supplementary Figure 334. ^{13}C NMR spectrum of **3wn**

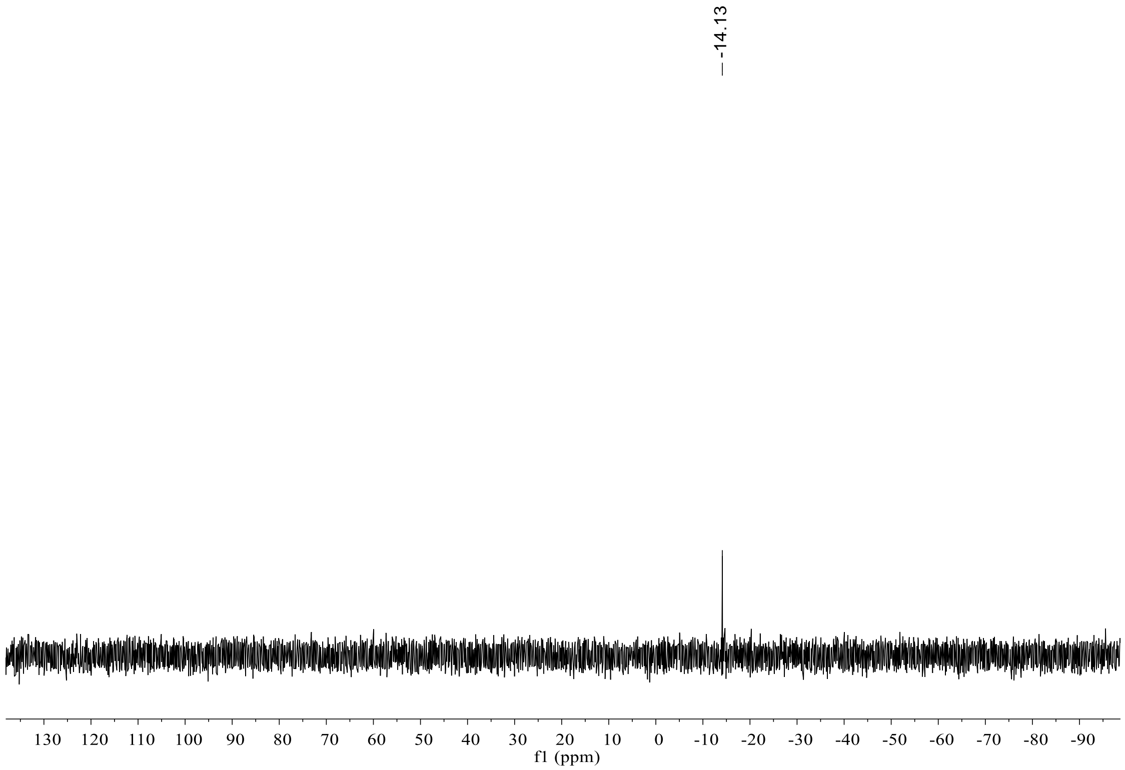

Supplementary Figure 335. ^{29}Si NMR spectrum of **3wn**

3.7 Copies of HPLC Spectra

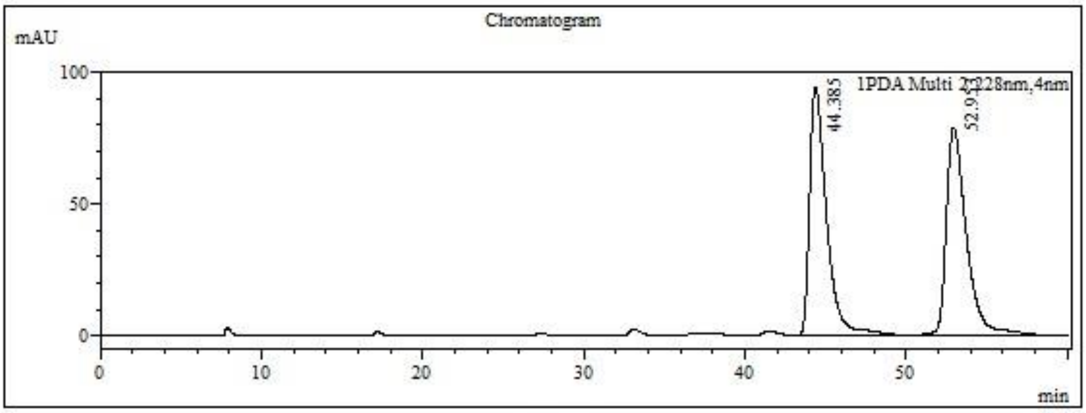

Peak Table

PDA Ch2 228nm

Peak#	Ret. Time	Area	Area%
1	44.385	6868876	50.305
2	52.953	6785597	49.695
总计		13654473	100.000

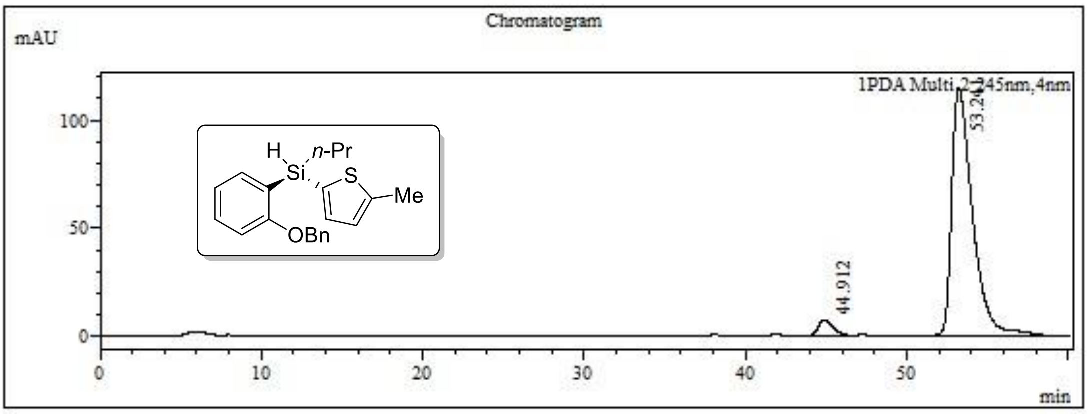

Peak Table

PDA Ch2 245nm

Peak#	Ret. Time	Area	Area%
1	44.912	452520	4.216
2	53.241	10281643	95.784
总计		10734163	100.000

Supplementary Figure 336. HPLC trace of **3aa**

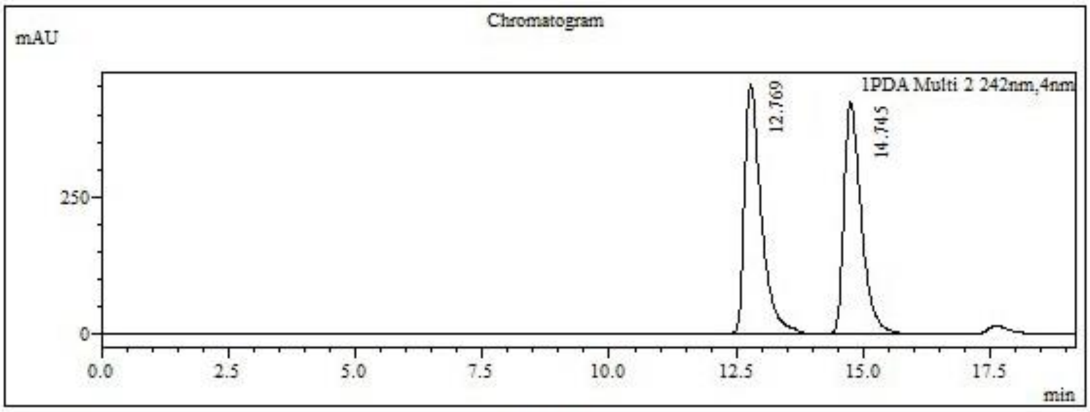

Peak Table

PDA Ch2 242nm

Peak#	Ret. Time	Area	Area%
1	12.769	7783709	50.720
2	14.745	7562600	49.280
总计		15346309	100.000

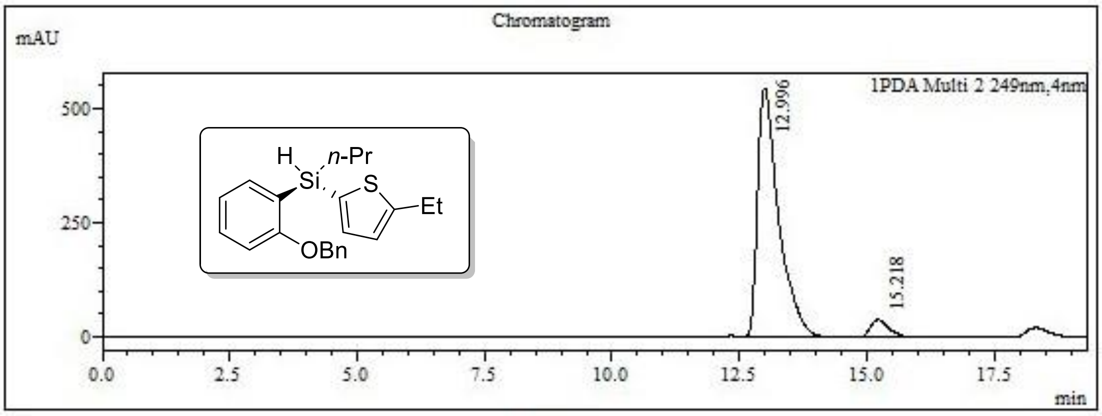

Peak Table

PDA Ch2 249nm

Peak#	Ret. Time	Area	Area%
1	12.996	15652539	94.864
2	15.218	847354	5.136
总计		16499893	100.000

Supplementary Figure 337. HPLC trace of **3ab**

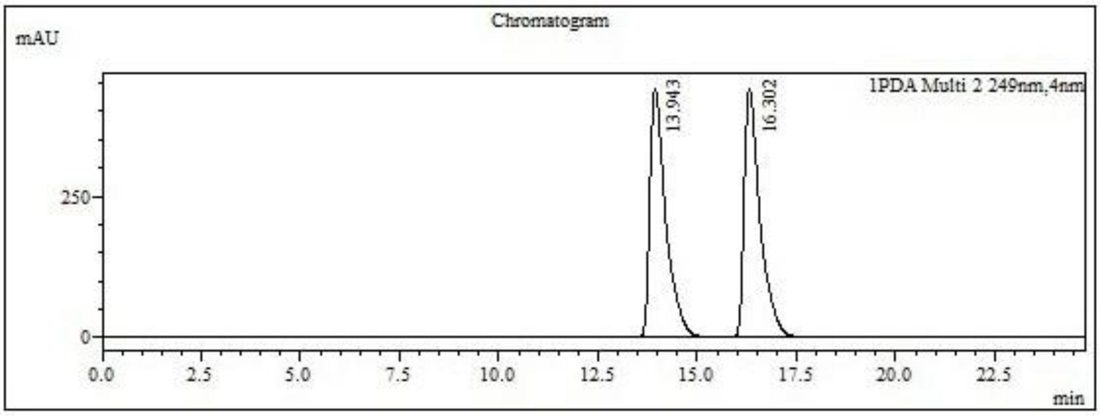

Peak Table

PDA Ch2 249nm

Peak#	Ret. Time	Area	Area%
1	13.943	6665925	49.161
2	16.302	6893408	50.839
总计		13559333	100.000

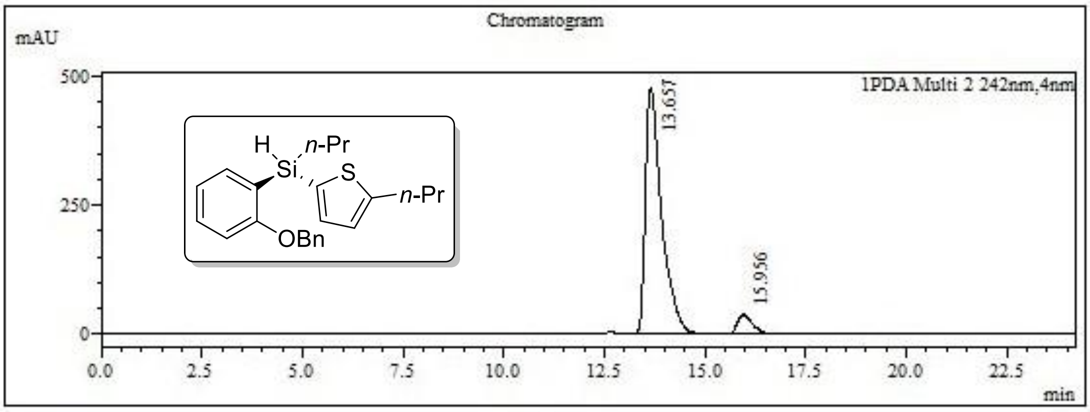

Peak Table

PDA Ch2 242nm

Peak#	Ret. Time	Area	Area%
1	13.657	13439912	94.156
2	15.956	834220	5.844
总计		14274132	100.000

Supplementary Figure 338. HPLC trace of **3ac**

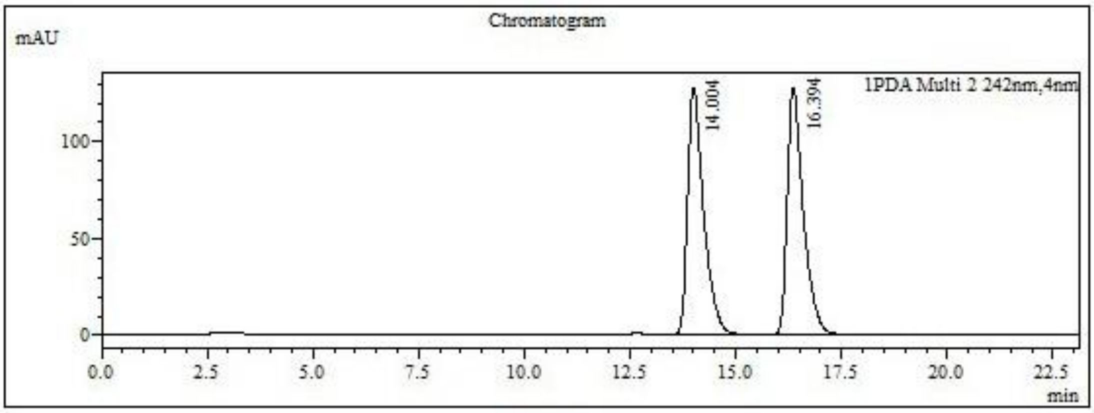

Peak Table

PDA Ch2 242nm

Peak#	Ret. Time	Area	Area%
1	14.004	2634097	49.479
2	16.394	2689574	50.521
总计		5323671	100.000

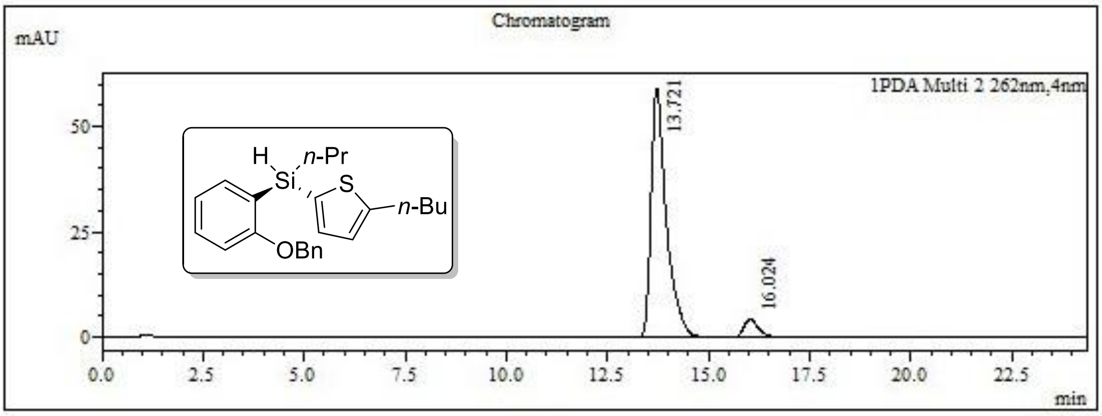

Peak Table

PDA Ch2 262nm

Peak#	Ret. Time	Area	Area%
1	13.721	1561357	94.073
2	16.024	98378	5.927
总计		1659734	100.000

Supplementary Figure 339. HPLC trace of **3ad**

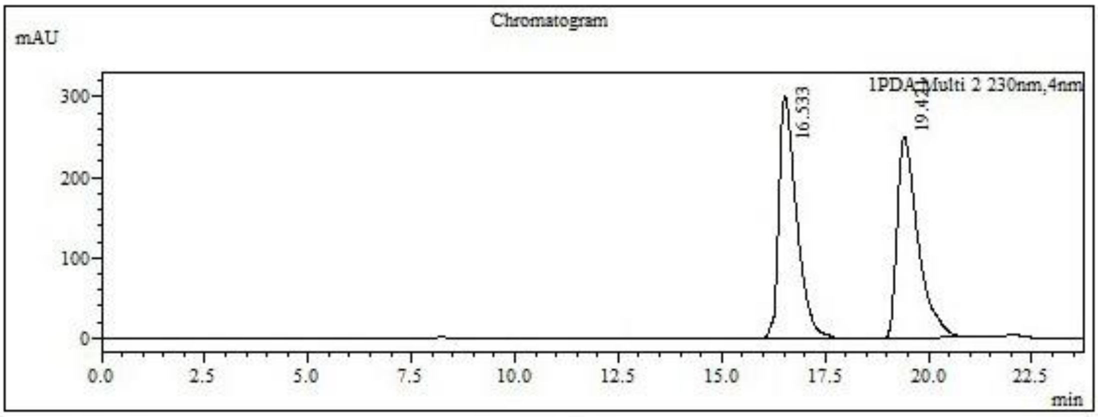

Peak Table

PDA Ch2 230nm

Peak#	Ret. Time	Area	Area%
1	16.533	9902934	49.935
2	19.421	9928531	50.065
总计		19831466	100.000

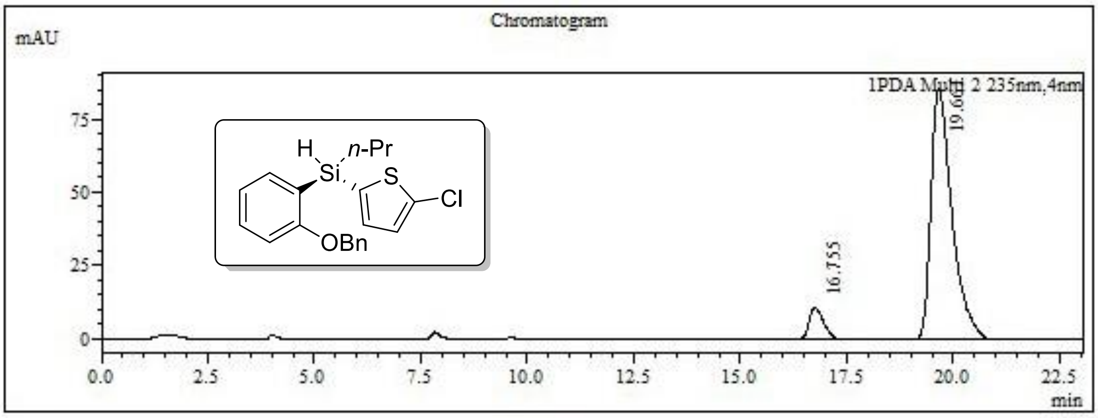

Peak Table

PDA Ch2 235nm

Peak#	Ret. Time	Area	Area%
1	16.755	321021	9.318
2	19.661	3124304	90.682
总计		3445325	100.000

Supplementary Figure 340. HPLC trace of **3ae**

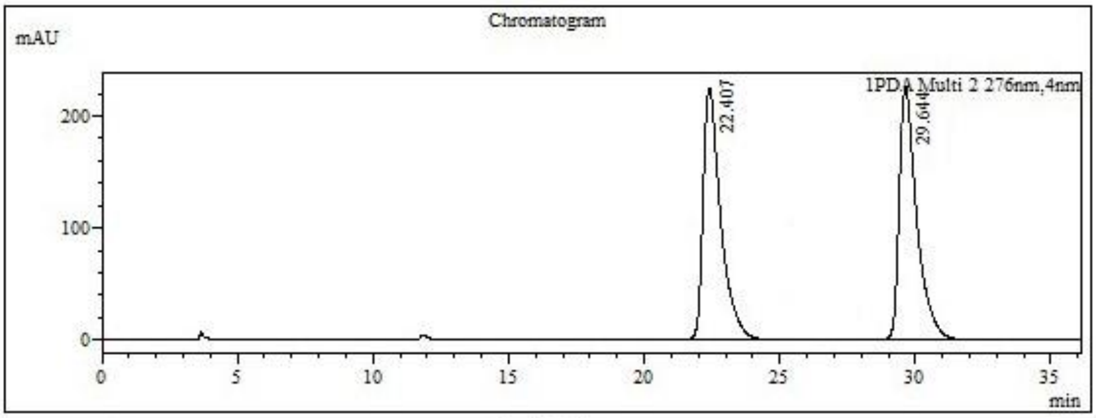

Peak Table

PDA Ch2 276nm

Peak#	Ret. Time	Area	Area%
1	22.407	2908061	49.393
2	29.644	2979479	50.607
总计		5887540	100.000

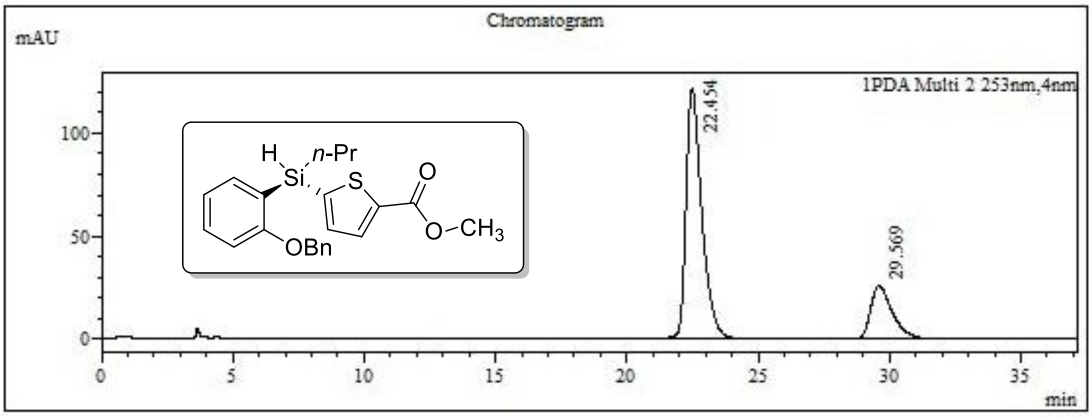

Peak Table

PDA Ch2 253nm

Peak#	Ret. Time	Area	Area%
1	22.454	5094620	77.845
2	29.569	1449971	22.155
总计		6544591	100.000

Supplementary Figure 341. HPLC trace of **3af**

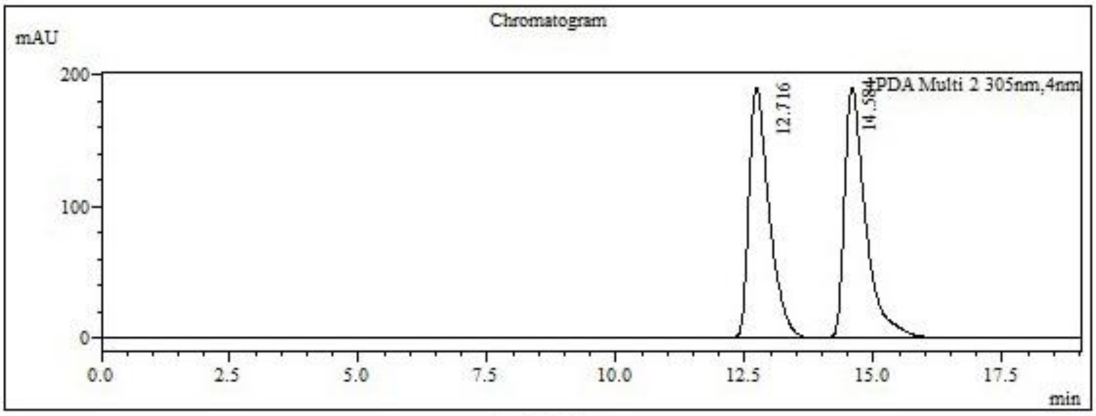

Peak Table

PDA Ch2 305nm

Peak#	Ret. Time	Area	Area%
1	12.716	1610621	49.922
2	14.584	1615646	50.078
总计		3226267	100.000

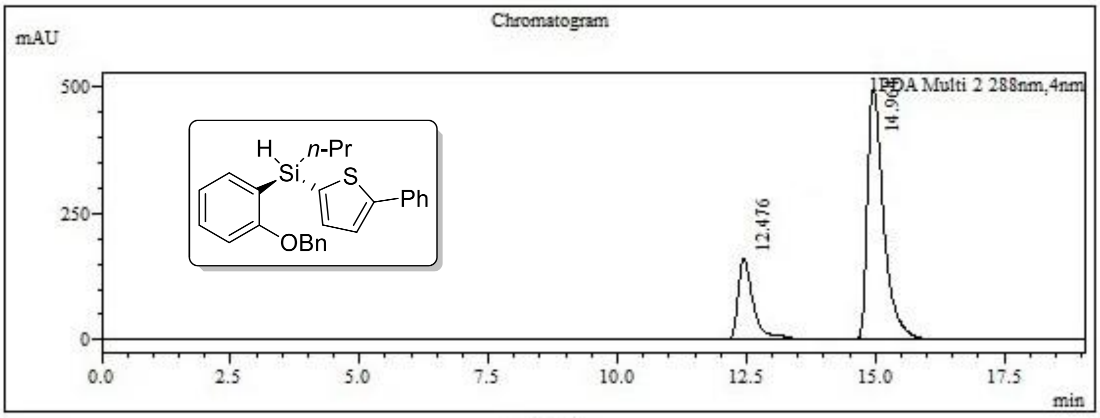

Peak Table

PDA Ch2 288nm

Peak#	Ret. Time	Area	Area%
1	12.476	3244403	23.119
2	14.964	10788987	76.881
总计		14033390	100.000

Supplementary Figure 342. HPLC trace of **3ag**

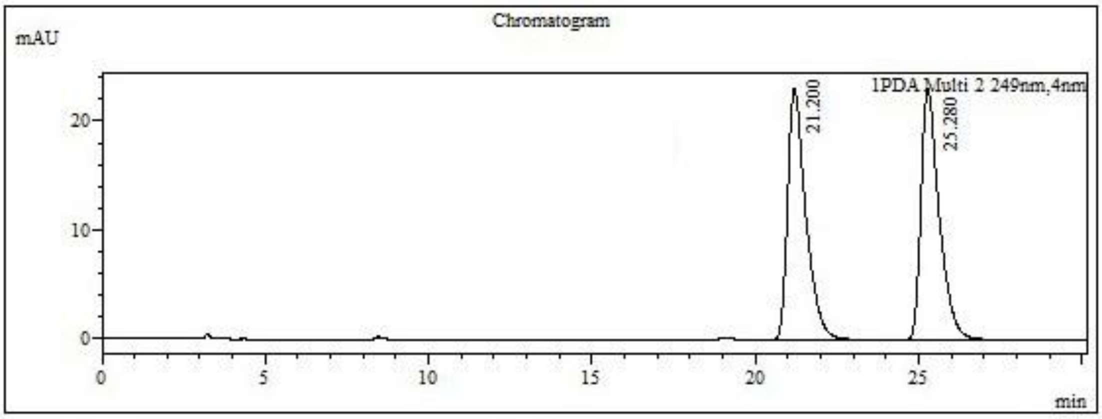

Peak Table

PDA Ch2 249nm

Peak#	Ret. Time	Area	Area%
1	21.200	1184882	50.582
2	25.280	1157614	49.418
总计		2342496	100.000

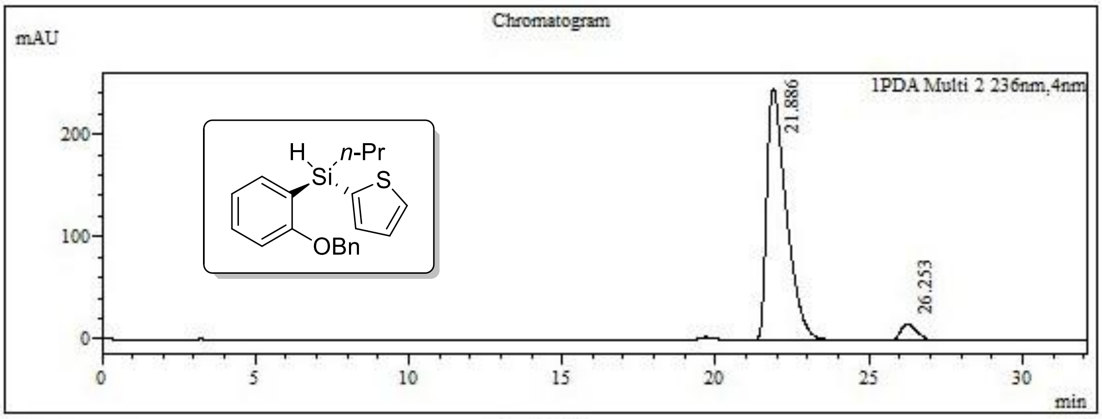

Peak Table

PDA Ch2 236nm

Peak#	Ret. Time	Area	Area%
1	21.886	10897993	94.875
2	26.253	588722	5.125
总计		11486715	100.000

Supplementary Figure 343. HPLC trace of **3ah**

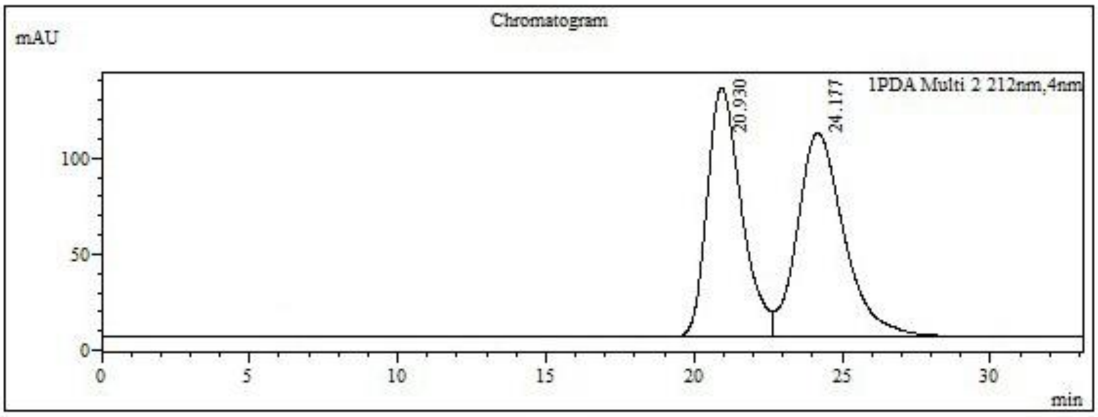

Peak Table

PDA Ch2 212nm

Peak#	Ret. Time	Area	Area%
1	20.930	10753379	50.785
2	24.177	10420905	49.215
总计		21174284	100.000

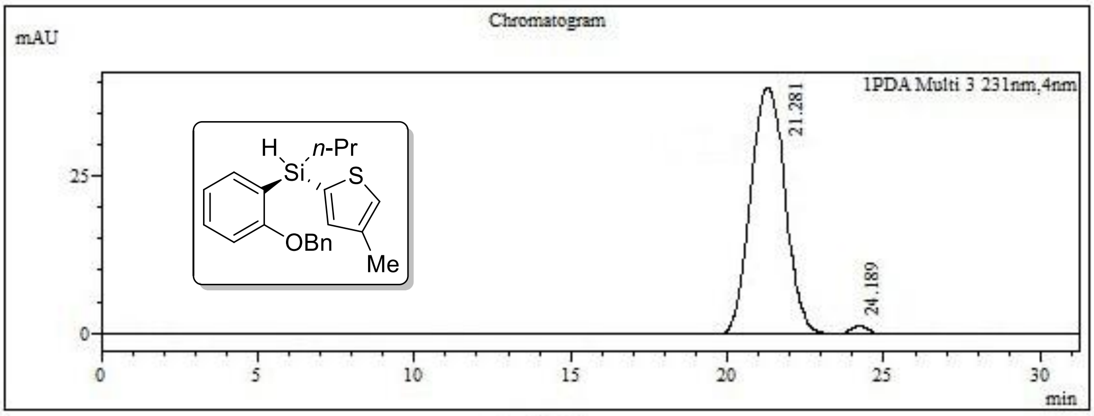

Peak Table

PDA Ch3 231nm

Peak#	Ret. Time	Area	Area%
1	21.281	3598822	94.774
2	24.189	198452	5.226
总计		3797275	100.000

Supplementary Figure 344. HPLC trace of **3ai**

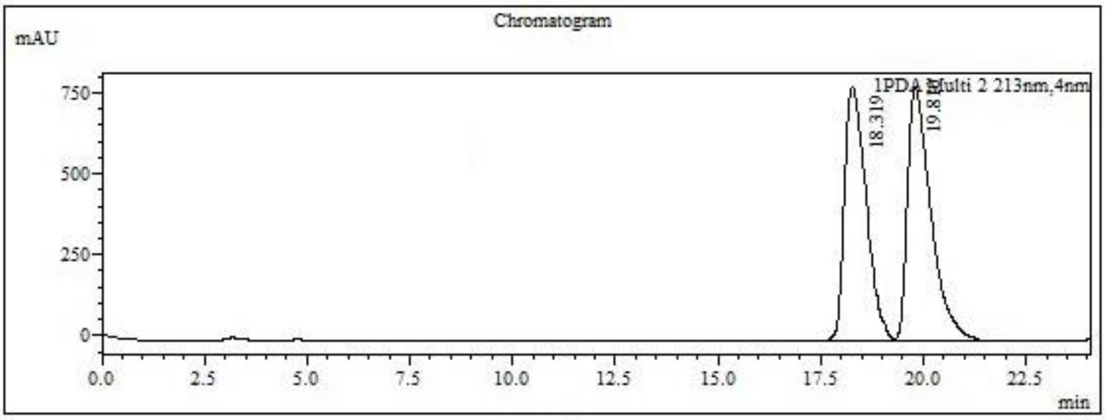

Peak Table

PDA Ch2 213nm

Peak#	Ret. Time	Area	Area%
1	18.319	16801947	49.473
2	19.810	17160104	50.527
总计		33962051	100.000

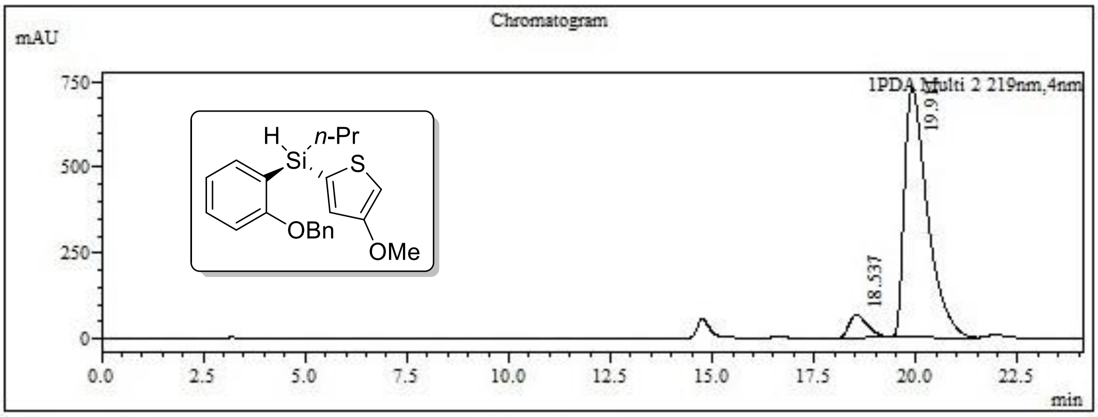

Peak Table

PDA Ch2 219nm

Peak#	Ret. Time	Area	Area%
1	18.537	2089534	6.789
2	19.911	28687101	93.211
总计		30776635	100.000

Supplementary Figure 345. HPLC trace of **3aj**

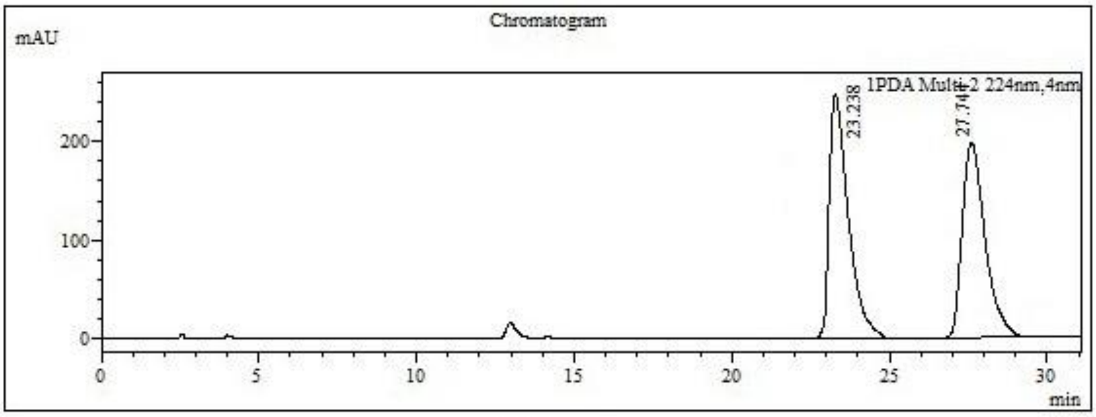

Peak Table

PDA Ch2 224nm

Peak#	Ret. Time	Area	Area%
1	23.238	10133460	50.351
2	27.744	9992293	49.649
总计		20125753	100.000

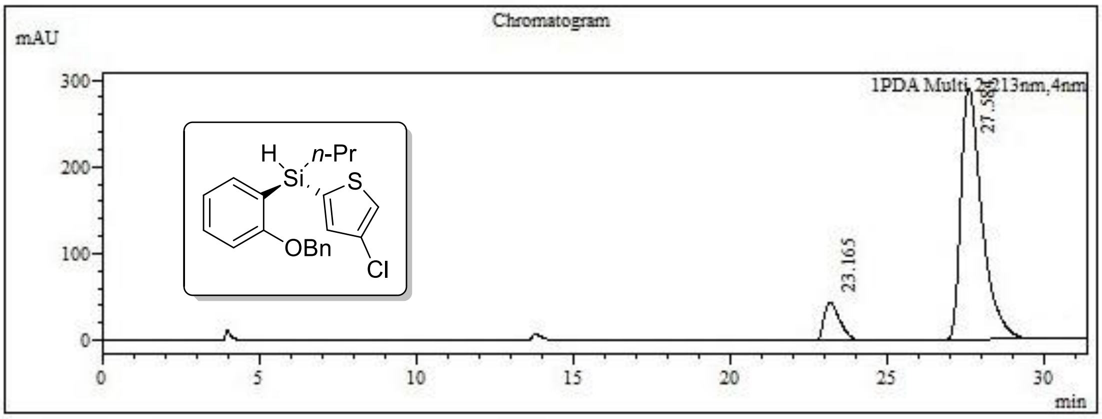

Peak Table

PDA Ch2 213nm

Peak#	Ret. Time	Area	Area%
1	23.165	1541514	10.220
2	27.584	13542216	89.780
总计		15083730	100.000

Supplementary Figure 346. HPLC trace of **3ak**

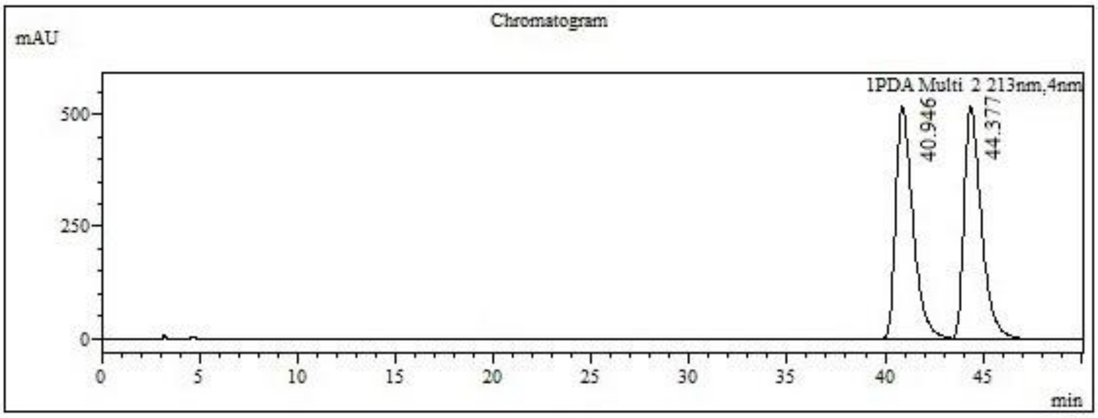

Peak Table

PDA Ch2 213nm

Peak#	Ret. Time	Area	Area%
1	40.946	7783709	50.720
2	44.377	7562600	49.280
总计		15346309	100.000

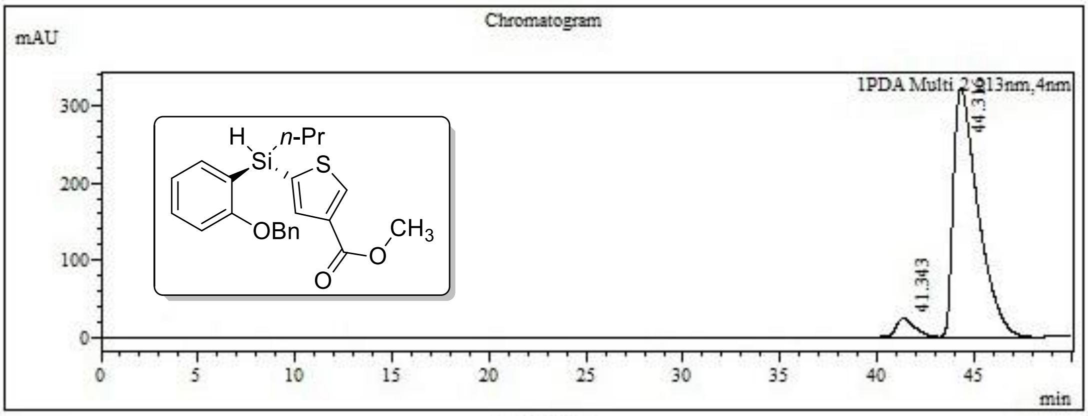

Peak Table

PDA Ch2 213nm

Peak#	Ret. Time	Area	Area%
1	41.343	1618014	5.338
2	44.316	28692823	94.662
总计		30310837	100.000

Supplementary Figure 347. HPLC trace of **3al**

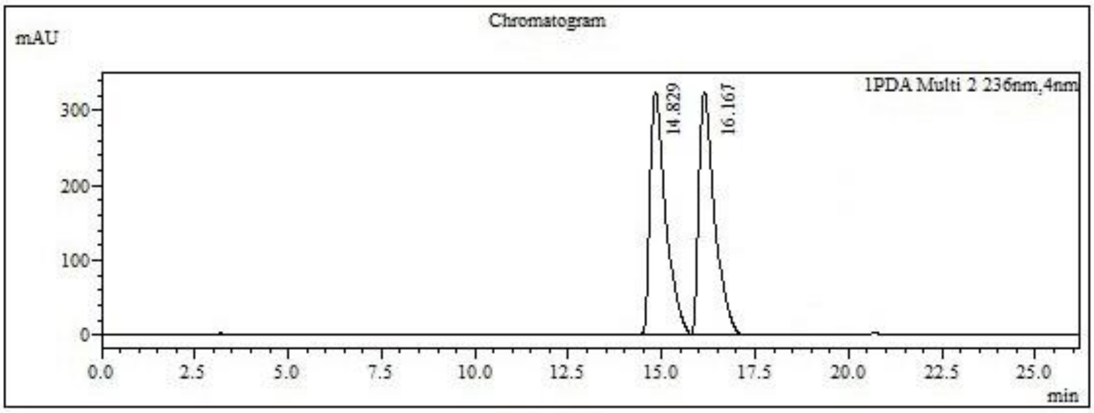

Peak Table

PDA Ch2 236nm

Peak#	Ret. Time	Area	Area%
1	14.829	3730701	50.065
2	16.167	3721038	49.935
总计		7451739	100.000

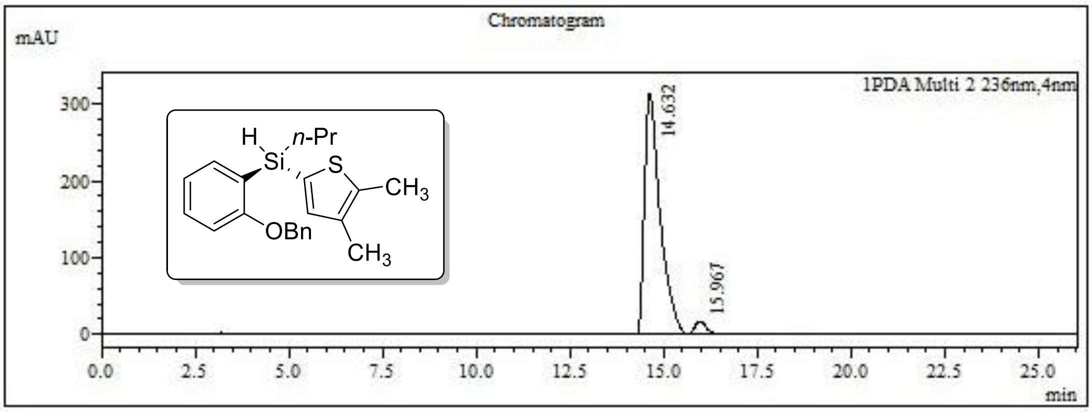

Peak Table

PDA Ch2 236nm

Peak#	Ret. Time	Area	Area%
1	14.632	9245723	95.240
2	15.967	462079	4.760
总计		9707802	100.000

Supplementary Figure 348. HPLC trace of **3am**

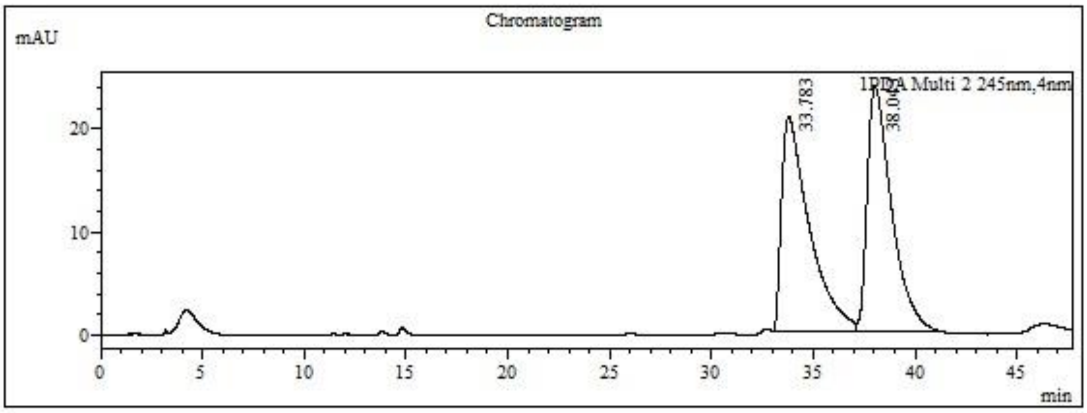

Peak Table

PDA Ch2 245nm

Peak#	Ret. Time	Area	Area%
1	33.783	1967213	49.660
2	38.043	1994176	50.340
总计		3961389	100.000

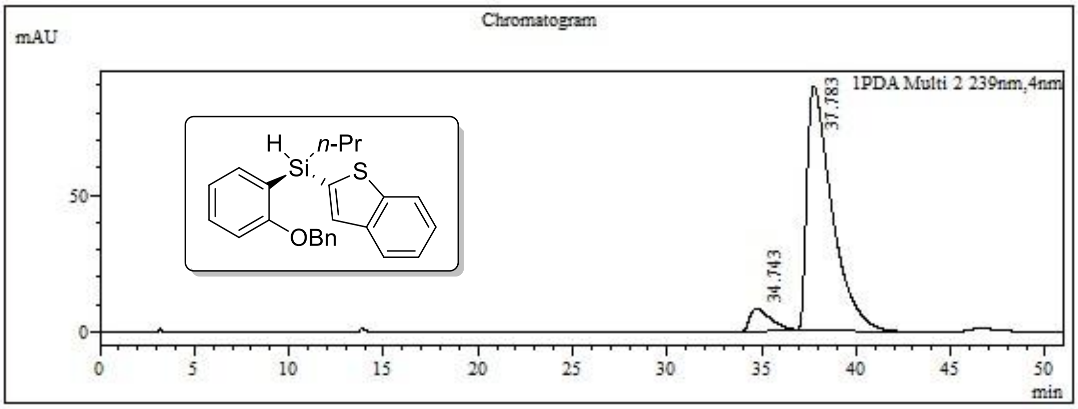

Peak Table

PDA Ch2 239nm

Peak#	Ret. Time	Area	Area%
1	34.743	629275	7.139
2	37.783	8185589	92.861
总计		8814863	100.000

Supplementary Figure 349. HPLC trace of **3an**

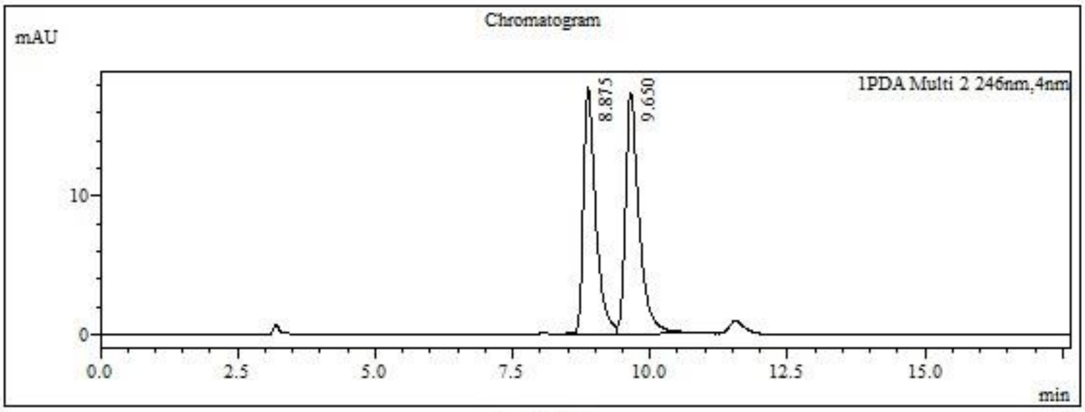

Peak Table

PDA Ch2 246nm

Peak#	Ret. Time	Area	Area%
1	8.875	260394	49.135
2	9.650	269566	50.865
总计		529960	100.000

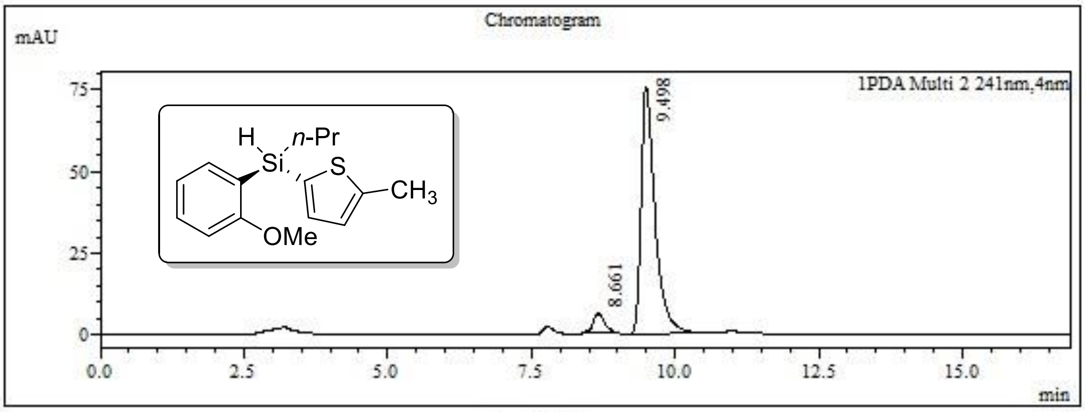

Peak Table

PDA Ch2 241nm

Peak#	Ret. Time	Area	Area%
1	8.661	83283	6.064
2	9.498	1290189	93.936
总计		1373472	100.000

Supplementary Figure 350. HPLC trace of **3ba**

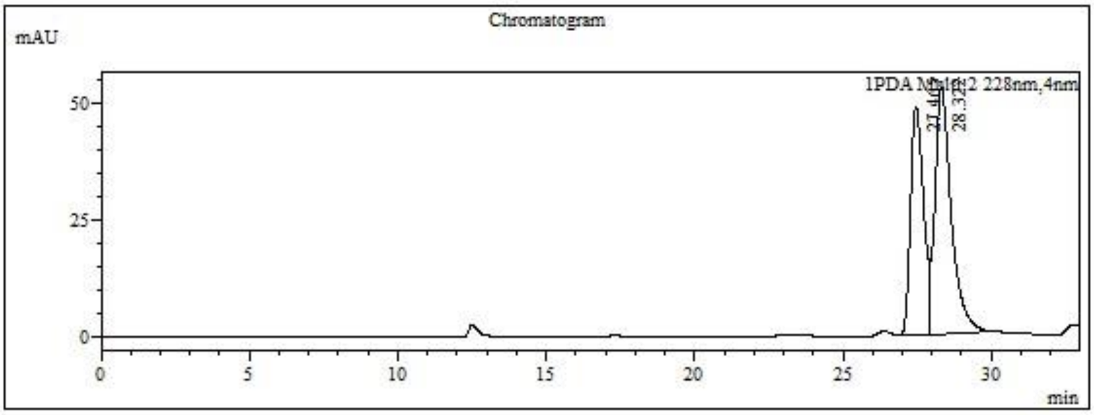

Peak Table

PDA Ch2 228nm

Peak#	Ret. Time	Area	Area%
1	27.465	1758240	49.834
2	28.322	1769927	50.166
总计		3528167	100.000

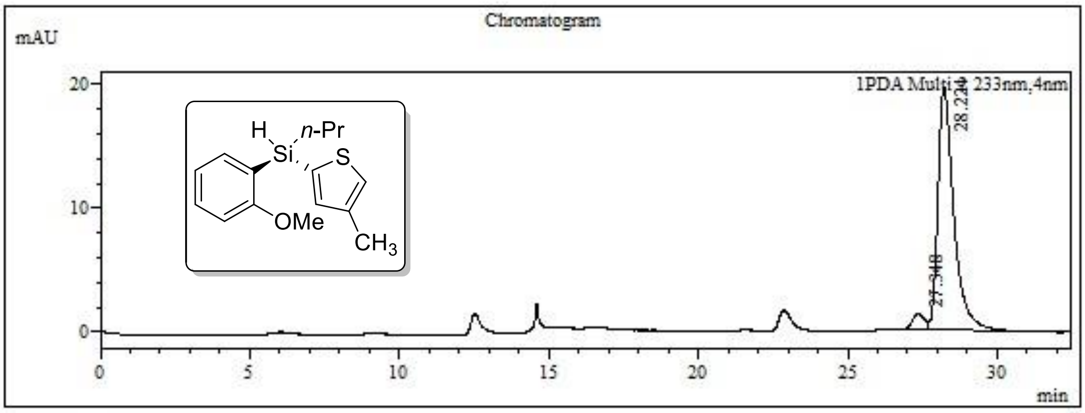

Peak Table

PDA Ch2 233nm

Peak#	Ret. Time	Area	Area%
1	27.348	35820	4.703
2	28.224	725751	95.297
总计		761571	100.000

Supplementary Figure 351. HPLC trace of **3bi**

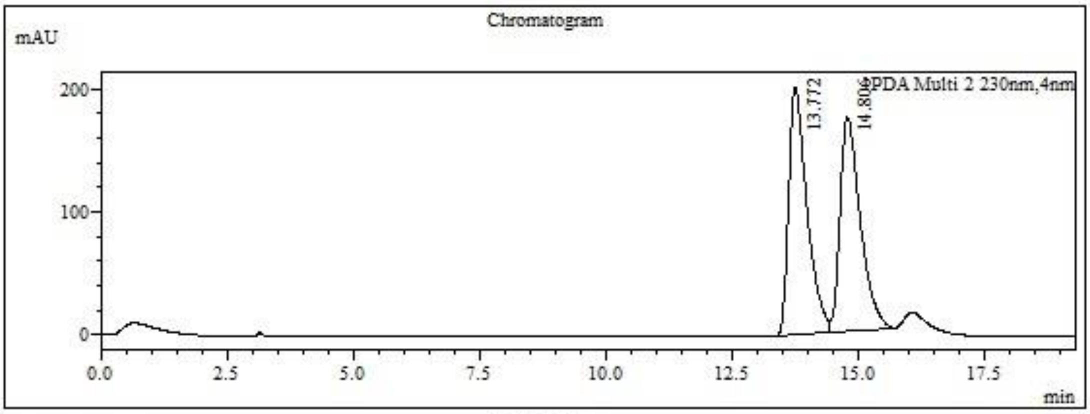

Peak Table

PDA Ch2 230nm

Peak#	Ret. Time	Area	Area%
1	13.772	5154201	49.412
2	14.806	5276932	50.588
总计		10431133	100.000

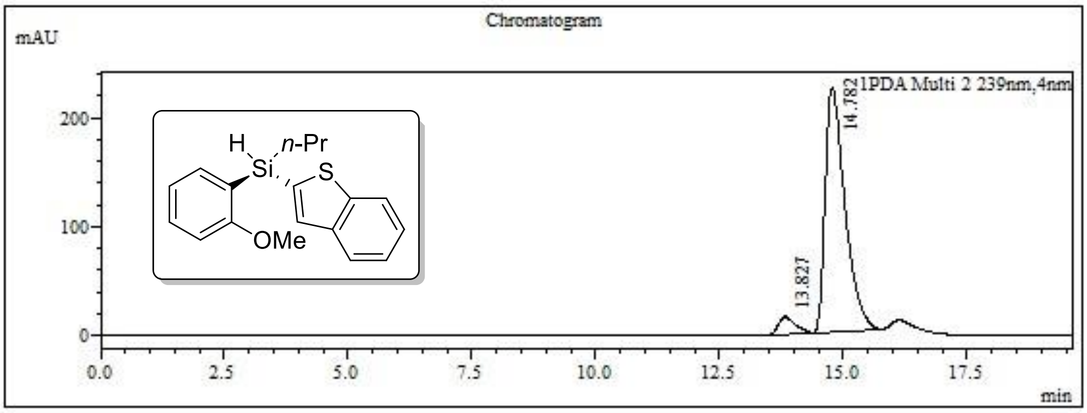

Peak Table

PDA Ch2 239nm

Peak#	Ret. Time	Area	Area%
1	13.827	383640	5.788
2	14.782	6244981	94.212
总计		6628622	100.000

Supplementary Figure 352. HPLC trace of **3bn**

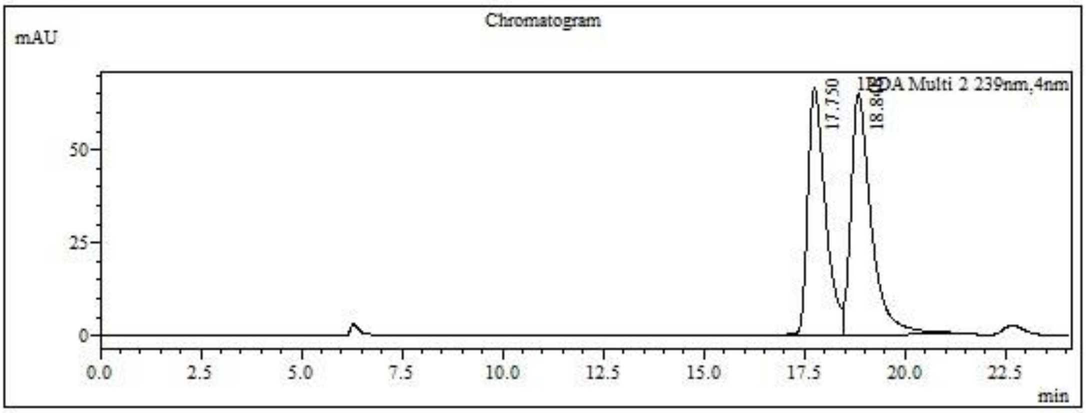

Peak Table

PDA Ch2 239nm

Peak#	Ret. Time	Area	Area%
1	17.750	1968208	49.169
2	18.846	2034705	50.831
总计		4002913	100.000

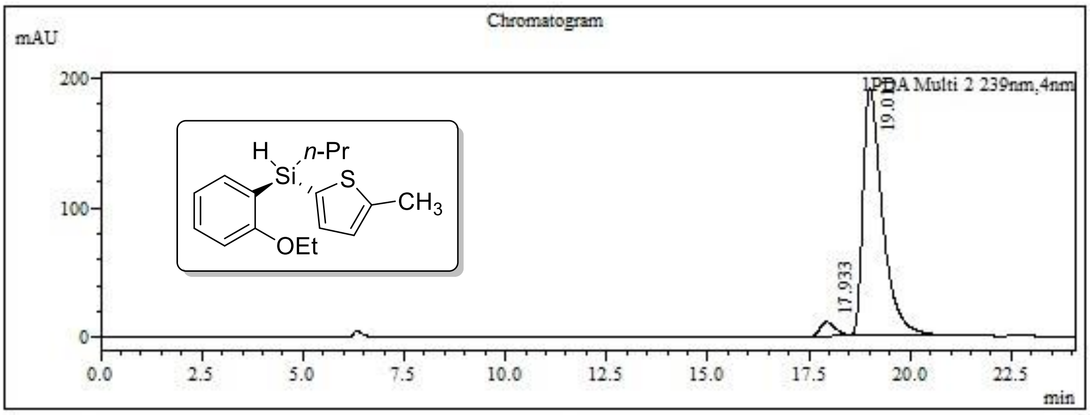

Peak Table

PDA Ch2 239nm

Peak#	Ret. Time	Area	Area%
1	17.933	273945	4.104
2	19.011	6401599	95.896
总计		6675544	100.000

Supplementary Figure 353. HPLC trace of **3ca**

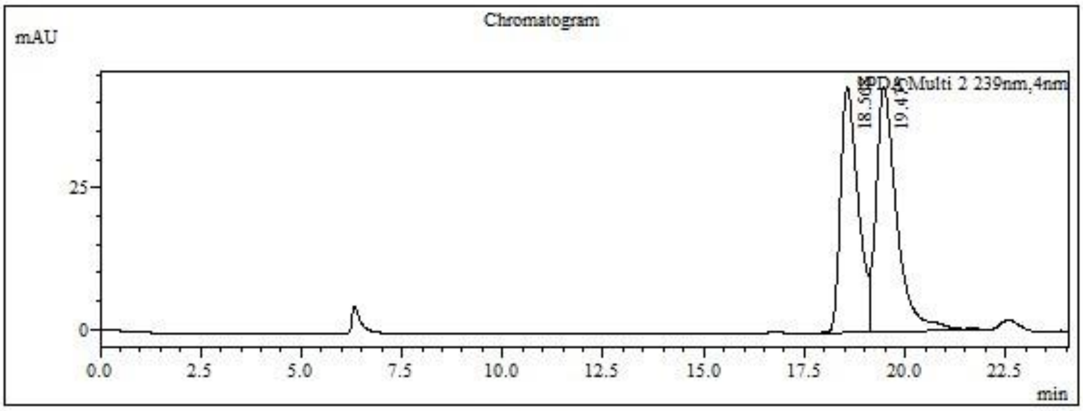

Peak Table

PDA Ch2 239nm

Peak#	Ret. Time	Area	Area%
1	18.568	1251631	49.672
2	19.475	1268173	50.328
总计		2519804	100.000

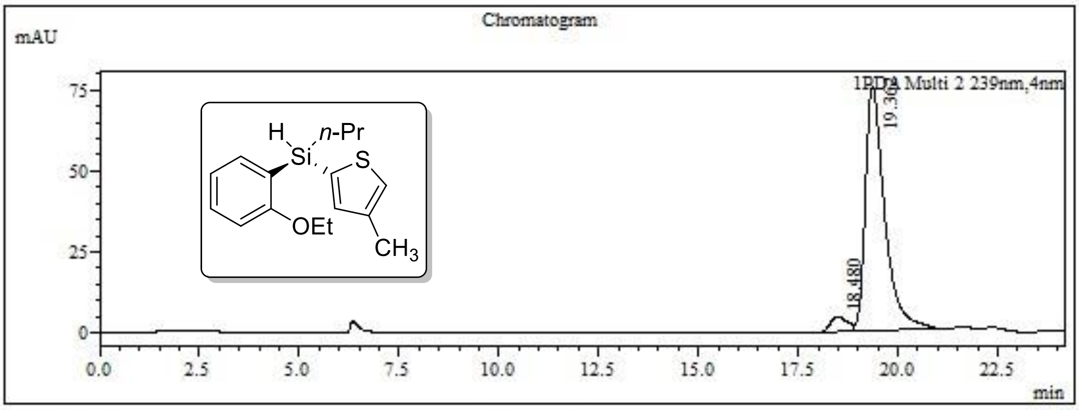

Peak Table

PDA Ch2 239nm

Peak#	Ret. Time	Area	Area%
1	18.480	134077	5.002
2	19.362	2546224	94.998
总计		2680301	100.000

Supplementary Figure 354. HPLC trace of **3ci**

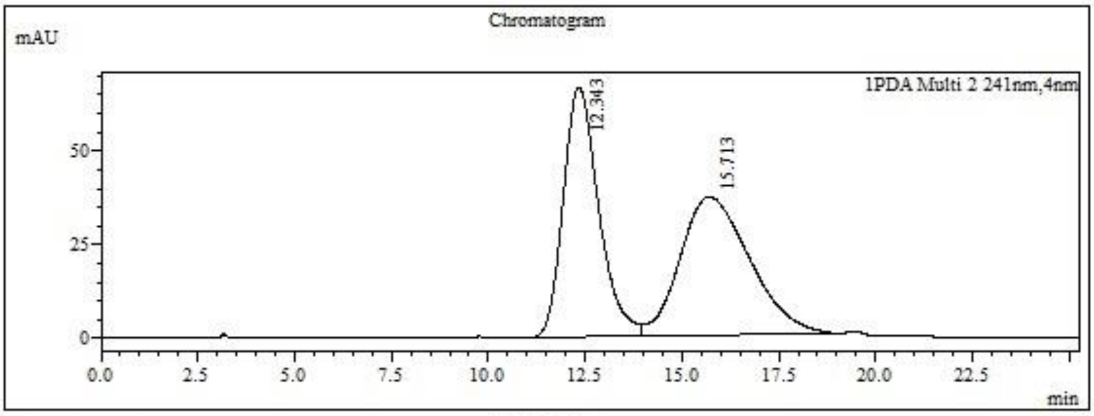

Peak Table

PDA Ch2 241nm

Peak#	Ret. Time	Area	Area%
1	12.343	4349611	49.595
2	15.713	4420629	50.405
总计		8770240	100.000

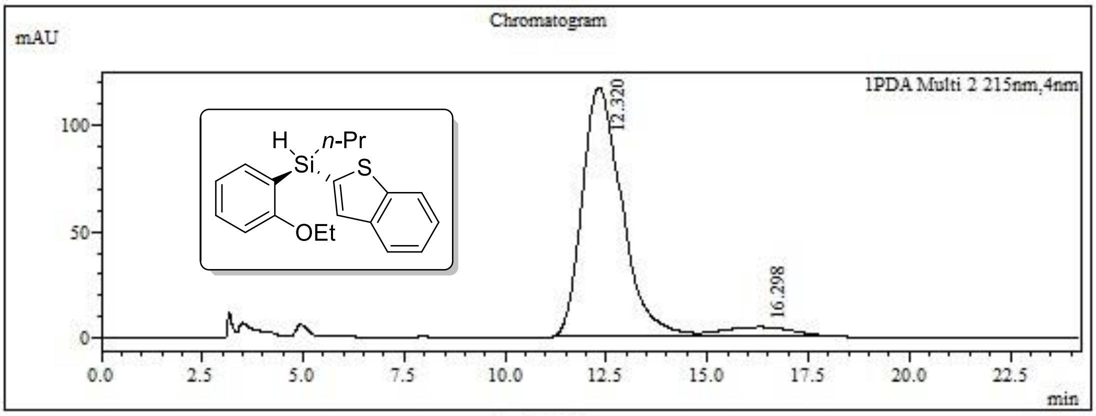

Peak Table

PDA Ch2 215nm

Peak#	Ret. Time	Area	Area%
1	12.320	7956224	94.481
2	16.298	464728	5.519
总计		8420951	100.000

Supplementary Figure 355. HPLC trace of **3cn**

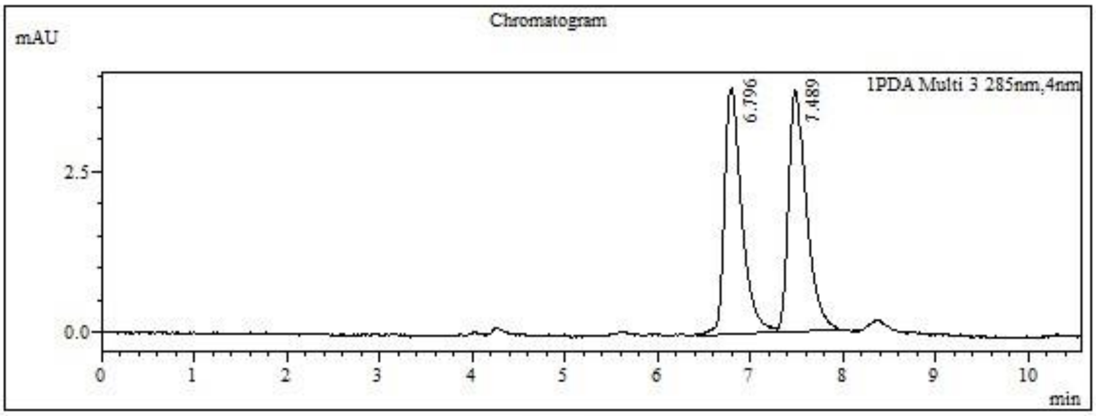

Peak Table

PDA Ch3 285nm

Peak#	Ret. Time	Area	Area%
1	6.796	48337	49.241
2	7.489	49826	50.759
总计		98163	100.000

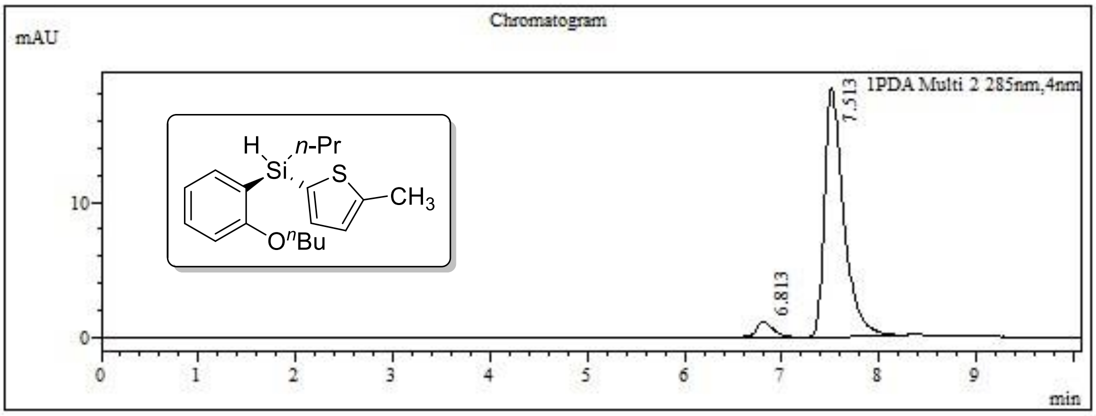

Peak Table

PDA Ch2 285nm

Peak#	Ret. Time	Area	Area%
1	6.813	13690	5.182
2	7.513	250497	94.818
总计		264187	100.000

Supplementary Figure 356. HPLC trace of **3da**

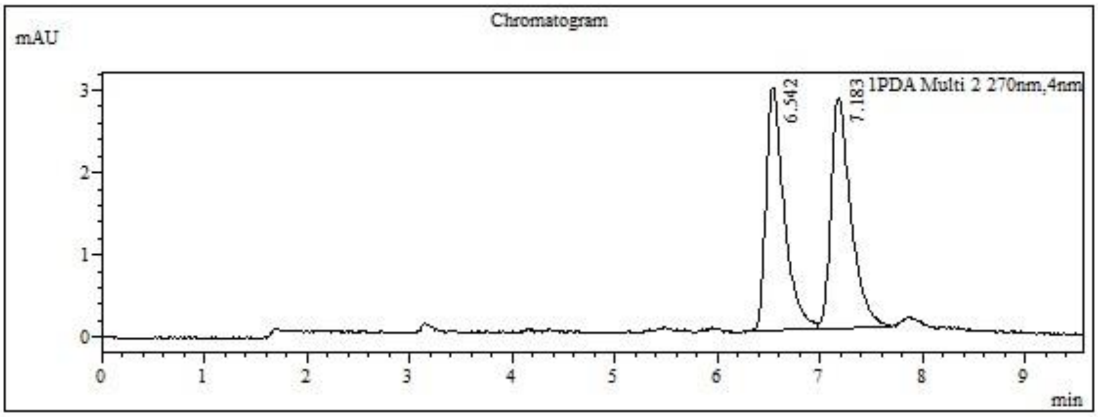

Peak Table

PDA Ch2 270nm

Peak#	Ret. Time	Area	Area%
1	6.542	36564	49.049
2	7.183	37981	50.951
总计		74545	100.000

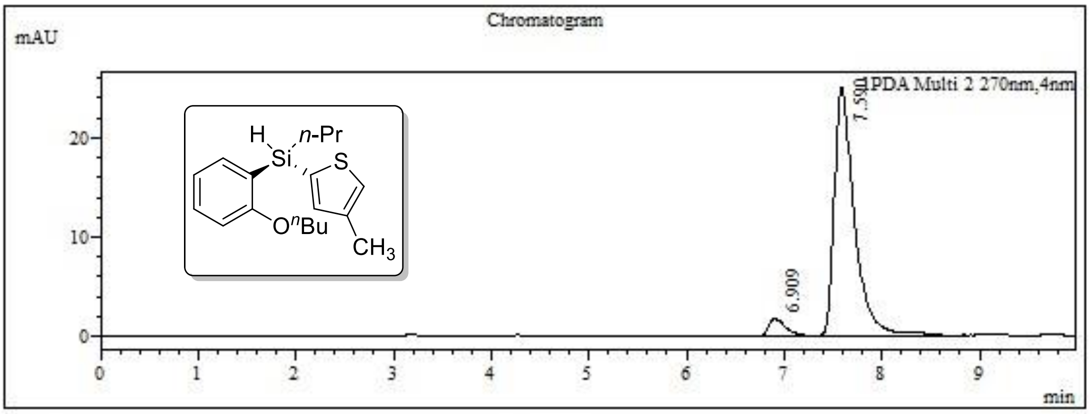

Peak Table

PDA Ch2 270nm

Peak#	Ret. Time	Area	Area%
1	6.909	21384	5.667
2	7.590	355951	94.333
总计		377334	100.000

Supplementary Figure 357. HPLC trace of **3di**

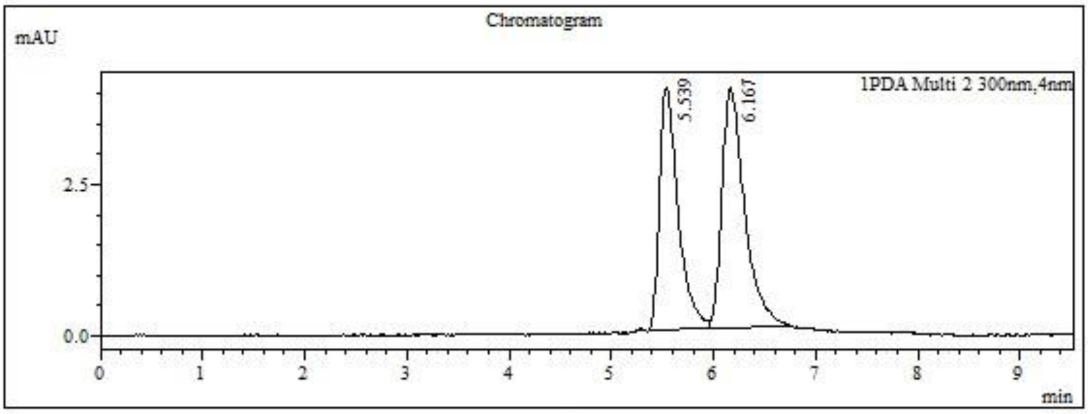

Peak Table

PDA Ch2 300nm

Peak#	Ret. Time	Area	Area%
1	5.539	48158	49.133
2	6.167	49857	50.867
总计		98015	100.000

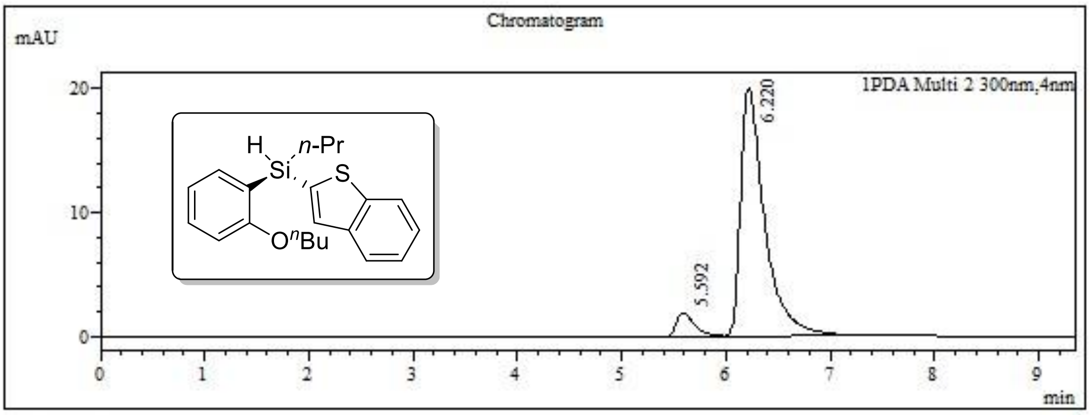

Peak Table

PDA Ch2 300nm

Peak#	Ret. Time	Area	Area%
1	5.592	24242	6.999
2	6.220	322113	93.001
总计		346355	100.000

Supplementary Figure 358. HPLC trace of **3dn**

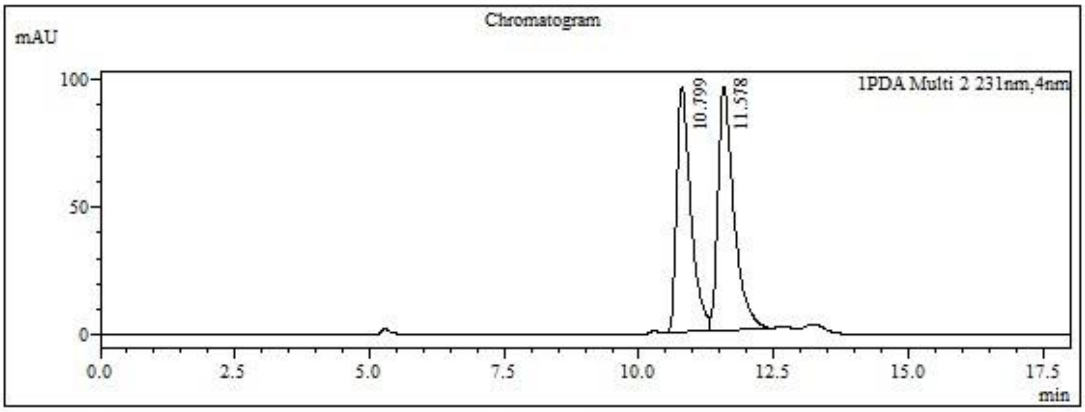

Peak Table

PDA Ch2 231nm

Peak#	Ret. Time	Area	Area%
1	10.799	1625503	49.417
2	11.578	1663877	50.583
总计		3289380	100.000

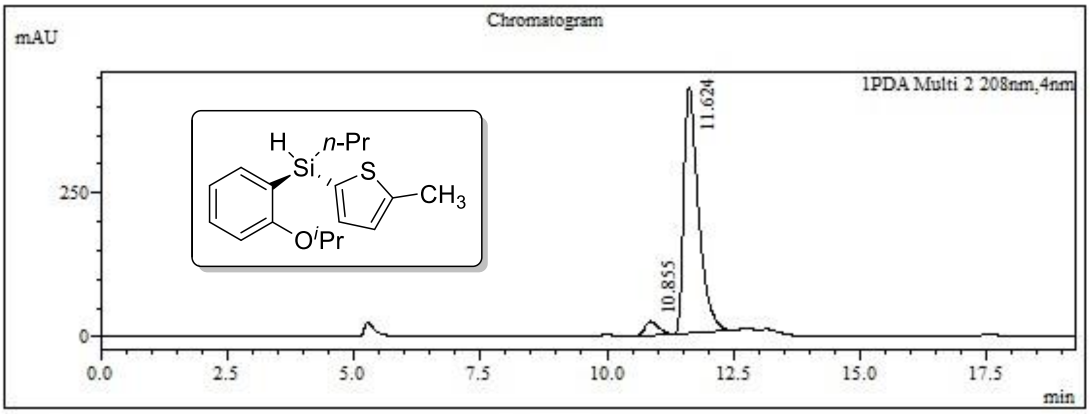

Peak Table

PDA Ch2 208nm

Peak#	Ret. Time	Area	Area%
1	10.855	454718	5.025
2	11.624	8594558	94.975
总计		9049276	100.000

Supplementary Figure 359. HPLC trace of **3ea**

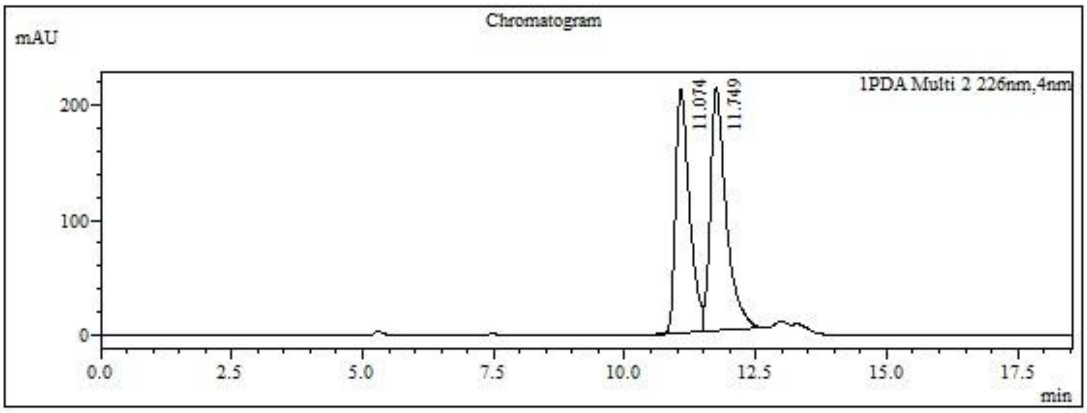

Peak Table

PDA Ch2 226nm

Peak#	Ret. Time	Area	Area%
1	11.074	3397446	49.063
2	11.749	3527159	50.937
总计		6924605	100.000

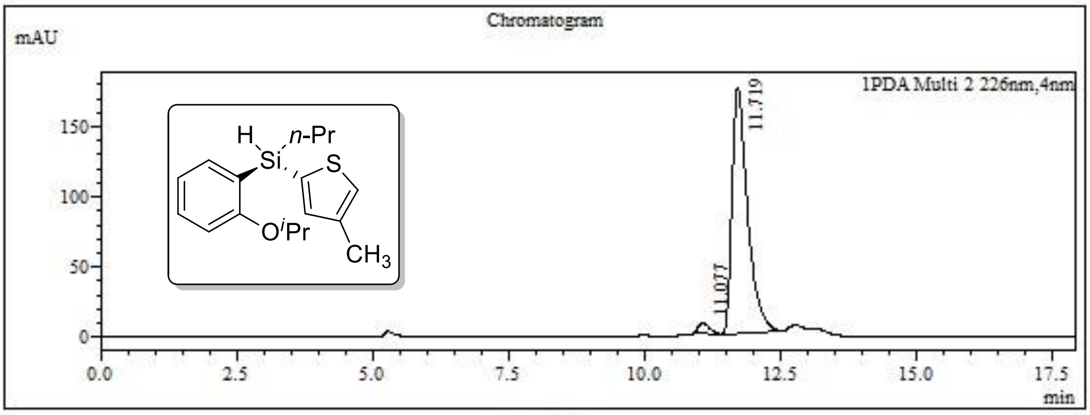

Peak Table

PDA Ch2 226nm

Peak#	Ret. Time	Area	Area%
1	11.077	108152	3.034
2	11.719	3456820	96.966
总计		3564972	100.000

Supplementary Figure 360. HPLC trace of **3ei**

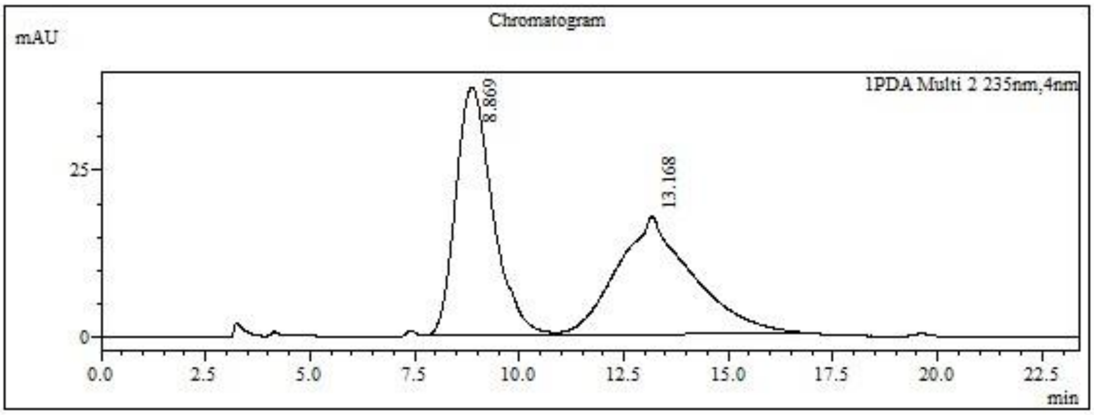

Peak Table

PDA Ch2 235nm

Peak#	Ret. Time	Area	Area%
1	8.869	2268191	50.816
2	13.168	2195322	49.184
总计		4463514	100.000

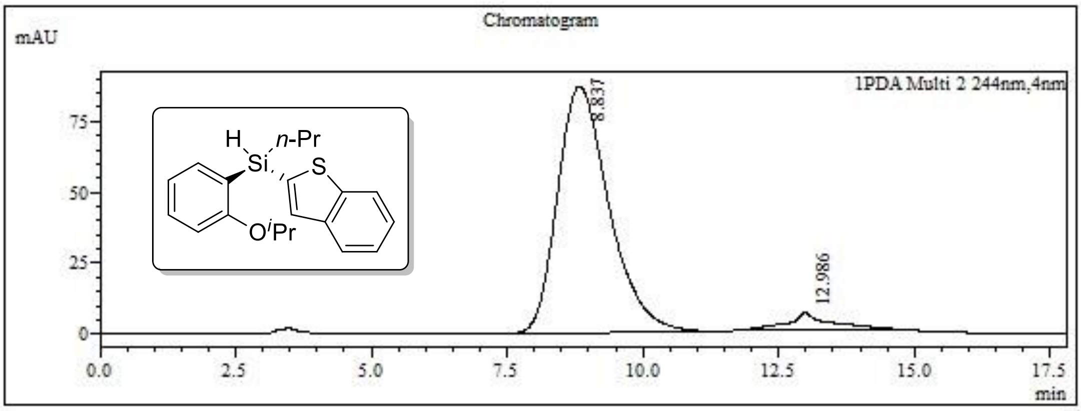

Peak Table

PDA Ch2 244nm

Peak#	Ret. Time	Area	Area%
1	8.837	5714649	94.790
2	12.986	314109	5.210
总计		6028758	100.000

Supplementary Figure 361. HPLC trace of **3en**

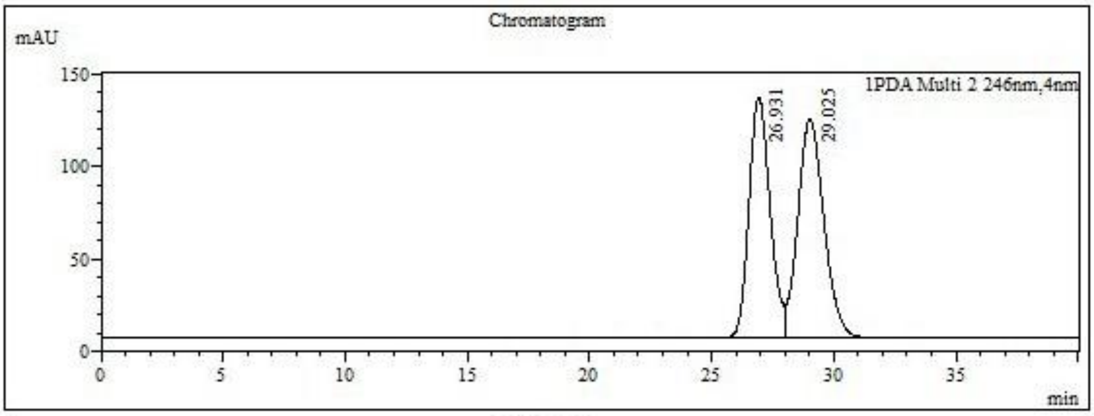

Peak Table

PDA Ch2 246nm

Peak#	Ret. Time	Area	Area%
1	26.931	7145099	49.663
2	29.025	7242016	50.337
总计		14387116	100.000

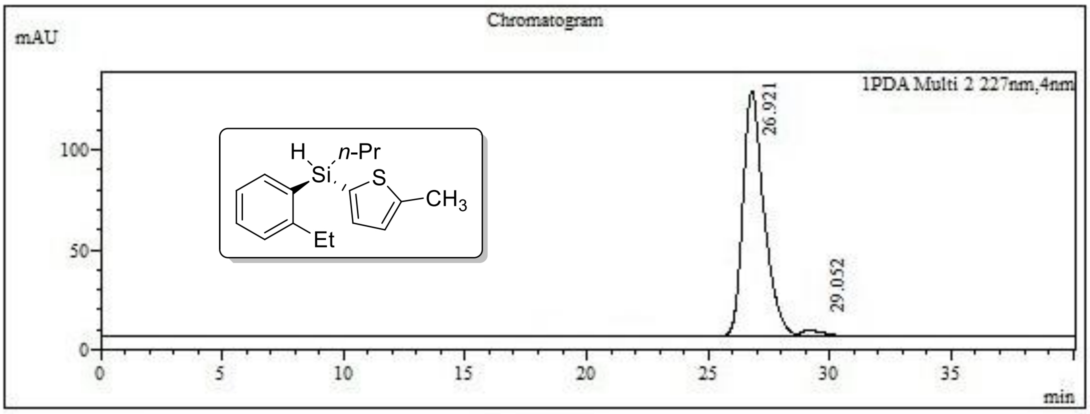

Peak Table

PDA Ch2 227nm

Peak#	Ret. Time	Area	Area%
1	26.921	8960695	97.479
2	29.052	231738	2.521
总计		9192433	100.000

Supplementary Figure 362. HPLC trace of **3fa**

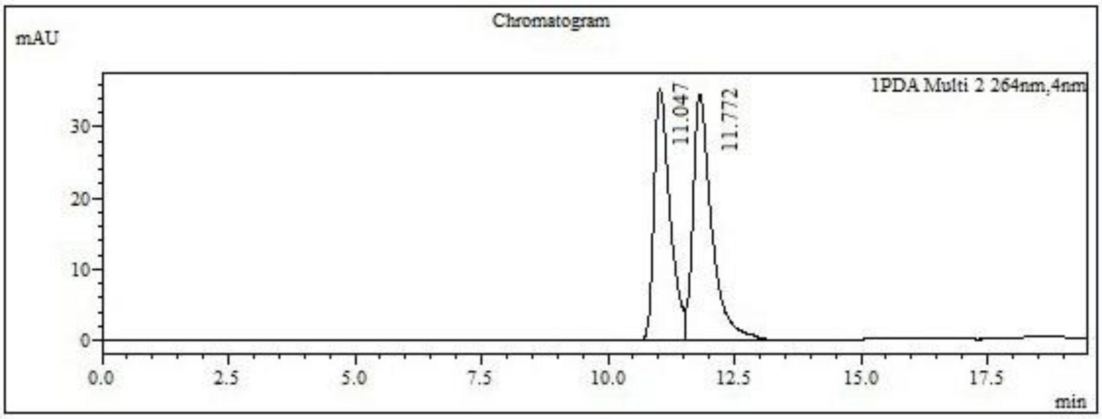

Peak Table

PDA Ch2 264nm

Peak#	Ret. Time	Area	Area%
1	11.047	7891462	50.581
2	11.772	7710215	49.419
总计		15601677	100.000

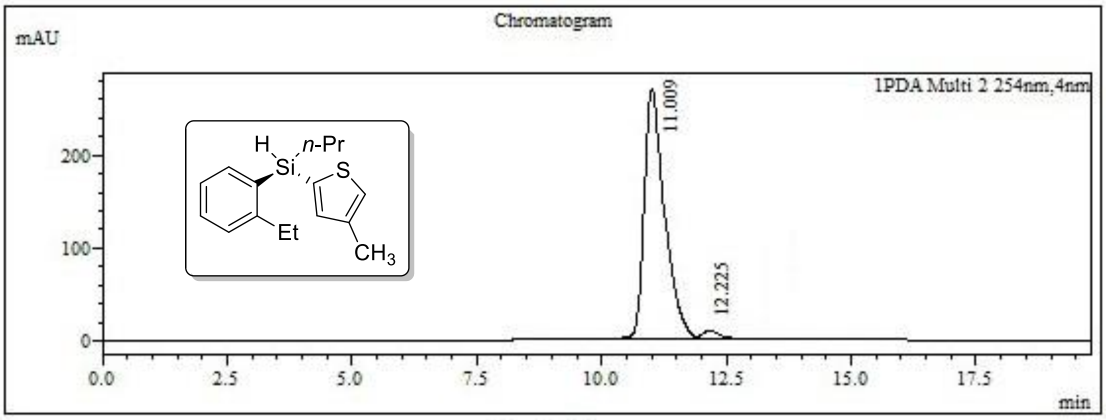

Peak Table

PDA Ch2 254nm

Peak#	Ret. Time	Area	Area%
1	11.009	9526231	95.645
2	12.225	433792	4.355
总计		9960023	100.000

Supplementary Figure 363. HPLC trace of **3fi**

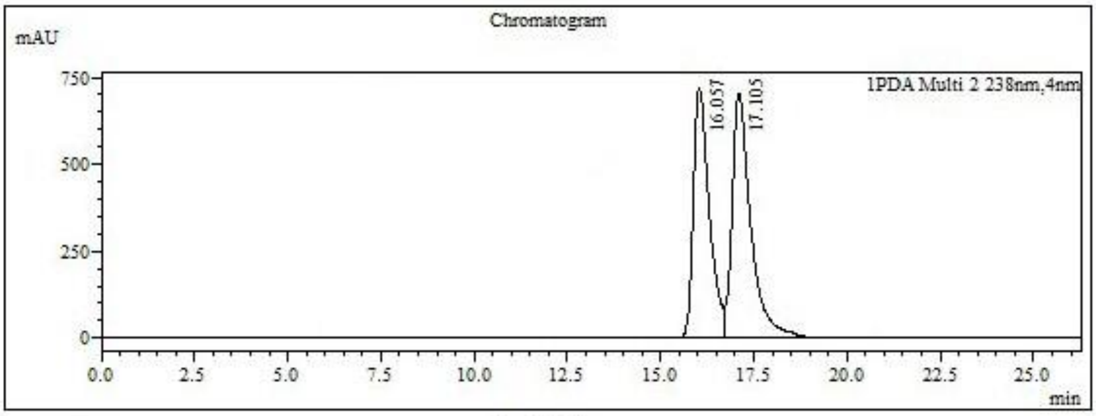

Peak Table

PDA Ch2 238nm

Peak#	Ret. Time	Area	Area%
1	16.057	11464268	49.449
2	17.105	11719529	50.551
总计		23183797	100.000

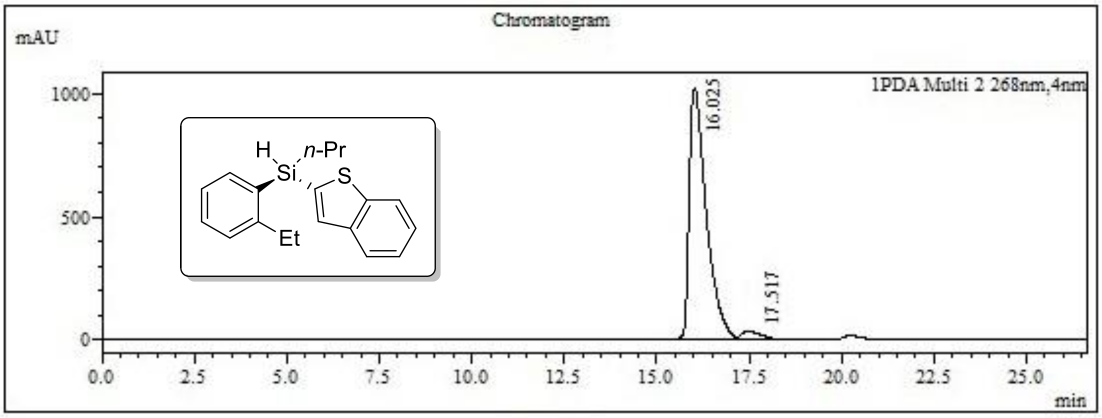

Peak Table

PDA Ch2 268nm

Peak#	Ret. Time	Area	Area%
1	16.025	33432681	96.095
2	17.517	1358666	3.905
总计		34791347	100.000

Supplementary Figure 364. HPLC trace of **3fn**

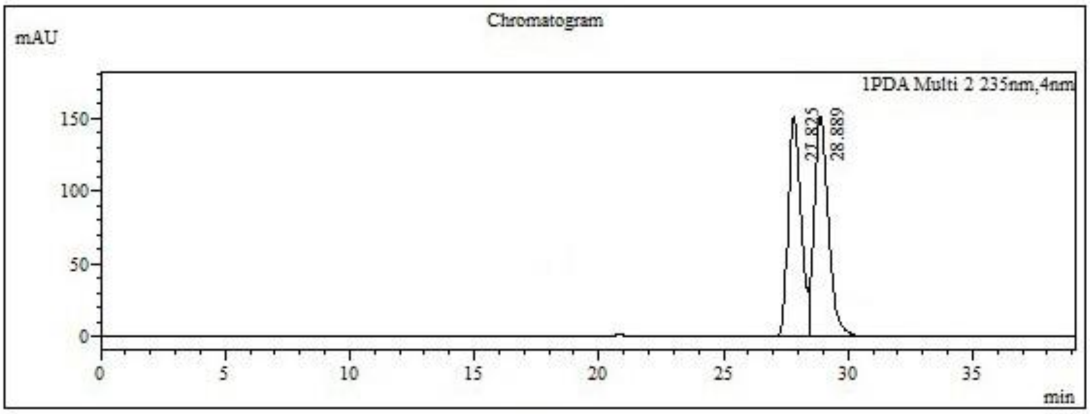

Peak Table

PDA Ch2 235nm

Peak#	Ret. Time	Area	Area%
1	27.825	4510853	50.050
2	28.889	4501845	49.950
总计		9012698	100.000

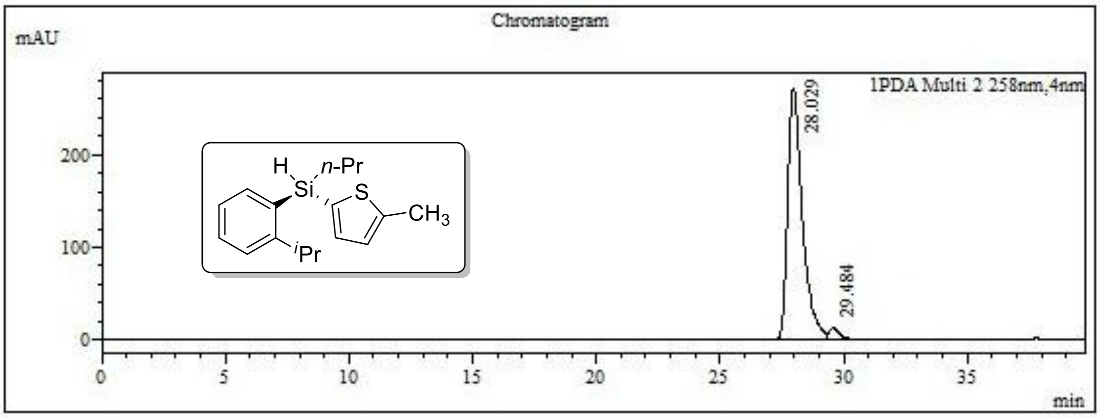

Peak Table

PDA Ch2 258nm

Peak#	Ret. Time	Area	Area%
1	28.029	11857239	94.790
2	29.484	651737	5.210
总计		12508976	100.000

Supplementary Figure 365. HPLC trace of **3ga**

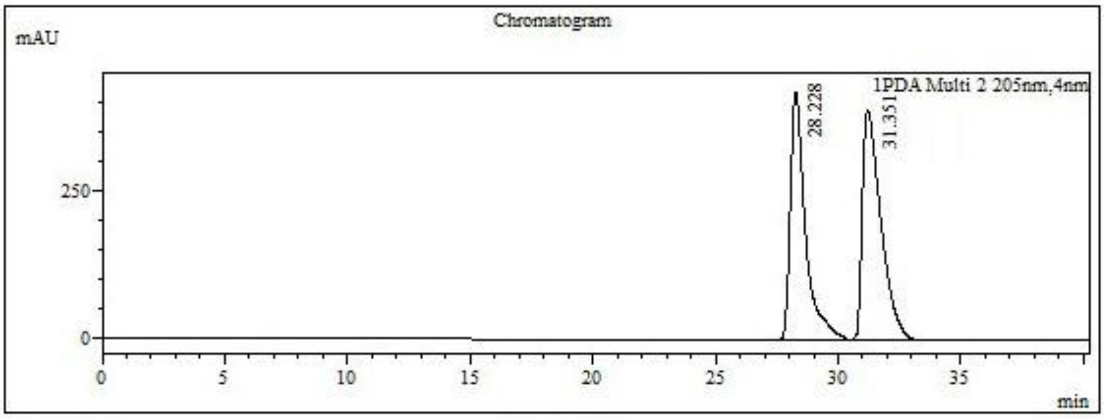

Peak Table

PDA Ch2 205nm

Peak#	Ret. Time	Area	Area%
1	28.228	4011057	50.361
2	31.351	3953556	49.639
总计		7964613	100.000

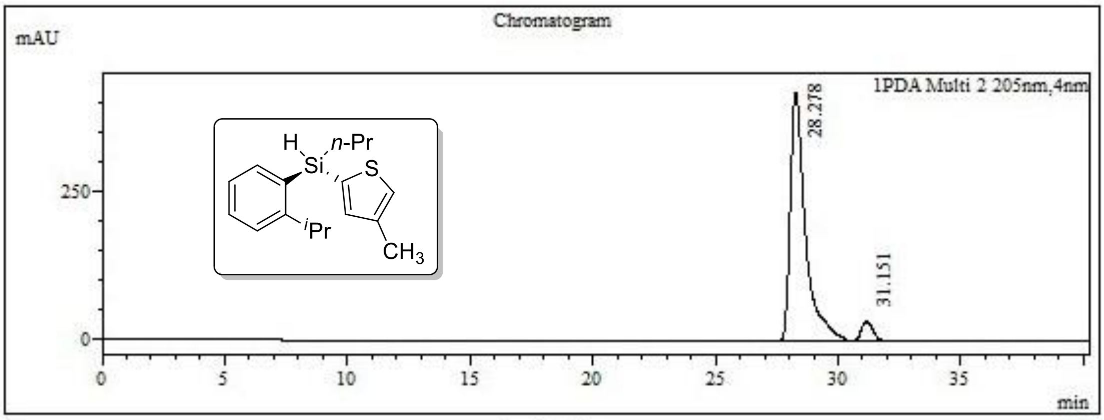

Peak Table

PDA Ch2 205nm

Peak#	Ret. Time	Area	Area%
1	28.278	18078942	94.892
2	31.151	973191	5.108
总计		19052132	100.000

Supplementary Figure 366. HPLC trace of **3gi**

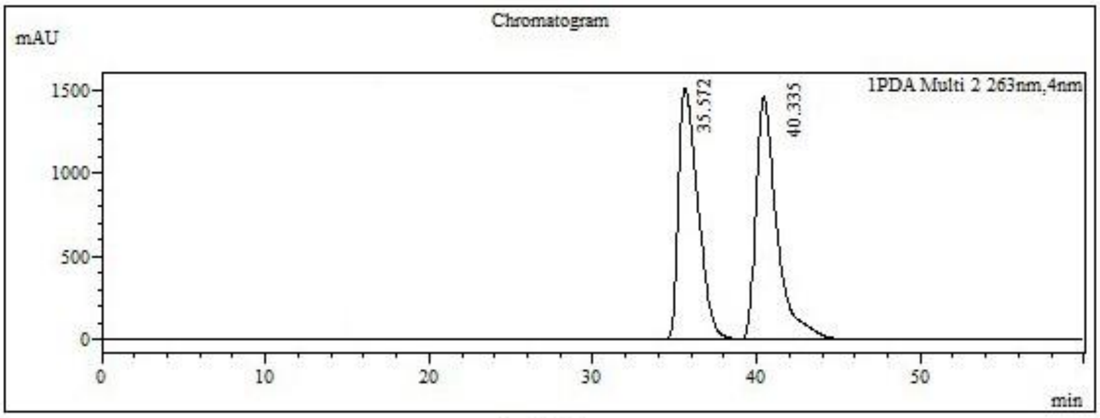

Peak Table

PDA Ch2 263nm

Peak#	Ret. Time	Area	Area%
1	35.572	13809561	50.999
2	40.335	13268709	49.001
总计		27078270	100.000

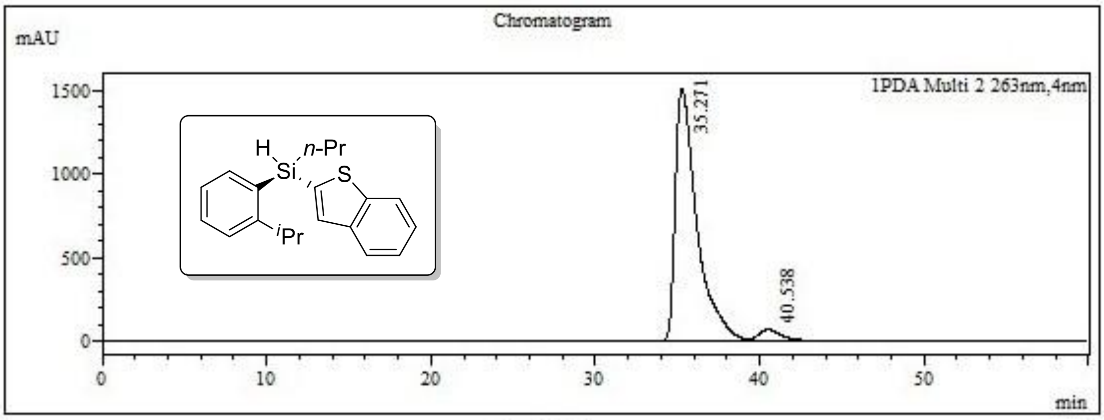

Peak Table

PDA Ch2 263nm

Peak#	Ret. Time	Area	Area%
1	35.271	136580252	96.303
2	40.538	5243073	3.697
总计		141823325	100.000

Supplementary Figure 367. HPLC trace of **3gn**

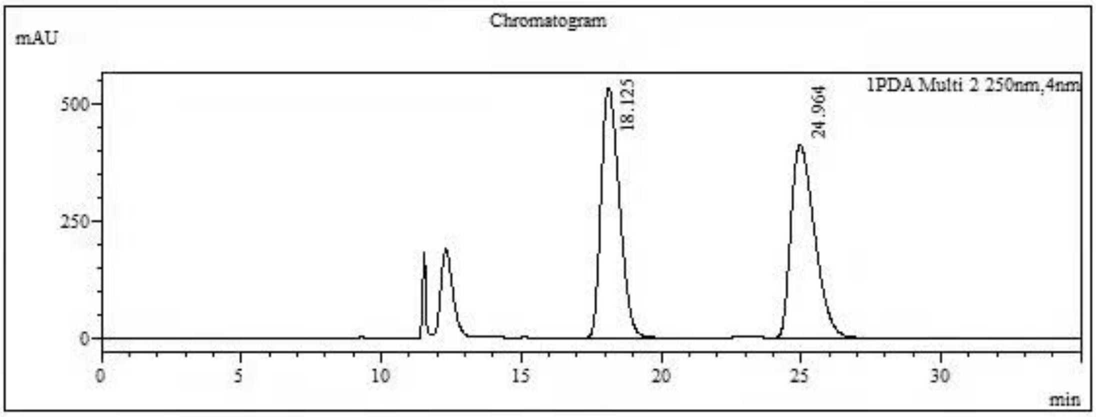

PDA Ch2 250nm

Peak Table

Peak#	Ret. Time	Area	Area%
1	18.125	24122766	49.961
2	24.964	24160104	50.039
总计		48282870	100.000

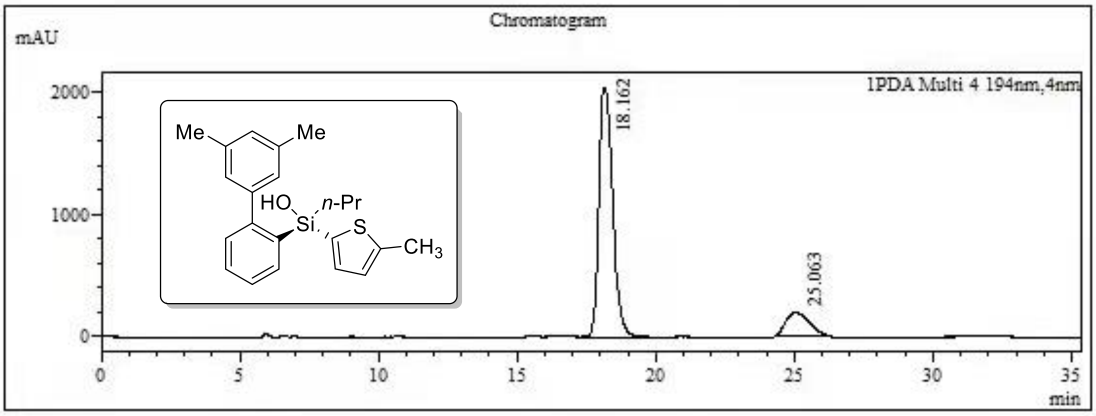

PDA Ch2 194nm

Peak Table

Peak#	Ret. Time	Area	Area%
1	18.162	70577424	86.932
2	25.063	10609856	13.068
总计		81187280	100.000

Supplementary Figure 368. HPLC trace of **3ha-1**

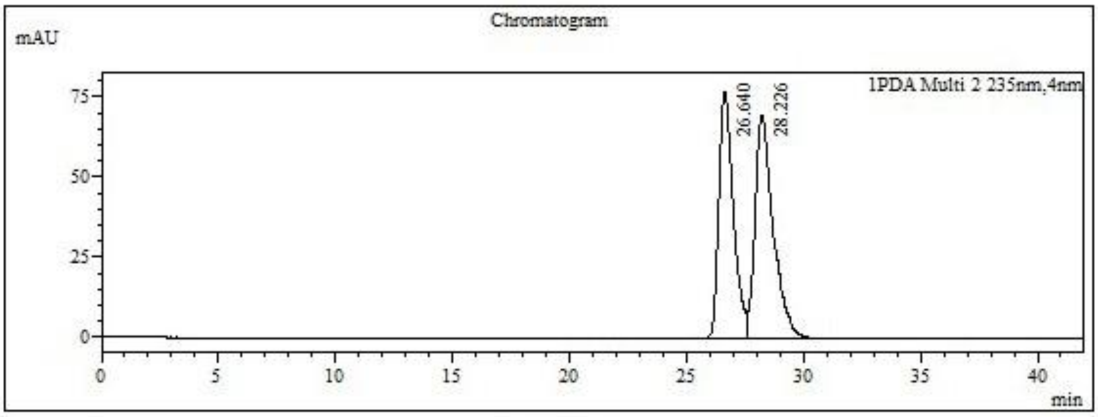

Peak Table

PDA Ch2 235nm

Peak#	Ret. Time	Area	Area%
1	26.640	2916060	50.380
2	28.226	2872016	49.620
总计		5788077	100.000

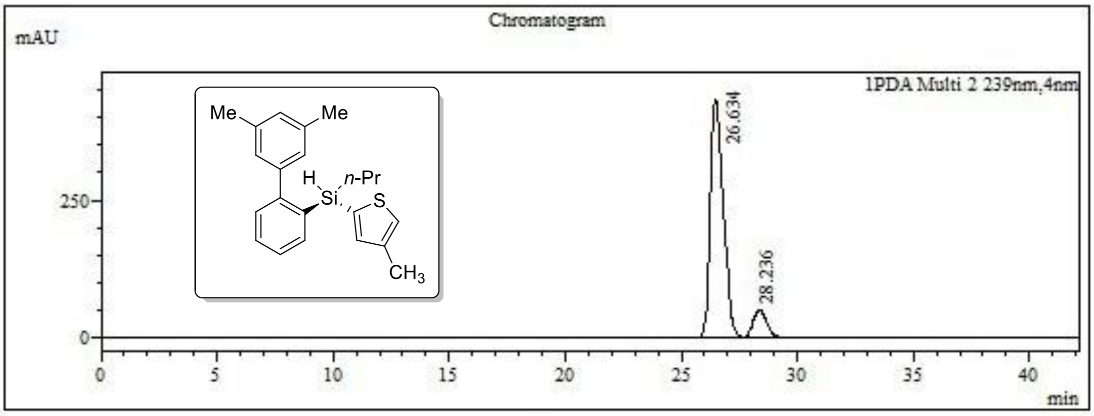

Peak Table

PDA Ch2 239nm

Peak#	Ret. Time	Area	Area%
1	26.634	17309131	90.460
2	28.236	1825399	9.540
总计		19134529	100.000

Supplementary Figure 369. HPLC trace of **3hi**

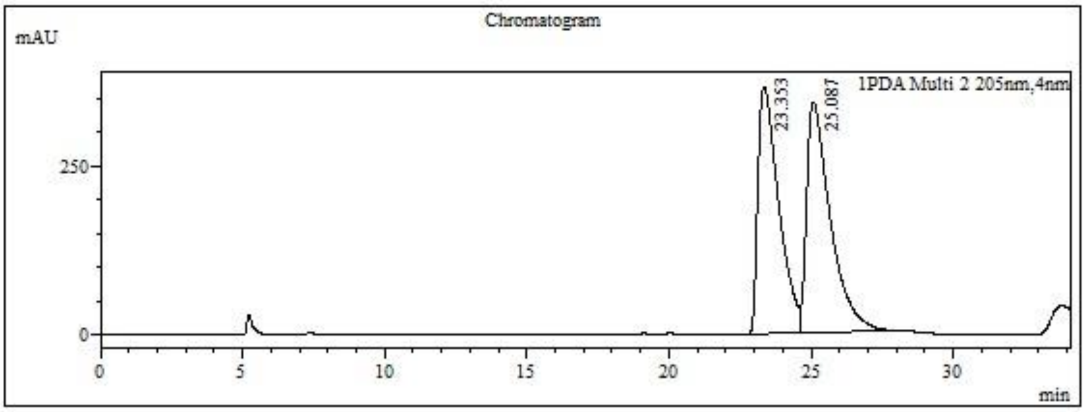

PDA Ch2 205nm

Peak Table

Peak#	Ret. Time	Area	Area%
1	23.353	18404691	49.435
2	25.087	18825106	50.565
总计		37229797	100.000

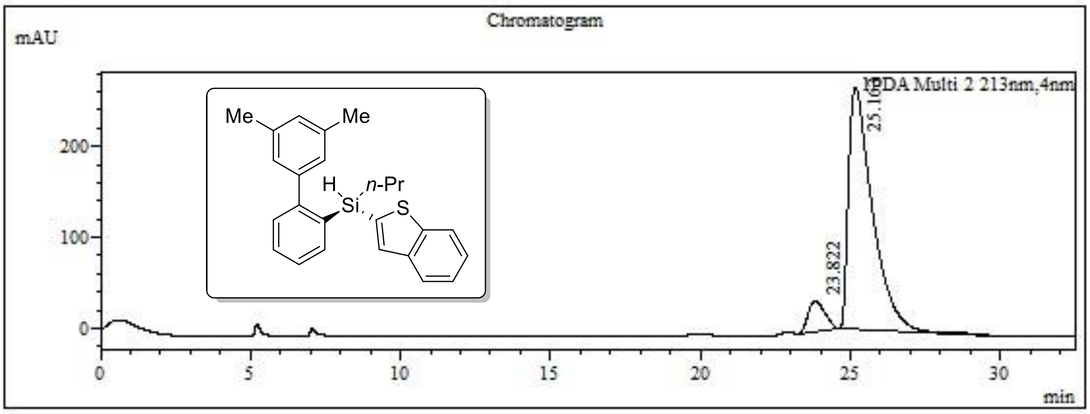

PDA Ch2 213nm

Peak Table

Peak#	Ret. Time	Area	Area%
1	23.822	1314217	8.079
2	25.163	14952239	91.921
总计		16266456	100.000

Supplementary Figure 370. HPLC trace of **3hn**

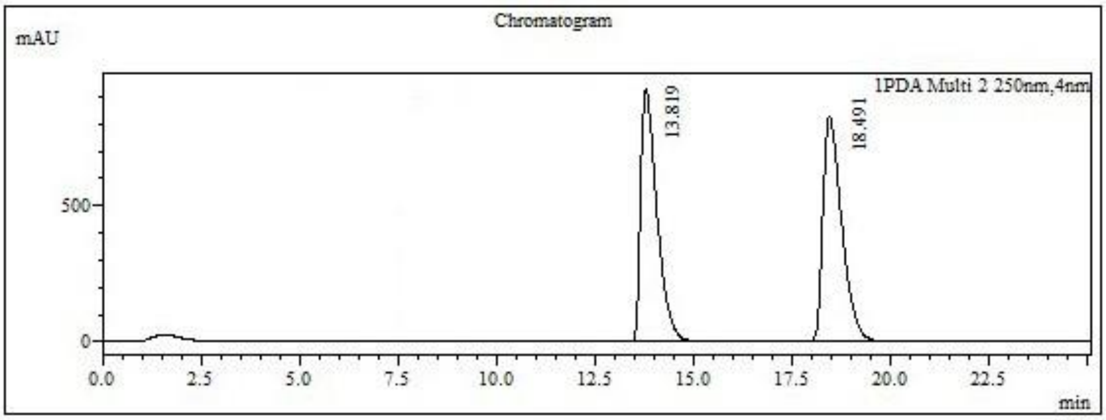

Peak Table

PDA Ch2 250nm

Peak#	Ret. Time	Area	Area%
1	13.819	1650406	49.986
2	18.491	1651356	50.014
总计		3301763	100.000

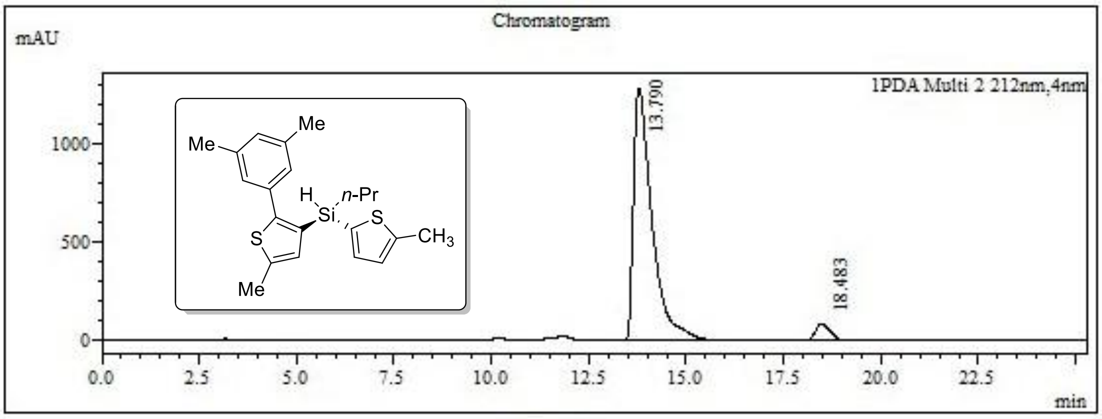

Peak Table

PDA Ch2 212nm

Peak#	Ret. Time	Area	Area%
1	13.790	42373227	94.984
2	18.483	2237764	5.016
总计		44610991	100.000

Supplementary Figure 371. HPLC trace of **3ia**

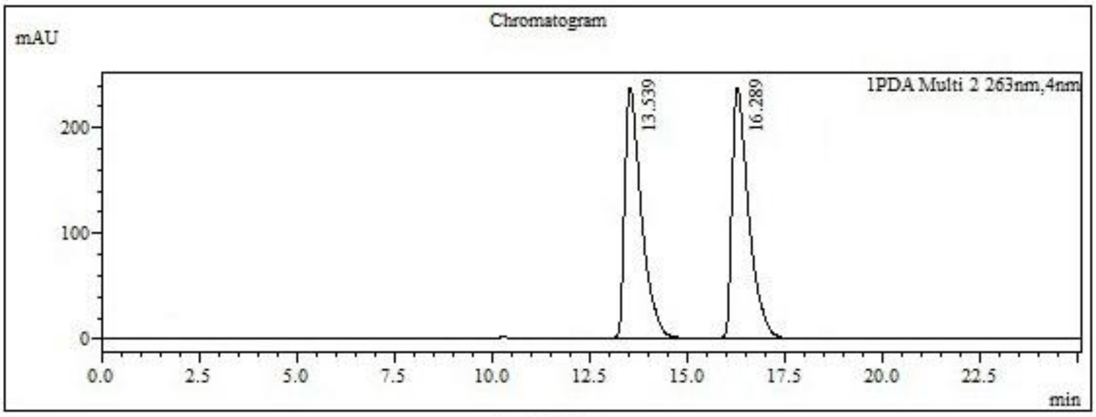

Peak Table

PDA Ch2 263nm

Peak#	Ret. Time	Area	Area%
1	13.539	12361009	49.204
2	16.289	12760822	50.796
总计		25121832	100.000

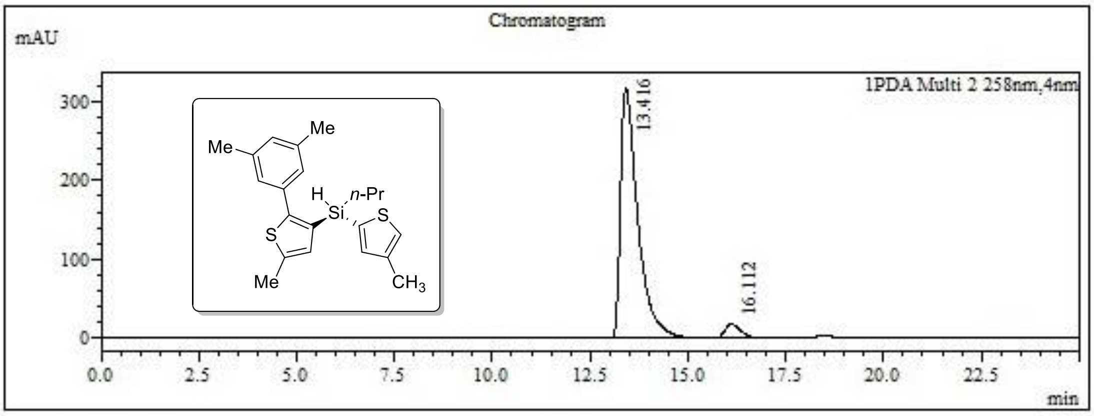

Peak Table

PDA Ch2 258nm

Peak#	Ret. Time	Area	Area%
1	13.416	9862916	95.598
2	16.112	454138	4.402
总计		10317055	100.000

Supplementary Figure 372. HPLC trace of **3ii**

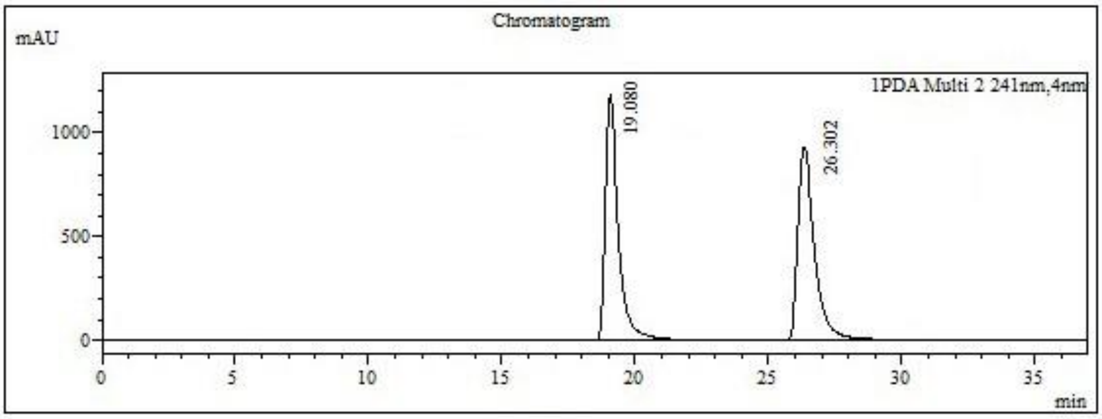

Peak Table

PDA Ch2 241nm

Peak#	Ret. Time	Area	Area%
1	19.080	14008587	49.506
2	26.302	14287998	50.494
总计		28296585	100.000

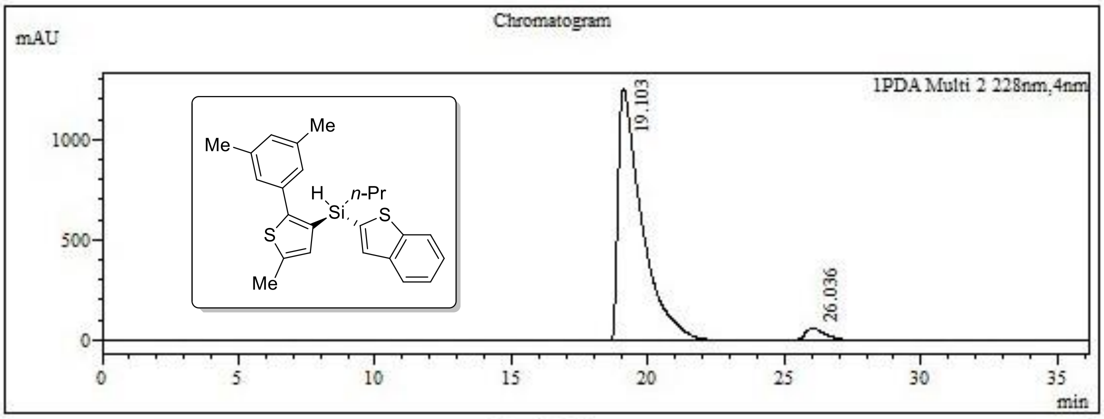

Peak Table

PDA Ch2 228nm

Peak#	Ret. Time	Area	Area%
1	19.103	76135942	96.644
2	26.036	2643476	3.356
总计		78779417	100.000

Supplementary Figure 373. HPLC trace of **3in**

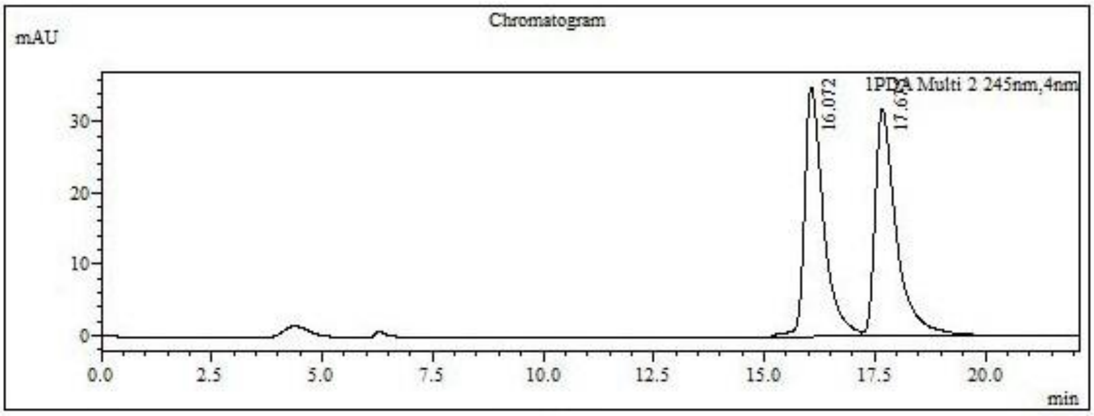

Peak Table

PDA Ch2 245nm

Peak#	Ret. Time	Area	Area%
1	16.072	1049386	49.761
2	17.675	1059469	50.239
总计		2108855	100.000

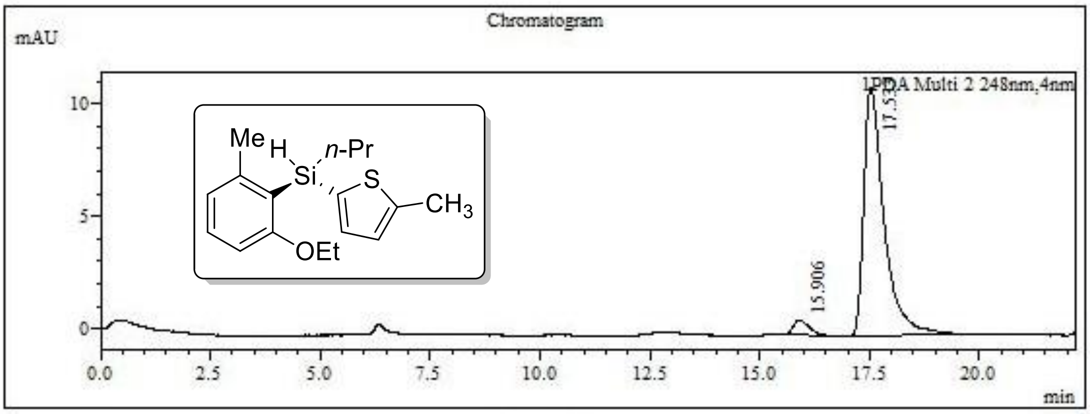

Peak Table

PDA Ch2 248nm

Peak#	Ret. Time	Area	Area%
1	15.906	17409	4.678
2	17.533	354756	95.322
总计		372165	100.000

Supplementary Figure 374. HPLC trace of **3ja**

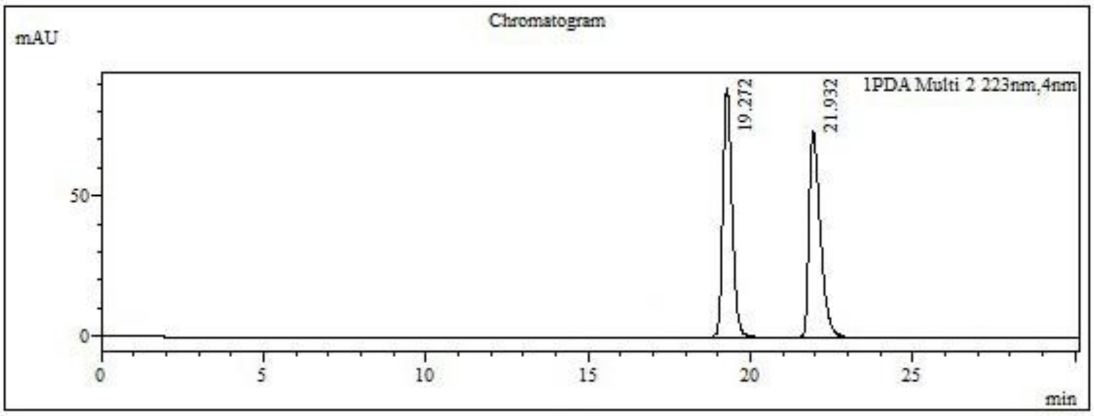

Peak Table

PDA Ch2 223nm

Peak#	Ret. Time	Area	Area%
1	19.272	1708392	49.267
2	21.932	1759222	50.733
总计		3467614	100.000

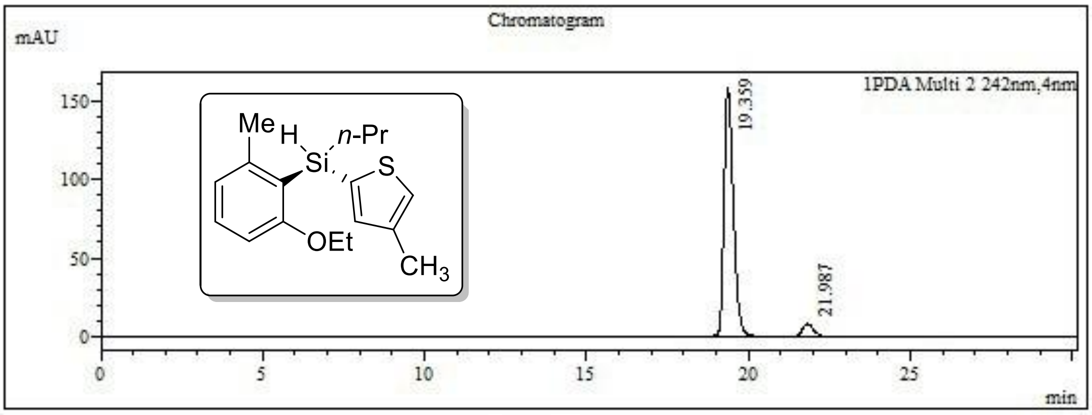

Peak Table

PDA Ch2 242nm

Peak#	Ret. Time	Area	Area%
1	19.359	3179638	94.937
2	21.987	169575	5.063
总计		3349212	100.000

Supplementary Figure 375. HPLC trace of **3ji**

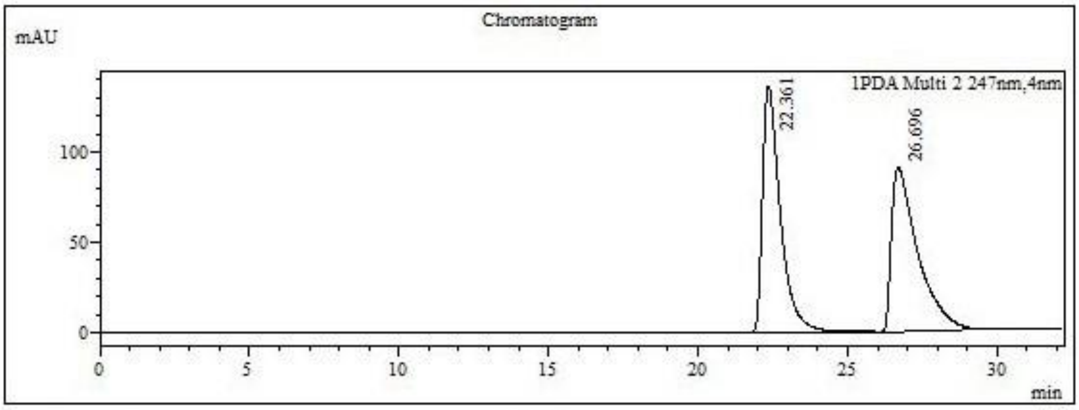

Peak Table

PDA Ch2 247nm

Peak#	Ret. Time	Area	Area%
1	22.361	5724040	50.930
2	26.696	5515014	49.070
总计		11239055	100.000

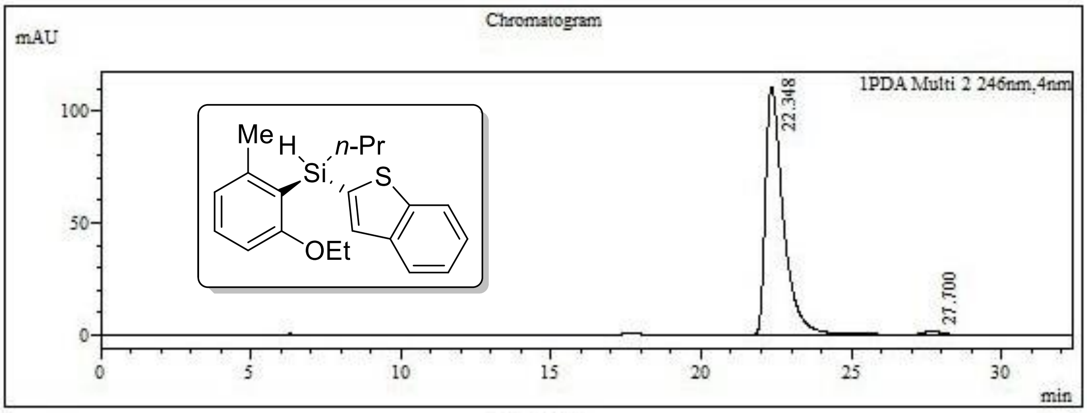

Peak Table

PDA Ch2 246nm

Peak#	Ret. Time	Area	Area%
1	22.348	4704123	98.135
2	27.700	89400	1.865
总计		4793523	100.000

Supplementary Figure 376. HPLC trace of **3jn**

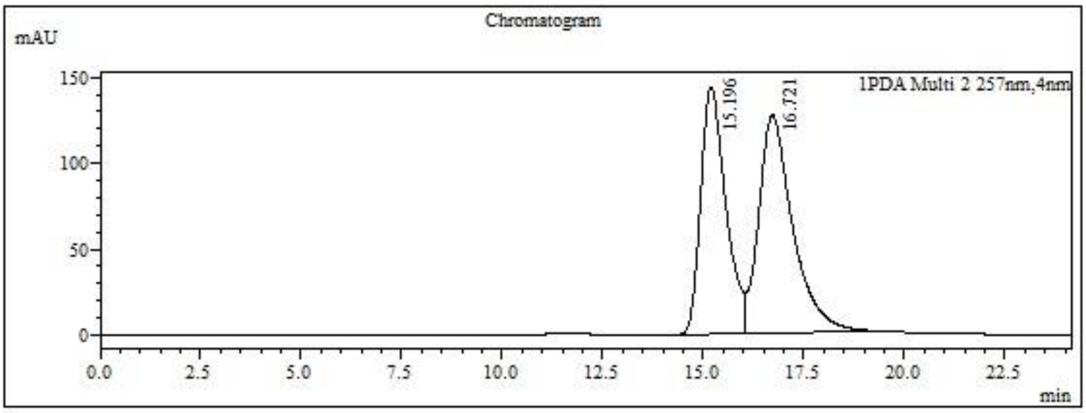

Peak Table

PDA Ch2 257nm

Peak#	Ret. Time	Area	Area%
1	15.196	5983350	49.662
2	16.721	6064777	50.338
总计		12048127	100.000

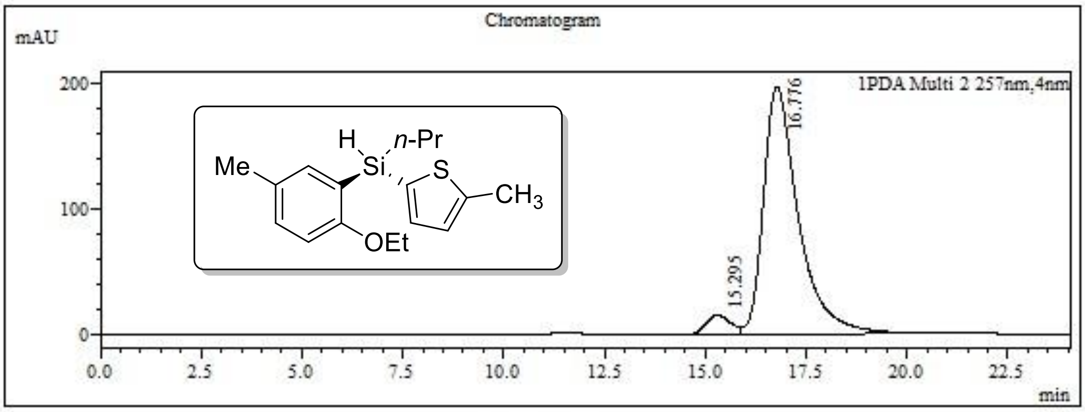

Peak Table

PDA Ch2 257nm

Peak#	Ret. Time	Area	Area%
1	15.295	668378	5.301
2	16.776	11940743	94.699
总计		12609121	100.000

Supplementary Figure 377. HPLC trace of **3ka**

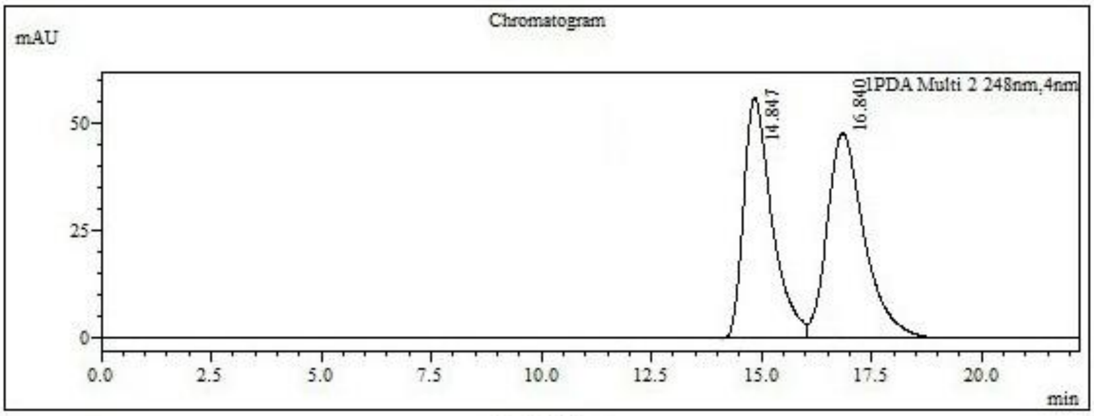

Peak Table

PDA Ch2 248nm

Peak#	Ret. Time	Area	Area%
1	14.847	2312135	49.634
2	16.840	2346215	50.366
总计		4658351	100.000

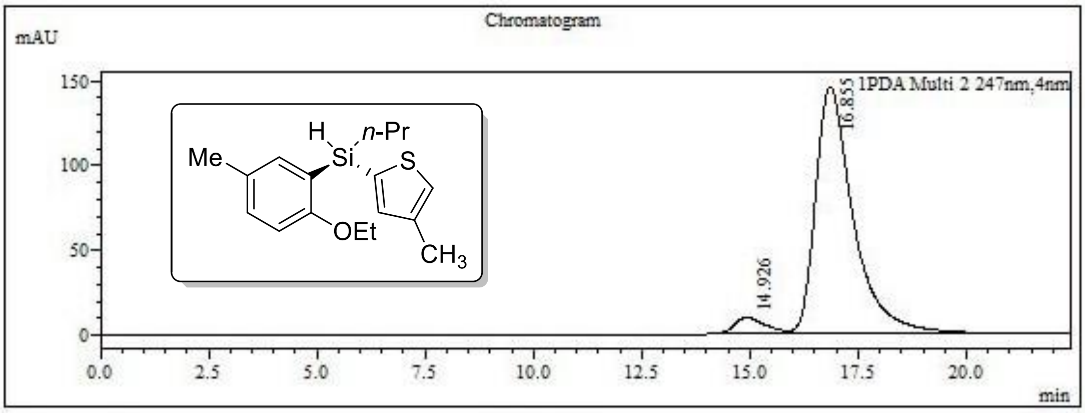

Peak Table

PDA Ch2 247nm

Peak#	Ret. Time	Area	Area%
1	14.926	420734	4.595
2	16.855	8734990	95.405
总计		9155724	100.000

Supplementary Figure 378. HPLC trace of **3ki**

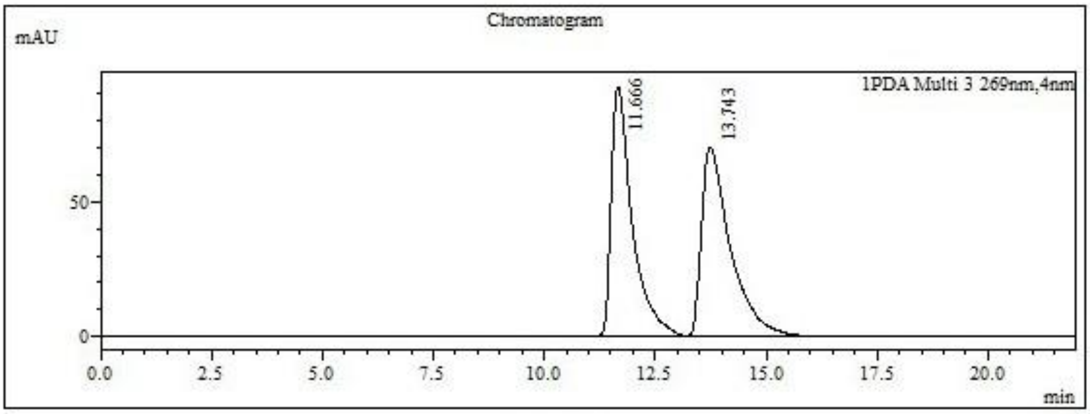

Peak Table

PDA Ch3 269nm

Peak#	Ret. Time	Area	Area%
1	11.666	3144501	50.272
2	13.743	3110533	49.728
总计		6255034	100.000

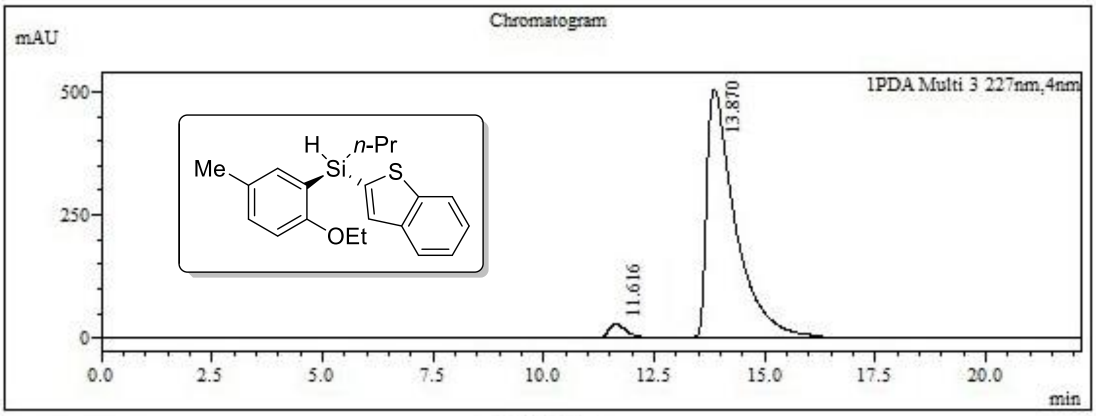

Peak Table

PDA Ch3 227nm

Peak#	Ret. Time	Area	Area%
1	11.616	725830	3.030
2	13.870	23226092	96.970
总计		23951922	100.000

Supplementary Figure 379. HPLC trace of **3kn**

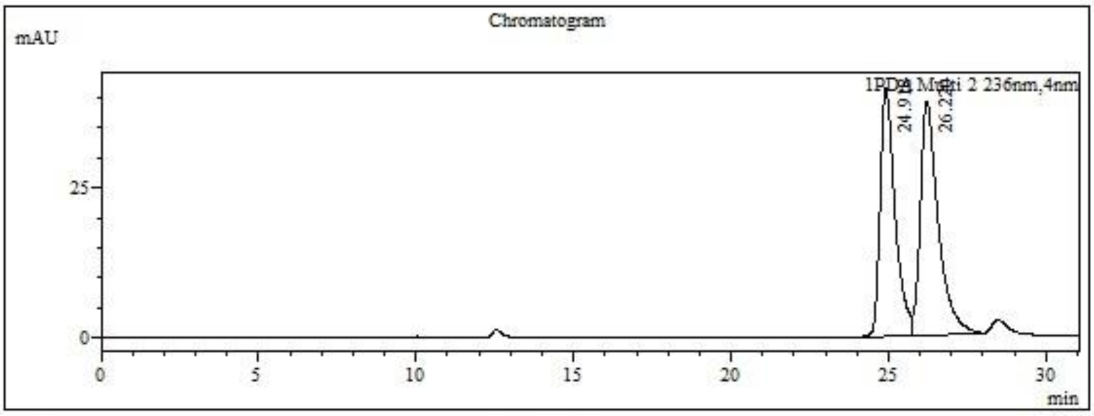

Peak Table

PDA Ch2 236nm

Peak#	Ret. Time	Area	Area%
1	24.918	1210217	49.342
2	26.220	1242494	50.658
总计		2452711	100.000

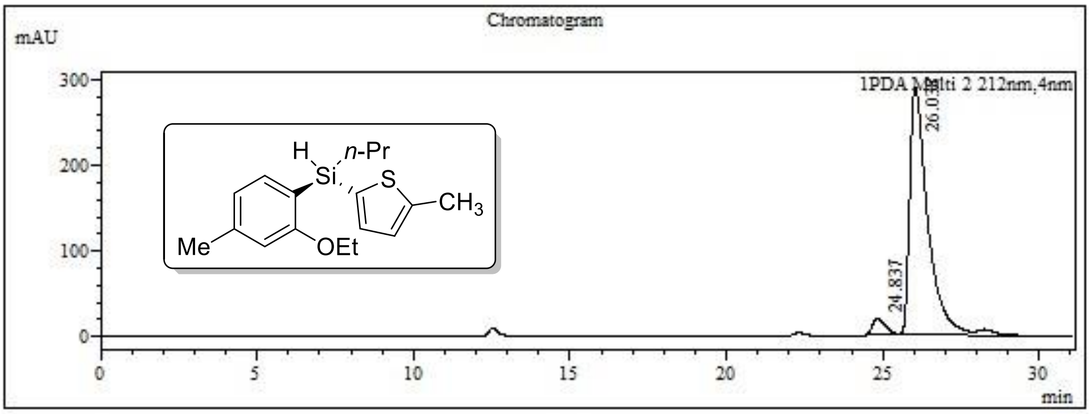

Peak Table

PDA Ch2 212nm

Peak#	Ret. Time	Area	Area%
1	24.837	538518	4.410
2	26.038	11673800	95.590
总计		12212318	100.000

Supplementary Figure 380. HPLC trace of **3la**

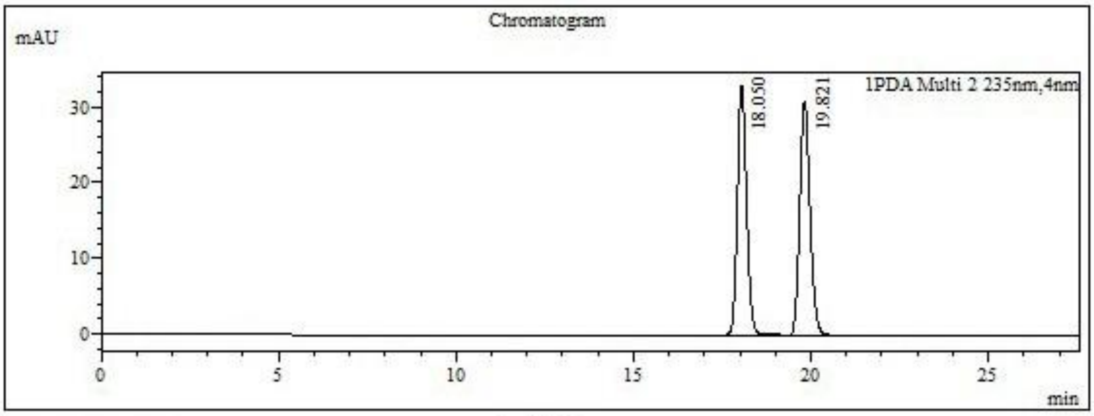

Peak Table

PDA Ch2 235nm

Peak#	Ret. Time	Area	Area%
1	18.050	601734	49.233
2	19.821	620471	50.767
总计		1222204	100.000

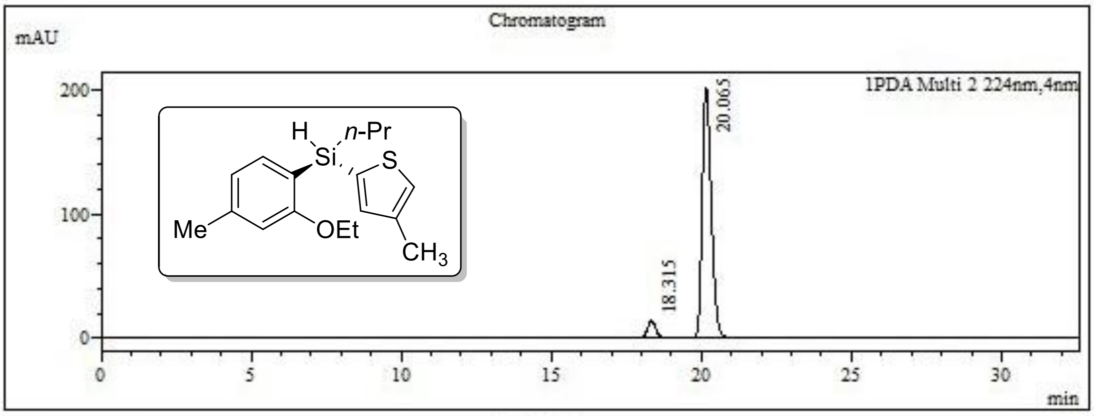

Peak Table

PDA Ch2 224nm

Peak#	Ret. Time	Area	Area%
1	18.315	233340	5.289
2	20.065	4178295	94.711
总计		4411634	100.000

Supplementary Figure 381. HPLC trace of **3li**

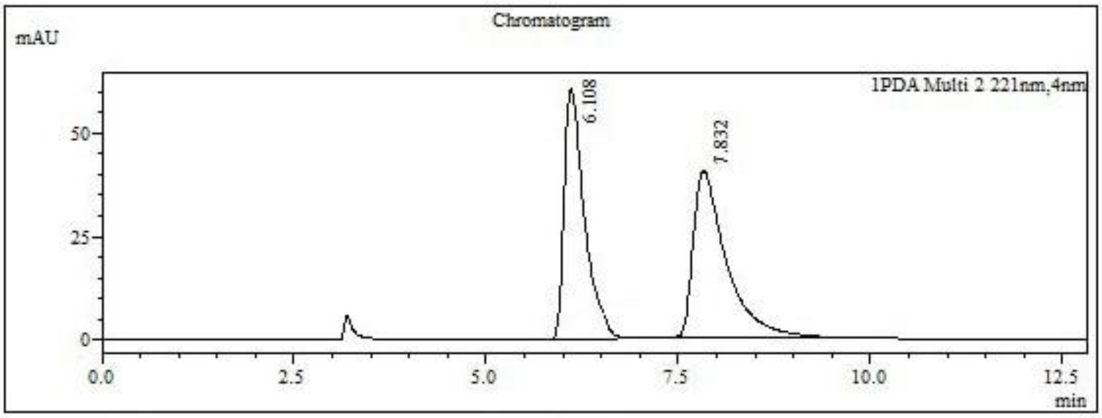

Peak Table

PDA Ch2 221nm

Peak#	Ret. Time	Area	Area%
1	6.108	1227116	50.523
2	7.832	1201713	49.477
总计		2428829	100.000

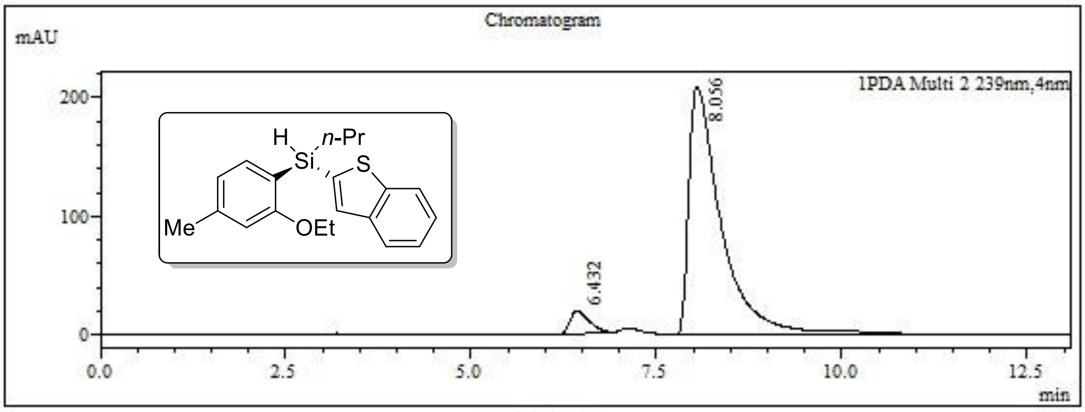

Peak Table

PDA Ch2 239nm

Peak#	Ret. Time	Area	Area%
1	6.432	354442	5.246
2	8.056	6402433	94.754
总计		6756875	100.000

Supplementary Figure 382. HPLC trace of **3ln**

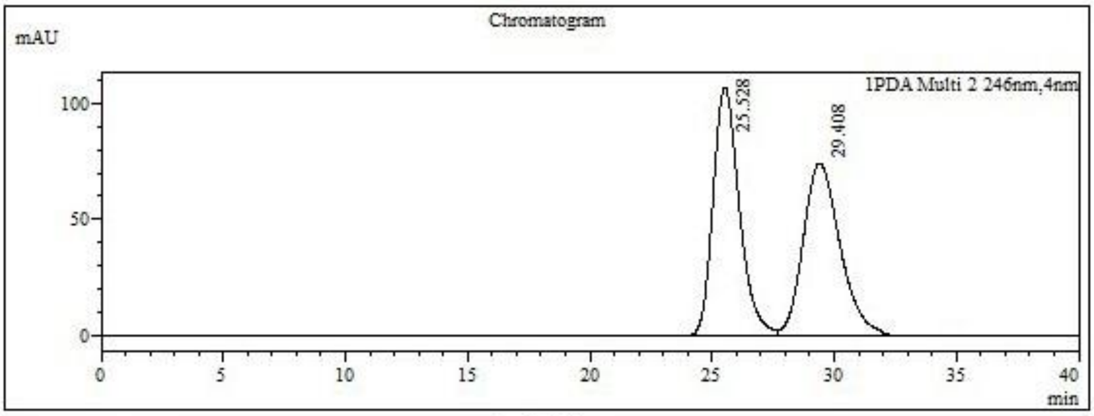

Peak Table

PDA Ch2 246nm

Peak#	Ret. Time	Area	Area%
1	25.528	7989833	50.892
2	29.408	7709652	49.108
总计		15699485	100.000

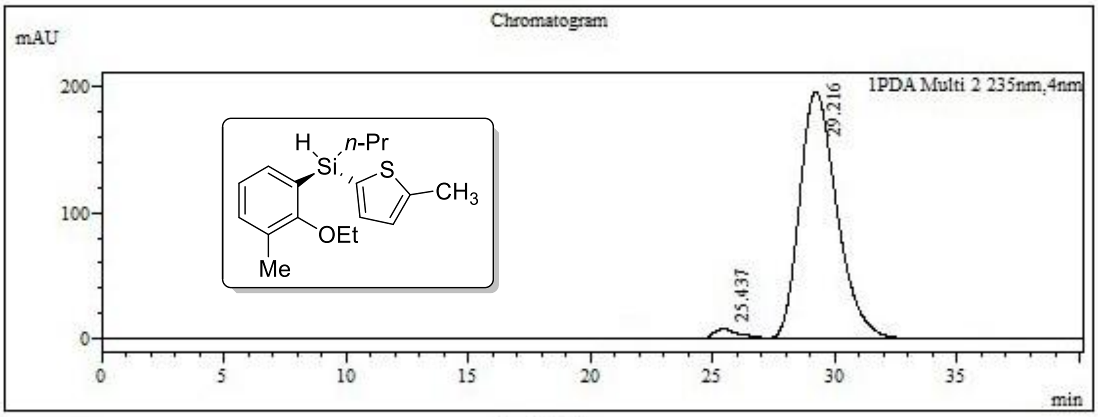

Peak Table

PDA Ch2 235nm

Peak#	Ret. Time	Area	Area%
1	25.437	617770	2.904
2	29.216	20651956	97.096
总计		21269726	100.000

Supplementary Figure 383. HPLC trace of **3ma**

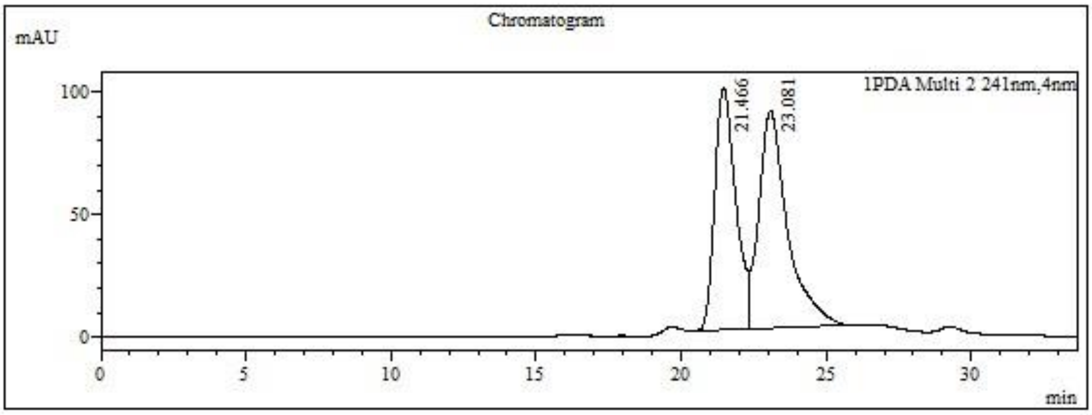

Peak Table

PDA Ch2 241nm

Peak#	Ret. Time	Area	Area%
1	21.466	4796269	49.222
2	23.081	4947945	50.778
总计		9744214	100.000

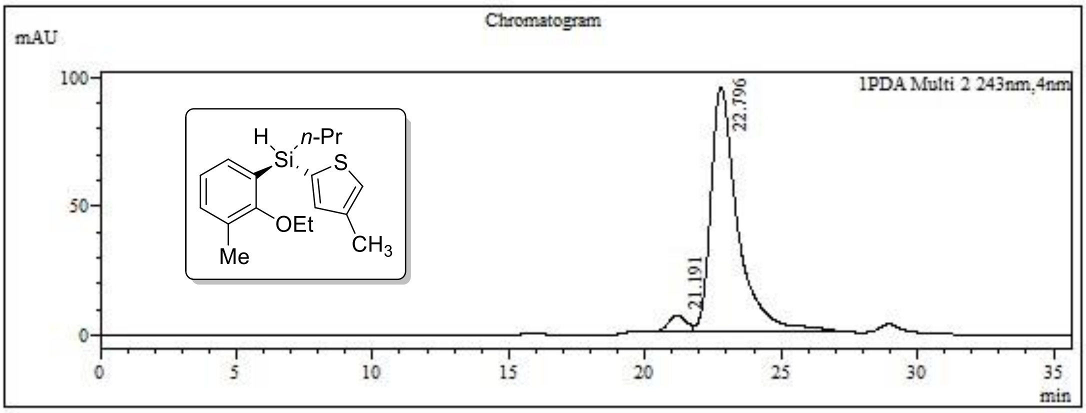

Peak Table

PDA Ch2 243nm

Peak#	Ret. Time	Area	Area%
1	21.191	274570	4.113
2	22.796	6400350	95.887
总计		6674920	100.000

Supplementary Figure 384. HPLC trace of **3mi**

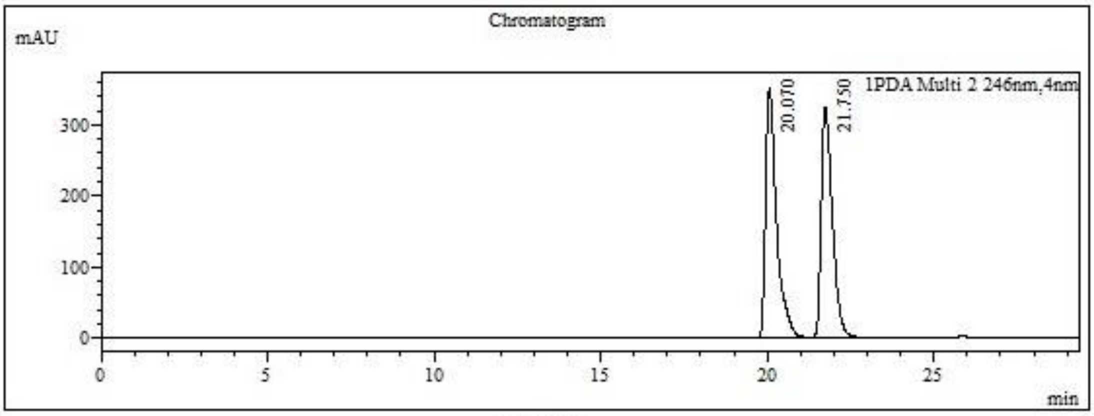

Peak Table

PDA Ch2 246nm

Peak#	Ret. Time	Area	Area%
1	20.070	7369718	49.554
2	21.750	7502472	50.446
总计		14872190	100.000

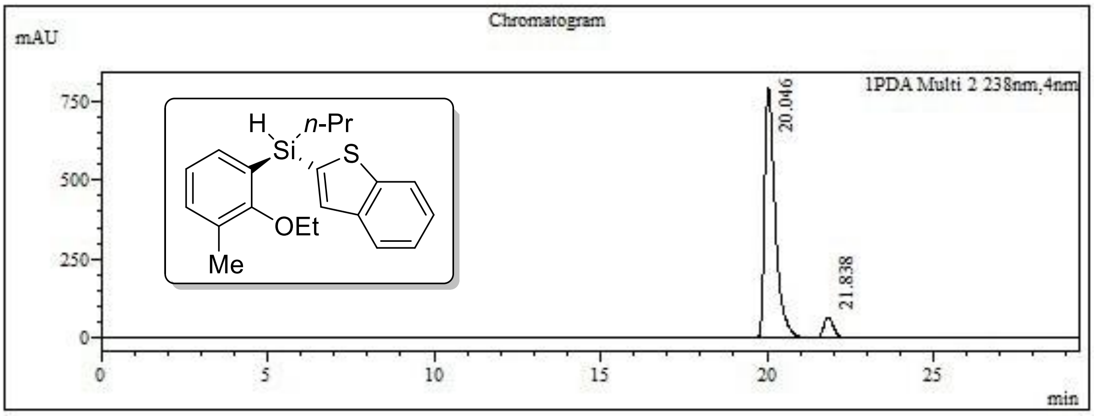

Peak Table

PDA Ch2 238nm

Peak#	Ret. Time	Area	Area%
1	20.046	17496874	93.328
2	21.838	1250879	6.672
总计		18747753	100.000

Supplementary Figure 385. HPLC trace of **3mn**

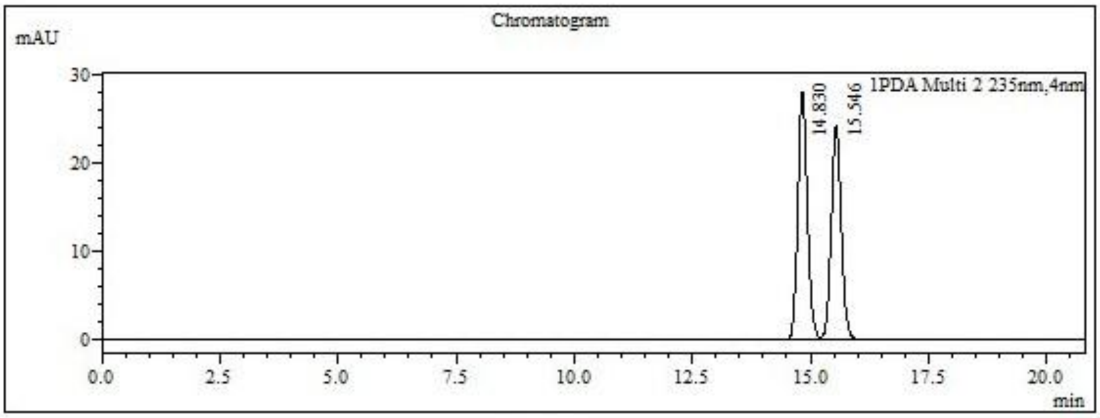

Peak Table

PDA Ch2 235nm

Peak#	Ret. Time	Area	Area%
1	14.830	352540	50.614
2	15.546	343989	49.386
总计		696530	100.000

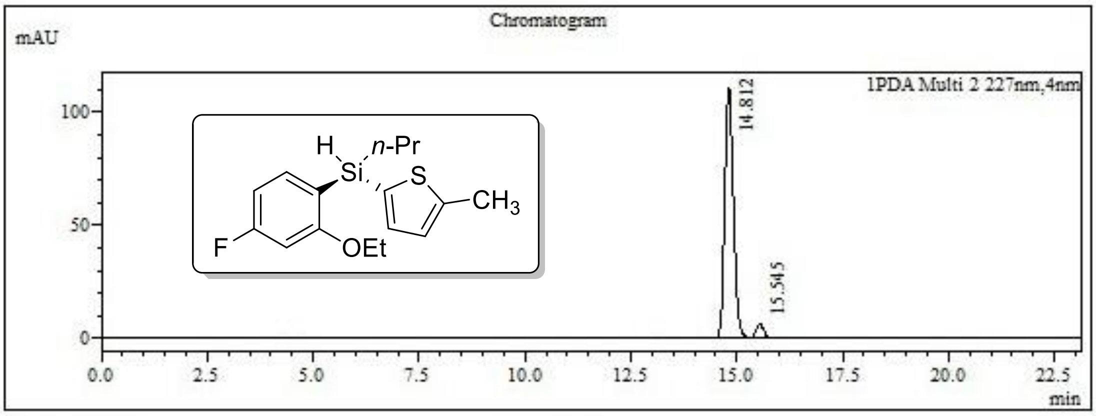

Peak Table

PDA Ch2 227nm

Peak#	Ret. Time	Area	Area%
1	14.812	1553239	94.579
2	15.545	89027	5.421
总计		1642266	100.000

Supplementary Figure 386. HPLC trace of **3na**

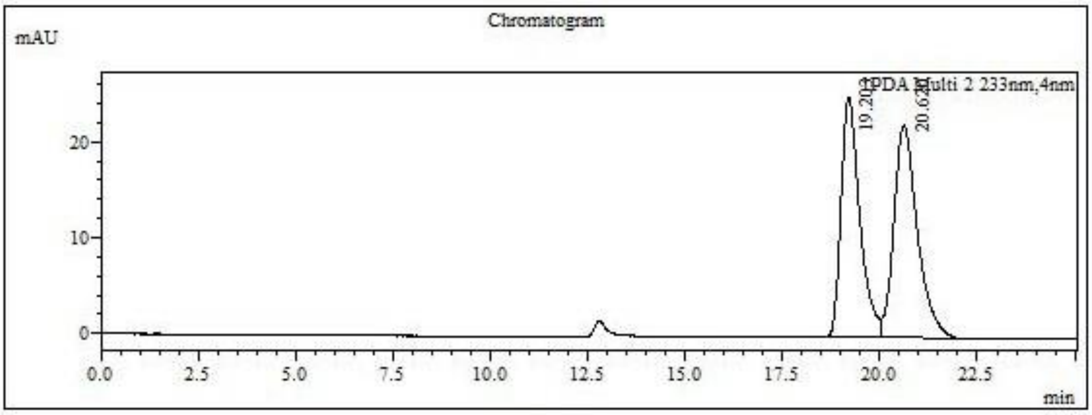

Peak Table

PDA Ch2 233nm

Peak#	Ret. Time	Area	Area%
1	19.203	743038	49.400
2	20.620	761082	50.600
总计		1504120	100.000

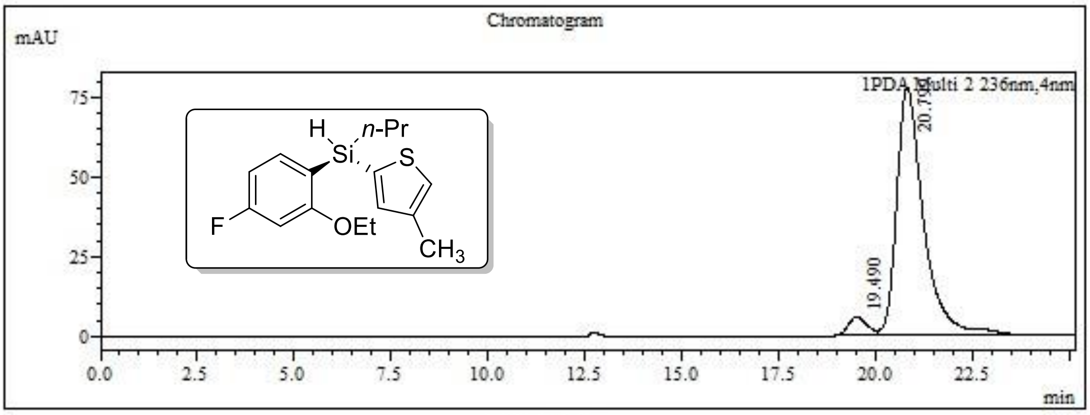

Peak Table

PDA Ch2 236nm

Peak#	Ret. Time	Area	Area%
1	19.490	198176	4.998
2	20.799	3766988	95.002
总计		3965165	100.000

Supplementary Figure 387. HPLC trace of **3ni**

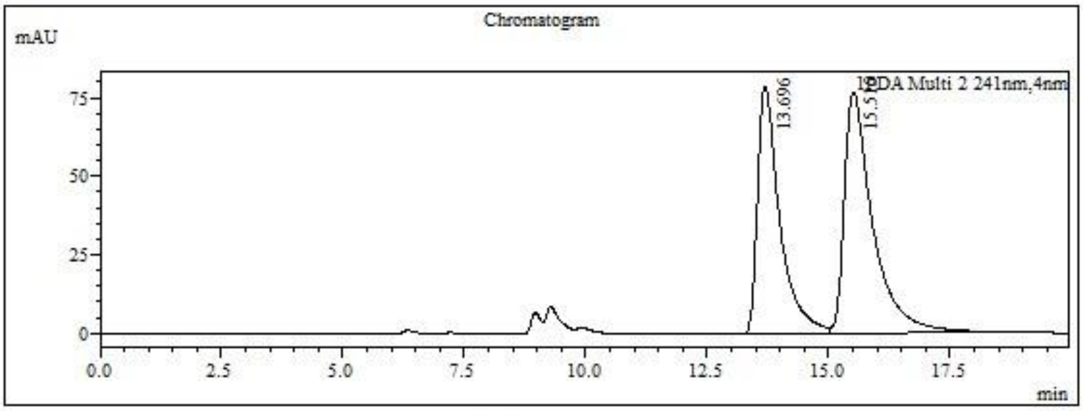

Peak Table

PDA Ch2 241nm

Peak#	Ret. Time	Area	Area%
1	13.696	2211911	49.389
2	15.515	2266679	50.611
总计		4478590	100.000

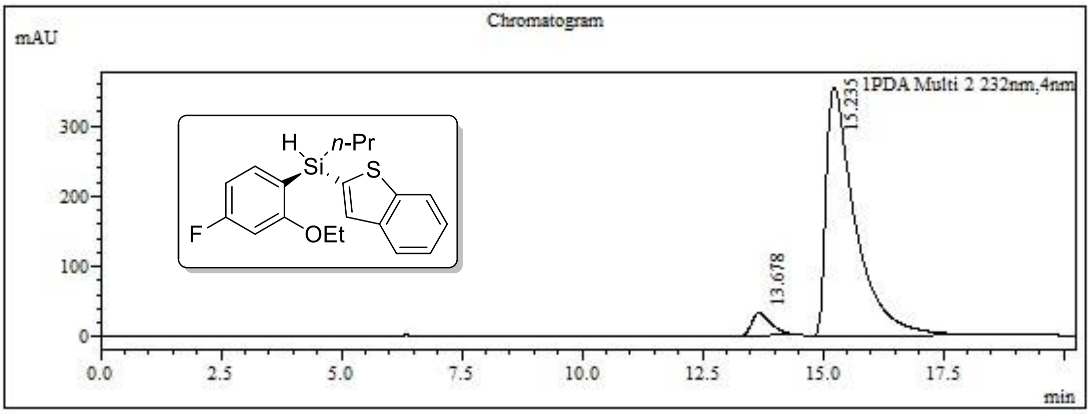

Peak Table

PDA Ch2 232nm

Peak#	Ret. Time	Area	Area%
1	13.678	945367	5.922
2	15.235	15017785	94.078
总计		15963152	100.000

Supplementary Figure 388. HPLC trace of **3nn**

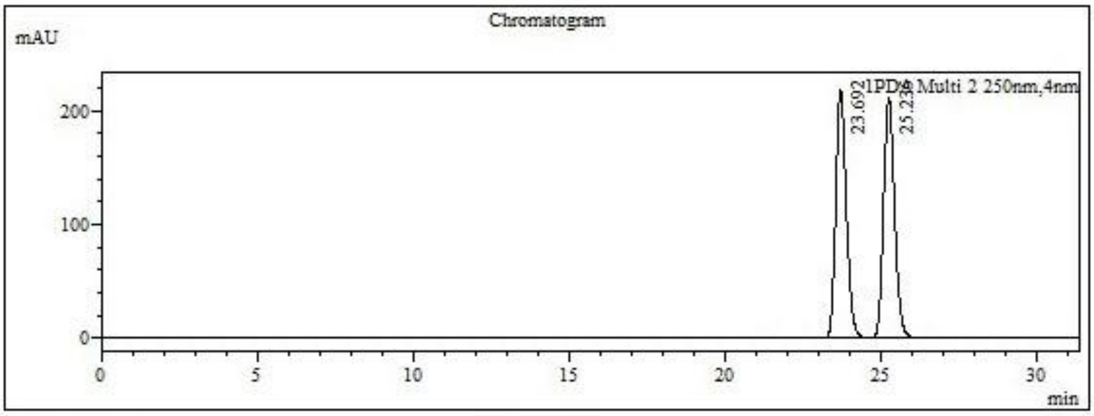

Peak Table

PDA Ch2 250nm

Peak#	Ret. Time	Area	Area%
1	23.692	5122687	49.735
2	25.235	5177291	50.265
总计		10299978	100.000

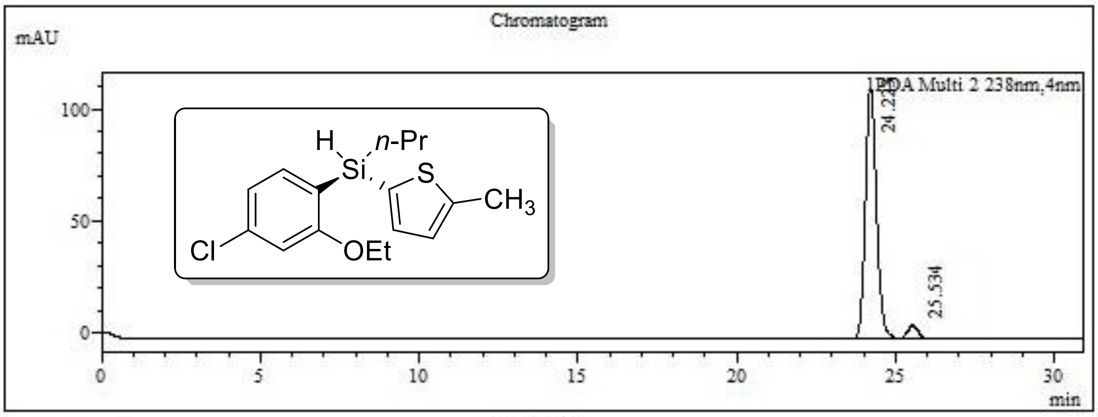

Peak Table

PDA Ch2 238nm

Peak#	Ret. Time	Area	Area%
1	24.223	2692251	95.576
2	25.534	124609	4.424
总计		2816860	100.000

Supplementary Figure 389. HPLC trace of **3oa**

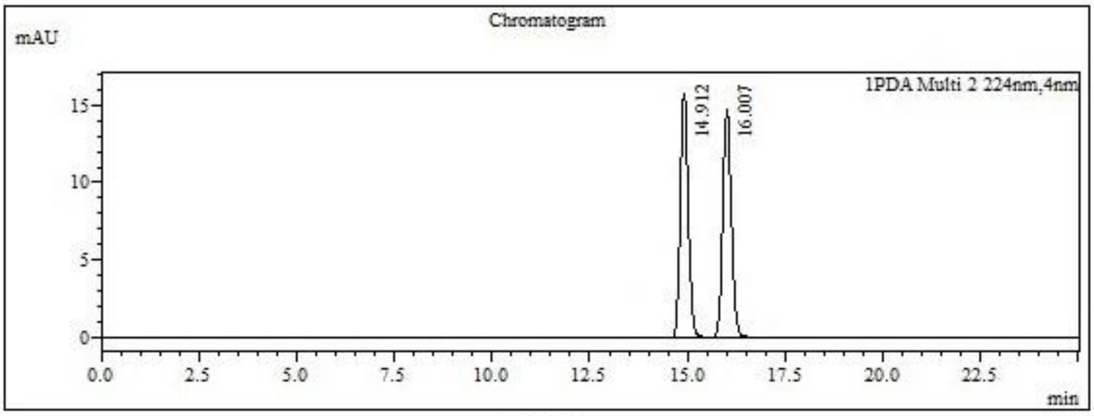

Peak Table

PDA Ch2 224nm

Peak#	Ret. Time	Area	Area%
1	14.912	221088	50.240
2	16.007	218972	49.760
总计		440060	100.000

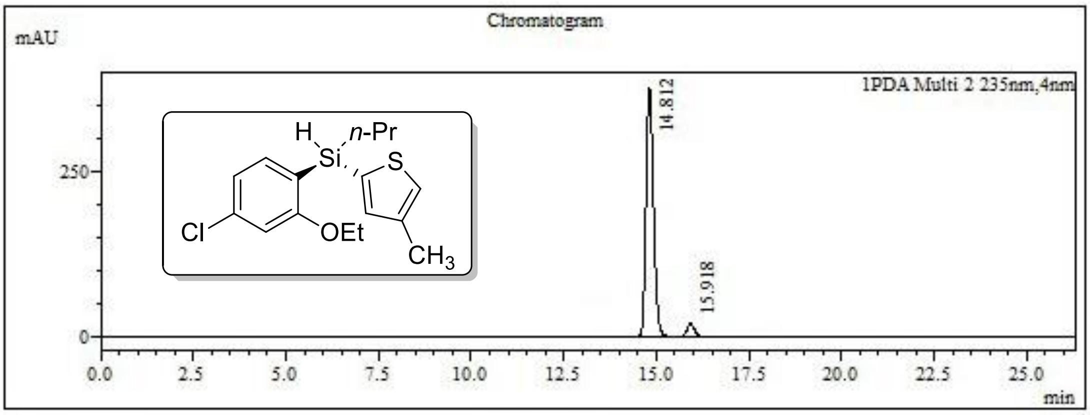

Peak Table

PDA Ch2 235nm

Peak#	Ret. Time	Area	Area%
1	14.812	5324074	95.148
2	15.918	271478	4.852
总计		5595552	100.000

Supplementary Figure 390. HPLC trace of **3oi**

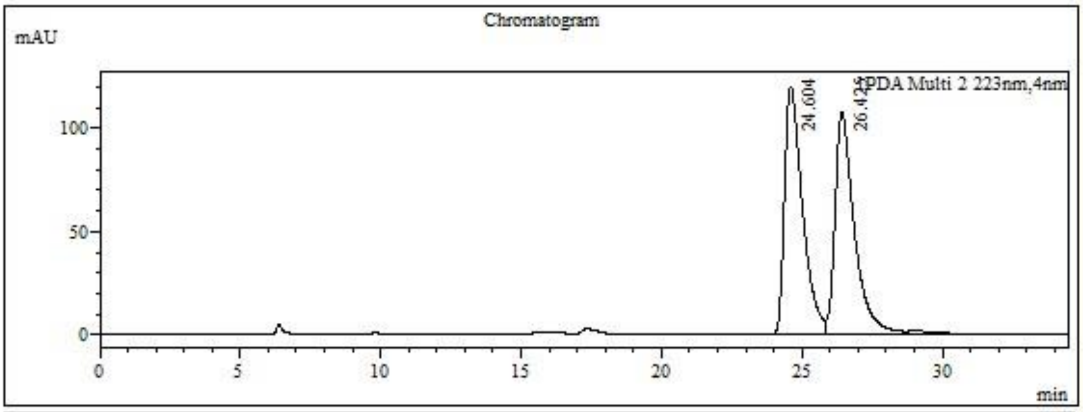

Peak Table

PDA Ch2 223nm

Peak#	Ret. Time	Area	Area%
1	24.604	5362756	50.342
2	26.425	5289970	49.658
总计		10652726	100.000

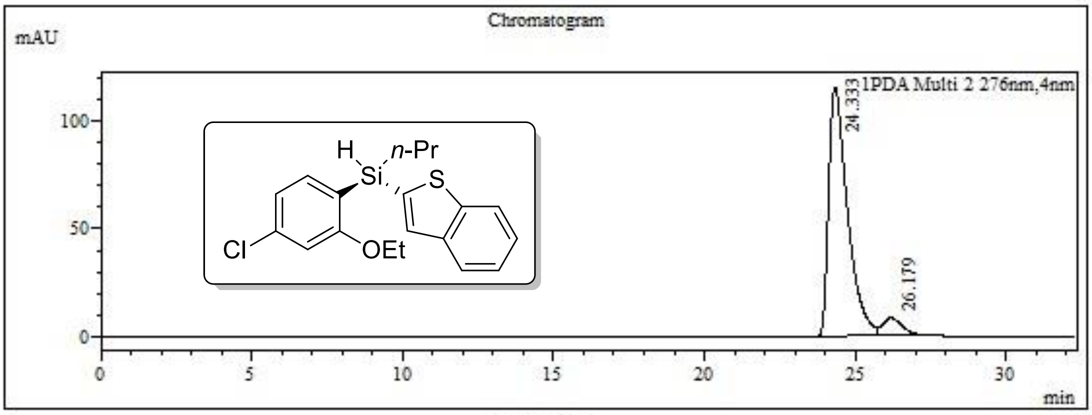

Peak Table

PDA Ch2 276nm

Peak#	Ret. Time	Area	Area%
1	24.333	4970539	93.656
2	26.179	336716	6.344
总计		5307255	100.000

Supplementary Figure 391. HPLC trace of **3on**

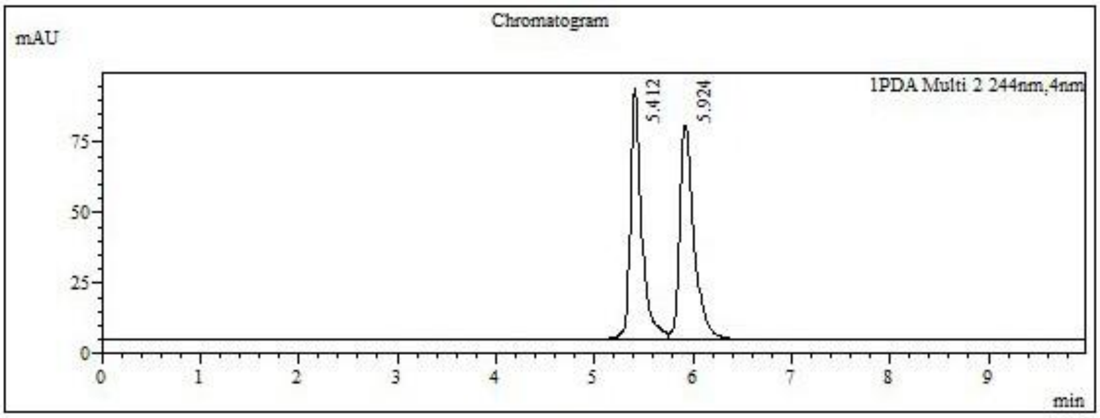

Peak Table

PDA Ch2 244nm

Peak#	Ret. Time	Area	Area%
1	5.412	739186	49.321
2	5.924	759525	50.679
总计		1498711	100.000

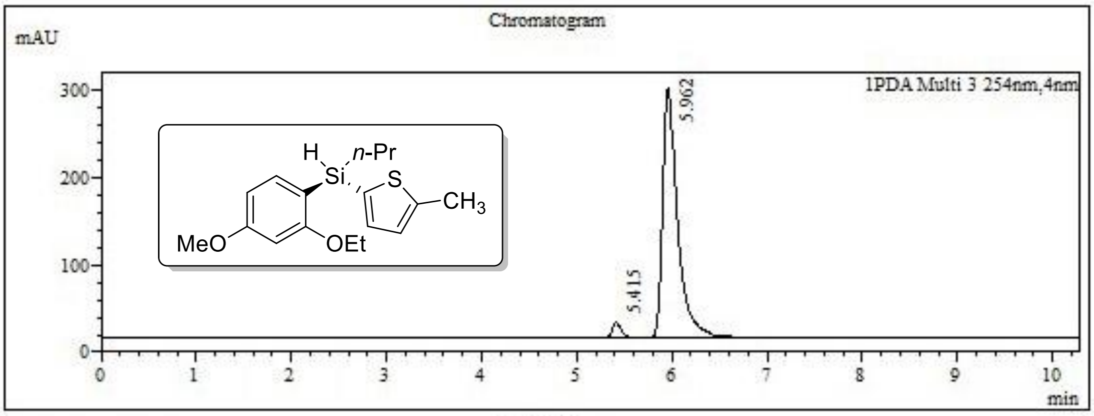

Peak Table

PDA Ch3 254nm

Peak#	Ret. Time	Area	Area%
1	5.415	139281	3.803
2	5.962	3522939	96.197
总计		3662220	100.000

Supplementary Figure 392. HPLC trace of **3pa**

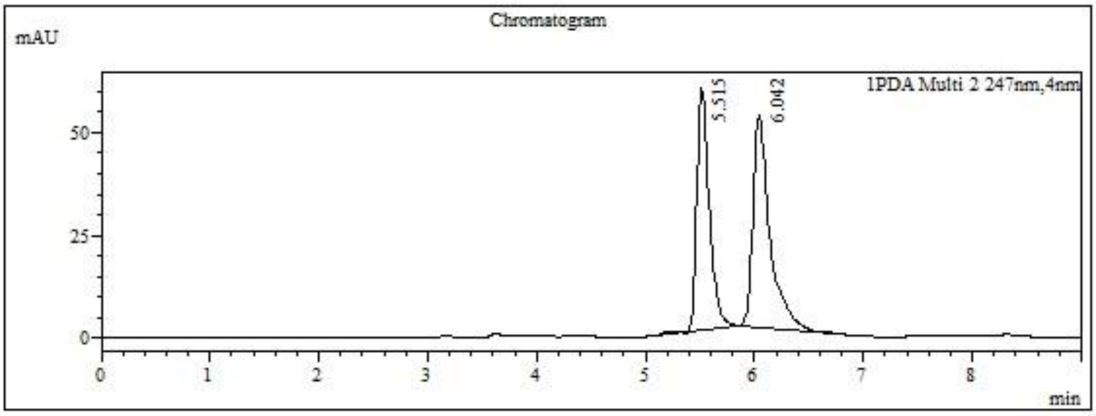

Peak Table

PDA Ch2 247nm

Peak#	Ret. Time	Area	Area%
1	5.515	462636	50.178
2	6.042	459347	49.822
总计		921983	100.000

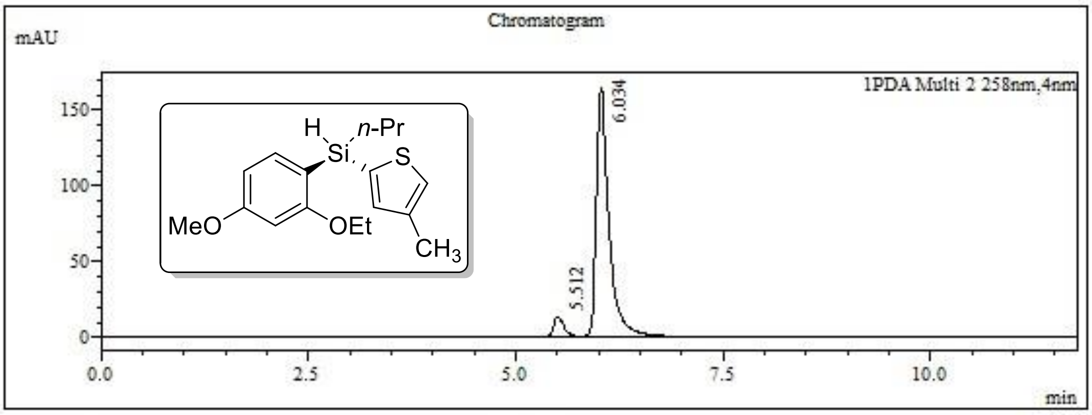

Peak Table

PDA Ch2 258nm

Peak#	Ret. Time	Area	Area%
1	5.512	108663	5.922
2	6.034	1726279	94.078
总计		1834942	100.000

Supplementary Figure 393. HPLC trace of **3pi**

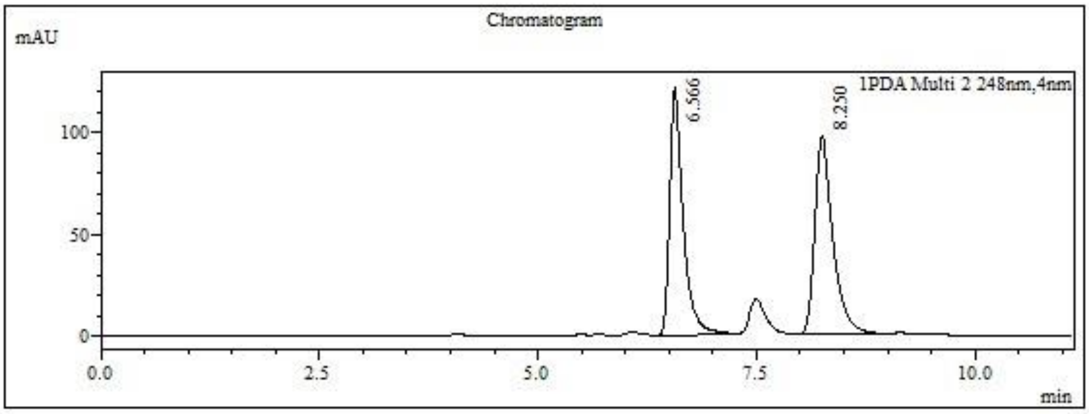

Peak Table

PDA Ch2 248nm

Peak#	Ret. Time	Area	Area%
1	6.566	1318953	49.905
2	8.250	1323964	50.095
总计		2642917	100.000

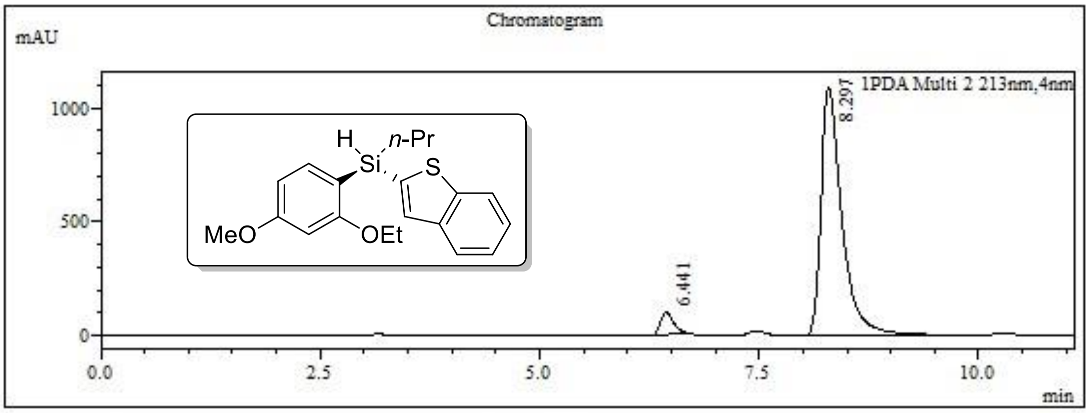

Peak Table

PDA Ch2 213nm

Peak#	Ret. Time	Area	Area%
1	6.441	1041793	5.437
2	8.297	18118278	94.563
总计		19160072	100.000

Supplementary Figure 394. HPLC trace of **3pn**

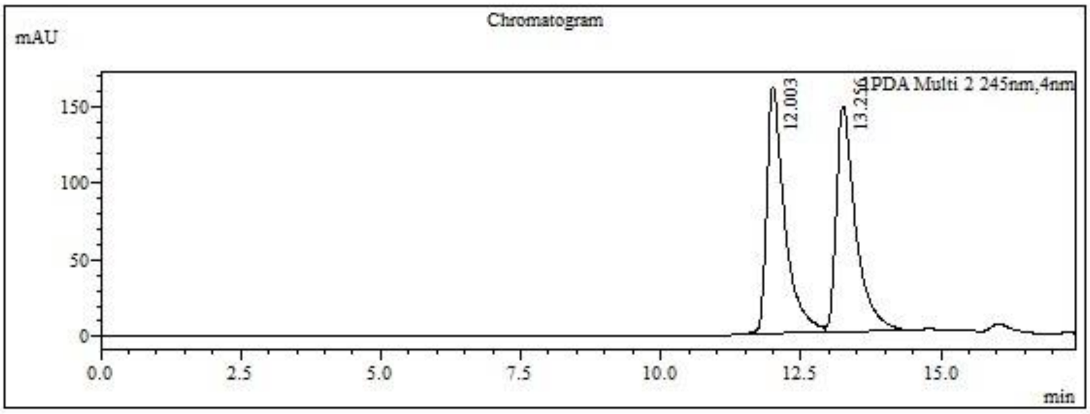

Peak Table

PDA Ch2 245nm

Peak#	Ret. Time	Area	Area%
1	12.003	3613374	50.197
2	13.256	3585055	49.803
总计		7198429	100.000

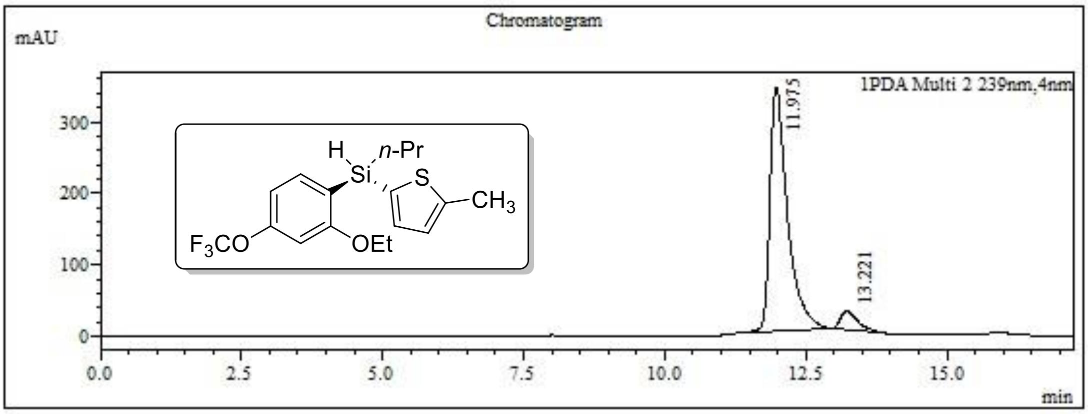

Peak Table

PDA Ch2 239nm

Peak#	Ret. Time	Area	Area%
1	11.975	7355901	93.079
2	13.221	546958	6.921
总计		7902859	100.000

Supplementary Figure 395. HPLC trace of **3qa**

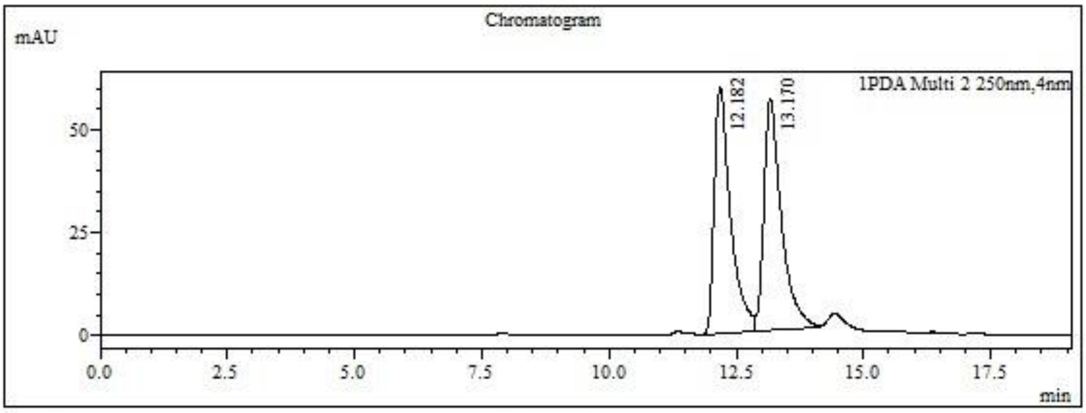

Peak Table

PDA Ch2 250nm

Peak#	Ret. Time	Area	Area%
1	12.182	1329217	50.013
2	13.170	1328548	49.987
总计		2657766	100.000

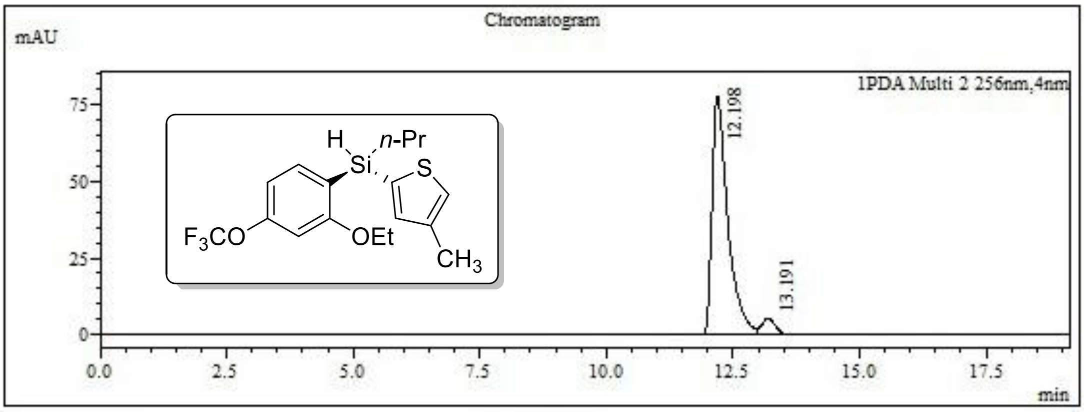

Peak Table

PDA Ch2 256nm

Peak#	Ret. Time	Area	Area%
1	12.198	1772635	94.052
2	13.191	112098	5.948
总计		1884733	100.000

Supplementary Figure 396. HPLC trace of **3qi**

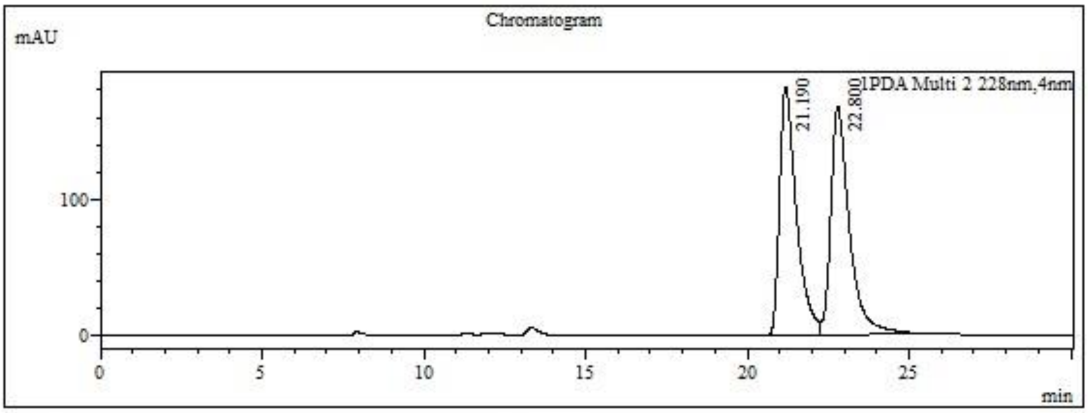

Peak Table

PDA Ch2 228nm

Peak#	Ret. Time	Area	Area%
1	21.190	6631148	49.929
2	22.800	6650117	50.071
总计		13281265	100.000

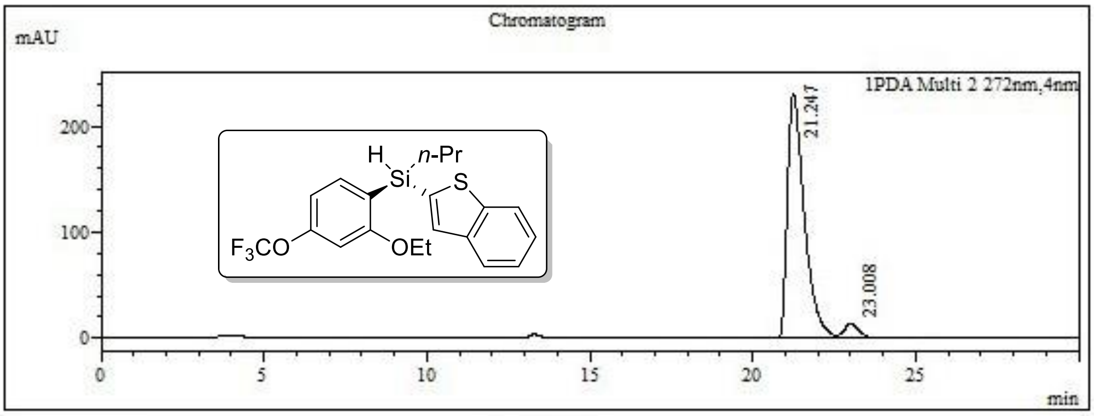

Peak Table

PDA Ch2 272nm

Peak#	Ret. Time	Area	Area%
1	21.247	8470420	95.517
2	23.008	397565	4.483
总计		8867985	100.000

Supplementary Figure 397. HPLC trace of **3qn**

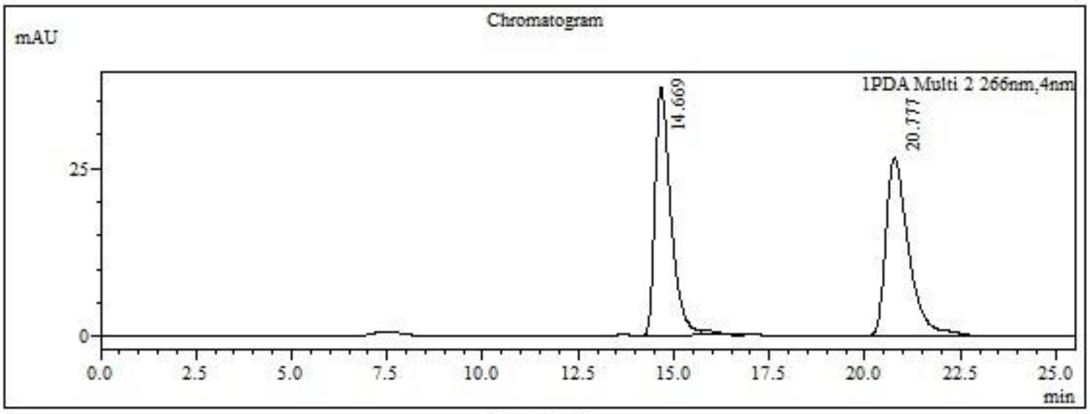

Peak Table

PDA Ch2 266nm

Peak#	Ret. Time	Area	Area%
1	14.669	1076121	49.351
2	20.777	1104406	50.649
总计		2180527	100.000

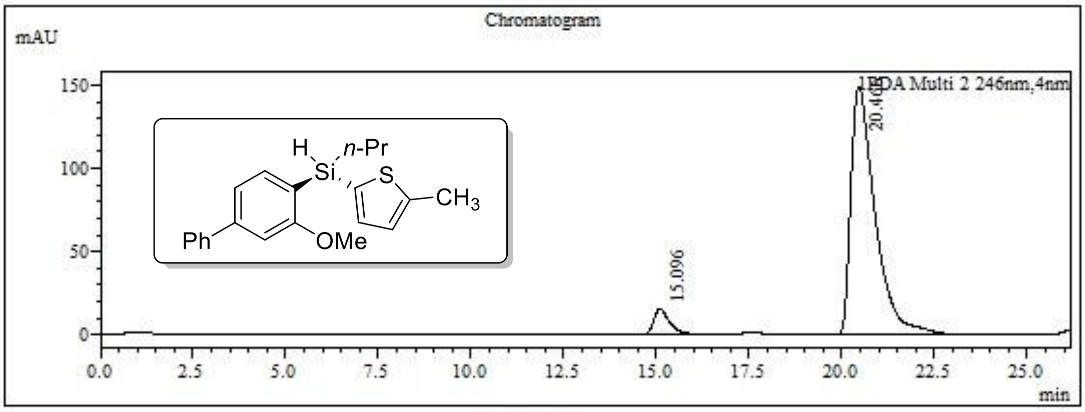

Peak Table

PDA Ch2 246nm

Peak#	Ret. Time	Area	Area%
1	15.096	430138	5.954
2	20.466	6794085	94.046
总计		7224223	100.000

Supplementary Figure 398. HPLC trace of **3ra**

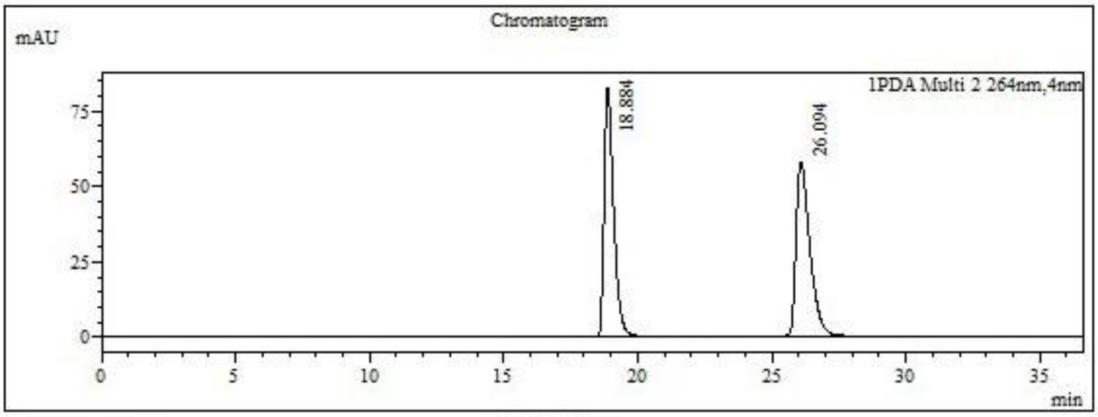

Peak Table

PDA Ch2 264nm

Peak#	Ret. Time	Area	Area%
1	18.884	2012910	49.471
2	26.094	2055962	50.529
总计		4068872	100.000

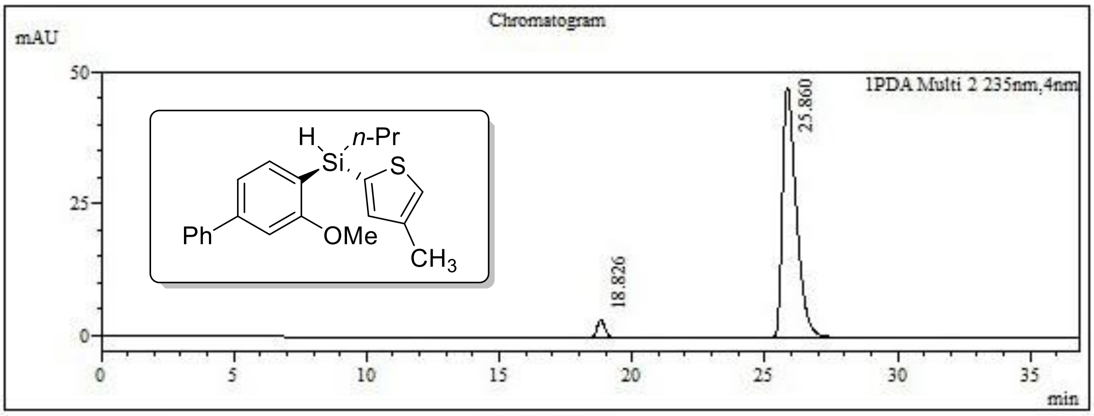

Peak Table

PDA Ch2 235nm

Peak#	Ret. Time	Area	Area%
1	18.826	77557	4.291
2	25.860	1729907	95.709
总计		1807464	100.000

Supplementary Figure 399. HPLC trace of **3ri**

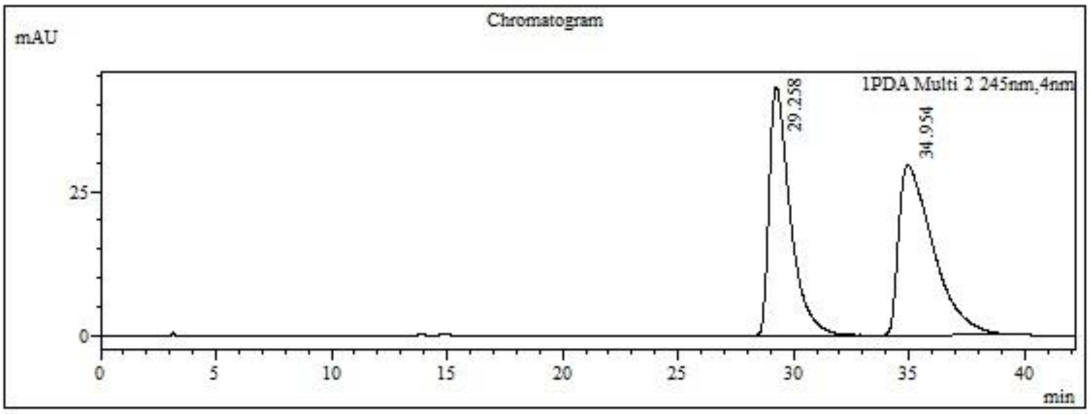

Peak Table

PDA Ch2 245nm

Peak#	Ret. Time	Area	Area%
1	29.258	2724271	49.786
2	34.954	2747744	50.214
总计		5472015	100.000

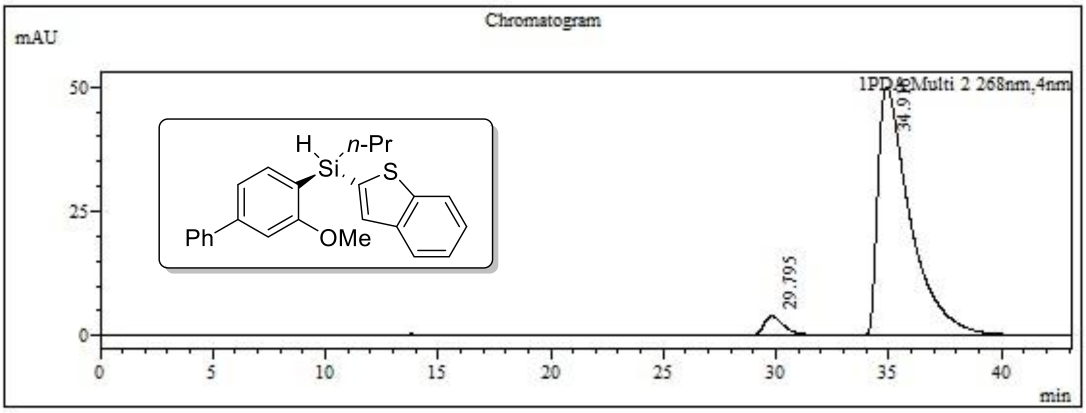

Peak Table

PDA Ch2 268nm

Peak#	Ret. Time	Area	Area%
1	29.795	229904	4.410
2	34.916	4983586	95.590
总计		5213490	100.000

Supplementary Figure 400. HPLC trace of **3rn**

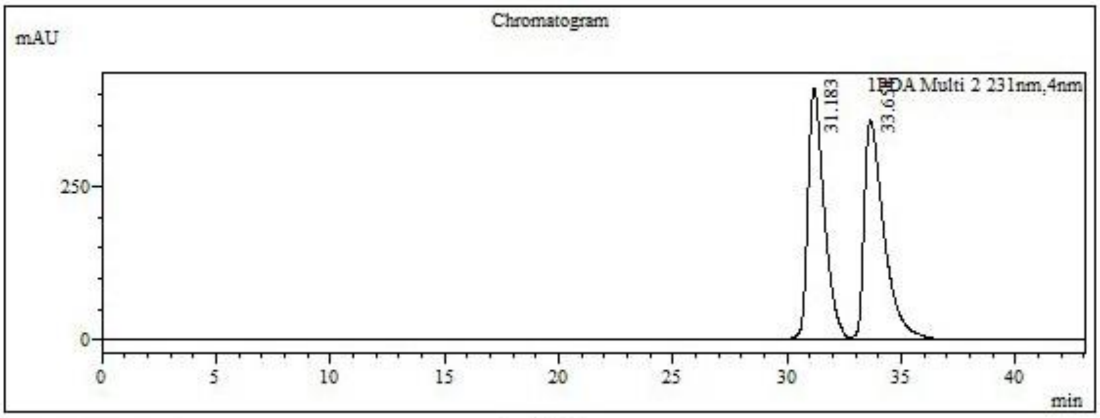

Peak Table

PDA Ch2 231nm

Peak#	Ret. Time	Area	Area%
1	31.183	19696687	49.773
2	33.654	19876417	50.227
总计		39573104	100.000

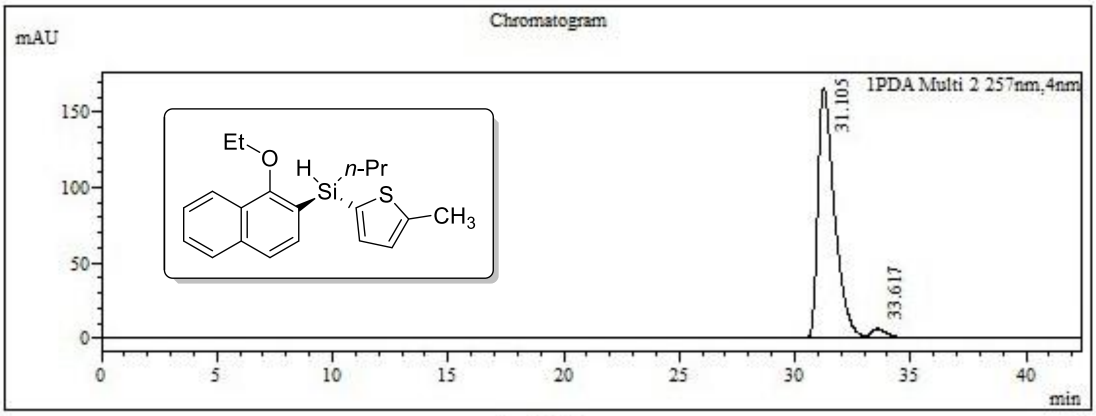

Peak Table

PDA Ch2 257nm

Peak#	Ret. Time	Area	Area%
1	31.105	8238057	96.849
2	33.617	268044	3.151
总计		8506101	100.000

Supplementary Figure 401. HPLC trace of **3sa**

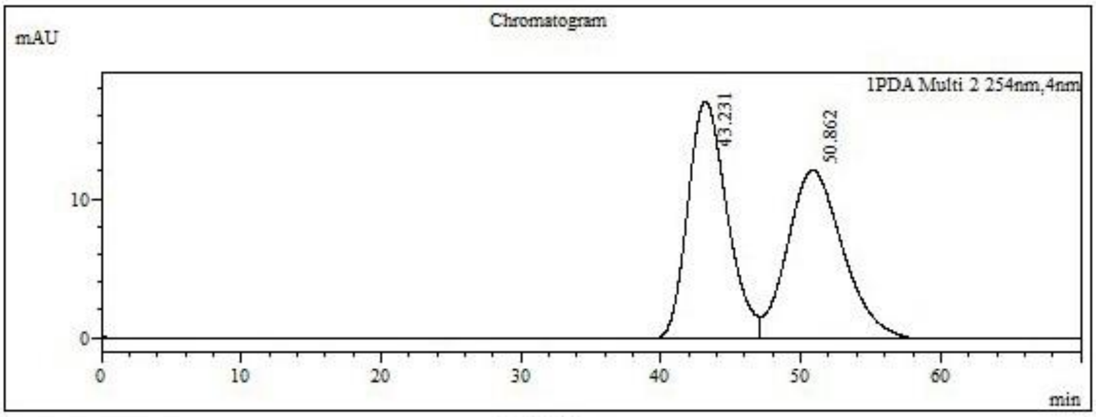

Peak Table

PDA Ch2 254nm

Peak#	Ret. Time	Area	Area%
1	43.231	3302168	50.194
2	50.862	3276582	49.806
总计		6578751	100.000

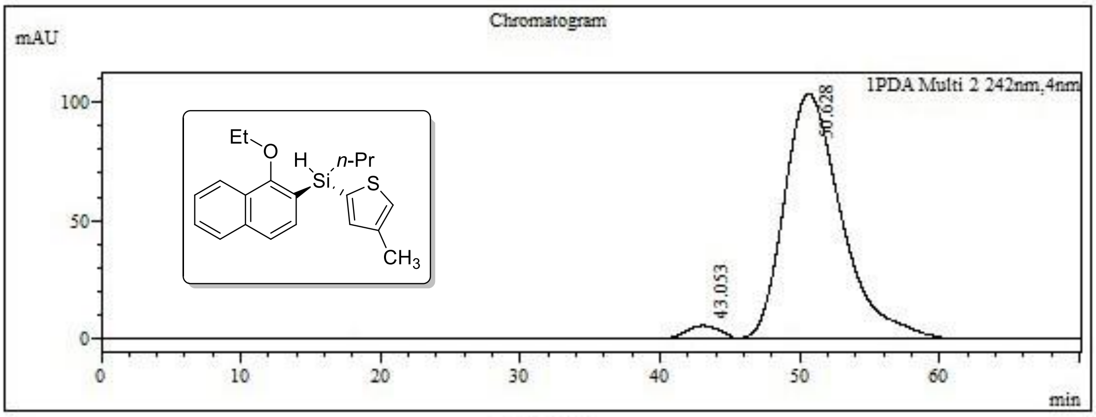

Peak Table

PDA Ch2 242nm

Peak#	Ret. Time	Area	Area%
1	43.053	968175	3.174
2	50.628	29531001	96.826
总计		30499176	100.000

Supplementary Figure 402. HPLC trace of **3si**

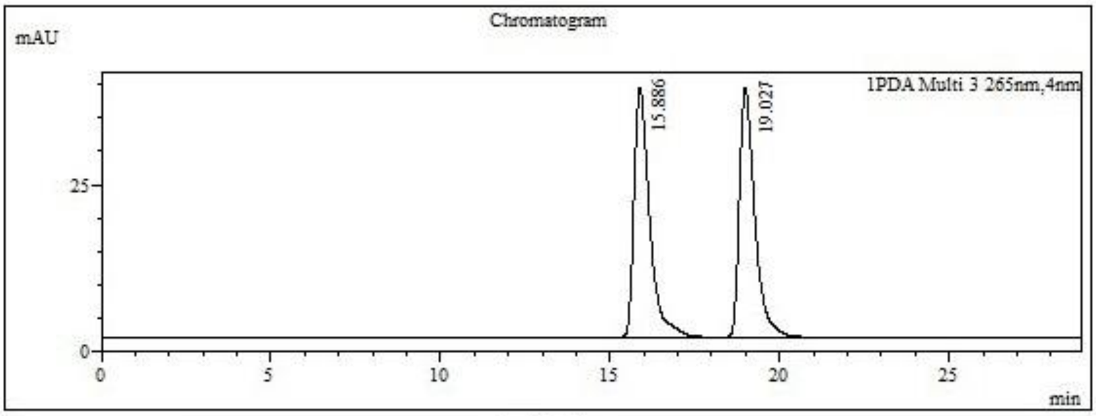

Peak Table

PDA Ch3 265nm

Peak#	Ret. Time	Area	Area%
1	15.886	86223	49.614
2	19.027	87565	50.386
总计		173788	100.000

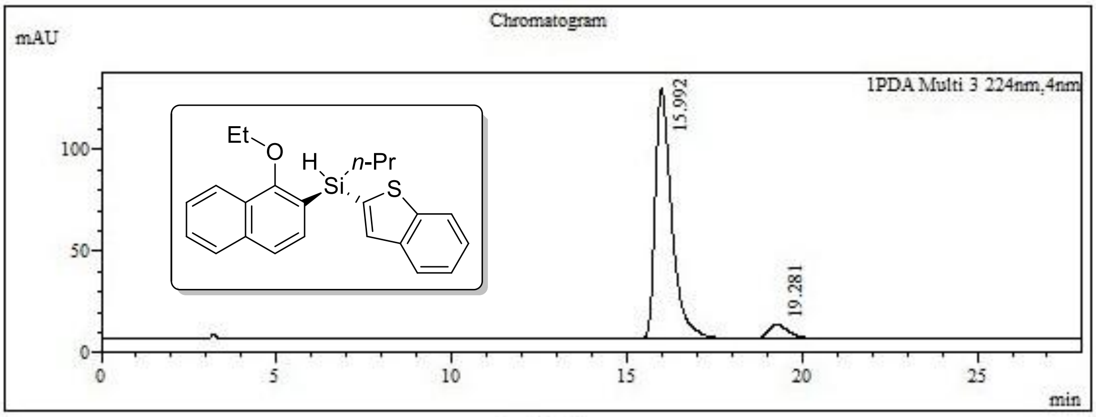

Peak Table

PDA Ch3 224nm

Peak#	Ret. Time	Area	Area%
1	15.992	4579418	94.375
2	19.281	272964	5.625
总计		4852382	100.000

Supplementary Figure 403. HPLC trace of **3sn**

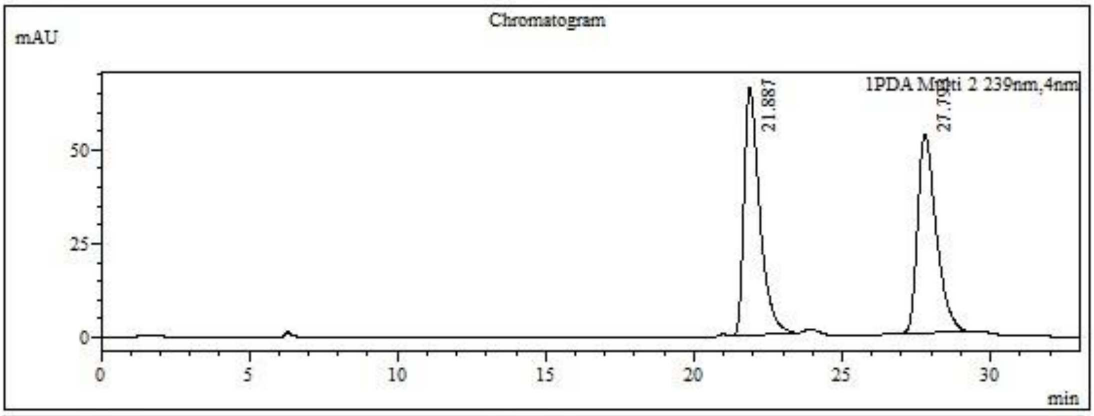

Peak Table

PDA Ch2 239nm

Peak#	Ret. Time	Area	Area%
1	21.887	2423060	50.738
2	27.793	2352555	49.262
总计		4775616	100.000

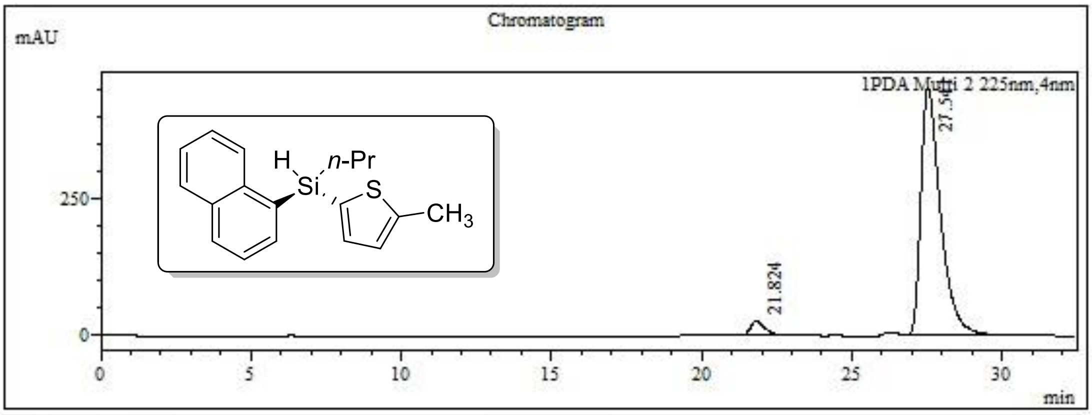

Peak Table

PDA Ch2 225nm

Peak#	Ret. Time	Area	Area%
1	21.824	837360	3.859
2	27.541	20863217	96.141
总计		21700577	100.000

Supplementary Figure 404. HPLC trace of **3ta**

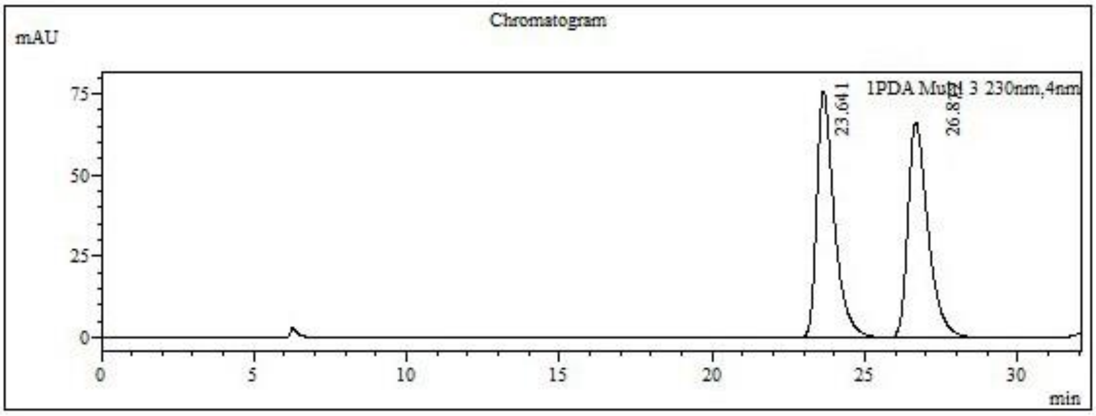

Peak Table

PDA Ch3 230nm

Peak#	Ret. Time	Area	Area%
1	23.641	3107242	50.607
2	26.872	3032729	49.393
总计		6139971	100.000

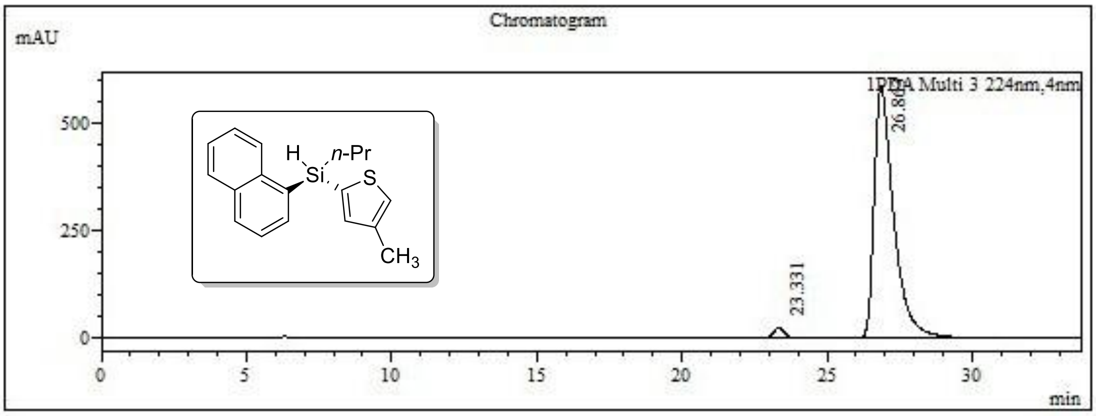

Peak Table

PDA Ch3 224nm

Peak#	Ret. Time	Area	Area%
1	23.331	589192	2.085
2	26.863	27665080	97.915
总计		28254272	100.000

Supplementary Figure 405. HPLC trace of **3ti**

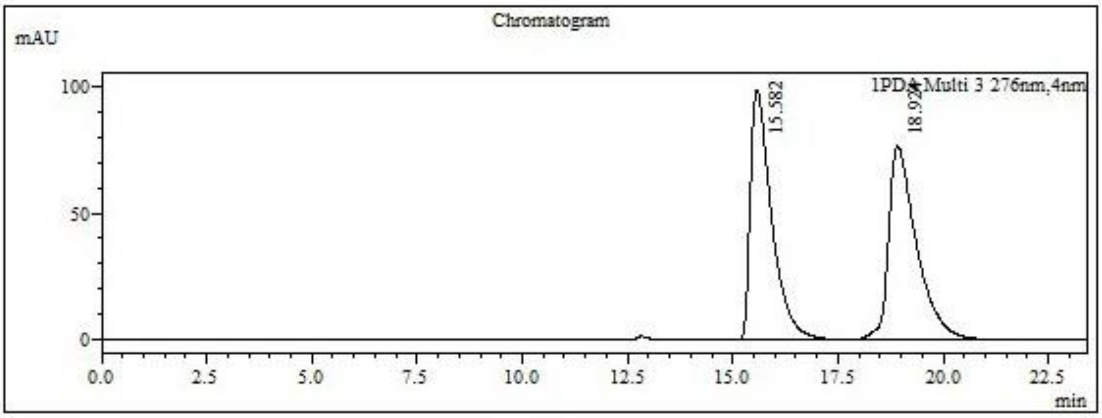

Peak Table

PDA Ch3 276nm

Peak#	Ret. Time	Area	Area%
1	15.582	3502874	50.325
2	18.924	3457615	49.675
总计		6960490	100.000

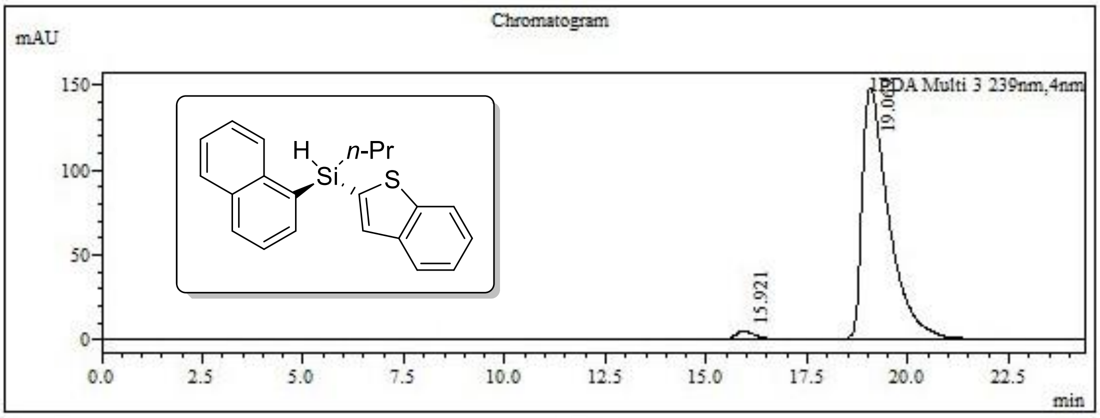

Peak Table

PDA Ch3 239nm

Peak#	Ret. Time	Area	Area%
1	15.921	147662	2.099
2	19.069	6886469	97.901
总计		7034131	100.000

Supplementary Figure 406. HPLC trace of **3tn**

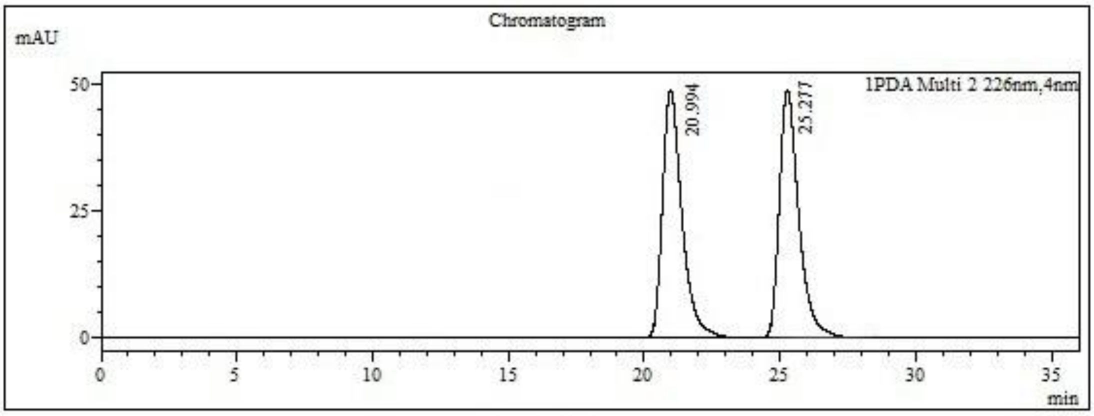

Peak Table

PDA Ch2 226nm

Peak#	Ret. Time	Area	Area%
1	20.994	260969	50.796
2	25.277	252789	49.204
总计		513758	100.000

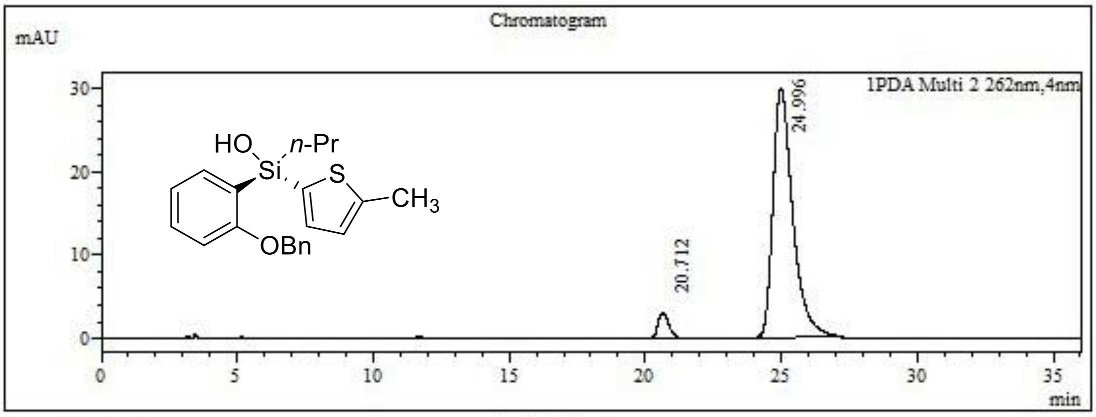

Peak Table

PDA Ch2 262nm

Peak#	Ret. Time	Area	Area%
1	20.712	79547	5.002
2	24.996	1510671	94.998
总计		1590219	100.000

Supplementary Figure 407. HPLC trace of **3aa-1**

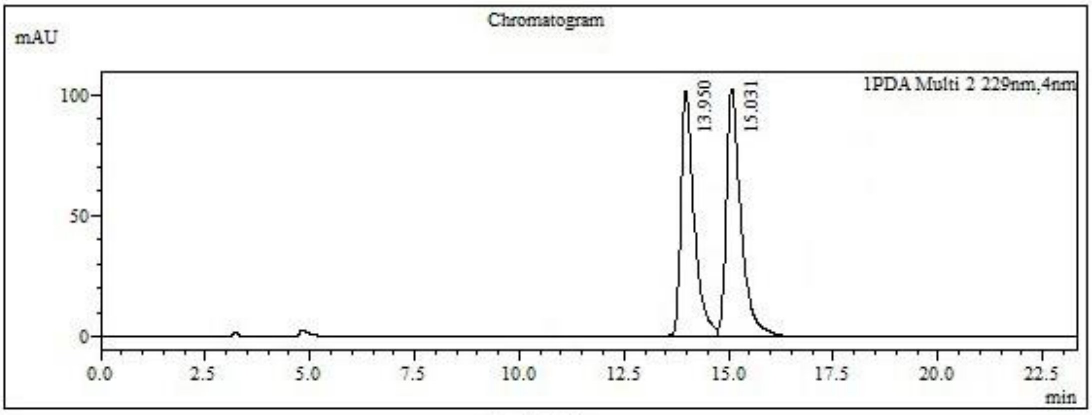

Peak Table

PDA Ch2 229nm

Peak#	Ret. Time	Area	Area%
1	13.950	118629	49.504
2	15.031	121005	50.496
总计		239634	100.000

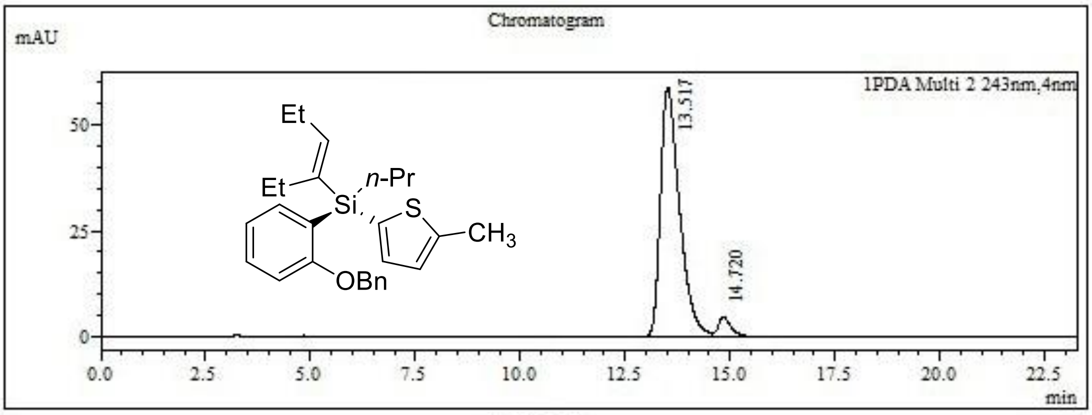

Peak Table

PDA Ch2 243nm

Peak#	Ret. Time	Area	Area%
1	13.517	1727031	94.672
2	14.720	97189	5.328
总计		1824220	100.000

Supplementary Figure 408. HPLC trace of **3aa-2**

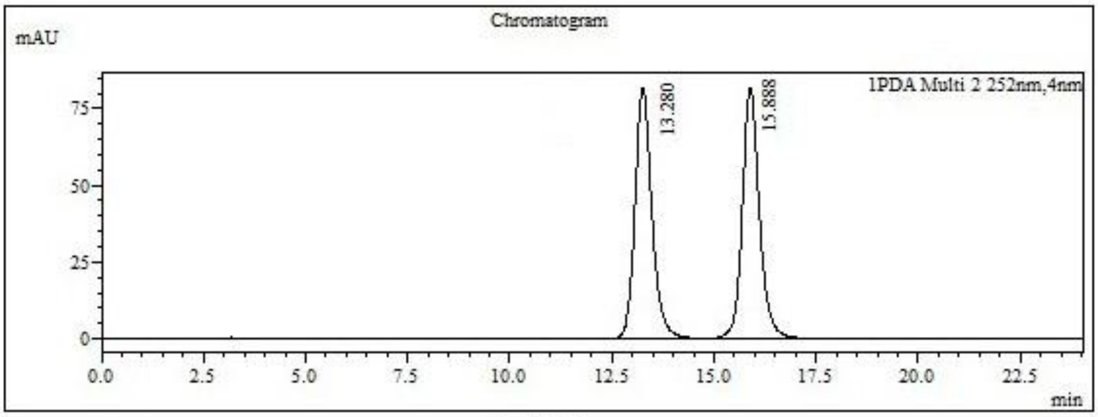

Peak Table

PDA Ch2 252nm

Peak#	Ret. Time	Area	Area%
1	13.280	1218556	49.159
2	15.888	1260252	50.841
总计		2478808	100.000

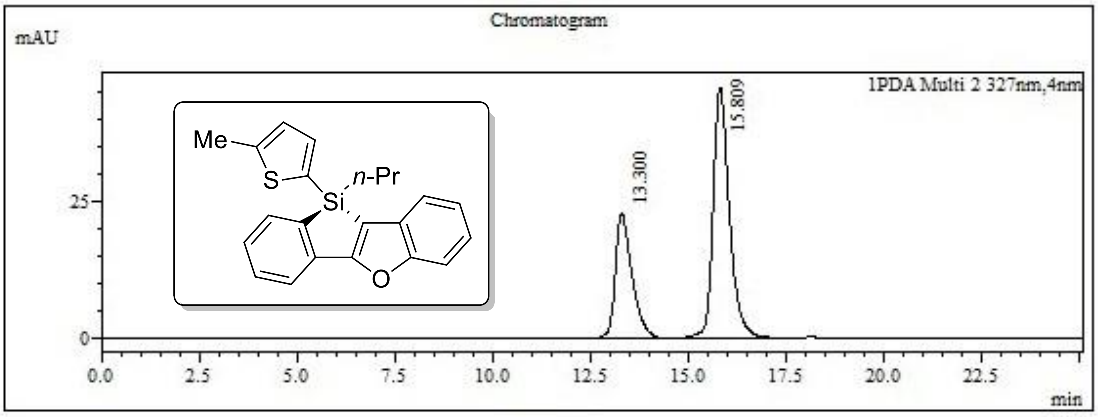

Peak Table

PDA Ch2 327nm

Peak#	Ret. Time	Area	Area%
1	13.300	684873	34.257
2	15.809	1314368	65.743
总计		1999241	100.000

Supplementary Figure 409. HPLC trace of **3va**

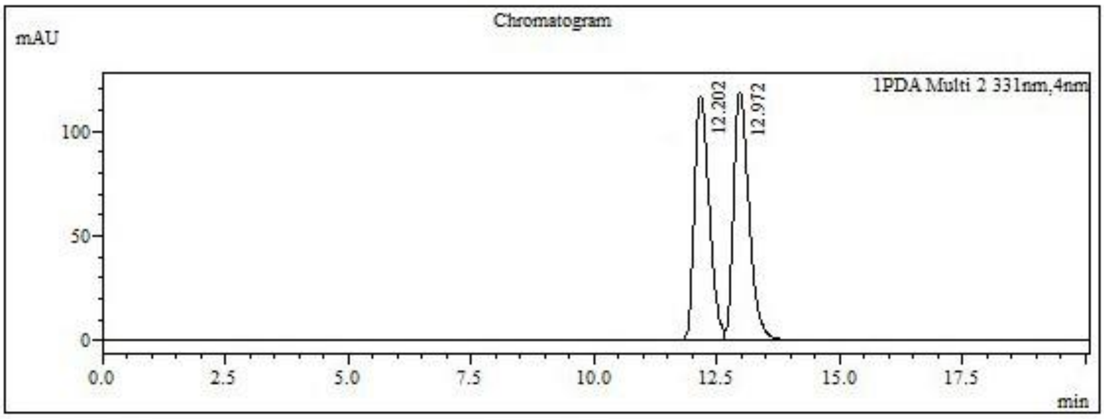

Peak Table

PDA Ch2 331nm

Peak#	Ret. Time	Area	Area%
1	12.202	712797	50.890
2	12.972	687856	49.110
总计		1400653	100.000

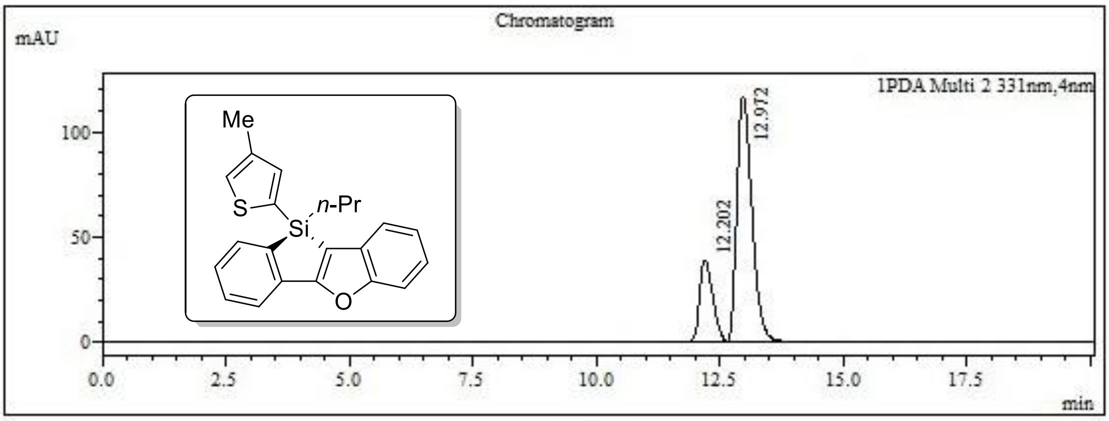

Peak Table

PDA Ch2 331nm

Peak#	Ret. Time	Area	Area%
1	12.202	712797	22.233
2	12.972	2493282	77.767
总计		3206079	100.000

Supplementary Figure 410. HPLC trace of **3vi**

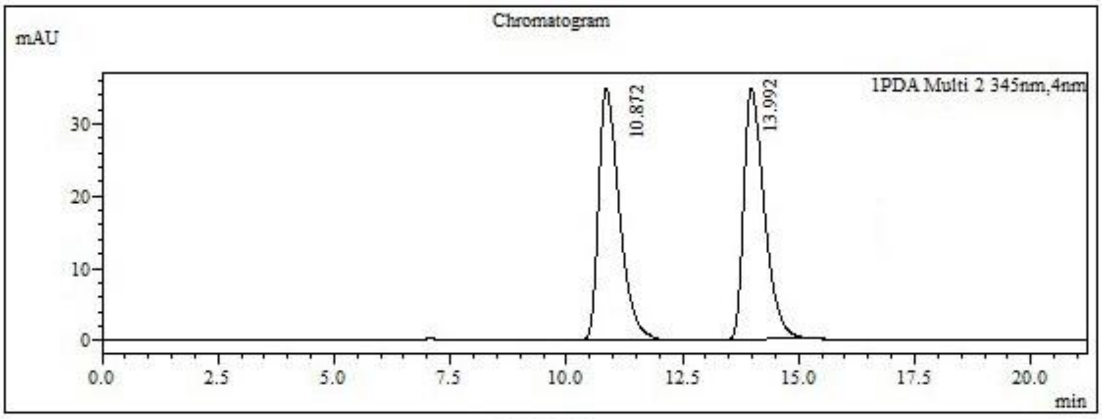

Peak Table

PDA Ch2 345nm

Peak#	Ret. Time	Area	Area%
1	10.872	404578	40.585
2	13.992	592296	59.415
总计		996874	100.000

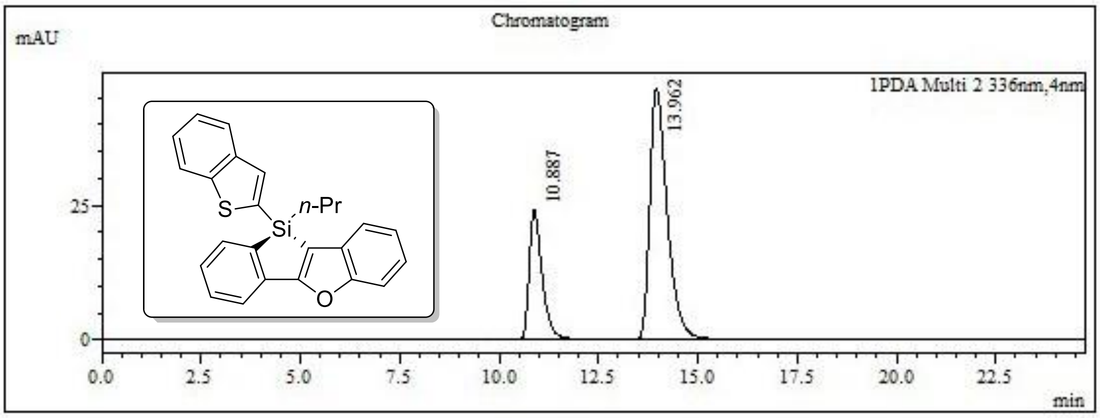

Peak Table

PDA Ch2 336nm

Peak#	Ret. Time	Area	Area%
1	10.887	529375	27.160
2	13.962	1419746	72.840
总计		1949122	100.000

Supplementary Figure 411. HPLC trace of **3vn**

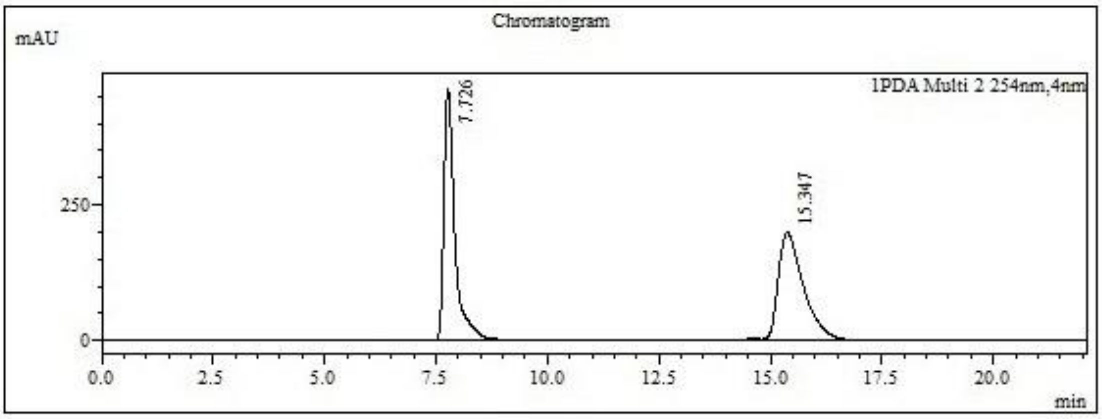

Peak Table

PDA Ch2 254nm

Peak#	Ret. Time	Area	Area%
1	7.726	7804086	49.862
2	15.347	7847256	50.138
总计		15651342	100.000

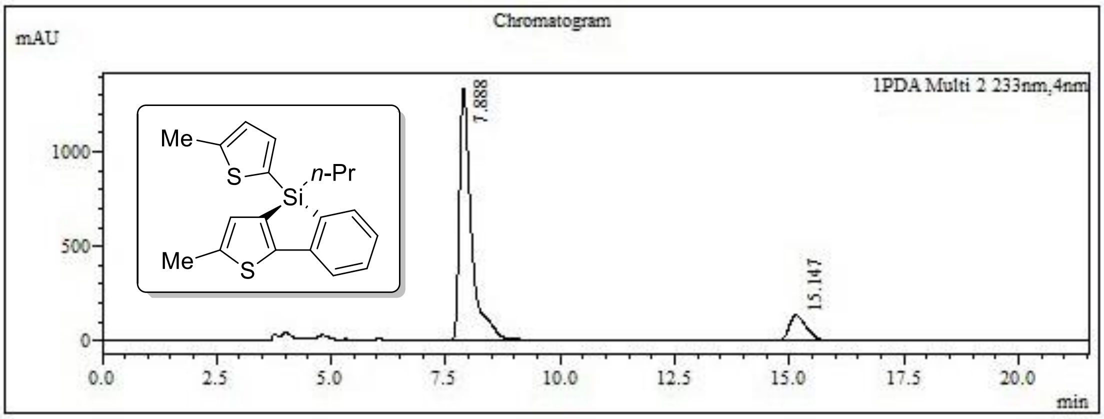

Peak Table

PDA Ch2 233nm

Peak#	Ret. Time	Area	Area%
1	7.888	24326791	87.469
2	15.147	3485119	12.531
总计		27811910	100.000

Supplementary Figure 412. HPLC trace of **3wa**

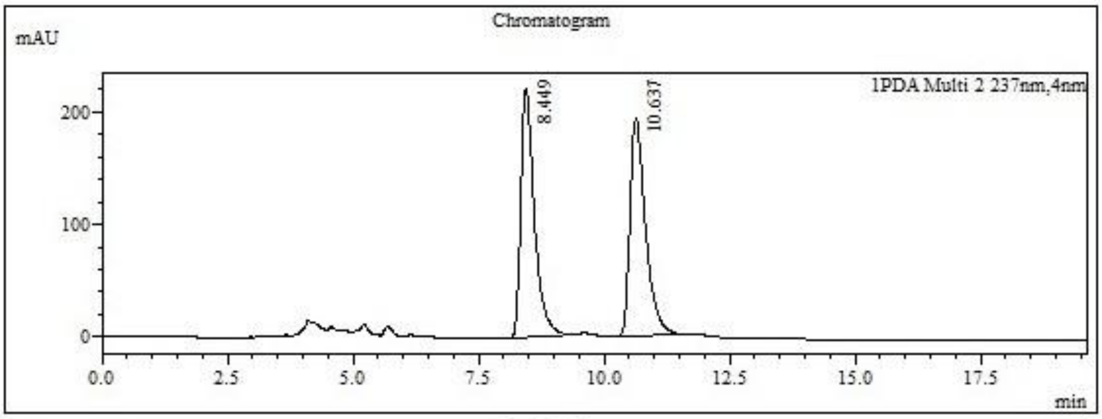

Peak Table

PDA Ch2 237nm

Peak#	Ret. Time	Area	Area%
1	8.449	4139488	50.069
2	10.637	4128043	49.931
总计		8267531	100.000

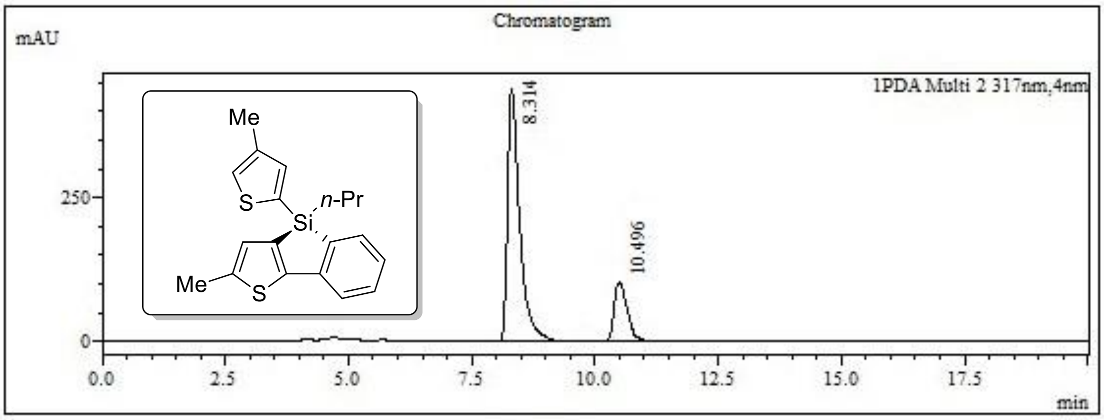

Peak Table

PDA Ch2 317nm

Peak#	Ret. Time	Area	Area%
1	8.314	7612788	80.608
2	10.496	1831425	19.392
总计		9444213	100.000

Supplementary Figure 413. HPLC trace of **3wi**

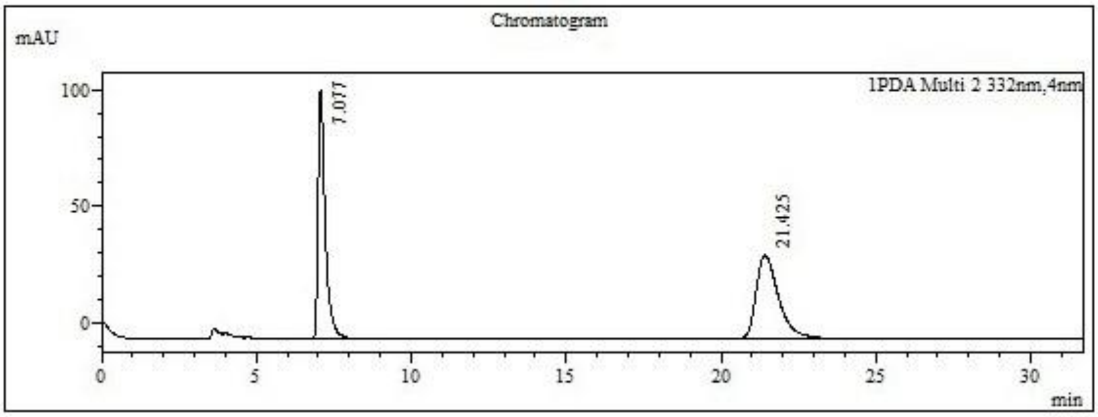

Peak Table

PDA Ch2 332nm

Peak#	Ret. Time	Area	Area%
1	7.077	1790515	49.471
2	21.425	1828791	50.529
总计		3619306	100.000

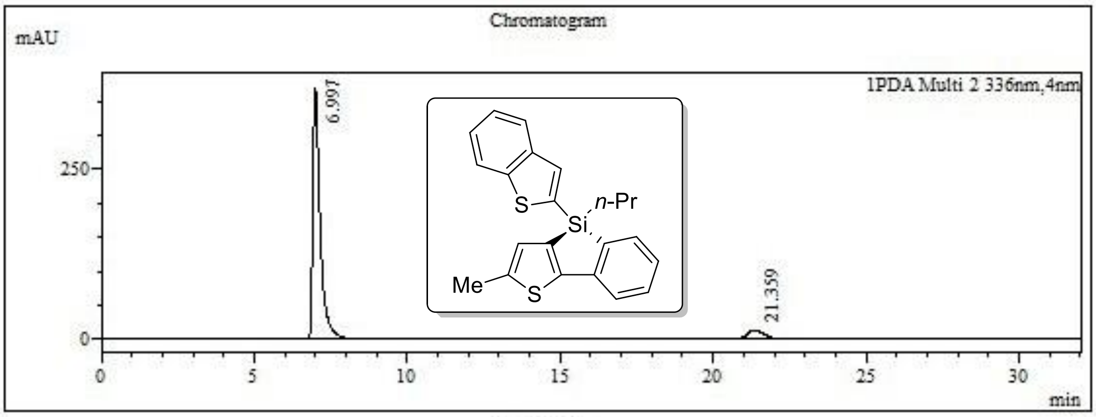

Peak Table

PDA Ch2 336nm

Peak#	Ret. Time	Area	Area%
1	6.997	6378932	92.914
2	21.359	486510	7.086
总计		6865442	100.000

Supplementary Figure 414. HPLC trace of **3wn**

4. Supplementary References

(1) Zhang, Q.-W.; An, K.; Liu, L.-C.; Yue, Y.; He, W. Rhodium-Catalyzed Enantioselective Intramolecular C−H Silylation for the Syntheses of Planar-Chiral Metallocene Siloles, *Angew. Chem., Int. Ed.* **2015**, *54*, 6918-6921.

(2) Sevov, C. S.; Hartwig, J. F. Iridium-Catalyzed Oxidative Olefination of Furans with Unactivated Alkenes. *J. Am. Chem. Soc.* **2014**, *136*, 10625−10631.

(3) Zhang, L.; An, K.; Wang, Y.; Wu Y.-D.; Zhang, X.; Yu, Z.-X.; He, W. A Combined Computational and Experimental Study of Rh-Catalyzed C−H Silylation with Silacyclobutanes: Insights Leading to a More Efficient Catalyst System. *J. Am. Chem. Soc.* **2021**, *143*, 3571-3582.

(4) Meißner, A.; Preetz, A.; Drexler, H.-J.; Baumann, W.; Spannenberg, A.; König, A.; Heller, D. In Situ Synthesis of Neutral Dinuclear Rhodium Diphosphine Complexes [{Rh(diphosphine)(μ_2-X)}$_2$]: Systematic Investigations. *ChemPlusChem* **2015**, *80*, 169-180.

(5) Sargent, A. L.; Titus, E. P. C-S and C-H Bond Activation of Thiophene by Cp*Rh(PMe_3): A DFT Theoretical Investigation. *Organometallics* **1998**, *17*, 65-77.
